# Pervasive summertime nitrous oxide undersaturation in U.S. lakes and reservoirs

**DOI:** 10.1038/s41467-026-74705-6

**Published:** 2026-07-11

**Authors:** Jake J. Beaulieu, Roy W. Martin, Michael G. McManus

**Affiliations:** US Environmental Protection Agency, Office of Research and Development, Cincinnati, OH USA

**Keywords:** Element cycles, Limnology

## Abstract

Lakes and reservoirs are estimated to be globally important sources of nitrous oxide (N_2_O) to the atmosphere but recent evidence of N_2_O uptake across a broad range of lakes have called the accuracy of emission estimates into question. Here, we use a new national-scale dataset of dissolved N_2_O concentration and a Bayesian hierarchical model to predict summertime N_2_O concentration and emission rates in 465,896 waterbodies in the conterminous U.S. (CONUS). We found that N_2_O undersaturation was pervasive throughout the CONUS during the summer of 2017, with an estimated 72.9% (95% credible interval: 68.9–76.6%) of lakes functioning as N_2_O sinks. The model predicts dissolved N_2_O concentrations reasonably well based partly on interactions between nitrate concentration, waterbody surface area, and water temperature. Despite working with the largest aquatic N_2_O dataset to date, our national-scale estimate of summertime N_2_O emissions from CONUS lakes is poorly constrained, with a 95% credible interval ranging from net uptake to net emission (−282 − 482 metric tons N_2_O summer^−1^). Pervasive N_2_O undersaturation in CONUS waterbodies during the summer highlights the need to revisit N_2_O models which presume surface waters are a N_2_O source.

## Abstract (web summary)

A new study finds that 73% of US lakes and reservoirs function as N_2_O sinks during the summer, but uncertainty in large lake N_2_O concentrations precludes a determination of whether US lakes and reservoirs function as a net source or sink of atmospheric N_2_O.

## Introduction

Lakes and reservoirs (lakes hereafter) are considered globally significant sources of nitrous oxide (N_2_O)^1^, a trace gas that contributes to stratospheric ozone destruction^2^ and has a radiative forcing potential 273 times that of carbon dioxide^3^. It is generally assumed that widespread nutrient enrichment has caused surface waters to produce and emit N_2_O^1,4^, but recent modeling efforts suggest that 38% of global freshwaters may function as N_2_O sinks^5^. This finding is supported by reports of N_2_O uptake in lakes across Canada^6,7^, the United States^5^, and Finland^8^. These recent studies have been conducted at relatively small spatial scales, or with a relatively small number of lakes, and the prevalence of N_2_O uptake in lakes at national to continental scales is unknown.

Nitrous oxide can be produced and consumed via microbially mediated nitrogen transformations occurring in the sediment and water column of lakes^9^. The balance between N_2_O production and consumption determines whether a lake functions as a N_2_O source or sink to the atmosphere. Nitrous oxide production and consumption rates are determined by a broad range of factors including inorganic nitrogen, dissolved organic carbon, and oxygen concentrations, among others, making it difficult to predict the balance between these processes^6,7,10^. Furthermore, N_2_O production and consumption rates can vary spatially and temporally within a lake, with some waterbodies even alternating between N_2_O sources and sinks through time^11,12^, adding additional challenges to predicting the source/sink status of lakes.

Here we present results from the first national-scale survey of N_2_O concentrations and emissions from lakes in the continental U.S. (CONUS). The survey was conducted during the warm summer months when biogeochemical processing rates are expected to be greatest. We used the data to train a Bayesian hierarchical model to predict N_2_O concentration and emission rates in 465,896 CONUS lakes. The data and model enabled us to estimate the prevalence of summertime N_2_O uptake in CONUS lakes for the first time and to identify important environmental controls on N_2_O biogeochemistry.

## Results and discussion

### N_2_O undersaturation extent

**Fig. 1:**
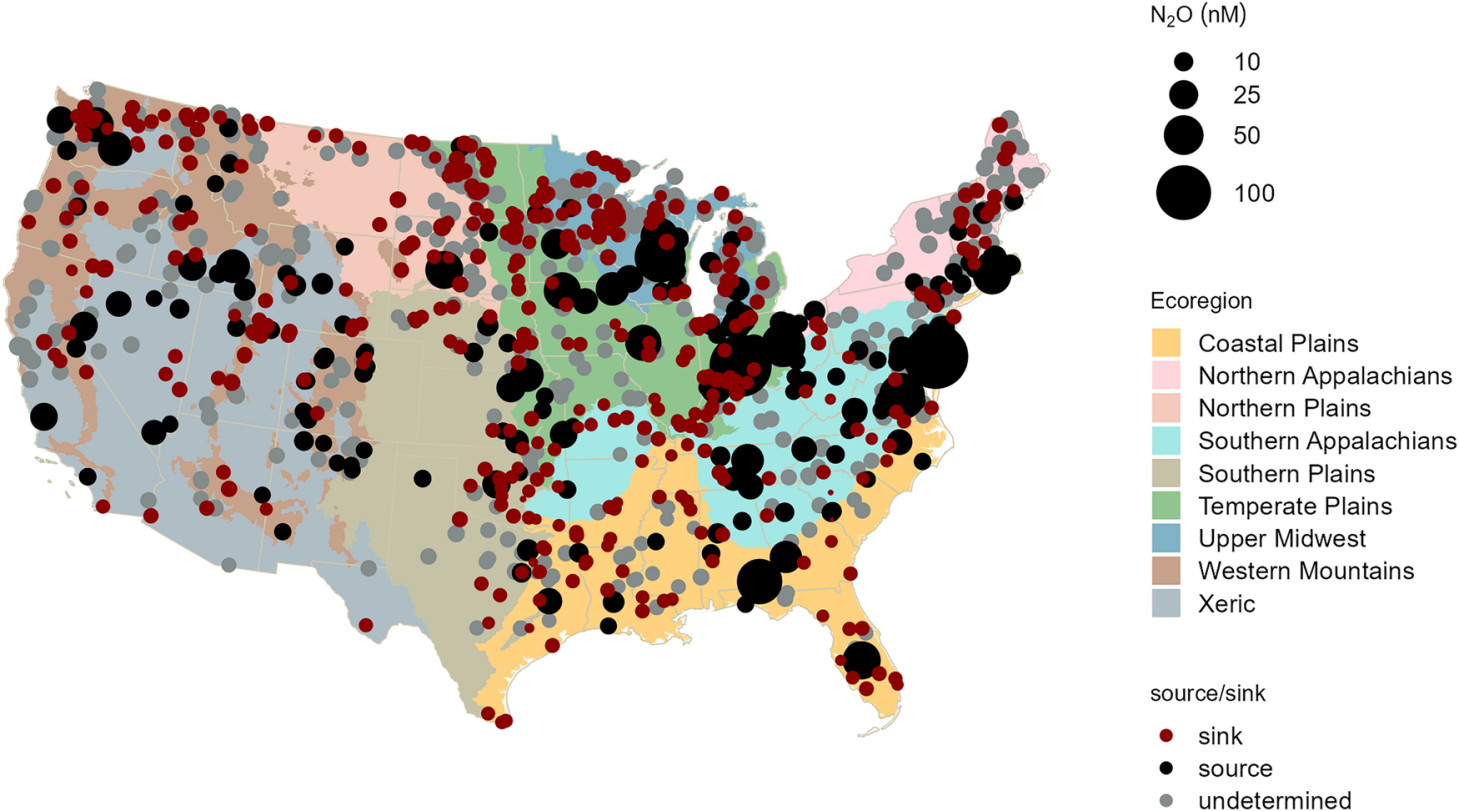
Location of sampling sites with an underlay of the nine aggregated level III US ecoregions. Color of the points indicates whether the waterbody was functioning as a source or sink of nitrous oxide (N_2_O). Point size indicates the dissolved N_2_O concentration (nmol L^-1^, abbreviated as nM in legend).

During the summer of 2017, the United States Environmental Protection Agency conducted the first national-scale survey of N_2_O concentrations and emissions in CONUS lakes as part of the National Lakes Assessment (NLA)^13^. The population of interest (POI) for the survey was 465,896 CONUS lakes that had a surface area of at least 1 ha. A representative sample (*n* = 984) was selected from the POI using a generalized random-tessellation stratified survey design^14^ (Fig. 1). The survey design was stratified by state and used unequal probability categories based on lake size (surface area) to ensure that large lakes, which are far less common than small lakes, were included in the study. Sample sites were distributed across all nine major CONUS ecoregions^15^ and varied widely in water chemistry, morphometry, watershed land-use, and climate^16^. The sample data and survey design information (e.g., stratification variables and unequal probability categories) were used to develop a Bayesian hierarchical model to predict dissolved and equilibrium N_2_O concentrations for the POI. Predictor variables for the model were restricted to those required to generate predictions that reproduced the observed N_2_O measures in an out-of-sample dataset and were available for the POI. See Methods and SI_1 for a detailed discussion of variable selection.

During at least one site visit, 69 percent of the sampled lakes had a dissolved N_2_O concentration that was lower than the concentration expected if the lake were in equilibrium with the atmosphere, suggesting those lakes were functioning as N_2_O sinks (Fig. 1). Nitrous oxide concentrations were close to equilibrium values in some of these lakes, however, and after accounting for measurement uncertainty in both dissolved and equilibrium N_2_O concentration (SI_2), we can confidently report that a minimum of 39 percent of the sampled lakes were functioning as sinks during at least one site visit, whereas only 18 percent were supersaturated (Fig. 1).

The Bayesian hierarchical model predicted that the majority lakes in the POI (posterior mean = 72.9%, 95% credible interval (CI): 68.9–76.6%) functioned as net N_2_O sinks during the summer months, a surprisingly high estimate, particularly for nitrogen rich ecoregions that are managed for agricultural production. This finding runs counter to the long-standing paradigm that surface waters are a source of N_2_O to the atmosphere^4^ and joins other recent reports that lakes, ponds, streams, and rivers can function as N_2_O sinks^5–8^. While these latter surveys were conducted at regional scales or had relatively few observation sites, the national-scale results presented here provide the strongest evidence to date that most lakes function as N_2_O sinks during the summer months.

### N_2_O saturation ratio

**Fig. 2:**
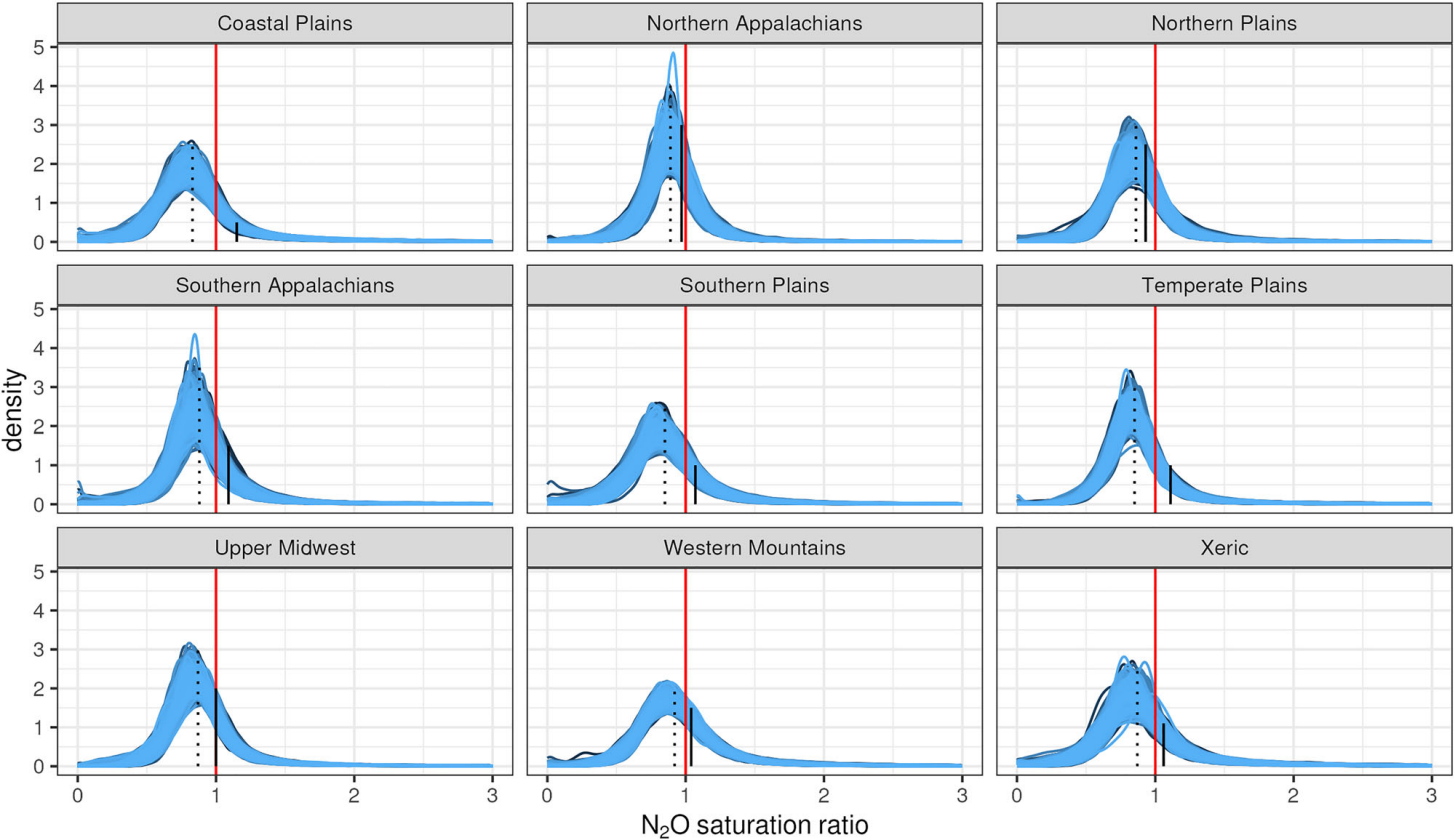
Predicted distribution of N_2_O saturation values across lakes in the nine major ecoregions. Each blue line (*n* = 2000) represents the distribution of values from a single realization of the posterior predictive distribution. Vertical black dashed and solid lines indicate point estimates of the median and mean, respectively, across all realizations (Table 1). Vertical red line indicates a saturation ratio of one. *x*-axis is truncated at a value of three to better show the area where 97% of the observations occurred.

**Fig. 3:**
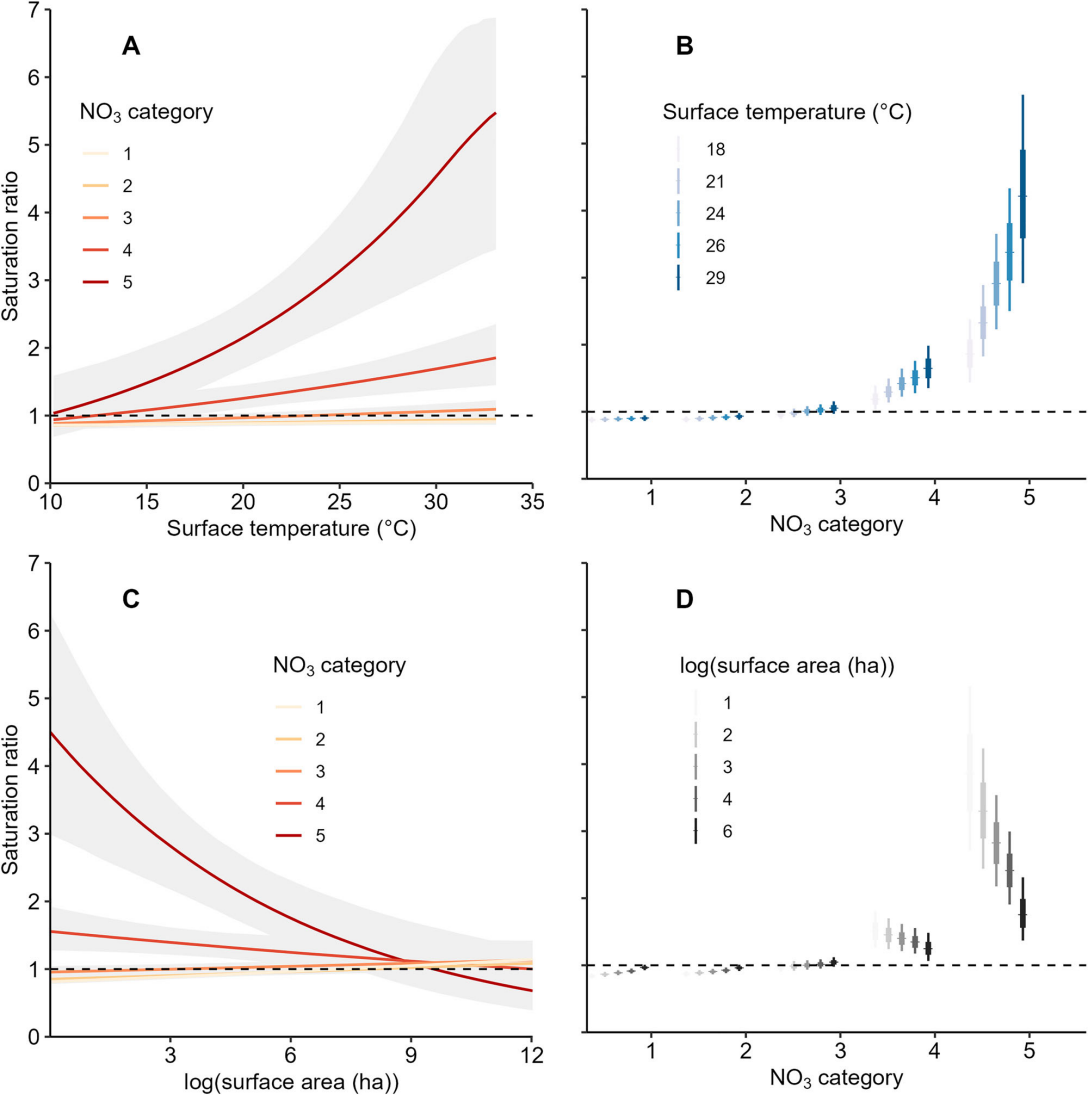
Predicted relationship between N_2_O saturation ratio and environmental drivers. Estimated interactive effects of surface water temperature (**A**, **B**) and waterbody size (**C**, **D**) with NO_3_ levels on the N_2_O saturation ratio. **A** The relationship between N_2_O saturation and surface water temperature, dependent upon nitrate (NO_3_) level. The five, ordered, categorical levels for NO_3_ correspond to concentration (*μ*g N L^−1^) ranges in the observed data of 1) below detection, 2) ≥ 0.55 and <4.09, 3) ≥ 4.09 and <30.20, 4) ≥ 30.20 and <223.13, and 5) ≥ 223.13. **B** The relationship between N_2_O saturation and NO_3_ level conditional on surface water temperature. The NO_3_ effect was estimated at five quantiles of the surface temperatures observed (10%, 25%, 50%, 75%, 90%). **C** The relationship between N_2_O saturation and waterbody surface area (log-transformed hectares) conditional on NO_3_ level. **D** The relationship between N_2_O saturation and NO_3_ level conditional on (log) waterbody surface area (five quantiles). Horizontal dashed lines in each panel reflect an N_2_O saturation value of one.

Despite pervasive summertime N_2_O undersaturation across the POI, estimated means for N_2_O saturation ratio (ratio of measured to equilibrium concentration) were indistinguishable from a value of one in all ecoregions (Table 1, Fig. 2). The apparent contradiction of pervasive N_2_O undersaturation and average dissolved N_2_O concentrations close to equilibrium can be attributed to the prominent right skew in the predicted distribution of dissolved N_2_O concentrations across the POI (Fig. 2). Across the CONUS, and within each of the nine major ecoregions, the predicted distribution of N_2_O saturation ratios had a median value less than one, indicating that most lakes in these populations were undersaturated. However, the long right tails on these distributions, comprised of relatively rare lakes with very high levels of N_2_O supersaturation, were sufficient to drive the population means to values close to, or even greater than one. Thus, most lakes in the CONUS were functioning as N_2_O sinks during the summer months, but a few functioned as strong N_2_O sources, particularly at high NO_3_ and high water temperature (Fig. 3).

**Table 1:** Mean saturation ratio, undersaturation extent, emission rate, and flux estimates for N_2_O at the national and ecoregional scales

Spatial domain	Total waterbody surface area (hectares)	Mean N_2_O saturation ratio^a^	N_2_O undersaturation extent (% lakes)^a, b^	N_2_O emission rate (mg N_2_O m^−2^ d^−1^)^a, c^	N_2_O flux (metric ton N_2_O summer^−1^)^a, c^
National	9,755,663	1.08 (0.99–1.2)	72.9 (68.9–76.6)	0.018 (−0.005–0.049)	114 (−282–482)
Coastal Plains	2,385,064	1.15 (0.98–1.42)	73.6 (66.5–79.7)	0.035 (−0.012–0.107)	−72 (−413–201)
Northern Appalachians	976,789	0.97 (0.91–1.07)	72.5 (64.1–80)	−0.008 (−0.024–0.016)	17 (−9–42)
Northern Plains	513,875	0.93 (0.86–1.01)	76.1 (68.4–83.2)	−0.022 (−0.041–0.003)	−4 (−62–31)
Southern Appalachians	1,054,281	1.09 (0.95–1.32)	71.5 (62.6–79.6)	0.019 (−0.009–0.066)	65 (24–133)
Southern Plains	584,240	1.07 (0.92–1.32)	72.4 (64.8–79.9)	0.019 (−0.019–0.088)	29 (2–77)
Temperate Plains	863,378	1.11 (0.97–1.31)	75.2 (68.3–81.4)	0.026 (−0.005–0.073)	41 (10–87)
Upper Midwest	1,718,068	1 (0.93–1.09)	73.7 (66.4–79.7)	0 (−0.017–0.021)	33 (−7–75)
Western Mountains	790,624	1.04 (0.95–1.17)	63.1 (56.6–69.7)	0.009 (−0.017–0.046)	16 (−15–50)
Xeric	869,343	1.06 (0.94–1.24)	69 (60.3–76.8)	0.014 (−0.02–0.064)	−11 (−85–57)

^a^Mean and 95% credible intervals based on 2000 draws from the posterior distribution;

^b^Percent of population of interest with N_2_O saturation ratio <1;

^c^Negative values indicate N_2_O uptake;

The model results suggest that NO_3_ may be an important predictor of summertime N_2_O, but the relationship is complicated by interactions with water temperature and lake surface area **(**Fig. 3**)**. These complex interactions may explain contrasting literature reports regarding the role of NO_3_ in regulating N_2_O. Several studies have reported positive correlations between NO_3_ and N_2_O in lakes^8,17^ whereas others have found no relationship^6,18^. It is possible that the NO_3_ ~ N_2_O relationship was not detected in the latter studies because it was confounded by interactions with temperature, lake size, thermal stratification, or other environmental variables.

The influence of NO_3_ levels on N_2_O saturation (Fig. 3) may reflect a relationship between NO_3_ concentration and denitrification, a type of anaerobic metabolism that couples the oxidation of organic matter with the sequential reduction of NO_3_ to nitrite (NO_2_), nitric oxide (NO), N_2_O, and N_2_^19,20^. The reduction of NO_3_ to N_2_ can be incomplete, however, resulting in the release of N_2_O to the water column. Incomplete denitrification often occurs when NO_3_ is relatively abundant^10,11^, likely because the final reduction of N_2_O to N_2_ is the least energetic reaction in the denitrification pathway^20,21^. When NO_3_ is limiting, however, denitrification can resort to scavenging N_2_O from the water column and reducing it to N_2_, thereby functioning as an N_2_O sink. N_2_O consumption via denitrification may explain the N_2_O undersaturation predicted at low NO_3_ levels, whereas denitrification may be a source of N_2_O at higher NO_3_ levels.

It is also possible that the correlation between N_2_O and NO_3_ reflects the simultaneous production of these solutes via nitrification. Nitrification, an aerobic process which can produce N_2_O as a byproduct, is the microbial oxidation of ammonium (NH_4_^+^) to NO_3_^19^. Nitrification may be more prevalent than denitrification in the predominantly well oxygenated surface waters where the N_2_O samples were collected because denitrification is an anaerobic process. Thus the N_2_O - NO_3_ relationship may be reflective of some combination of N_2_O production/consumption via denitrification and N_2_O production via nitrification.

The estimated pattern of increasing N_2_O saturation with water temperature (Fig. 3) is consistent with nitrogen transformations occurring through temperature sensitive biological processes, but the relationship was dependent on NO_3_ levels. At low NO_3_ levels, temperature was estimated to have a relatively minor effect on N_2_O saturation, possibly because N_2_O production/consumption was limited by substrate rather than temperature. The effect of temperature was more pronounced at greater NO_3_ levels when substrate limitation was less severe. Elevated water temperature may serve to magnify rates of N_2_O production that may be largely dictated by NO_3_ availability.

**Fig. 4:**
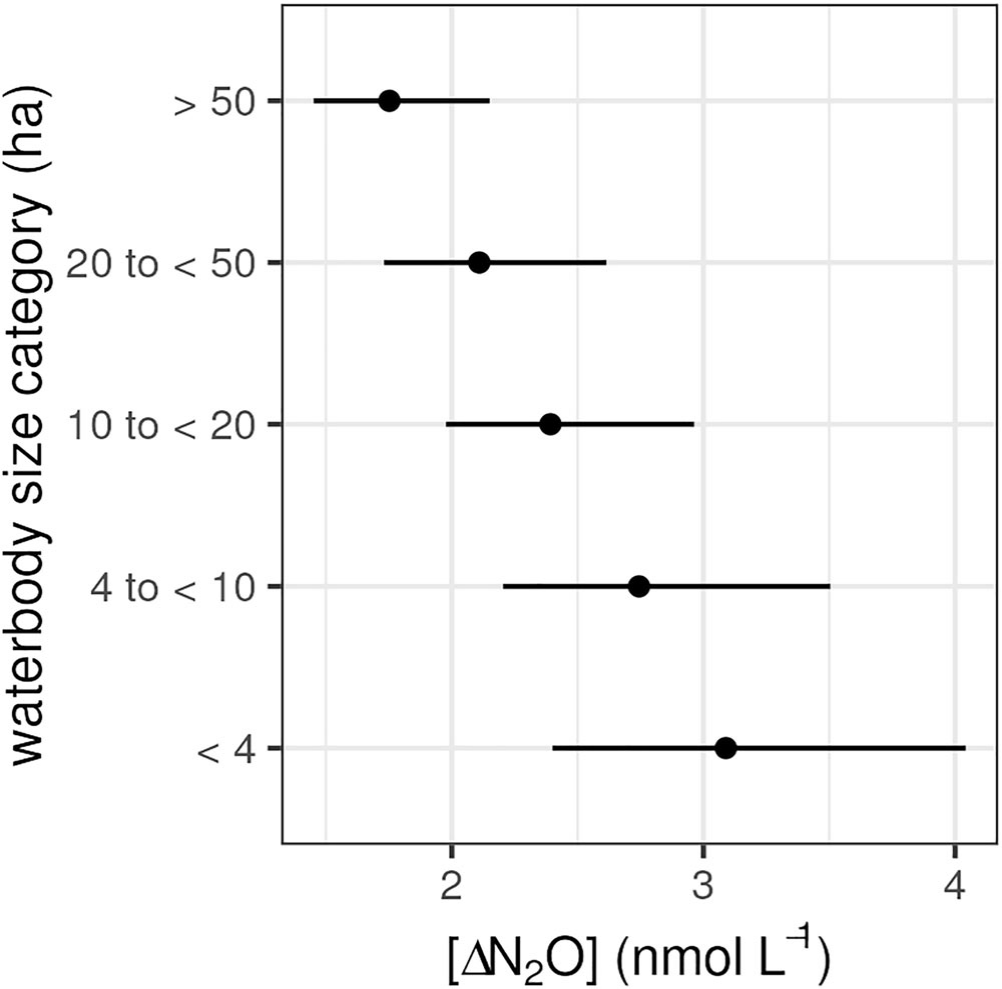
Relationship between N_2_O disequilibrium and waterbody size. Estimated mean Δ N_2_O (difference between dissolved and equilibrium N_2_O concentrations) versus waterbody surface area (ha) size categories. Posterior mean (points) and 95% credible intervals (horizontal bars) were based on 2000 draws from the posterior distribution.

Lake size (surface area) was also related to N_2_O saturation ratio, but the relationship was dependent on NO_3_ concentration (Fig. 3). Small lakes with low NO_3_ levels were predicted to be undersaturated, whereas small lakes with high NO_3_ were predicted to be highly supersaturated. Large lakes, regardless of NO_3_ status, were predicted to have N_2_O saturation ratios closer to equilibrium. Thus N_2_O saturation ratio tended to deviate from a value of one more strongly in small lakes as compared to larger lakes. This pattern can also be seen by calculating the absolute magnitude of the difference between the measured and equilibrium N_2_O concentration, known as Δ N_2_O. When pooled across all ecoregions, Δ N_2_O is predicted to decline as lake size increases (Fig. 4). This pattern holds regardless of whether N_2_O was supersaturated or undersaturated. Lake size and shape can affect dissolved gas concentration through several mechanisms. For example, N_2_O inputs from streams or groundwater^22^ may constitute a substantial N_2_O subsidy to small lakes but are more likely to be diluted in larger lakes with greater volumes. Furthermore, sediment may warm faster in small and shallow lakes, whereas thermal stratification may substantially delay sediment warming in large and deep waterbodies, resulting in greater rates of N_2_O production or consumption in small lakes. Thermal stratification may also prevent the exchange of water between the lake surface and the hypolimnion, thereby preventing N_2_O depleted or enriched water from advecting to the lake surface^10,11^.

Persistent patterns in the air-water gas exchange rate across lake size classes may also contribute to greater N_2_O disequilibrium in smaller lakes. Air-water gas exchange will vent excess N_2_O from surface waters or facilitate the invasion of air that can replenish depleted N_2_O in undersaturated lakes. Regardless of whether a lake is supersaturated or undersaturated in N_2_O, air-water gas exchange will function to move the dissolved N_2_O concentration toward equilibrium. One of the primary drivers of air-water gas exchange in lentic waters is wind speed^23^. Wind speeds tend to be greater in large lakes with long fetches and lower in small lakes, particularly if they are sheltered by shoreline vegetation. Thus, greater air-water gas exchange rates in large lakes may maintain dissolved N_2_O concentrations close to equilibrium, whereas the effect of biogeochemistry on dissolved N_2_O may be more pronounced in small lakes because low air-water gas exchange limits the rate at which excess N_2_O can be ventilated or depleted N_2_O can be replenished. While previous surveys have documented negative relationships between lake size and dissolved methane concentration^24–26^, to the best of our knowledge this is the first study to document a relationship between lake size and Δ N_2_O.

### Emission rates

**Fig. 5:**
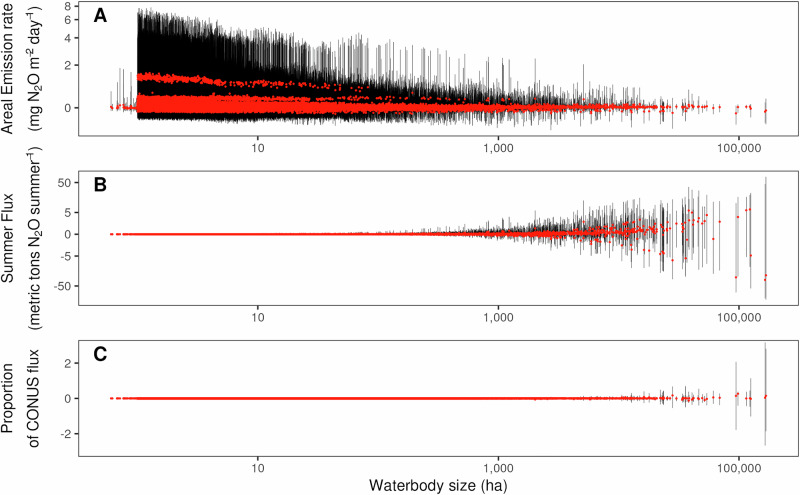
Predicted summertime N_2_O emission rate and fluxes. **A** Predicted summertime N_2_O emission rate and **B** flux plotted against waterbody size for all 465,896 lakes in the population of interest. Points (red) and bars (black) reflect the posterior means and 95% credible intervals, respectively, based on 2000 draws from the posterior predictive distribution for each waterbody. **C** Proportion of total CONUS summertime N_2_O flux attributed to each of the 465,896 lakes in the POI. Points (red) and bars (black) reflect the posterior means and 95% credible intervals, respectively, based on 2000 draws from the posterior predictive distribution for each waterbody.

The predicted summertime emission rates for the POI spanned a broad range (95% CI min: −1.184 to −0.46 mg N_2_O m^-2^ d^-1^, 95% CI max: 12.777–82.655 mg N_2_O m^-2^ d^−1^), which was expected for this large and diverse collection of lakes, but is within the range of values reported in the literature^7,8,17^, despite the literature containing values from multiple seasons, whereas this study was limited to the summer. Variability and magnitude of predicted N_2_O emission rates decreased with increasing lake size (Fig. 5A), which reflects the relationship between N_2_O saturation and lake size (Fig. 3C,D). Despite pervasive N_2_O undersaturation predicted across the POI, the estimate for the national average N_2_O emission rate was centered over positive values (posterior mean = 0.018, 95% CI: -0.005 - 0.049 mg N_2_O m^-2^ d^-1^) (Table 1) and clearly exceeded the negative estimate for the national median (posterior median = −0.032, 95% CI: −0.039 to −0.026 mg N_2_O m^-2^ d^-1^). This departure from the median was due to relatively rare lakes with very high emission rates pulling the mean towards the direction of positive skew (Fig. S1).

### Fluxes

Total N_2_O flux from the surface of a lake is calculated as the product of the N_2_O emission rate and lake surface area. The four smaller size classes ( < 50 Ha) in the survey design represented 97% of all lakes in the POI but were estimated to have contributed less, on average, to the total N_2_O flux from CONUS lakes than the largest lake size class (Fig. 5C). The reason for this pattern is two-fold. First, lakes in the smaller size classes constituted only 20% of total waterbody surface area, thereby limiting their N_2_O flux. Second, pervasive N_2_O uptake in these size classes largely offset the very high emission rates predicted to occur in a minority of the POI. Lakes in the largest size class ( > 50 Ha) represented only 3% of the total number of lakes in the population but had 80% of total lake surface area. This lake size class was estimated to be, on average, the largest contributor to the total N_2_O flux across the POI (Fig. 5C), though the flux estimate is poorly constrained (posterior mean = 77.1, 95% CI: −306.4 to 420.9 metric tons N_2_O summer^-1^). While uncertainty in N_2_O emission rates was smallest for large lakes (Fig. 5A), these small uncertainties propagated to large uncertainties in flux when expressed across the surface area of large lakes (Fig. 5B).

The estimated summertime N_2_O flux across the POI was centered over positive values (posterior mean = 114 metric tons N_2_O summer^-1^) but the credible interval included zero (−282 to 482 metric tons N_2_O summer^−1^), thus we are unable to infer the extent to which CONUS lakes, overall, were a net source or sink of N_2_O during the summer. We do not scale our flux estimates to the year due to the potential for seasonal variability that was not captured during our summer sampling. Seasonal measurements of N_2_O emission rates are rare and the few published studies are inconsistent. Some systems exhibit maximum N_2_O emission rates during the winter^8,12^, others in the fall^11^, and yet others during the summer^27^. A better understanding of N_2_O seasonality in CONUS lakes is required to produce a robust annual emission estimate.

While other published estimates of N_2_O flux from CONUS lakes are reported at the annual-scale, a comparison with our summer flux estimates can still inform our understanding of N_2_O dynamics and modeling approaches. Lauerwald et al.^28^ used a mechanistic model to estimate that CONUS lakes emit 8425 metric tons N_2_O year^-1^. That estimate is nearly 75-fold greater than the posterior mean of our estimate (114 metric tons N_2_O summer^-1^), despite our estimate including an order of magnitude more lakes than Lauerwald et al.^28^. A possible explanation for the large difference between these estimates, aside from the role of seasonality, is that the Lauerwald model does not allow for lakes to behave as N_2_O sinks, a limitation that extends to many mechanistic models used to estimate N_2_O fluxes from inland waters^4,29,30^. However, even models that allow for net N_2_O uptake predict much greater N_2_O fluxes than reported here. For example, Wang et al.^22^. used a model that allows for N_2_O consumption via denitrification to predict that CONUS lakes emitted an average of 47,614 metric tons N_2_O year^-1^ from 2011−2015. This estimate is two orders of magnitude greater than the posterior mean of our estimate and suggests the mechanistic model does not properly describe N_2_O consumption. Given our estimate of pervasive N_2_O consumption in CONUS lakes, failure to properly describe this dynamic in aquatic N_2_O models may yield high-biased emission estimates.

Not only is our estimate of N_2_O emissions from CONUS lakes small compared to other published estimates^22,28^, it is also small relative to other N_2_O sources including CONUS streams and rivers^31^ (55,490 metric tons N_2_O year^−1^), wastewater treatment (88,00 metric tons N_2_O year^-1^), stationary combustion (84,000 metric tons N_2_O year^-1^), and manure management (66,000 metric tons N_2_O year^-1^)^32^. The contribution of CONUS lakes to the US N_2_O budget is likely small by comparison, regardless of the strength of seasonality.

Our observations of dissolved N_2_O were well predicted using a regression model with interactions between observed water temperature, NO_3_ concentration, and waterbody size (SI_1). These modeling results suggested that NO_3_ levels may play a role in determining whether a waterbody functions as a sink or source of N_2_O, and the magnitude of the source or sink may be exaggerated with increasing water temperature. These results suggest that the convergence of the ongoing warming of CONUS lakes^33–35^ and projected increases in freshwater nitrogen loading^36^ may increase lentic N_2_O emissions over the next century^22^.

Although lakes in the largest lake size class tend to have N_2_O levels close to equilibrium, they make a large contribution to CONUS N_2_O flux due to their expansive surface area. The N_2_O flux from large lakes is poorly constrained, however, because relatively small uncertainties in dissolved and equilibrium N_2_O concentrations translate to large uncertainties in flux when propagated across the surface of large lakes. Efforts to improve the accuracy of the lentic N_2_O flux estimate should include an assessment of intra-waterbody spatial variation in N_2_O^37,38^, as well as careful accounting of measurement uncertainty. The measurement methods employed in this study have a 95% credible interval of 0.11 (unitless) on the N_2_O saturation ratio. The 95% CI can be halved by employing gas chromatography optimized for quantifying N_2_O in the range of values observed in this study^39^ and decreased by an order of magnitude using membrane inlet mass spectrometry^40,41^ (SI_2).

The smallest lake size classes were estimated to have both the highest and lowest levels of N_2_O saturation, depending largely on NO_3_ availability. It is unclear whether this variability is related to differences in NO_3_ loading or is an artifact of temporal variation in NO_3_ processing. For example, reservoirs have been shown to function as N_2_O sources in early summer when NO_3_ is replete, but switch to N_2_O sinks in later summer when the NO_3_ supply has been exhausted^11^. A better understanding of the temporal evolution of N_2_O production and consumption is an important research need to determine the extent to which small lakes can remove excess nitrogen from surface waters and simultaneously mitigate N_2_O emissions.

## Methods

### Survey design

The population of interest (POI) for the 2017 NLA survey^13^ was CONUS lakes of at least 1 hectare in area, at least 1 meter deep, with at least 0.1 hectare of open water, and a minimum residence time of one week (Fig. 1). Inferences from the survey samples were assumed relevant to average conditions during the summer index period between June and end of September 2017. The POI was sampled according to a generalized random-tessellation design^14^ with stratification along three factors: surface area (hectares; 5 ordered levels), ecoregion^15,42,43^ (9 levels), and US state (excluding AK and HI; 48 levels). The size categories were sampled with unequal probability, such that larger lakes were over-sampled relative to smaller lakes. In total, 984 lakes were sampled on a single date and 95 were re-visited for a second sample. The revisit samples were used to assess out-of-sample model performance.

### Sampling and analysis

Samples were collected from approximately the middle of each lake for analysis of NO_3_ and dissolved N_2_O, among other analytes^13^. Duplicate dissolved N_2_O samples were collected approximately 0.05 m below the water surface by drawing 105 mL of water into a 140 mL syringe equipped with a stopcock and containing 35 mL of air. The syringes were gently shaken for five minutes and the headspace gas transferred to a 30 mL syringe. Finally, the gas sample was transferred into a 12 mL pre-evacuated ( < 50 mTorr) glass vial (Labco, Wales, UK) sealed with a PTFE/Silicone septa stacked on top of a gray chlorobutyl septa. The temperature of the water in the syringe was recorded immediately after the gas was transferred into the vial. Duplicate 20 mL air samples were collected from approximately 2 m above the water surface and transferred to 12 mL glass vials. A vertical profile of water temperature was measured at the same site.

Nitrous oxide partial pressure in the gas samples was measured via gas chromatography (Bruker 450, Billerica, MA, USA), an electron capture detector, and autosampler (CTC Analytics, PAL-xt, Zwingen, Switzerland). A 90:10 mixture of argon and methane was used as the carrier gas. The original dissolved N_2_O concentration was calculated from the measured headspace partial pressure, the temperature specific Bunsen coefficient, and a mass balance for the headspace equilibration system as described in Beaulieu et al.^44^. The equilibrium dissolved gas concentration at the time of sampling was calculated from the measured N_2_O partial pressure in the air samples, the measured barometric pressure, and the water temperature. Dissolved N_2_O concentration is presented as a saturation ratio, defined as the ratio of measured to equilibrium concentration.

Analytical, quality assurance, and field sampling details for all measurements can be found at the 2017 National Lakes Assessment Laboratory Operations Manual^45^, 2017 National Lakes Assessment Quality Assurance Project Plan^46^, and 2017 National Lakes Assessment Field Operations Manual^47^, respectively. All data can be accessed online^16^.

### Statistical inference

The following is an abbreviated description of the statistical analysis; see SI_1 for more details. The survey samples were treated as a randomly selected subset of the POI, but with a pattern of “missingess” prescribed by the selection algorithm^48,49^. For inferences, estimates were statistically adjusted to account for the selection mechanism using a multilevel regression with poststratification (MRP) approach^50–52^. MRP is a model-based approach carried out in two steps. In the first step, the sampled response (e.g., N_2_O) is conditioned on the survey design variables (e.g., ecoregion, state, size) in a multilevel regression model. Multilevel models are often recommended for this adjustment because they naturally provide (1) regularized estimates along the design groupings; and (2) estimates for design levels that may be missing from the sample, but are part of the POI^50,52,53^. In the second step of MRP, the fitted regression parameters are used to “poststratify” or re-weight the response based on a known or assumed distribution of units (e.g., CONUS lakes) in the POI^49–53^. Although it is typical to poststratify estimates at a grouping level above the observational unit (e.g., ecoregion), in this study, N_2_O flux needed to be calculated as a function of lake-level properties (i.e., ${N}_{2}{O}_{{flux}}\propto {{\rm{surface}}} \; {{\rm{area}}}$). Therefore, predictions were made to each of the 465,896 lakes in the POI. For group level inferences, the posterior predictive distribution of the response was summarized across all lakes for the groupings of interest. The fully Bayesian implementation made propagation of uncertainty from the waterbody-level predictions to higher level summaries straightforward. As a check on our model-based approach to population-level inferences, we also constructed several design-based estimates, conditioned on the survey weights, as a basis for comparison. In all cases, the MRP results were similar to the design-based results, but the MRP estimates were consistently more precise and “shrunk” towards the overall means, as is expected for this method^50,52^; see SI_1 for details.

The multilevel regression model was used to make probabilistic predictions for (1) dissolved and equilibrium N_2_O concentrations; (2) the N_2_O saturation ratio; and (3) the proportion of under-saturated water bodies (i.e., saturation ratio <1). Saturation ratio was calculated as the ratio of dissolved to equilibrium N_2_O. Because dissolved and equilibrium N_2_O were each measured on the same sample units (i.e., individual lakes), their joint distribution was modeled in order to account for potential statistical dependencies due to, for example, conditional dependencies on missing covariates (e.g., geography). The modeled response variable was, therefore, multivariate. Although the mean marginal probabilities estimated from separate models may have provided comparable estimates, the joint model allowing correlated responses was expected to better capture uncertainty and potentially improve out-of-sample predictions, should the variables be conditionally correlated^54,55^.

The saturation ratio was not modeled or predicted directly, but was estimated *post hoc* as a derived quantity^48,56^ by dividing the posterior predictive distribution (PPD) for dissolved N_2_O by the PPD for equilibrium N_2_O. Similarly, the proportion of under-saturated lakes was calculated from this PPD as the proportion of lakes with a saturation ratio less than 1. All models were fit using the brms^57^ package in R^58^ as an interface to Stan^59^, a software for fitting fully Bayesian models using Hamiltonian Monte Carlo (HMC). Fully reproducible code, results, and model workflow details are provided in the Modeling Supplement Document.

Our initial models conditioned the response on just the survey design variables, however, those models consistently resulted in poor fits to some aspects of the observed N_2_O data. These models worked reasonably well at replicating the central tendency of the observed N_2_O data, but other properties of the observed distributions, such as the variance, skewness, and kurtosis, proved difficult to replicate. A consequence of the relatively poor fit to the tail properties of the data was a downward bias in estimates of the saturation ratio. In subsequent models, a measure of NO_3_ levels was included as a covariate on the dissolved N_2_O response, which substantially improved the fit according to the posterior predictive model checks. In addition, including surface temperature and elevation measures as covariates on equilibrium N_2_O resulted in improvements, as did allowing for heterogeneous errors for both dissolved and equilibrium N_2_O. Both dissolved and equilibrium N_2_O were allowed to vary according to the survey design variables and environmental covariates.

Including covariates, however, necessitated additional steps in the MRP process. To make inferences to the POI, the covariates needed to be (1) fully observed across the POI or (2) their missingness needed to be modeled. For the lake area and elevation covariates, data were available for all lakes in the POI from previously compiled geospatial databases. However, surface temperature and NO_3_ were not observed for lakes outside of the sample. Therefore, a more complex model was developed to include surface temperature and NO_3_ as additional responses in the multivariate model conditioned on the survey design variables and the fully observed covariates. This approach is similar to MRP approaches with “non-census” variables^60^. It could also be described as akin to a Bayesian structural equation model^61,62^.The dependence structure of this model can be characterized where:

$$p({{{{\bf{N}}}}}_{{{{\bf{2}}}}}{{{{\bf{O}}}}}_{{{{\bf{diss}}}}},{{{{\bf{N}}}}}_{{{{\bf{2}}}}}{{{{\bf{O}}}}}_{{{{\bf{eq}}}}},{{{\bf{N}}}}{{{{\bf{O}}}}}_{{{{\bf{3}}}}},	 {{{\bf{Temp}}}} |{Survey},{Area},{Elev},{Lat},{Day})=\\ 	 p({{{{\bf{N}}}}}_{{{{\bf{2}}}}}{{{{\bf{O}}}}}_{{{{\bf{diss}}}}}\; |\;{Survey},{Area},{{{\bf{N}}}}{{{{\bf{O}}}}}_{{{{\bf{3}}}}},\,{{{\bf{Temp}}}})\times \\ 	 p({{{{\bf{N}}}}}_{{{{\bf{2}}}}}{{{{\bf{O}}}}}_{{{{\bf{eq}}}}}\; |\;{Survey},{Elev},{{{\bf{Temp}}}})\times \\ 	 p({{{\bf{N}}}}{{{{\bf{O}}}}}_{{{{\bf{3}}}}}\; |\;{Survey},{Area},{{{\bf{Temp}}}})\times \\ 	 p({{{\bf{Temp}}}}\; |\;{Survey},{Lat},{Elev},{Day})$$

Variables in bold text above were treated as partially observed in the POI (i.e., observed only in the sample), whereas variables not in bold were fully observed. The partially observed variables, including dissolved and equilibrium N_2_O, NO_3_, and surface temperature, were all modeled conditional on the survey design variables and other partially and/or fully observed covariates. In order to make predictions and propagate uncertainty through the dependency structure, the fitted multivariate model was used to first predict surface temperature in the POI, since it depended only on the fully observed covariates. That predictive distribution was then used alongside the relevant fully observed covariates to predict NO_3_ in the POI. Finally, the predictive distributions for temperature and NO_3_ were used in predicting both N_2_O responses in the POI.

### Emission estimates and upscaling

Areal emission rates were calculated for each waterbody and draw from the posterior predictive distribution as:

1 $${{{\rm{Emission}}}}\; {{{\rm{rate}}}}={{{\rm{k}}}}\left({{{{\rm{N}}}}}_{2}{{{{\rm{O}}}}}_{{{{\rm{diss}}}}}{{{-}}}{{{{\rm{N}}}}}_{2}{{{{\rm{O}}}}}_{{{{\rm{eq}}}}}\right)$$

where N_2_O_diss_ is the predicted N_2_O concentration in the waterbody, N_2_O_eq_ is the predicted concentration if the waterbody were in equilibrium with the atmosphere, and k is the air-water gas exchange rate. The air-water gas exchange was calculated as:

2 $${{{\rm{k}}}}={{{\rm{k}}}}_{600} * {({{{\rm{Sc}}}}_{{{\rm{T}}}}/600)}^{-2/3}$$

where k_600_ is k normalized to a Schmidt (Sc) number of 600 and Sc_T_ is the Sc of N_2_O at the predicted surface water temperature for each waterbody (Wanninkof et al.^63^). K_600_ was estimated from wind speed and lake area^23^:

3 $${{{\rm{k}}}}_{600}=2.51+1.{48} * {{{\rm{U}}}}_{10}+0.3{9} * {{{\rm{U}}}}_{10} * {\log }_{10}({{{\rm{Lake}}}}\; {{{\rm{area}}}})$$

where U_10_ is wind speed 10 m above the water surface. Average U_10_ values for the sampling period (sunrise to sunset; June 1, 2017–September 30, 2017) were extracted from the ERA-5 Land database^64^ (7.5 km grid) for each lake in the POI.

### Reporting summary

Further information on research design is available in the Nature Portfolio Reporting Summary linked to this article.

## Supplementary information

Supplementary Information

Reporting Summary

Transparent Peer Review file

## Data Availability

All data presented in this manuscript are available at https://github.com/USEPA/DissolvedGasNla and have been archived at 10.5281/zenodo.19886361^65^.

## Code availability

The code to reproduce the entire analysis is available at https://github.com/USEPA/DissolvedGasNla. Supplementary information files 1 and 2 can be found at https://github.com/USEPA/DissolvedGasNla/blob/master/scripts/NLA_N2O_models.md and https://github.com/USEPA/DissolvedGasNla/blob/master/scripts/DG_sensitivity_to_measurement_error.md, respectively. A stable version of the repository is archived at 10.5281/zenodo.19886361^65^.
