# Supplementary material for: Pervasive summertime nitrous oxide undersaturation in U.S. lakes and reservoirs
Source: Nat Commun. 2026 Jul 11;17:8551. doi: 10.1038/s41467-026-74705-6 (PMC13482806; doi:10.1038/s41467-026-74705-6)
Supplement: Supplementary file 1 [file 41467_2026_74705_MOESM1_ESM.pdf]

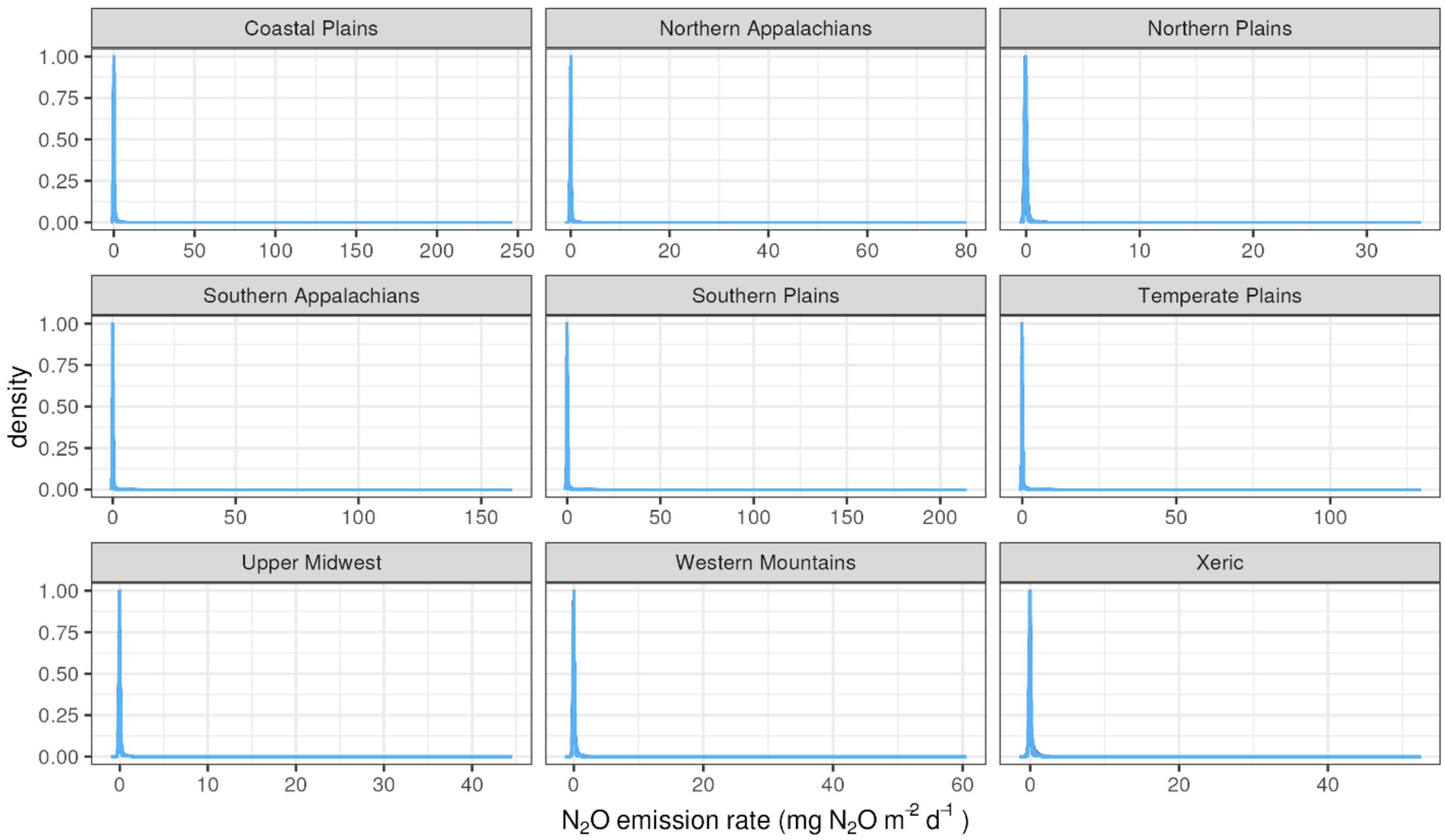

Figure S1. Distribution of N~2~O emission rates by ecoregion. Each blue line (n=500) represents the distribution of values from a single realization of the posterior prediction.

Supplemental Information 1: Modeling workflow: NLA N2O survey data

Roy Martin, Jake Beaulieu, Michael McManus 2025-03-17

1 Introduction

This supplement documents the modeling workflow used for estimating dissolved and equilibrium N2O gas concentrations and saturation ratios for freshwater lakes (natural and man-made) across the continental United States (CONUS). Data for the estimates came from the 2017 Nation Lakes Assessment (NLA) survey, which aimed to provide representative samples for describing environmental conditions across CONUS lakes during the summer index period of 2017 (U.S. EPA 2015). The population of interest (POI) for this study was defined as all lakes and reservoirs in the CONUS that were (1) larger than 1 hectare, (2) with at least 0.1 hectares of open water, (3) at least 1 meter deep, and (4) with a residence time of less than 1 week. The 2017 NLA sampling frame consisted of 465,897 lakes delineated from georeferenced data documented in the National Hydrography Dataset (NHD). The samples were drawn according to a spatially balanced, stratified, and unequal probability design. Sampling was stratified among categories of lake size (surface area in hectares), WSA9 ecoregion, and US state (excluding AK and HI). The probability of selection was greater for larger lakes relative to smaller ones in order to sufficiently represent larger lakes in a sample of practical size. Small lakes vastly outnumber large ones in the POI.

The POI was defined in this study explicitly by the NLA 2017 sampling frame ((U.S. EPA 2015)). Therefore, the effective and stated POI may depart in important ways that would be difficult to confirm in practice. For example, it would be impractical to visit all lakes in the sampling frame to determine which contained less than 0.1 ha of open water or were less than 1 meter deep. These conditions were evaluated in the field for sites that were sampled. If a field crew arrived at a site that did not meet the conditions for inclusion, another site could be chosen from an oversample frame. In effect, the size of the stated POI is likely smaller than the 465,897 lakes described in the sampling frame and, therefore, the possibility of some remaining biases in these estimates should be considered, if only to emphasize that there remains uncertainty in the population-level estimates beyond what

was estimated from modeling. Though it may be practically impossible to determine the precise magnitude or direction in which any particular estimate could be biased, it is clear that estimates could vary systematically given any substantial mismatches between the POI and the sampling frame.

When making inferences to a POI from a sample, estimates should be adjusted to address potential biases resulting from known differences between the POI and sample. For this study, lakes in the POI were missing from the sample not completely at random, but systematically and according to the probability assigned by the survey design. This type of "missingness" is not ignorable for making inferences from a sample, but may be considered ignorable if the inferences are adjusted such that they are conditional on the survey design (Gelman et al. 2014, Ch. 8; Gelman, Hill, and Vehtari 2020 Ch. 17). A straightforward approach to adjustment is to simply include the design variables as predictors in a regression model for the surveyed responses of interest (Gelman, Hill, and Vehtari 2020, Ch. 17). Then, in a second step referred to as "poststratification" (Gelman, Hill, and Vehtari 2020, Ch. 17), the model parameter estimates can be used to make population-level estimates by summarizing over the known distribution of the design variables in the POI or sub-populations therein. Multilevel regression models are often preferred in this approach, which is why it is often referred to as multilevel regression with poststratification, or MRP (Park, Gelman, and Bafumi 2004; Gelman and Little 1997; Gelman, Hill, and Vehtari 2020, Ch. 17; Kennedy and Gelman 2021). Multilevel regression models are recommended because they naturally provide regularized estimates along the design groupings, which can improve out-of-sample inferences (Gelman and Little 1997). Likewise, estimates for group levels that may be missing from the sample (e.g., an unsampled U.S. state), but are part of the POI, are straightforward using the multilevel approach (Gelman, Hill, and Vehtari 2020 Ch. 17; McElreath 2020). For a worked and narrated example using this approach, see a recent paper by Kennedy and Gelman (Kennedy and Gelman 2021). For an example in the context of national surveys of environmental resources, see a recent paper from Zachmann and others (Zachmann et al. 2022).

In this study, we used the 2017 NLA survey data and an adjustment approach similar to MRP to provide population-level estimates for (1) dissolved and equilibrium N2O concentrations; (2) the N2O saturation ratio (i.e., dissolved N2O/equilibrium N2O); and (3) the proportion of under-saturated water bodies (i.e., saturation ratio < 1). The resulting estimates were later combined with lake-level attributes (e.g., surface area) to provide estimates of N2O emission rates and flux. A number of Bayesian multilevel regression models were fit to the survey data and predictions from those models were checked against both the data used in their fitting as well as a subset of held-out data from re-visited sites. Because dissolved and equilibrium N2O were each observed on the same sample units (a

single site within each waterbody), our regression models were constructed to estimate their joint probability distribution. The multivariate response allowed for conditional dependencies between dissolved and equilibrium N2O due to, for example, physiography. Although predictions from separate models for each N2O response may have provided comparable estimates, a joint model was expected to better capture uncertainty and potentially improve out-of-sample predictions, should the data be conditionally correlated (Warton et al. 2015; Poggiato et al. 2021). The saturation ratio (saturation = dissolved N2O/equilibrium N2O) was estimated as a derived quantity calculated from the joint posterior predictive distribution of the two N2O variables.

Our initial regression models were focused on conditioning the N2O data on the survey design factors only, which included multilevel groupings of the sampled lakes according to WSA9 ecoregion, states within ecoregions, and size classes within states. Although these initial models performed well at replicating the observed group-level means, further model checking indicated that they did a relatively poor job of replicating some tail properties, such as the long right tail of the observed dissolved N2O data. As a result of this misfit, the predictive distribution for the saturation ratio was also consistently biased low relative to the observed data. In subsequent models, we found that including an NO3 covariate was particularly helpful for better replicating these extreme values. The addition of predictors for surface temperature and lake surface area, along with interaction terms for these predictors with NO3, also lead to apparent improvements. Likewise, the inclusion of surface temperature and elevation covariates in the equilibrium N2O response resulted in improvements over the initial models, as did added flexibility in the distributional terms (heterogeneous errors) for both dissolved and equilibrium N2O.

A number of additional, potentially relevant covariates were evaluated in exploratory analyses and models, but are not covered in this document. These variables are, however, available to the public should the reader wish to further explore their utility. These measures, which were taken at the time of N2O sampling or constructed from those measures, included chlorophyll a concentration, dissolved oxygen (DO) content at the surface, and a buoyancy metric. Some of these variables are also covered in the data munging document at:
https://github.com/USEPA/DissolvedGasNla/blob/master/scripts/dgIndicatorAnalysis.html.
An exploratory analysis using classification trees in that document suggested that NO3 as well as both mean depth and chlorophyll a concentration were associated with dissolved N2O concentration. The decision to not include these variables in the final model was based on several issues. First, our primary goal was to make population-level estimates for N2O while considering the survey design structure. As a result, we were less concerned with exploring the relative contributions of environmental correlates to N2O variation. However,

our initial models excluding all covariates proved to result in relatively poor fits to important aspects of the observed data. In an attempt to improving the fit, the NO3 covariate was then included due to the relatively large effects implied in exploratory analyses (see “Sample Data Exploration” to follow). Conversely, our evaluations of exploratory plots and models with chlorophyll a, surface DO, and the BF covariates did not result in similarly substantial improvements. Second, these other covariates were only available in the sample and, therefore, were not fully observed in the POI. As such, using them to make population-level predictions would have come with additional complexity (discussed below) that didn’t appear to be reciprocated by critical improvements in model fit. Finally, several observations were missing from the sample for these covariates. To include them in our model would have required sub-models for the missing data, resulting in added complexity and computational burden that, again, did not appear to be worth the added complexity and computational burden. Hence, the progression of models presented in this supplement was intended to efficiently convey the improvements in fit following the addition of key covariates and parameterizations that ultimately comprised the final model.

The first regression model we fit was constructed to estimate the joint distribution of log-transformed dissolved and equilibrium N2O conditional only on the the survey design factors, such that:

$$Y \sim MVN(\nu, \Sigma)$$

Indexing to each response, p:

$$Y = [y_{p=1}, y_{p=2}] \sim MVN(\nu = [\mu_{p=1}, \mu_{p=2}], \Sigma)$$

Indexing to the observation level, i, within each response:

$$\mu_{pijkl} = \alpha_{0(pi)} + \alpha_{1(pij)} + \alpha_{2(pijk)} + \alpha_{3(pijkl)}$$

$$\alpha_1 \sim MVN(0, \Lambda_1)$$

$$\alpha_2 \sim MVN(0, \Lambda_2)$$

$$\alpha_3 \sim MVN(0, \Lambda_3)$$

$$\Lambda_{\alpha=1,..,3} = \begin{pmatrix} 1 & \tau^2_{p=1} \\ \tau^2_{p=2} & 1 \end{pmatrix} \chi_{\alpha=1,..,3} \begin{pmatrix} 1 & \tau^2_{p=1} \\ \tau^2_{p=2} & 1 \end{pmatrix}$$

$$\chi_{\alpha=1,..,3} = \begin{pmatrix} 1 & \varrho_{p1,p2} \\ \varrho_{p1,p2} & 1 \end{pmatrix}$$

$$\Sigma = \begin{pmatrix} 1 & \sigma^2_{p=1} \\ \sigma^2_{p=2} & 1 \end{pmatrix} \Omega \begin{pmatrix} 1 & \sigma^2_{p=1} \\ \sigma^2_{p=2} & 1 \end{pmatrix}$$

$$\Omega = \begin{pmatrix} 1 & \rho_{p1,p2} \\ \rho_{p1,p2} & 1 \end{pmatrix}$$

Each log-transformed observation, $i \in 1, .., N = 984$, for each N2O response variable, $p \in 1 : P = 2$, was assumed to be drawn from a multivariate normal (MVN) distribution with the parameters ν and Σ, where ν is the multivariate mean and Σ is a covariance matrix containing the observation-level variances and (residual) correlations.

The multivariate mean was defined by a vector of location parameters, $\nu = [\mu_{p=1}, \mu_{p=2}]$, for each response, p. As noted above, each location parameter was also defined according to a linear combination of parameters indexed to each response p and observation i. This linear combination included a fixed global intercept, α_0, conditioned on the data, and three latent group-level effects matrices, $\alpha_1, \alpha_2, \alpha_3$. The effects in each of these matrices were also drawn from MVN probability distributions centered on zero in p-dimensional space. The spread of the effects around zero were determined by a corresponding variance-covariance matrix, $\Lambda_1, \Lambda_2, \text{or } \Lambda_3$. The standard deviation parameters, τ , of those matrices were conditioned on the data and captured group-level standard deviations that controlled the spread of the group-level effects for each response. The X parameter contained the group-level residual correlation matrix, wherein ϱ denotes the group-level residual correlations (i.e., between responses).

The explicit indexing in the notation above conveys the relationship between the parameters and each observation, i, and emphasizes the nested structure of the observations within the group effects. Specifically, each observation, i, was nested in a lake size category, l, which was nested in a state, k, and ecoregion, j. The group-level effects in α_1, therefore, accounted for ecoregion-level deviations from the global mean; α_2 accounted for state-level group effects nested in ecoregions; and α_3 accounted for lake size category effects within states and ecoregions.

Finally, the observation-level covariance term, Σ, comprised of the observation-level standard deviations, σ, for each response, and Ω or the observation-level residual correlation matrix, wherein ρ captured residual correlation between responses.

For model fitting, priors were needed for all parameters conditioned directly on the data, which included the global intercepts, α_0; the scale parameters of the latent group effects, τ and σ; and the correlation parameters, ϱ and ρ. A normal or Gaussian prior, $N(\mu = 2, \sigma = 1)$ centered near the (log-scale) data means, was used as the prior for the

global mean parameter for each response. This prior was considered minimally informative as it placed most (~80%) of the prior mass over values between about 2 and 27 ng/L for median N2O and median N2O equilibrium concentration; and included support in the tails for values approaching 0 ng/L on the lower end and 80 ng/L on the high end. We placed $Exp(2)$ priors over all scale parameters, which placed most of the support between values very close to 0 and values near 1 (central 80% density interval from approximately 0.005 to 1.15). Finally, for the correlation matrices, an $LKJ(\eta = 2)$ prior was used, which, for a 2-dimensional response, placed most support for correlations between approximately -0.9 and 0.9. This prior seemed reasonable as there is no direct causal link between these responses that would warrant a very strong correlation . Any residual dependence was expected to be conditional or indirect due to, for example, a common correlate (e.g., elevation, temperature). For more information on prior choice recommendations in Stan, see: https://github.com/stan-dev/stan/wiki/Prior-Choice-Recommendations

The $\mathbf{brms}$ package (Bürkner 2017) for $\mathbf{R}$ (R Core Team 2021) was used to fit all of the models in a fully Bayesian setting. The formula syntax of the $\mathbf{brms}$ package is similar to the syntax used in the $\mathbf{lme4}$ package that is widely used to fit mixed effects models in frequentist settings. In either package, the linear predictor for μ described above, but ignoring multivariate dependencies, would be expressed as:

$$\sim 1 + (1|WSA9) + (1|WSA9 : state) + (1|WSA9 : state : size)$$

In the $\mathbf{brms}$ package, there is additional functionality and formula syntax for multivariate responses, which allows for the varying intercepts of the separate linear predictors in a multivariate model to be correlated across responses, e.g.,:

$$N_2O_{diss} \sim 1 + (1|a|WSA9) + (1|b|WSA9 : state) + (1|c|WSA9 : state : size)$$
$$N_2O_{equi} \sim 1 + (1|a|WSA9) + (1|b|WSA9 : state) + (1|c|WSA9 : state : size)$$

The above formula syntax indicates that the linear predictor for both N2O responses have the same group-level varying effects terms, and that the effects associated with each of those terms can be correlated across responses. The relevant sections of $\mathbf{R}$ code that are helpful for describing the first model above using $\mathbf{brms}$ syntax are below:

```
# Assign separate linear predictors for the dissolved and equilibrium N2O responses
bf_n2o <- bf(log(n2o) ~ 1 +
               (1 | a | WSA9) +
               (1 | b | WSA9:state) +
               (1 | c | WSA9:state:size_cat),
             family = gaussian())
```

```
bf_n2oeq <- bf(log(n2o_eq) ~ 1 +
               (1 | a | WSA9) +
               (1 | b | WSA9:state) +
               (1 | c | WSA9:state:size_cat),
             family = gaussian())

# Set priors for parameters
priors <- c(
  prior(normal(2, 1), class = "Intercept", resp = "logn2o"), # alpha_0
  prior(exponential(2), class = "sd", resp = "logn2o"), # sd alpha_1..3
  prior(exponential(2), class = "sigma", resp = "logn2o"), # observation level sd
  prior(normal(2, 1), class = "Intercept", resp = "logn2oeq"),
  prior(exponential(2), class = "sd", resp = "logn2oeq"),
  prior(exponential(2), class = "sigma", resp = "logn2oeq"),
  prior(lkj(2), class = "rescor"), # observation level residual correlation
  prior(lkj(2), class = "cor") # correlation for group effects
  )

# Fit the multivariate model
n2o_mod1 <- brm(bf_n2o + bf_n2oeq + set_rescor(rescor = TRUE), ...)
```

The first two objects assigned in the above code example are the linear predictors assigned to the dissolved and equilibrium N2O responses. The second object is a list of priors as defined above. The third object results from the `brm()` call to fit the model. In this syntax, the two linear predictor objects are “added” along with the `set_rescor(rescor = TRUE)` option, which defines whether the residual correlation is to be modeled (TRUE) or not (FALSE). Note that, with `set_rescor(rescor = FALSE)` and without the correlated group effects (i.e., ‘a’, ‘b’, ‘c’), **brms** would effectively treat the two responses as entirely independent in fitting.

For the remainder of this document, only this simplified syntax is presented to describe the model structure. For more information on **brms** functionality and syntax with multivariate response models, see the package vignette: https://cran.r-project.org/web/packages/brms/vignettes/brms_multivariate.html.

The relevant code for the final model used for inference and described in the main paper is below:

```
# Assign separate linear predictors for each response: N2O, N2o-eq, Surface temp, and

# N2O
bf_n2o <- bf(n2o ~ mo(no3_cat) +
               log_area +
               surftemp +
```

```
                 mo(no3_cat):log_area +
                 mo(no3_cat):surftemp +
                 (mo(no3_cat) | a | WSA9) +
                 (mo(no3_cat) | b | WSA9:state) +
                 (1 | c | WSA9:state:size_cat),
               shape ~ log_area +
                 mo(no3_cat) +
                 (1 | WSA9) +
                 (1 | WSA9:state) +
                 (1 | WSA9:state:size_cat),
               family = Gamma(link = "log"))

# Equilibrium N2O
bf_n2oeq <- bf(n2o_eq ~ surftemp +
                 log_elev +
                 surftemp:log_elev +
                 (1 | a | WSA9) +
                 (1 | b | WSA9:state) +
                 (1 | c | WSA9:state:size_cat),
               shape ~ surftemp +
                 log_elev +
                 (1 | WSA9) +
                 (1 | WSA9:state) +
                 (1 | WSA9:state:size_cat),
               family = Gamma(link = "log"))

# Surface temperature
bf_temp <- bf(surftemp ~ lat +
                s(log_elev) +
                s(jdate) +
                (1 | a | WSA9) +
                (1 | b | WSA9:state) +
                (1 | c | WSA9:state:size_cat),
               shape ~ lat,
               family = Gamma(link = "log"))

# NO3 level
bf_no3 <- bf(no3_cat ~ surftemp +
               log_area +
               (1 | a | WSA9) +
               (1 | b | WSA9:state) +
               (1 | c | WSA9:state:size_cat),
             family = cumulative(link = "logit", threshold="flexible"))

# Assign priors
priors <- c( # N2O
  prior(normal(2, 1), class = "Intercept", resp = "n2o"),
  prior(normal(0, 1), class = "b", resp = "n2o"),
  prior(exponential(2), class = "sd", resp = "n2o"),
```

```
  prior(normal(5, 4), class = "Intercept", dpar = "shape", resp = "n2o"),
  prior(normal(0, 1), class = "b", dpar = "shape", resp = "n2o"),
  prior(exponential(2), class = "sd", dpar = "shape", resp = "n2o"),

  # Equil N2O
  prior(normal(2, 1), class = "Intercept", resp = "n2oeq"),
  prior(normal(0, 1), class = "b", resp = "n2oeq"),
  prior(exponential(2), class = "sd", resp = "n2oeq"),
  prior(normal(5, 4), class = "Intercept", dpar = "shape", resp = "n2oeq"),
  prior(normal(0, 1), class = "b", dpar = "shape", resp = "n2oeq"),
  prior(exponential(2), class = "sd", dpar = "shape", resp = "n2oeq"),

  # Surface temp
  prior(normal(3, 1), class = "Intercept", resp = "surftemp"),
  prior(normal(0, 1), class = "b", resp = "surftemp"),
  prior(exponential(0.5), class = "sds", resp = "surftemp"),
  prior(exponential(2), class = "sd", resp = "surftemp"),
  prior(normal(5, 4), class = "Intercept", dpar = "shape", resp = "surftemp"),
  prior(normal(0, 1), class = "b", dpar = "shape", resp = "surftemp"),

  # NO3
  prior(normal(0, 3), class = "Intercept", resp = "no3cat"),
  prior(normal(0, 1), class = "b", resp = "no3cat"),
  prior(exponential(1), class = "sd", resp = "no3cat"),

  prior(lkj(2), class = "cor")
  )

# Fit the multivariate model
n2o_mod6 <- brm(bf_n2o + bf_n2oeq + bf_temp + bf_no3 + set_rescor(rescor = FALSE), ..
```

In this final model, the dissolved and equilibrium N2O responses were each modeled with Gamma distributed errors. The Gamma error structure appeared to result in slightly better performance in the predictive checks compared to the Gaussian errors. This was primarily apparent in the saturation ratio checks, which may have been more sensitive to model performance in the tails of the N2O responses. Others have indicated that the Gamma error distribution can work well for modeling dissolved N2O data (Webb et al. 2019). The linear predictor for dissolved N2O included the survey design effects along with covariate terms for lake area (ha), NO3 level, and surface water temperature. To accomodate heterogeneous errors, the shape parameter of the Gamma distribution was modeled as a function of NO3 level, lake area, and the survey design factors. The equilibrium N2O response was modeled as a function of the survey design factors, lake elevation, and lake surface temperature. The shape parameter was, again, allowed to vary conditional on the survey factors, surface temperature, and elevation.

The sub-model for the surface temperature response also assumed a Gamma distributed error distribution and covariates included the survey design variables, latitude, elevation, and Julian date. The shape parameter was also modeled as a function of latitude to address increasing temperature variance along the latitudinal gradient.

The NO3 sub-model was a cumulative logit model and the linear predictor included all of the survey factors as well as surface temperature and lake area.

Note that there was no observation-level residual correlation term for this final model, since the residuals are undefined for the Gamma and cumulative logit models. Dropping the observation-level residual correlation term was deemed a reasonable compromise that enabled the inclusion of NO3 as a covariate on dissolved N2O. The random intercepts, however, still allowed for potential correlations between the four responses at the group levels. As mentioned previously, including covariates that were not available in the full POI necessitated some additional steps in the poststratification process. To make population-level inferences from a model with covariates, those covariates need to be (1) fully observed across the POI or (2) their “missingness” needs to be addressed with modeling. For the lake area and elevation covariates, for example, data was available for the entire POI from previously compiled geospatial databases. However, neither surface temperature or NO3 were observed for lakes outside of the sample. That is, they were partially observed with respect to the POI. Therefore, the final multivariate model, coded above included these as additional responses, with each also being conditioned on the survey design structure and the fully observed covariates. The dependence structure among the fully and partially observed variables in this model could be described such that:

$$\begin{aligned}
p(\boldsymbol{N_2O_{diss}} &| Survey, Area, \boldsymbol{NO_3}, \boldsymbol{Temp}) \\
p(\boldsymbol{N_2O_{equil}} &| Survey, Elev, \boldsymbol{Temp}) \\
p(\boldsymbol{NO_3} &| Survey, Area, \boldsymbol{Temp}) \\
p(\boldsymbol{Temp} &| Survey, Lat, Elev, Day)
\end{aligned}$$

Variables in bold text above were partially observed with respect to the POI (i.e., observed only in the sample), whereas variables not in bold were considered fully observed. The partially observed variables, being dissolved and equilibrium N2O, NO3, and surface temperature, were each modeled conditional on the survey design variables and other partially and/or fully observed covariates. This piece-wise approach then required a more complex set of post-processing steps compared to a typical MRP analysis. In order to propagate uncertainty through to the population-level inferences, the fitted model was first used to predict surface temperature in the target population, since it depended only on the fully observed covariates. That predictive distribution was then used alongside the relevant

fully observed covariates to predict NO3 in the target population. Finally, these predictive distributions for temperature and NO3 were used to predict the N2O responses. These steps were carried out in the "Predict to population" section below. This approach is similar to MRP approaches with "non-census" variables (Kennedy and Gelman 2015). It could also be described as a form of Bayesian structural equation model (Merkle et al. 2021; Merkle and Rosseel 2018).

Finally, because N2O flux was to be later estimated as a function of lake-level measures (*e.g.*, surface area), predictions for N2O were made to each of the 465,897 individual lakes in the POI. For population level estimates of dissolved N2O, equilibrium N2O, and N2O saturation, the lake-level predictions were summarized across the relevant units (*e.g.*, CONUS, ecoregion). Predictions were assumed relevant for average conditions during the biological index period of 2017.

All of the models were constructed and assessed within in `R` (R Core Team 2021) using the `brms` package (Bürkner 2017) as an interface to Stan, a software package for fitting fully Bayesian models via Hamiltonian Monte Carlo (HMC, Stan Development Team 2018b, 2018c, 2018a). More specific details on the model structures and fits is provided in the "Model fitting" section below.

2 Data

As explained in the data munging document document (https://github.com/USEPA/DissolvedGasNla/blob/master/scripts/dgIndicatorAnalysis.html), duplicate dissolved gas samples were collected at a depth of ~0.1m at designated index sites distributed across 1091 lakes nationwide, of which 95 were sampled twice as repeat visits. This subset of revisit sites was used as a test set for assessing model fit and out-of-sample performance.

Water samples were analyzed via gas chromotography and concentrations were recorded to the nearest 0.001 nmol/L. As part of the survey design, each gas observation was indexed to an individual lake selected with unequal probability from 5 different lake size categories, $j \in j = 1, ..., J = 5$, according to surface area (ha), and from within a state, $k \in k = 1, ..., K = 48$, situated within an aggregated, WSA9 or Omernik ecoregion, $l \in l = 1, ..., L = 9$. All WSA9 ecoregions were represented, including the Xeric (XER), Western Mountain (WMT), Northern Plains (NPL), Southern Plains (SPL), Temperate Plains (TPL), Coastal Plains (CPL), Upper Midwest (UMW), Northern Appalachian (NAP), and Southern Appalachian (SAP) regions. Gas data from the first and second (follow-up) visits

were separated, with $n = 984$ *vs.* $n = 95$ observations each. In addition to the design variables, a number of potentially useful covariates were also indexed to the gas observations, including measures of NO3, surface water temperature, elevation, chlorophyll a content, dissolved oxygen content, and waterbody size (hectares). These gas data and covariates were previously described and munged at: https://github.com/USEPA/DissolvedGasNla/blob/master/scripts/dataMunge.html.

2.1 Import

That originally munged gas dataset is imported below.

```
load("./inputData/dg.RData")
```

A new data frame for modeling was constructed from the original file, including only the variables of interest: (1) the N2O gas observations; (2) the survey design variables indexed to those observations; and (3) measures and metrics considered potentially useful as covariates for improving the fit and/or practical interpretation of the model. The modeling data frame below excluded the second-visit observations, which would later be used for model checking. Some variables from the imported data were renamed for convenience. In addition, the NO3 covariate was rounded according to the documented measurement precision and an alternative version was created by log-transforming and re-coding the variable as an ordered factor with five levels. The left-most cut point separated observations below the detection limit from the completely observed samples. The remaining cut points were drawn at equal distances in the positive direction along the log scale.

```
dg %>%
  filter(sample.source == "DG") %>%
  nrow() # number of dissolved gas observations before filtering out probability site
```

```
## [1] 1185
```

```
df_model <- dg %>%
  filter(sample.source == "DG", # dissolved gas info
         sitetype == "PROB") %>% # probability samples only
  filter(visit.no == 1) %>% # first-visit sites only
  mutate(n2o = round(dissolved.n2o.nmol, 2),
         n2o_eq = round(sat.n2o.nmol, 2),
         n2o_sat = n2o.sat.ratio,
```

```r
        n2o_em = e.n2o.nmol.d,
        n2o_flux = f.n2o.m.d,
        WSA9 = factor(ag.eco9),
        state = factor(state.abb[match(state.nm, state.name)]),
        area_ha = area.ha,
        log_area = log(area_ha),
        chla = chla.result,
        log_chla = log(chla),
        elev = elevation,
        log_elev = log(elev + 1),
        do_surf = o2.surf,
        log_do = log(do_surf),
        bf_max = max.bf,
        sqrt_bf = sqrt(bf_max),
        size_cat = recode(area.cat6, # simpler naming conventions for the size categ
                          "(1,4]" = "min_4" ,
                          "(10,20]" = "10_20",
                          "(20,50]" = "20_50",
                          "(4,10]" = "4_10",
                          ">50" = "50_max")) %>%
mutate(size_cat = factor(size_cat,
                          levels = c("min_4", "4_10", "10_20", "20_50", "50_max"),
                          ordered = TRUE)) %>%
mutate(no3 = ifelse(nitrate.n.result <= 0.0005, 0.0005, round(nitrate.n.result, 4))
mutate(no3_cat = cut(log(no3), # convert no3 to ordered factor with 5 levels
                      breaks = c(-Inf, -7.5, -5.5, -3.5, -1.5, Inf),
                      labels =seq(1, 5, 1))) %>%
mutate(no3_cat = factor(no3_cat,
                         levels = seq(1, 5, 1),
                         ordered = TRUE)) %>%
mutate(date = as.Date(date.col)) %>%
mutate(jdate = as.numeric(format(date, "%j"))) %>%
mutate(lat = map.lat.dd,
       lon = map.lon.dd) %>% # longitude
mutate(surftemp = surftemp,
       log_surftemp = log(surftemp)) %>%
select(WSA9,
       state,
       size_cat,
       site.id,
       lat,
       lon,
       date,
       jdate,
       surftemp,
       log_surftemp,
       area_ha,
       log_area,
       elev,
```

```
        log_elev,
        chla,
        log_chla,
        do_surf,
        log_do,
        bf_max,
        sqrt_bf,
        n2o,
        n2o_eq,
        no3,
        no3_cat
        )

save(df_model, file = "/modelFiles/df_model.rda")
```

A preview of the 984 probability samples to be used in modeling:

```
df_model %>%
  head()
```

```
## # A tibble: 6 × 24
##   WSA9  state size_cat site.id          lat   lon date       jdate surftemp
##   <fct> <fct> <ord>    <chr>          <dbl> <dbl> <date>     <dbl>    <dbl>
## 1 SAP   AL    50_max   NLA17_AL-10001  33.3 -87.4 2017-07-10   191     23.9
## 2 SAP   AL    50_max   NLA17_AL-10002  33.7 -86.2 2017-08-14   226     28.1
## 3 CPL   AL    50_max   NLA17_AL-10003  32.5 -87.8 2017-07-12   193     27.2
## 4 CPL   AL    20_50    NLA17_AL-10004  31.6 -88.4 2017-07-14   195     29
## 5 CPL   AL    4_10     NLA17_AL-10005  33.4 -88.2 2017-07-11   192     28
## 6 CPL   AL    10_20    NLA17_AL-10008  32.2 -87.8 2017-07-13   194     29
## # ℹ 15 more variables: log_surftemp <dbl>, area_ha <dbl>, log_area <dbl>,
## #   elev <dbl>, log_elev <dbl>, chla <dbl>, log_chla <dbl>, do_surf <dbl>,
## #   log_do <dbl>, bf_max <dbl>, sqrt_bf <dbl>, n2o <dbl>, n2o_eq <dbl>,
## #   no3 <dbl>, no3_cat <ord>
```

A second data frame, including only the second visit observations was constructed below. These data were later used as a “test set” to assess the out-of-sample performance of the model developed on the first-visit or “training set”.

```
df_test <- dg %>%
  filter(sample.source == "DG", # dissolved gas info
         sitetype == "PROB") %>% # probability samples only
  filter(visit.no == 2) %>% # second-visit sites only
  mutate(n2o = round(dissolved.n2o.nmol, 2),
         n2o_eq = round(sat.n2o.nmol, 2),
```

```r
        n2o_sat = n2o.sat.ratio,
        n2o_em = e.n2o.nmol.d,
        n2o_flux = f.n2o.m.d,
        WSA9 = factor(ag.eco9),
        state = factor(state.abb[match(state.nm, state.name)]),
        area_ha = area.ha,
        log_area = log(area_ha),
        chla = chla.result,
        log_chla = log(chla),
        elev = elevation,
        log_elev = log(elev + 1),
        do_surf = o2.surf,
        log_do = log(do_surf),
        bf_max = max.bf,
        sqrt_bf = sqrt(bf_max),
        size_cat = recode(area.cat6, # simpler naming conventions for the size categ
                          "(1,4]" = "min_4" ,
                          "(10,20]" = "10_20",
                          "(20,50]" = "20_50",
                          "(4,10]" = "4_10",
                          ">50" = "50_max")) %>%
  mutate(size_cat = factor(size_cat,
                          levels = c("min_4", "4_10", "10_20", "20_50", "50_max"),
                          ordered = TRUE)) %>%
  mutate(no3 = ifelse(nitrate.n.result <= 0.0005, 0.0005, round(nitrate.n.result, 4))
  mutate(no3_cat = cut(log(no3), # convert no3 to ordered factor with 5 levels
                      breaks = c(-Inf, -7.5, -5.5, -3.5, -1.5, Inf),
                      labels =seq(1, 5, 1))) %>%
  mutate(no3_cat = factor(no3_cat,
                         levels = seq(1, 5, 1),
                         ordered = TRUE)) %>%
  mutate(date = as.Date(date.col)) %>%
  mutate(jdate = as.numeric(format(date, "%j"))) %>%
  mutate(lat = map.lat.dd,
        lon = map.lon.dd) %>% # longitude
  mutate(surftemp = surftemp,
        log_surftemp = log(surftemp)) %>%
  select(WSA9,
        state,
        size_cat,
        site.id,
        lat,
        lon,
        date,
        jdate,
        surftemp,
        log_surftemp,
        area_ha,
        log_area,
```

```
         elev,
         log_elev,
         chla,
         log_chla,
         do_surf,
         log_do,
         bf_max,
         sqrt_bf,
         n2o,
         n2o_eq,
         no3,
         no3_cat
         )

save(df_test, file = "modelFiles/df_test.rda")
```

A preview of the re-visit data with 95 observations:

```
df_test %>%
  head()
```

```
## # A tibble: 6 × 24
##   WSA9  state size_cat site.id          lat    lon date       jdate surftemp
##   <fct> <fct> <ord>    <chr>          <dbl>  <dbl> <date>     <dbl>    <dbl>
## 1 SAP   AL    50_max   NLA17_AL-10001  33.3  -87.4 2017-08-14   226     28.2
## 2 CPL   AL    20_50    NLA17_AL-10004  31.6  -88.4 2017-08-16   228     29
## 3 CPL   AR    50_max   NLA17_AR-10001  34.5  -92.3 2017-07-20   201     28.8
## 4 CPL   AR    4_10     NLA17_AR-10003  33.1  -92.7 2017-09-06   249     24.1
## 5 XER   AZ    50_max   NLA17_AZ-10001  33.6 -112.  2017-08-30   242     28
## 6 WMT   AZ    20_50    NLA17_AZ-10006  33.8 -109.  2017-08-08   220     20.2
## # ℹ 15 more variables: log_surftemp <dbl>, area_ha <dbl>, log_area <dbl>,
## #   elev <dbl>, log_elev <dbl>, chla <dbl>, log_chla <dbl>, do_surf <dbl>,
## #   log_do <dbl>, bf_max <dbl>, sqrt_bf <dbl>, n2o <dbl>, n2o_eq <dbl>,
## #   no3 <dbl>, no3_cat <ord>
```

2.2 Target population

Below, the 2017 NLA sampling frame was imported and filtered to include only lakes in the assumed POI. The resulting target population included a total of 465,897 waterbodies.

```
df_pop <- read.csv(archive::archive_read("inputData/NLA_Sample_Frame.7z")) # This tak

sframe <- df_pop %>%
```

```
  filter(nla17_sf != "Exclude2017") %>%
  filter(nla17_sf != "Exclude2017_Include2017NH") %>%
  filter(state != "DC") %>%
  filter(state != "HI") %>%
  droplevels() %>%
  mutate(WSA9 = factor(ag_eco9),
         WSA9 = forcats::fct_drop(WSA9), # remove NA level
         state = factor(state),
         size_cat = factor(area_cat6),
         lat = lat_dd83,
         lon = lon_dd83,
         log_area = log(area_ha),
         elev = elevation,
         log_elev = ifelse(elev <= 0, 0, elev), # assumed elev < 0 to be elev = 0
         log_elev = log(log_elev + 1)
         ) %>%
  mutate(size_cat = recode(size_cat,
                           "(1,4]" = "min_4" ,
                           "(10,20]" = "10_20",
                           "(20,50]" = "20_50",
                           "(4,10]" = "4_10",
                           ">50" = "50_max")) %>%
  mutate(size_cat = factor(size_cat,
                           levels = c("min_4", "4_10", "10_20", "20_50", "50_max"),
                           ordered = TRUE)) %>%
  select(WSA9, state, size_cat, lat, lon, area_ha, log_area, elev, log_elev)

rm(df_pop)

save(sframe, file = "C:/Users/rmartin/OneDrive - Environmental Protection Agency (EPA
```

Below is a sample of the first ten rows of the POI:

```
sframe %>%
  head(10)
```

```
## # A tibble: 10 × 9
##    WSA9  state size_cat   lat   lon area_ha log_area  elev log_elev
##    <fct> <fct> <ord>    <dbl> <dbl>   <dbl>    <dbl> <int>    <dbl>
##  1 NAP   MA    50_max    42.4 -72.3   9544.     9.16   159     5.08
##  2 NAP   VT    50_max    42.8 -72.9    812.     6.70   454     6.12
##  3 NAP   ME    50_max    44.9 -69.2   1888.     7.54    60     4.11
##  4 NAP   NY    50_max    44.3 -74.1    525.     6.26   465     6.14
##  5 NAP   NY    50_max    43.2 -74.1   9493.     9.16   234     5.46
##  6 CPL   VA    50_max    37.4 -76.8    361.     5.89    17     2.89
##  7 SAP   VA    50_max    37.3 -77.7   1317.     7.18    45     3.83
```

```
##   8 CPL   VA    50_max    36.8 -76.6    339.      5.83     8      2.20
##   9 SAP   NC    50_max    36.5 -78.9   1102.      7.01   131      4.88
##  10 CPL   NC    50_max    35.9 -77.9    287.      5.66    36      3.61
```

Cross tabulations below show the structure of the target population with respect to the survey design variables. The cross-tabulation shows that, of course, each ecoregion does not contain each state. Therefore, states were nested within ecoregions.

```
sframe %>%
  group_by(WSA9, state) %>%
  summarise(n = n(), .groups = "drop") %>%
  spread(state, n)
```

```
## # A tibble: 9 × 49
##   WSA9     AL    AR    AZ    CA    CO    CT    DE    FL    GA    IA    ID    IL
##   <fct> <int> <int> <int> <int> <int> <int> <int> <int> <int> <int> <int> <int>
## 1 CPL    7326  6395    NA    NA    NA    NA   529 37888 23761    NA    NA    44
## 2 NAP      NA    NA    NA    NA    NA  2143    NA    NA    NA    NA    NA    NA
## 3 NPL      NA    NA    NA    NA    NA    NA    NA    NA    NA    NA    NA    NA
## 4 SAP    3877  2362    NA    NA    NA    NA    10    NA  9014    NA    NA   275
## 5 SPL      NA    NA    NA    NA  2006    NA    NA    NA    NA    NA    NA    NA
## 6 TPL      NA    NA    NA    NA    NA    NA    NA    NA    NA  5015    NA 10308
## 7 UMW      NA    NA    NA    NA    NA    NA    NA    NA    NA   205    NA    68
## 8 WMT      NA    NA   332  4261  2958    NA    NA    NA    NA    NA  1998    NA
## 9 XER      NA    NA   578  5043   606    NA    NA    NA    NA    NA   923    NA
## # ℹ 36 more variables: IN <int>, KS <int>, KY <int>, LA <int>, MA <int>,
## #   MD <int>, ME <int>, MI <int>, MN <int>, MO <int>, MS <int>, MT <int>,
## #   NC <int>, ND <int>, NE <int>, NH <int>, NJ <int>, NM <int>, NV <int>,
## #   NY <int>, OH <int>, OK <int>, OR <int>, PA <int>, RI <int>, SC <int>,
## #   SD <int>, TN <int>, TX <int>, UT <int>, VA <int>, VT <int>, WA <int>,
## #   WI <int>, WV <int>, WY <int>
```

The cross-tablulation below indicates that lake size category was nested within state (which was nested in ecoregion). That is, not every ecoregion:state contained every size category.

```
sframe %>%
  group_by(WSA9, state, size_cat) %>%
  summarise(n = n(), .groups = "drop") %>%
  spread(size_cat, n)
```

```
## # A tibble: 110 × 7
##    WSA9  state min_4 `4_10` `10_20` `20_50` `50_max`
```

```
##    <fct> <fct> <int>  <int>   <int>   <int>    <int>
##  1 CPL   AL     5812   1078     253     119       64
##  2 CPL   AR     4178   1246     473     314      184
##  3 CPL   DE      380     72      42      28        7
##  4 CPL   FL    27613   6138    1918    1294      925
##  5 CPL   GA    20135   2814     481     238       93
##  6 CPL   IL       25     14      NA       2        3
##  7 CPL   KY      398     45      20       9       NA
##  8 CPL   LA    14429   3670    1163     699      636
##  9 CPL   MA      422    155      87      44       44
## 10 CPL   MD     1111    234      75      41       11
## # ℹ 100 more rows
```

Below, the sampling frame was used to create a typical post-stratification table. There were 536 “types” or groupings of lakes in the POI with respect to the sampling design. The total counts of those lake types (n_lakes) and their proportions (prop_cell) relative to the counts in the target population were tabulated.

```
pframe <- sframe %>%
  mutate(obs = 1) %>%
  group_by(WSA9, state, size_cat) %>%
  summarise(n_lakes = sum(obs), .groups = "drop") %>%
  ungroup() %>%
  mutate(prop_cell = n_lakes/sum(n_lakes)) %>%
  mutate(type = "population")

save(pframe, file = "C:/Users/rmartin/OneDrive - Environmental Protection Agency (EPA
```

```
pframe %>%
  head(10)
```

```
## # A tibble: 10 × 6
##    WSA9  state size_cat n_lakes prop_cell type
##    <fct> <fct> <ord>      <dbl>     <dbl> <chr>
##  1 CPL   AL    min_4       5812  0.0125   population
##  2 CPL   AL    4_10        1078  0.00231  population
##  3 CPL   AL    10_20        253  0.000543 population
##  4 CPL   AL    20_50        119  0.000255 population
##  5 CPL   AL    50_max        64  0.000137 population
##  6 CPL   AR    min_4       4178  0.00897  population
##  7 CPL   AR    4_10        1246  0.00267  population
##  8 CPL   AR    10_20        473  0.00102  population
```

```
##  9 CPL   AR    20_50         314  0.000674 population
## 10 CPL   AR    50_max        184  0.000395 population
```

2.3 Sample vs. population

Below, the distribution of waterbody types in the POI were compared to their proportions in the observed sample. There were 352 waterbody types in the sample compared to the 536 in the POI. There were 984 observations distributed across these 352 lake types in the sample; and the number of samples was not distributed evenly across the types. Some cells were represented by as few as 1 lake. In total, 536-352 = 184 lake types in the population of interest were not represented in the sample.

```
samp_props <- df_model %>%
  mutate(obs = 1) %>%
  group_by(WSA9, state, size_cat) %>%
  summarize(n_lakes = sum(obs), .groups = "drop") %>%
  ungroup() %>%
  mutate(prop_cell = round(n_lakes / sum(n_lakes), 7)) %>%
  mutate(type = "sample")

save(samp_props, file = "C:/Users/rmartin/OneDrive - Environmental Protection Agency
```

```
samp_props %>%
  print(n = 10)
```

```
## # A tibble: 352 × 6
##    WSA9  state size_cat n_lakes prop_cell type
##    <fct> <fct> <ord>      <dbl>     <dbl> <chr>
##  1 CPL   AL    min_4          1   0.00102 sample
##  2 CPL   AL    4_10           1   0.00102 sample
##  3 CPL   AL    10_20          1   0.00102 sample
##  4 CPL   AL    20_50          1   0.00102 sample
##  5 CPL   AL    50_max         1   0.00102 sample
##  6 CPL   AR    min_4          1   0.00102 sample
##  7 CPL   AR    4_10           1   0.00102 sample
##  8 CPL   AR    10_20          3   0.00305 sample
##  9 CPL   AR    20_50          1   0.00102 sample
## 10 CPL   AR    50_max         1   0.00102 sample
## # ℹ 342 more rows
```

Below, a graphical comparison was constructed to depict the distribution of cells in the POI *vs.* those in the sample.

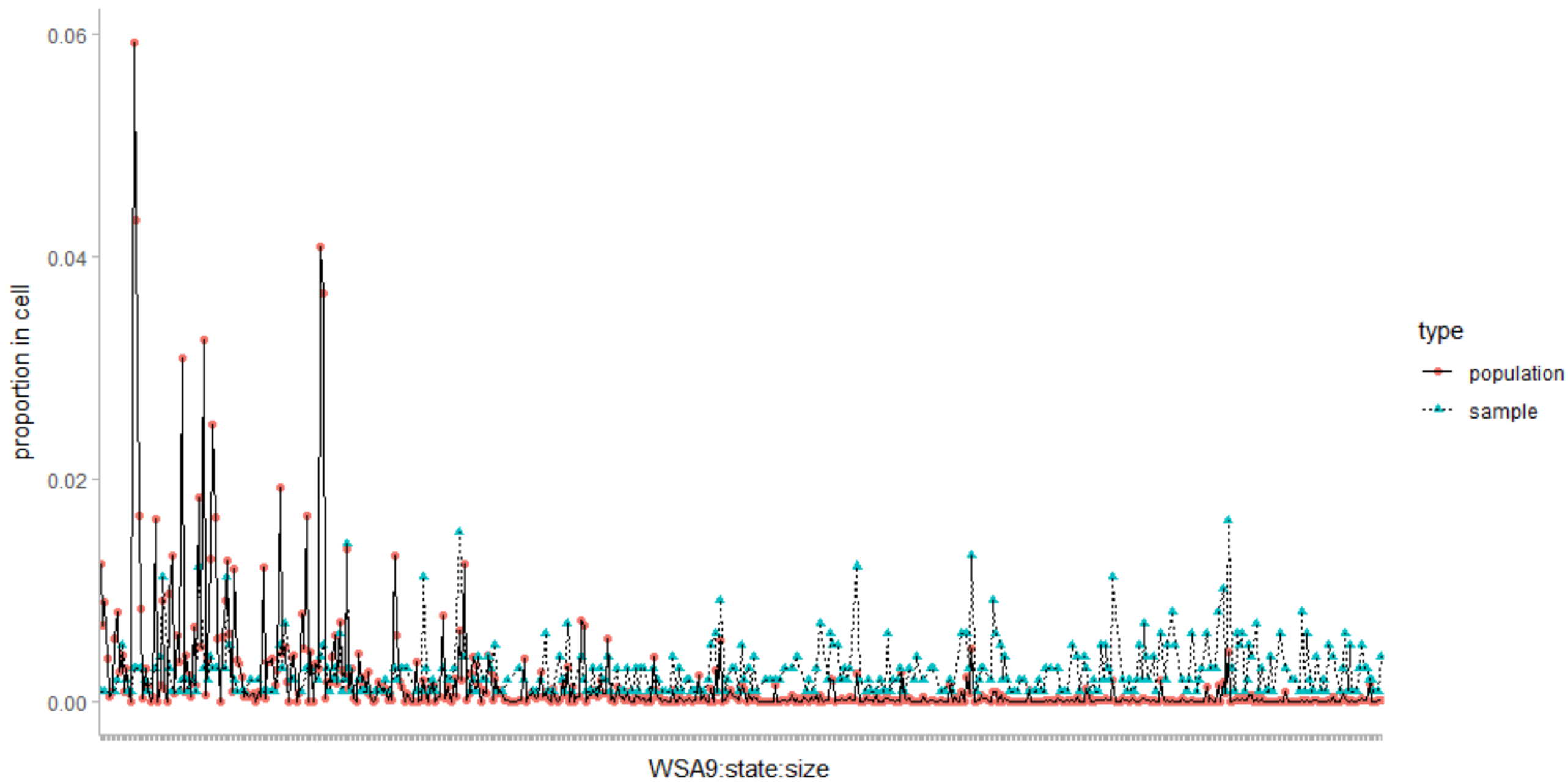

Another comparison between population and sample was constructed by ecoregion below. Lakes in the Coastal Plains (CPL) ecoregion, for example, were clearly undersampled relative to their proportion of the population.

```
pframe_eco <- pframe %>%
  group_by(WSA9) %>%
  summarise(n_lakes = sum(n_lakes)) %>%
  ungroup() %>%
  mutate(prop_cell = round(n_lakes/sum(n_lakes), 7)) %>%
  ungroup() %>%
  mutate(type = 'population')

samp_props_eco <- samp_props %>%
  group_by(WSA9) %>%
  summarise(n_lakes = sum(n_lakes)) %>%
  ungroup() %>%
  mutate(prop_cell = round(n_lakes/sum(n_lakes), 7)) %>%
  ungroup() %>%
  mutate(type = 'sample')

pframe_eco %>%
  bind_rows(samp_props_eco) %>%
  ggplot(mapping = aes(x = WSA9, y = prop_cell, group = type, linetype = type)) +
  geom_point(stat = "identity", aes( shape = type, color = type), size = 3) +
  geom_line() +
  theme_tidybayes() +
  xlab("Ecoregion") +
  ylab("proportion in cell") +
```

```
  theme(legend.position = "top",
        legend.title = element_blank(),
        legend.text = element_text(size = 14)) +
  theme(text = element_text(size = 12))
```

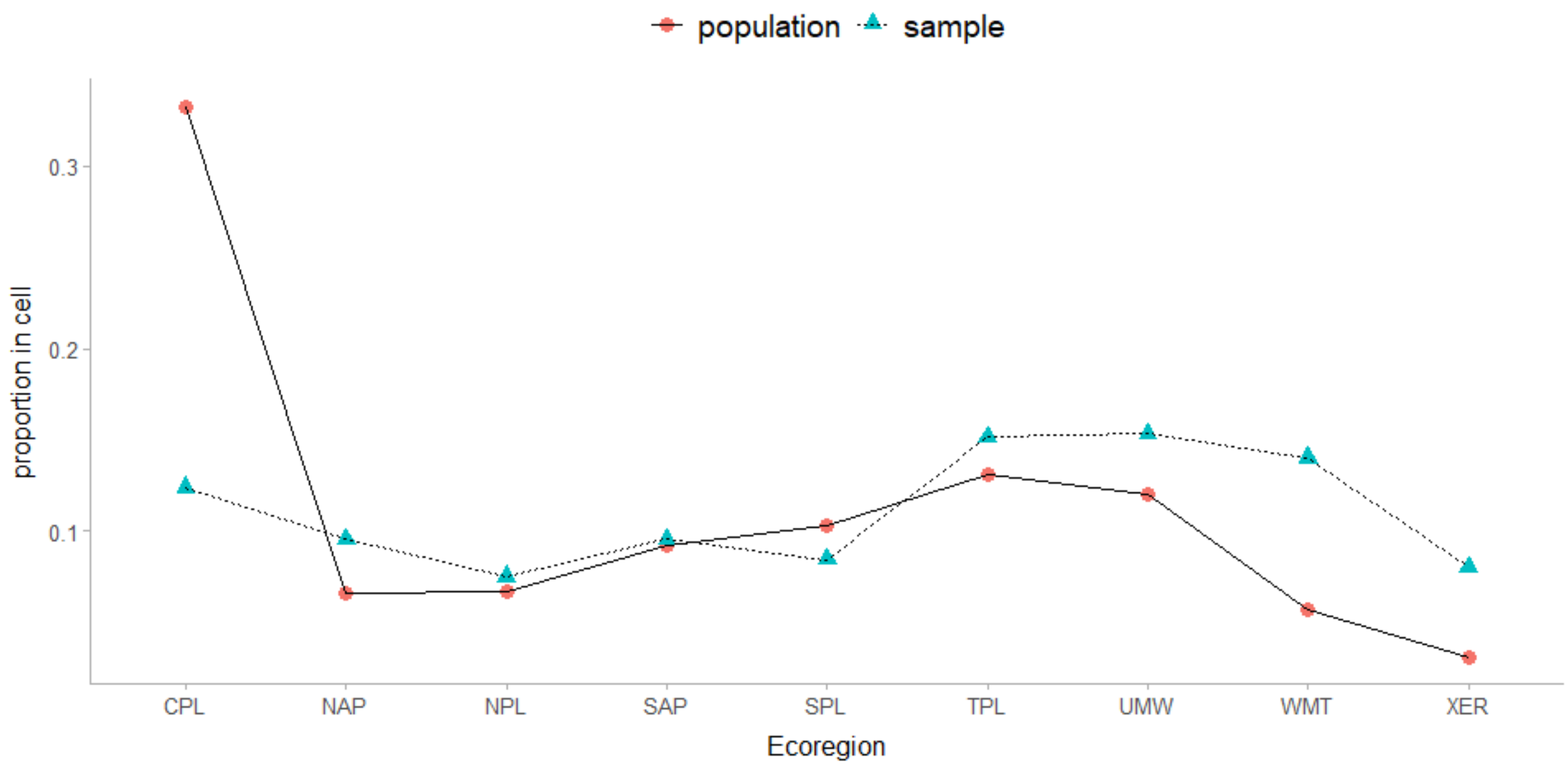

A similar comparison by state was constructed below.

```
pframe_state <- pframe %>%
  group_by(state) %>%
  summarise(n_lakes = sum(n_lakes)) %>%
  ungroup() %>%
  mutate(prop_cell = round(n_lakes/sum(n_lakes), 7)) %>%
  ungroup() %>%
  mutate(type = 'population')

samp_props_state <- samp_props %>%
  group_by(state) %>%
  summarise(n_lakes = sum(n_lakes)) %>%
  ungroup() %>%
  mutate(prop_cell = round(n_lakes/sum(n_lakes), 7)) %>%
  ungroup() %>%
  mutate(type = 'sample')

pframe_state %>%
  bind_rows(samp_props_state) %>%
  ggplot(mapping = aes(x = state, y = prop_cell, group = type, linetype = type)) +
  geom_point(stat = "identity", aes(shape = type, color = type)) +
  geom_line() +
  theme_tidybayes() +
  theme(axis.text.x = element_text(angle = 45)) +
```

```
  xlab("State") +
  ylab("proportion in cell")
```

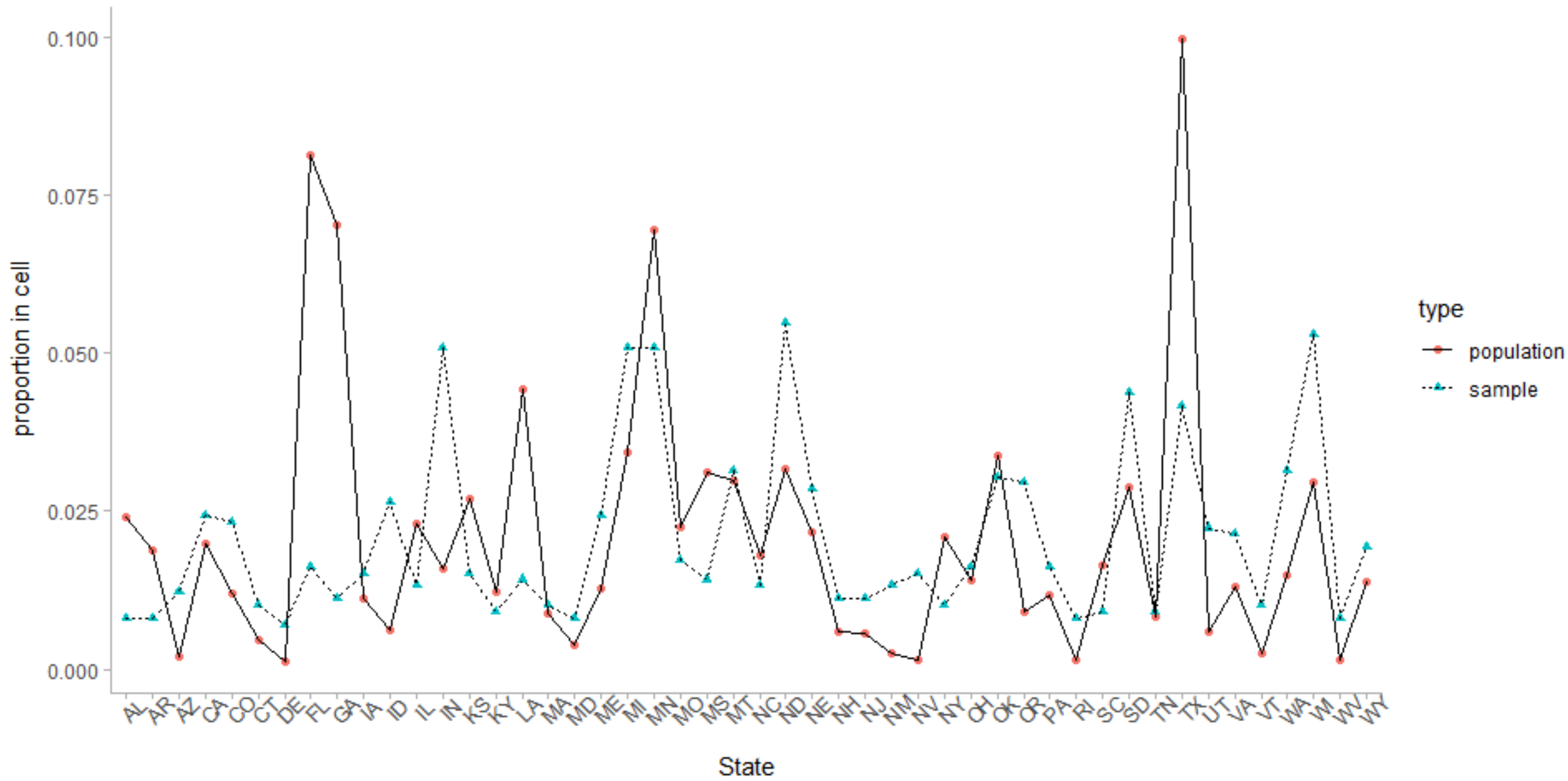

Finally, a comparison by NLA size category is shown below. Waterbodies of the smallest size category were heavily under-sampled relative to the POI and those from the larger size categories were over-sampled.

```
pframe_size <- pframe %>%
  group_by(size_cat) %>%
  summarise(n_lakes = sum(n_lakes)) %>%
  ungroup() %>%
  mutate(prop_cell = round(n_lakes/sum(n_lakes), 7)) %>%
  ungroup() %>%
  mutate(type = 'population')

samp_props_size <- samp_props %>%
  group_by(size_cat) %>%
  summarise(n_lakes = sum(n_lakes)) %>%
  ungroup() %>%
  mutate(prop_cell = round(n_lakes/sum(n_lakes), 7)) %>%
  ungroup() %>%
  mutate(type = 'sample')

pframe_size %>%
  bind_rows(samp_props_size) %>%
  ggplot(mapping = aes(x = size_cat, y = prop_cell, group = type, linetype = type)) +
  geom_point(stat = "identity", aes( shape = type, color = type)) +
  geom_line() +
  theme_tidybayes() +
```

```
  xlab("Size category") +
  ylab("proportion in cell")
```

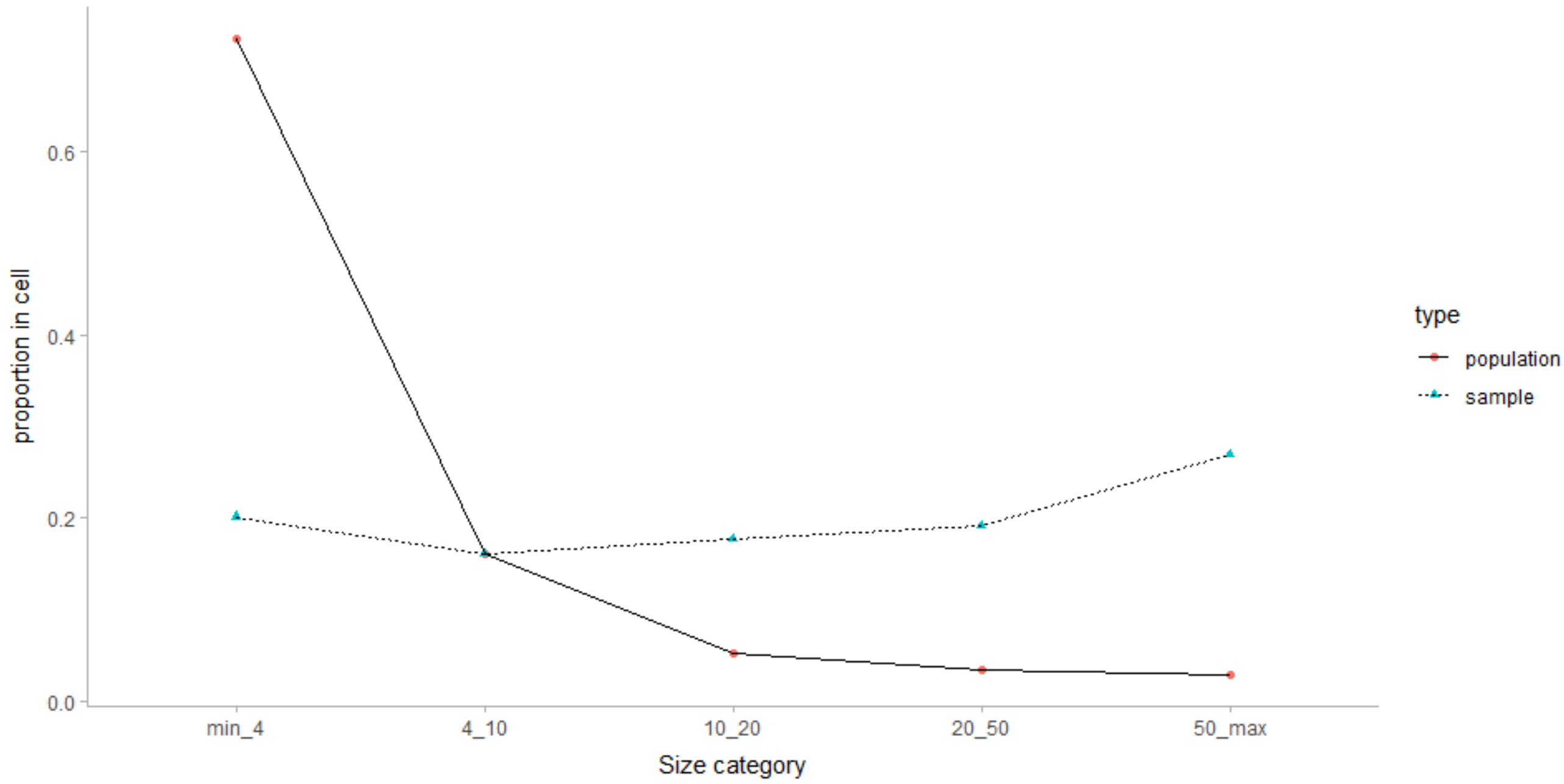

2.4 Sample-based estimates

Below are (naive) estimates, based only on the sample, for national means for dissolved and equilibrium N2O and the saturation ratio.

Dissolved N2O:

```
df_model %>%
  summarise(mean = mean(n2o),
            sd = sd(n2o))
```

```
## # A tibble: 1 × 2
##    mean    sd
##   <dbl> <dbl>
## 1  8.72  9.52
```

Equilibrium N2O:

```
df_model %>%
  summarise(mean = mean(n2o_eq),
            sd = sd(n2o_eq))
```

```
## # A tibble: 1 × 2
##    mean    sd
##   <dbl> <dbl>
## 1  7.48 0.845
```

Saturation ratio:

```
df_model %>%
  summarise(mean = mean(n2o / n2o_eq),
            sd = sd(n2o / n2o_eq))
```

```
## # A tibble: 1 × 2
##    mean    sd
##   <dbl> <dbl>
## 1  1.17  1.30
```

Roughly 67% of lakes in the sample were under-saturated (i.e., saturation ratio < 1):

```
df_model %>%
  summarise(prop_undersat = sum((n2o / n2o_eq) < 1) / 984)
```

```
## # A tibble: 1 × 1
##   prop_undersat
##           <dbl>
## 1         0.666
```

Using only the sample observations again, a plot was constructed of the overall mean (dashed line) along with the ecoregion-specific means (black circles). The shaded areas indicate +/- 1 standard deviation. Neither dissolved N2O nor the saturation ratio were clearly structured by ecoregion in the sample, but there did appear to be some potential structure along this variable in the equilibrium N2O observations.

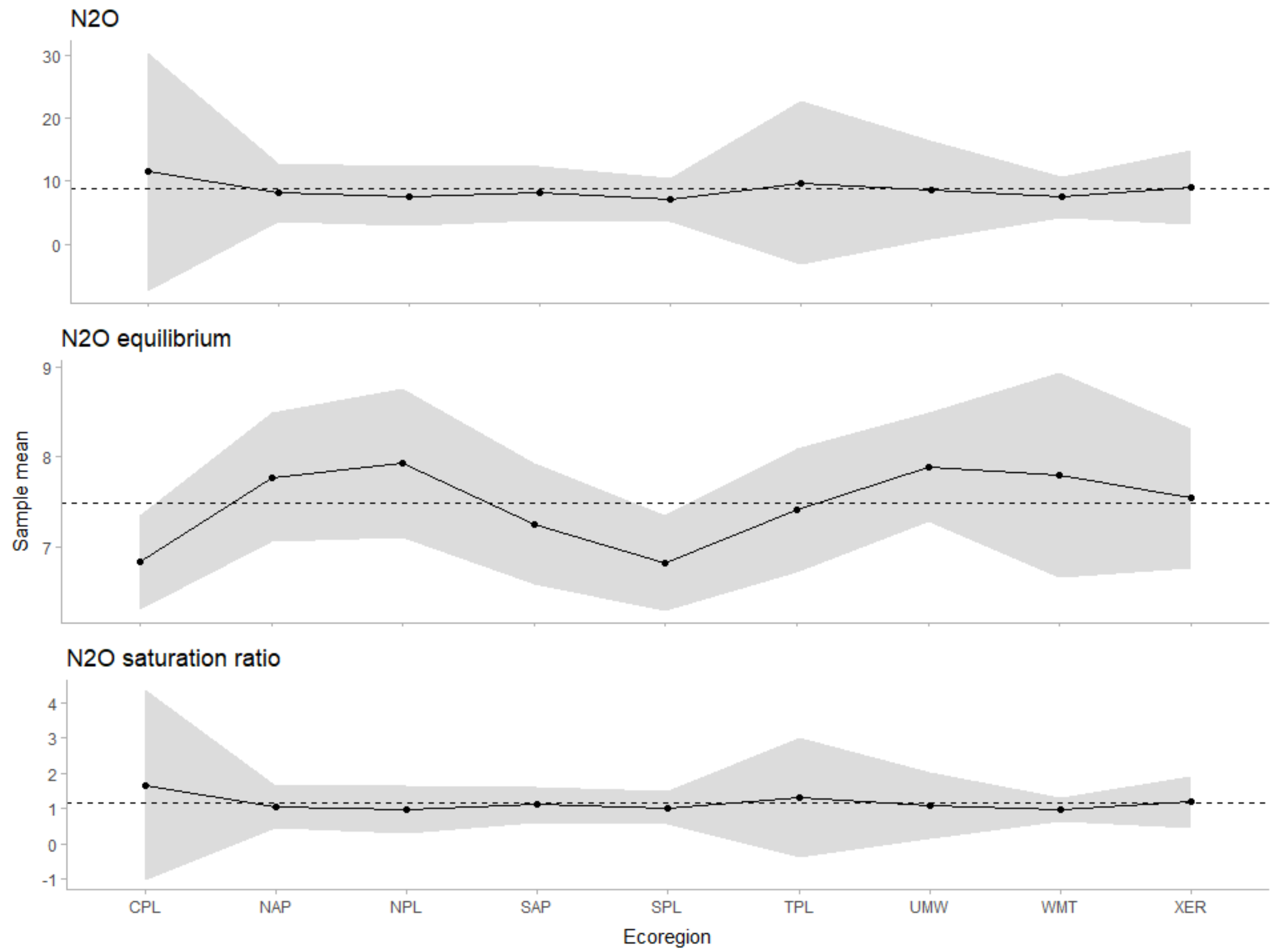

The same summary by state is below.

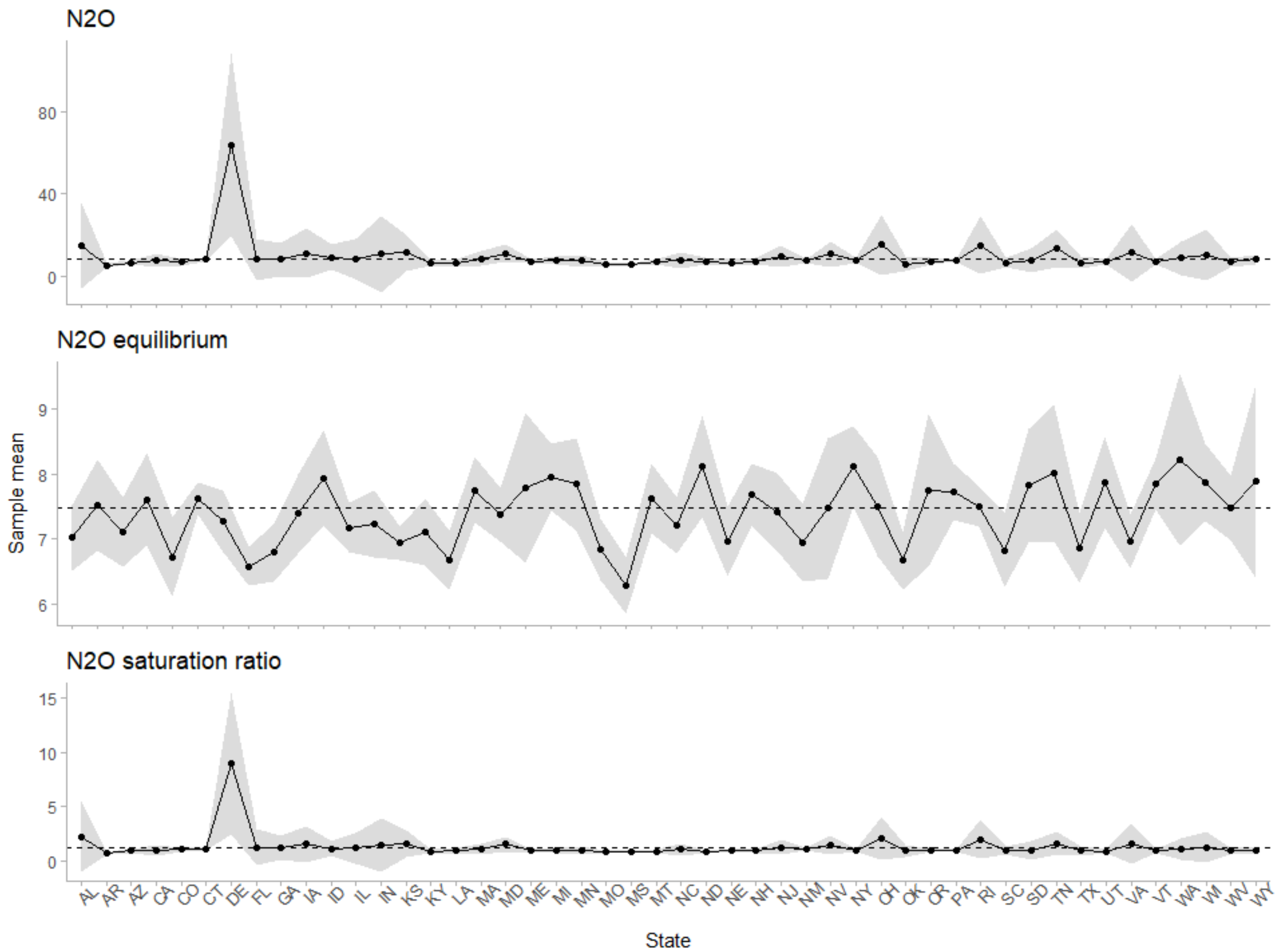

Finally, the same summary by size category.

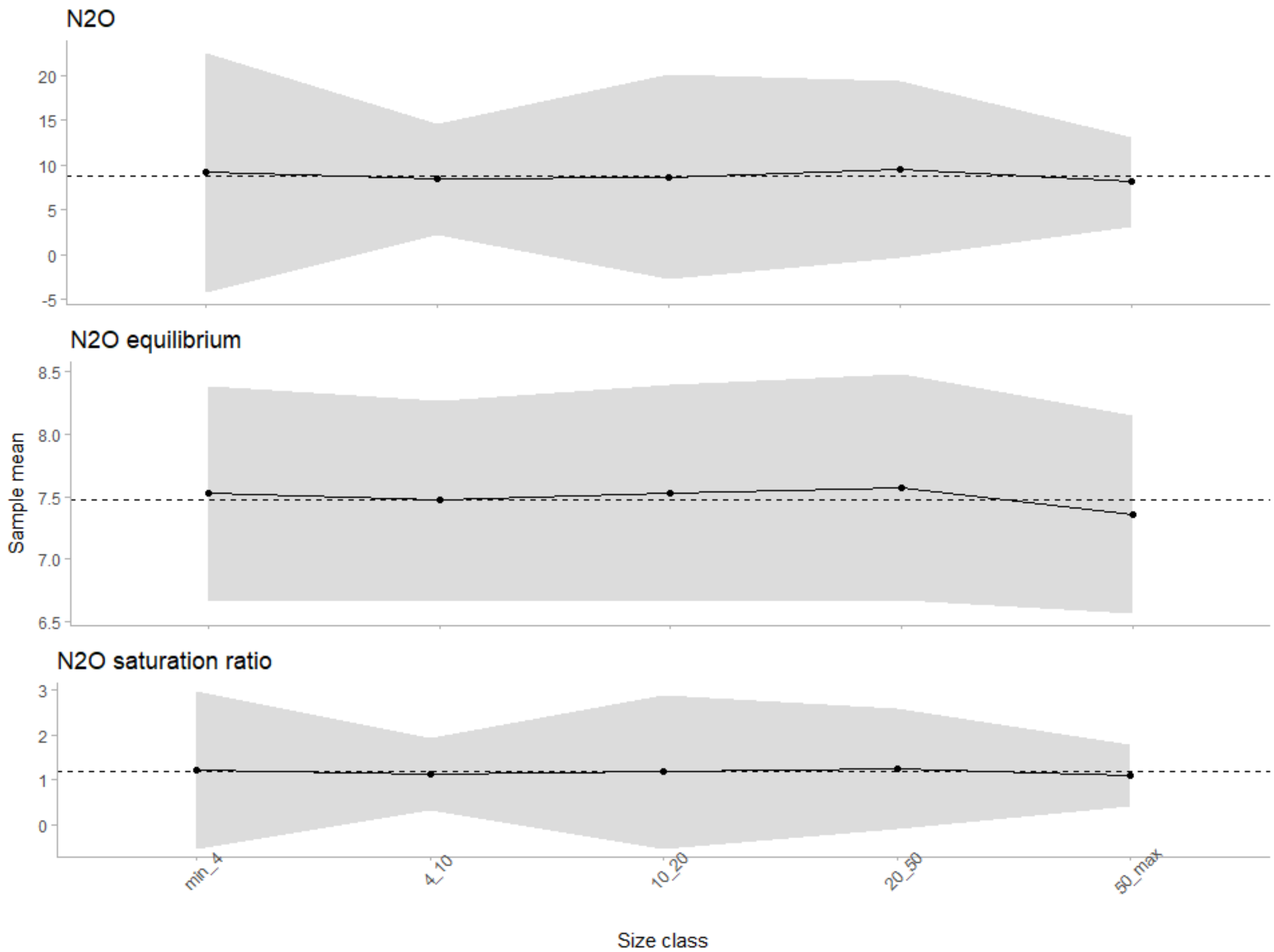

2.5 Sample data exploration

Below, the distribution N2O concentrations for the sample was summarized using a density and rug plot. Note the natural log scale of the x-axis. Both the dissolved and equilibrium N2O data had considerable right skew even after the log transformation. This was not unexpected and has been noted in other studies (*e.g.*, Webb et al. 2019). The saturation ratio was also skewed (sat_ratio = n2o / n2o_eq).

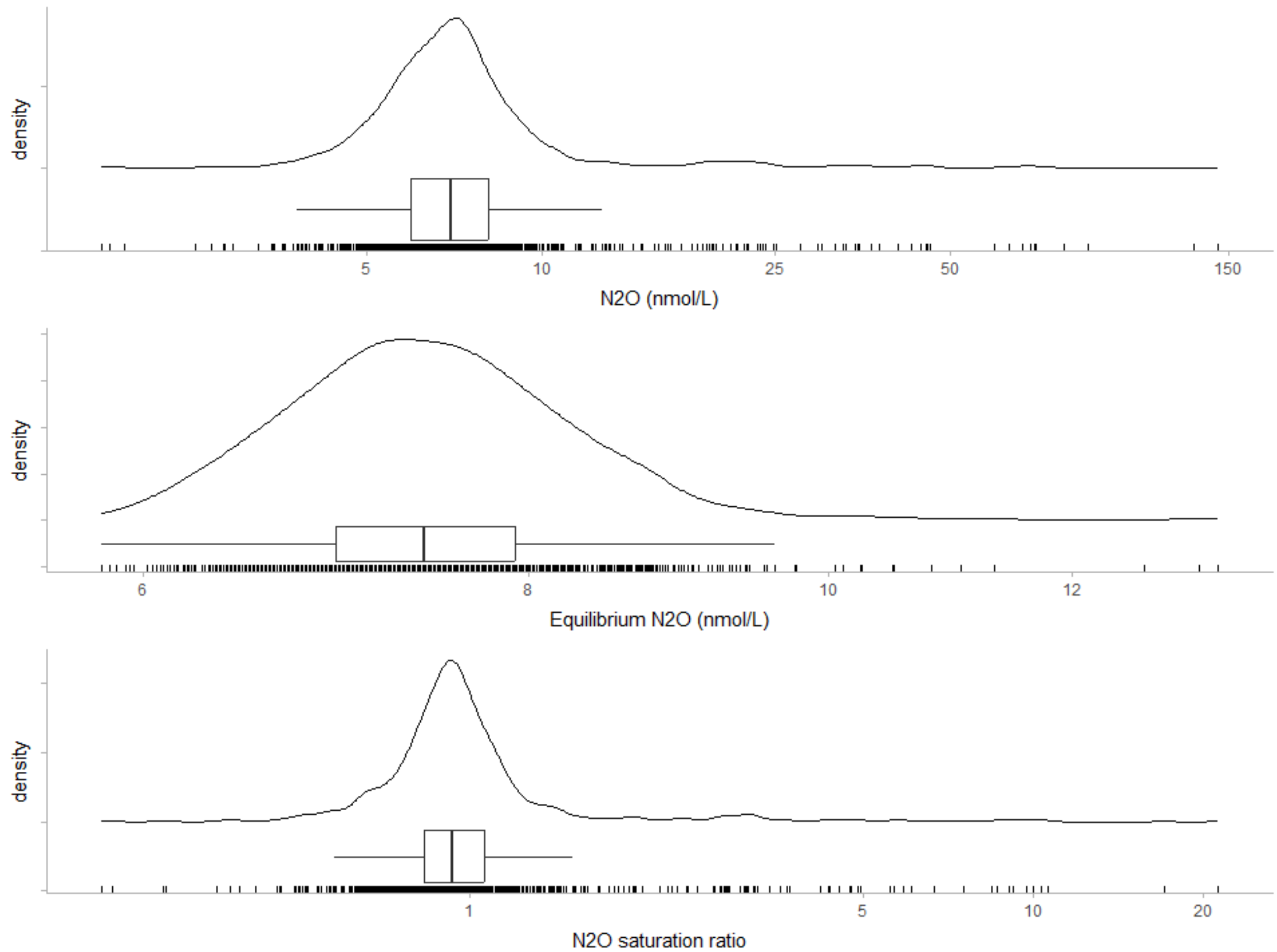

Below are plots of dissolved N2O vs. NO3 and some other potential covariates. This first plot shows log(N2O) vs. log(NO3), as well as the ordinal categories assigned to NO3 (vertical lines). The leftmost vertical line is dashed and separates the NO3 observations below the detection limit. The trend is increasing and nonlinear on the log scale, with increasing variance in N2O as NO3 increased.

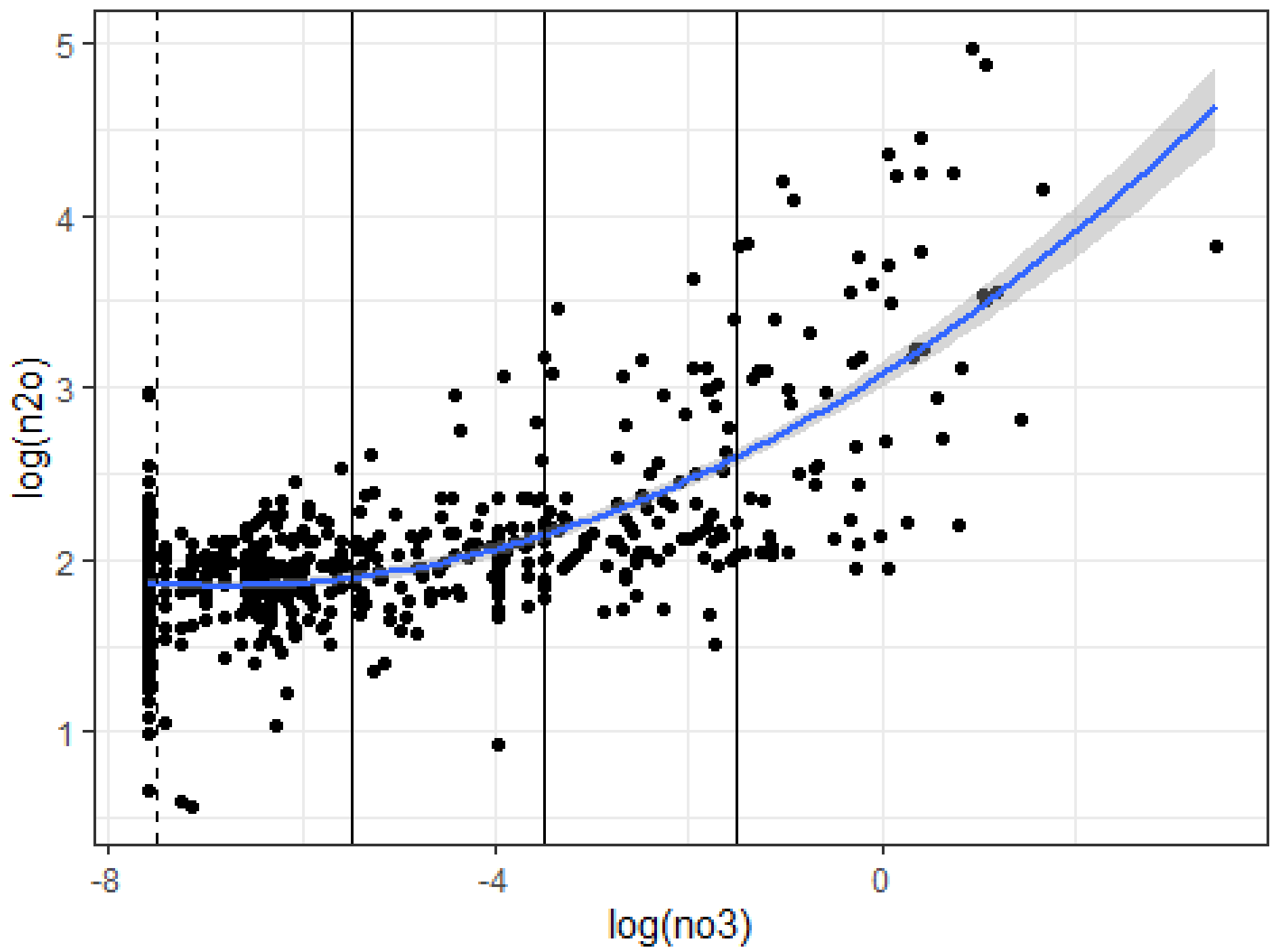

Below is a plot of N2O vs. the surface temperature observations.

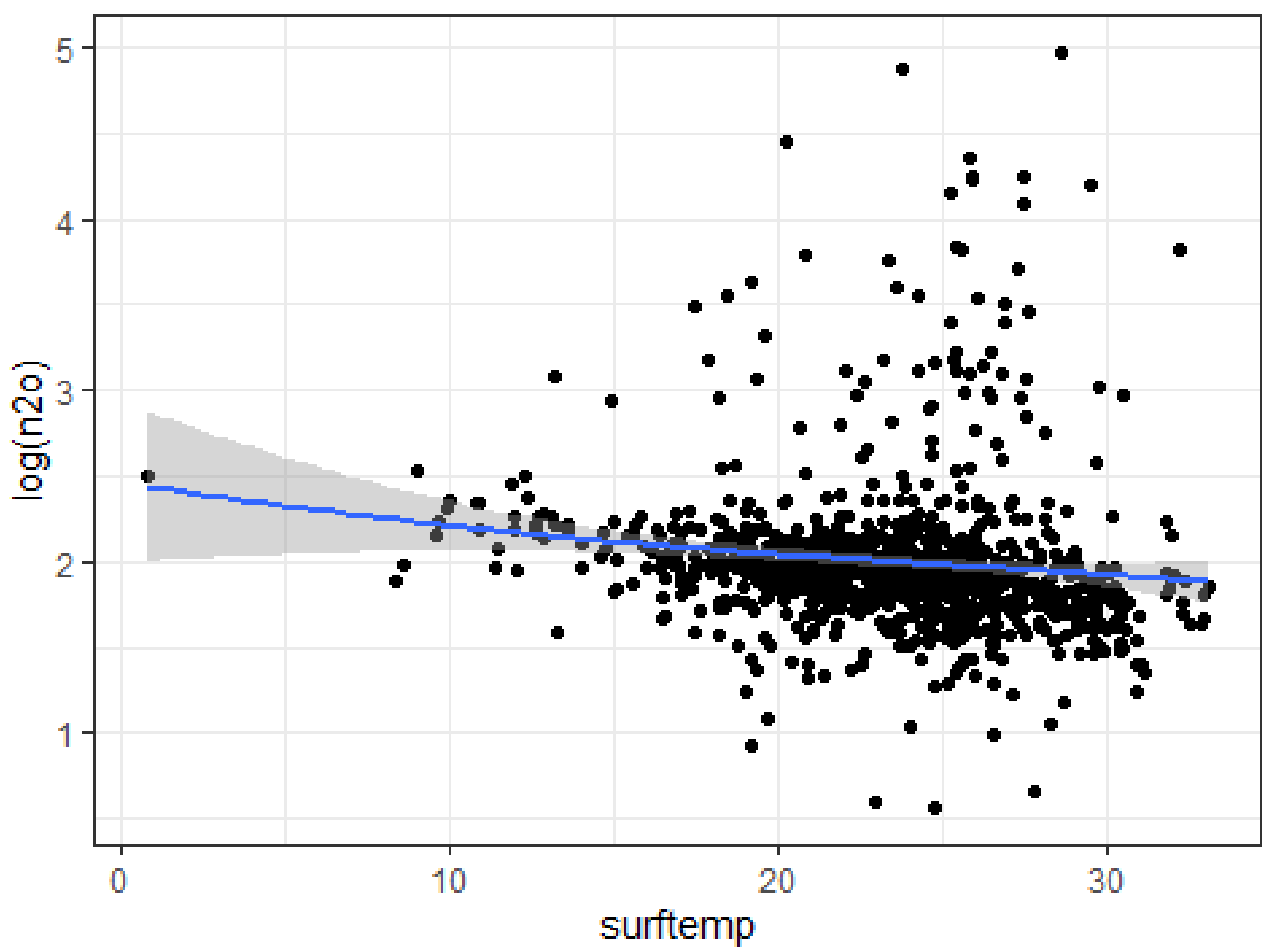

Below are plots of dissolved N2O vs. NO3 for 3 quantiles of the surface temperature measurements (quantiles increasing from 1 to 3). This plot suggested that the NO3 effect on N2O may have been stronger in lakes with higher observed temperatures.

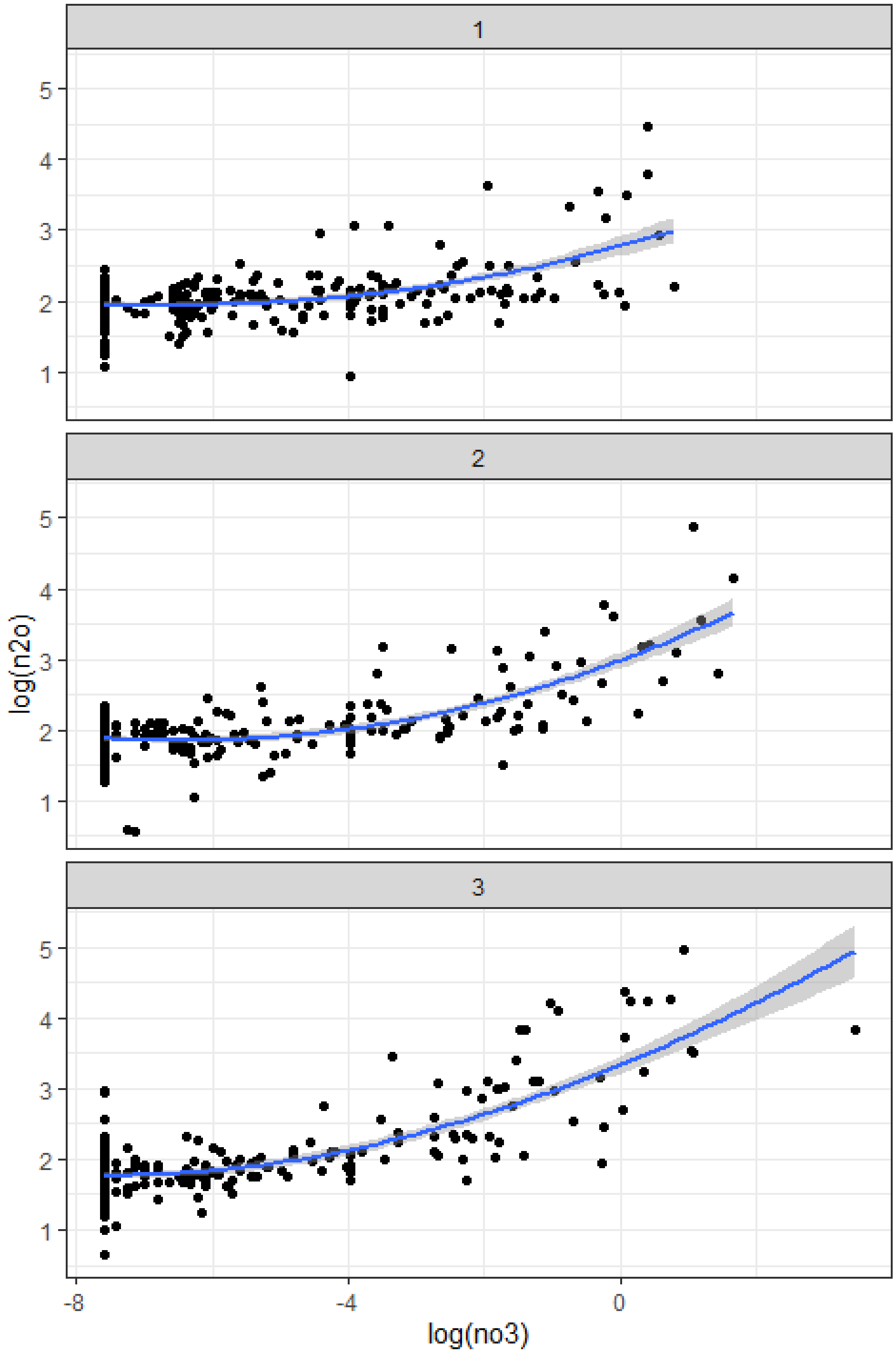

Below is a plot of N2O vs. the natural log of the surface area data for each lake from the NHD. The plot doesn't suggest a strong effect, but the pattern of variation suggests more variability in dissolved N2O in smaller lakes.

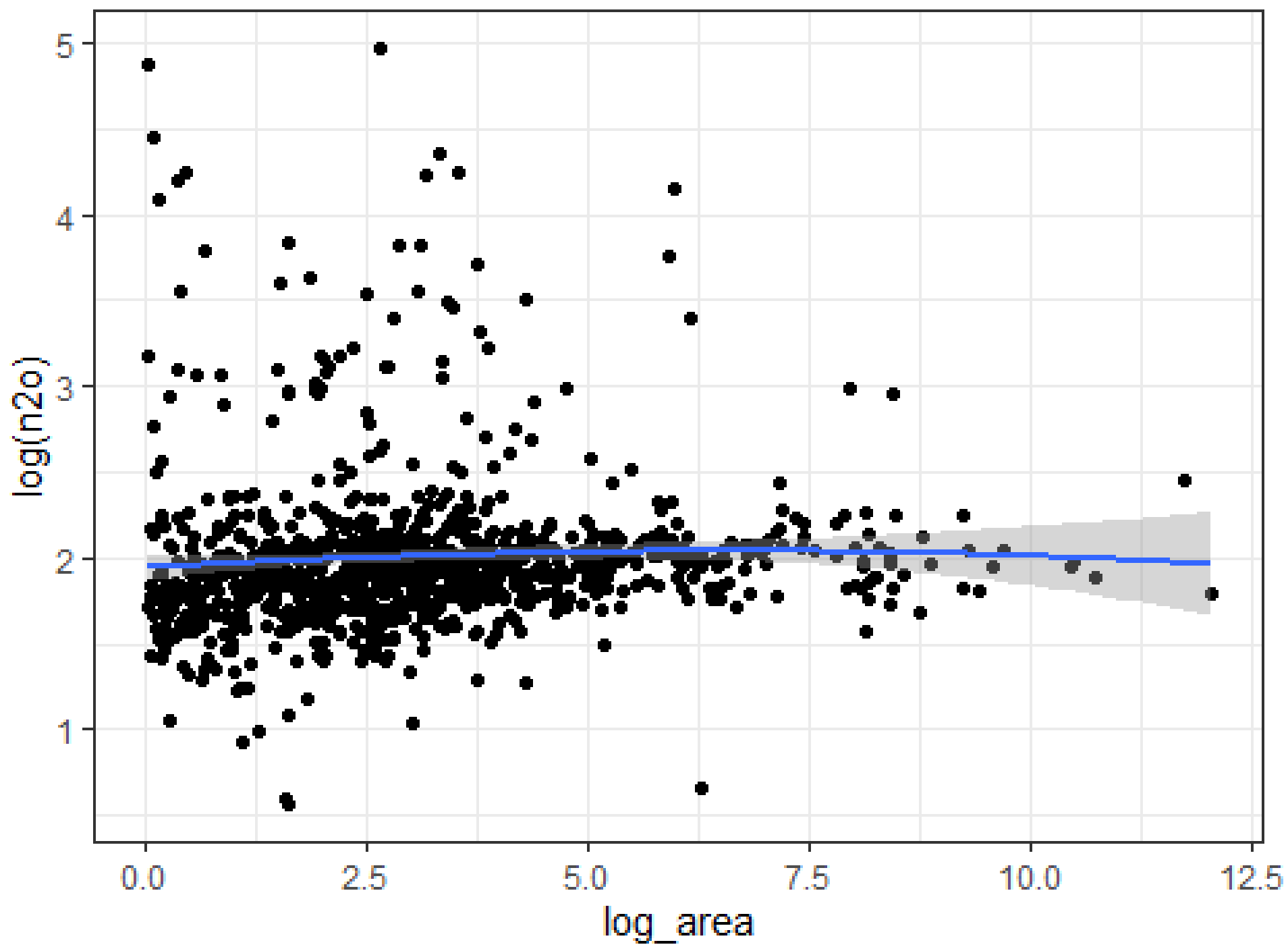

The next plot below shows the relationship between dissolved N2O and NO3 at 3 different quantiles (increasing 1 to 3) of the log-scaled lake surface area estimates. This plot perhaps suggests a weaker NO3 effect in the largest lakes.

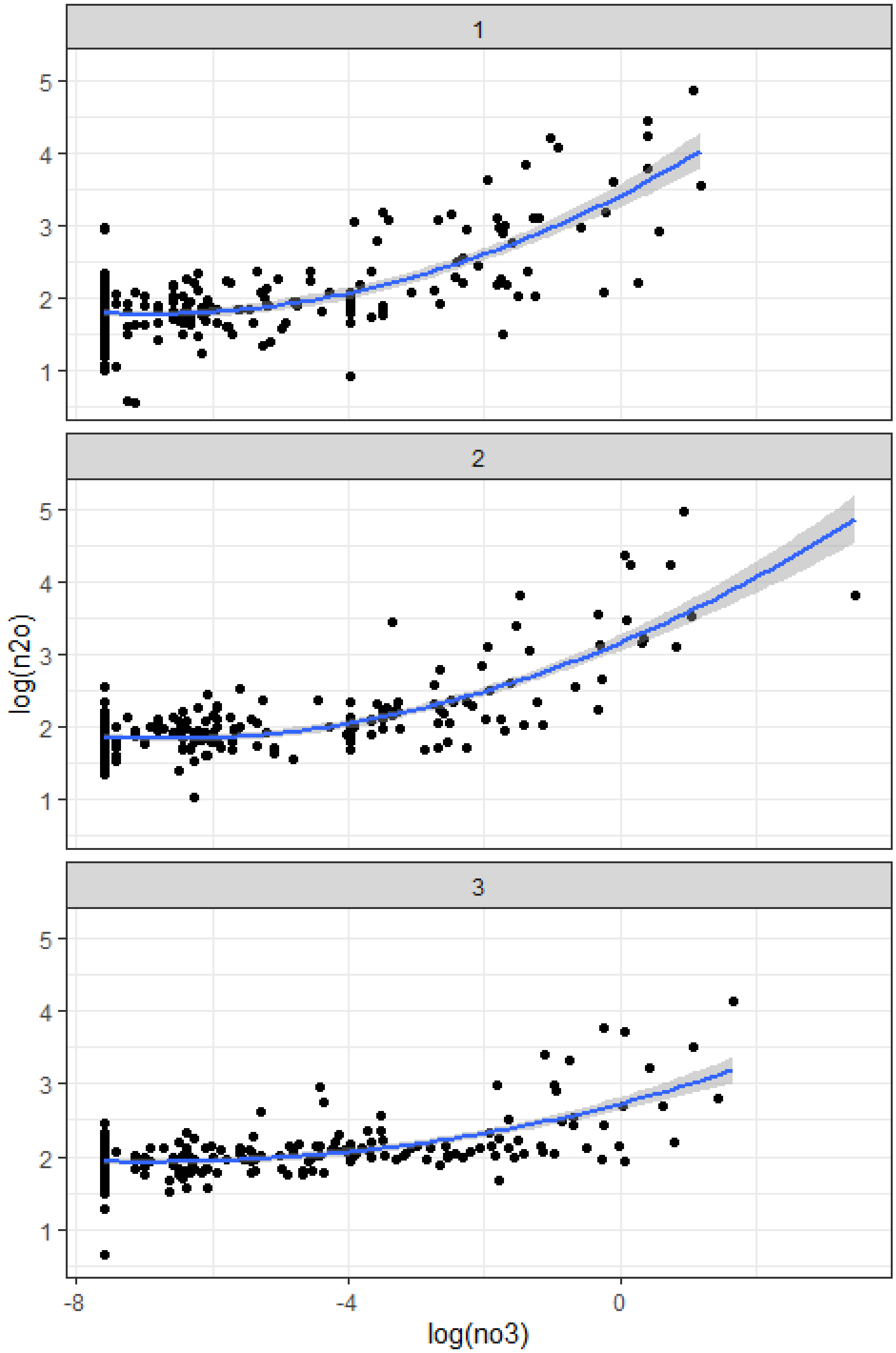

Below is a plot of N2O vs. the natural log of the dissolved oxygen measurements. Measurements for DO were missing from 4 samples and these observations were excluded from the plot below. The plot suggests a positive effect of DO, but the pattern may be dominated by extreme values at the upper and lower end of log(DO).

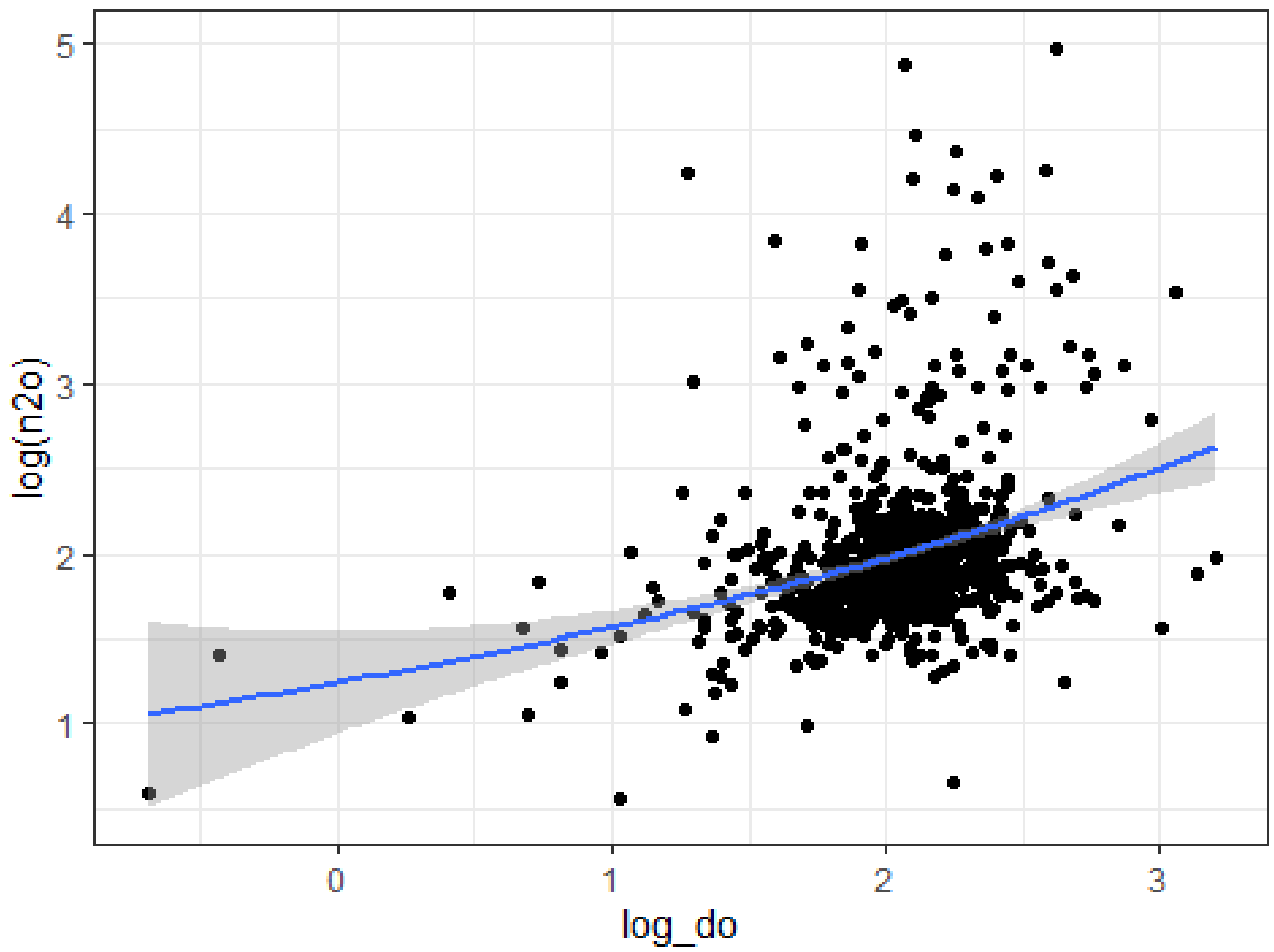

The next plot below shows the relationship between dissolved N2O and NO3 at 3 different quantiles (increasing 1 to 3) of the log-scaled dissolved oxygen measurements. Measurements for DO were missing from 4 samples and these observations were excluded from the plot below. This plot doesn't suggest a clear interaction of DO with NO3.

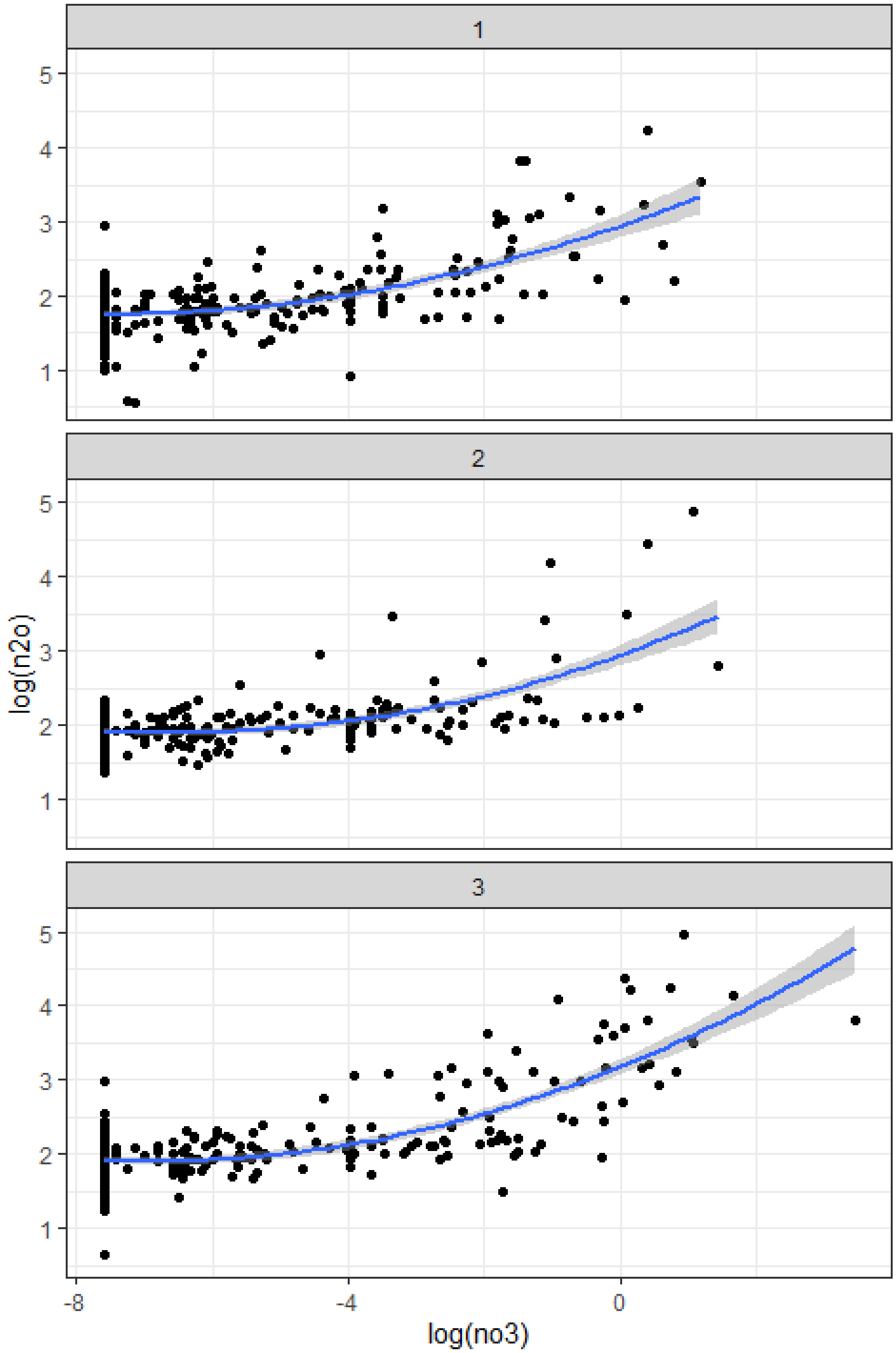

Below is a plot of N2O vs. the natural log of the chlorophyll a measurements. Measurements were missing from 4 samples and these observations were excluded from the plot. The plot suggests perhaps a slight negative effect of chl-a.

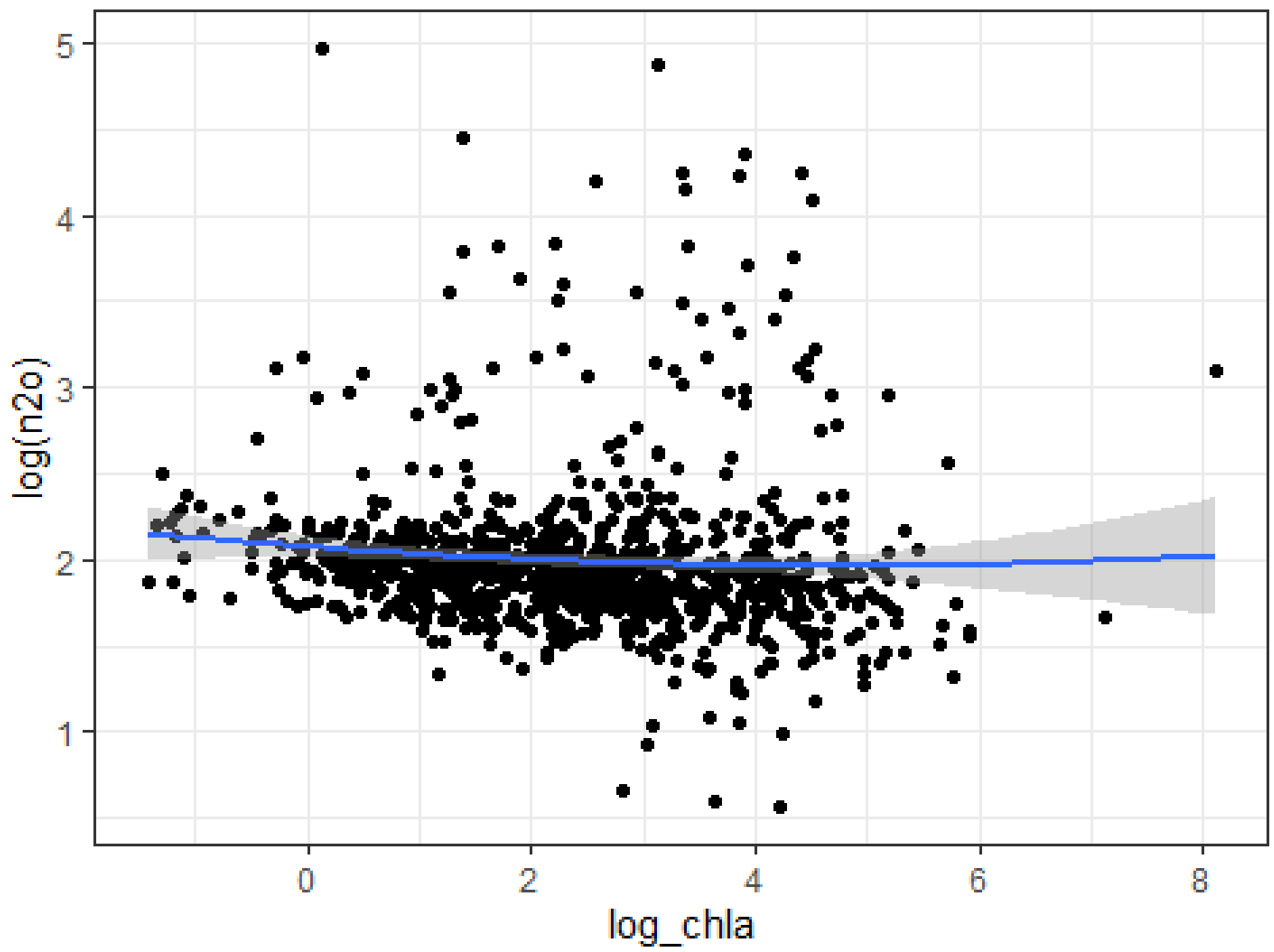

The next plot below shows the relationship between dissolved N2O and NO3 at 3 different quantiles (increasing 1 to 3) of the log-scaled chlorophyll a measurements. Measurements for chl-a were missing from 4 samples and these observations were excluded from the plot below. There is no clear interaction indicated in the plot below, but the NO3 effect may be stronger at high chl-a levels.

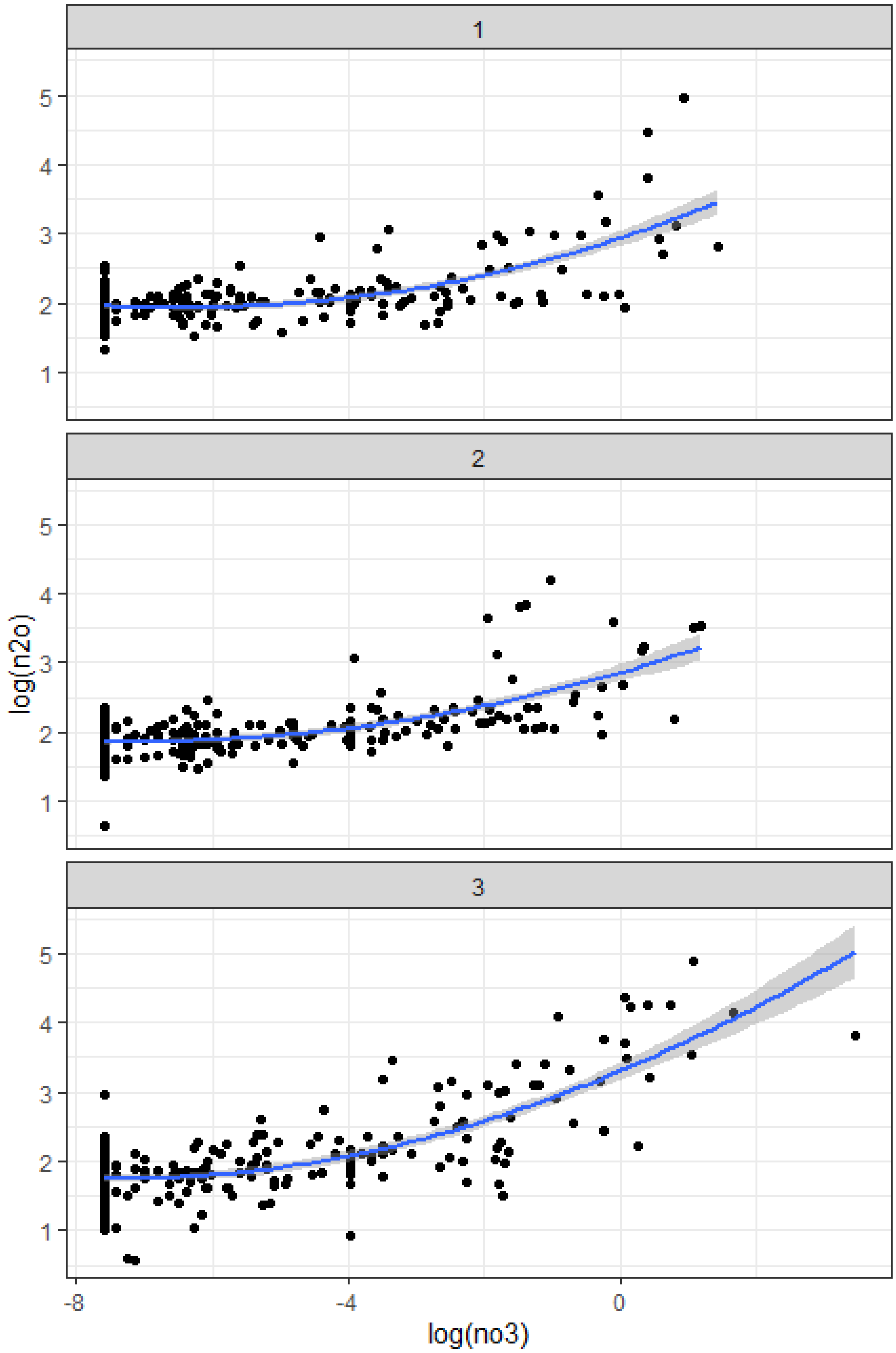

Below is a plot of N2O vs. a square root transformation of the bouyancy factor (BF) metric. Again, measurements were missing from 4 samples and these observations were excluded from the plot. The plot suggests perhaps a slight negative effect of the BF metric.

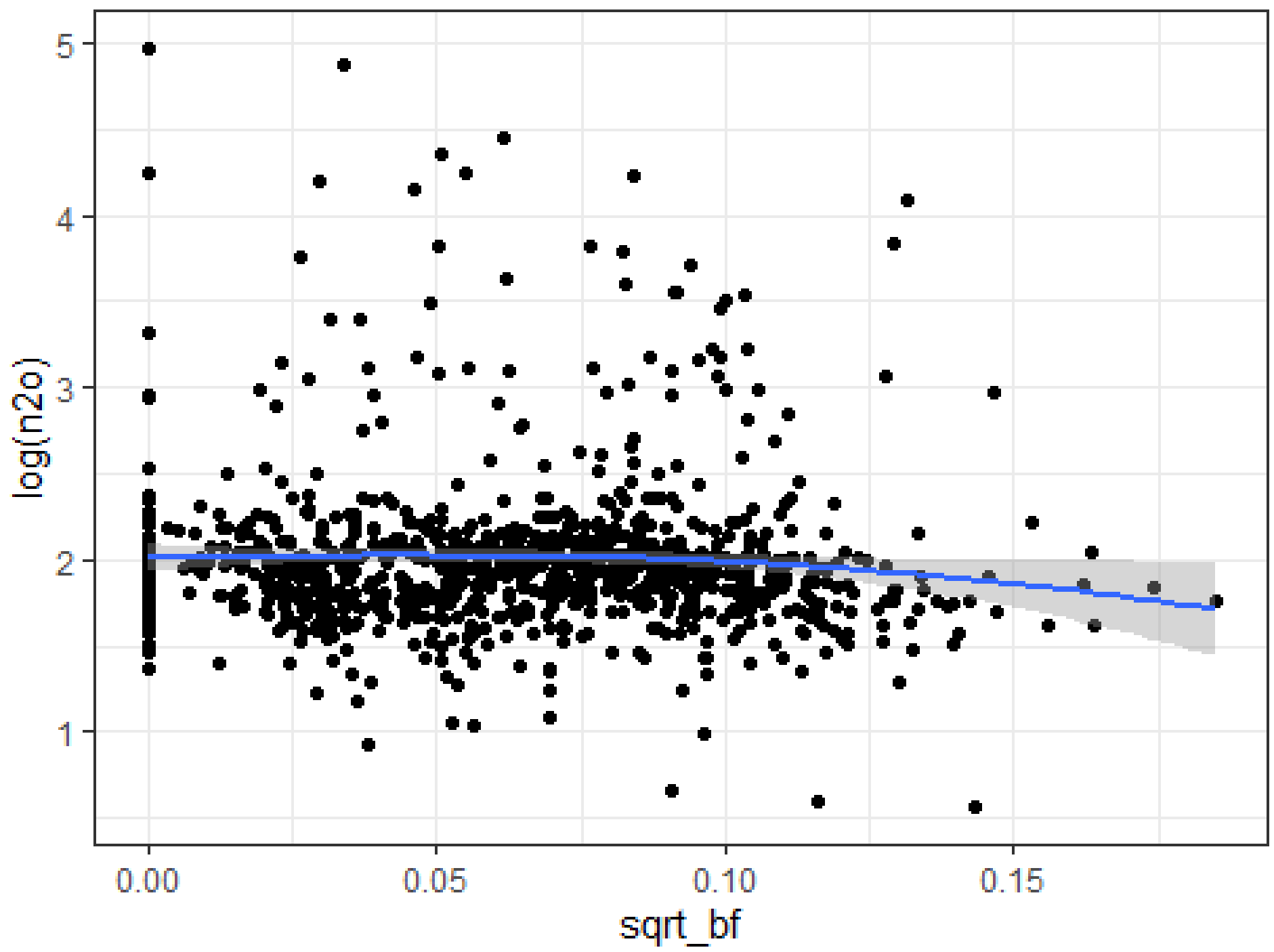

The next plot below shows the relationship between dissolved N2O and NO3 at 3 different quantiles (increasing 1 to 3) of the BF metric. There is no clear interaction indicated in the plot below.

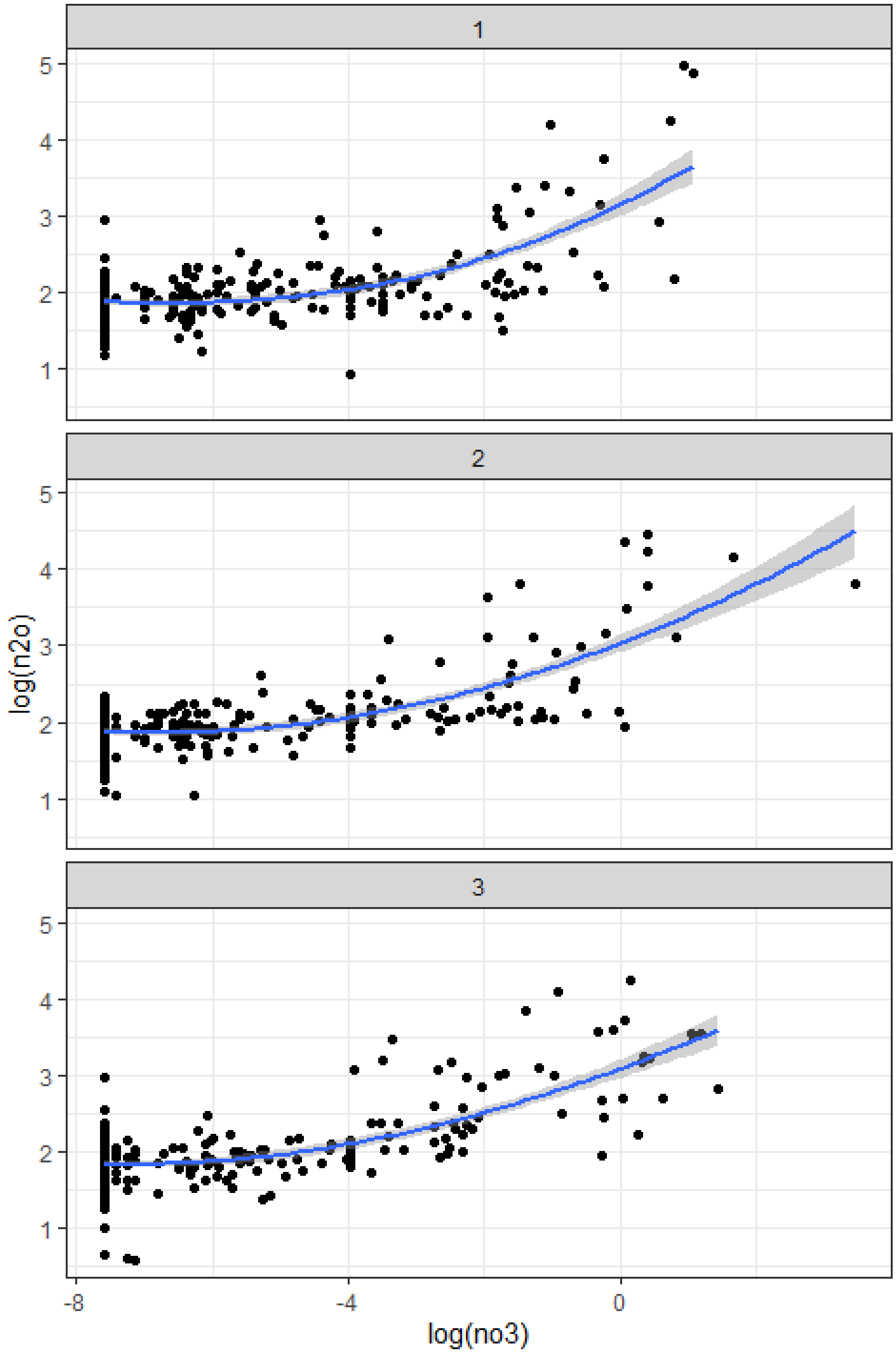

Similar plots are below, but with NO3 expressed as an ordered categorical variable with 5 levels. The positive and monotonic trends area similar to the previous plots where NO3 was treated as continuous. Note the large number of observations in the first NO3 category (no3_cat = 1). This category represented all of the observations for NO3 that were below the detection limit, which was most of the data.

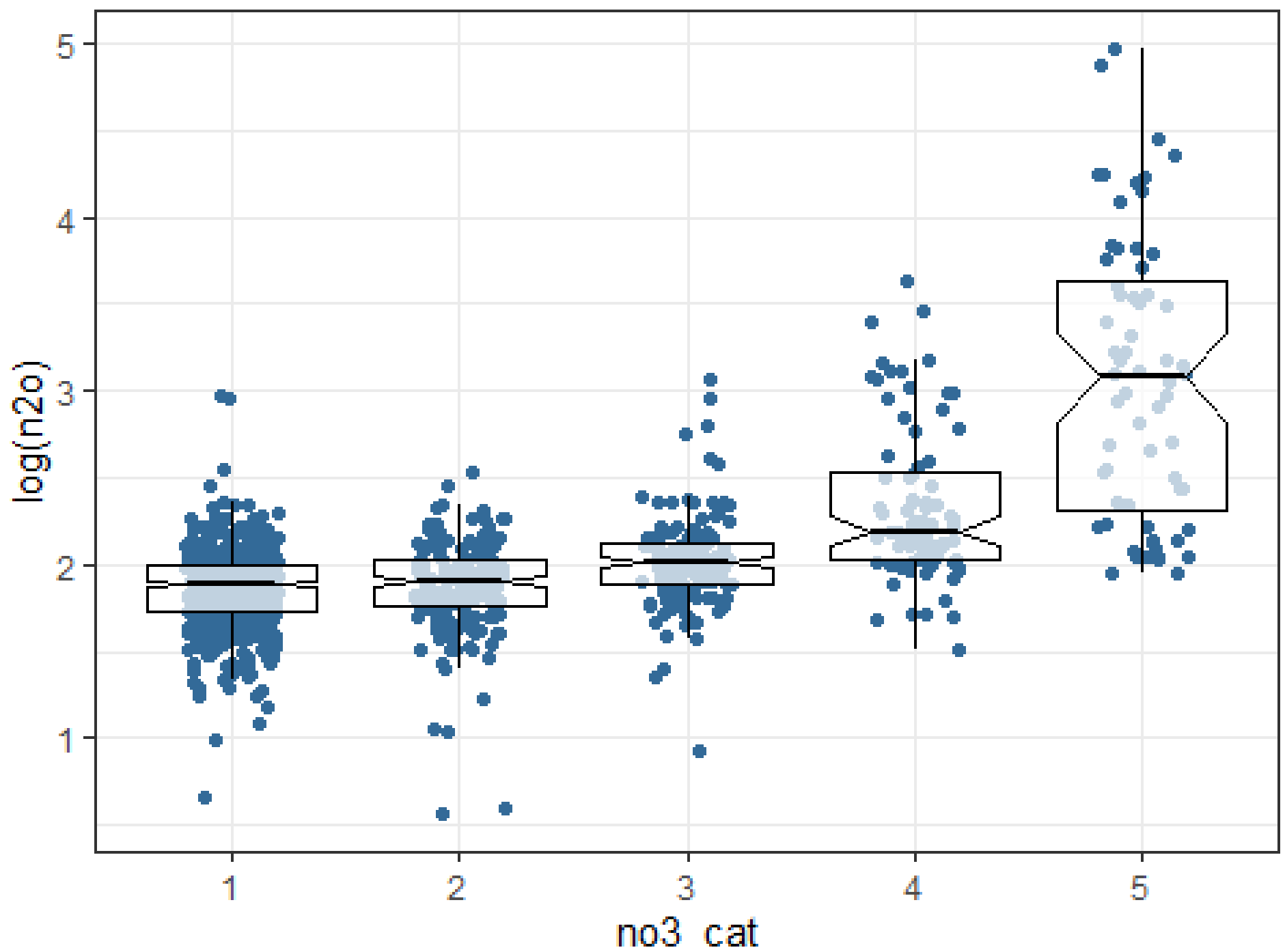

The plot below is N2O vs NO3 for three quantiles (increasing) of surface temperature.

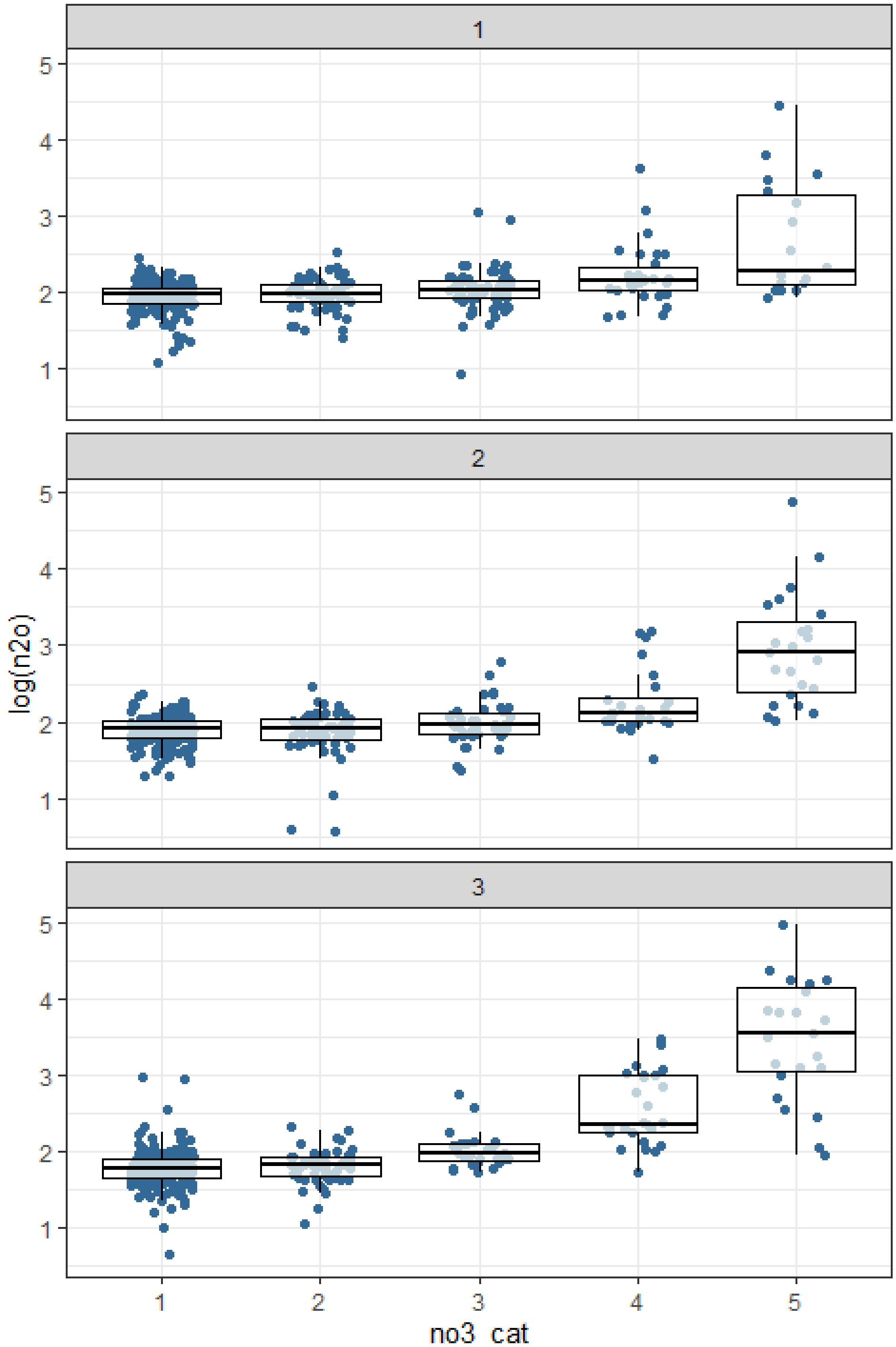

The plot below is N2O vs NO3 for three quantiles (increasing) of (log) surface area.

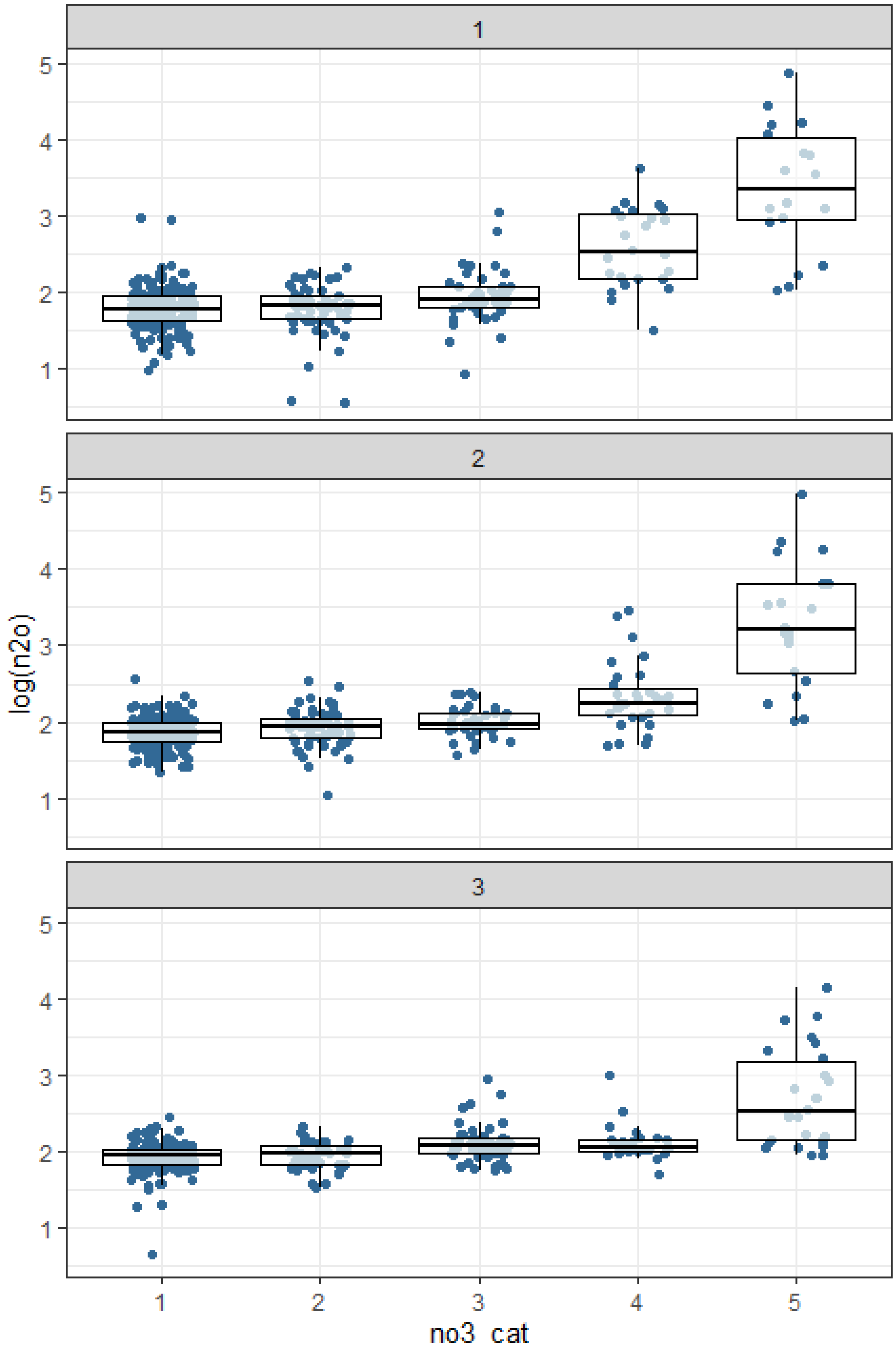

Below is a plot of log(dissolved N2O) vs. log(NO3) by ecoregion, which suggested that the NO3 effect on dissolved N2O may have varied by ecoregion.

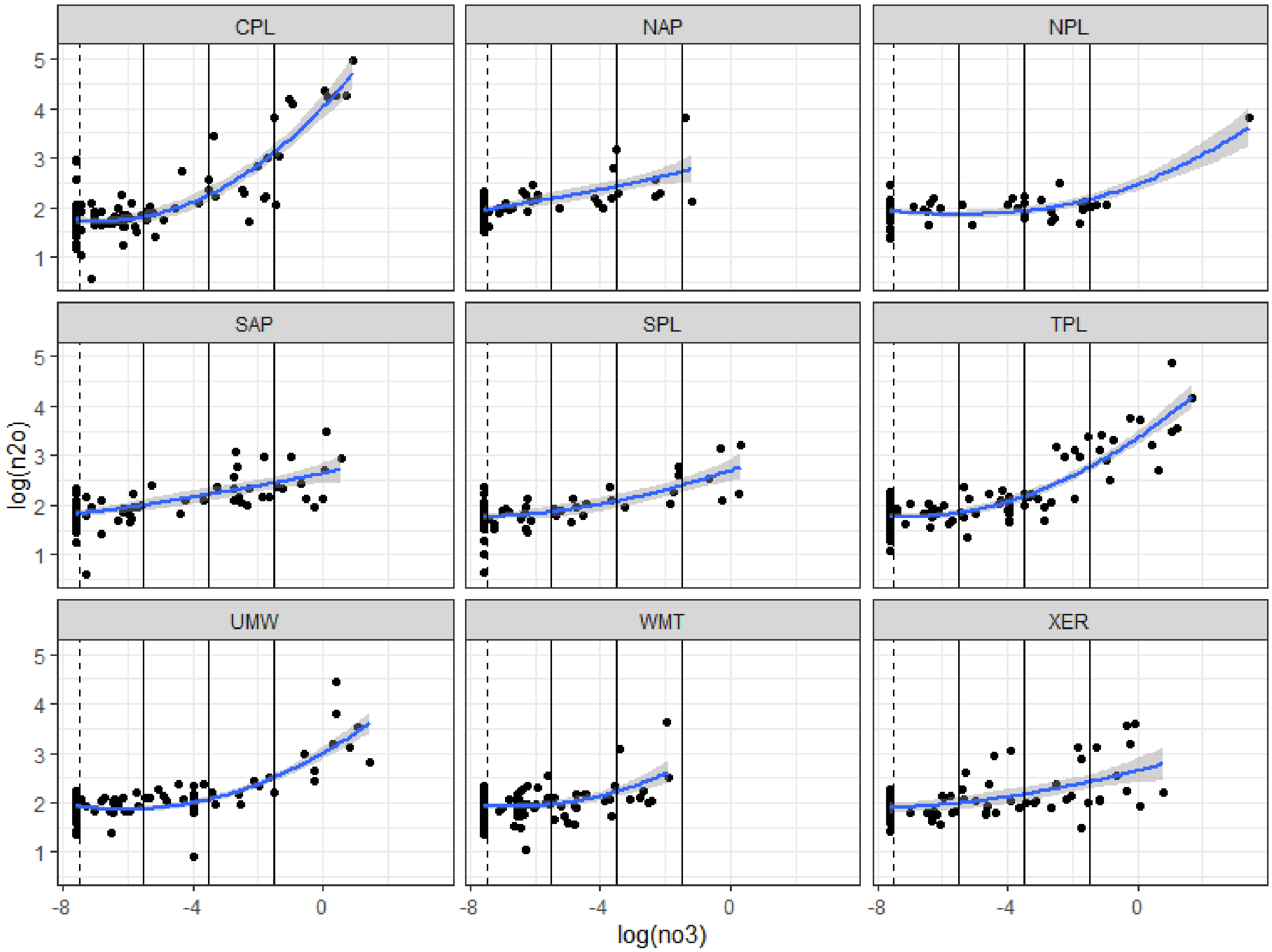

Below is the same plot as above but for the ordered categorical version of NO3.

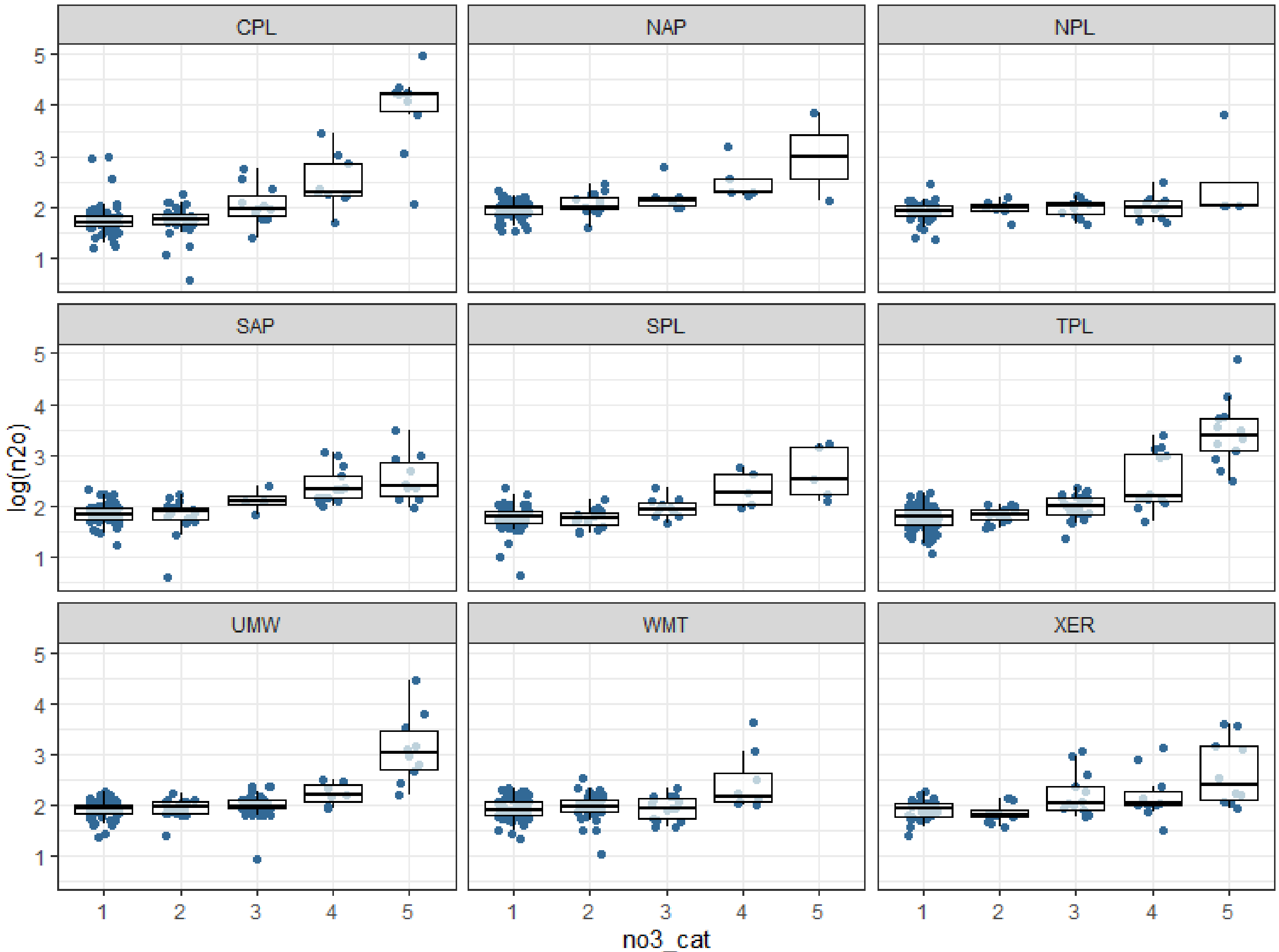

A plot below shows the relationships between dissolved N2O and log(NO3) by state, but within just the Temperate Plains (TPL) ecoregion. Within states, the number of observations were relatively small, but the trends were perhaps more linear.

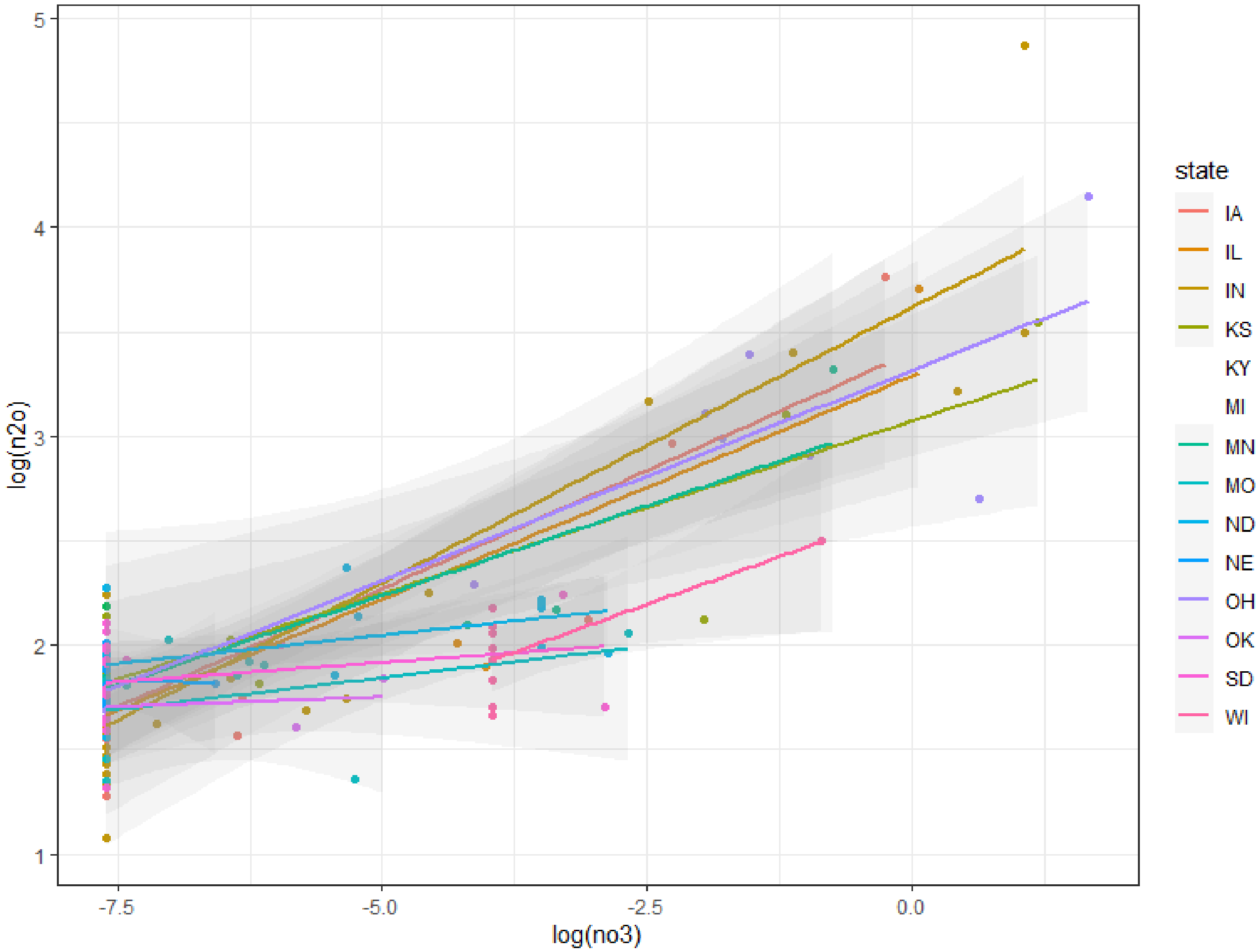

3 Model Fitting

3.1 Model 1

The first model for the two N2O responses, conditioned only on the study design factors.

```
bf_n2o <- bf(log(n2o) ~ 1 +
               (1 | a | WSA9) +
               (1 | b | WSA9:state) +
               (1 | c | WSA9:state:size_cat),
             family = gaussian())

bf_n2oeq <- bf(log(n2o_eq) ~ 1 +
               (1 | a | WSA9) +
               (1 | b | WSA9:state) +
               (1 | c | WSA9:state:size_cat),
             family = gaussian())

priors <- c(
```

```r
  prior(normal(2, 1), class = "Intercept", resp = "logn2o"), # centered near data mea
  prior(exponential(2), class = "sd", resp = "logn2o"),
  prior(exponential(2), class = "sigma", resp = "logn2o"),
  prior(normal(2, 1), class = "Intercept", resp = "logn2oeq"), # centered near data m
  prior(exponential(2), class = "sd", resp = "logn2oeq"),
  prior(exponential(2), class = "sigma", resp = "logn2oeq"),
  prior(lkj(2), class = "rescor"),
  prior(lkj(2), class = "cor")
  )

n2o_mod1 <- brm(bf_n2o + bf_n2oeq + set_rescor(rescor = TRUE),
                data = df_model,
                prior = priors,
                control = list(adapt_delta = 0.99, max_treedepth = 14),
                #sample_prior = "only",
                save_pars = save_pars(all = TRUE),
                seed = 145,
                chains=4,
                iter=5000,
                cores=4)

save(n2o_mod1, file = "C:/Users/rmartin/OneDrive - Environmental Protection Agency (E
```

3.1.1 Summarize fit

The summaries of the estimated parameters and key HMC convergence diagnostics for the fitted model are printed below. There were no apparent issues with the HMC sampling. All $\hat{R}$ values were less than 1.01 and effective sample size (ESS) calculations suggested that the posterior contained a sufficient number of effective samples for conducting inference.

```
##  Family: MV(gaussian, gaussian)
##   Links: mu = identity; sigma = identity
##          mu = identity; sigma = identity
## Formula: log(n2o) ~ 1 + (1 | a | WSA9) + (1 | b | WSA9:state) + (1 | c | WSA9:stat
##          log(n2o_eq) ~ 1 + (1 | a | WSA9) + (1 | b | WSA9:state) + (1 | c | WSA9:s
##    Data: df_model (Number of observations: 984)
##   Draws: 4 chains, each with iter = 5000; warmup = 2500; thin = 1;
##          total post-warmup draws = 10000
##
## Priors:
## Intercept_logn2o ~ normal(2, 1)
## Intercept_logn2oeq ~ normal(2, 1)
## L ~ lkj_corr_cholesky(2)
## Lrescor ~ lkj_corr_cholesky(2)
## <lower=0> sd_logn2o ~ exponential(2)
## <lower=0> sd_logn2oeq ~ exponential(2)
```

```
## <lower=0> sigma_logn2o ~ exponential(2)
## <lower=0> sigma_logn2oeq ~ exponential(2)
##
## Multilevel Hyperparameters:
## ~WSA9 (Number of levels: 9)
##                                      Estimate Est.Error l-95% CI u-95% CI
## sd(logn2o_Intercept)                     0.04      0.03     0.00     0.13
## sd(logn2oeq_Intercept)                   0.06      0.02     0.03     0.11
## cor(logn2o_Intercept,logn2oeq_Intercept)     0.05      0.44    -0.80     0.83
##                                      Rhat Bulk_ESS Tail_ESS
## sd(logn2o_Intercept)                 1.00     2475     4230
## sd(logn2oeq_Intercept)               1.00     3326     5332
## cor(logn2o_Intercept,logn2oeq_Intercept) 1.01     1141     3129
##
## ~WSA9:state (Number of levels: 96)
##                                      Estimate Est.Error l-95% CI u-95% CI
## sd(logn2o_Intercept)                     0.26      0.03     0.21     0.32
## sd(logn2oeq_Intercept)                   0.04      0.01     0.03     0.05
## cor(logn2o_Intercept,logn2oeq_Intercept)     0.24      0.15    -0.07     0.52
##                                      Rhat Bulk_ESS Tail_ESS
## sd(logn2o_Intercept)                 1.00     3199     4775
## sd(logn2oeq_Intercept)               1.00     3549     5476
## cor(logn2o_Intercept,logn2oeq_Intercept) 1.00     3332     5553
##
## ~WSA9:state:size_cat (Number of levels: 352)
##                                      Estimate Est.Error l-95% CI u-95% CI
## sd(logn2o_Intercept)                     0.09      0.04     0.01     0.16
## sd(logn2oeq_Intercept)                   0.02      0.01     0.00     0.03
## cor(logn2o_Intercept,logn2oeq_Intercept)    -0.22      0.37    -0.85     0.58
##                                      Rhat Bulk_ESS Tail_ESS
## sd(logn2o_Intercept)                 1.00      863     1397
## sd(logn2oeq_Intercept)               1.00      928     1365
## cor(logn2o_Intercept,logn2oeq_Intercept) 1.00     1661     2807
##
## Regression Coefficients:
##                    Estimate Est.Error l-95% CI u-95% CI Rhat Bulk_ESS Tail_ESS
## logn2o_Intercept       2.02      0.04     1.95     2.09 1.00     2641     3963
## logn2oeq_Intercept     2.00      0.02     1.96     2.04 1.00     3446     4550
##
## Further Distributional Parameters:
##                Estimate Est.Error l-95% CI u-95% CI Rhat Bulk_ESS Tail_ESS
## sigma_logn2o       0.40      0.01     0.37     0.42 1.00     2749     5523
## sigma_logn2oeq     0.08      0.00     0.08     0.09 1.00     4790     6698
##
## Residual Correlations:
##                          Estimate Est.Error l-95% CI u-95% CI Rhat Bulk_ESS
## rescor(logn2o,logn2oeq)     0.21      0.04     0.14     0.27 1.00     6172
##                          Tail_ESS
## rescor(logn2o,logn2oeq)     7243
```

```
##
## Draws were sampled using sampling(NUTS). For each parameter, Bulk_ESS
## and Tail_ESS are effective sample size measures, and Rhat is the potential
## scale reduction factor on split chains (at convergence, Rhat = 1).
```

In the summary above, the estimated standard deviations for the varying group effects on the mean behavior of the dissolved N2O response suggested fairly low, but non-zero variability across each of the three levels. The standard deviations estimated for the same varying effects for equilibrium N2O were also relatively small. Finally, note the relatively small, but positive residual correlation between the two N2O responses.

Before investing too much into inferences from this model, however, the model fit was evaluated below using a series of graphical posterior predictive checks (PPC, Gelman et al. 2014; Gelman, Hill, and Vehtari 2020, Ch. 11).

3.1.2 Model checks

3.1.2.1 Dissolved N2O

Below are a series of panels illustrating graphical PPCs for the dissolved N2O component of the multivariate model. The top left panel compares a density plot of the observed data (black line) to density estimates drawn for 200 samples from the posterior predictive distribution (PPD; blue lines) of the fitted model. The top right panel compares the cumulative density distributions in the same manner. The left middle panel compares means *vs.* standard deviations for 1000 draws from the PPD (blue dots) to the sample mean *vs.* standard deviation (black dot). The right middle panel compares skewness *vs.* kurtosis for 1000 draws from the PPD to the skewness *vs.* kurtosis estimates for the observed data. The bottom left panel compares max *vs.* min values for 1000 draws from the PPD to the max *vs.* min values of the sample data. Finally, the bottom right panel shows the observed *vs.* average predicted values for each observation in the sample. The average predicted values were calculated as the mean prediction for each observation in the PPD based on 1000 draws.

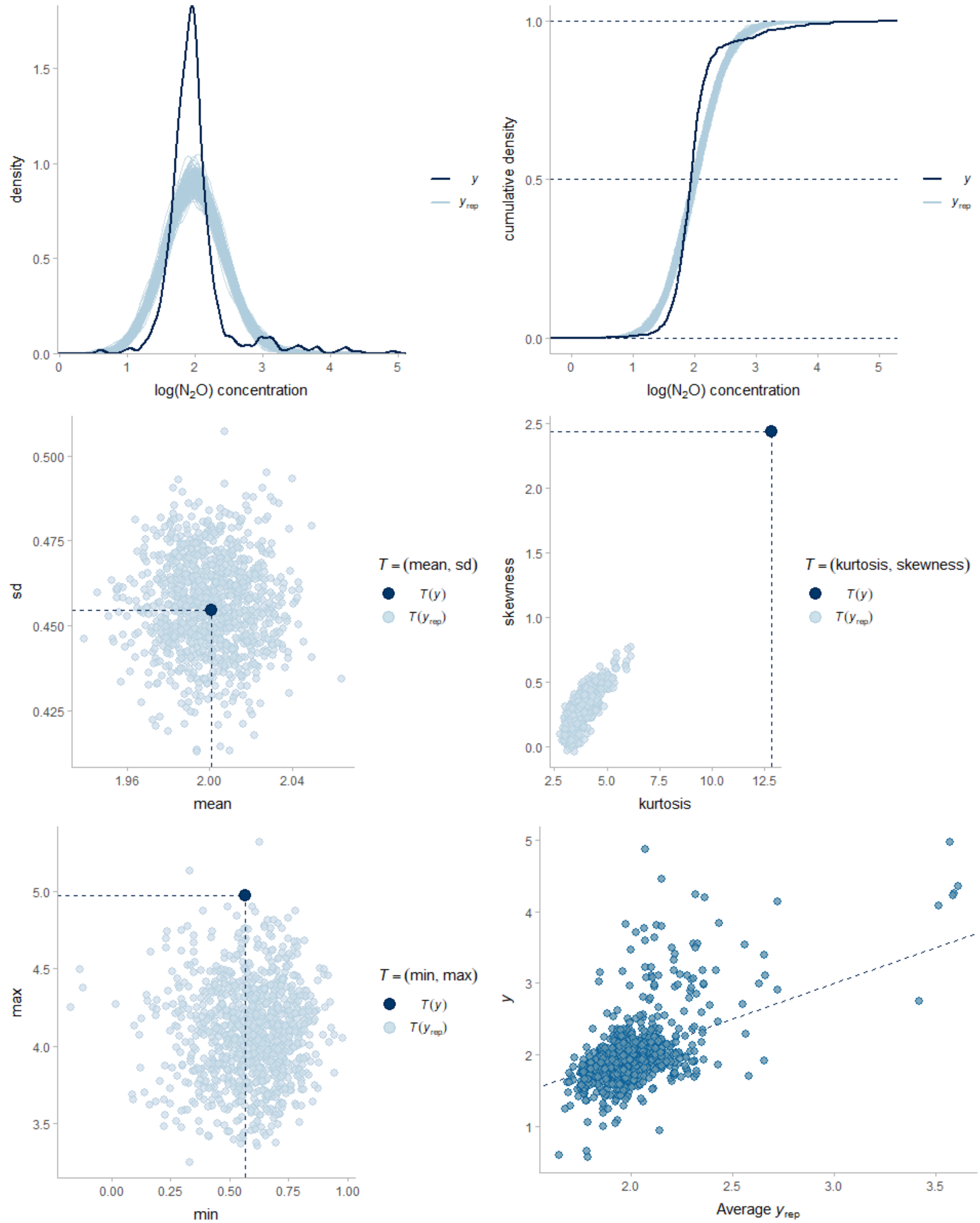

The general takeaway from the PPCs above was that the model replicated the central tendency of the observed data fairly well, but failed to sufficiently replicate other important aspects of the distribution, such as skewness and kurtosis. The observed *vs.* average predictions scatterplot also suggested substantial heteroscedasticity in the errors.

The same checks were run below, but for the test set of 95 held-out, second-visit data points. The patterns in misfit were similar to the patterns indicated in the PPCs with the training data above.

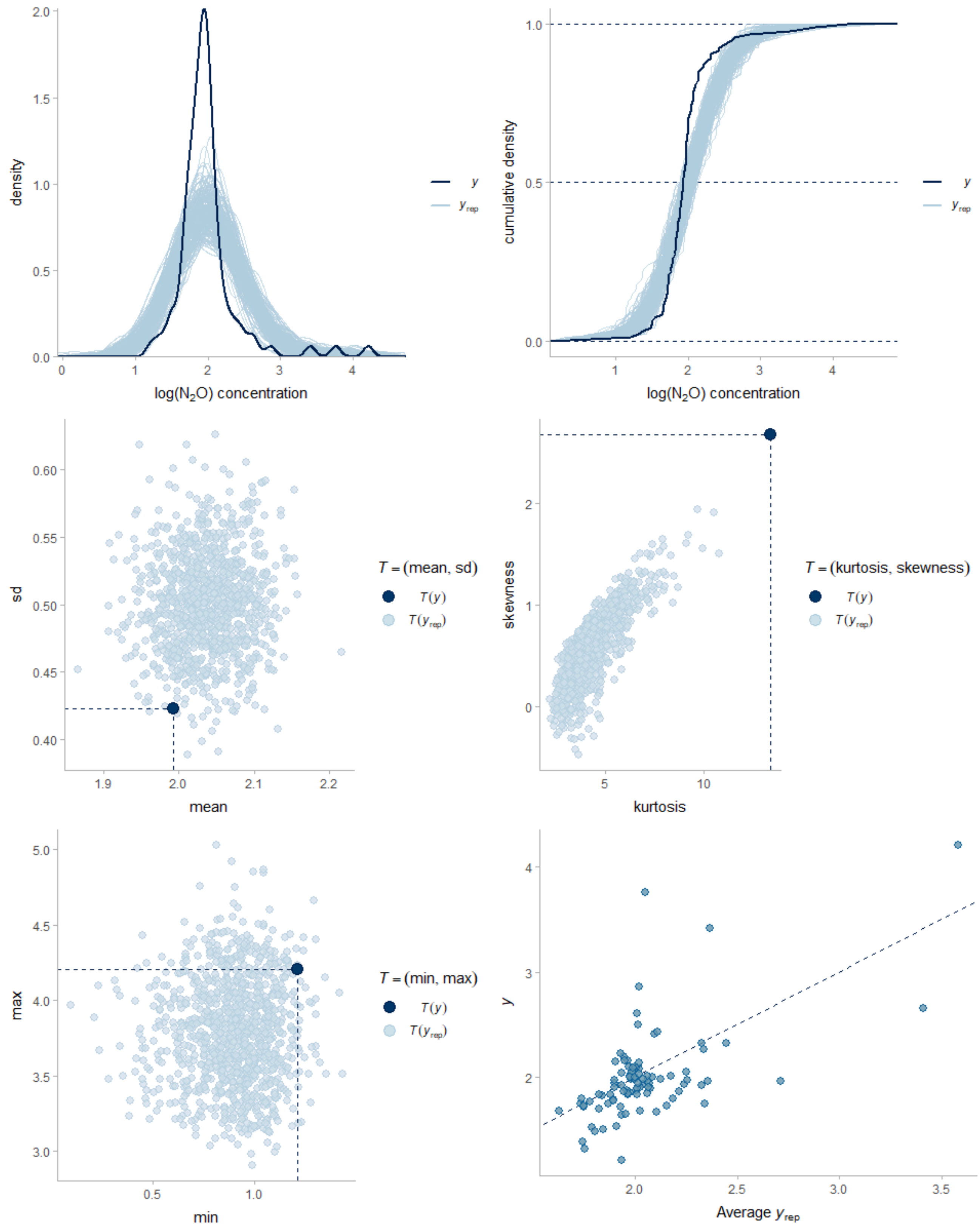

3.1.2.2 Equilibrium N2O

Below are PPCs for the equilibrium N2O component of the model. As with the dissolved N2O PPCs, the model seemed to do an OK job at replicating the central tendency, but performed less well at replicating other important aspects of the overall distribution.

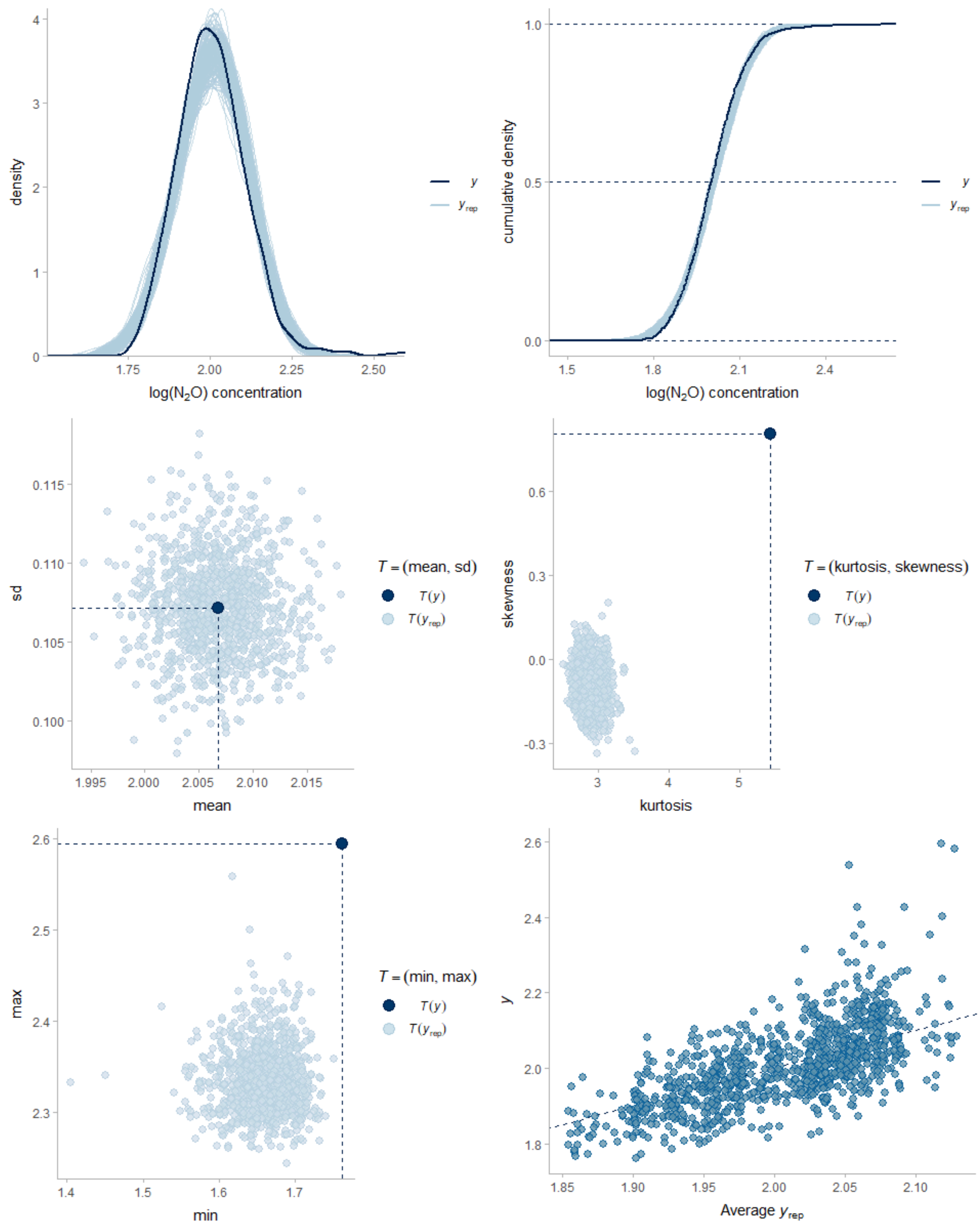

Below are the same PPCs for equilibrium N2O in the re-visit sites.

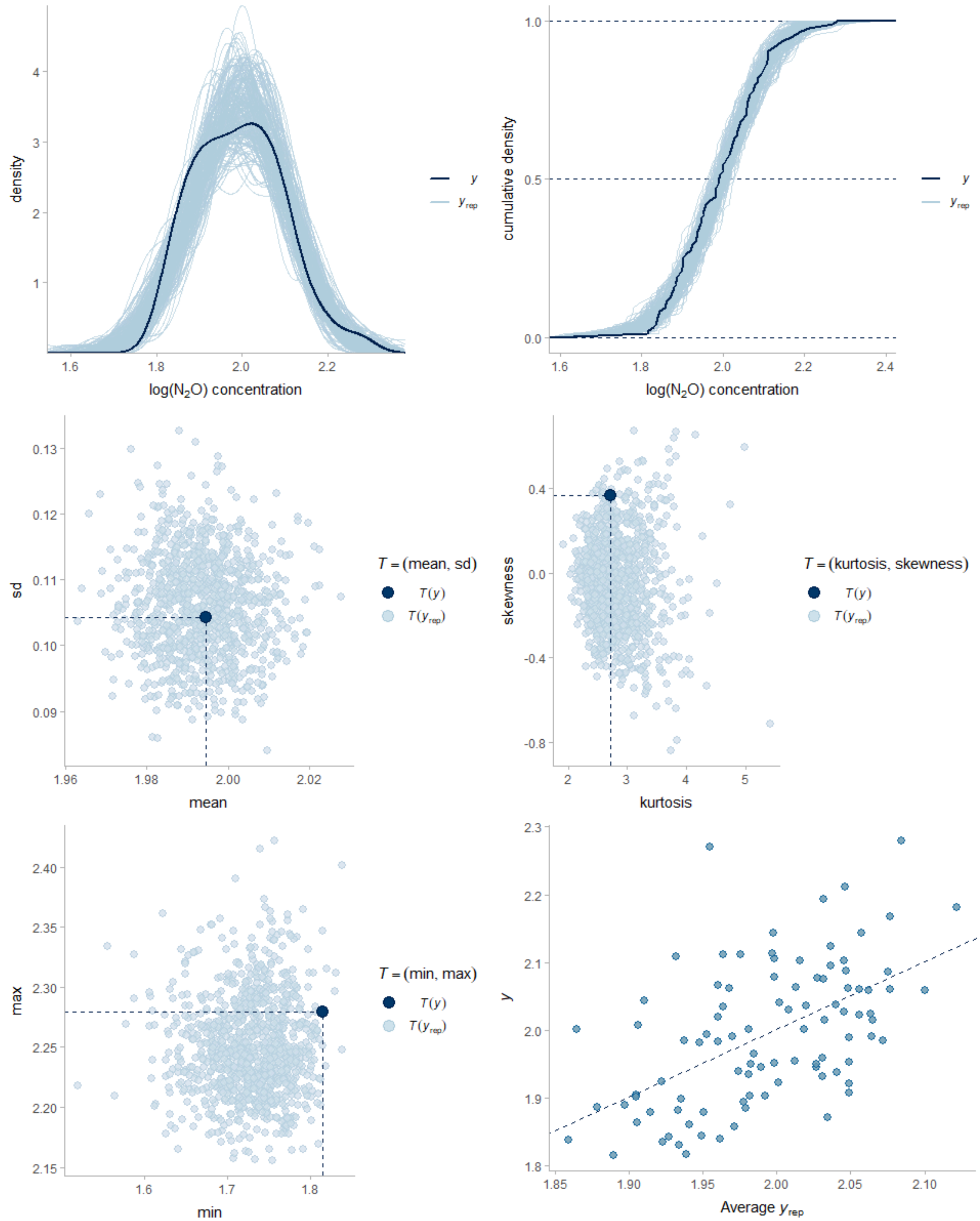

3.1.2.3 Bivariate

The graphical check below compared bivariate density contours estimated from the observed data (black lines) to density contours estimated for each of 20 draws from the PPD. The model appeared to do a good job of replicating the bivariate mean, but was poor at representing the overall joint distribution.

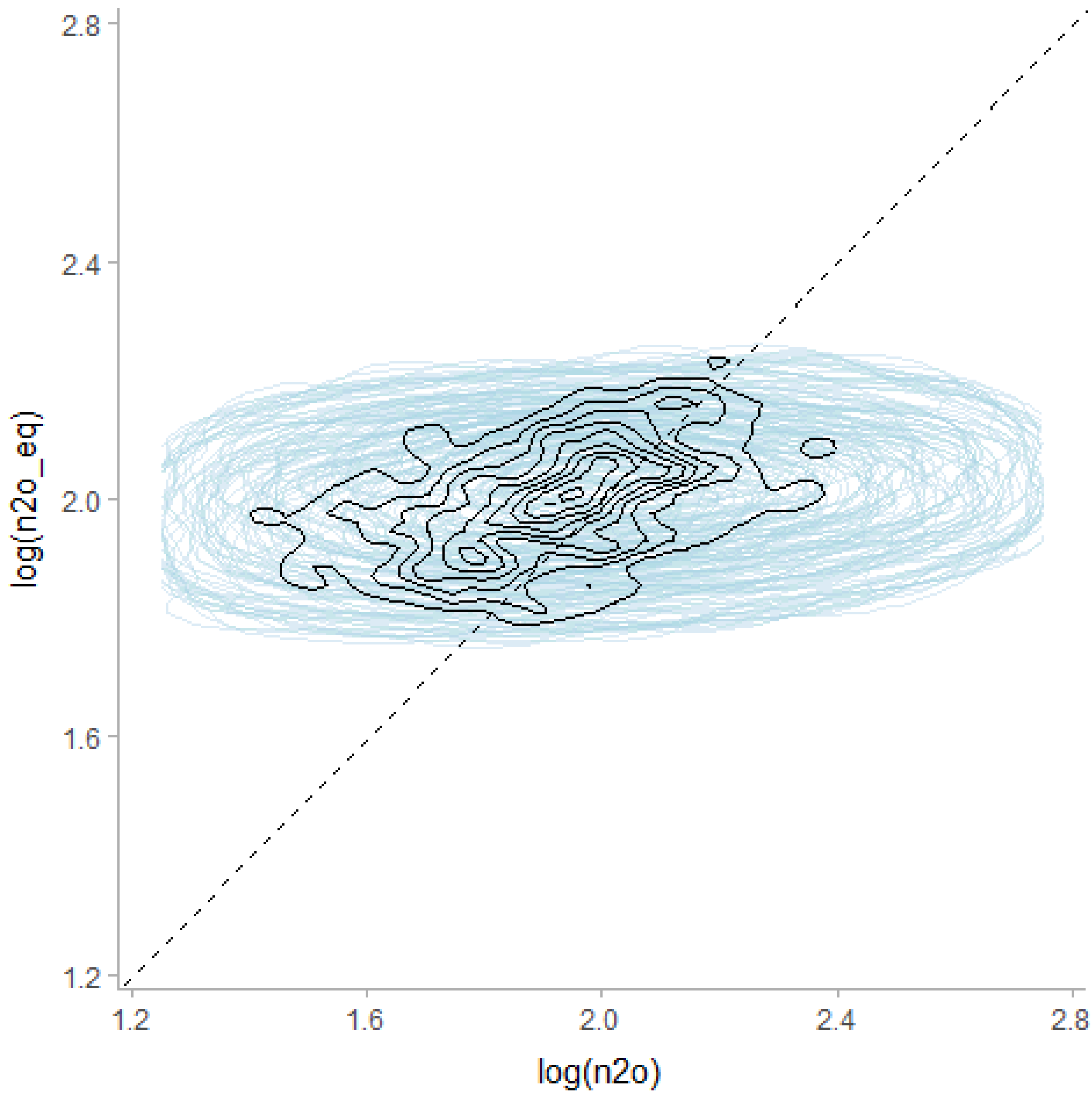

The same bivariate check is shown below for the re-visit data.

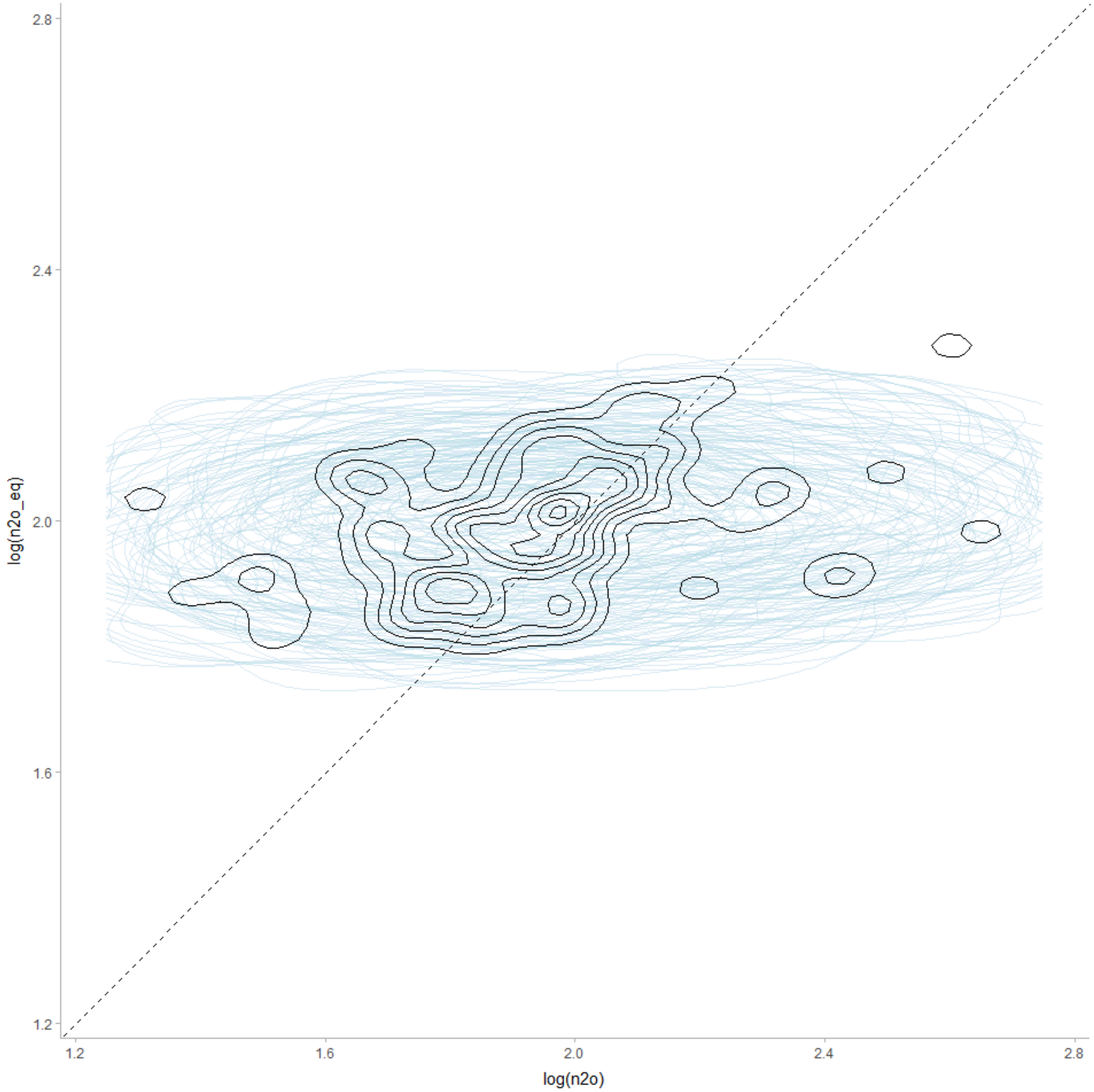

3.1.2.4 Saturation

The graphical PPCs below were aimed at evaluating how well the multivariate model did at representing the observed saturation ratio:

$$\frac{N_2O_{diss}}{N_2O_{equi}}$$

This quantity was estimated as a derived variable by dividing the dissolved N2O PPD by the equilibrium N2O PPD. The proportion of under-saturated lakes in the sample was estimated by summing the number of lakes from each posterior predictive draw wherein the ratio was < 1 and dividing that number by the total number of lakes in the sample, which was 984. Overall, these checks indicated that properly representing the tails of the dissolved N2O and

N2O-eq observations would likely be necessary in order to better replicate the observed saturation metrics. The observed proportion of under-saturated lakes was underestimated by more than 10 percentage points, on average.

The top left panel, below, is a density plot of the observed saturation ratio (black line) compared to an estimate using 50 draws from the derived PPD (blue lines). The top right panel shows the observed proportion of under-saturated lakes compared to a model estimate based on 1000 draws from the PPD. The left middle panel shows the mean *vs.* standard deviation of the saturation ratio for the observed data compared to the same estimates for 500 draws from the PPD. The right middle panel shows the max *vs.* min for the sample compared to 500 draws from the PPD. Finally, the bottom left panel shows the observed *vs.* average predicted saturation ratio for all 984 lakes sampled in the dataset.

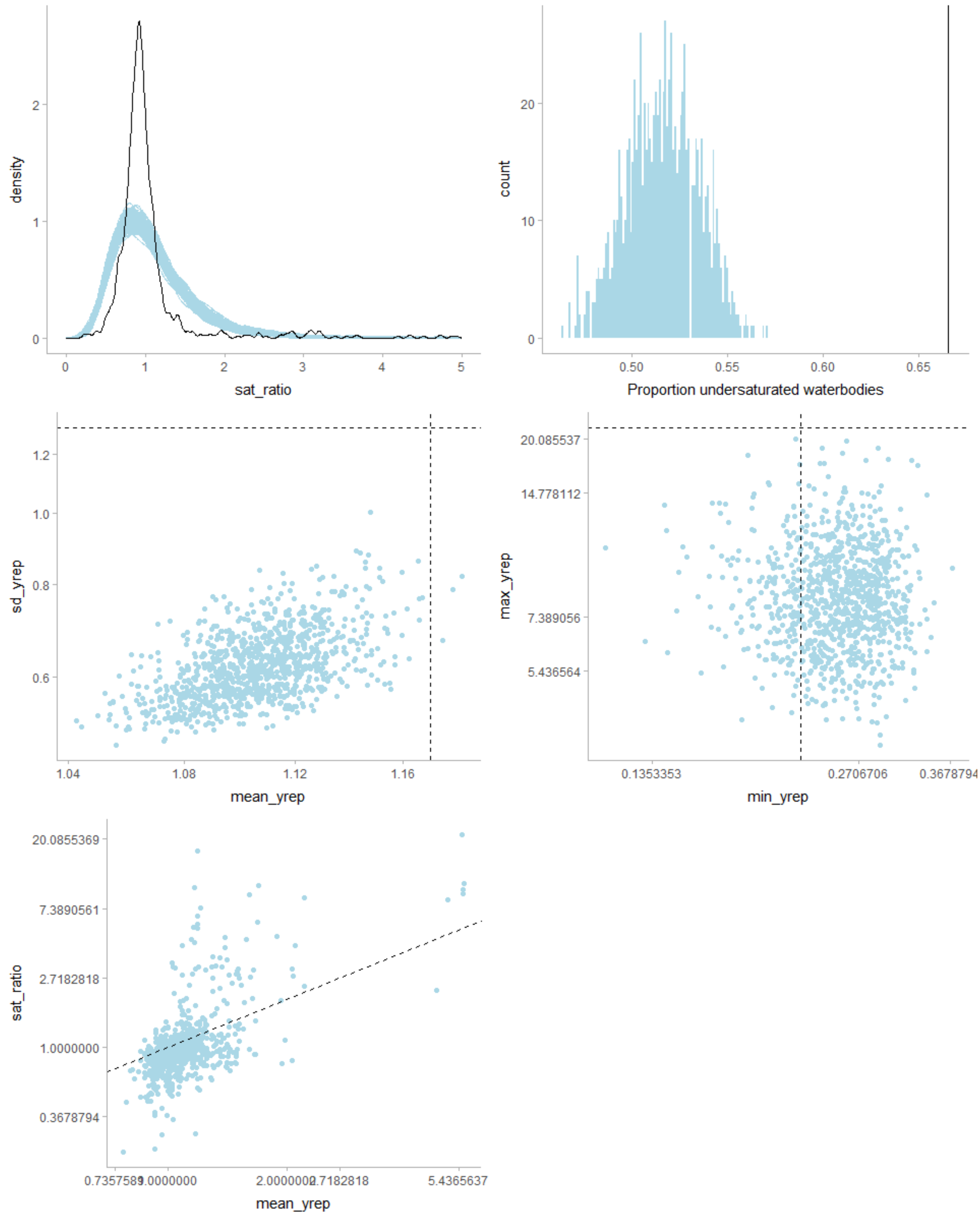

The same PPCs are shown below for the revisit data. These checks indicated that the model did a similarly underwhelming job of replicating the re-visit data.

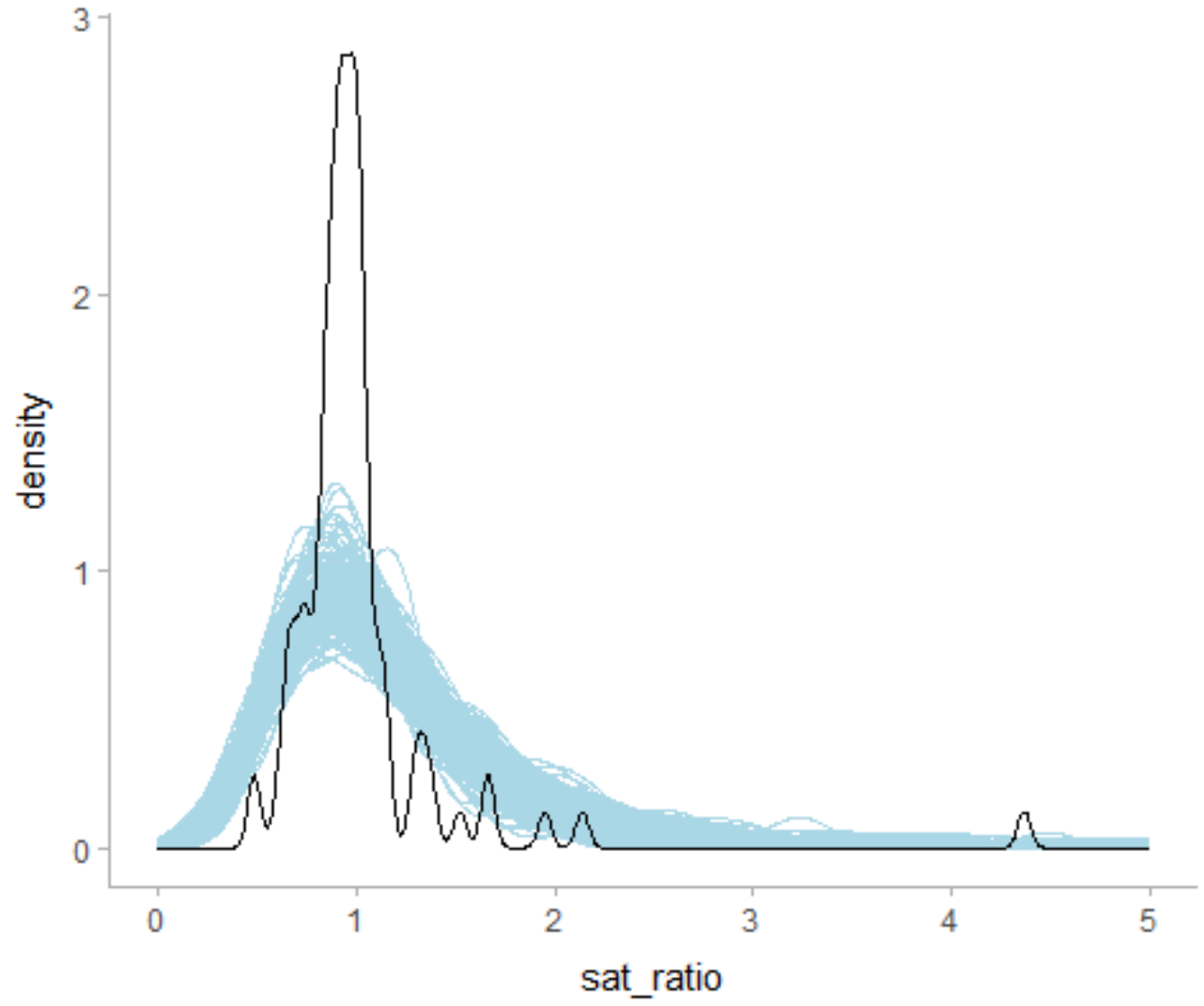

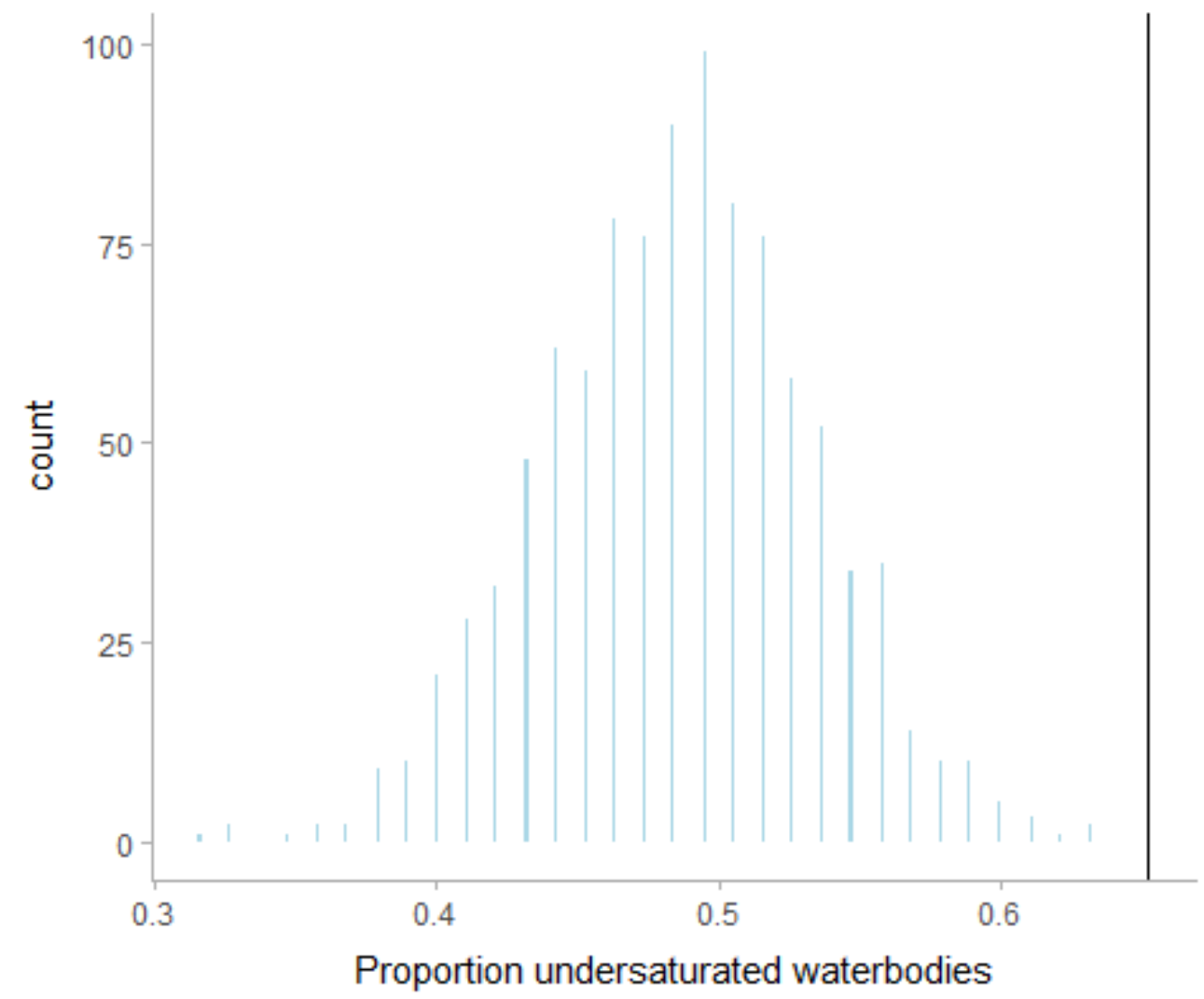

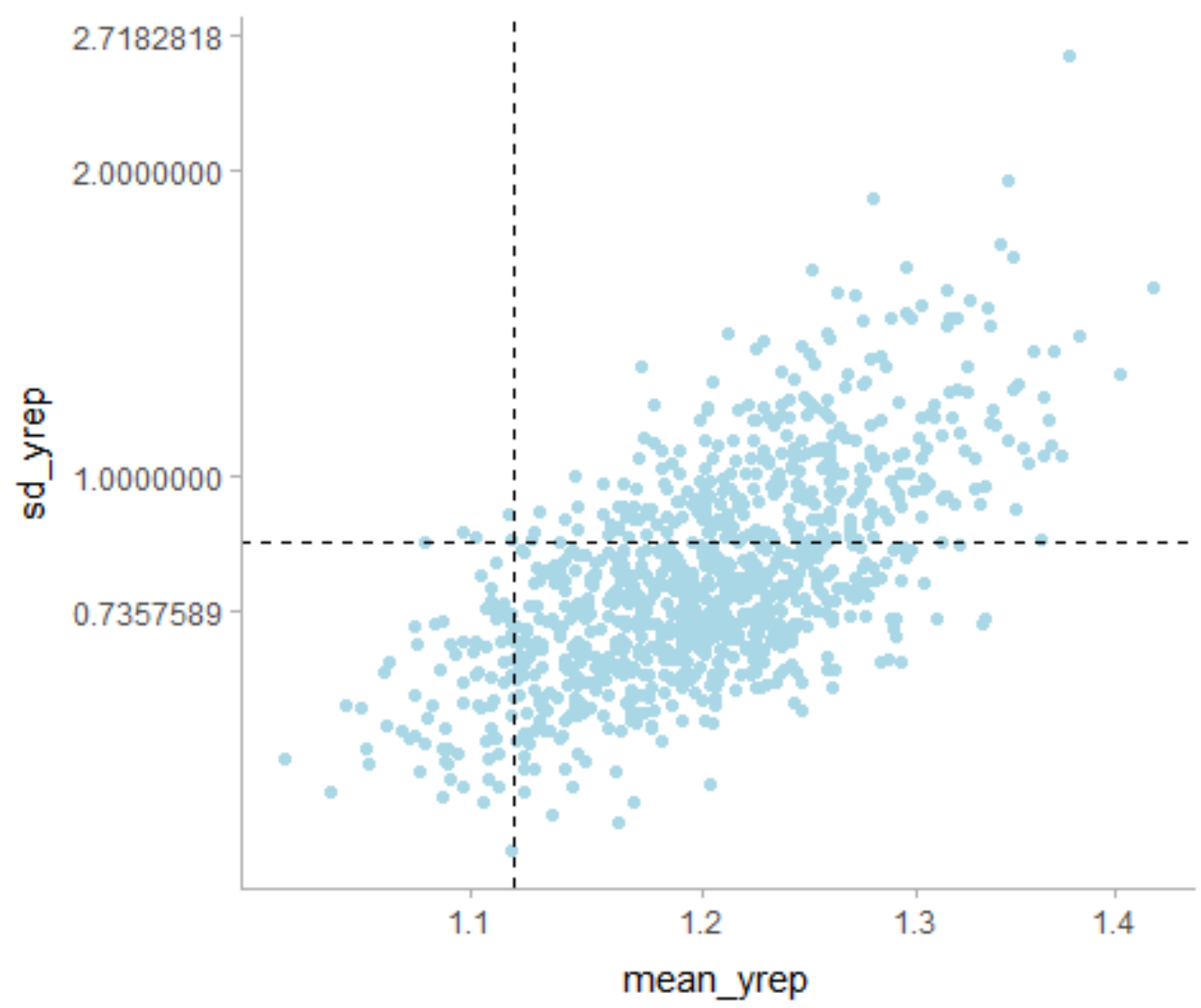

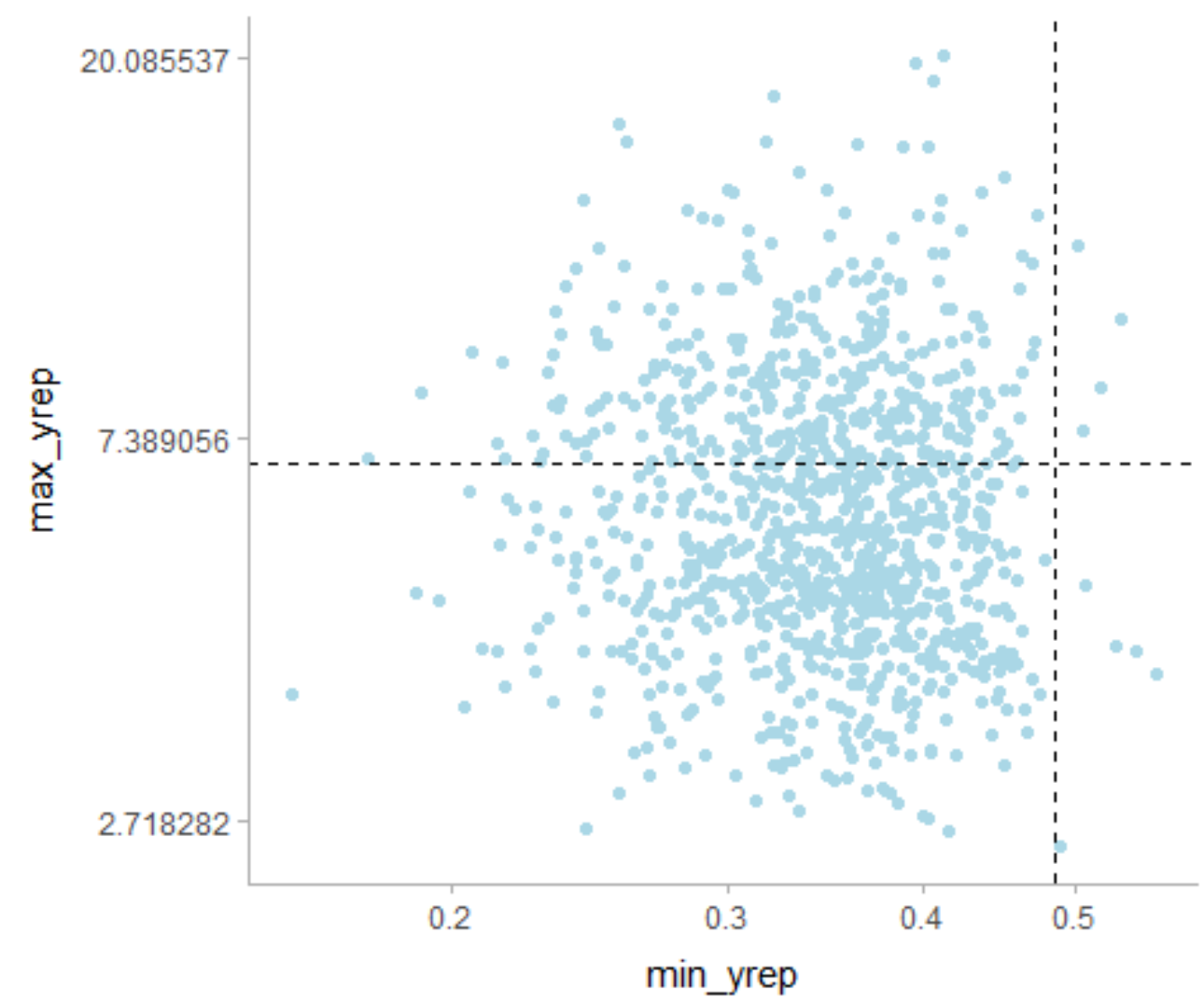

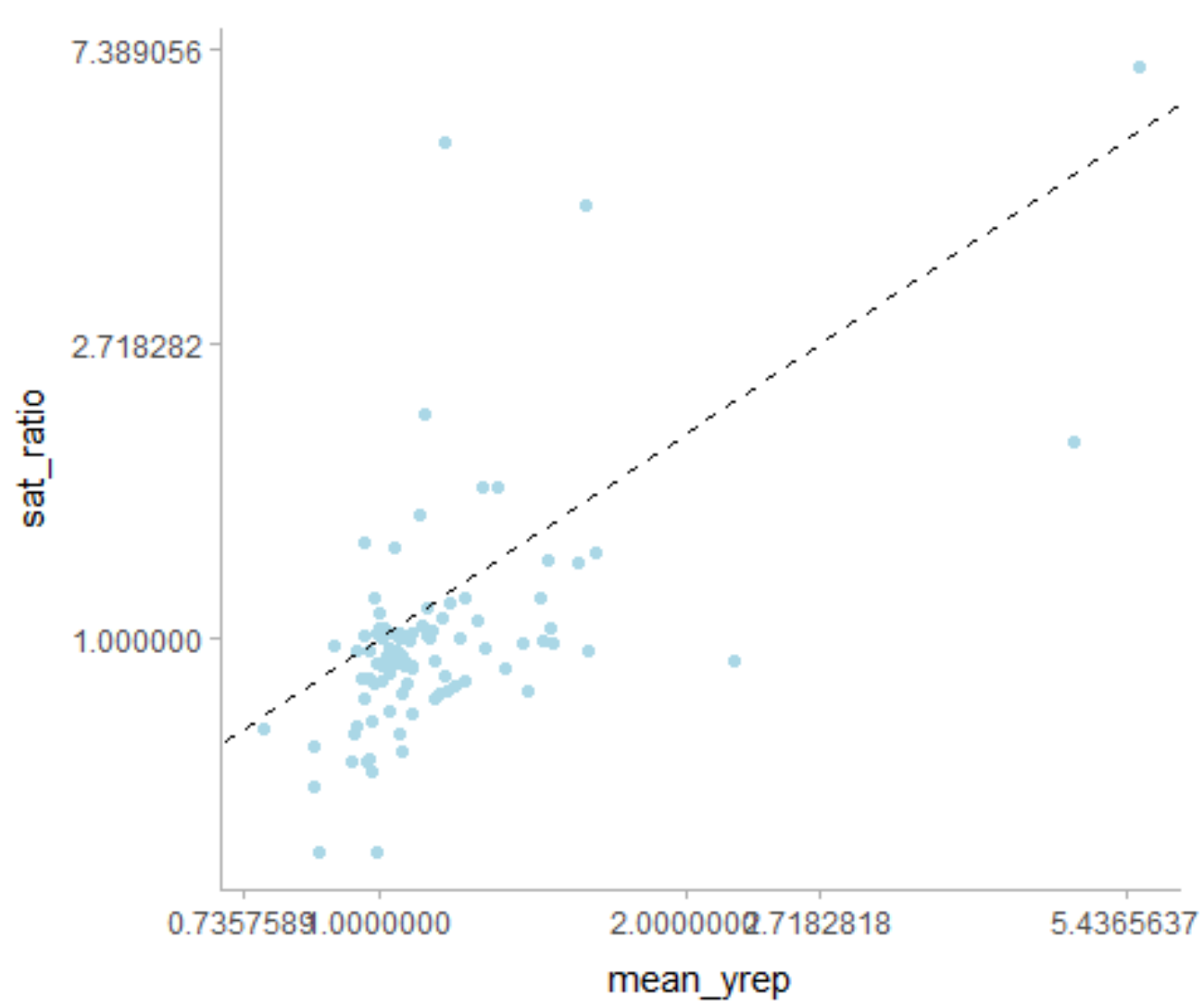

3.1.2.5 R-square

Below, the Bayesian R^2 values are reported for each response in the model.

```
##           Estimate Est.Error  Q2.5 Q97.5
## R2logn2o     0.247     0.031 0.187 0.309
```

```
##             Estimate Est.Error  Q2.5 Q97.5
## R2logn2oeq    0.377     0.025 0.328 0.425
```

The R^2 were also estimated for the re-visit data.

```
##           Estimate Est.Error  Q2.5 Q97.5
## R2logn2o    0.413      0.04 0.331 0.489

##             Estimate Est.Error  Q2.5 Q97.5
## R2logn2oeq    0.322     0.026 0.271 0.374
```

3.2 Model 2

In an attempt to better fit the observed data, the next model included a distributional sub-model that allowed for heterogeneous variances. The distributional terms for both N2O responses were each fit as a function of the survey design structure. The same structure as for the models for the submodels for the mean component before.

```
bf_n2o <- bf(log(n2o) ~ 1 +
               (1 | a | WSA9) +
               (1 | b | WSA9:state) +
               (1 | c | WSA9:state:size_cat),
             sigma ~ 1 +
               (1 | WSA9) +
               (1 | WSA9:state) +
               (1 | WSA9:state:size_cat),
             family = gaussian())

bf_n2oeq <- bf(log(n2o_eq) ~ 1 +
                 (1 | a | WSA9) +
                 (1 | b | WSA9:state) +
                 (1 | c | WSA9:state:size_cat),
             sigma ~ 1 +
               (1 | WSA9) +
               (1 | WSA9:state) +
               (1 | WSA9:state:size_cat),
             family = gaussian())

priors <- c(
  prior(normal(2, 1), class = "Intercept", resp = "logn2o"),
  prior(exponential(2), class = "sd", resp = "logn2o"),
  prior(normal(-1, 2), class = "Intercept", dpar = "sigma", resp = "logn2o"),
  prior(exponential(2), class = "sd", dpar = "sigma", resp = "logn2o"),
```

```
  prior(normal(2, 1), class = "Intercept", resp = "logn2oeq"),
  prior(exponential(2), class = "sd", resp = "logn2oeq"),
  prior(normal(-1, 2), class = "Intercept", dpar = "sigma", resp = "logn2oeq"),
  prior(exponential(2), class = "sd", dpar = "sigma", resp = "logn2oeq"),

  prior(lkj(2), class = "rescor"),
  prior(lkj(2), class = "cor")
  )

n2o_mod2 <- brm(bf_n2o + bf_n2oeq + set_rescor(rescor = TRUE),
                data = df_model,
                prior = priors,
  control = list(adapt_delta = 0.975, max_treedepth = 12),
  #sample_prior = "only",
  save_pars = save_pars(all = TRUE),
  seed = 84512,
  chains=4,
  iter=5000,
  cores=4)

save(n2o_mod2, file = "C:/Users/rmartin/OneDrive - Environmental Protection Agency (E
```

3.2.1 Summarize fit

The summaries of the estimated parameters and key HMC convergence diagnostics for the fitted model are printed below.

```
##  Family: MV(gaussian, gaussian)
##   Links: mu = identity; sigma = log
##          mu = identity; sigma = log
## Formula: log(n2o) ~ 1 + (1 | a | WSA9) + (1 | b | WSA9:state) + (1 | c | WSA9:stat
##          sigma ~ 1 + (1 | WSA9) + (1 | WSA9:state) + (1 | WSA9:state:size_cat)
##          log(n2o_eq) ~ 1 + (1 | a | WSA9) + (1 | b | WSA9:state) + (1 | c | WSA9:s
##          sigma ~ 1 + (1 | WSA9) + (1 | WSA9:state) + (1 | WSA9:state:size_cat)
##    Data: df_model (Number of observations: 984)
##   Draws: 4 chains, each with iter = 5000; warmup = 2500; thin = 1;
##          total post-warmup draws = 10000
##
## Priors:
## Intercept_logn2o ~ normal(2, 1)
## Intercept_logn2o_sigma ~ normal(-1, 2)
## Intercept_logn2oeq ~ normal(2, 1)
## Intercept_logn2oeq_sigma ~ normal(-1, 2)
## L ~ lkj_corr_cholesky(2)
## Lrescor ~ lkj_corr_cholesky(2)
## <lower=0> sd_logn2o ~ exponential(2)
## <lower=0> sd_logn2o_sigma ~ exponential(2)
```

```
## <lower=0> sd_logn2oeq ~ exponential(2)
## <lower=0> sd_logn2oeq_sigma ~ exponential(2)
##
## Multilevel Hyperparameters:
## ~WSA9 (Number of levels: 9)
##                                          Estimate Est.Error l-95% CI u-95% CI
## sd(logn2o_Intercept)                         0.06      0.03     0.02     0.12
## sd(logn2oeq_Intercept)                       0.05      0.02     0.03     0.09
## sd(sigma_logn2o_Intercept)                   0.22      0.12     0.02     0.50
## sd(sigma_logn2oeq_Intercept)                 0.16      0.08     0.04     0.34
## cor(logn2o_Intercept,logn2oeq_Intercept)     0.57      0.30    -0.18     0.95
##                                          Rhat Bulk_ESS Tail_ESS
## sd(logn2o_Intercept)                     1.00     2247     1772
## sd(logn2oeq_Intercept)                   1.00     3423     4761
## sd(sigma_logn2o_Intercept)               1.00     1892     3094
## sd(sigma_logn2oeq_Intercept)             1.00     2413     1991
## cor(logn2o_Intercept,logn2oeq_Intercept) 1.00     2696     3538
##
## ~WSA9:state (Number of levels: 96)
##                                          Estimate Est.Error l-95% CI u-95% CI
## sd(logn2o_Intercept)                         0.08      0.02     0.04     0.11
## sd(logn2oeq_Intercept)                       0.04      0.01     0.03     0.05
## sd(sigma_logn2o_Intercept)                   0.56      0.08     0.40     0.74
## sd(sigma_logn2oeq_Intercept)                 0.22      0.05     0.11     0.32
## cor(logn2o_Intercept,logn2oeq_Intercept)     0.42      0.19    -0.00     0.74
##                                          Rhat Bulk_ESS Tail_ESS
## sd(logn2o_Intercept)                     1.00     1276     1733
## sd(logn2oeq_Intercept)                   1.00     2695     5090
## sd(sigma_logn2o_Intercept)               1.00     2007     3462
## sd(sigma_logn2oeq_Intercept)             1.00     1851     1881
## cor(logn2o_Intercept,logn2oeq_Intercept) 1.00     1076     1960
##
## ~WSA9:state:size_cat (Number of levels: 352)
##                                          Estimate Est.Error l-95% CI u-95% CI
## sd(logn2o_Intercept)                         0.06      0.01     0.03     0.09
## sd(logn2oeq_Intercept)                       0.02      0.01     0.00     0.03
## sd(sigma_logn2o_Intercept)                   0.61      0.05     0.51     0.72
## sd(sigma_logn2oeq_Intercept)                 0.19      0.06     0.07     0.29
## cor(logn2o_Intercept,logn2oeq_Intercept)    -0.00      0.34    -0.70     0.62
##                                          Rhat Bulk_ESS Tail_ESS
## sd(logn2o_Intercept)                     1.00     1329     2182
## sd(logn2oeq_Intercept)                   1.00     1052     1452
## sd(sigma_logn2o_Intercept)               1.00     2622     4939
## sd(sigma_logn2oeq_Intercept)             1.00     1603     1725
## cor(logn2o_Intercept,logn2oeq_Intercept) 1.00      996     2063
##
## Regression Coefficients:
##                          Estimate Est.Error l-95% CI u-95% CI Rhat Bulk_ESS
## logn2o_Intercept             1.93      0.03     1.88     1.99 1.00     3338
```

```
## sigma_logn2o_Intercept      -1.40      0.12    -1.63    -1.17 1.00     3511
## logn2oeq_Intercept           2.00      0.02     1.96     2.03 1.00     3384
## sigma_logn2oeq_Intercept    -2.60      0.07    -2.75    -2.45 1.00     4004
##                          Tail_ESS
## logn2o_Intercept             4462
## sigma_logn2o_Intercept       5077
## logn2oeq_Intercept           4145
## sigma_logn2oeq_Intercept     4848
##
## Residual Correlations: 
##                         Estimate Est.Error l-95% CI u-95% CI Rhat Bulk_ESS
## rescor(logn2o,logn2oeq)     0.36      0.03     0.29     0.43 1.00     6474
##                         Tail_ESS
## rescor(logn2o,logn2oeq)     7733
##
## Draws were sampled using sampling(NUTS). For each parameter, Bulk_ESS
## and Tail_ESS are effective sample size measures, and Rhat is the potential
## scale reduction factor on split chains (at convergence, Rhat = 1).
```

3.2.2 Model checks

Below the same PPCs were performed as with the initial model (see above for more details on each panel). Though the checks below suggested some improvement in replicating the tails of the observed data, the overall fit again suggested room for improvement.

3.2.2.0.1 Dissolved N2O

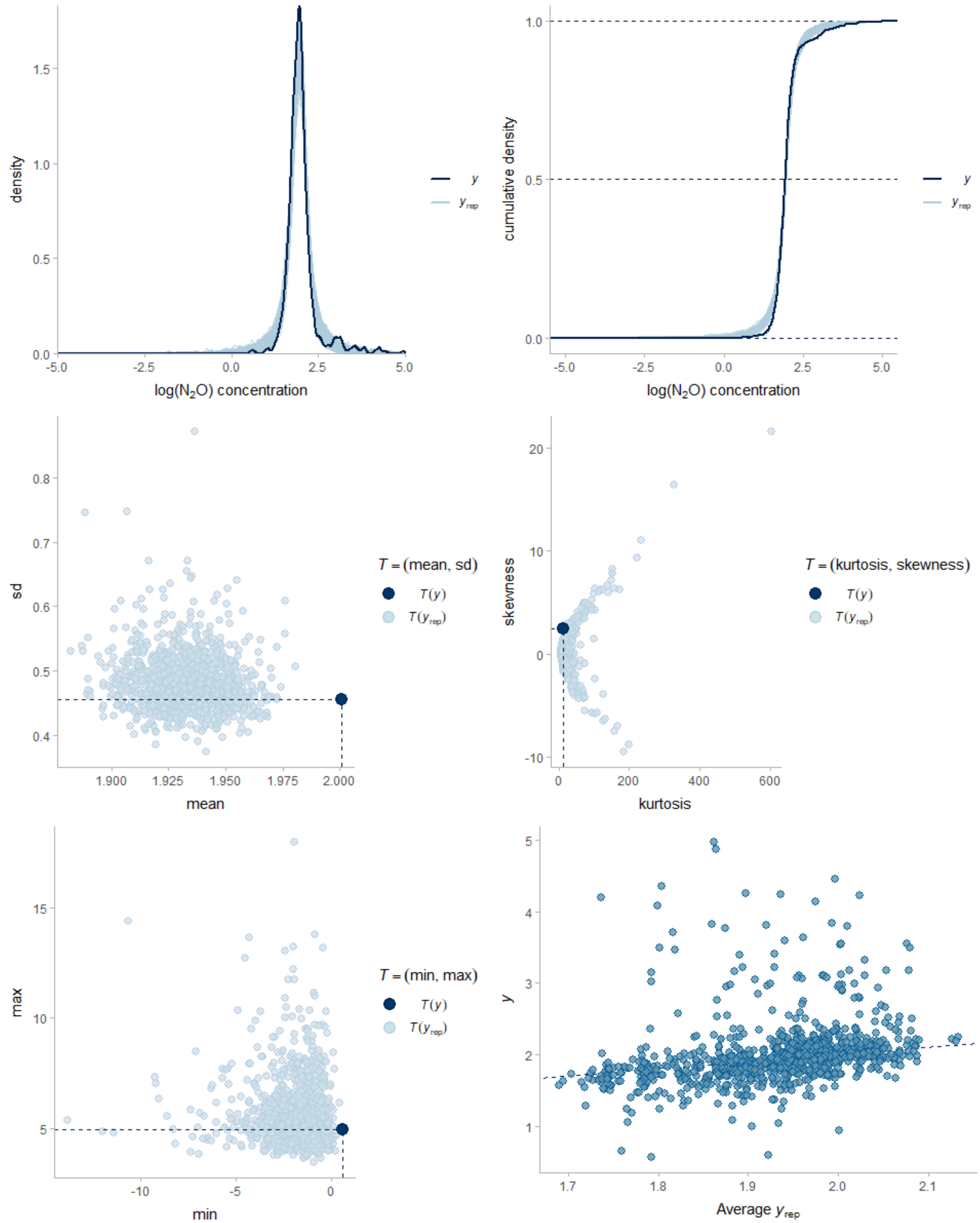

The same check is below for the 95 re-visit sites.

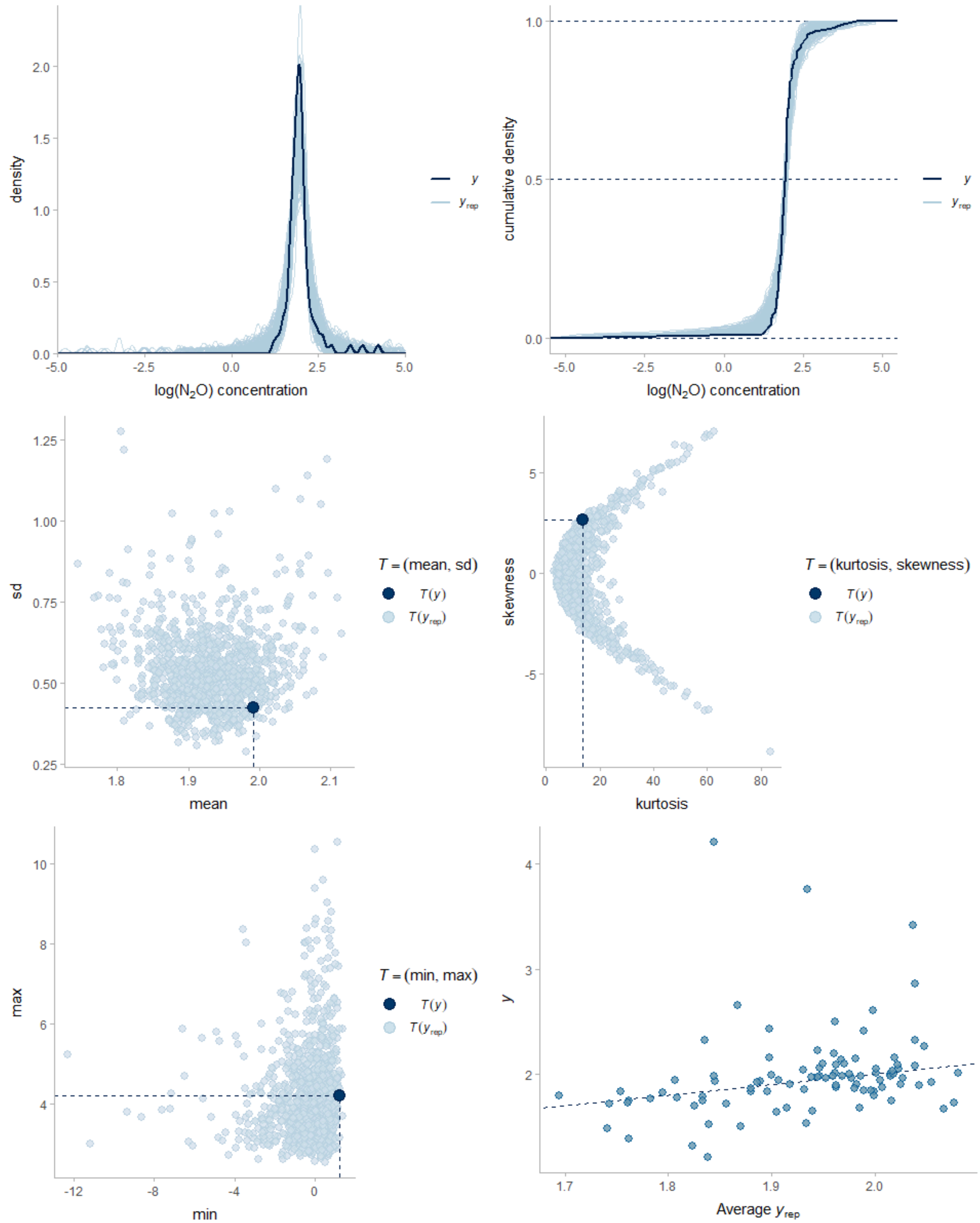

3.2.2.1 Equilibrium N2O

The checks below suggested that this model offered little improvement upon the initial model for equilibrium N2O.

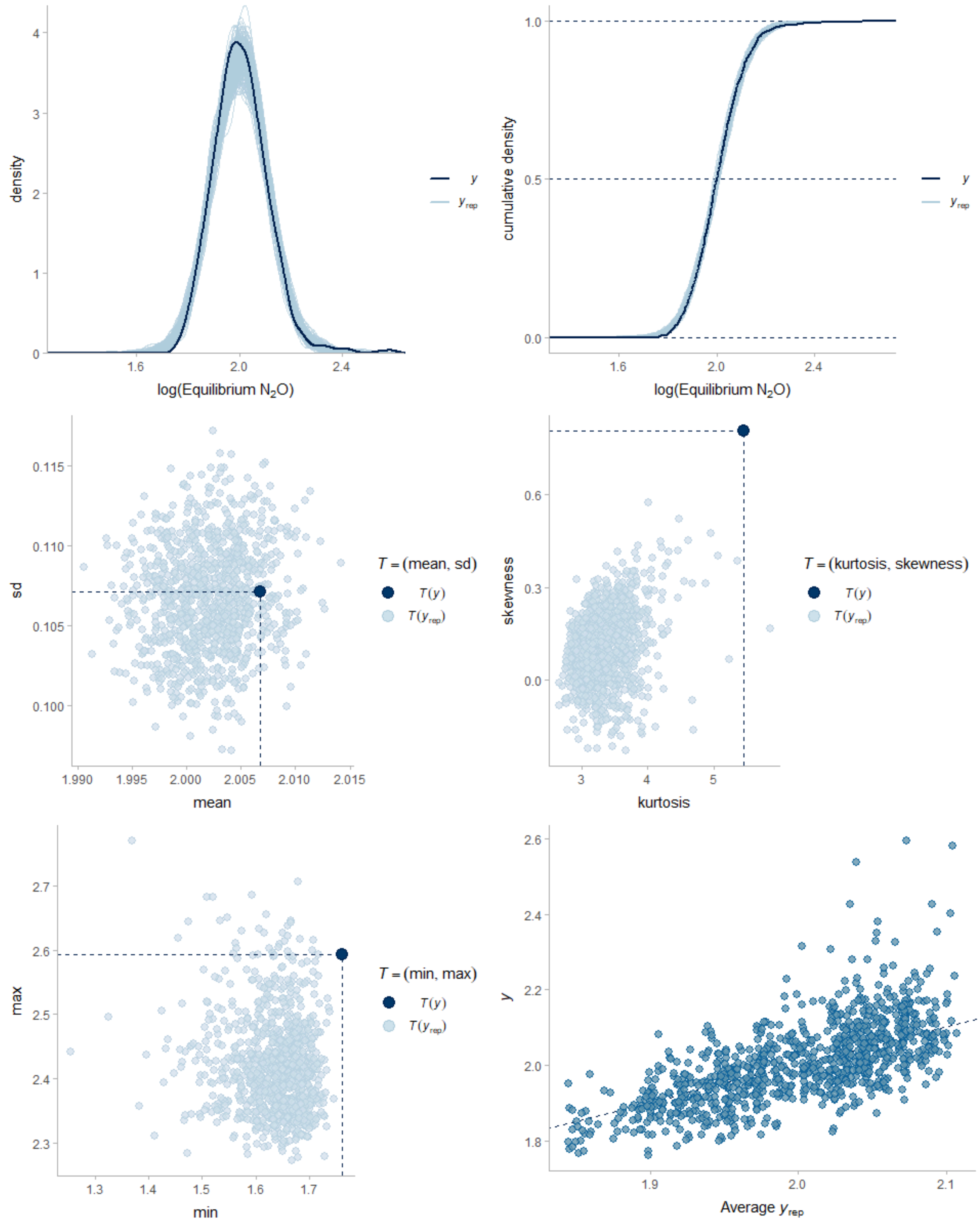

The same check with the re-visit data is below.

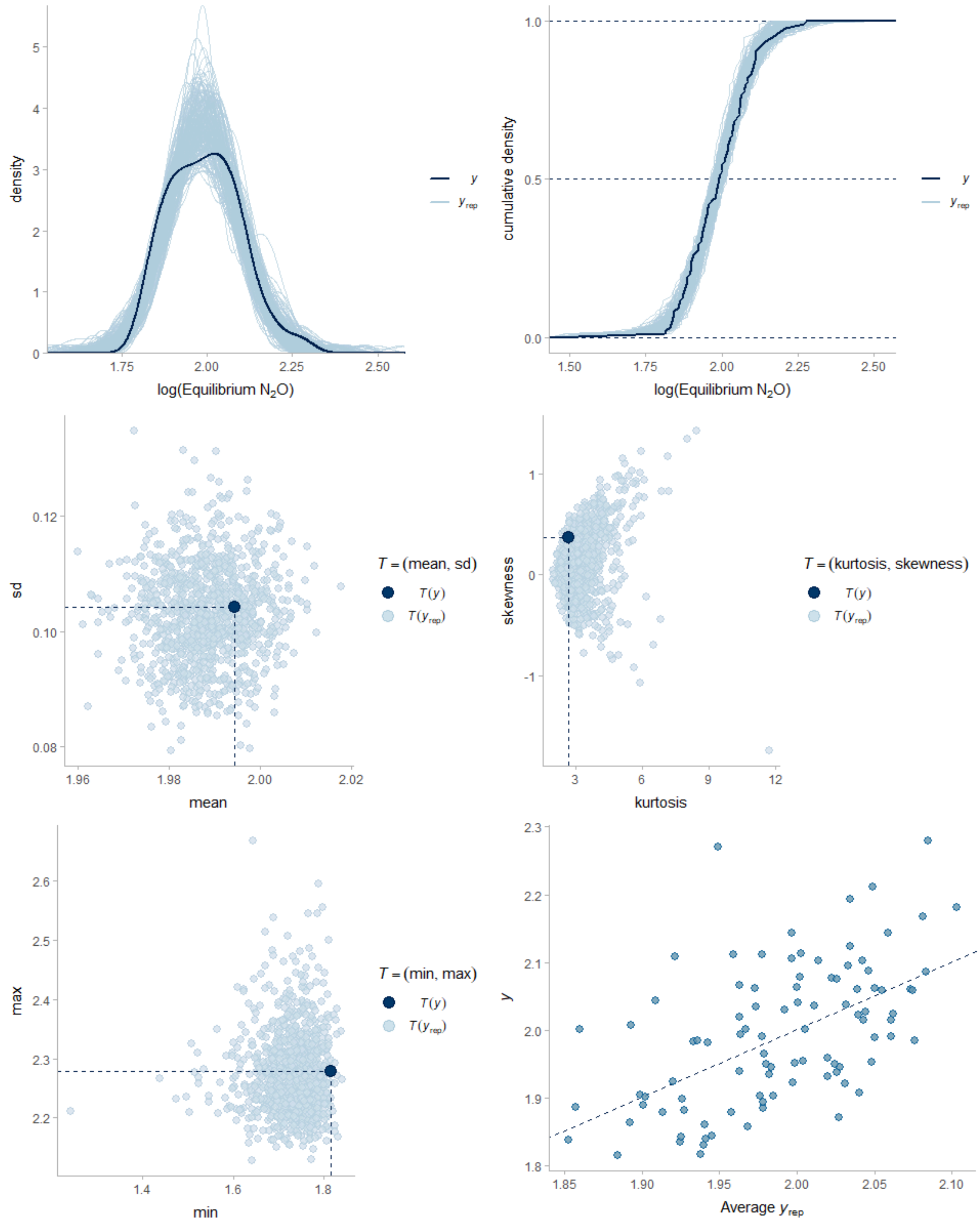

3.2.2.2 Bivariate

This check perhaps suggested an improvement with regard to replicating the joint density. However, the predictions were still clearly over-dispersed relative to the observations.

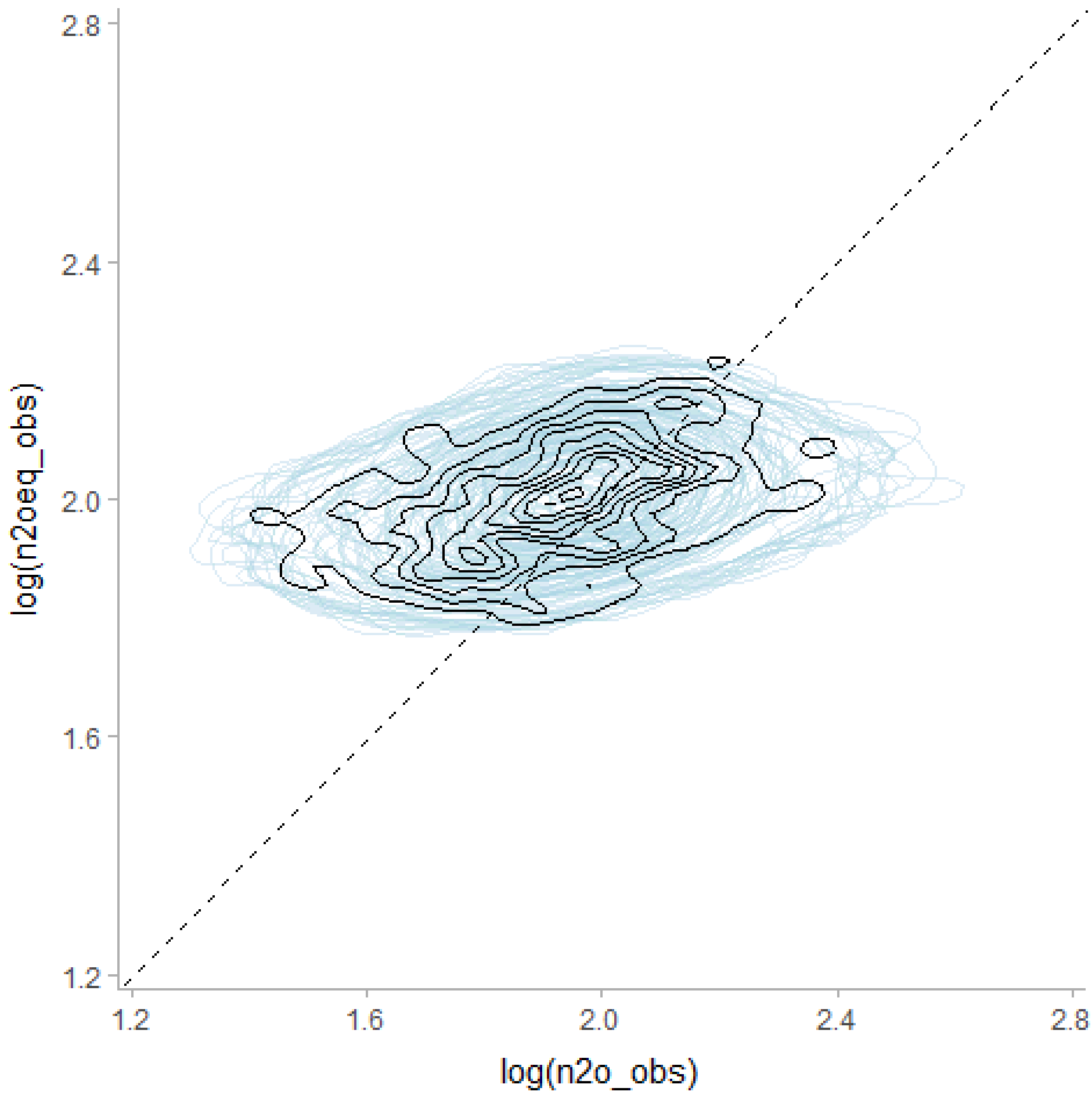

The same check with the re-visit data is below.

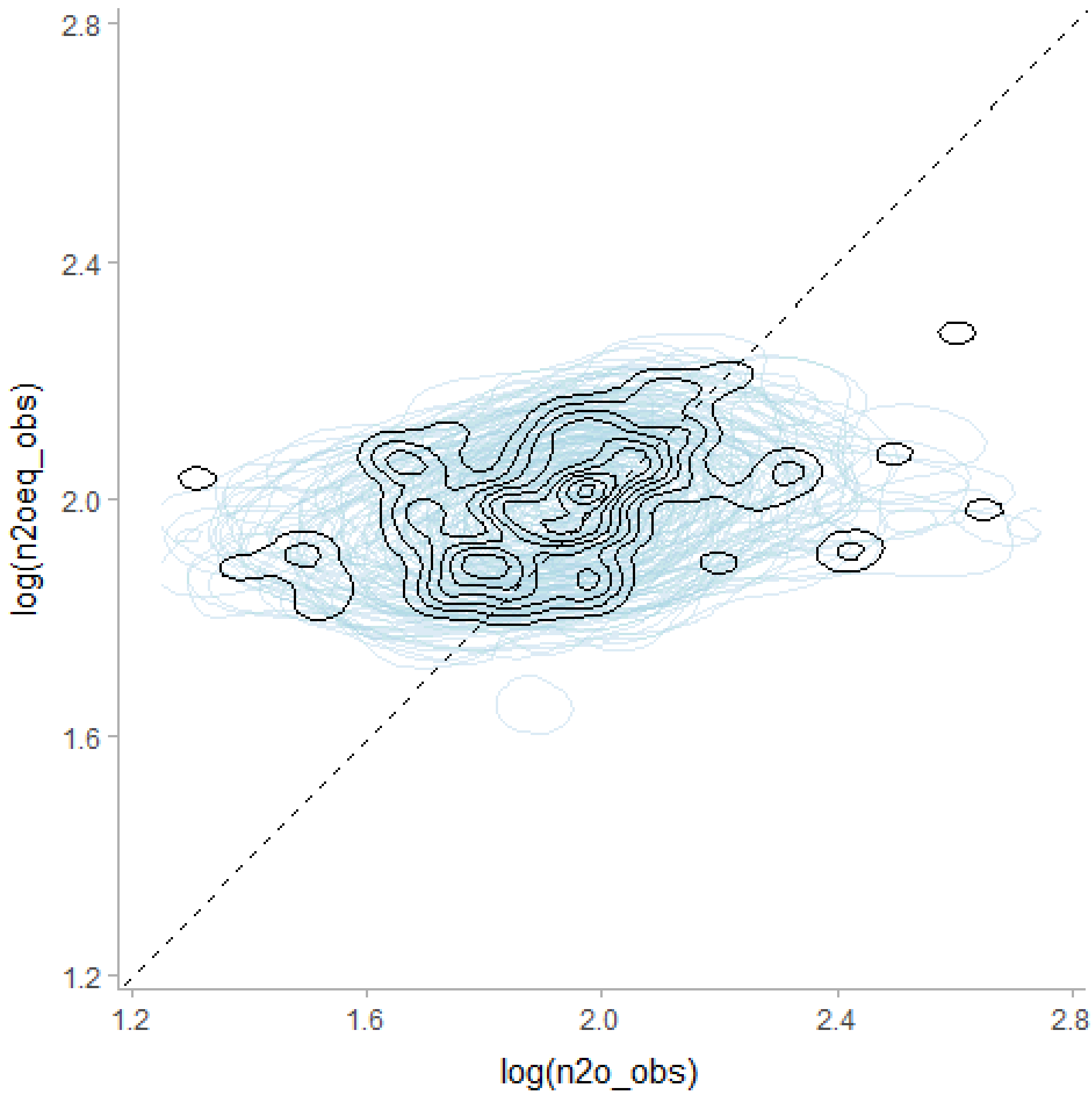

3.2.2.3 Saturation

The PPCs for the saturation metrics below indicated that including the distributional models was perhaps an improvement on the initial model in some aspects; in particular, the bias in the predicted proportion of under-saturated lakes was substantially decreased. However, there appeared to still be issues in replicating the tails as well as issues with central tendency.

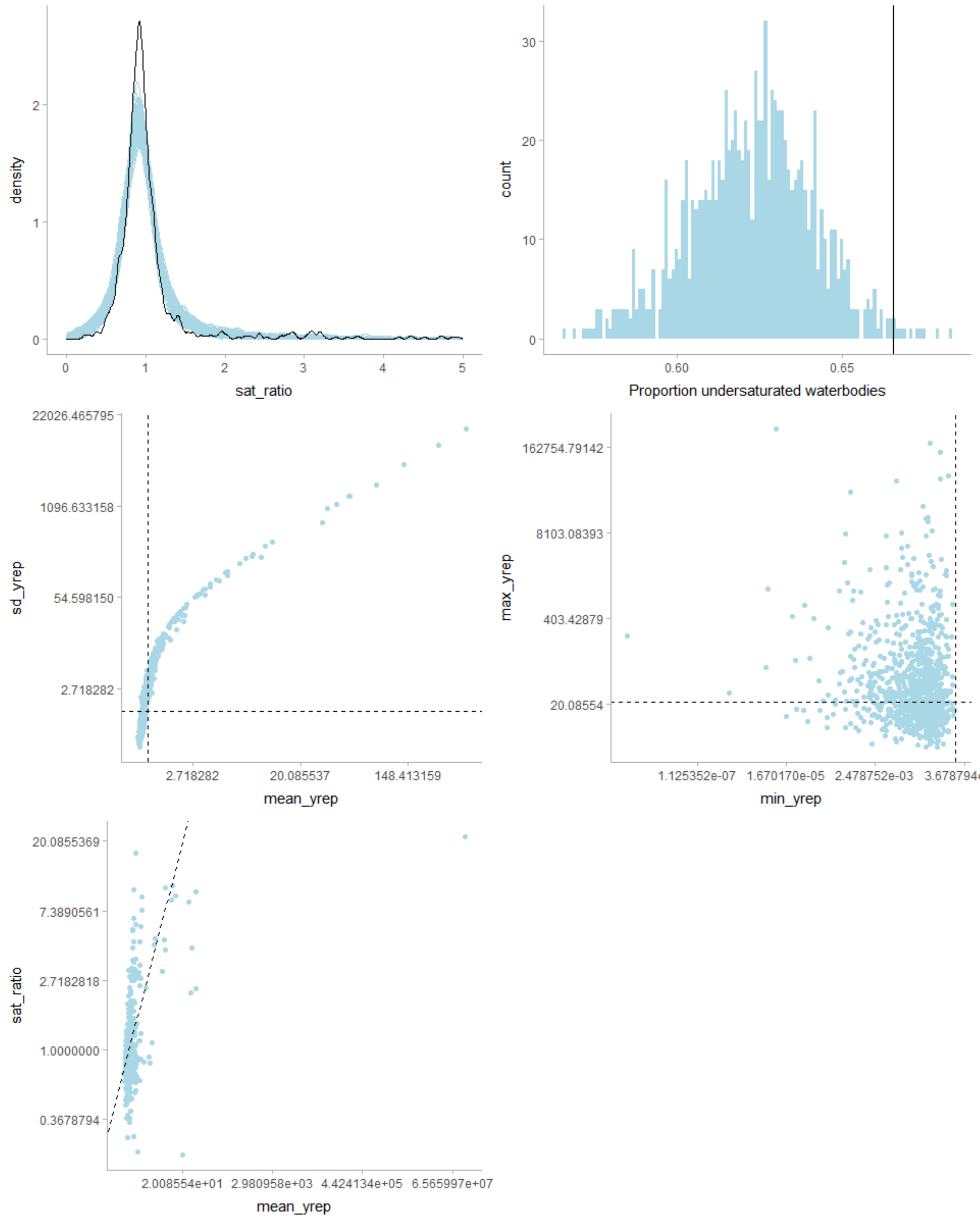

The check for the revisit data is below.

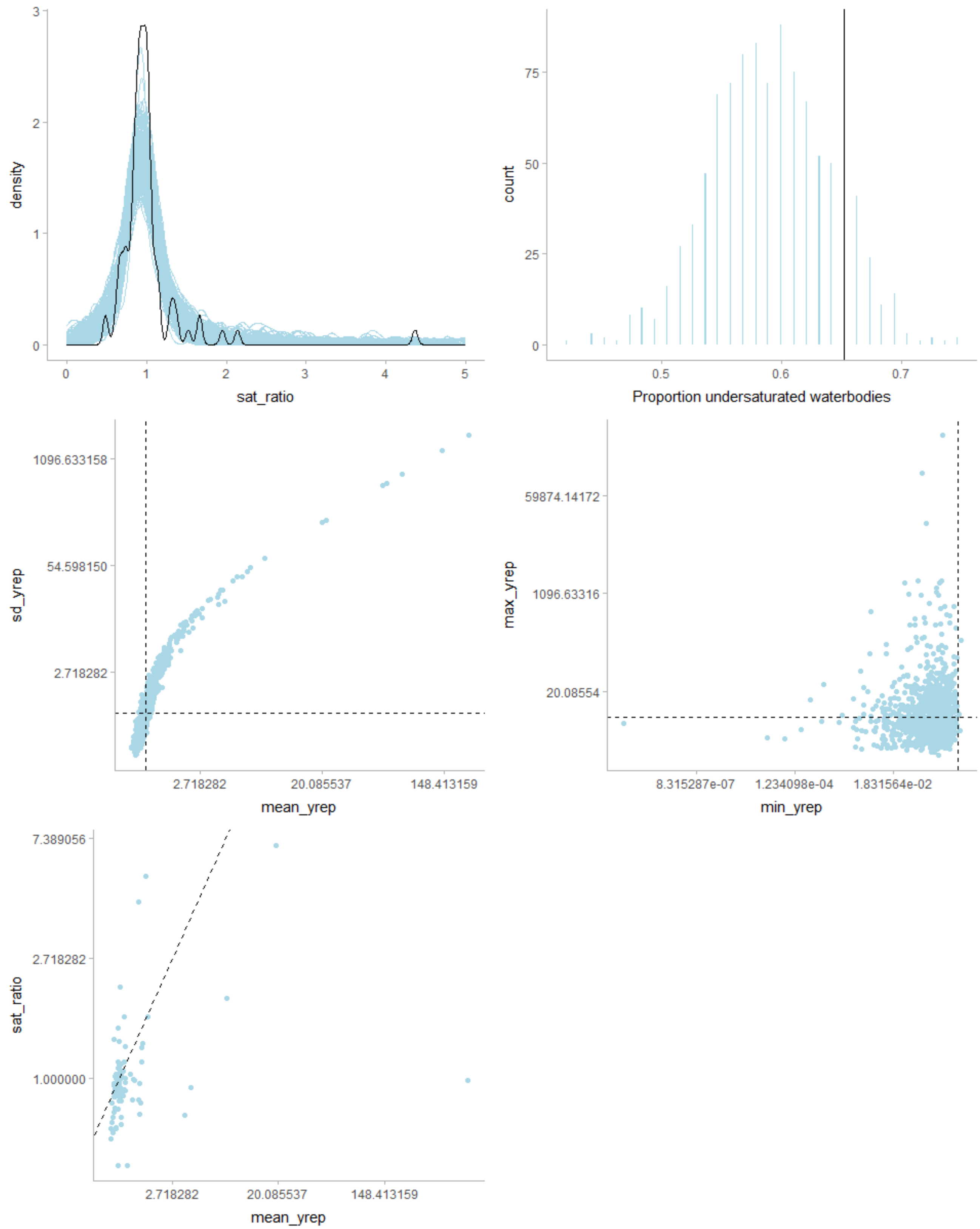

3.2.2.4 R-square

Relative to model 1, there was a substantial decrease in the R^2 estimate for the dissolved N2O component of this model. The estimate for the equilibrium N2O-eq component was similar to model 1.

```
##          Estimate Est.Error  Q2.5 Q97.5
## R2logn2o    0.056     0.009 0.038 0.075

##            Estimate Est.Error  Q2.5 Q97.5
## R2logn2oeq    0.379     0.022 0.335 0.421
```

And for the re-visit data:

```
##          Estimate Est.Error  Q2.5 Q97.5
## R2logn2o    0.071     0.014 0.046   0.1

##            Estimate Est.Error  Q2.5 Q97.5
## R2logn2oeq    0.323     0.023 0.278 0.369
```

3.3 Model 3

In the next model, the categorical version of the NO3 covariate and a surface temperature covariate were included to try to improve the fit. The ordinal NO3 variable was used as a monotonic, ordinal effect and only in the dissolved N2O component of the model. For the equlibrium N2O component, surface temperature and log-transformed elevation were used, along with their interaction. The model also retained the same distributional specifications included in model 2 above.

```
bf_n2o <- bf(log(n2o) ~ mo(no3_cat) +
               surftemp +
               (mo(no3_cat) | a | WSA9) +
               (mo(no3_cat) | b | WSA9:state) +
               (1 | c | WSA9:state:size_cat),
             sigma ~ 1 +
               (1 | WSA9) +
               (1 | WSA9:state) +
               (1 | WSA9:state:size_cat),
             family = gaussian())

bf_n2oeq <- bf(log(n2o_eq) ~ surftemp +
                 log_elev +
                 surftemp:log_elev +
                 (1 | a | WSA9) +
                 (1 | b | WSA9:state) +
                 (1 | c | WSA9:state:size_cat),
             sigma ~ 1 +
               (1 | WSA9) +
```

```
              (1 | WSA9:state) +
              (1 | WSA9:state:size_cat),
            family = gaussian())

priors <- c(
  prior(normal(2, 1), class = "Intercept", resp = "logn2o"),
  prior(normal(0, 1), class = "b", resp = "logn2o"),
  prior(exponential(2), class = "sd", resp = "logn2o"),
  prior(normal(-1, 2), class = "Intercept", dpar = "sigma", resp = "logn2o"),
  prior(exponential(2), class = "sd", dpar = "sigma", resp = "logn2o"),
  prior(normal(2, 1), class = "Intercept", resp = "logn2oeq"),
  prior(normal(0, 1), class = "b", resp = "logn2oeq"),
  prior(exponential(2), class = "sd", resp = "logn2oeq"),
  prior(normal(-1, 2), class = "Intercept", dpar = "sigma", resp = "logn2oeq"),
  prior(exponential(2), class = "sd", dpar = "sigma", resp = "logn2oeq"),

  prior(lkj(2), class = "rescor"),
  prior(lkj(2), class = "cor")
  )

n2o_mod3 <- brm(bf_n2o + bf_n2oeq + set_rescor(rescor = TRUE),
                data = df_model,
                prior = priors,
  control = list(adapt_delta = 0.975, max_treedepth = 12),
  #sample_prior = "only",
  save_pars = save_pars(all = TRUE),
  seed = 98456,
  chains=4,
  iter=5000,
  cores=4)

save(n2o_mod3, file = "C:/Users/rmartin/OneDrive - Environmental Protection Agency (E
```

3.3.1 Summarize fit

The fitted parameters and MCMC diagnostics are below.

```
##  Family: MV(gaussian, gaussian)
##   Links: mu = identity; sigma = log
##          mu = identity; sigma = log
## Formula: log(n2o) ~ mo(no3_cat) + surftemp + (mo(no3_cat) | a | WSA9) + (mo(no3_ca
##          sigma ~ 1 + (1 | WSA9) + (1 | WSA9:state) + (1 | WSA9:state:size_cat)
##          log(n2o_eq) ~ surftemp + log_elev + surftemp:log_elev + (1 | a | WSA9) +
##          sigma ~ 1 + (1 | WSA9) + (1 | WSA9:state) + (1 | WSA9:state:size_cat)
##    Data: df_model (Number of observations: 984)
##   Draws: 4 chains, each with iter = 5000; warmup = 2500; thin = 1;
##          total post-warmup draws = 10000
```

```
##
## Priors:
## b_logn2o ~ normal(0, 1)
## b_logn2oeq ~ normal(0, 1)
## Intercept_logn2o ~ normal(2, 1)
## Intercept_logn2o_sigma ~ normal(-1, 2)
## Intercept_logn2oeq ~ normal(2, 1)
## Intercept_logn2oeq_sigma ~ normal(-1, 2)
## L ~ lkj_corr_cholesky(2)
## Lrescor ~ lkj_corr_cholesky(2)
## <lower=0> sd_logn2o ~ exponential(2)
## <lower=0> sd_logn2o_sigma ~ exponential(2)
## <lower=0> sd_logn2oeq ~ exponential(2)
## <lower=0> sd_logn2oeq_sigma ~ exponential(2)
## simo_logn2o_mono3_cat1 ~ dirichlet(1)
##
## Multilevel Hyperparameters:
## ~WSA9 (Number of levels: 9)
##                                          Estimate Est.Error l-95% CI u-95% CI
## sd(logn2o_Intercept)                         0.05      0.02     0.02     0.10
## sd(logn2o_mono3_cat)                         0.14      0.05     0.06     0.26
## sd(logn2oeq_Intercept)                       0.04      0.01     0.02     0.07
## sd(sigma_logn2o_Intercept)                   0.12      0.08     0.01     0.31
## sd(sigma_logn2oeq_Intercept)                 0.36      0.11     0.19     0.64
## cor(logn2o_Intercept,logn2o_mono3_cat)      -0.14      0.33    -0.72     0.53
## cor(logn2o_Intercept,logn2oeq_Intercept)     0.36      0.29    -0.28     0.83
## cor(logn2o_mono3_cat,logn2oeq_Intercept)     0.37      0.29    -0.26     0.82
##                                          Rhat Bulk_ESS Tail_ESS
## sd(logn2o_Intercept)                     1.00     4265     3917
## sd(logn2o_mono3_cat)                     1.00     4747     5482
## sd(logn2oeq_Intercept)                   1.00     4798     5704
## sd(sigma_logn2o_Intercept)               1.00     3419     4599
## sd(sigma_logn2oeq_Intercept)             1.00     5027     6638
## cor(logn2o_Intercept,logn2o_mono3_cat)   1.00     4970     6018
## cor(logn2o_Intercept,logn2oeq_Intercept) 1.00     5073     6636
## cor(logn2o_mono3_cat,logn2oeq_Intercept) 1.00     7256     7538
##
## ~WSA9:state (Number of levels: 96)
##                                          Estimate Est.Error l-95% CI u-95% CI
## sd(logn2o_Intercept)                         0.03      0.02     0.00     0.07
## sd(logn2o_mono3_cat)                         0.14      0.02     0.10     0.18
## sd(logn2oeq_Intercept)                       0.03      0.00     0.03     0.04
## sd(sigma_logn2o_Intercept)                   0.30      0.09     0.09     0.47
## sd(sigma_logn2oeq_Intercept)                 0.28      0.06     0.17     0.40
## cor(logn2o_Intercept,logn2o_mono3_cat)      -0.30      0.32    -0.82     0.45
## cor(logn2o_Intercept,logn2oeq_Intercept)     0.00      0.27    -0.53     0.55
## cor(logn2o_mono3_cat,logn2oeq_Intercept)     0.45      0.13     0.16     0.69
##                                          Rhat Bulk_ESS Tail_ESS
## sd(logn2o_Intercept)                     1.00      894     2100
```

```
## sd(logn2o_mono3_cat)                      1.00     3795     5396
## sd(logn2oeq_Intercept)                    1.00     4164     5957
## sd(sigma_logn2o_Intercept)                1.00     1157     1279
## sd(sigma_logn2oeq_Intercept)              1.00     2747     4313
## cor(logn2o_Intercept,logn2o_mono3_cat)    1.00      550      693
## cor(logn2o_Intercept,logn2oeq_Intercept) 1.01      596      971
## cor(logn2o_mono3_cat,logn2oeq_Intercept) 1.00     2536     4431
##
## ~WSA9:state:size_cat (Number of levels: 352)
##                                          Estimate Est.Error l-95% CI u-95% CI
## sd(logn2o_Intercept)                         0.06      0.01     0.04     0.08
## sd(logn2oeq_Intercept)                       0.00      0.00     0.00     0.01
## sd(sigma_logn2o_Intercept)                   0.58      0.06     0.47     0.70
## sd(sigma_logn2oeq_Intercept)                 0.28      0.05     0.18     0.39
## cor(logn2o_Intercept,logn2oeq_Intercept)     0.26      0.39    -0.60     0.87
##                                          Rhat Bulk_ESS Tail_ESS
## sd(logn2o_Intercept)                     1.00     1429     2386
## sd(logn2oeq_Intercept)                   1.00     1779     3895
## sd(sigma_logn2o_Intercept)               1.00     2003     4421
## sd(sigma_logn2oeq_Intercept)             1.00     1812     3745
## cor(logn2o_Intercept,logn2oeq_Intercept) 1.00     4523     5554
##
## Regression Coefficients:
##                            Estimate Est.Error l-95% CI u-95% CI Rhat Bulk_ESS
## logn2o_Intercept               2.40      0.05     2.29     2.50 1.00     5046
## sigma_logn2o_Intercept        -1.70      0.08    -1.87    -1.55 1.00     5162
## logn2oeq_Intercept             3.10      0.05     3.01     3.19 1.00     8903
## sigma_logn2oeq_Intercept      -3.54      0.14    -3.81    -3.27 1.00     4065
## logn2o_surftemp               -0.02      0.00    -0.03    -0.02 1.00     5529
## logn2oeq_surftemp             -0.04      0.00    -0.04    -0.04 1.00     9722
## logn2oeq_log_elev             -0.07      0.01    -0.09    -0.06 1.00     9200
## logn2oeq_surftemp:log_elev     0.00      0.00     0.00     0.00 1.00     9527
## logn2o_mono3_cat               0.23      0.05     0.12     0.34 1.00     3948
##                            Tail_ESS
## logn2o_Intercept               6296
## sigma_logn2o_Intercept         5452
## logn2oeq_Intercept             7808
## sigma_logn2oeq_Intercept       5338
## logn2o_surftemp                6684
## logn2oeq_surftemp              7658
## logn2oeq_log_elev              7505
## logn2oeq_surftemp:log_elev     8028
## logn2o_mono3_cat               4777
##
## Monotonic Simplex Parameters:
##                      Estimate Est.Error l-95% CI u-95% CI Rhat Bulk_ESS
## logn2o_mono3_cat1[1]     0.02      0.01     0.00     0.04 1.00     5535
## logn2o_mono3_cat1[2]     0.09      0.02     0.04     0.13 1.00     4281
## logn2o_mono3_cat1[3]     0.21      0.05     0.13     0.32 1.00     3683
```

```
## logn2o_mono3_cat1[4]     0.69      0.05     0.58     0.77 1.00     3498
##                      Tail_ESS
## logn2o_mono3_cat1[1]     5409
## logn2o_mono3_cat1[2]     5251
## logn2o_mono3_cat1[3]     4649
## logn2o_mono3_cat1[4]     4251
##
## Residual Correlations:
##                         Estimate Est.Error l-95% CI u-95% CI Rhat Bulk_ESS
## rescor(logn2o,logn2oeq)     0.15      0.04     0.07     0.23 1.00    11381
##                         Tail_ESS
## rescor(logn2o,logn2oeq)     7610
##
## Draws were sampled using sampling(NUTS). For each parameter, Bulk_ESS
## and Tail_ESS are effective sample size measures, and Rhat is the potential
## scale reduction factor on split chains (at convergence, Rhat = 1).
```

3.3.2 Model checks

3.3.2.1 Dissolved N2O

The PPCs below indicated a better fit compared to the previous models. The central tendency and tail behavior looked to be reasonably replicated by comparison. However, the observed *vs.* predicted plot suggested that larger N2O observations were likely being systematically underestimated.

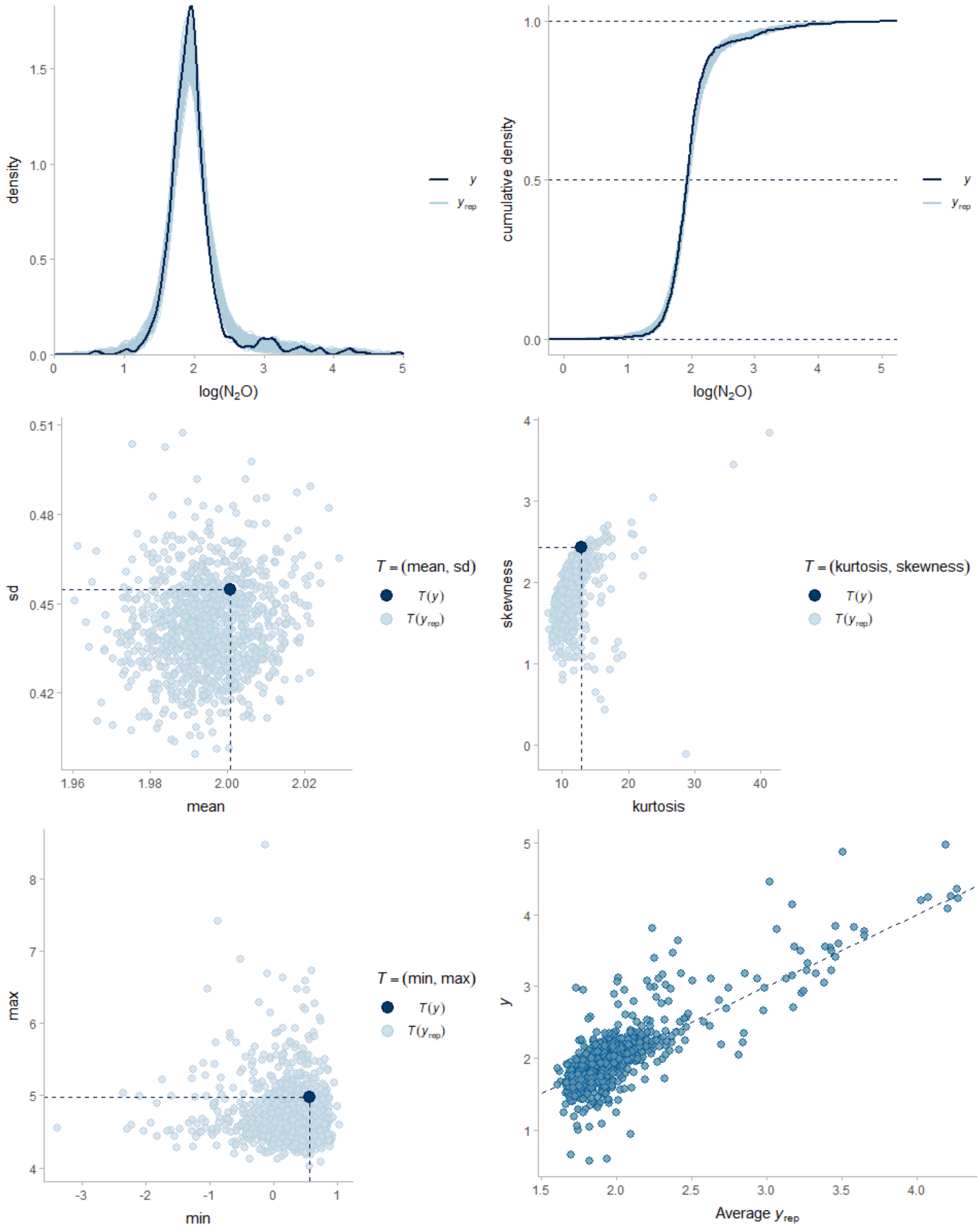

Below are the same PPCs for the second-visit data.

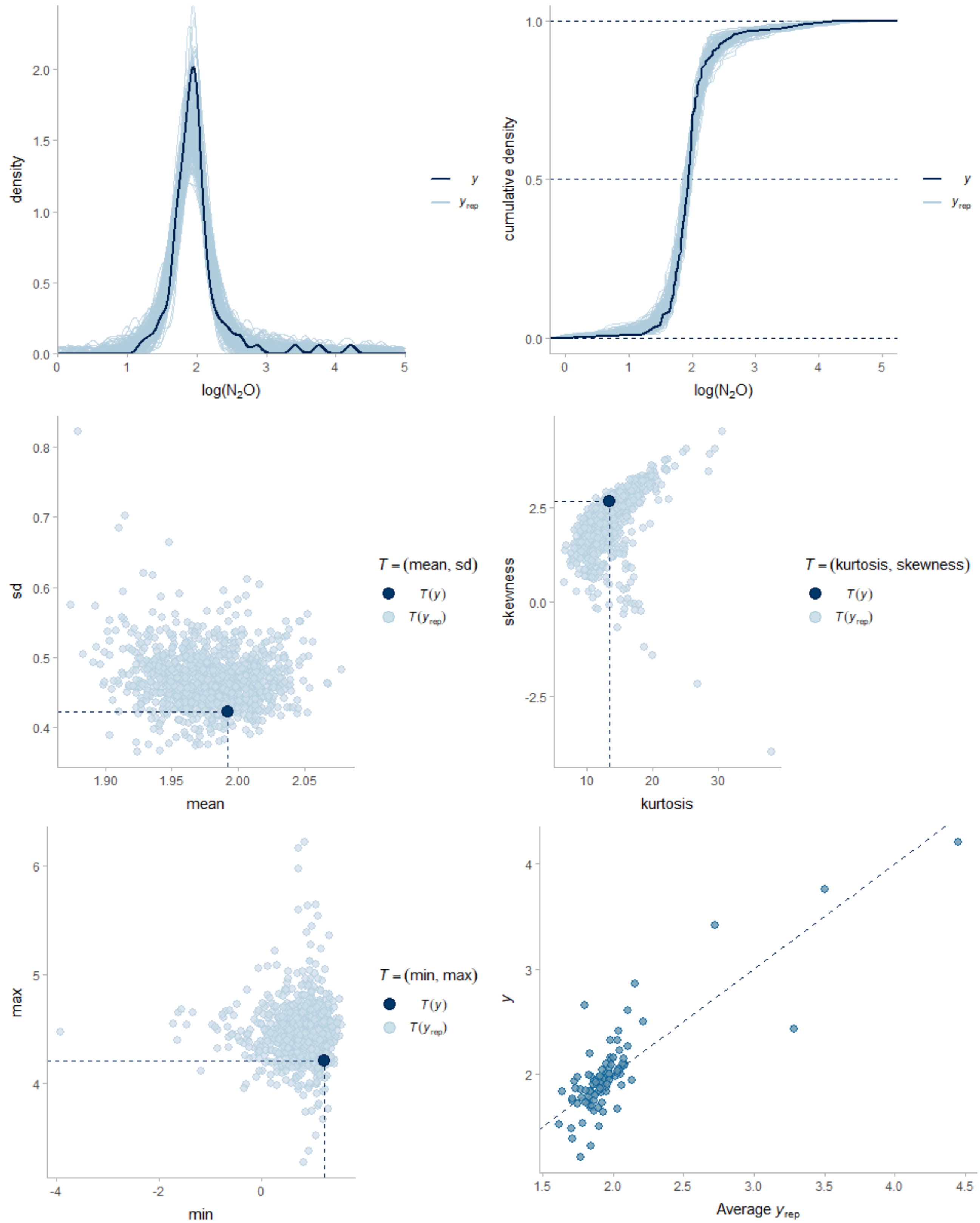

3.3.2.2 Equilibrium N2O

The PPCs below indicated that this model appeared to be an improvement for equilibrium N2O as well. However, some checks (e.g., skewness) suggested some room for additional improvement.

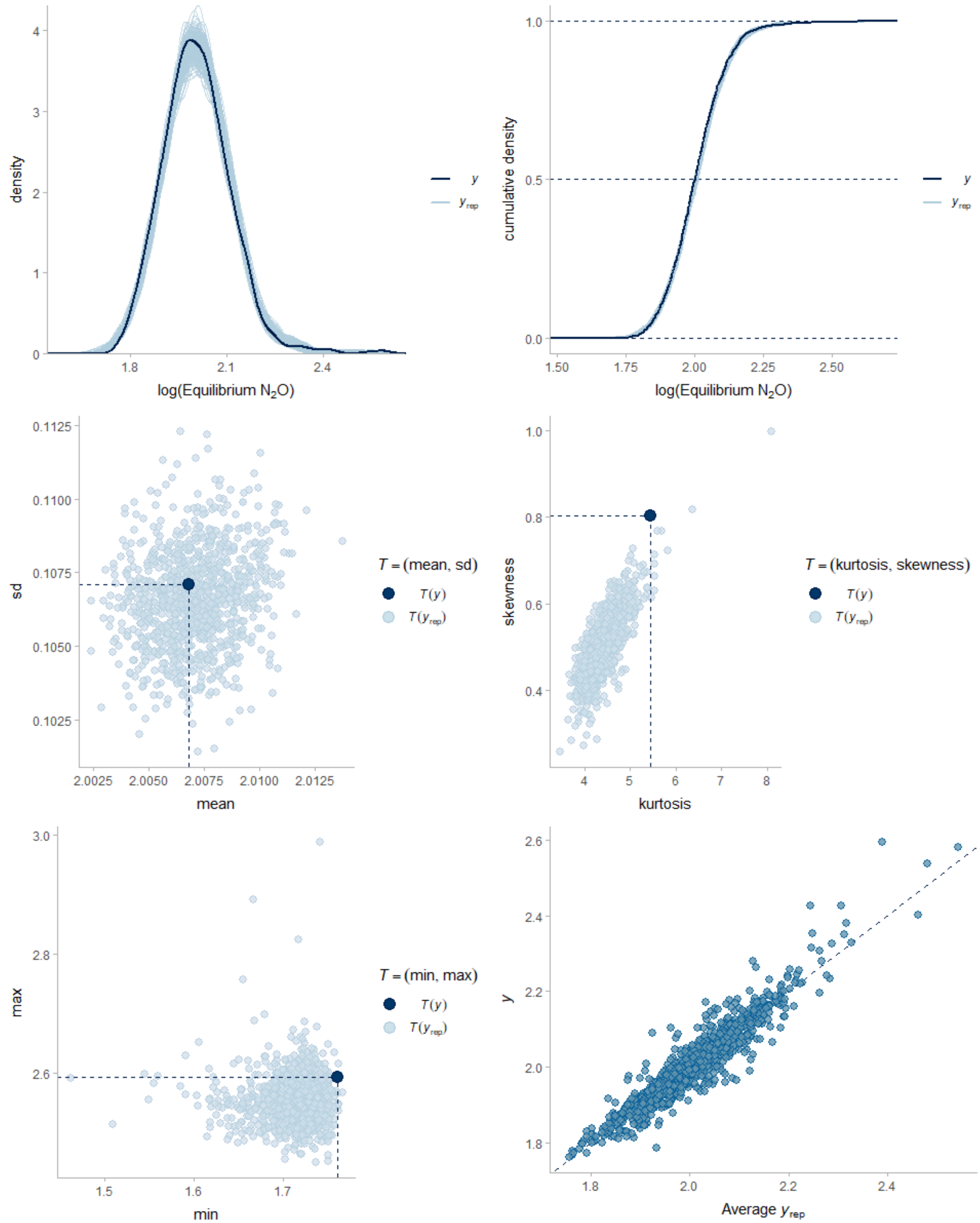

Checks for the 2nd visit data are below.

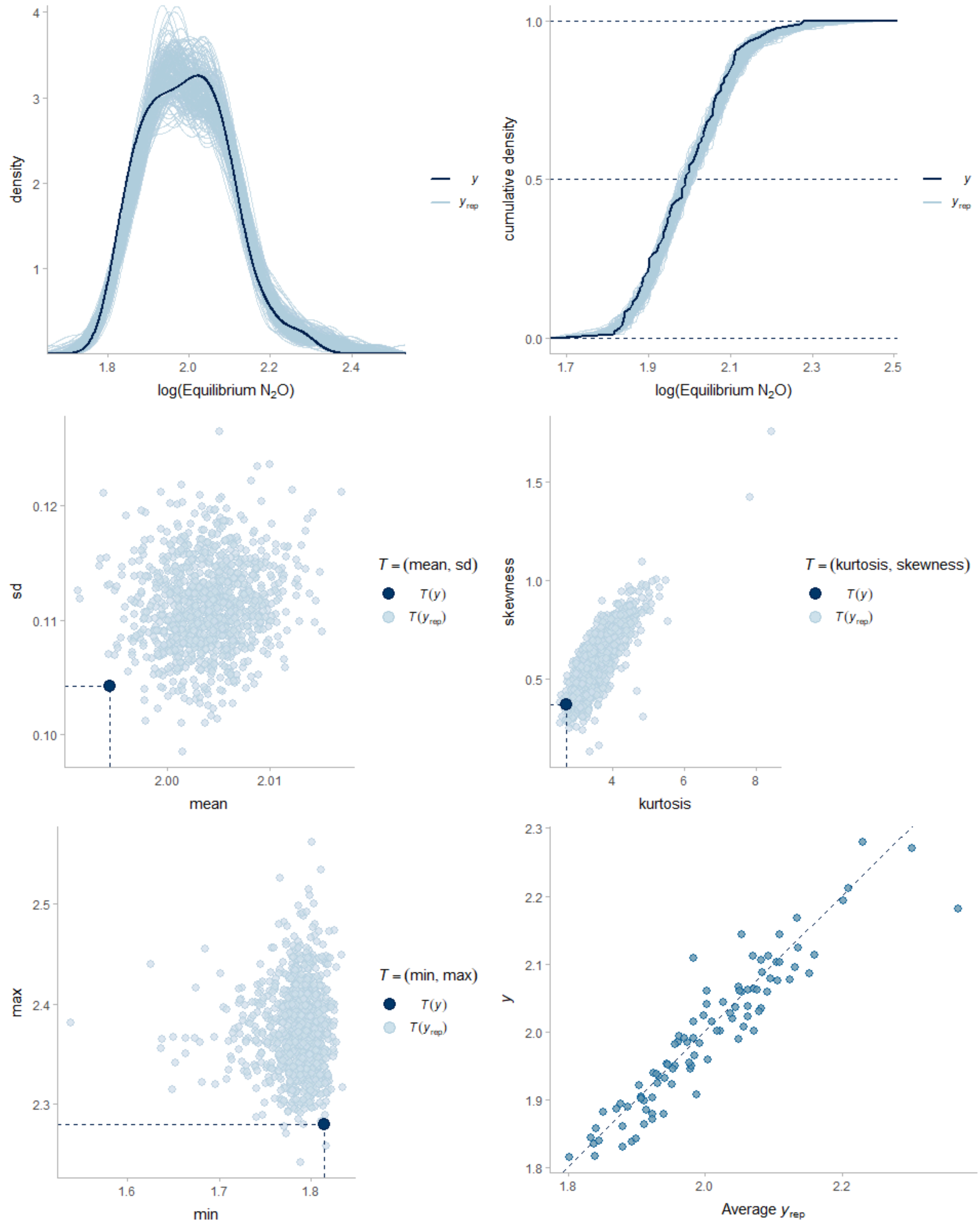

3.3.2.3 Bivariate

The check for the joint distribution below also suggested an improvement upon the previous models.

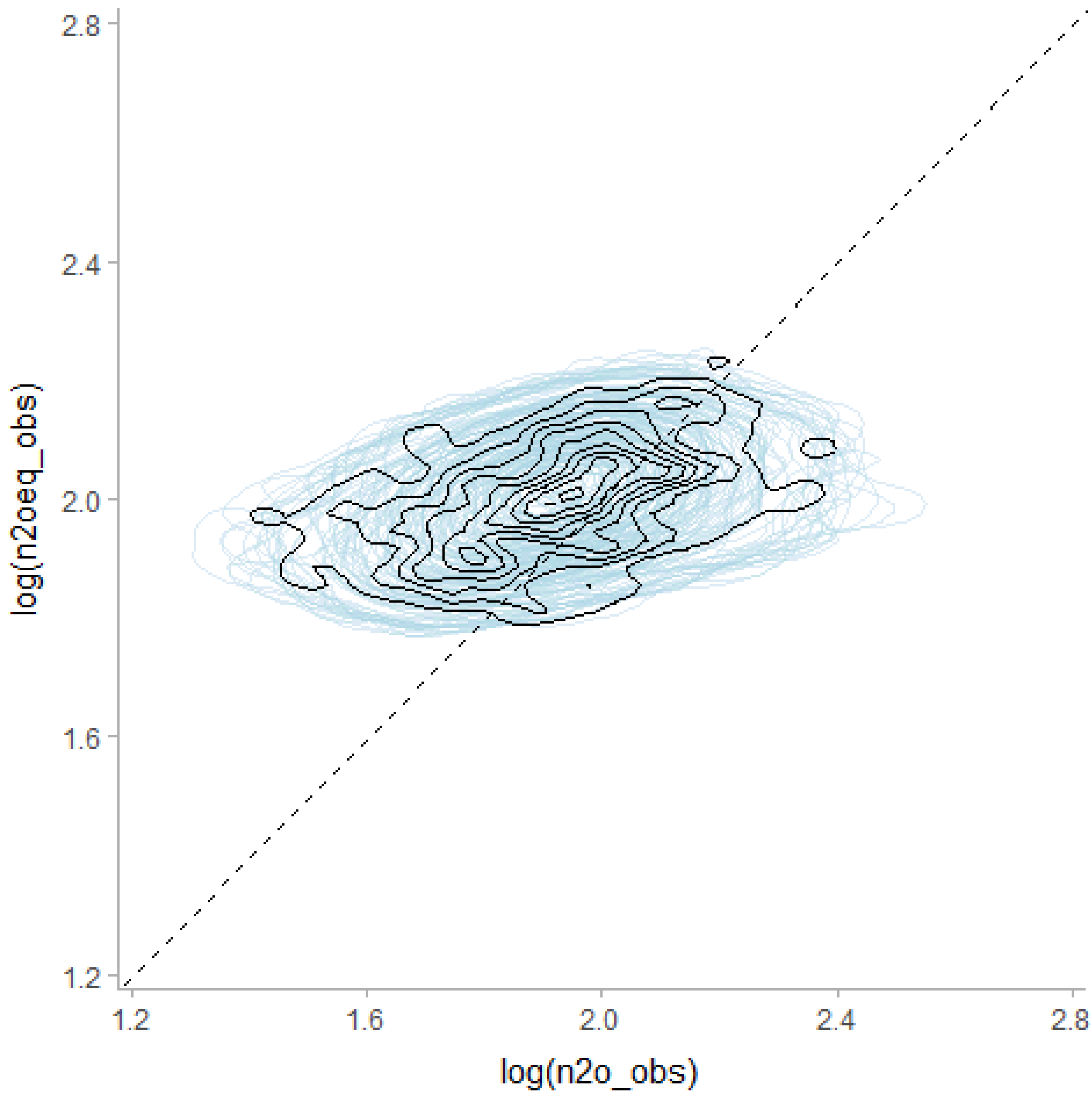

The same check below for the second-visit sites.

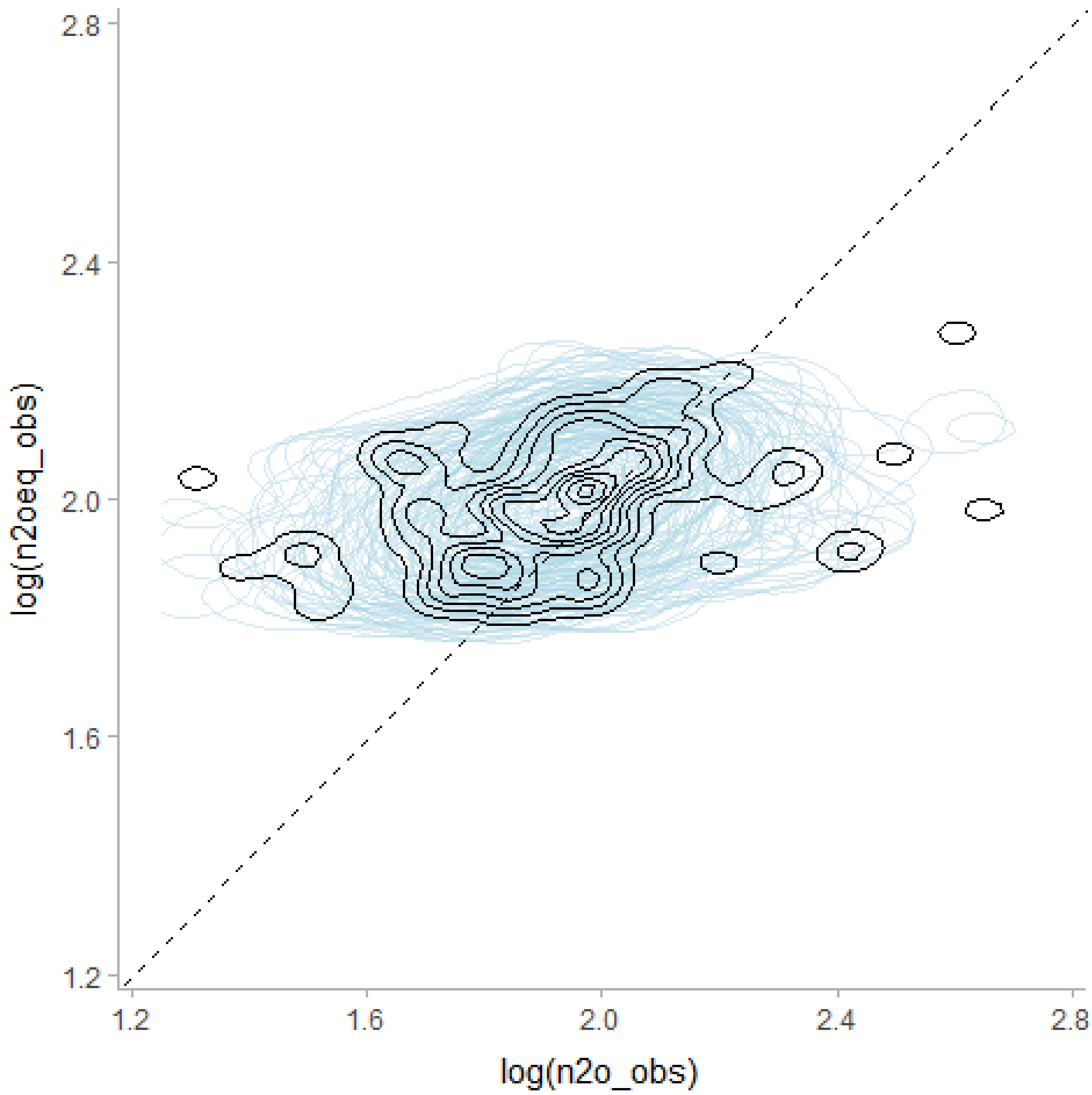

3.3.2.4 Saturation

This model looked to be an improvement with regard to the PPCs for the saturation metrics. However, the proportion of under-saturated lakes remained biased low and other checks indicated that further improvements would be ideal.

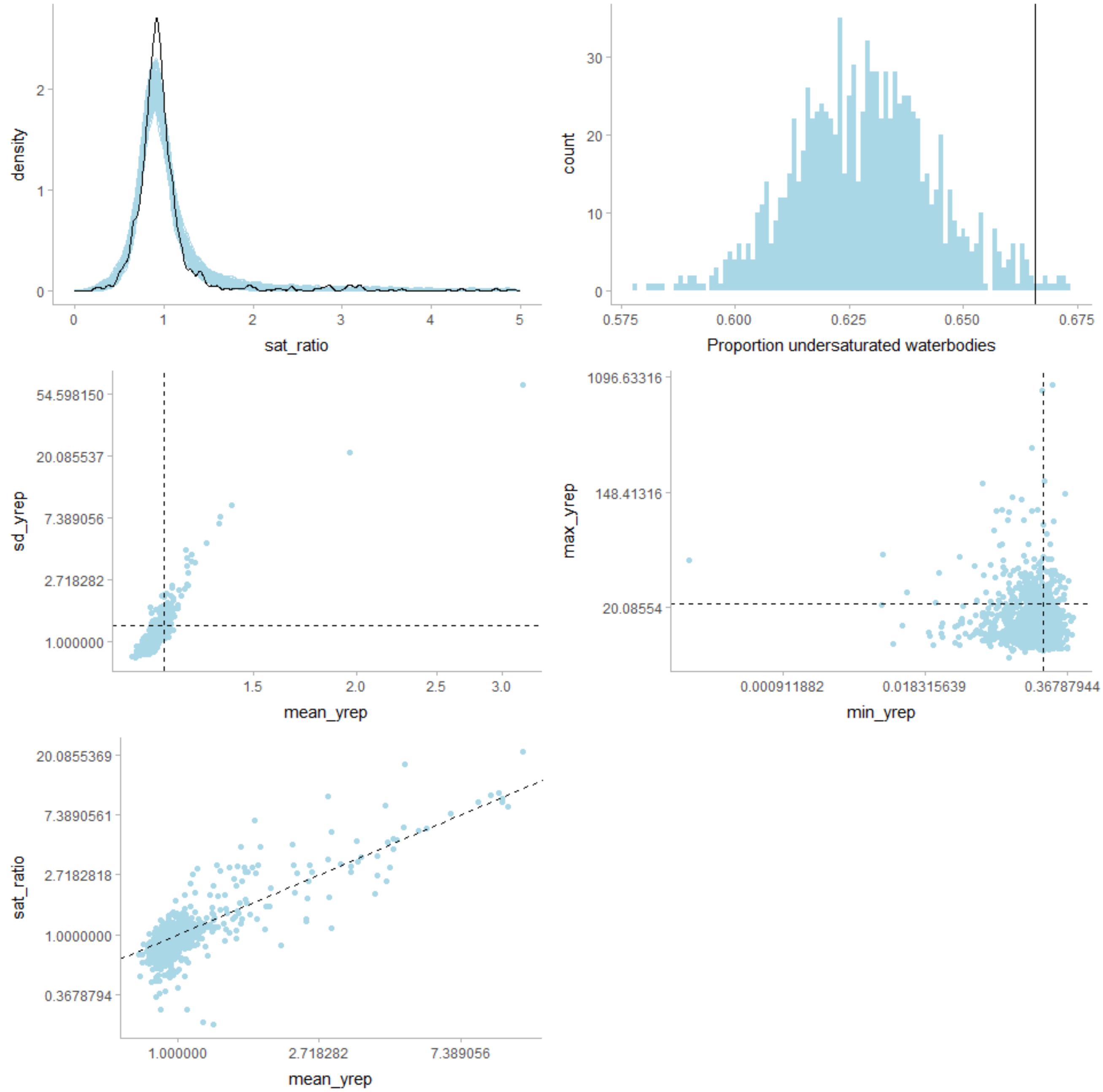

The same check for the second-visit sites are next.

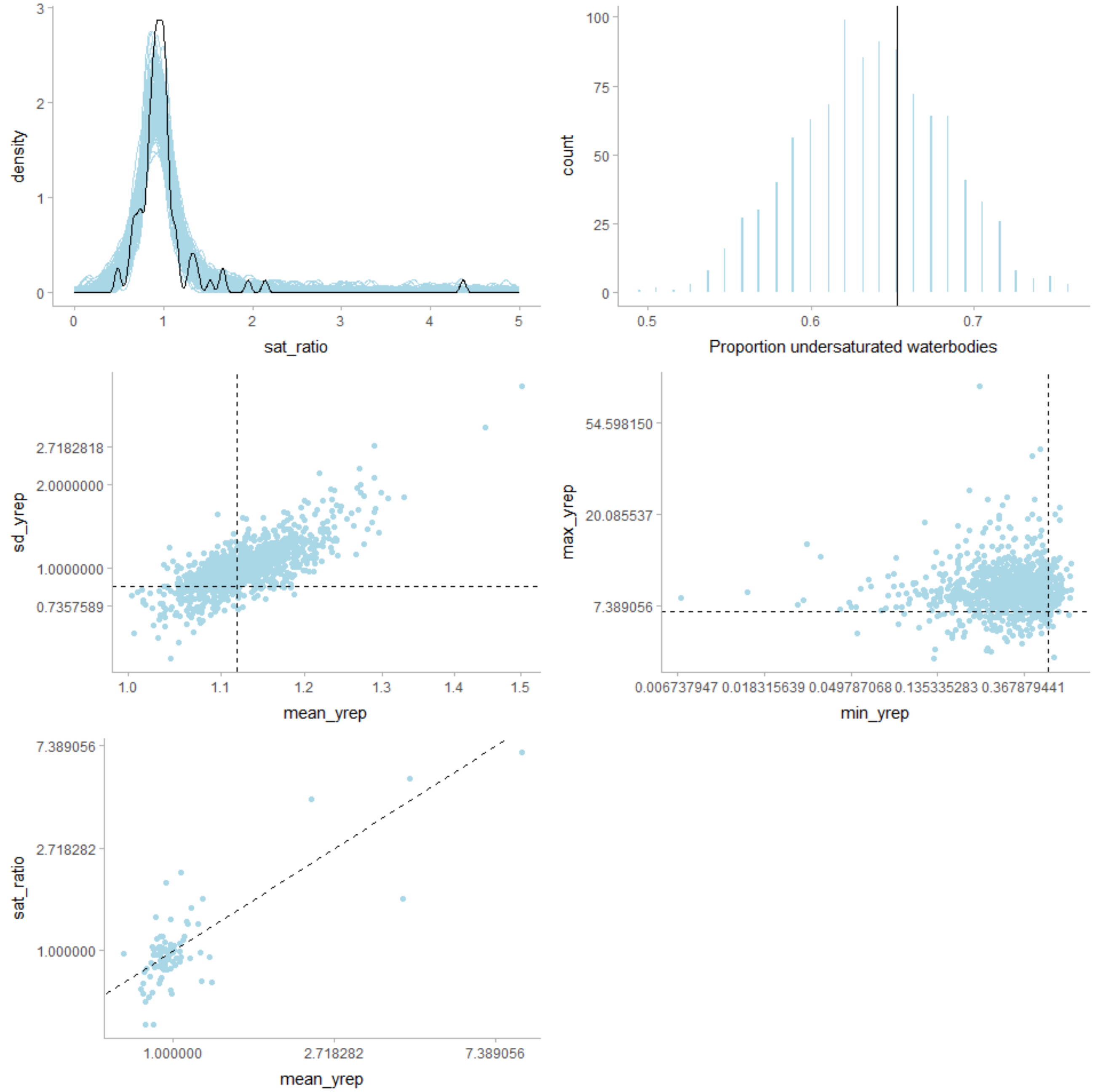

3.3.2.5 R-square

The R^2 estimates for this model are below and suggested substantial improvements over the previous models.

```
##           Estimate Est.Error  Q2.5 Q97.5
## R2logn2o     0.626     0.017 0.591  0.66

##             Estimate Est.Error Q2.5 Q97.5
## R2logn2oeq     0.879     0.004 0.87 0.886
```

Next, R^2 for the 2nd visit sites.

```
##          Estimate Est.Error  Q2.5 Q97.5
## R2logn2o    0.686     0.026 0.625 0.728

##            Estimate Est.Error  Q2.5 Q97.5
## R2logn2oeq    0.875     0.006 0.862 0.887
```

3.3.3 Covariate effects

Below are plots illustrating the modeled effects of covariates on both N2O and equilibrium N2O.

3.3.3.1 Dissolved N2O

The conditional effects plots below for dissolved N2O illustrated a positive, monotonic, and non-linear relationship with NO3; and a negative, linear relationship with surface temperature.

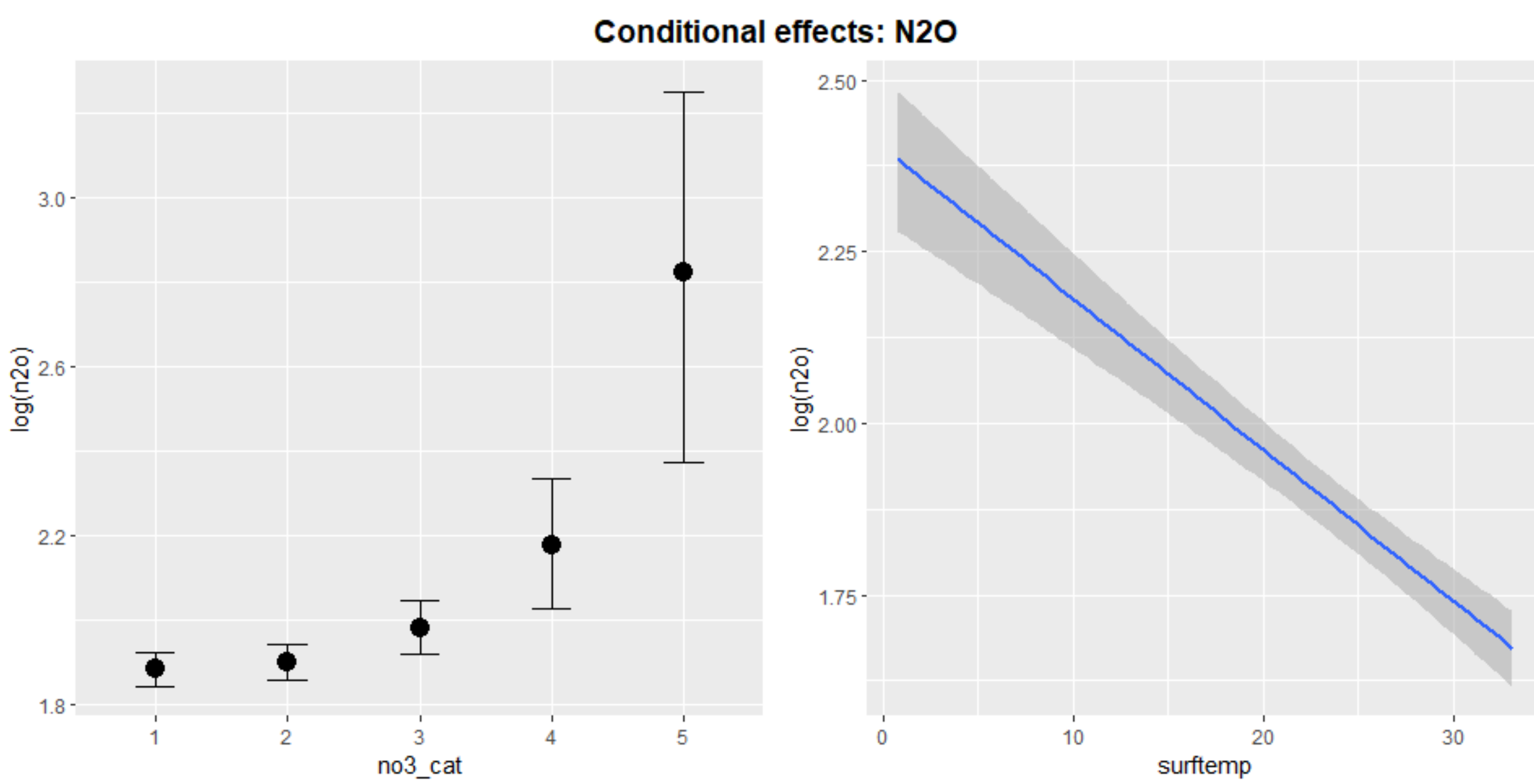

3.3.3.2 Equilibrium N2O

The modeled effects for the equilibrium N2O component illustrated a negative relationship with both predictors and an interaction such that the surface temperature effect became slightly steeper at lower elevations.

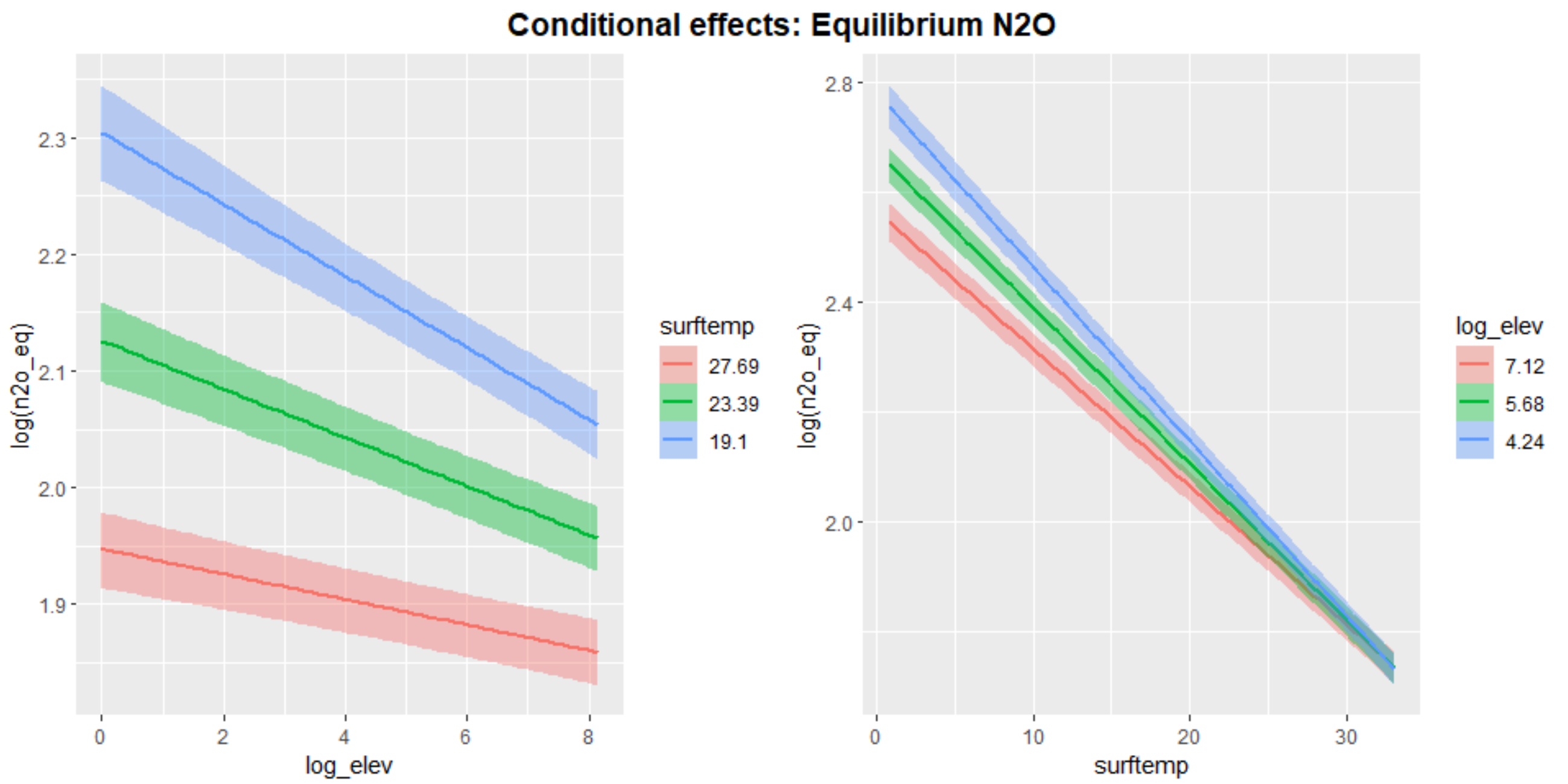

3.4 Model 4

In the next model, covariate terms were also included in the σ components of both models in order to try to better capture remaining heterogeneity in the variances of both N2O and N2O-eq.

```
bf_n2o <- bf(log(n2o) ~ mo(no3_cat) +
               surftemp +
               (mo(no3_cat) | a | WSA9) +
               (mo(no3_cat) | b | WSA9:state) +
               (1 | c | WSA9:state:size_cat),
             sigma ~ mo(no3_cat) +
               surftemp +
               (1 | WSA9) +
               (1 | WSA9:state) +
               (1 | WSA9:state:size_cat),
             family = gaussian())

bf_n2oeq <- bf(log(n2o_eq) ~ surftemp +
                 log_elev +
                 surftemp:log_elev +
                 (1 | a | WSA9) +
                 (1 | b | WSA9:state) +
                 (1 | c | WSA9:state:size_cat),
             sigma ~ surftemp +
               log_elev +
               (1 | WSA9) +
               (1 | WSA9:state) +
               (1 | WSA9:state:size_cat),
```

```
             family = gaussian())

priors <- c(
  prior(normal(2, 1), class = "Intercept", resp = "logn2o"),
  prior(normal(0, 1), class = "b", resp = "logn2o"),
  prior(exponential(2), class = "sd", resp = "logn2o"),
  prior(normal(-1, 2), class = "Intercept", dpar = "sigma", resp = "logn2o"),
  prior(normal(0, 1), class = "b", dpar = "sigma", resp = "logn2o"),
  prior(exponential(2), class = "sd", dpar = "sigma", resp = "logn2o"),
  prior(normal(2, 1), class = "Intercept", resp = "logn2oeq"),
  prior(normal(0, 1), class = "b", resp = "logn2oeq"),
  prior(exponential(2), class = "sd", resp = "logn2oeq"),
  prior(normal(-1, 2), class = "Intercept", dpar = "sigma", resp = "logn2oeq"),
  prior(normal(0, 1), class = "b", dpar = "sigma", resp = "logn2oeq"),
  prior(exponential(2), class = "sd", dpar = "sigma", resp = "logn2oeq"),

  prior(lkj(2), class = "rescor"),
  prior(lkj(2), class = "cor")
  )

n2o_mod4 <- brm(bf_n2o + bf_n2oeq + set_rescor(rescor = TRUE),
                data = df_model,
                prior = priors,
  control = list(adapt_delta = 0.975, max_treedepth = 12),
  #sample_prior = "only",
  save_pars = save_pars(all = TRUE),
  seed = 15851,
  chains=4,
  iter=5000,
  cores=4)

save(n2o_mod4, file = "C:/Users/rmartin/OneDrive - Environmental Protection Agency (E
```

3.4.1 Summarize fit

Below is a summary of the fitted parameters along with some convergence diagnostics.

```
##  Family: MV(gaussian, gaussian)
##   Links: mu = identity; sigma = log
##          mu = identity; sigma = log
## Formula: log(n2o) ~ mo(no3_cat) + surftemp + (mo(no3_cat) | a | WSA9) + (mo(no3_ca
##          sigma ~ mo(no3_cat) + surftemp + (1 | WSA9) + (1 | WSA9:state) + (1 | WSA
##          log(n2o_eq) ~ surftemp + log_elev + surftemp:log_elev + (1 | a | WSA9) +
##          sigma ~ surftemp + log_elev + (1 | WSA9) + (1 | WSA9:state) + (1 | WSA9:s
##    Data: df_model (Number of observations: 984)
##   Draws: 4 chains, each with iter = 5000; warmup = 2500; thin = 1;
##          total post-warmup draws = 10000
```

```
##
## Priors:
## b_logn2o ~ normal(0, 1)
## b_logn2o_sigma ~ normal(0, 1)
## b_logn2oeq ~ normal(0, 1)
## b_logn2oeq_sigma ~ normal(0, 1)
## Intercept_logn2o ~ normal(2, 1)
## Intercept_logn2o_sigma ~ normal(-1, 2)
## Intercept_logn2oeq ~ normal(2, 1)
## Intercept_logn2oeq_sigma ~ normal(-1, 2)
## L ~ lkj_corr_cholesky(2)
## Lrescor ~ lkj_corr_cholesky(2)
## <lower=0> sd_logn2o ~ exponential(2)
## <lower=0> sd_logn2o_sigma ~ exponential(2)
## <lower=0> sd_logn2oeq ~ exponential(2)
## <lower=0> sd_logn2oeq_sigma ~ exponential(2)
## simo_logn2o_mono3_cat1 ~ dirichlet(1)
## simo_logn2o_sigma_mono3_cat1 ~ dirichlet(1)
##
## Multilevel Hyperparameters:
## ~WSA9 (Number of levels: 9)
##                                          Estimate Est.Error l-95% CI u-95% CI
## sd(logn2o_Intercept)                        0.050     0.020    0.020    0.100
## sd(logn2o_mono3_cat)                        0.145     0.054    0.065    0.272
## sd(logn2oeq_Intercept)                      0.036     0.012    0.019    0.065
## sd(sigma_logn2o_Intercept)                  0.113     0.080    0.005    0.303
## sd(sigma_logn2oeq_Intercept)                0.208     0.098    0.039    0.435
## cor(logn2o_Intercept,logn2o_mono3_cat)     -0.186     0.327   -0.755    0.489
## cor(logn2o_Intercept,logn2oeq_Intercept)    0.344     0.290   -0.277    0.817
## cor(logn2o_mono3_cat,logn2oeq_Intercept)    0.364     0.286   -0.269    0.819
##                                           Rhat Bulk_ESS Tail_ESS
## sd(logn2o_Intercept)                     1.000     4733     4348
## sd(logn2o_mono3_cat)                     1.000     4386     6208
## sd(logn2oeq_Intercept)                   1.001     3977     5215
## sd(sigma_logn2o_Intercept)               1.001     2543     4693
## sd(sigma_logn2oeq_Intercept)             1.001     2777     2177
## cor(logn2o_Intercept,logn2o_mono3_cat)   1.000     4616     6092
## cor(logn2o_Intercept,logn2oeq_Intercept) 1.000     5667     6074
## cor(logn2o_mono3_cat,logn2oeq_Intercept) 1.001     7634     7624
##
## ~WSA9:state (Number of levels: 96)
##                                          Estimate Est.Error l-95% CI u-95% CI
## sd(logn2o_Intercept)                        0.035     0.017    0.003    0.068
## sd(logn2o_mono3_cat)                        0.117     0.022    0.076    0.162
## sd(logn2oeq_Intercept)                      0.033     0.003    0.027    0.040
## sd(sigma_logn2o_Intercept)                  0.181     0.099    0.011    0.374
## sd(sigma_logn2oeq_Intercept)                0.287     0.057    0.177    0.403
## cor(logn2o_Intercept,logn2o_mono3_cat)     -0.317     0.311   -0.810    0.405
## cor(logn2o_Intercept,logn2oeq_Intercept)    0.025     0.265   -0.497    0.557
```

```
## cor(logn2o_mono3_cat,logn2oeq_Intercept)    0.448     0.165    0.098    0.738
##                                           Rhat Bulk_ESS Tail_ESS
## sd(logn2o_Intercept)                     1.001      981     2017
## sd(logn2o_mono3_cat)                     1.001     3149     4612
## sd(logn2oeq_Intercept)                   1.000     3457     6360
## sd(sigma_logn2o_Intercept)               1.004      858     2373
## sd(sigma_logn2oeq_Intercept)             1.001     2470     3862
## cor(logn2o_Intercept,logn2o_mono3_cat)   1.003      945     1286
## cor(logn2o_Intercept,logn2oeq_Intercept) 1.005      556      823
## cor(logn2o_mono3_cat,logn2oeq_Intercept) 1.001     1518     3021
##
## ~WSA9:state:size_cat (Number of levels: 352)
##                                          Estimate Est.Error l-95% CI u-95% CI
## sd(logn2o_Intercept)                        0.065     0.011    0.042    0.087
## sd(logn2oeq_Intercept)                      0.004     0.002    0.000    0.008
## sd(sigma_logn2o_Intercept)                  0.539     0.055    0.432    0.647
## sd(sigma_logn2oeq_Intercept)                0.263     0.051    0.162    0.363
## cor(logn2o_Intercept,logn2oeq_Intercept)    0.395     0.348   -0.479    0.904
##                                           Rhat Bulk_ESS Tail_ESS
## sd(logn2o_Intercept)                     1.003     1478     2832
## sd(logn2oeq_Intercept)                   1.001     1506     2453
## sd(sigma_logn2o_Intercept)               1.002     1443     3611
## sd(sigma_logn2oeq_Intercept)             1.001     2372     4366
## cor(logn2o_Intercept,logn2oeq_Intercept) 1.001     4231     4956
##
## Regression Coefficients:
##                            Estimate Est.Error l-95% CI u-95% CI  Rhat Bulk_ESS
## logn2o_Intercept              2.386     0.055    2.278    2.490 1.000     5971
## sigma_logn2o_Intercept       -1.855     0.281   -2.389   -1.294 1.000     5965
## logn2oeq_Intercept            3.115     0.051    3.016    3.217 1.001     7109
## sigma_logn2oeq_Intercept     -1.922     0.373   -2.634   -1.180 1.000     8445
## logn2o_surftemp              -0.021     0.002   -0.026   -0.017 1.000     5850
## sigma_logn2o_surftemp        -0.001     0.011   -0.023    0.021 1.001     6175
## logn2oeq_surftemp            -0.042     0.002   -0.046   -0.039 1.001     8285
## logn2oeq_log_elev            -0.080     0.008   -0.096   -0.065 1.001     7656
## logn2oeq_surftemp:log_elev    0.002     0.000    0.002    0.003 1.000     8407
## sigma_logn2oeq_surftemp      -0.065     0.010   -0.085   -0.046 1.000    10043
## sigma_logn2oeq_log_elev      -0.019     0.038   -0.095    0.054 1.000     6646
## logn2o_mono3_cat              0.225     0.058    0.108    0.340 1.000     4488
## sigma_logn2o_mono3_cat        0.256     0.037    0.187    0.331 1.000     6036
##                            Tail_ESS
## logn2o_Intercept               7057
## sigma_logn2o_Intercept         7704
## logn2oeq_Intercept             7366
## sigma_logn2oeq_Intercept       7581
## logn2o_surftemp                7677
## sigma_logn2o_surftemp          7768
## logn2oeq_surftemp              7516
## logn2oeq_log_elev              7564
```

```
## logn2oeq_surftemp:log_elev     7502
## sigma_logn2oeq_surftemp        8163
## sigma_logn2oeq_log_elev        7910
## logn2o_mono3_cat               5143
## sigma_logn2o_mono3_cat         7643
## 
## Monotonic Simplex Parameters:
##                            Estimate Est.Error l-95% CI u-95% CI  Rhat Bulk_ESS
## logn2o_mono3_cat1[1]          0.018     0.012    0.001    0.045 1.000     5709
## logn2o_mono3_cat1[2]          0.093     0.027    0.041    0.149 1.000     5034
## logn2o_mono3_cat1[3]          0.231     0.060    0.126    0.366 1.000     4974
## logn2o_mono3_cat1[4]          0.659     0.062    0.520    0.763 1.000     4446
## sigma_logn2o_mono3_cat1[1]    0.107     0.066    0.007    0.255 1.000     6168
## sigma_logn2o_mono3_cat1[2]    0.129     0.087    0.006    0.328 1.000     8105
## sigma_logn2o_mono3_cat1[3]    0.459     0.151    0.168    0.758 1.000     7272
## sigma_logn2o_mono3_cat1[4]    0.304     0.139    0.038    0.569 1.000     6289
##                            Tail_ESS
## logn2o_mono3_cat1[1]           4429
## logn2o_mono3_cat1[2]           5645
## logn2o_mono3_cat1[3]           5478
## logn2o_mono3_cat1[4]           5210
## sigma_logn2o_mono3_cat1[1]     4471
## sigma_logn2o_mono3_cat1[2]     5010
## sigma_logn2o_mono3_cat1[3]     5802
## sigma_logn2o_mono3_cat1[4]     4264
## 
## Residual Correlations: 
##                         Estimate Est.Error l-95% CI u-95% CI  Rhat Bulk_ESS
## rescor(logn2o,logn2oeq)    0.146     0.040    0.067    0.223 1.001     9839
##                         Tail_ESS
## rescor(logn2o,logn2oeq)     8508
## 
## Draws were sampled using sampling(NUTS). For each parameter, Bulk_ESS
## and Tail_ESS are effective sample size measures, and Rhat is the potential
## scale reduction factor on split chains (at convergence, Rhat = 1).
```

3.4.2 Model checks

The same PPCs were employed for this model as above.

3.4.2.1 Dissolved N2O

This model appeared to be a moderate improvement on the previous models.

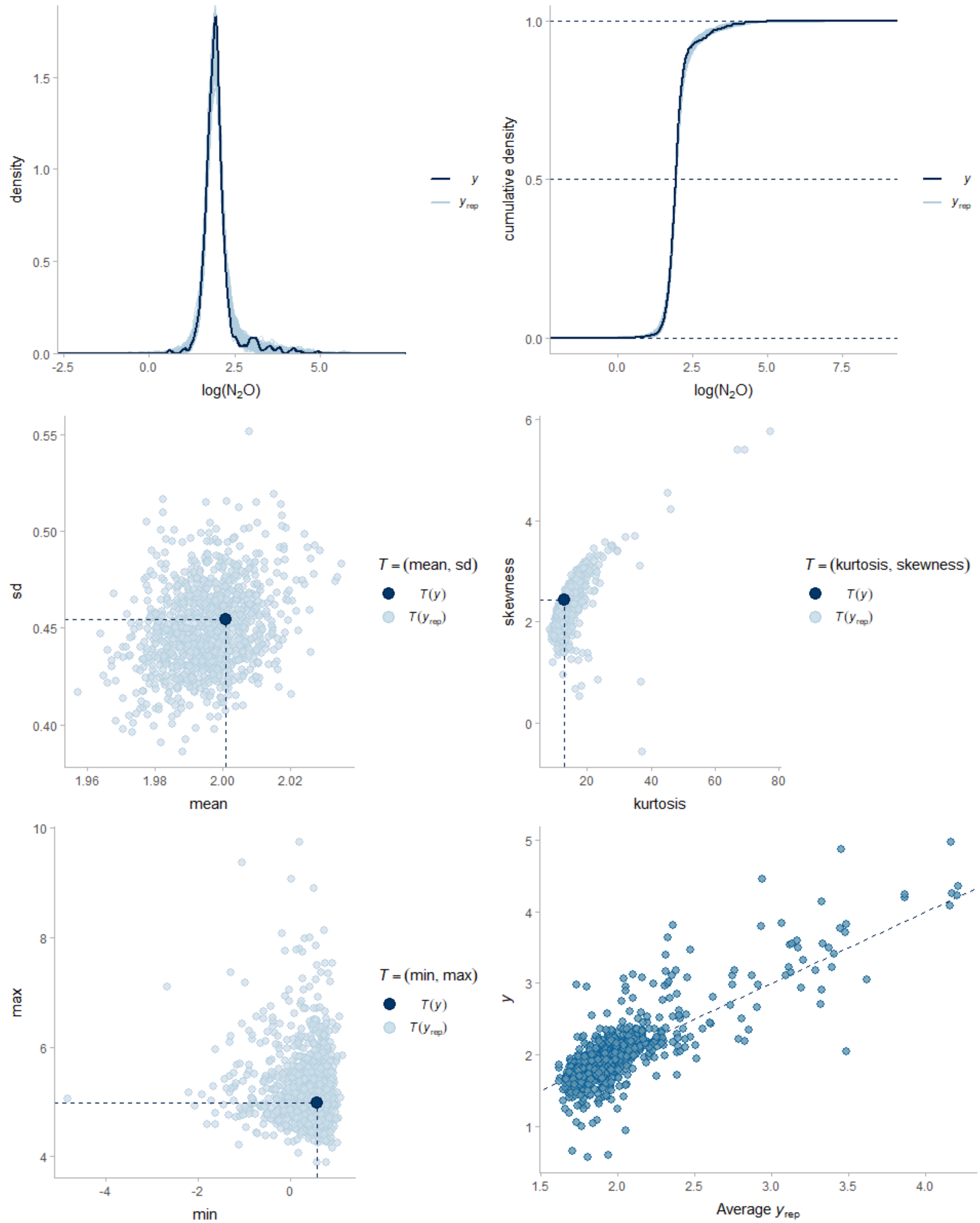

This same checks for the 2nd visit sites is below.

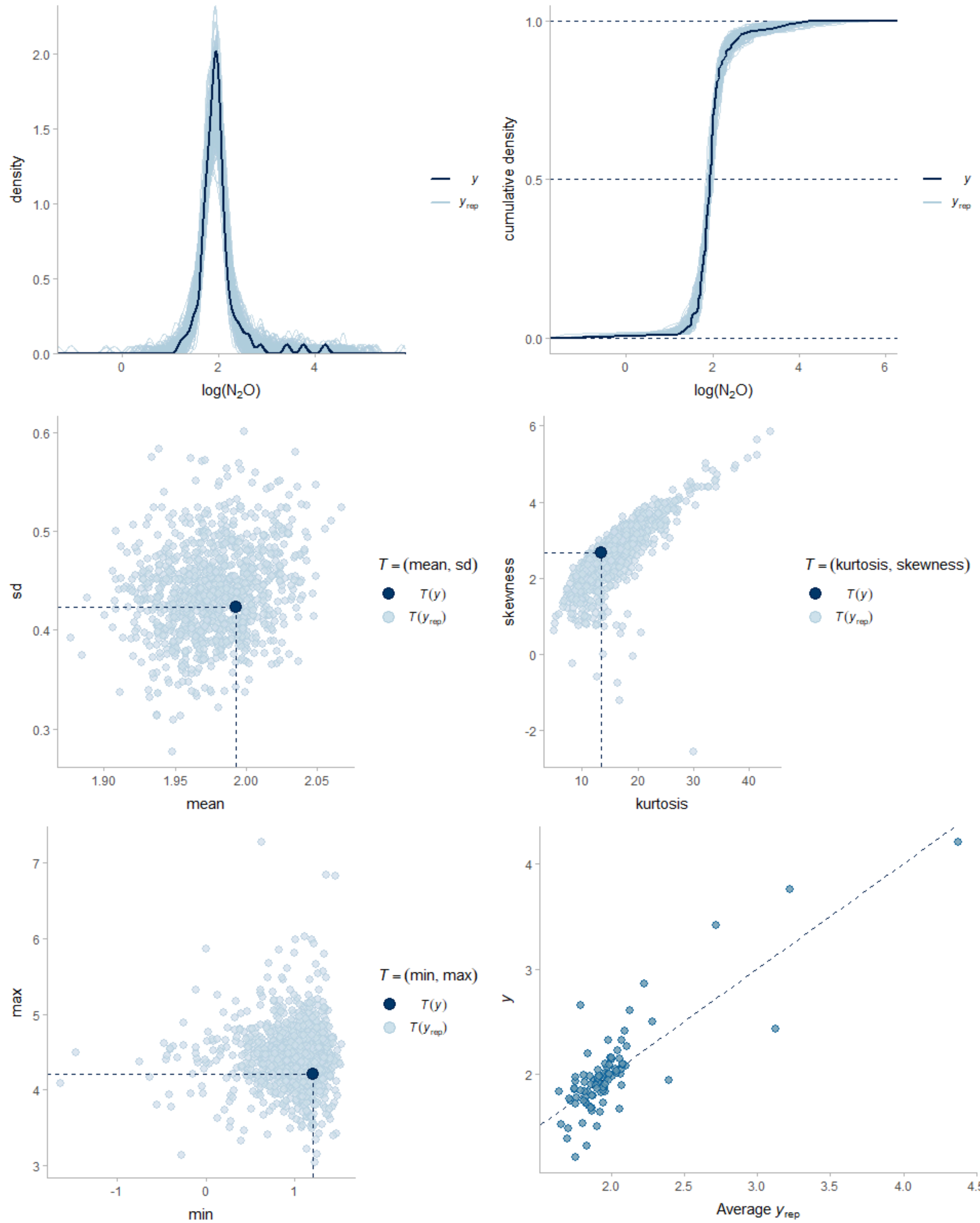

3.4.2.2 Equilibrium N2O

This component of the model also seemed to be an improvement over model 3, with better representation in the tails as indicated in the skewness *vs.* kurtosis PPC.

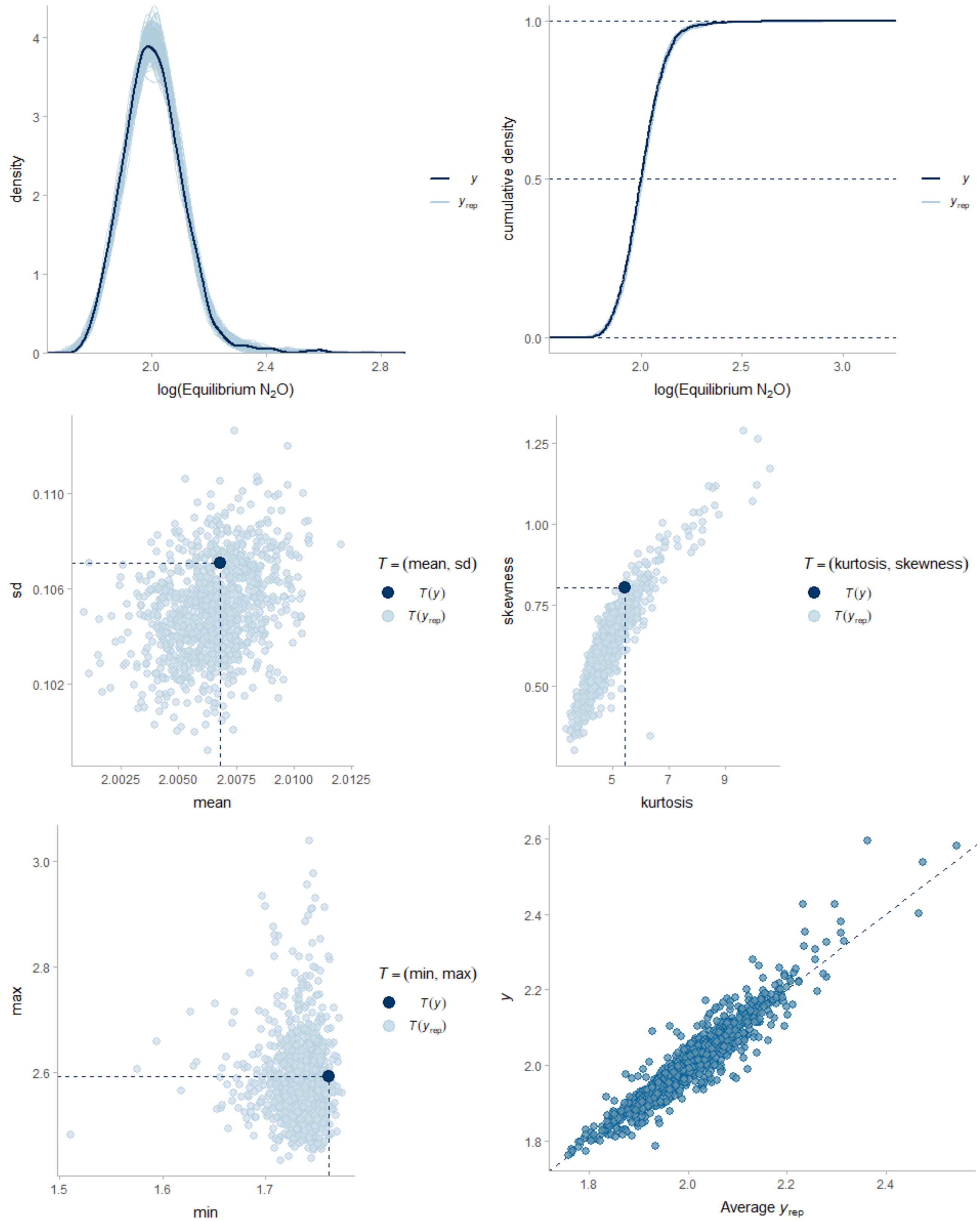

For the 2nd visit sites.

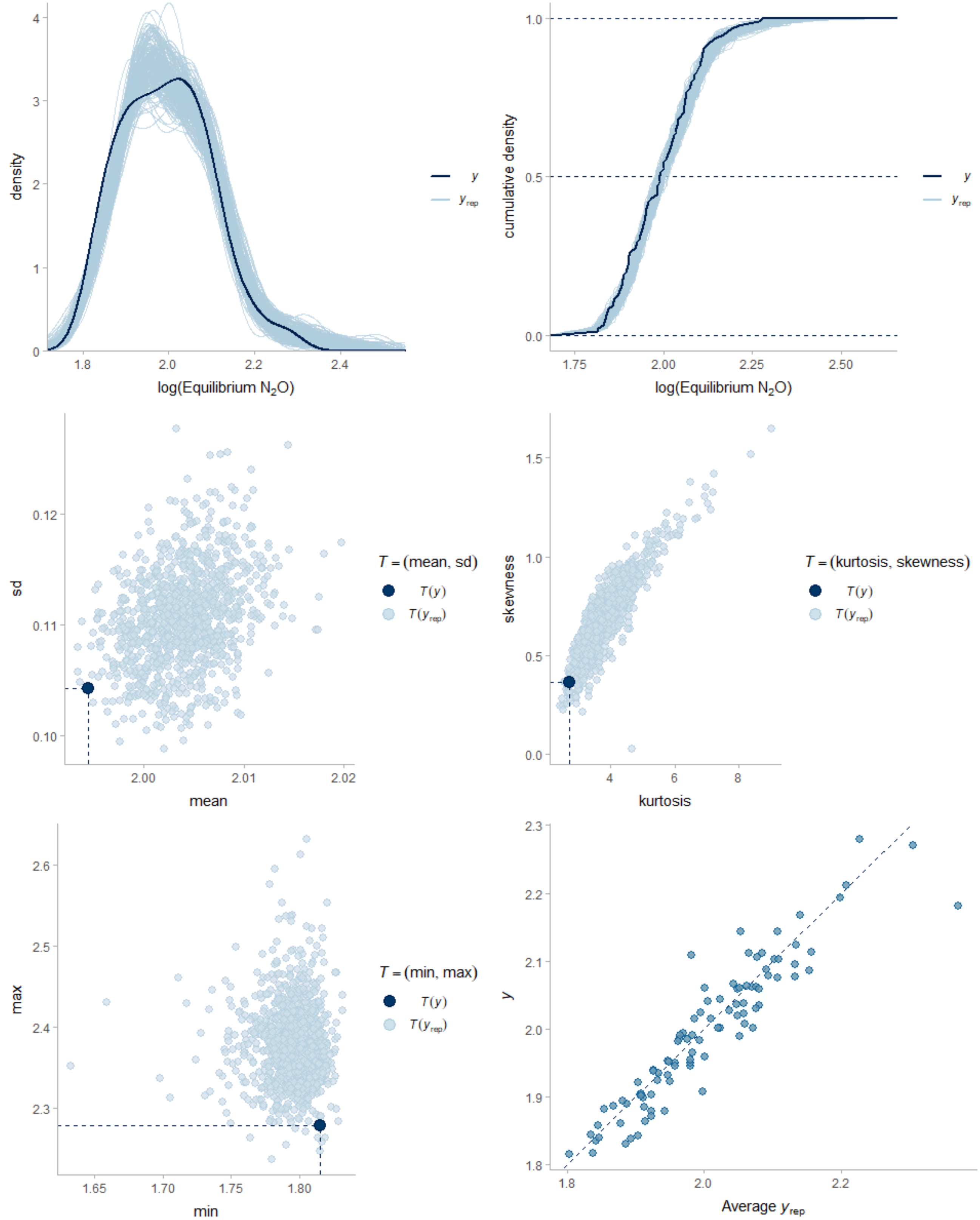

3.4.2.3 Bivariate

Again, an improvement over the previous model with a tighter fit of the PPC to the observed bivariate density.

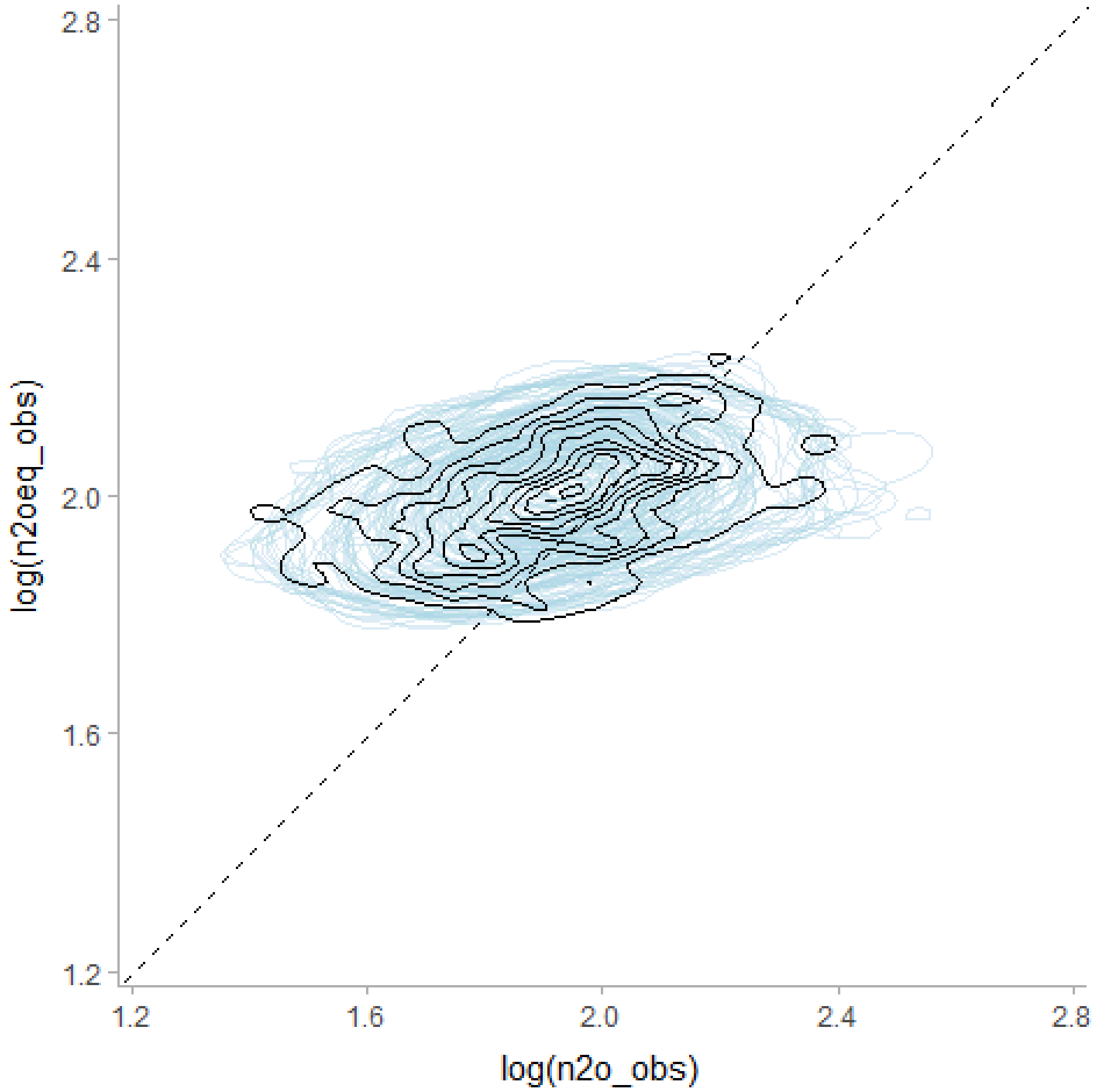

For the 2nd visit sites.

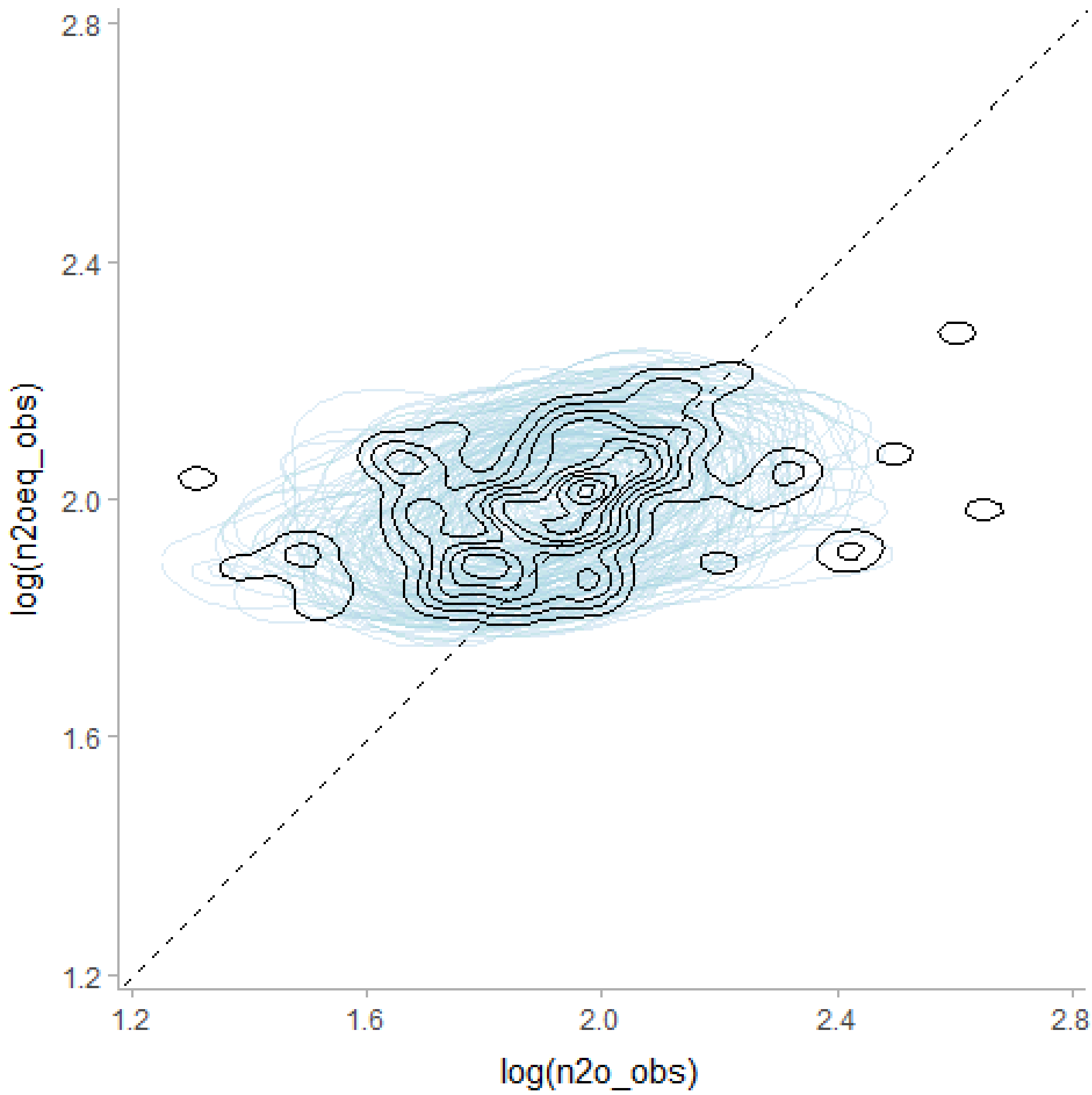

3.4.2.4 Saturation

This check also suggested an improvement over the previous models, with better tail behavior and less bias in the proportion under-saturated measure.

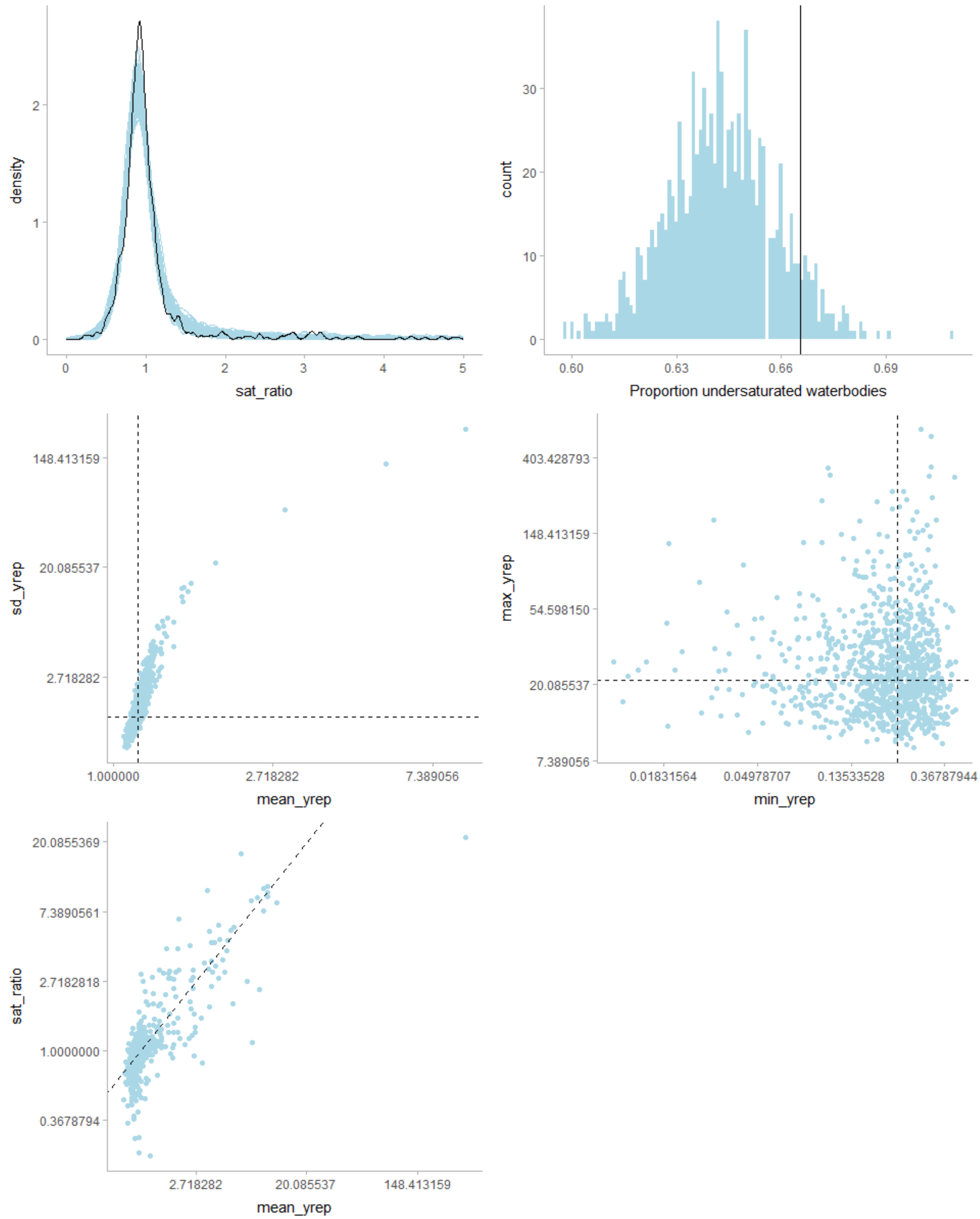

For the second visit sites:

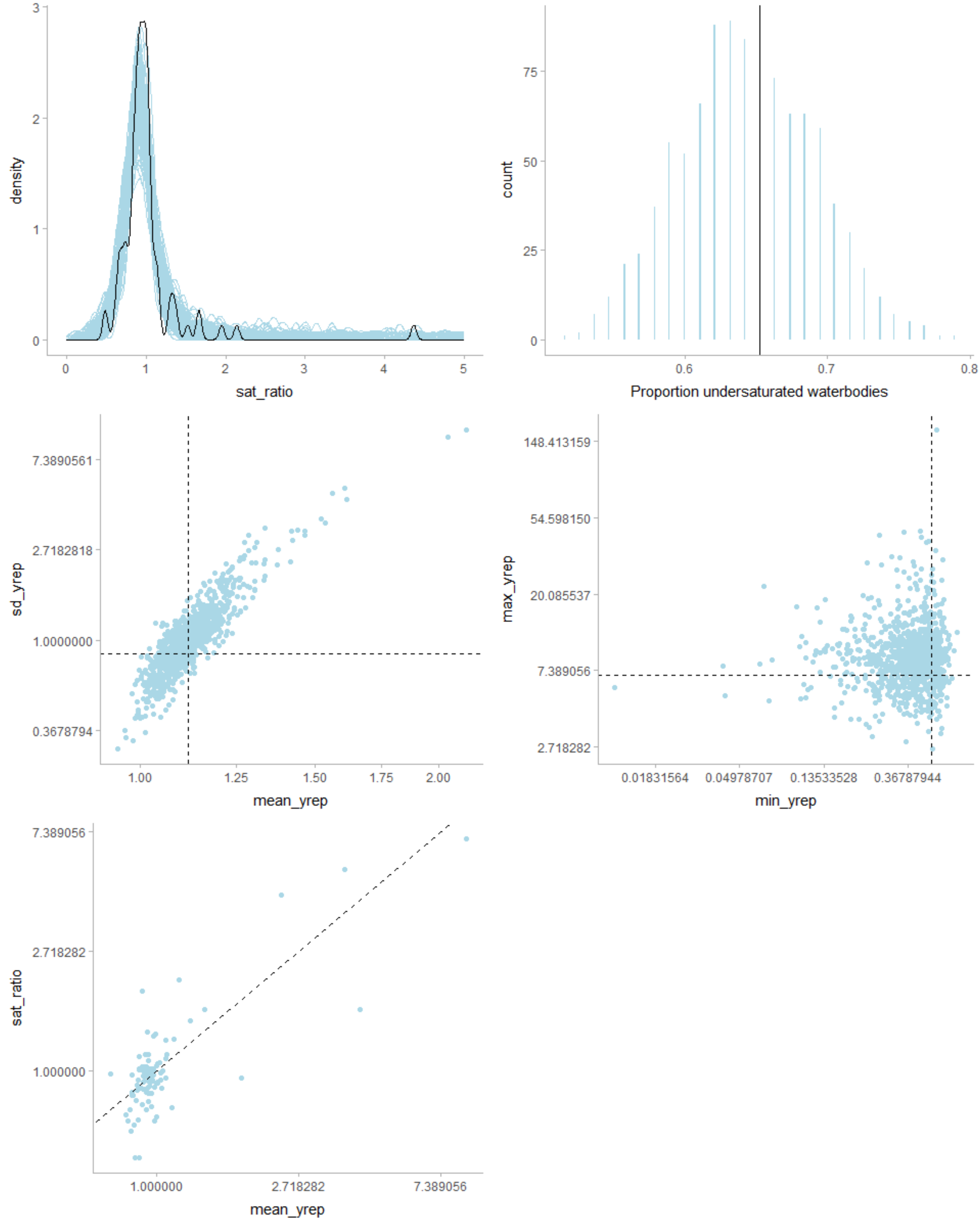

3.4.2.5 R-square

The Bayesian R^2 estimates below indicated an improvement from the previous models.

```
##           Estimate Est.Error  Q2.5 Q97.5
## R2logn2o     0.606     0.025 0.551 0.651
```

```
##             Estimate Est.Error  Q2.5 Q97.5
## R2logn2oeq    0.875     0.005 0.865 0.883
```

For the 2nd visit data.

```
##           Estimate Est.Error  Q2.5 Q97.5
## R2logn2o    0.661     0.043 0.564 0.731

##             Estimate Est.Error  Q2.5 Q97.5
## R2logn2oeq     0.87     0.007 0.855 0.883
```

3.4.3 Covariate effects

3.4.3.1 Dissolved N2O

The conditional effects plots for the covariate effects on dissolved N2O remained similar to the previous model.

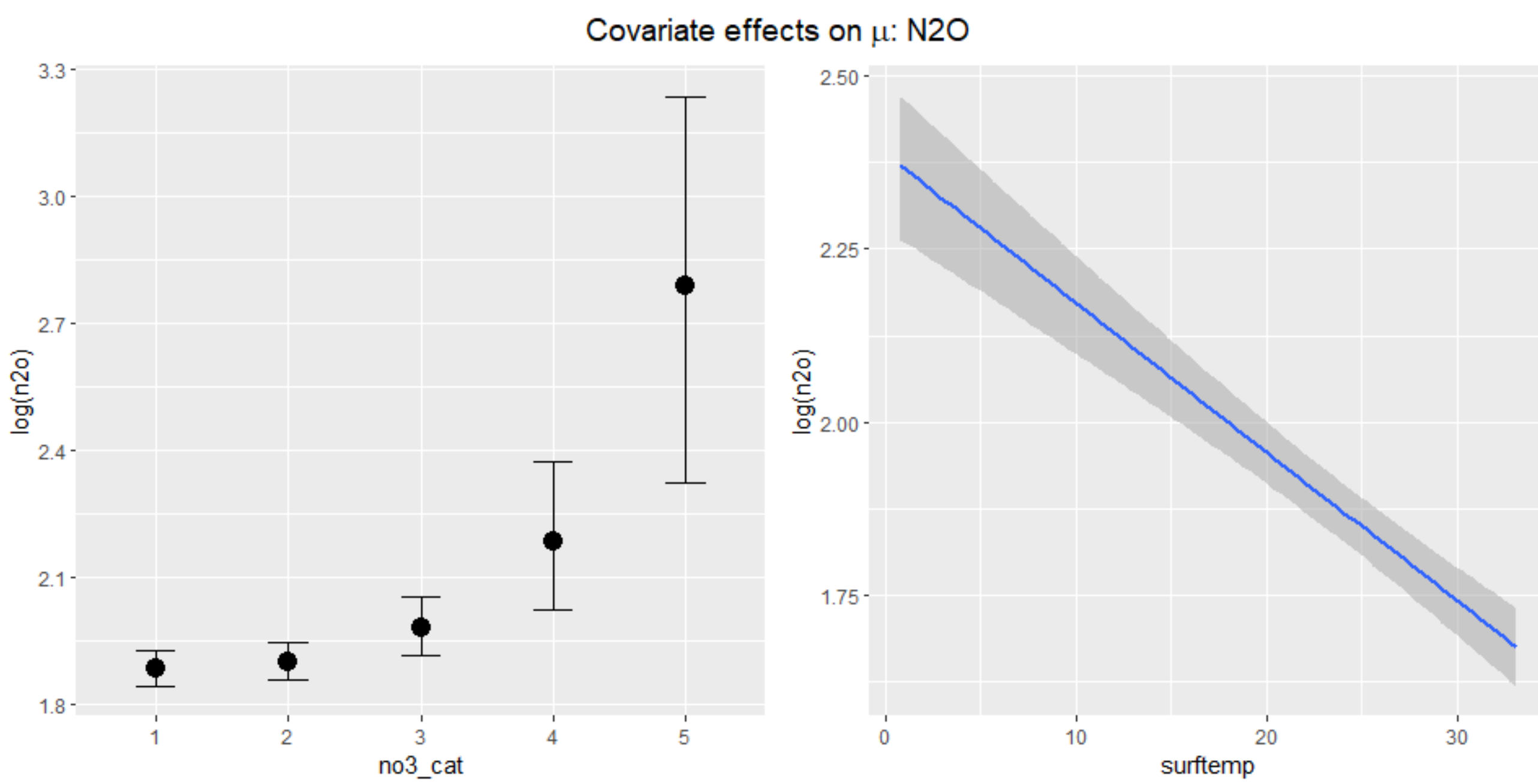

Below are estimates of the conditional effects of the covariates on σ for N2O. These plots suggested a large effect of NO3 on the variance of N2O, but little to no effect of surface temperature.

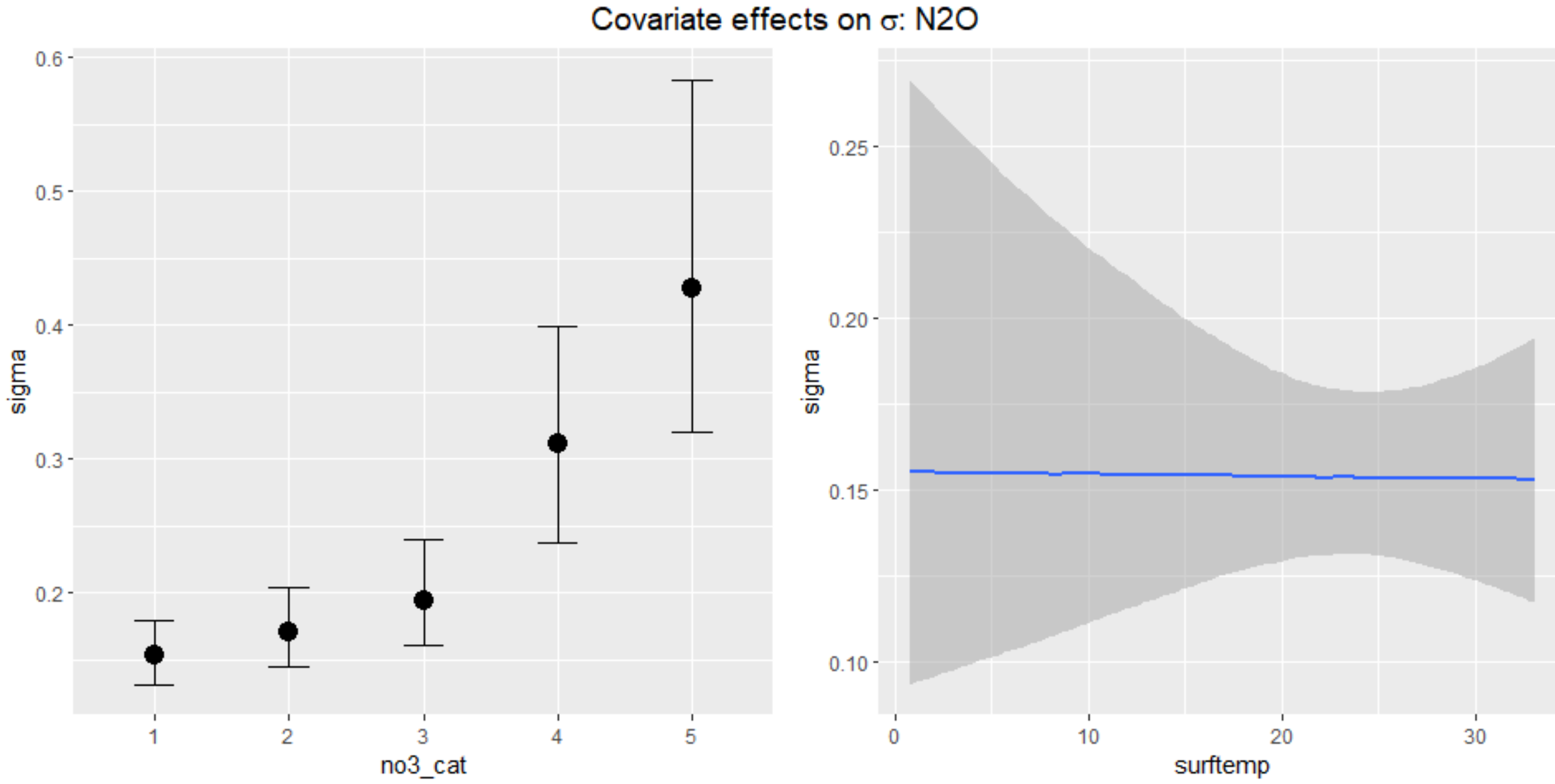

3.4.3.2 Equilibrium N2O

The covariate effects on equilibrium N2O remained largely the same as for the previous model.

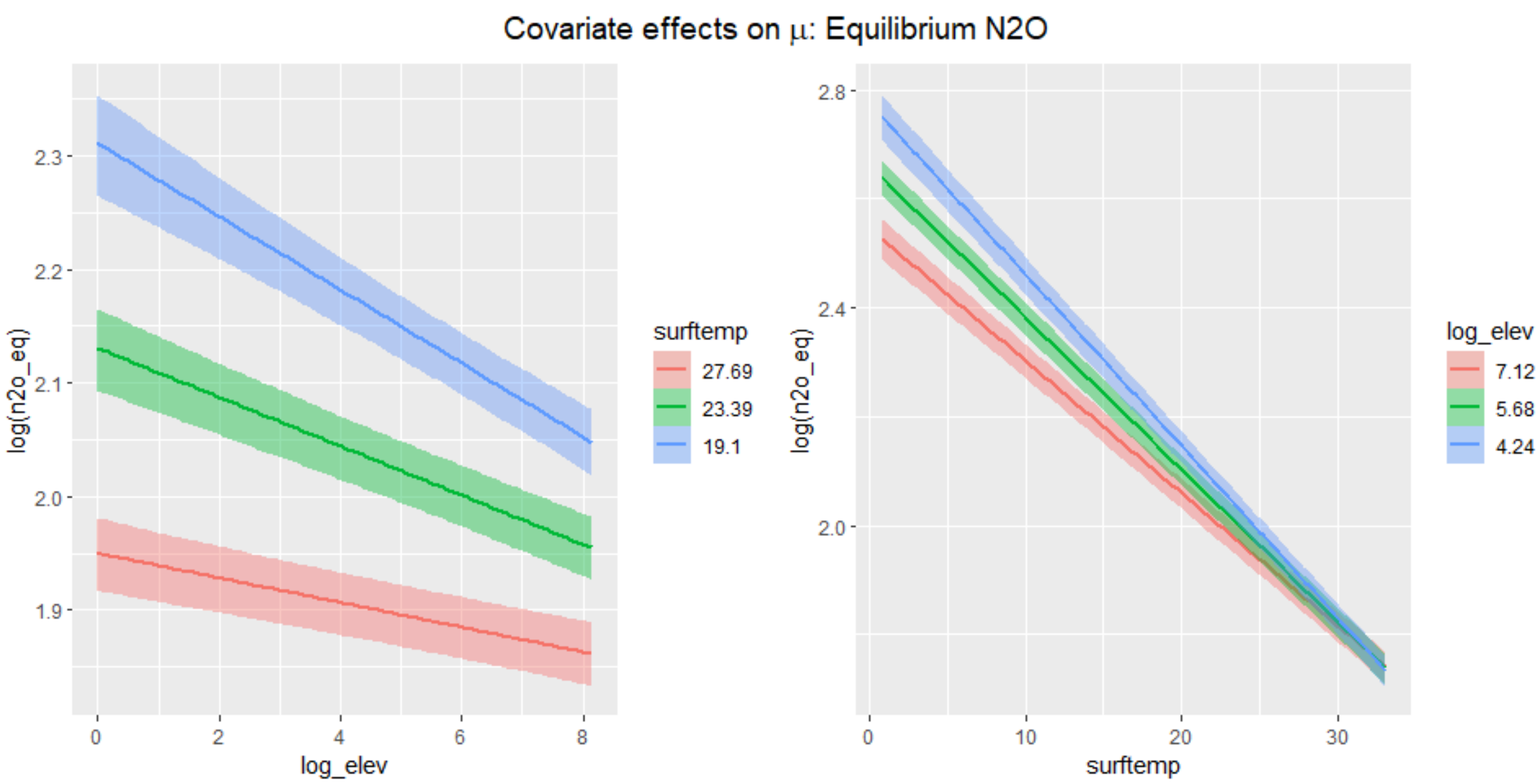

The covariate effects on σ for N2O-eq suggested a negative effect of surface temperature and litte to no effect of elevation.

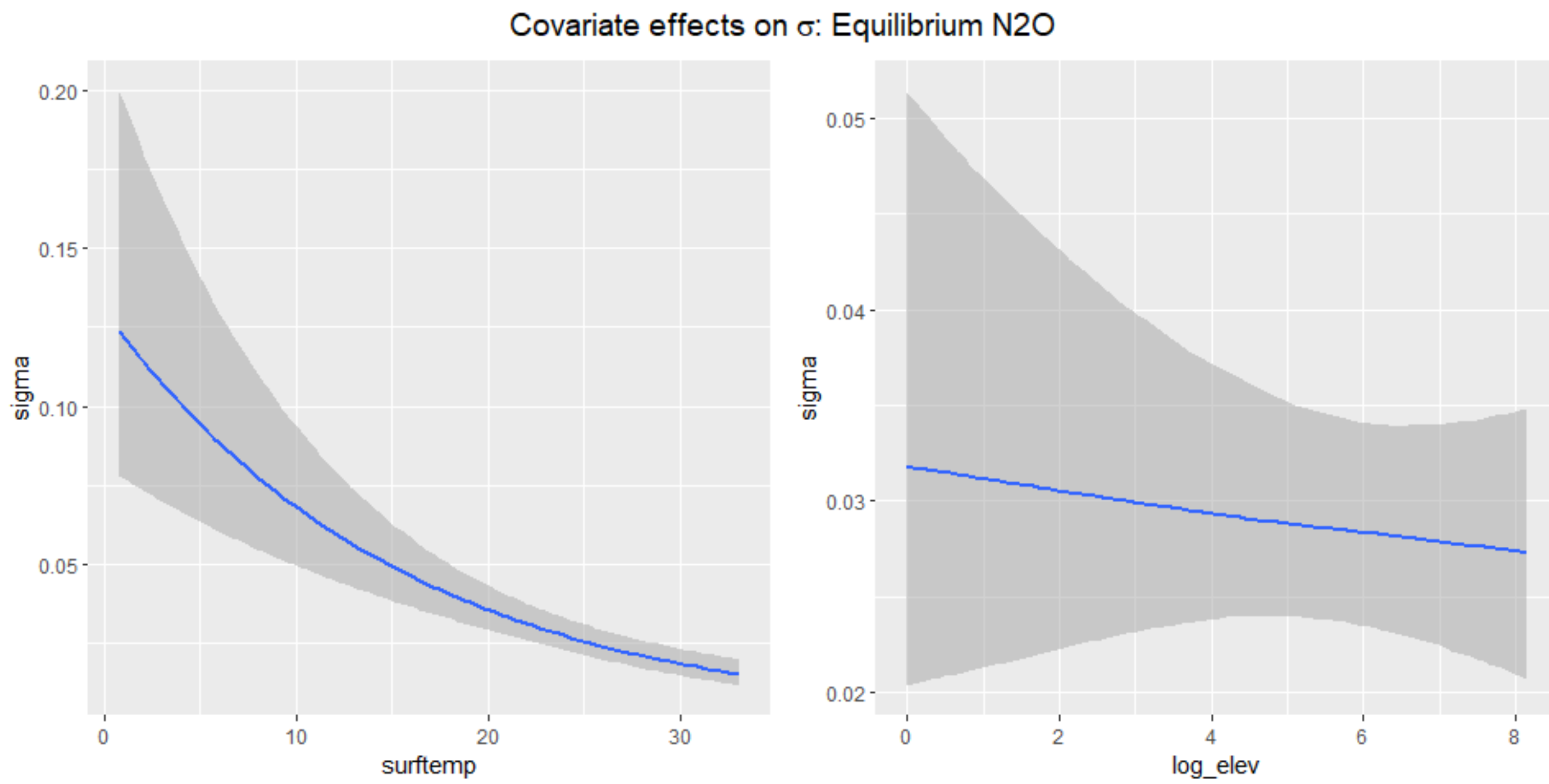

3.5 Model 5

In the next model, additional complexity is added to the dissolved N2O component by including a covariate for continuous lake surface area (log scale) as well as interactions between NO3 and log(surface area) and surface temperature. Although some variation due to lake size was likely captured by the categorical survey variable for size in the models above, the categorical distinction may not well represent the true continuous nature of waterbody size and its effects on the response. For example, the largest size category represented all lakes >50 hecatares, which included an extremely wide range of waterbody sizes ranging from 50 to over 300,000 hectares at max. Including the continuous size covariate at the observation level in this manner was expected to do a better job of resolving trends with size. Colinearity between the grouped and observation-level measures were not a major, concern, since the observation-level measure would account for any variation first. Though not presented in this document, violations of the Gauss-Markov assumptions were checked by looking for potential correlations between model errors and the covariates (Bafumi and Gelman 2007).

```
bf_n2o <- bf(log(n2o) ~ mo(no3_cat) +
               log_area +
               surftemp +
               mo(no3_cat):log_area +
               mo(no3_cat):surftemp +
               (mo(no3_cat) | a | WSA9) +
               (mo(no3_cat) | b | WSA9:state) +
               (1 | c | WSA9:state:size_cat),
             sigma ~ log_area +
```

```
                 mo(no3_cat) +
                 (1 | WSA9) +
                 (1 | WSA9:state) +
                 (1 | WSA9:state:size_cat),
               family = gaussian())

bf_n2oeq <- bf(log(n2o_eq) ~ surftemp +
                   log_elev +
                   surftemp:log_elev +
                   (1 | a | WSA9) +
                   (1 | b | WSA9:state) +
                   (1 | c | WSA9:state:size_cat),
               sigma ~ surftemp +
                 log_elev +
                 (1 | WSA9) +
                 (1 | WSA9:state) +
                 (1 | WSA9:state:size_cat),
               family = gaussian())

priors <- c(
  prior(normal(2, 1), class = "Intercept", resp = "logn2o"),
  prior(normal(0, 1), class = "b", resp = "logn2o"),
  prior(exponential(2), class = "sd", resp = "logn2o"),
  prior(normal(-1, 2), class = "Intercept", dpar = "sigma", resp = "logn2o"),
  prior(normal(0, 1), class = "b", dpar = "sigma", resp = "logn2o"),
  prior(exponential(2), class = "sd", dpar = "sigma", resp = "logn2o"),

  prior(normal(2, 1), class = "Intercept", resp = "logn2oeq"),
  prior(normal(0, 1), class = "b", resp = "logn2oeq"),
  prior(exponential(2), class = "sd", resp = "logn2oeq"),
  prior(normal(-1, 2), class = "Intercept", dpar = "sigma", resp = "logn2oeq"),
  prior(normal(0, 1), class = "b", dpar = "sigma", resp = "logn2oeq"),
  prior(exponential(2), class = "sd", dpar = "sigma", resp = "logn2oeq"),

  prior(lkj(2), class = "rescor"),
  prior(lkj(2), class = "cor")
  )

n2o_mod5 <- brm(bf_n2o +
                    bf_n2oeq +
                    set_rescor(rescor = TRUE),
                data = df_model,
                prior = priors,
  control = list(adapt_delta = 0.975, max_treedepth = 12),
  #sample_prior = "only",
  save_pars = save_pars(all = TRUE),
  seed = 54741,
  chains=4,
  iter=5000,
```

```
  cores=4)

save(n2o_mod5, file = "C:/Users/rmartin/OneDrive - Environmental Protection Agency (E
```

3.5.1 Summarize fit

Below is a summary of the fitted parameters along with MCMC convergence diagnostics.

```
##  Family: MV(gaussian, gaussian)
##   Links: mu = identity; sigma = log
##          mu = identity; sigma = log
## Formula: log(n2o) ~ mo(no3_cat) + log_area + surftemp + mo(no3_cat):log_area + mo(
##          sigma ~ log_area + mo(no3_cat) + (1 | WSA9) + (1 | WSA9:state) + (1 | WSA
##          log(n2o_eq) ~ surftemp + log_elev + surftemp:log_elev + (1 | a | WSA9) +
##          sigma ~ surftemp + log_elev + (1 | WSA9) + (1 | WSA9:state) + (1 | WSA9:s
##    Data: df_model (Number of observations: 984)
##   Draws: 4 chains, each with iter = 5000; warmup = 2500; thin = 1;
##          total post-warmup draws = 10000
##
## Priors:
## b_logn2o ~ normal(0, 1)
## b_logn2o_sigma ~ normal(0, 1)
## b_logn2oeq ~ normal(0, 1)
## b_logn2oeq_sigma ~ normal(0, 1)
## Intercept_logn2o ~ normal(2, 1)
## Intercept_logn2o_sigma ~ normal(-1, 2)
## Intercept_logn2oeq ~ normal(2, 1)
## Intercept_logn2oeq_sigma ~ normal(-1, 2)
## L ~ lkj_corr_cholesky(2)
## Lrescor ~ lkj_corr_cholesky(2)
## <lower=0> sd_logn2o ~ exponential(2)
## <lower=0> sd_logn2o_sigma ~ exponential(2)
## <lower=0> sd_logn2oeq ~ exponential(2)
## <lower=0> sd_logn2oeq_sigma ~ exponential(2)
## simo_logn2o_mono3_cat:log_area1 ~ dirichlet(1)
## simo_logn2o_mono3_cat:surftemp1 ~ dirichlet(1)
## simo_logn2o_mono3_cat1 ~ dirichlet(1)
## simo_logn2o_sigma_mono3_cat1 ~ dirichlet(1)
##
## Multilevel Hyperparameters:
## ~WSA9 (Number of levels: 9)
##                                           Estimate Est.Error l-95% CI u-95% CI
## sd(logn2o_Intercept)                         0.048     0.019    0.020    0.094
## sd(logn2o_mono3_cat)                         0.081     0.050    0.007    0.199
## sd(logn2oeq_Intercept)                       0.034     0.011    0.018    0.062
## sd(sigma_logn2o_Intercept)                   0.111     0.076    0.006    0.293
## sd(sigma_logn2oeq_Intercept)                 0.209     0.102    0.036    0.445
```

```
## cor(logn2o_Intercept,logn2o_mono3_cat)     -0.056     0.360   -0.713    0.644
## cor(logn2o_Intercept,logn2oeq_Intercept)    0.464     0.284   -0.173    0.885
## cor(logn2o_mono3_cat,logn2oeq_Intercept)    0.259     0.341   -0.467    0.824
##                                           Rhat Bulk_ESS Tail_ESS
## sd(logn2o_Intercept)                     1.000     4413     4624
## sd(logn2o_mono3_cat)                     1.003     1412     2265
## sd(logn2oeq_Intercept)                   1.000     4061     6633
## sd(sigma_logn2o_Intercept)               1.000     2790     4064
## sd(sigma_logn2oeq_Intercept)             1.001     2176     1898
## cor(logn2o_Intercept,logn2o_mono3_cat)   1.000     5823     6525
## cor(logn2o_Intercept,logn2oeq_Intercept) 1.000     4935     6214
## cor(logn2o_mono3_cat,logn2oeq_Intercept) 1.000     4684     4770
##
## ~WSA9:state (Number of levels: 96)
##                                          Estimate Est.Error l-95% CI u-95% CI
## sd(logn2o_Intercept)                        0.046     0.014    0.016    0.072
## sd(logn2o_mono3_cat)                        0.097     0.024    0.053    0.146
## sd(logn2oeq_Intercept)                      0.033     0.003    0.027    0.040
## sd(sigma_logn2o_Intercept)                  0.207     0.088    0.024    0.369
## sd(sigma_logn2oeq_Intercept)                0.285     0.056    0.177    0.395
## cor(logn2o_Intercept,logn2o_mono3_cat)     -0.265     0.284   -0.762    0.336
## cor(logn2o_Intercept,logn2oeq_Intercept)    0.163     0.200   -0.232    0.559
## cor(logn2o_mono3_cat,logn2oeq_Intercept)    0.266     0.209   -0.155    0.649
##                                           Rhat Bulk_ESS Tail_ESS
## sd(logn2o_Intercept)                     1.001     1323     1765
## sd(logn2o_mono3_cat)                     1.001     2612     2569
## sd(logn2oeq_Intercept)                   1.001     3321     5286
## sd(sigma_logn2o_Intercept)               1.007      776     1082
## sd(sigma_logn2oeq_Intercept)             1.002     2464     3702
## cor(logn2o_Intercept,logn2o_mono3_cat)   1.001     1708     3060
## cor(logn2o_Intercept,logn2oeq_Intercept) 1.005      960     1715
## cor(logn2o_mono3_cat,logn2oeq_Intercept) 1.002      883     1843
##
## ~WSA9:state:size_cat (Number of levels: 352)
##                                          Estimate Est.Error l-95% CI u-95% CI
## sd(logn2o_Intercept)                        0.035     0.015    0.004    0.062
## sd(logn2oeq_Intercept)                      0.003     0.002    0.000    0.007
## sd(sigma_logn2o_Intercept)                  0.479     0.056    0.374    0.591
## sd(sigma_logn2oeq_Intercept)                0.260     0.052    0.160    0.360
## cor(logn2o_Intercept,logn2oeq_Intercept)    0.235     0.413   -0.658    0.872
##                                           Rhat Bulk_ESS Tail_ESS
## sd(logn2o_Intercept)                     1.004      819     1375
## sd(logn2oeq_Intercept)                   1.003     1569     3553
## sd(sigma_logn2o_Intercept)               1.006     1318     3346
## sd(sigma_logn2oeq_Intercept)             1.001     2326     3796
## cor(logn2o_Intercept,logn2oeq_Intercept) 1.002     2950     5425
##
## Regression Coefficients:
##                            Estimate Est.Error l-95% CI u-95% CI  Rhat Bulk_ESS
```

```
## logn2o_Intercept              2.380     0.055    2.273    2.487 1.001     4020
## sigma_logn2o_Intercept       -1.596     0.097   -1.789   -1.408 1.002     4961
## logn2oeq_Intercept            3.116     0.051    3.019    3.218 1.000     6860
## sigma_logn2oeq_Intercept     -1.922     0.374   -2.639   -1.179 1.000     7865
## logn2o_log_area               0.029     0.003    0.023    0.034 1.000     9054
## logn2o_surftemp              -0.025     0.002   -0.029   -0.021 1.000     3996
## sigma_logn2o_log_area        -0.095     0.020   -0.135   -0.055 1.001     6068
## logn2oeq_surftemp            -0.042     0.002   -0.046   -0.039 1.000     7418
## logn2oeq_log_elev            -0.080     0.008   -0.097   -0.065 1.000     6834
## logn2oeq_surftemp:log_elev    0.003     0.000    0.002    0.003 1.000     7339
## sigma_logn2oeq_surftemp      -0.065     0.010   -0.085   -0.046 1.000     9671
## sigma_logn2oeq_log_elev      -0.018     0.038   -0.096    0.054 1.000     6265
## logn2o_mono3_cat              0.026     0.127   -0.226    0.275 1.004     1971
## logn2o_mono3_cat:log_area    -0.036     0.010   -0.054   -0.016 1.001     2602
## logn2o_mono3_cat:surftemp     0.014     0.006    0.003    0.026 1.004     1478
## sigma_logn2o_mono3_cat        0.246     0.036    0.179    0.321 1.001     4246
##                            Tail_ESS
## logn2o_Intercept               6632
## sigma_logn2o_Intercept         6145
## logn2oeq_Intercept             7546
## sigma_logn2oeq_Intercept       7774
## logn2o_log_area                8090
## logn2o_surftemp                7106
## sigma_logn2o_log_area          7491
## logn2oeq_surftemp              8290
## logn2oeq_log_elev              7865
## logn2oeq_surftemp:log_elev     7992
## sigma_logn2oeq_surftemp        8132
## sigma_logn2oeq_log_elev        7038
## logn2o_mono3_cat               3252
## logn2o_mono3_cat:log_area      4549
## logn2o_mono3_cat:surftemp      2416
## sigma_logn2o_mono3_cat         6738
##
## Monotonic Simplex Parameters:
##                               Estimate Est.Error l-95% CI u-95% CI  Rhat
## logn2o_mono3_cat1[1]             0.026     0.024    0.001    0.089 1.001
## logn2o_mono3_cat1[2]             0.091     0.066    0.005    0.252 1.001
## logn2o_mono3_cat1[3]             0.250     0.146    0.034    0.615 1.002
## logn2o_mono3_cat1[4]             0.633     0.166    0.206    0.867 1.003
## logn2o_mono3_cat:log_area1[1]    0.060     0.041    0.005    0.161 1.000
## logn2o_mono3_cat:log_area1[2]    0.042     0.039    0.001    0.142 1.000
## logn2o_mono3_cat:log_area1[3]    0.297     0.161    0.047    0.681 1.000
## logn2o_mono3_cat:log_area1[4]    0.602     0.176    0.166    0.865 1.000
## logn2o_mono3_cat:surftemp1[1]    0.043     0.040    0.006    0.144 1.002
## logn2o_mono3_cat:surftemp1[2]    0.080     0.056    0.015    0.222 1.001
## logn2o_mono3_cat:surftemp1[3]    0.276     0.111    0.105    0.573 1.001
## logn2o_mono3_cat:surftemp1[4]    0.601     0.142    0.168    0.785 1.002
## sigma_logn2o_mono3_cat1[1]       0.131     0.074    0.010    0.288 1.000
```

```
## sigma_logn2o_mono3_cat1[2]       0.151     0.096    0.010    0.367 1.000
## sigma_logn2o_mono3_cat1[3]       0.444     0.149    0.149    0.734 1.001
## sigma_logn2o_mono3_cat1[4]       0.275     0.138    0.026    0.544 1.001
##                               Bulk_ESS Tail_ESS
## logn2o_mono3_cat1[1]              4261     5305
## logn2o_mono3_cat1[2]              1875     3916
## logn2o_mono3_cat1[3]              2027     2856
## logn2o_mono3_cat1[4]              1411     2439
## logn2o_mono3_cat:log_area1[1]     4395     3509
## logn2o_mono3_cat:log_area1[2]     7129     6046
## logn2o_mono3_cat:log_area1[3]     5220     5683
## logn2o_mono3_cat:log_area1[4]     4431     4505
## logn2o_mono3_cat:surftemp1[1]     3541     3002
## logn2o_mono3_cat:surftemp1[2]     3667     2580
## logn2o_mono3_cat:surftemp1[3]     3634     3352
## logn2o_mono3_cat:surftemp1[4]     2731     2289
## sigma_logn2o_mono3_cat1[1]        6840     4269
## sigma_logn2o_mono3_cat1[2]        7630     5795
## sigma_logn2o_mono3_cat1[3]        6331     6964
## sigma_logn2o_mono3_cat1[4]        4902     5797
##
## Residual Correlations:
##                         Estimate Est.Error l-95% CI u-95% CI  Rhat Bulk_ESS
## rescor(logn2o,logn2oeq)    0.141     0.038    0.066    0.216 1.000    11829
##                         Tail_ESS
## rescor(logn2o,logn2oeq)     8204
##
## Draws were sampled using sampling(NUTS). For each parameter, Bulk_ESS
## and Tail_ESS are effective sample size measures, and Rhat is the potential
## scale reduction factor on split chains (at convergence, Rhat = 1).
```

3.5.2 Model checks

Again, the same PPCs as above were performed for this model.

3.5.2.1 Dissolved N2O

The PPC for dissolved N2O below suggested minimal improvements upon the the previous model.

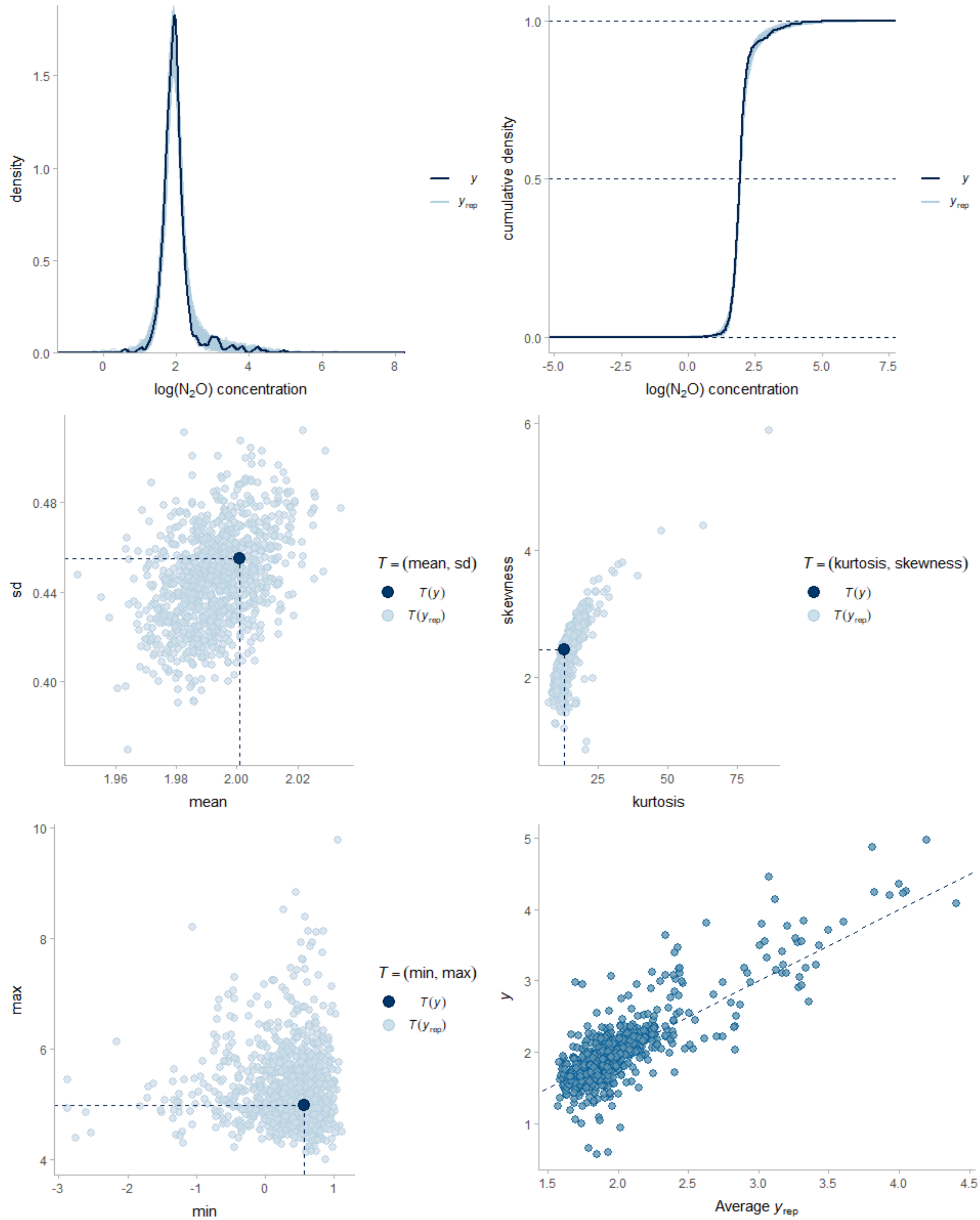

The same check for the 2nd visit data is below.

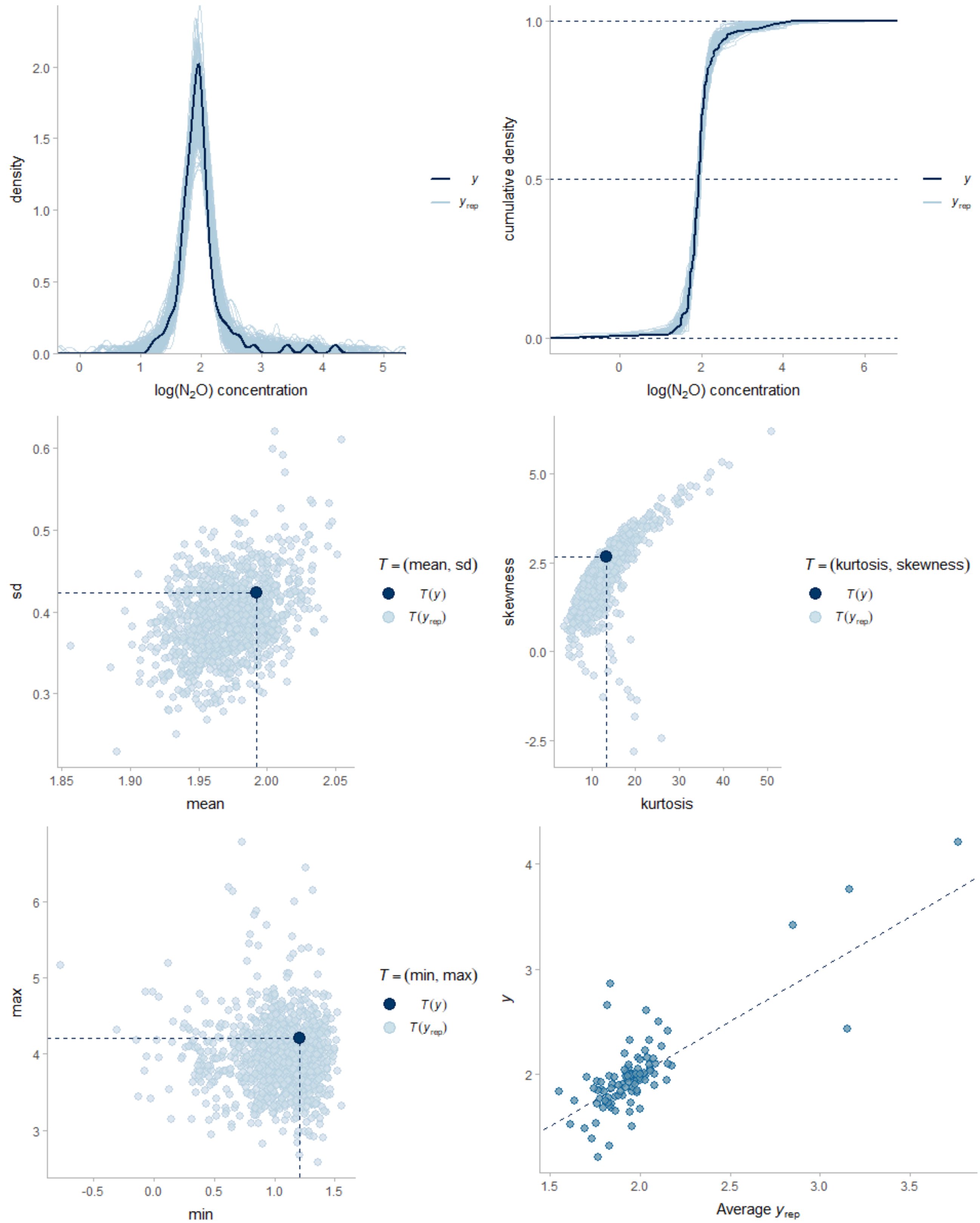

3.5.2.2 Equilibrium N2O

The PPCs for this model were similar to the previous model, which was unsurprising given that it was the same model for N2O-eq.

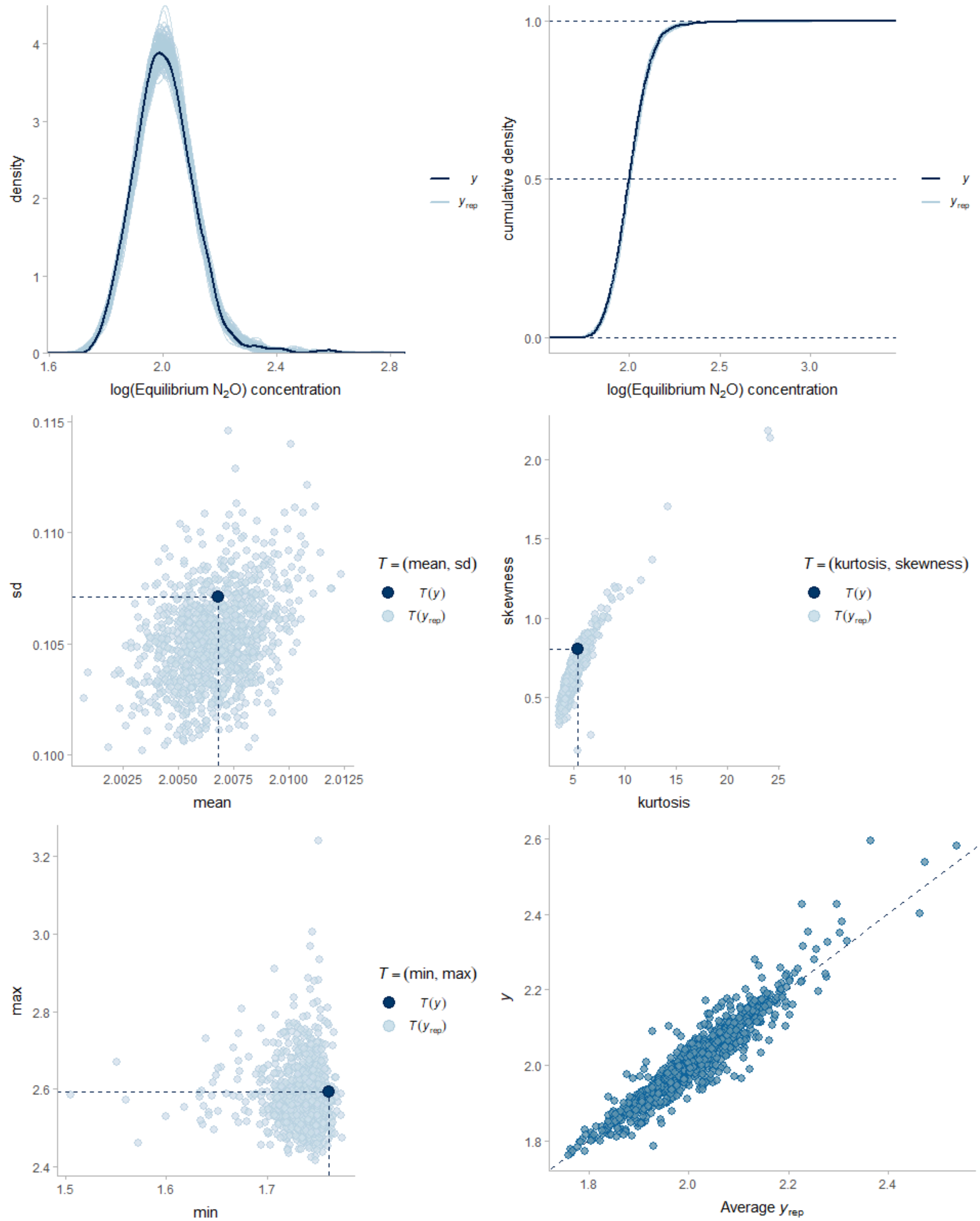

Below, is the same check using the 2nd visit data.

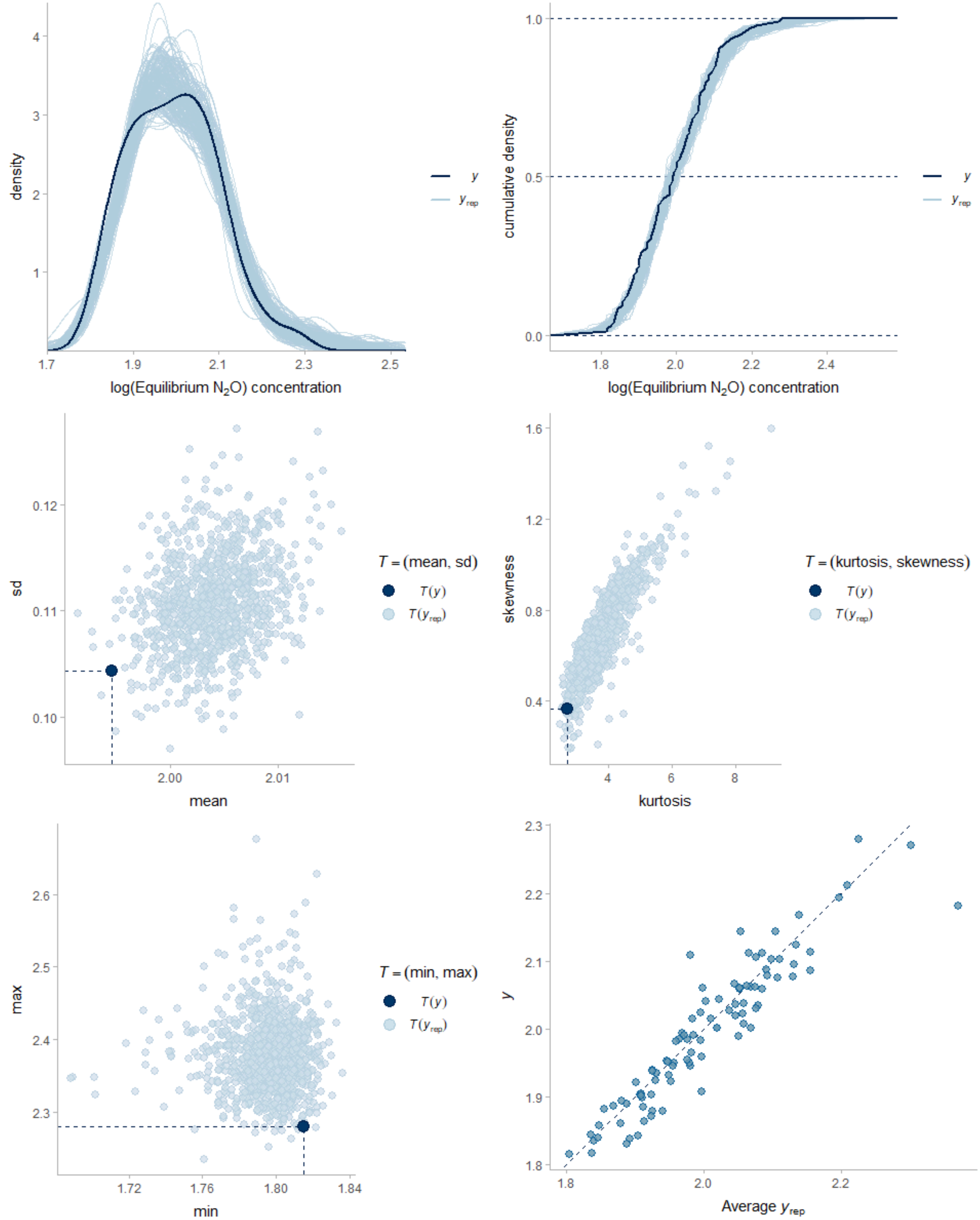

3.5.2.3 Bivariate

This PPC was also similar to the previous model.

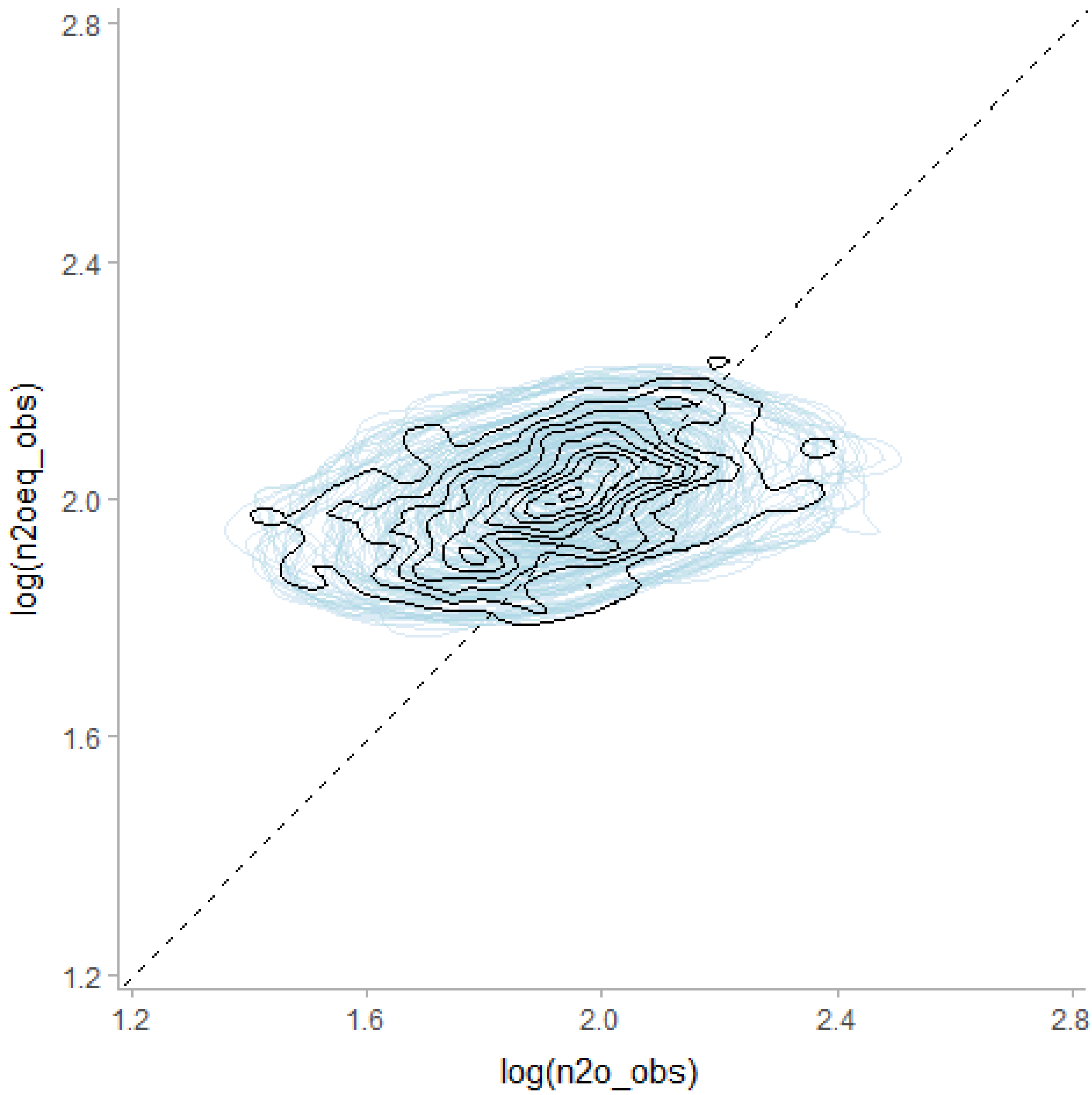

And the check with the 2nd visit data.

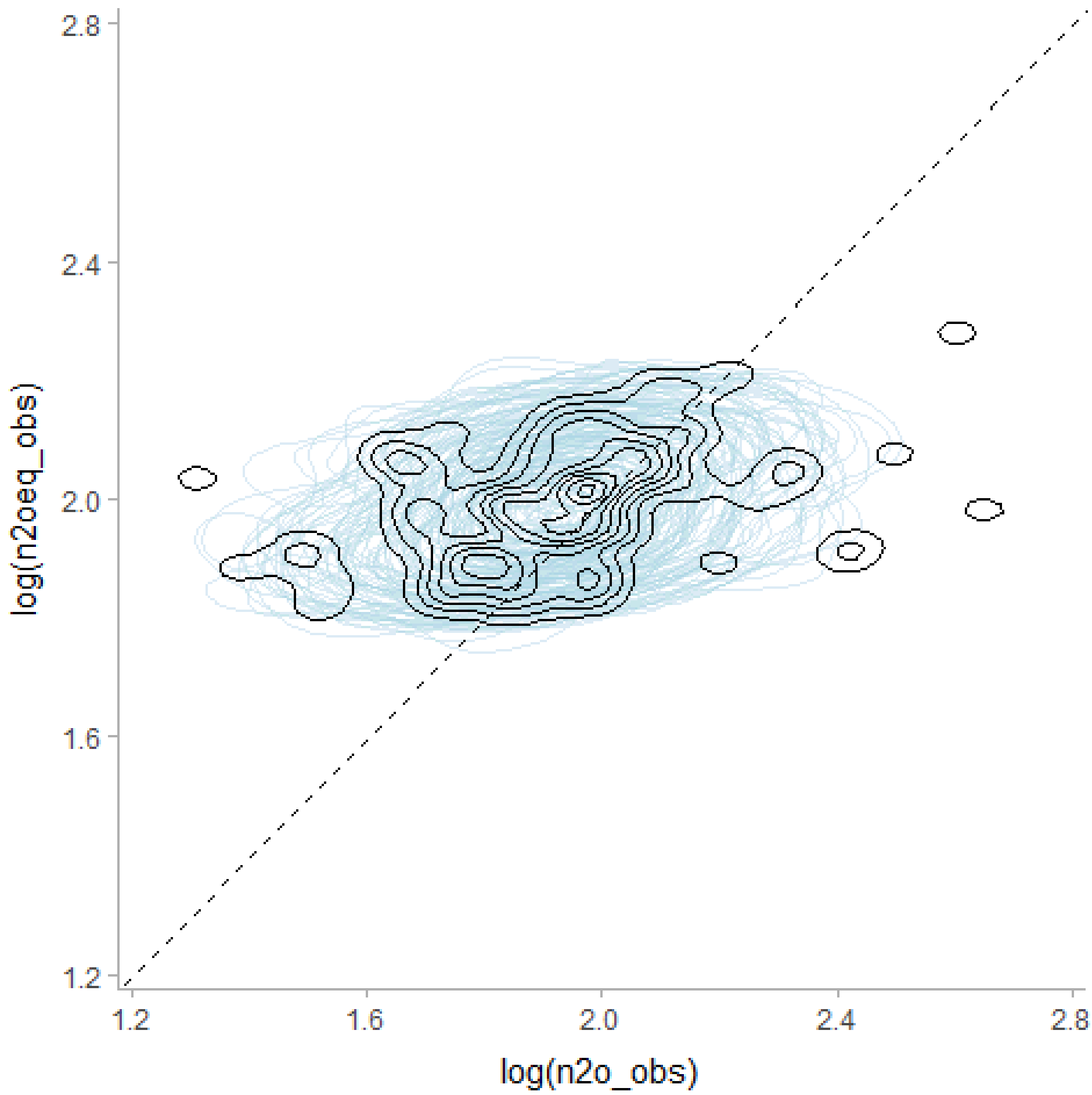

3.5.2.4 Saturation

This check was also similar to the prevoius model, with perhaps slightly less bias in the proportion unsaturated estimates. There was also a potentially concerning extreme prediction in the observed *vs* predicted PPC.

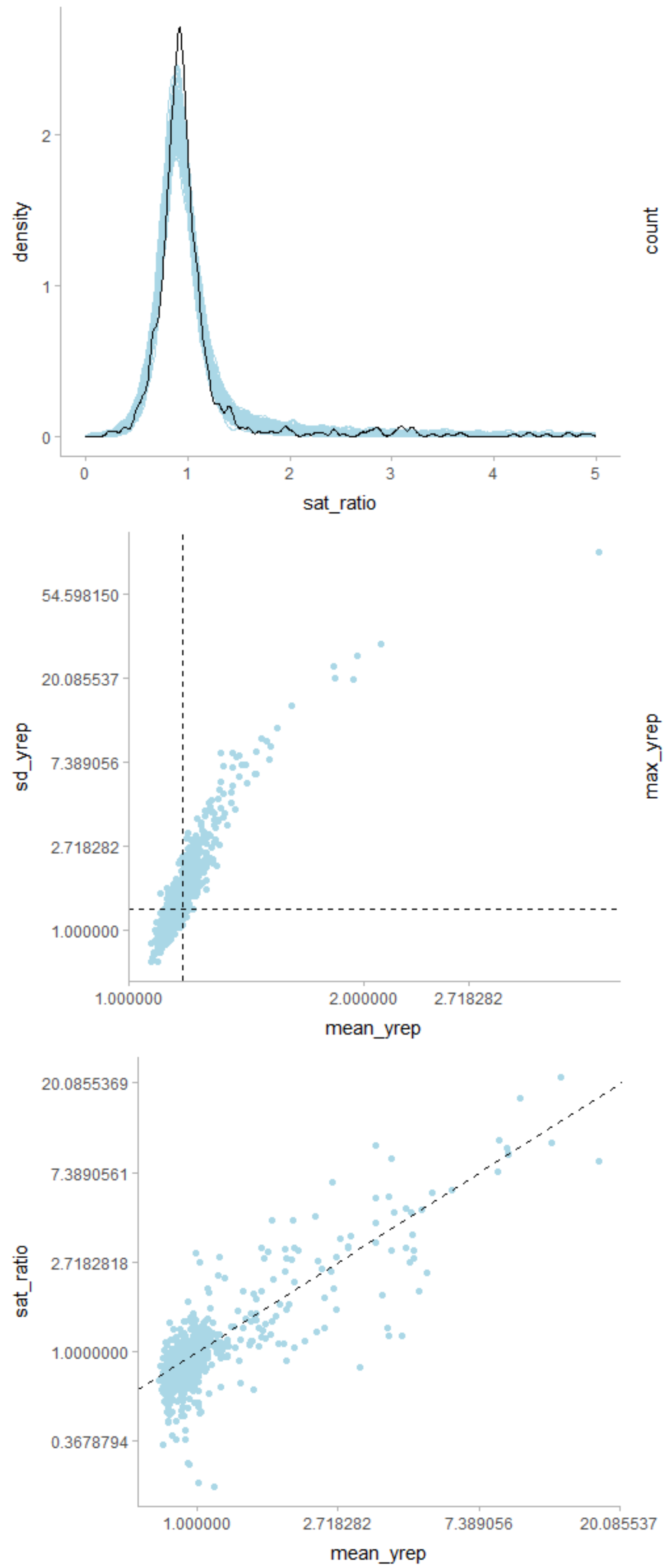

The same check for the revisit sites below:

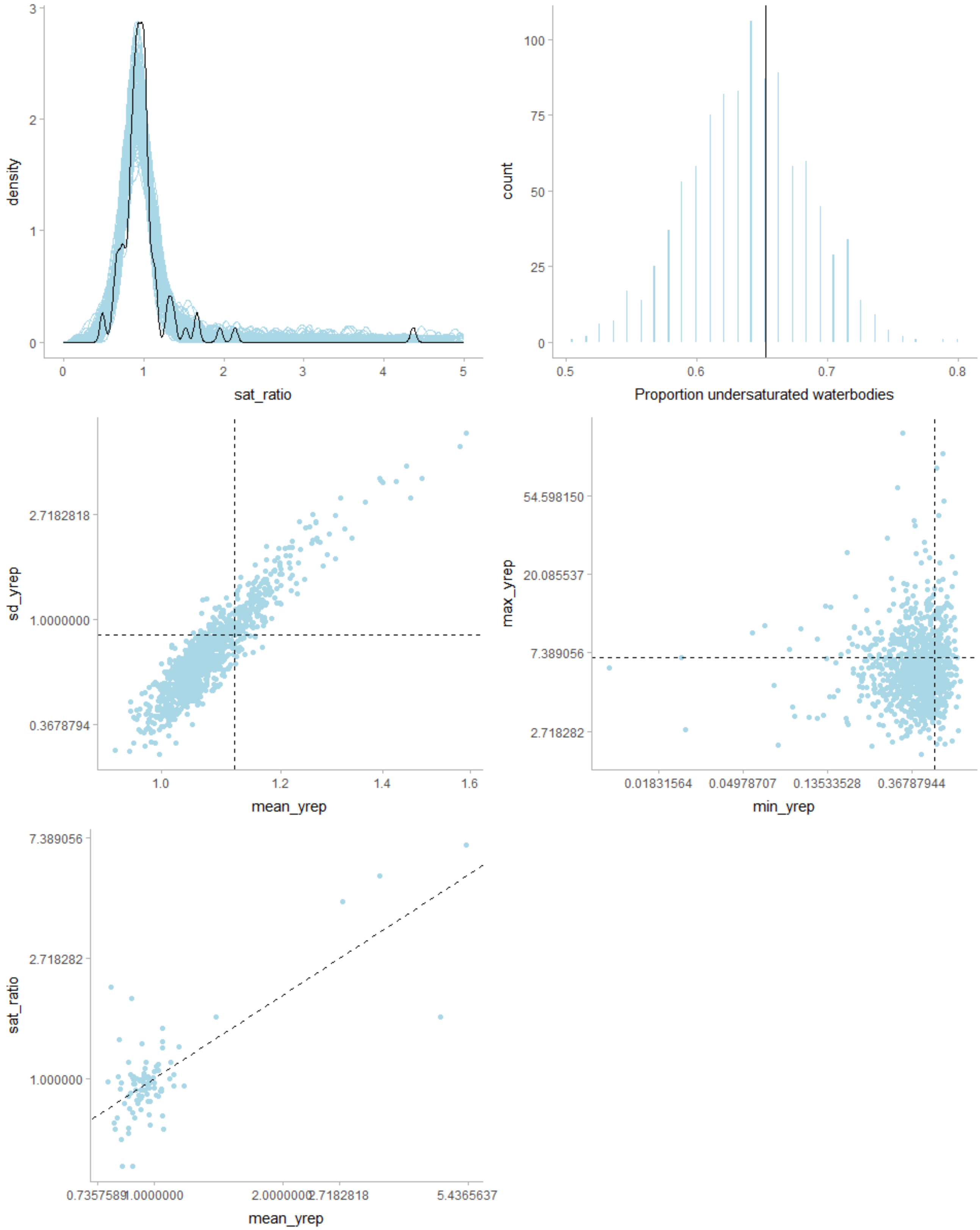

3.5.2.5 R-square

```
##           Estimate Est.Error  Q2.5 Q97.5
## R2logn2o     0.629      0.03 0.563  0.68
```

```
##            Estimate Est.Error  Q2.5 Q97.5
## R2logn2oeq    0.874     0.005 0.864 0.882
```

For the revisit sites:

```
##          Estimate Est.Error Q2.5 Q97.5
## R2logn2o    0.573     0.065 0.43 0.684

##            Estimate Est.Error  Q2.5 Q97.5
## R2logn2oeq    0.869     0.007 0.853 0.882
```

3.5.3 Covariate effects

3.5.3.1 Dissolved N2O

The conditional effects plots suggested a similar effect of NO3, but interesting interactions between NO3 and lake area and NO3 and surface temperature. For lake area, the effect was estimated to be larger and more negative at the highest levels of NO3; and slightly negative at the lowest level of NO3. For surface temperature, the effect was estimated to be largest and positive at the highest level of NO3; and negative at the lowest level of NO3.

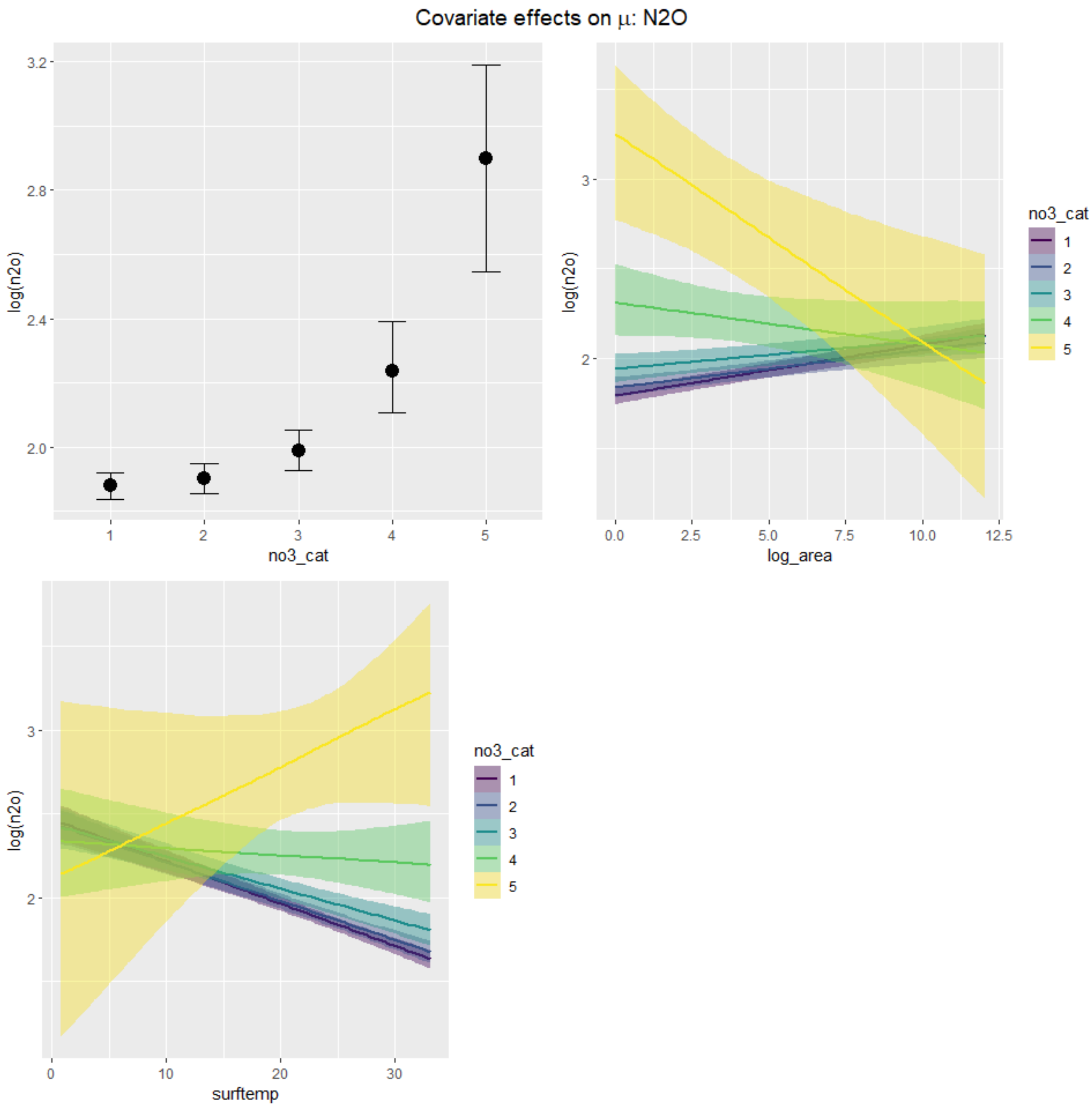

The estimated covariate effects on σ suggested a negative relationship with log(area) and a positive relationship, again, with NO3.

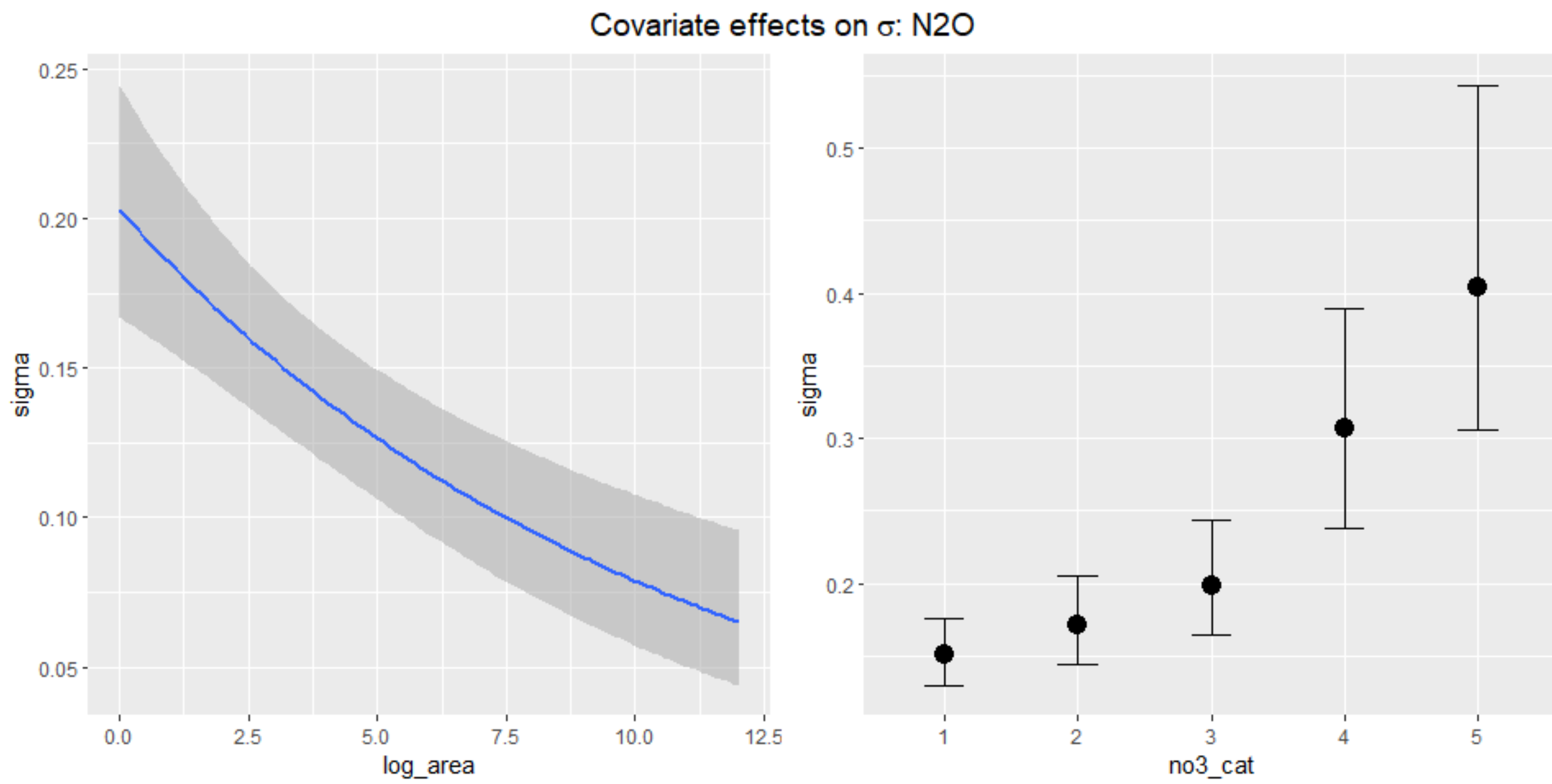

3.5.3.2 Equilibrium N2O

The estimated covariate effects on equilibrium N2O remained largely the same as estimated in the previous model.

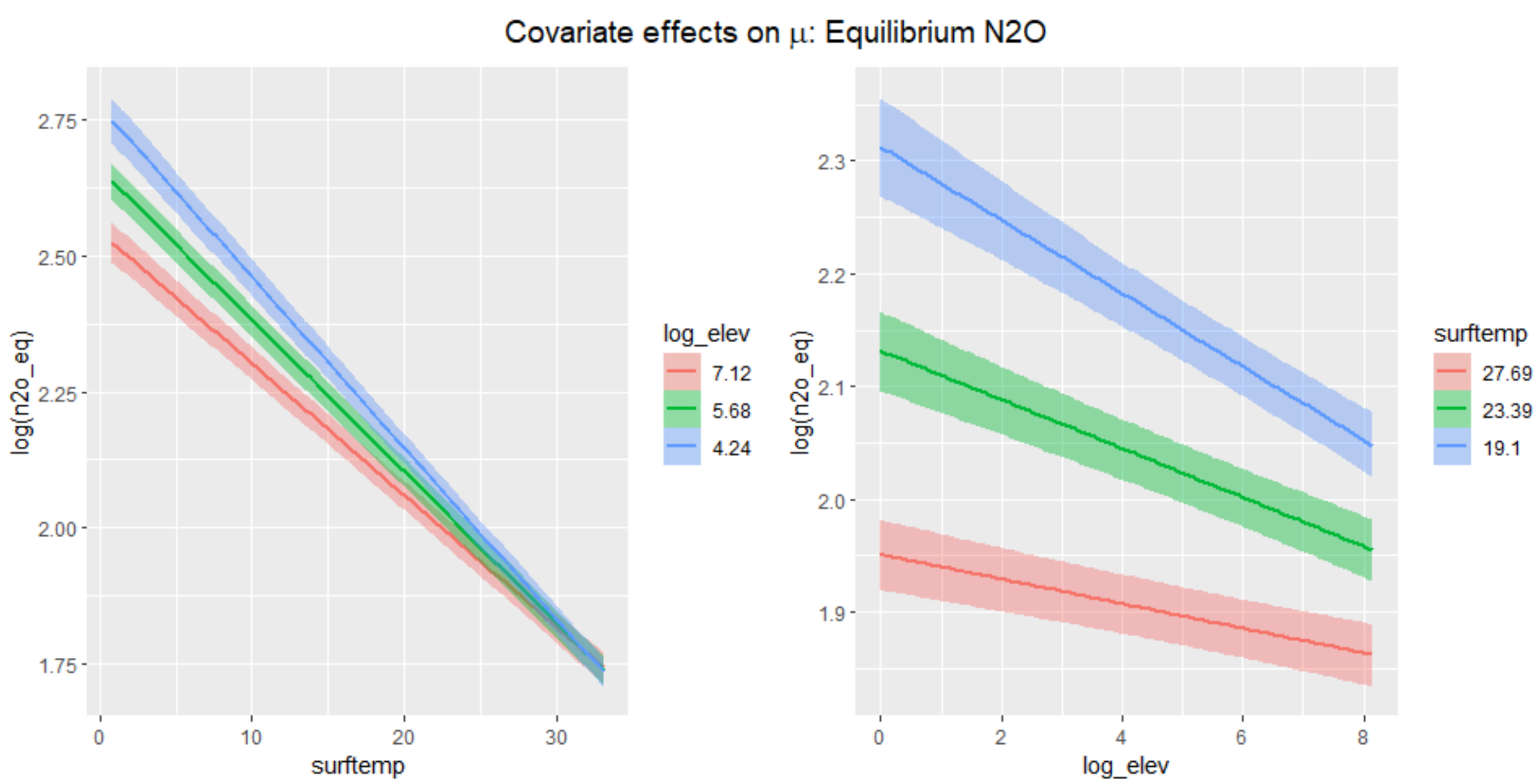

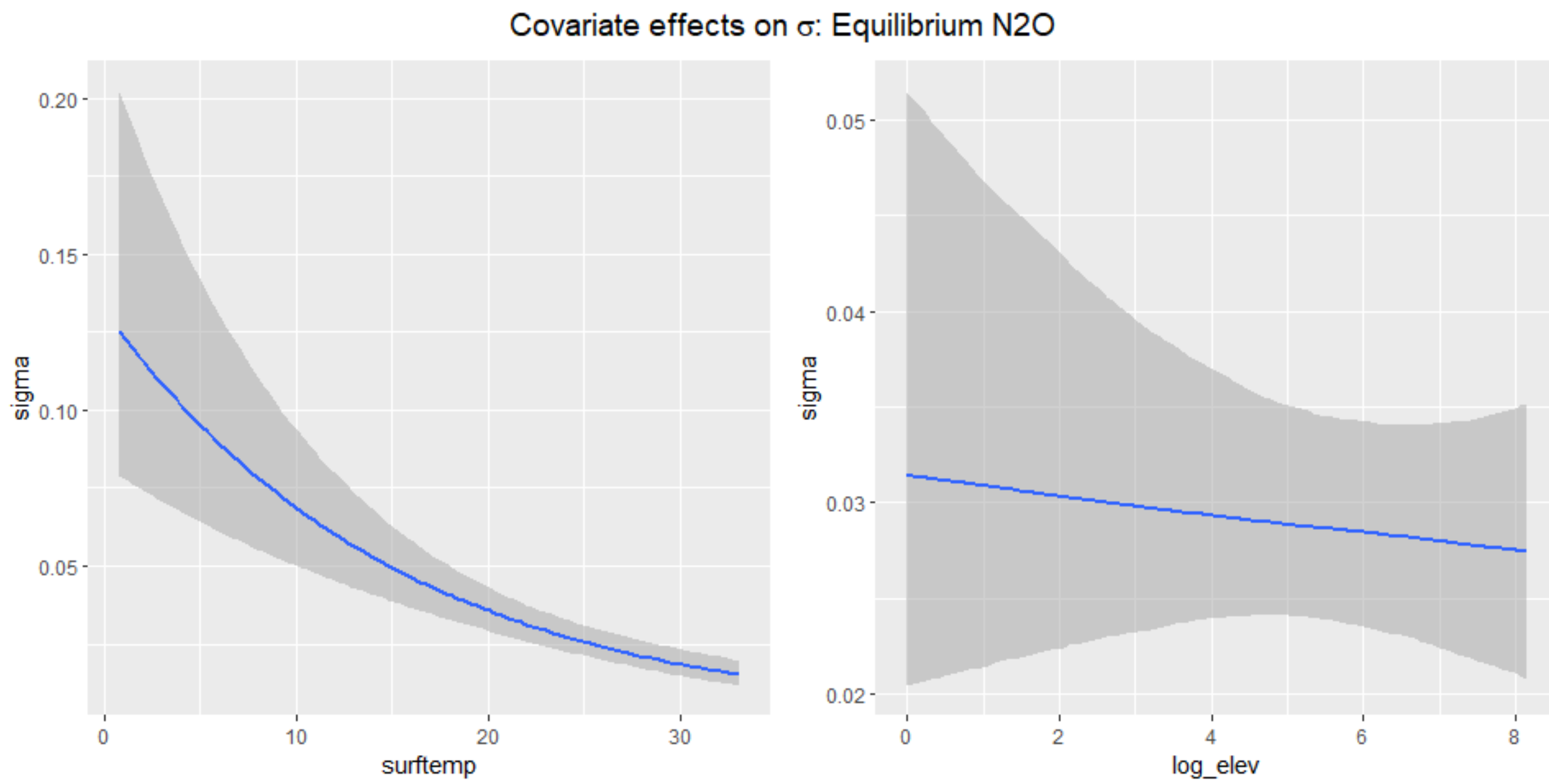

3.6 A Final Model

As demonstrated above, models excluding the NO3 covariate consistently resulted in poorer fits to to the observed dissolved N2O data and potentially biased estimates of the saturation ratio. Including surface temperature and elevation in the equilibrium N2O part of the model also resulted in substantially improved replication of key aspects of the observed data. Added flexibility in the distributional terms for both dissolved and equilibrium N2O also led to improvements. To make inferences to the population of interest from a model including these covariates, however, they needed to be (1) fully observed across that population or (2) their missingness needed to be modeled. For the lake area and elevation covariates, data *was* available for all lakes from previously compiled geospatial databases. However, neither surface temperature or NO3 were observed for lakes outside of the sample. Therefore, the final model below included surface temperature and NO3 as additional responses conditioned on the survey design variables and fully observed covariates.

In the final model below, the sub-model for surface temperature assumed a Gamma distributed error distribution and the linear predictor included the survey design variables, latitude, elevation, and Julian date. The shape parameter was also modeled as a function of latitude to address increasing response variance along the latitudinal gradient. The NO3 response was modeled with a cumulative logit likelihood and the linear predictor included all of the survey factors as well as surface temperature and lake area. The dissolved and equilibrium N2O responses were each modeled with Gamma distributed errors, but with the same covariate structure as in model 5. The same structure was also employed for the shape terms in these responses, corresponding to the σ terms in the previous models. Note

that there was no observation-level residual correlation term for this model, since the residuals are undefined for the Gamma and cumulative logit models. Dropping the observation-level residual correlation term was deemed a reasonable compromise that enabled the inclusion of NO3 and surface temperature in the N2O model response. The random intercepts for the group terms, however, still allowed for potential correlations between responses at the group levels.

```
bf_n2o <- bf(n2o ~ mo(no3_cat) +
               log_area +
               surftemp +
               mo(no3_cat):log_area +
               mo(no3_cat):surftemp +
               (mo(no3_cat) | a | WSA9) +
               (mo(no3_cat) | b | WSA9:state) +
               (1 | c | WSA9:state:size_cat),
             shape ~ log_area +
               mo(no3_cat) +
               (1 | WSA9) +
               (1 | WSA9:state) +
               (1 | WSA9:state:size_cat),
             family = Gamma(link = "log"))

bf_n2oeq <- bf(n2o_eq ~ surftemp +
                 log_elev +
                 surftemp:log_elev +
                 (1 | a | WSA9) +
                 (1 | b | WSA9:state) +
                 (1 | c | WSA9:state:size_cat),
             shape ~ surftemp +
               log_elev +
               (1 | WSA9) +
               (1 | WSA9:state) +
               (1 | WSA9:state:size_cat),
             family = Gamma(link = "log"))

bf_temp <- bf(surftemp ~ lat +
                s(log_elev) +
                s(jdate) +
                (1 | a | WSA9) +
                (1 | b | WSA9:state) +
                (1 | c | WSA9:state:size_cat),
              shape ~ lat,
              family = Gamma(link = "log"))

bf_no3 <- bf(no3_cat ~ surftemp +
               log_area +
               (1 | a | WSA9) +
```

```
                 (1 | b | WSA9:state) +
                 (1 | c | WSA9:state:size_cat),
               family = cumulative(link = "logit", threshold="flexible"))

priors <- c(
  prior(normal(2, 1), class = "Intercept", resp = "n2o"),
  prior(normal(0, 1), class = "b", resp = "n2o"),
  prior(exponential(2), class = "sd", resp = "n2o"),
  prior(normal(5, 4), class = "Intercept", dpar = "shape", resp = "n2o"),
  prior(normal(0, 1), class = "b", dpar = "shape", resp = "n2o"),
  prior(exponential(2), class = "sd", dpar = "shape", resp = "n2o"),

  prior(normal(2, 1), class = "Intercept", resp = "n2oeq"),
  prior(normal(0, 1), class = "b", resp = "n2oeq"),
  prior(exponential(2), class = "sd", resp = "n2oeq"),
  prior(normal(5, 4), class = "Intercept", dpar = "shape", resp = "n2oeq"),
  prior(normal(0, 1), class = "b", dpar = "shape", resp = "n2oeq"),
  prior(exponential(2), class = "sd", dpar = "shape", resp = "n2oeq"),

  prior(normal(3, 1), class = "Intercept", resp = "surftemp"),
  prior(normal(0, 1), class = "b", resp = "surftemp"),
  prior(exponential(0.5), class = "sds", resp = "surftemp"),
  prior(exponential(2), class = "sd", resp = "surftemp"),
  prior(normal(5, 4), class = "Intercept", dpar = "shape", resp = "surftemp"),
  prior(normal(0, 1), class = "b", dpar = "shape", resp = "surftemp"),

  prior(normal(0, 3), class = "Intercept", resp = "no3cat"),
  prior(normal(0, 1), class = "b", resp = "no3cat"),
  prior(exponential(1), class = "sd", resp = "no3cat"),

  prior(lkj(2), class = "cor")
  )

n2o_mod6 <- brm(bf_n2o +
                  bf_n2oeq +
                  bf_temp +
                  bf_no3 +
                  set_rescor(rescor = FALSE),
                data = df_model,
                prior = priors,
  control = list(adapt_delta = 0.975, max_treedepth = 14),
  #sample_prior = "only",
  save_pars = save_pars(all = TRUE),
  seed = 85132,#14548,
  #init = my_inits,
  init_r = 0.5,
  chains=4,
  iter=5000,
  cores=4)
```

```
save(n2o_mod6, file = "C:/Users/rmartin/OneDrive - Environmental Protection Agency (E
```

3.6.1 Summarize fit

Below is a summary of the fitted parameters and MCMC diagnostics.

```
##  Family: MV(gamma, gamma, gamma, cumulative)
##   Links: mu = log; shape = log
##          mu = log; shape = log
##          mu = log; shape = log
##          mu = logit; disc = identity
## Formula: n2o ~ mo(no3_cat) + log_area + surftemp + mo(no3_cat):log_area + mo(no3_c
##          shape ~ log_area + mo(no3_cat) + (1 | WSA9) + (1 | WSA9:state) + (1 | WSA
##          n2o_eq ~ surftemp + log_elev + surftemp:log_elev + (1 | a | WSA9) + (1 |
##          shape ~ surftemp + log_elev + (1 | WSA9) + (1 | WSA9:state) + (1 | WSA9:s
##          surftemp ~ lat + s(log_elev) + s(jdate) + (1 | a | WSA9) + (1 | b | WSA9:
##          shape ~ lat
##          no3_cat ~ surftemp + log_area + (1 | a | WSA9) + (1 | b | WSA9:state) + (
##    Data: df_model (Number of observations: 984)
##   Draws: 4 chains, each with iter = 5000; warmup = 2500; thin = 1;
##          total post-warmup draws = 10000
##
## Priors:
## b_n2o ~ normal(0, 1)
## b_n2o_shape ~ normal(0, 1)
## b_n2oeq ~ normal(0, 1)
## b_n2oeq_shape ~ normal(0, 1)
## b_no3cat ~ normal(0, 1)
## b_surftemp ~ normal(0, 1)
## b_surftemp_shape ~ normal(0, 1)
## Intercept_n2o ~ normal(2, 1)
## Intercept_n2o_shape ~ normal(5, 4)
## Intercept_n2oeq ~ normal(2, 1)
## Intercept_n2oeq_shape ~ normal(5, 4)
## Intercept_no3cat ~ normal(0, 3)
## Intercept_surftemp ~ normal(3, 1)
## Intercept_surftemp_shape ~ normal(5, 4)
## L ~ lkj_corr_cholesky(2)
## <lower=0> sd_n2o ~ exponential(2)
## <lower=0> sd_n2o_shape ~ exponential(2)
## <lower=0> sd_n2oeq ~ exponential(2)
## <lower=0> sd_n2oeq_shape ~ exponential(2)
## <lower=0> sd_no3cat ~ exponential(1)
## <lower=0> sd_surftemp ~ exponential(2)
## <lower=0> sds_surftemp ~ exponential(0.5)
## simo_n2o_mono3_cat:log_area1 ~ dirichlet(1)
```

```
## simo_n2o_mono3_cat:surftemp1 ~ dirichlet(1)
## simo_n2o_mono3_cat1 ~ dirichlet(1)
## simo_n2o_shape_mono3_cat1 ~ dirichlet(1)
##
## Smoothing Spline Hyperparameters:
##                            Estimate Est.Error l-95% CI u-95% CI  Rhat Bulk_ESS
## sds(surftemp_slog_elev_1)    1.161     0.370    0.638    2.079 1.001     2264
## sds(surftemp_sjdate_1)       0.571     0.277    0.226    1.273 1.000     2679
##                            Tail_ESS
## sds(surftemp_slog_elev_1)     4174
## sds(surftemp_sjdate_1)        4853
##
## Multilevel Hyperparameters:
## ~WSA9 (Number of levels: 9)
##                                           Estimate Est.Error l-95% CI u-95% CI
## sd(n2o_Intercept)                            0.048     0.018    0.020    0.091
## sd(n2o_mono3_cat)                            0.045     0.035    0.002    0.129
## sd(n2oeq_Intercept)                          0.033     0.011    0.018    0.061
## sd(surftemp_Intercept)                       0.031     0.014    0.011    0.064
## sd(no3cat_Intercept)                         0.690     0.256    0.296    1.308
## sd(shape_n2o_Intercept)                      0.211     0.141    0.011    0.535
## sd(shape_n2oeq_Intercept)                    0.402     0.183    0.075    0.814
## cor(n2o_Intercept,n2o_mono3_cat)            -0.049     0.336   -0.667    0.614
## cor(n2o_Intercept,n2oeq_Intercept)           0.393     0.268   -0.202    0.824
## cor(n2o_mono3_cat,n2oeq_Intercept)           0.102     0.328   -0.559    0.686
## cor(n2o_Intercept,surftemp_Intercept)       -0.350     0.296   -0.839    0.285
## cor(n2o_mono3_cat,surftemp_Intercept)        0.089     0.333   -0.564    0.703
## cor(n2oeq_Intercept,surftemp_Intercept)     -0.167     0.299   -0.705    0.437
## cor(n2o_Intercept,no3cat_Intercept)         -0.057     0.295   -0.605    0.522
## cor(n2o_mono3_cat,no3cat_Intercept)          0.141     0.333   -0.539    0.724
## cor(n2oeq_Intercept,no3cat_Intercept)        0.272     0.274   -0.305    0.740
## cor(surftemp_Intercept,no3cat_Intercept)     0.185     0.300   -0.436    0.718
##                                            Rhat Bulk_ESS Tail_ESS
## sd(n2o_Intercept)                         1.000     3011     2931
## sd(n2o_mono3_cat)                         1.003     1540     3502
## sd(n2oeq_Intercept)                       1.000     3147     4572
## sd(surftemp_Intercept)                    1.001     3705     4498
## sd(no3cat_Intercept)                      1.000     4184     5618
## sd(shape_n2o_Intercept)                   1.000     2379     3641
## sd(shape_n2oeq_Intercept)                 1.001     2208     1617
## cor(n2o_Intercept,n2o_mono3_cat)          1.000     6558     6134
## cor(n2o_Intercept,n2oeq_Intercept)        1.000     4604     6010
## cor(n2o_mono3_cat,n2oeq_Intercept)        1.000     3241     5029
## cor(n2o_Intercept,surftemp_Intercept)     1.000     4744     5916
## cor(n2o_mono3_cat,surftemp_Intercept)     1.000     5246     6447
## cor(n2oeq_Intercept,surftemp_Intercept)   1.000     7342     7508
## cor(n2o_Intercept,no3cat_Intercept)       1.000     5152     6479
## cor(n2o_mono3_cat,no3cat_Intercept)       1.002     3435     5897
## cor(n2oeq_Intercept,no3cat_Intercept)     1.001     6449     7400
```

```
## cor(surftemp_Intercept,no3cat_Intercept) 1.000     6490     7928
##
## ~WSA9:state (Number of levels: 96)
##                                         Estimate Est.Error l-95% CI u-95% CI
## sd(n2o_Intercept)                          0.047     0.013    0.021    0.072
## sd(n2o_mono3_cat)                          0.101     0.019    0.068    0.142
## sd(n2oeq_Intercept)                        0.033     0.003    0.026    0.040
## sd(surftemp_Intercept)                     0.035     0.006    0.023    0.047
## sd(no3cat_Intercept)                       0.878     0.128    0.649    1.147
## sd(shape_n2o_Intercept)                    0.383     0.178    0.032    0.707
## sd(shape_n2oeq_Intercept)                  0.561     0.114    0.336    0.781
## cor(n2o_Intercept,n2o_mono3_cat)          -0.145     0.254   -0.602    0.376
## cor(n2o_Intercept,n2oeq_Intercept)         0.183     0.189   -0.180    0.549
## cor(n2o_mono3_cat,n2oeq_Intercept)         0.201     0.164   -0.122    0.519
## cor(n2o_Intercept,surftemp_Intercept)     -0.023     0.237   -0.476    0.438
## cor(n2o_mono3_cat,surftemp_Intercept)     -0.232     0.215   -0.644    0.194
## cor(n2oeq_Intercept,surftemp_Intercept)   -0.134     0.190   -0.494    0.246
## cor(n2o_Intercept,no3cat_Intercept)        0.462     0.211    0.018    0.822
## cor(n2o_mono3_cat,no3cat_Intercept)        0.145     0.200   -0.251    0.531
## cor(n2oeq_Intercept,no3cat_Intercept)      0.054     0.140   -0.220    0.329
## cor(surftemp_Intercept,no3cat_Intercept)  -0.231     0.189   -0.586    0.154
##                                          Rhat Bulk_ESS Tail_ESS
## sd(n2o_Intercept)                       1.004     1025     1337
## sd(n2o_mono3_cat)                       1.001     3366     4975
## sd(n2oeq_Intercept)                     1.001     2809     4611
## sd(surftemp_Intercept)                  1.000     4124     5116
## sd(no3cat_Intercept)                    1.000     4389     6247
## sd(shape_n2o_Intercept)                 1.004      649     1304
## sd(shape_n2oeq_Intercept)               1.002     1908     1810
## cor(n2o_Intercept,n2o_mono3_cat)        1.002     1069     1955
## cor(n2o_Intercept,n2oeq_Intercept)      1.003      794     1260
## cor(n2o_mono3_cat,n2oeq_Intercept)      1.002      833     1873
## cor(n2o_Intercept,surftemp_Intercept)   1.004     1849     3618
## cor(n2o_mono3_cat,surftemp_Intercept)   1.001     2626     4267
## cor(n2oeq_Intercept,surftemp_Intercept) 1.000     6340     6860
## cor(n2o_Intercept,no3cat_Intercept)     1.004      893     1944
## cor(n2o_mono3_cat,no3cat_Intercept)     1.000     1746     3085
## cor(n2oeq_Intercept,no3cat_Intercept)   1.000     6378     7983
## cor(surftemp_Intercept,no3cat_Intercept) 1.001     3573     5949
##
## ~WSA9:state:size_cat (Number of levels: 352)
##                                         Estimate Est.Error l-95% CI u-95% CI
## sd(n2o_Intercept)                          0.038     0.014    0.006    0.064
## sd(n2oeq_Intercept)                        0.004     0.002    0.000    0.008
## sd(surftemp_Intercept)                     0.010     0.007    0.000    0.024
## sd(no3cat_Intercept)                       0.308     0.182    0.016    0.673
## sd(shape_n2o_Intercept)                    0.895     0.110    0.684    1.114
## sd(shape_n2oeq_Intercept)                  0.511     0.104    0.309    0.715
## cor(n2o_Intercept,n2oeq_Intercept)         0.310     0.349   -0.486    0.841
```

```
## cor(n2o_Intercept,surftemp_Intercept)       0.015     0.365   -0.677    0.705
## cor(n2oeq_Intercept,surftemp_Intercept)    -0.102     0.381   -0.761    0.659
## cor(n2o_Intercept,no3cat_Intercept)        -0.090     0.342   -0.716    0.601
## cor(n2oeq_Intercept,no3cat_Intercept)      -0.143     0.359   -0.757    0.611
## cor(surftemp_Intercept,no3cat_Intercept)    0.173     0.378   -0.599    0.807
##                                           Rhat Bulk_ESS Tail_ESS
## sd(n2o_Intercept)                         1.011      580     1036
## sd(n2oeq_Intercept)                       1.009     1062     3160
## sd(surftemp_Intercept)                    1.003     1917     3889
## sd(no3cat_Intercept)                      1.002     1078     2386
## sd(shape_n2o_Intercept)                   1.002     1068     2786
## sd(shape_n2oeq_Intercept)                 1.002     1397     2071
## cor(n2o_Intercept,n2oeq_Intercept)        1.004     2099     4705
## cor(n2o_Intercept,surftemp_Intercept)     1.000     4876     6414
## cor(n2oeq_Intercept,surftemp_Intercept)   1.001     4195     5741
## cor(n2o_Intercept,no3cat_Intercept)       1.001     3188     5334
## cor(n2oeq_Intercept,no3cat_Intercept)     1.001     2749     4946
## cor(surftemp_Intercept,no3cat_Intercept)  1.001     2607     5750
##
## Regression Coefficients:
##                          Estimate Est.Error l-95% CI u-95% CI  Rhat Bulk_ESS
## n2o_Intercept               2.392     0.056    2.285    2.500 1.000     3063
## shape_n2o_Intercept         3.215     0.189    2.849    3.589 1.001     3997
## n2oeq_Intercept             3.111     0.053    3.011    3.218 1.000     5146
## shape_n2oeq_Intercept       3.873     0.751    2.385    5.326 1.001     5619
## surftemp_Intercept          3.791     0.060    3.672    3.906 1.000     7414
## shape_surftemp_Intercept    8.637     0.460    7.721    9.522 1.001    10817
## no3cat_Intercept[1]        -3.025     0.615   -4.258   -1.864 1.000     5932
## no3cat_Intercept[2]        -2.059     0.605   -3.274   -0.903 1.000     6278
## no3cat_Intercept[3]        -1.027     0.600   -2.235    0.115 1.000     6689
## no3cat_Intercept[4]        -0.028     0.602   -1.247    1.113 1.000     7002
## n2o_log_area                0.028     0.003    0.022    0.034 1.000     6958
## n2o_surftemp               -0.025     0.002   -0.029   -0.021 1.000     3145
## shape_n2o_log_area          0.190     0.041    0.110    0.271 1.000     4322
## n2oeq_surftemp             -0.042     0.002   -0.045   -0.038 1.000     5836
## n2oeq_log_elev             -0.080     0.008   -0.097   -0.064 1.000     5425
## n2oeq_surftemp:log_elev     0.002     0.000    0.002    0.003 1.000     5857
## shape_n2oeq_surftemp        0.131     0.020    0.092    0.171 1.001     6725
## shape_n2oeq_log_elev        0.030     0.077   -0.117    0.186 1.001     4914
## surftemp_lat               -0.016     0.001   -0.019   -0.013 1.000     7349
## shape_surftemp_lat         -0.105     0.011   -0.127   -0.083 1.000    11330
## no3cat_surftemp            -0.141     0.023   -0.187   -0.096 1.001     6713
## no3cat_log_area             0.068     0.035   -0.001    0.137 1.000    11692
## surftemp_slog_elev_1       -3.477     0.479   -4.414   -2.557 1.000     5891
## surftemp_sjdate_1          -0.008     0.528   -1.074    1.017 1.000     4010
## n2o_mono3_cat               0.007     0.087   -0.172    0.175 1.004     1650
## n2o_mono3_cat:log_area     -0.046     0.009   -0.063   -0.027 1.001     2079
## n2o_mono3_cat:surftemp      0.018     0.004    0.009    0.026 1.004     1417
## shape_n2o_mono3_cat        -0.510     0.070   -0.649   -0.375 1.001     4691
```

```
##                           Tail_ESS
## n2o_Intercept                 5517
## shape_n2o_Intercept           6108
## n2oeq_Intercept               6075
## shape_n2oeq_Intercept         6535
## surftemp_Intercept            7632
## shape_surftemp_Intercept      7718
## no3cat_Intercept[1]           6622
## no3cat_Intercept[2]           6695
## no3cat_Intercept[3]           6957
## no3cat_Intercept[4]           6940
## n2o_log_area                  7668
## n2o_surftemp                  6260
## shape_n2o_log_area            5605
## n2oeq_surftemp                6494
## n2oeq_log_elev                6234
## n2oeq_surftemp:log_elev       6696
## shape_n2oeq_surftemp          7112
## shape_n2oeq_log_elev          6636
## surftemp_lat                  7598
## shape_surftemp_lat            7919
## no3cat_surftemp               7328
## no3cat_log_area               8177
## surftemp_slog_elev_1          6330
## surftemp_sjdate_1             5523
## n2o_mono3_cat                 1855
## n2o_mono3_cat:log_area        2821
## n2o_mono3_cat:surftemp        1621
## shape_n2o_mono3_cat           6283
##
## Monotonic Simplex Parameters:
##                             Estimate Est.Error l-95% CI u-95% CI  Rhat Bulk_ESS
## n2o_mono3_cat1[1]              0.025     0.023    0.001    0.083 1.000     4899
## n2o_mono3_cat1[2]              0.183     0.111    0.014    0.425 1.002     1056
## n2o_mono3_cat1[3]              0.408     0.158    0.103    0.725 1.002     2167
## n2o_mono3_cat1[4]              0.384     0.169    0.058    0.713 1.003     1608
## n2o_mono3_cat:log_area1[1]     0.046     0.029    0.003    0.112 1.000     4018
## n2o_mono3_cat:log_area1[2]     0.033     0.028    0.001    0.104 1.001     6173
## n2o_mono3_cat:log_area1[3]     0.289     0.134    0.057    0.598 1.000     3286
## n2o_mono3_cat:log_area1[4]     0.631     0.140    0.301    0.864 1.000     3186
## n2o_mono3_cat:surftemp1[1]     0.028     0.019    0.003    0.060 1.001     3744
## n2o_mono3_cat:surftemp1[2]     0.066     0.033    0.011    0.128 1.001     3821
## n2o_mono3_cat:surftemp1[3]     0.281     0.079    0.122    0.431 1.003     2897
## n2o_mono3_cat:surftemp1[4]     0.625     0.088    0.461    0.798 1.003     2740
## shape_n2o_mono3_cat1[1]        0.116     0.067    0.009    0.263 1.001     5363
## shape_n2o_mono3_cat1[2]        0.149     0.096    0.008    0.365 1.001     3469
## shape_n2o_mono3_cat1[3]        0.401     0.151    0.116    0.703 1.001     4122
## shape_n2o_mono3_cat1[4]        0.334     0.143    0.051    0.604 1.001     3853
##                             Tail_ESS
```

```
## n2o_mono3_cat1[1]             5237
## n2o_mono3_cat1[2]             3343
## n2o_mono3_cat1[3]             4583
## n2o_mono3_cat1[4]             3021
## n2o_mono3_cat:log_area1[1]     3309
## n2o_mono3_cat:log_area1[2]     4948
## n2o_mono3_cat:log_area1[3]     4461
## n2o_mono3_cat:log_area1[4]     4184
## n2o_mono3_cat:surftemp1[1]     3410
## n2o_mono3_cat:surftemp1[2]     3213
## n2o_mono3_cat:surftemp1[3]     2971
## n2o_mono3_cat:surftemp1[4]     3106
## shape_n2o_mono3_cat1[1]        4297
## shape_n2o_mono3_cat1[2]        3637
## shape_n2o_mono3_cat1[3]        5848
## shape_n2o_mono3_cat1[4]        4561
##
## Further Distributional Parameters:
##             Estimate Est.Error l-95% CI u-95% CI Rhat Bulk_ESS Tail_ESS
## disc_no3cat    1.000     0.000    1.000    1.000   NA       NA       NA
##
## Draws were sampled using sampling(NUTS). For each parameter, Bulk_ESS
## and Tail_ESS are effective sample size measures, and Rhat is the potential
## scale reduction factor on split chains (at convergence, Rhat = 1).
```

3.6.2 Model checks

Below, the same PPCs for dissolved and equilibrium N2O were used as before.

3.6.2.1 Dissolved N2O

The PPCs for dissolved N2O were similar to those for models 4 and 5 above.

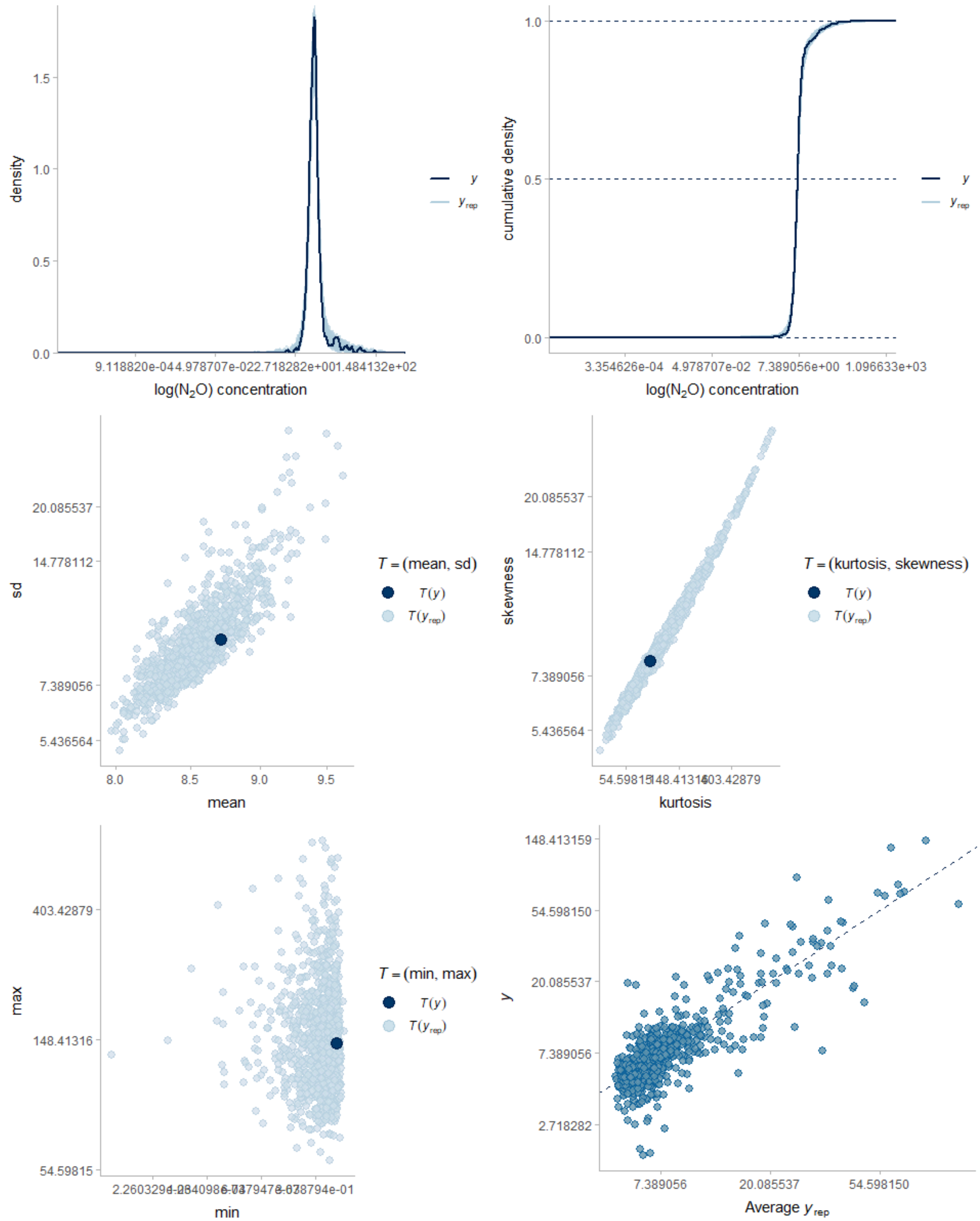

The same check for the re-visit data:

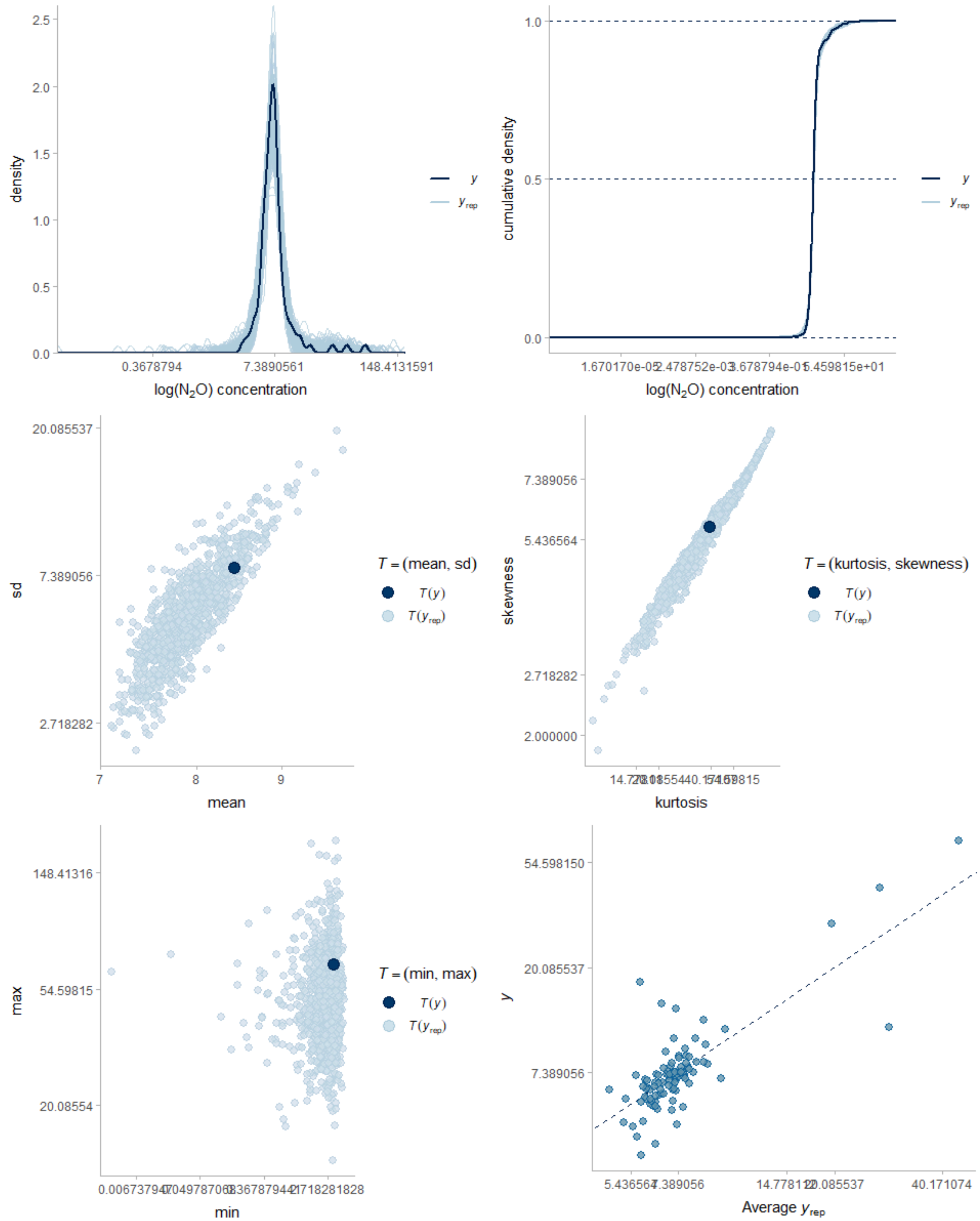

3.6.2.2 Equilibrium N2O

The PPCs for eqilibrium N2O were also similar to the same checks in models 4 and 5.

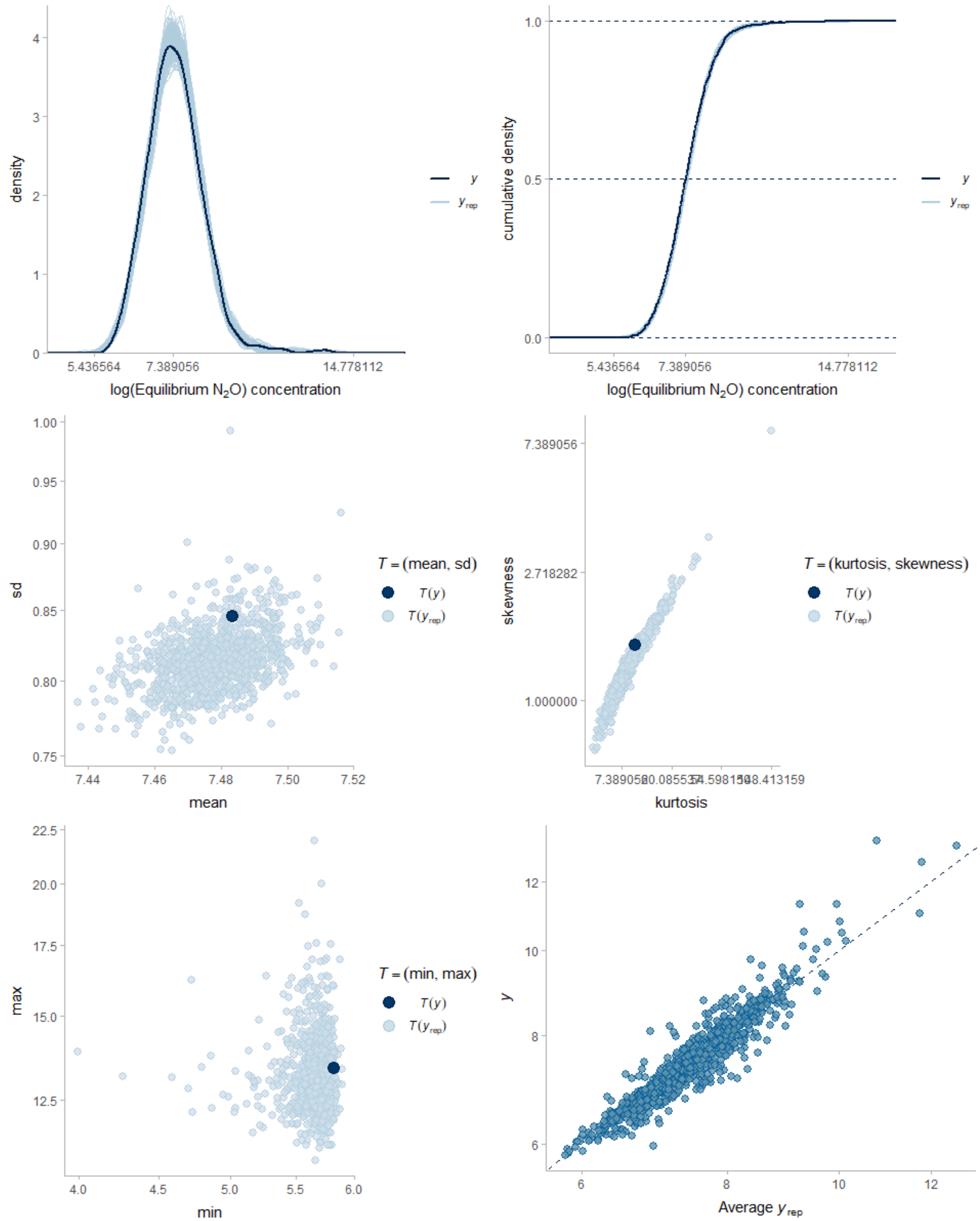

The same check with the re-visit data:

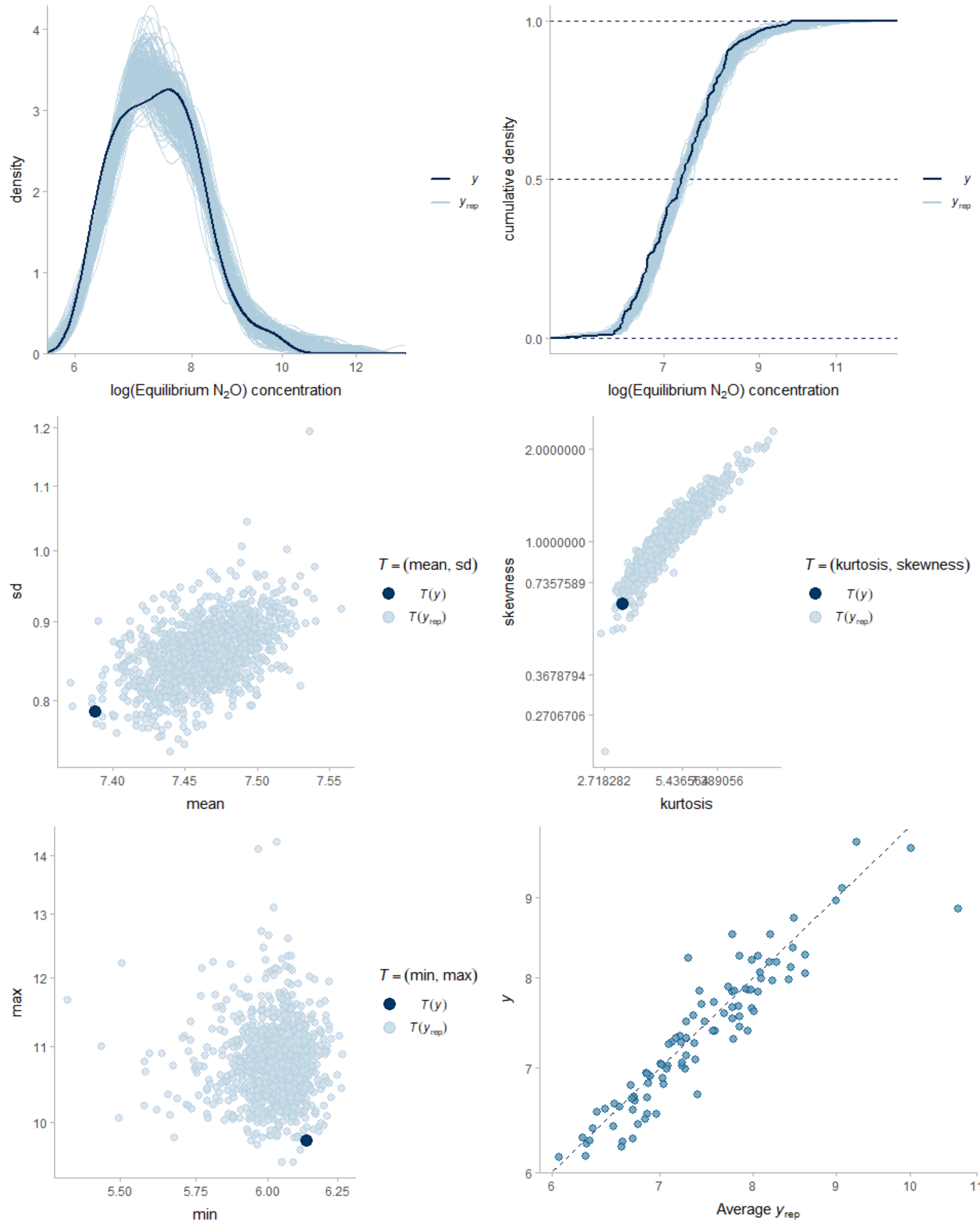

3.6.2.3 Bivariate

This final model provided a reasonable representation of the bivariate relationship between the two N2O responses.

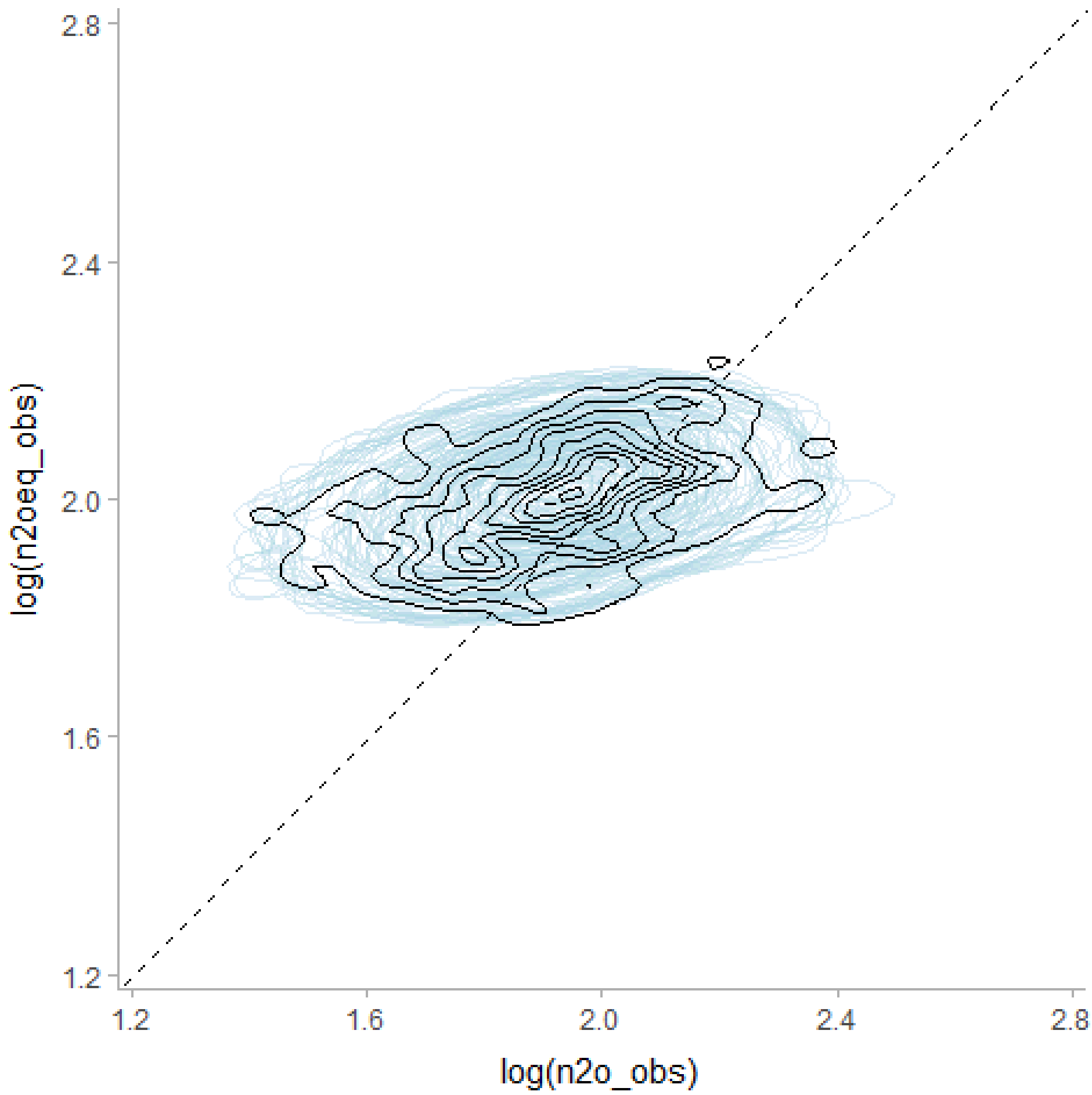

The check with the re-visit data:

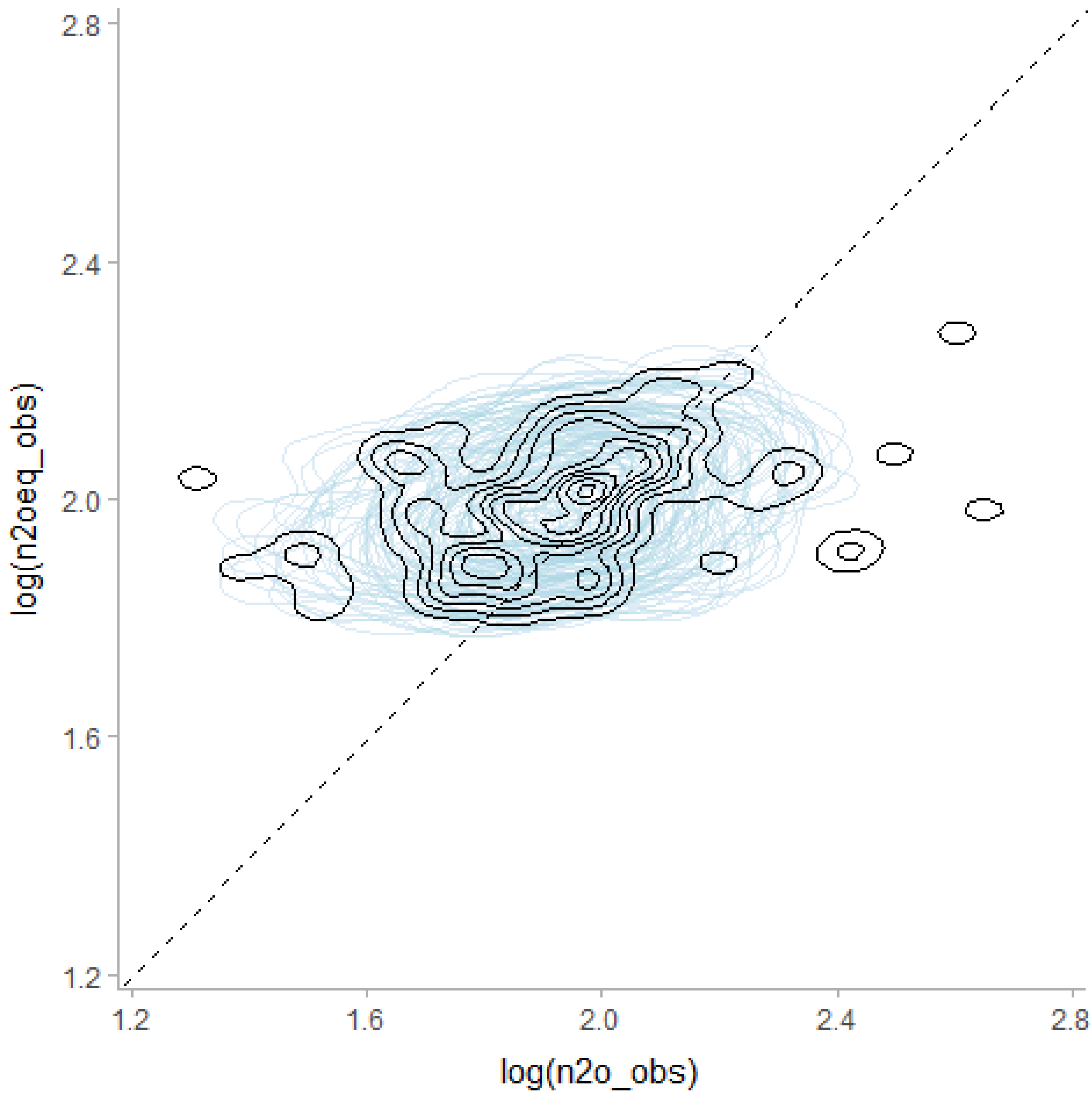

3.6.2.4 Saturation

The saturation ratio PPCs below suggested similar behavior as with models 4 and 5 above, but with perhaps slightly less bias in the predictions for the proportion of undersaturated waterbodies and fewer extreme predictions for the means and standard deviations. The observed *vs.* predicted PPC also appears to have a better behaved variance and no extreme predictions, compared to models 4 and 5.

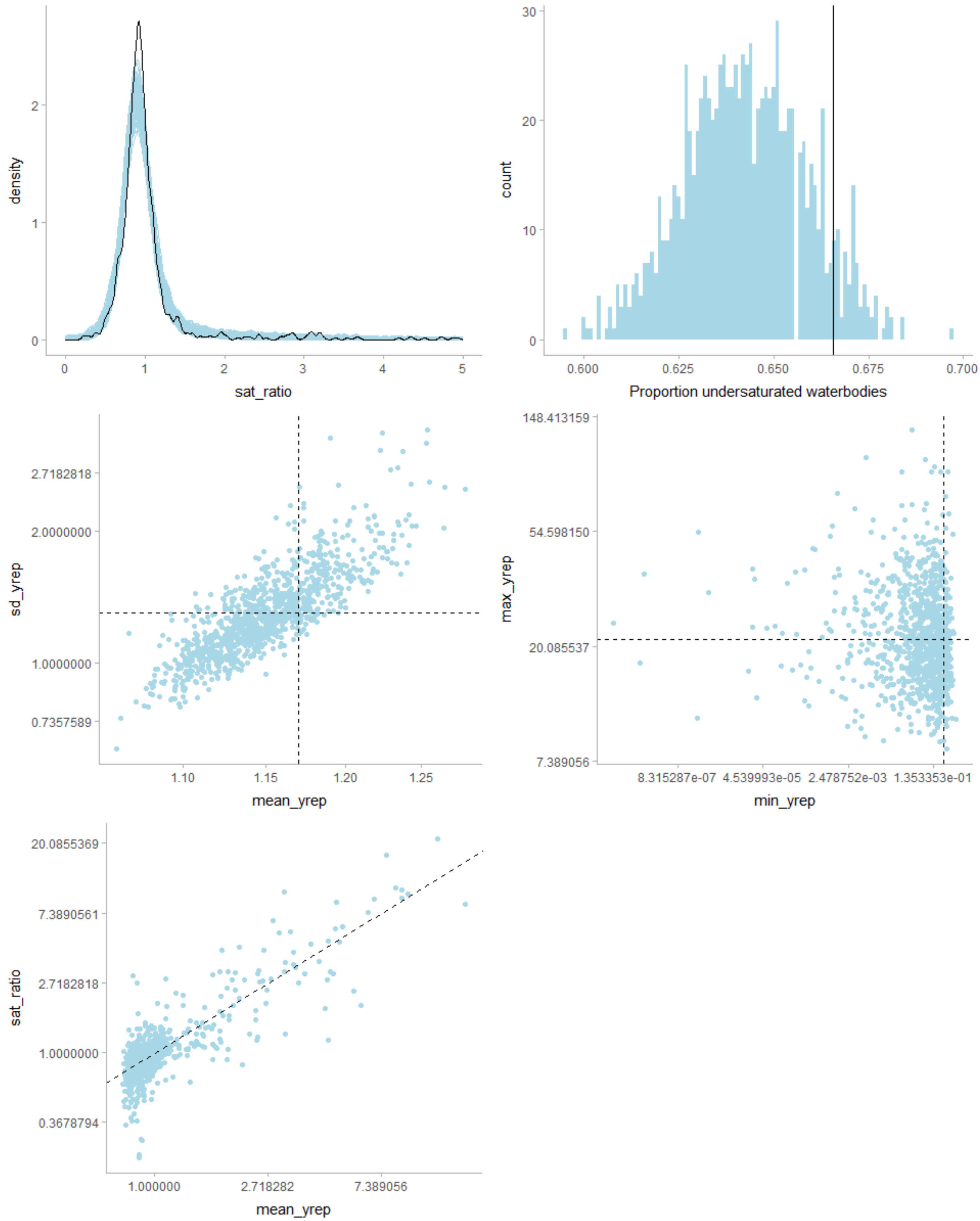

The check for the second-vist data:

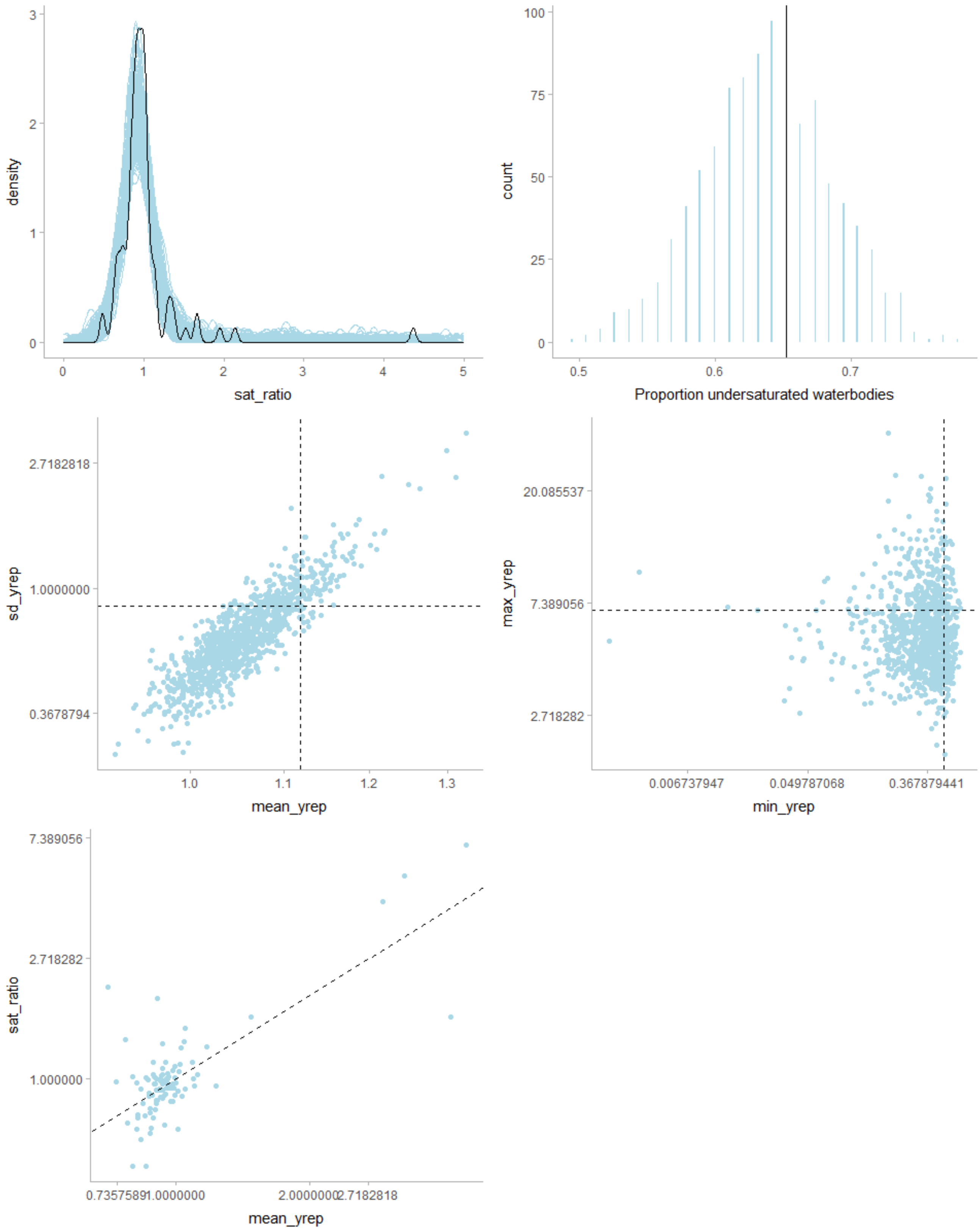

3.6.2.5 R-square

Below are estimates for the Bayesian R^2, which were largely similar for the N2O responses as with models 4 and 5 above. The R^2 for the surface temperature response also suggested a good fit.

```
##       Estimate Est.Error  Q2.5 Q97.5
## R2n2o    0.646     0.059 0.503 0.731

##         Estimate Est.Error  Q2.5 Q97.5
## R2n2oeq    0.863     0.006 0.851 0.874

##            Estimate Est.Error  Q2.5 Q97.5
## R2surftemp    0.652     0.036 0.574 0.718
```

Below are the R^2 estimates for the second-visit data. That these estimates were similar to those for the data used to fit the model was encouraging and suggested that the model may perform reasonably well out-of-sample.

```
##       Estimate Est.Error  Q2.5 Q97.5
## R2n2o    0.607     0.137 0.325  0.85

##         Estimate Est.Error Q2.5 Q97.5
## R2n2oeq    0.857     0.008 0.84 0.872

##            Estimate Est.Error  Q2.5 Q97.5
## R2surftemp    0.621     0.042 0.535 0.707
```

3.6.3 Covariate effects

3.6.3.1 Dissolved N2O

The conditional effects plot for the covariate effects on dissolved N2O suggested a similar effect of NO3 as in previous models, but with interesting potential interactions between NO3 and lake area and NO3 and surface temperature. The lake area effect was estimated to be larger and more negative at the highest levels of NO3 and slightly negative at the lowest level of NO3. The surface temperature effect was estimated to be largest and positive at the highest level of NO3 and negative at the lowest level of NO3.

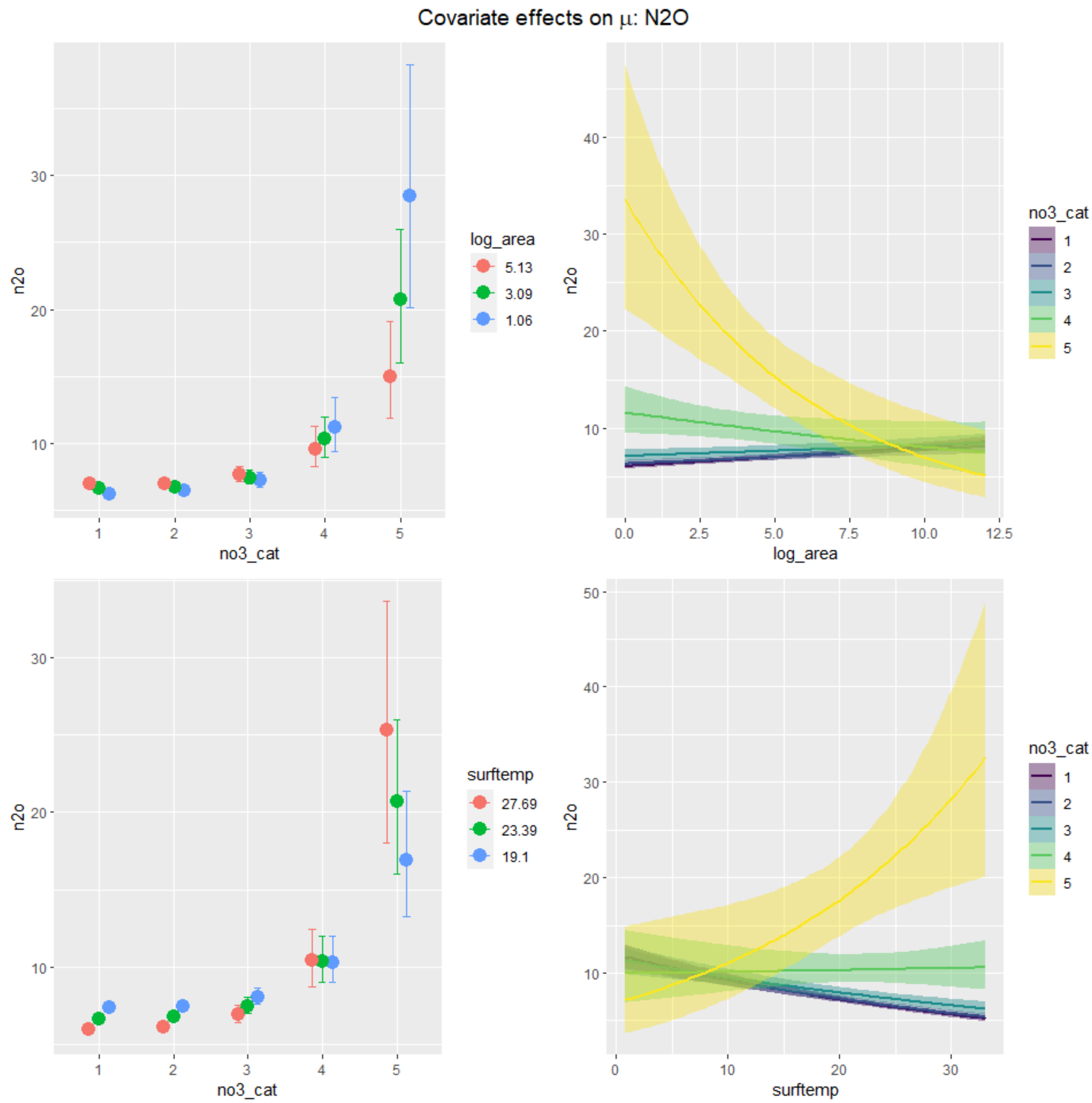

The estimated covariate effects on σ for dissolved N2O suggested a negative relationship with log(area) and a positive relationship with NO3.

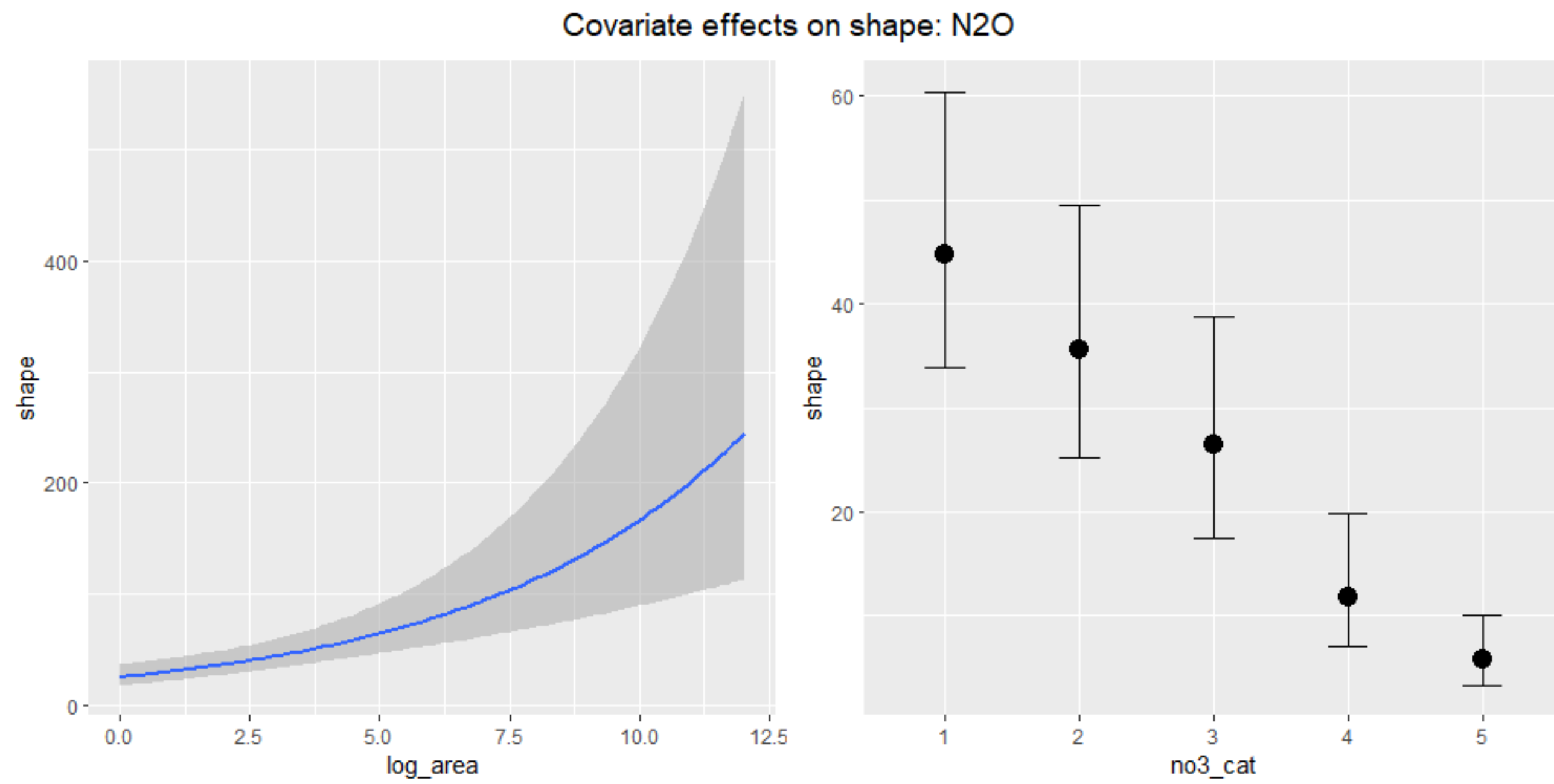

3.6.3.2 Equilibrium N2O

The estimated covariate effects on equilibrium N2O remained largely the same as estimated in the previous model.

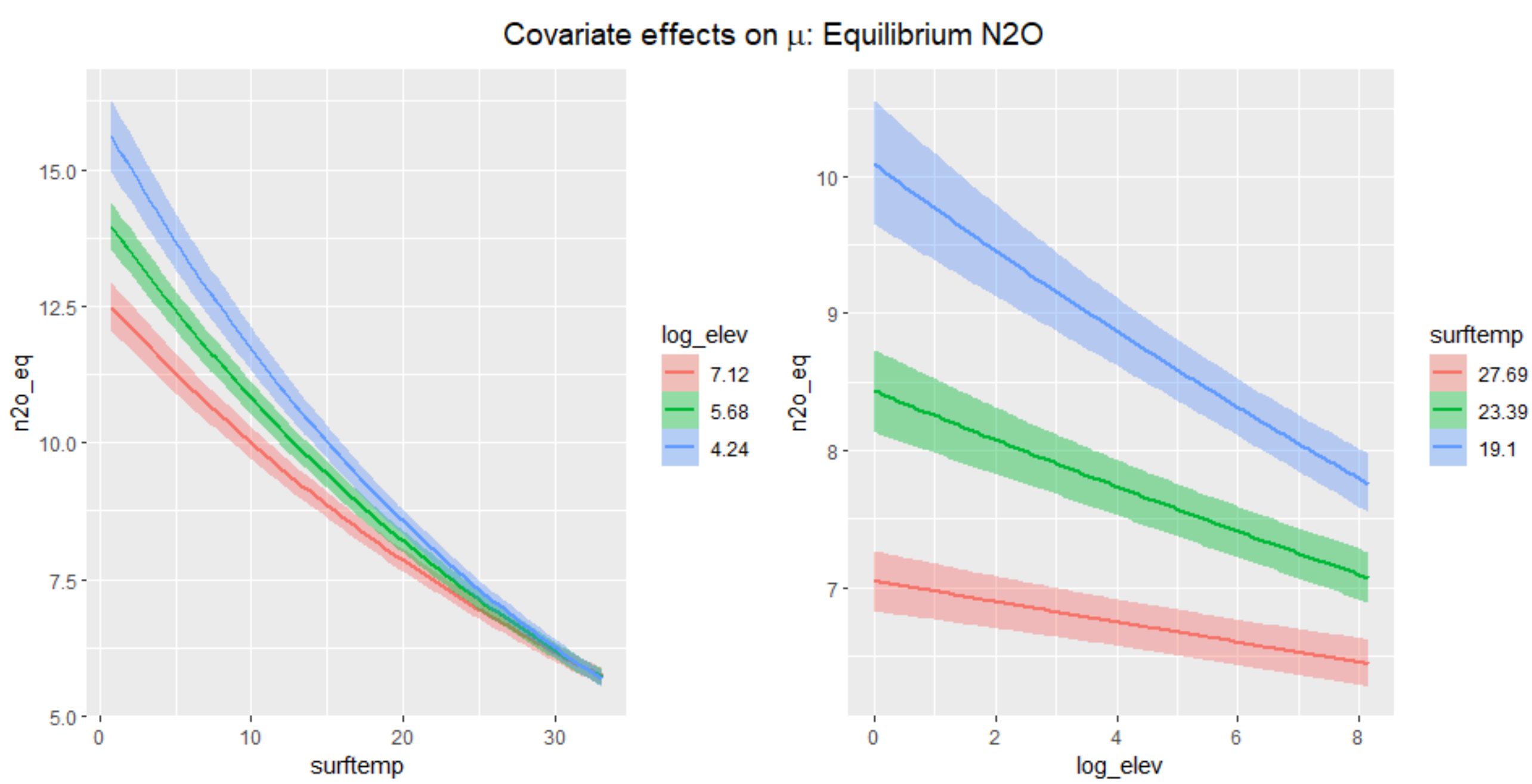

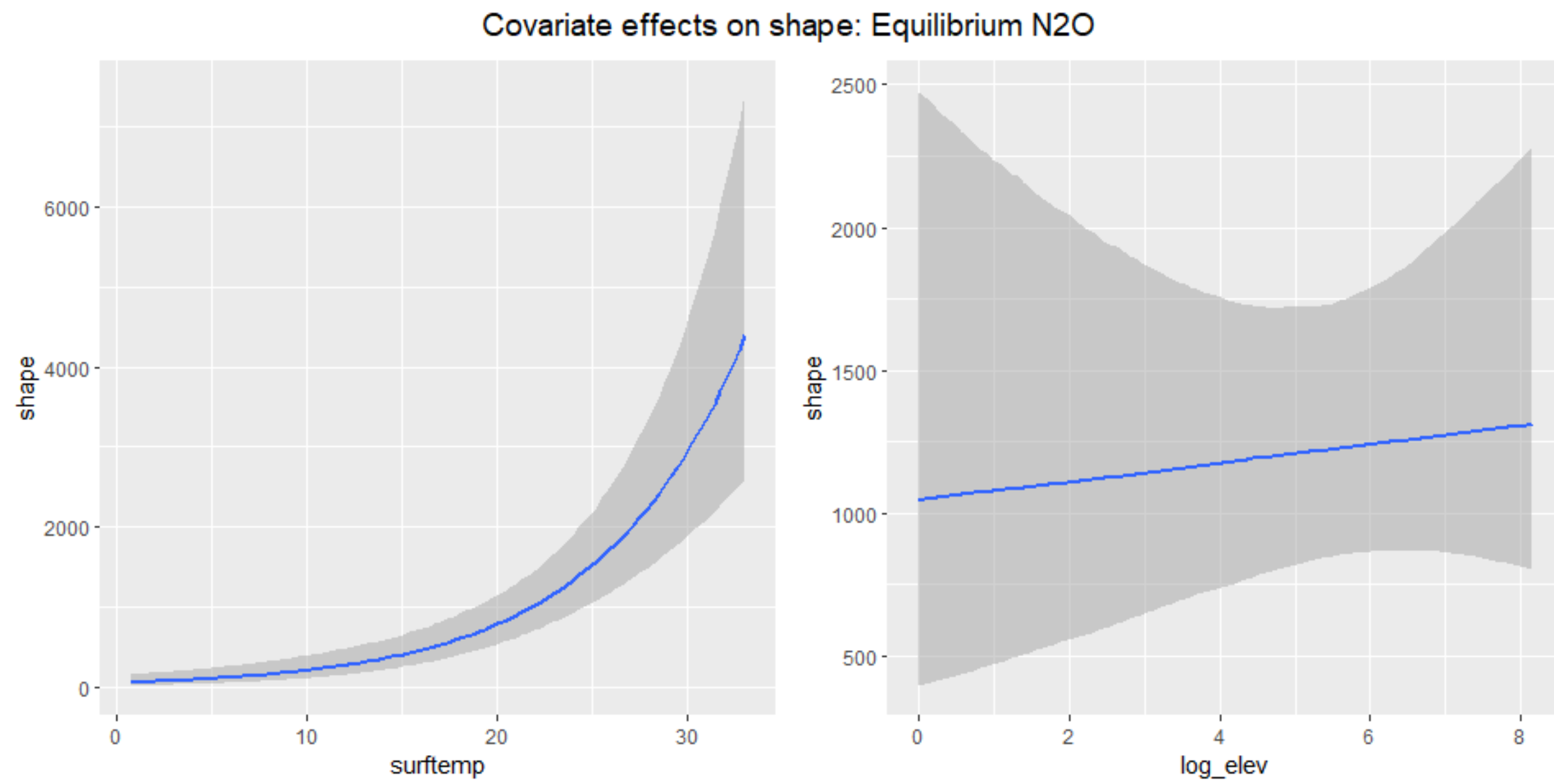

3.6.3.3 Saturation ratio

Below are the estimated conditional effects of the covariates on means for both n2o responses as well as the implied effects on the mean saturation ratio.

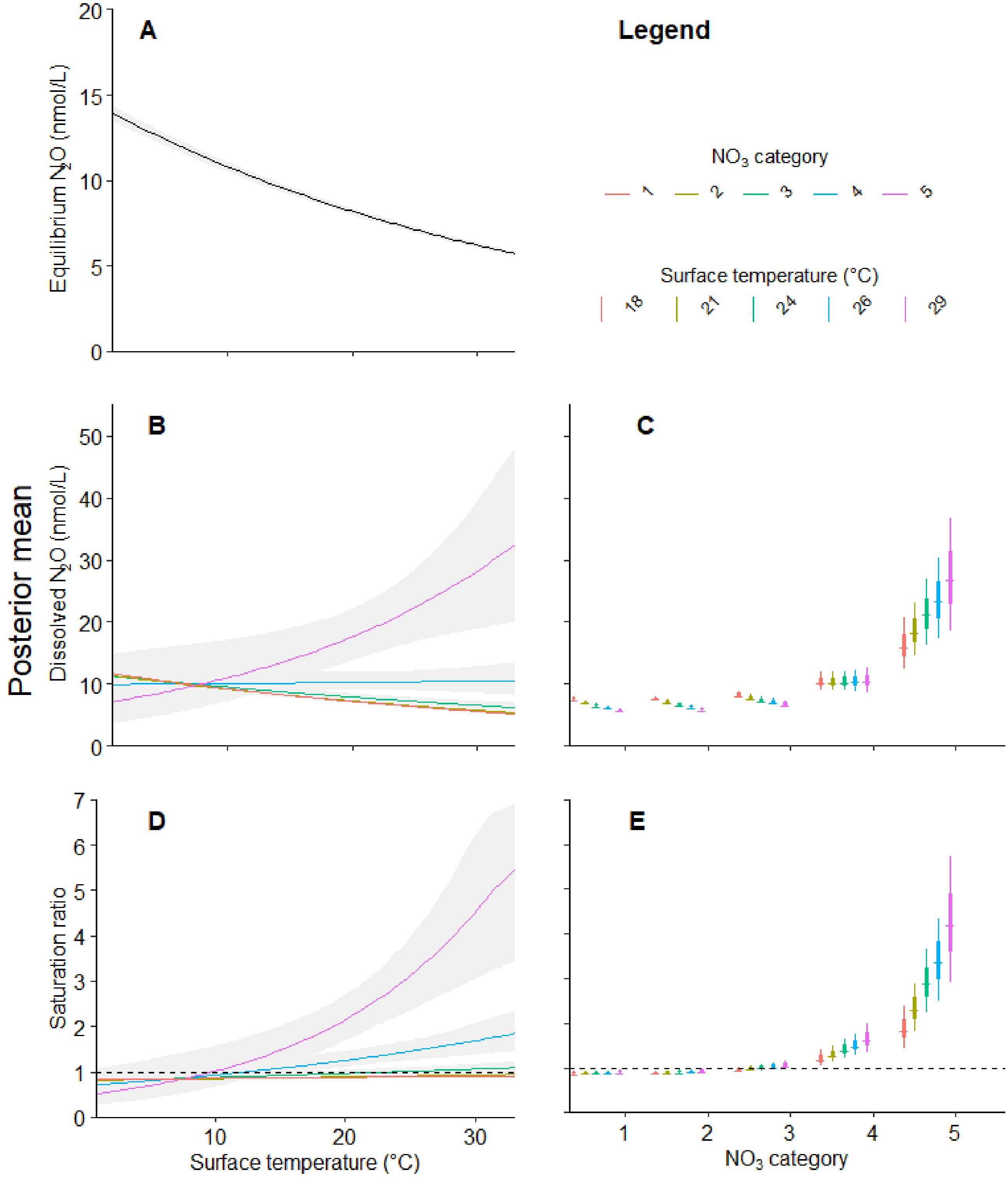


```
plt_area_effects
```

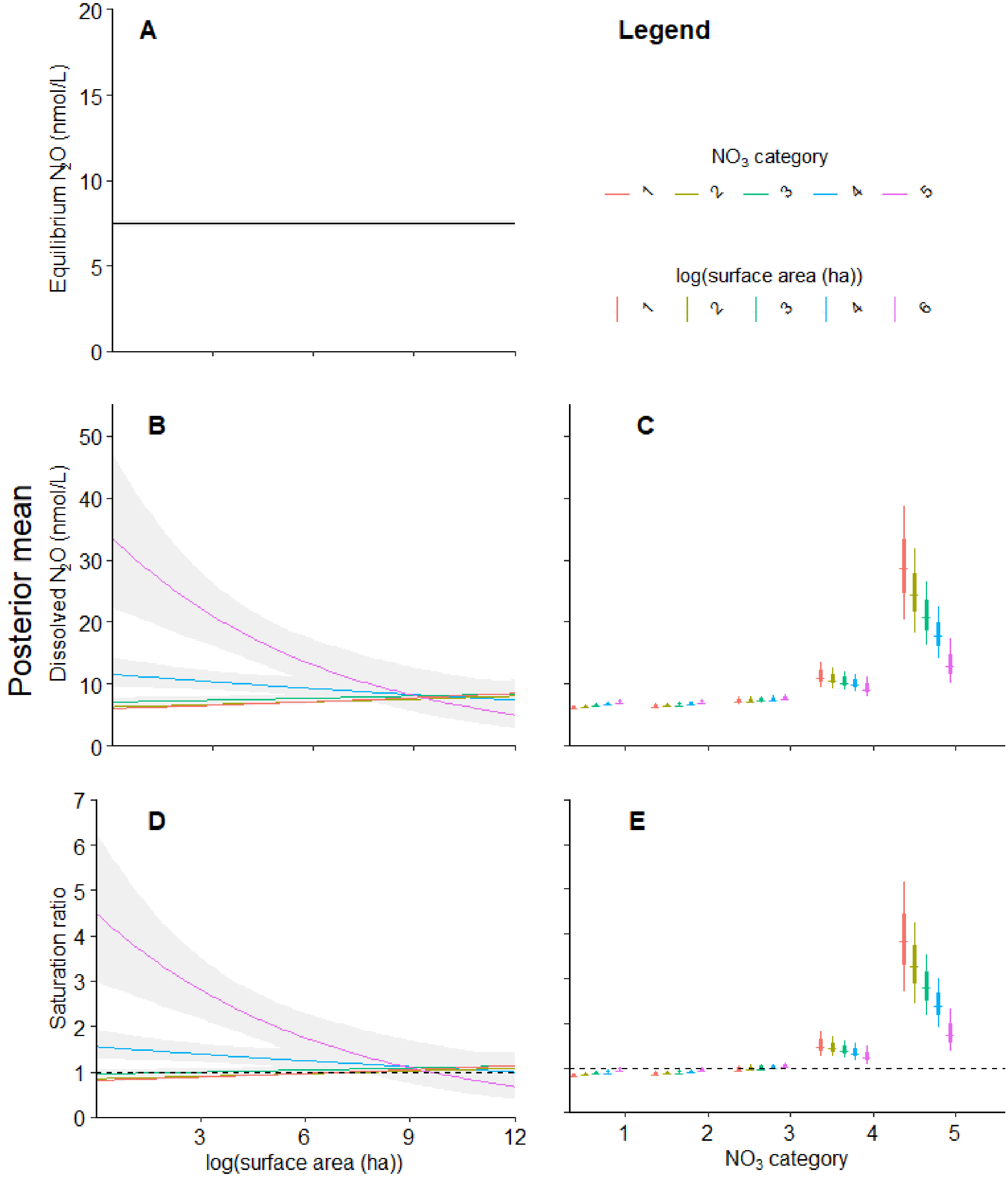


```
plt_temp_area_effects
```

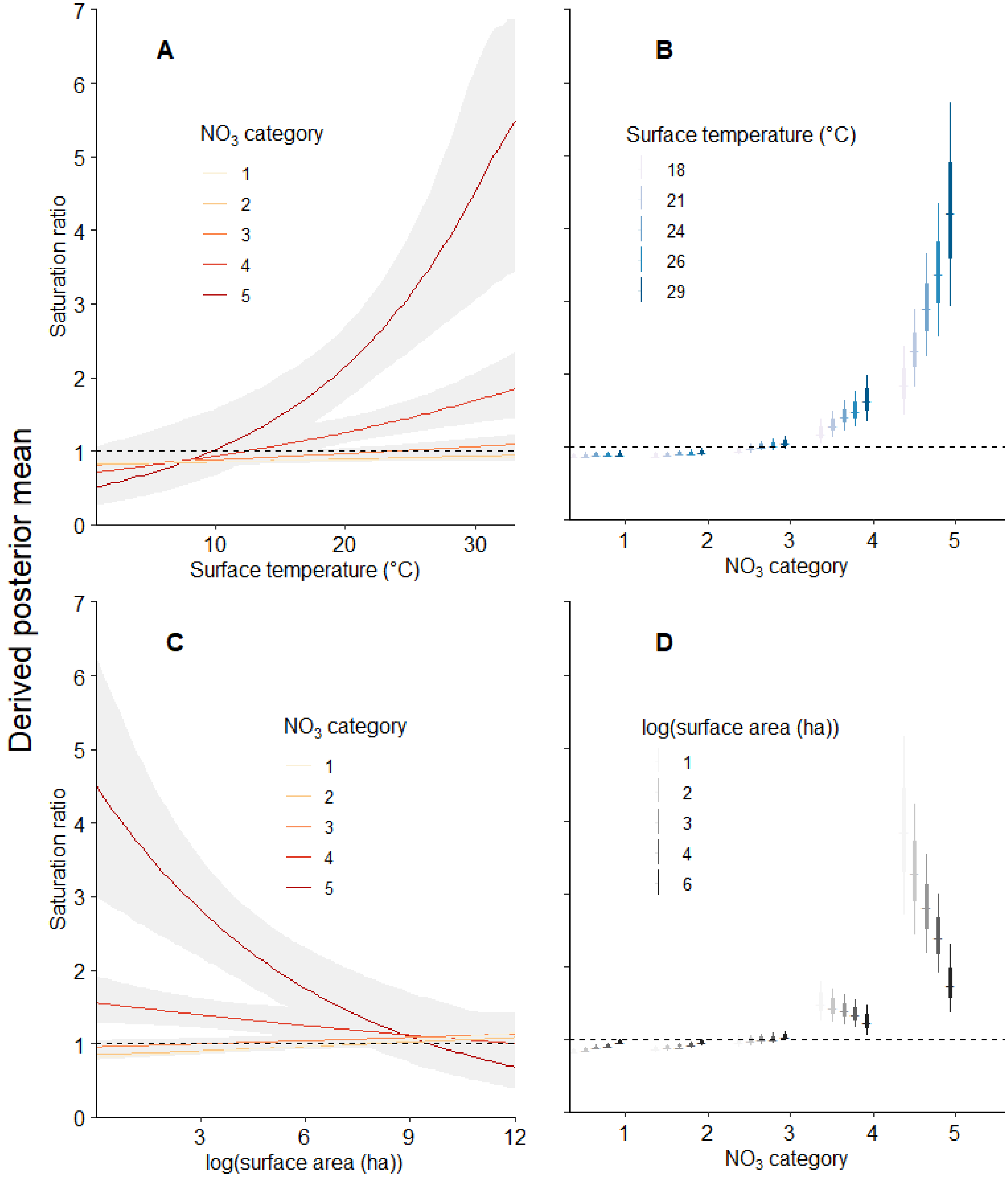

4 Predict to population

As mentioned above, in order to make inferences to the population of interest, the final model above was used to, first, predict surface temperature in the target population, since it depended only on the fully observed covariates. Next, the predictive distribution for surface temperature was used, along with the relevant fully observed covariates, to predict NO3 in

the target population. Finally, the predictive distributions for temperature and NO3 were used to predict the N2O responses. The code for these steps is outlined in the following.

The first step used the final model to predict surface temperature to the population:

```
load("C:/Users/rmartin/OneDrive - Environmental Protection Agency (EPA)/Documents/AE_
load("C:/Users/rmartin/OneDrive - Environmental Protection Agency (EPA)/Documents/AE_

predict_temp <- sframe %>%
  mutate(jdate = 205) %>%
  add_predicted_draws(n2o_mod6, resp=c("surftemp"),
                      allow_new_levels = TRUE,
                      cores =1,
                      ndraws = 2000) %>%
  mutate(surftemp = .prediction)

save(predict_temp, file = "C:/Users/rmartin/OneDrive - Environmental Protection Agenc
```

NO3 was next predicted using the posterior predictions for surface temperature and the other fully observed covariates. Note that only one posterior draw per posterior predictive draw for surface temperature was used in order to reduce excess simulations.

```
temp_X <- predict_temp %>% # select relevant columns as predictors
  ungroup() %>%
  arrange(.draw) %>%
  select(.row, .draw, WSA9, state, size_cat, log_area, log_elev, surftemp)


rm(predict_temp) # reduce memory
gc()

# set number of cores to use for parallel predictions
# and register the workers
cl <- parallel::makeCluster(25)
doSNOW::registerDoSNOW(cl)

# make a progress bar
pb <- txtProgressBar(max = 2000,
                     style = 3)
progress <- function(n) setTxtProgressBar(pb, n)
opts <- list(progress = progress)

system.time( # approx x hrs with 25 workers & 1 draw from PPD
predict_no3 <- foreach(sub_X = isplitRows(temp_X, chunkSize = 465897),
                       .combine = 'c',
                       .packages = c("brms"),
```

```
                         .options.snow = opts
                         ) %dopar% {
                           brms::posterior_predict(n2o_mod6,
                                                   newdata = sub_X,
                                                   resp = "no3cat",
                                                   allow_new_levels = T,
                                                   ndraws = 1,
                                                   cores = 1)
                         }
)

close(pb)
parallel::stopCluster(cl)

save(predict_no3, file = "C:/Users/rmartin/OneDrive - Environmental Protection Agency
```

Finally, dissolved and equilibrium N2O were predicted using the surface temperature and NO3 predictions, along with the survey variables and other fully observed covariates.

```
# re-load the temp predictions into memory
load("C:/Users/rmartin/OneDrive - Environmental Protection Agency (EPA)/Documents/AE_

# Assemble dataframe containing relevant covariates (known and predicted) for predict
n2o_X <- predict_temp %>%
  ungroup() %>%
  mutate(no3_cat = predict_no3) %>%
  select(WSA9,
         state,
         size_cat,
         log_area,
         surftemp,
         log_elev,
         no3_cat)

rm(predict_temp, predict_no3) # reduce memory

# set number of cores to use for parallel predictions
# and register the workers
cl <- parallel::makeCluster(25)
doSNOW::registerDoSNOW(cl)

# make a progress bar
pb <- txtProgressBar(max = 2000, style = 3)
progress <- function(n) setTxtProgressBar(pb, n)
opts <- list(progress = progress)

# make predictions in parallel
```

```
system.time(
predict_n2o <- foreach(sub_X = isplitRows(n2o_X, chunkSize = 465897),
                 .combine = rbind,
                 .options.snow = opts,
                 .packages = c("brms")) %dopar% {
  apply(posterior_predict(n2o_mod6,
                          newdata = sub_X,
                          resp = c("n2o", "n2oeq"),
                          allow_new_levels = T,
                          ndraws = 1,
                          cores = 1), 3, t)
                   }
)

close(pb)
parallel::stopCluster(cl)

colnames(predict_n2o) <- c("n2o", "n2oeq")

save(predict_n2o, file = "C:/Users/rmartin/OneDrive - Environmental Protection Agency
```

Finally, the predictions for all four partially observed responses were assembled into a new dataframe for use in inference:

```
load("C:/Users/rmartin/OneDrive - Environmental Protection Agency (EPA)/Documents/AE_
load("C:/Users/rmartin/OneDrive - Environmental Protection Agency (EPA)/Documents/AE_
load("C:/Users/rmartin/OneDrive - Environmental Protection Agency (EPA)/Documents/AE_

all_predictions <- predict_temp %>%
  ungroup() %>%
  mutate(no3cat = predict_no3) %>%
  bind_cols(predict_n2o) %>%
  mutate(n2osat = n2o / n2oeq, # calculate saturation ratio
         .row = rep(1:465897, each = 500),
         .draw = rep(seq(1,500, 1), 465897)) %>%
  mutate(area_ha = exp(log_area)) %>% # include area on ha scale
  select(WSA9,
         state,
         size_cat,
         area_ha,
         lat,
         lon,
         .row,
         .draw,
         surftemp,
         no3cat,
         n2o,
```

```
          n2oeq,
          n2osat)

save(all_predictions, file = "C:/Users/rmartin/OneDrive - Environmental Protection Ag
```

5 Population estimates

A number of estimates for the target population were assembled and presented below. First, the full posterior predictive distributions for dissolved N2O, equilibrium N2O, and the saturation ratio were assessed. These distributions summarized the predicted distribution of concentrations or ratios for all lakes in the population of interest and included parameter uncertainty propagated through the model. Next, population means were assessed, followed by comparisons of some model-based estimates to previously calculated design-based estimates.

5.1 Posterior predictive distributions

Below, a density plot summarized the posterior predictive distributions across the target population of lakes. The summaries of the PPDs were based on 2000 draws. Note that the x-axis was truncated at 50 nmol/L for a clearer visualization of the bulk of the predictive distribution. For reference, the max predicted value was 4403.2 nmol/L for dissolved N2O, 20.4 nmol/L for dissolved N2O, and 793.5 for the saturation ratio.

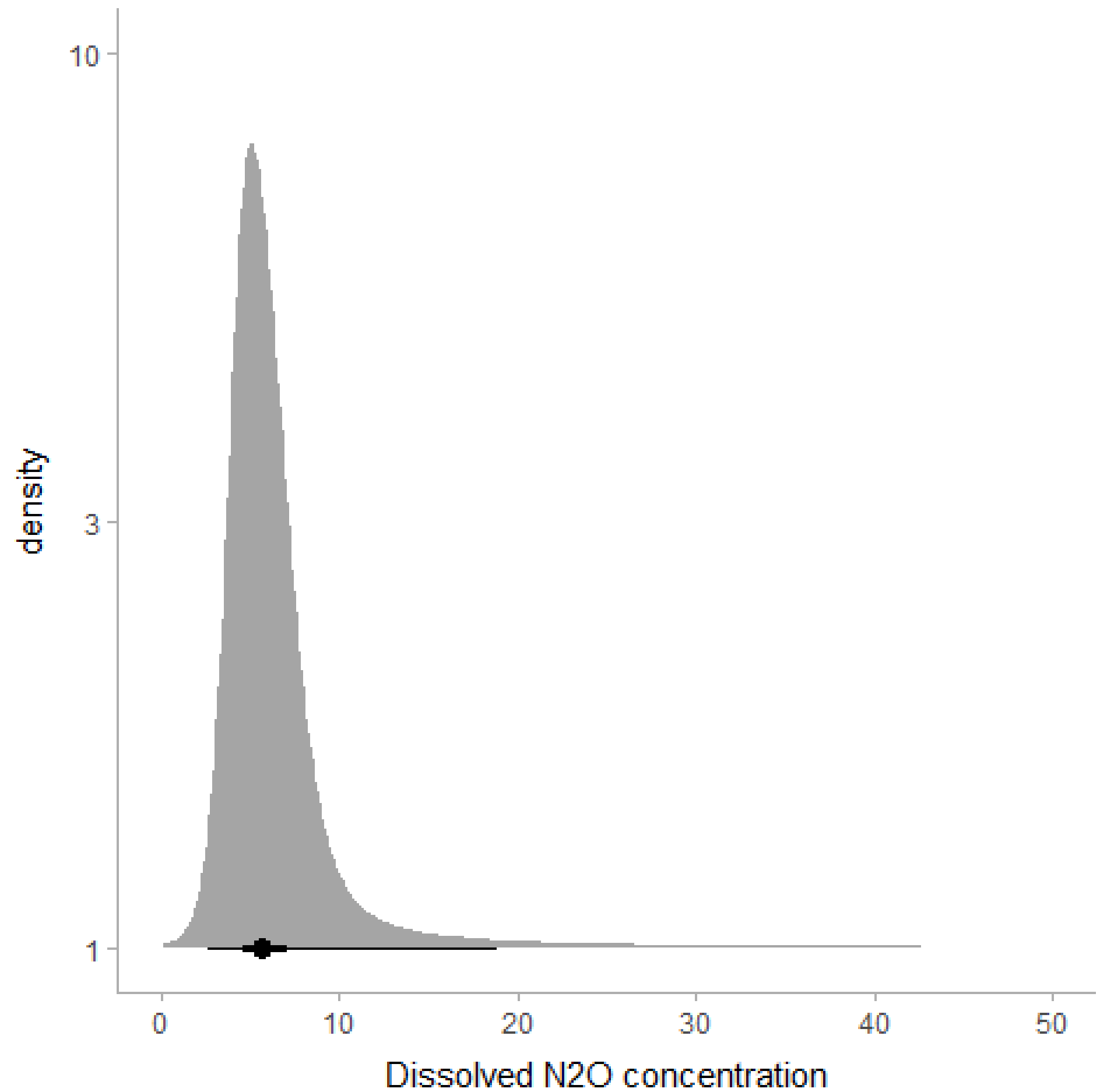
density
10
3
1
0
10
20
30
40
50
Dissolved N2O concentration

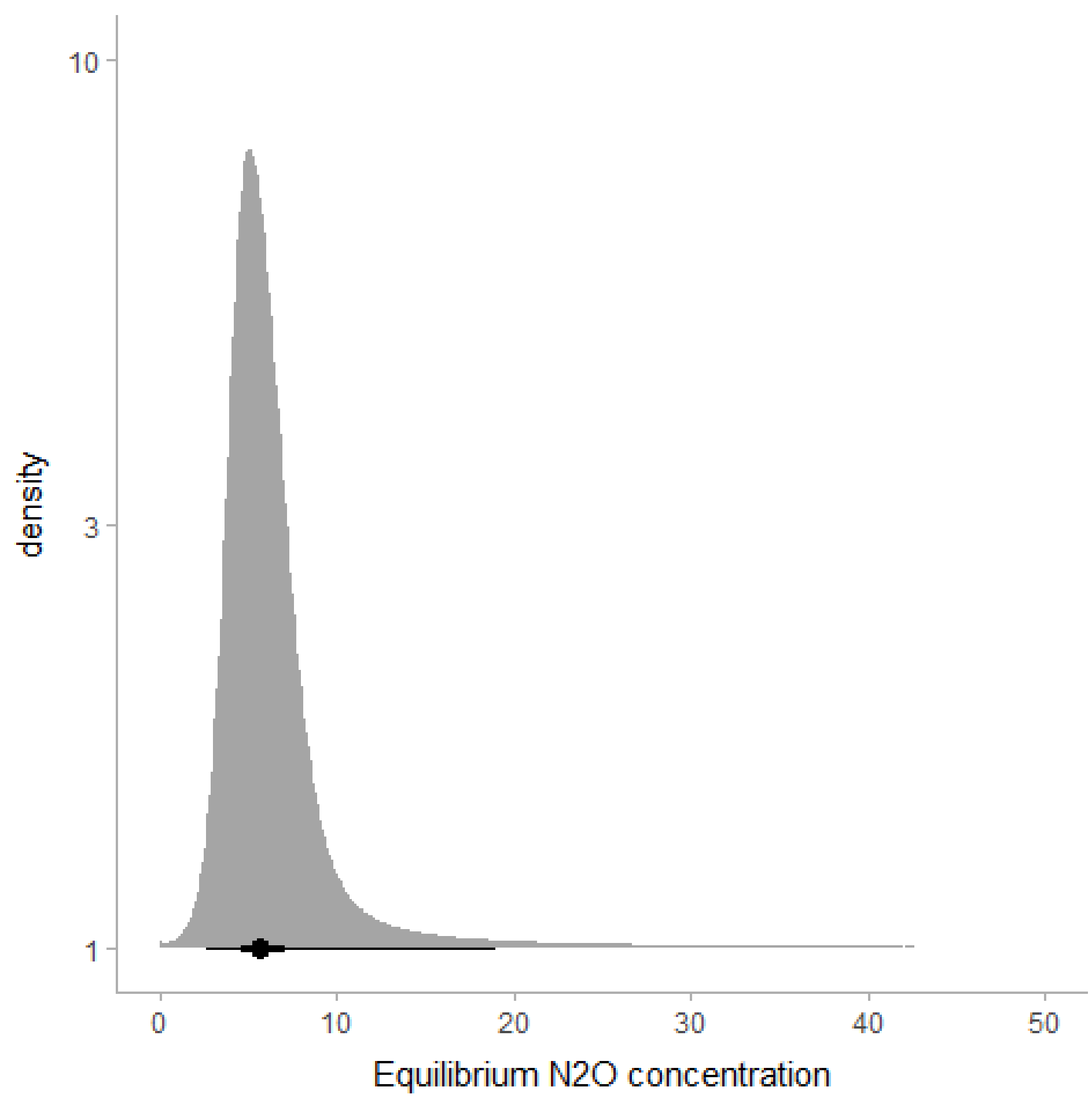
density
10
3
1
0
10
20
30
40
50
Equilibrium N2O concentration

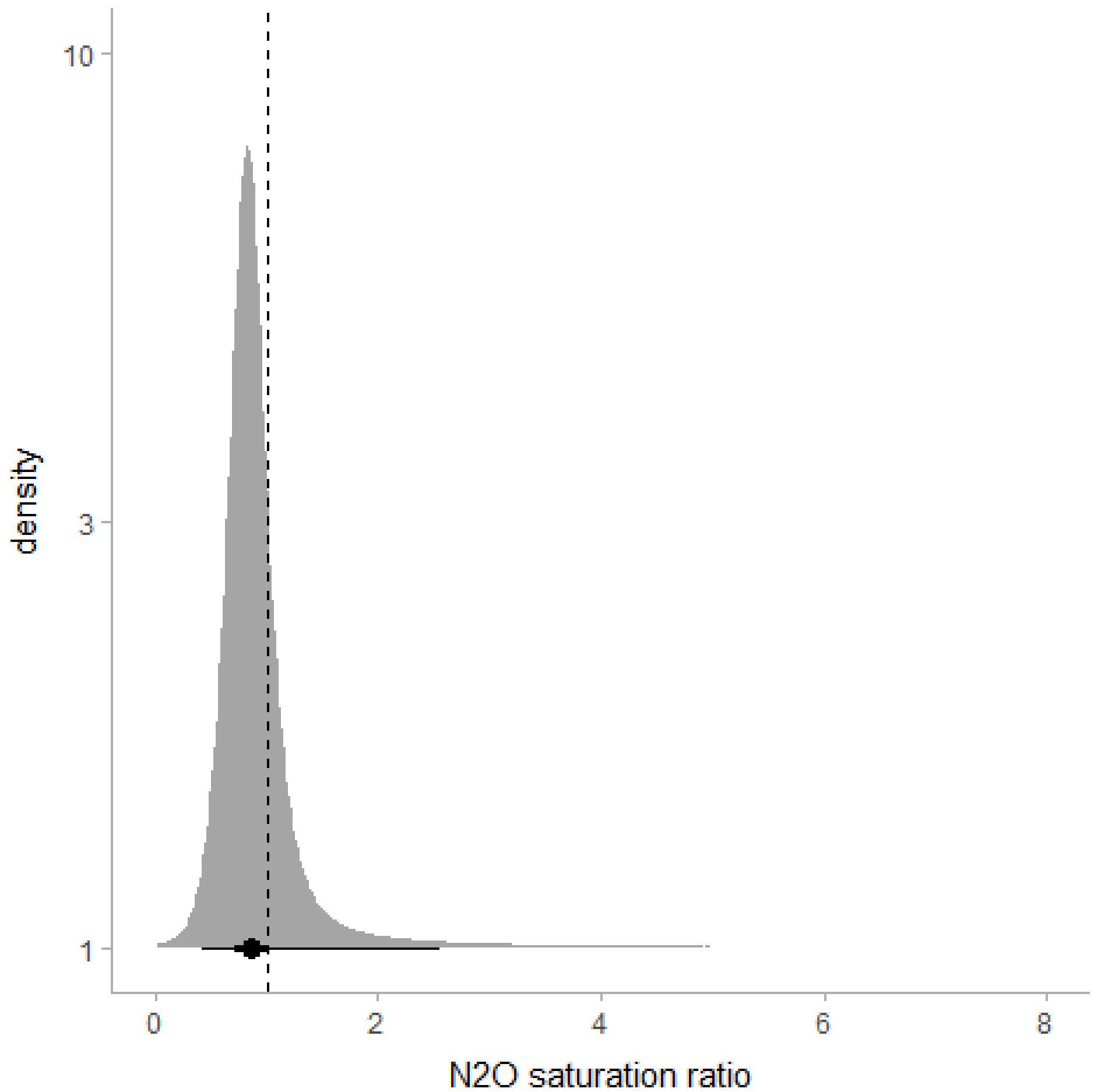

5.2 Estimated means

5.2.1 National

Below are density plots summarizing the posterior distribution of *means* for N2O concentrations and the saturation ratio for all US lakes and reservoirs > 4ha in the lower 48 states).

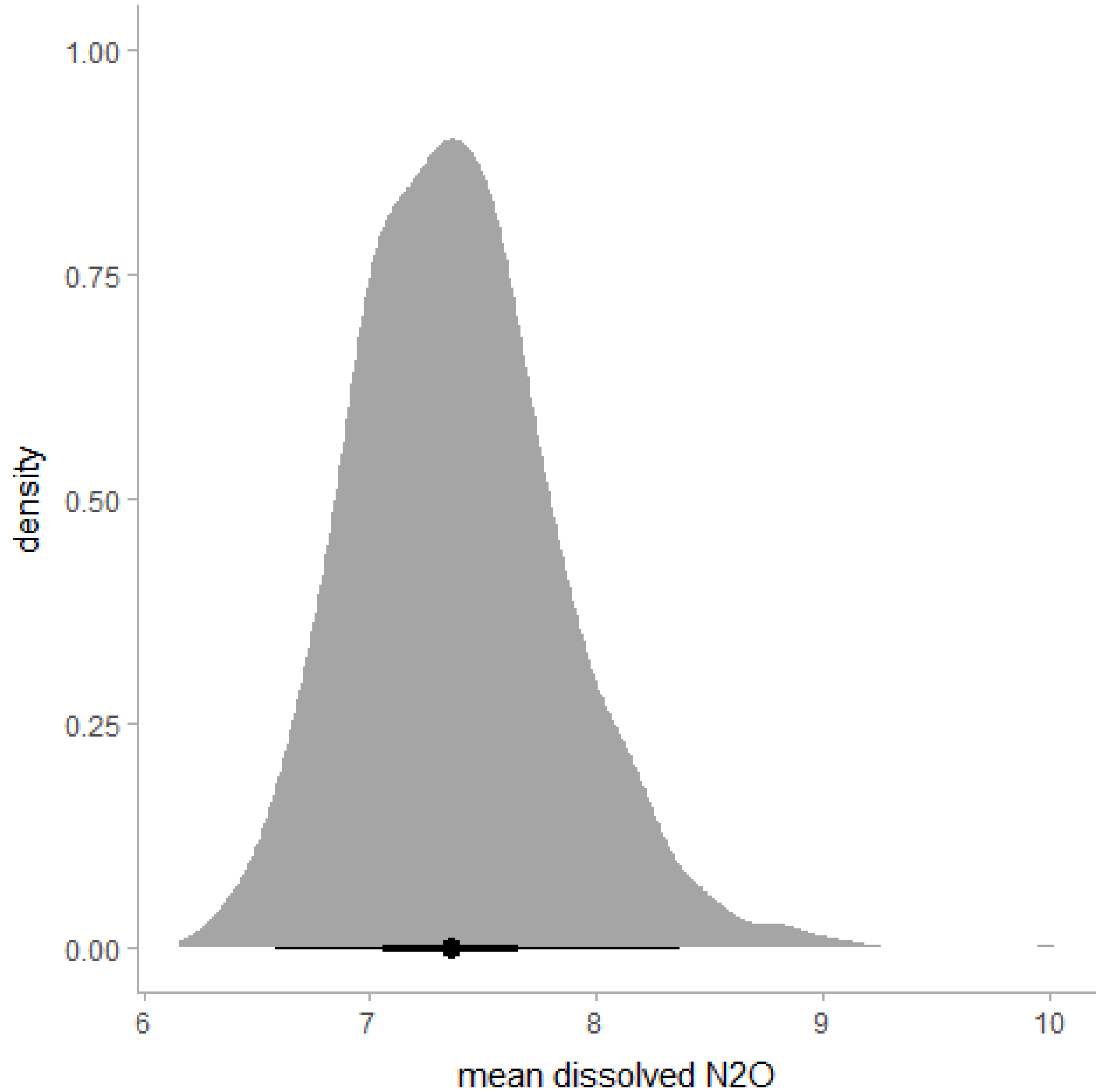
density
1.00
0.75
0.50
0.25
0.00
6
7
8
9
10
mean dissolved N2O

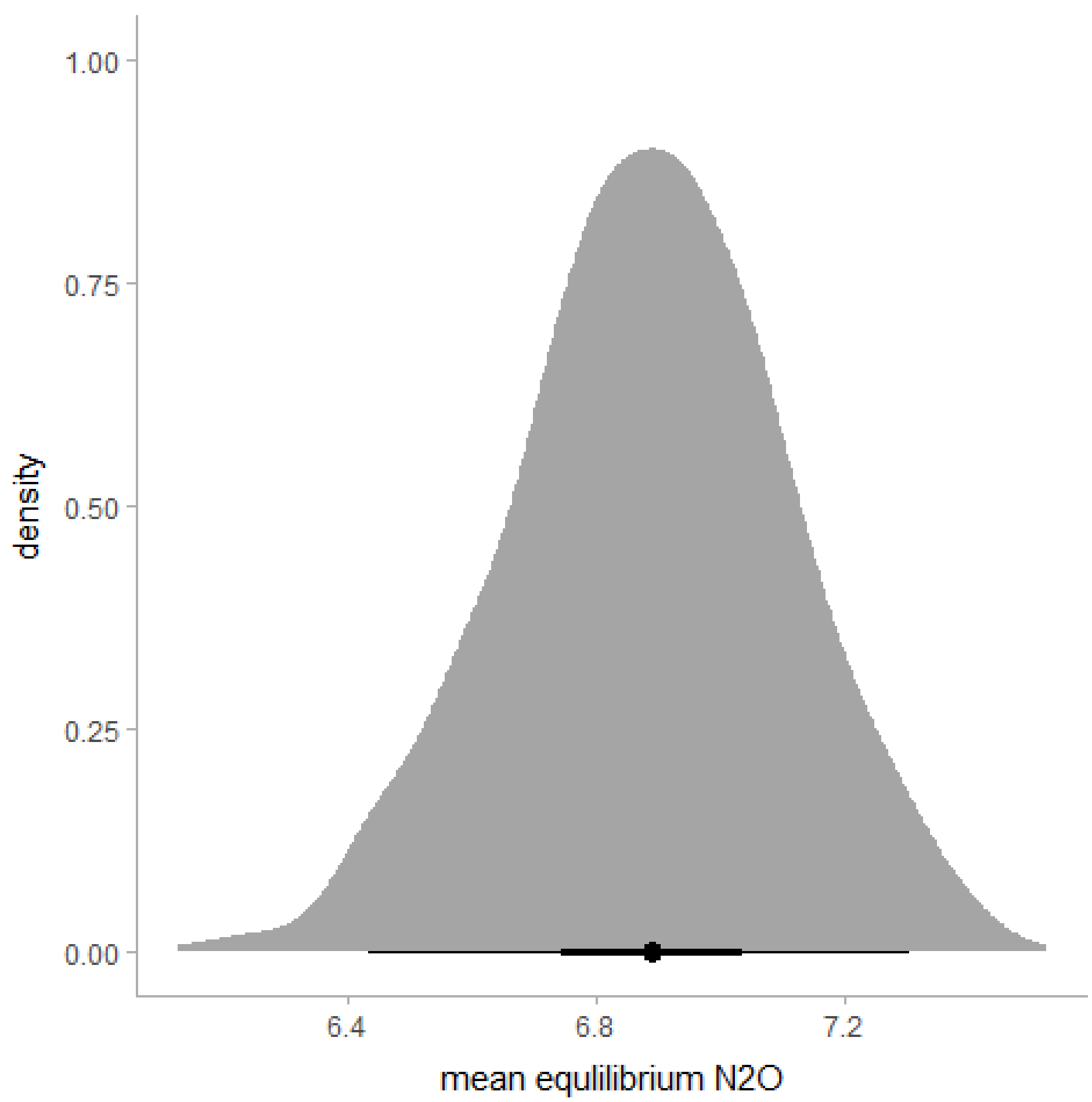
density
1.00
0.75
0.50
0.25
0.00
6.4
6.8
7.2
mean equlilibrium N2O

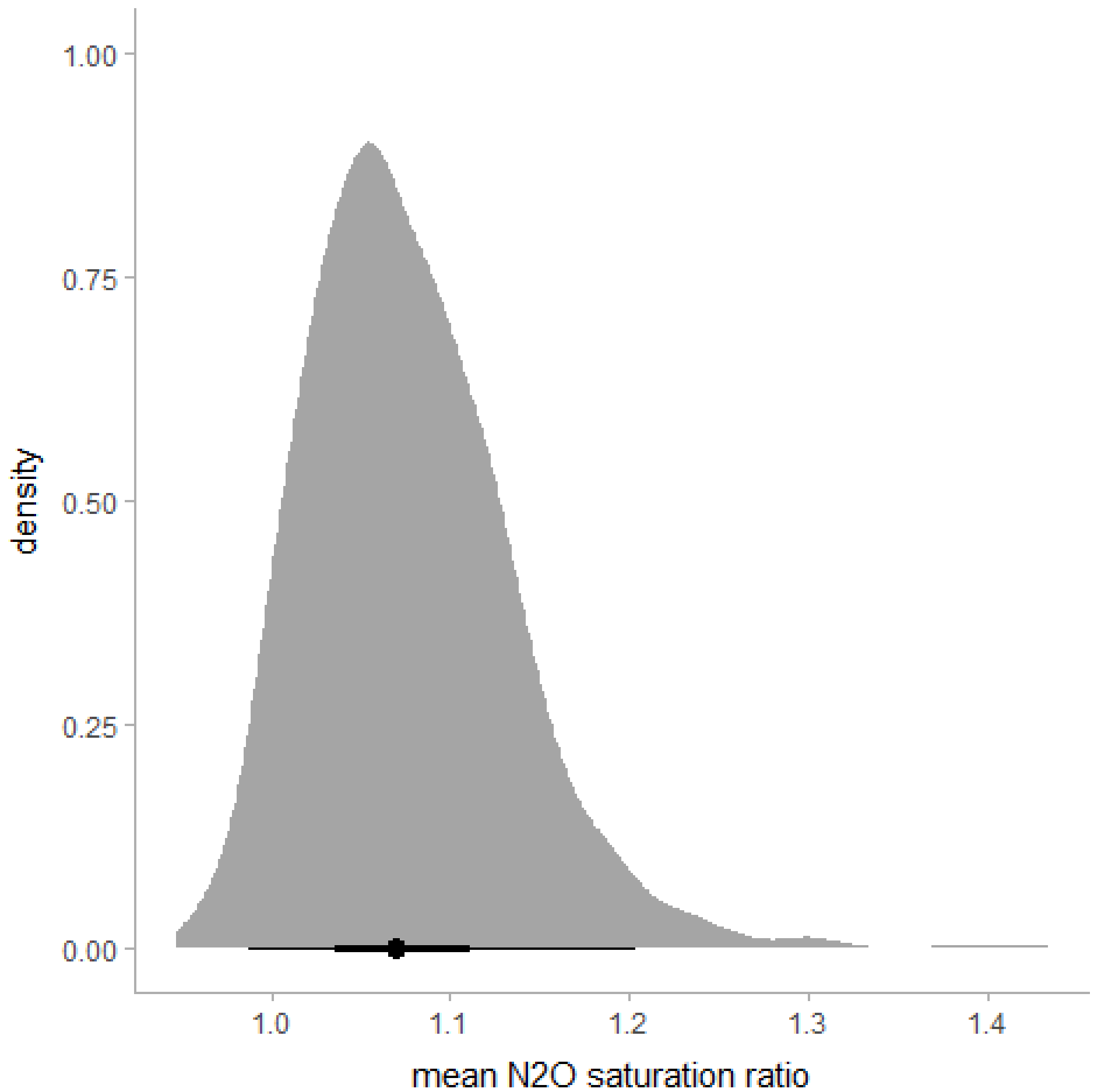

To illustrate the skewness in the predictive distribution for the saturation ratio, an estimate for the median ratio is shown below. The entire posterior distribution of the mean above was larger than 1, which represents the boundary of under- *vs.* oversaturation. By comparison, the posterior estimate of the median below only included values less than one, suggesting that though the mean saturation ratio was likely greater than 1, most lakes in the national population were undersaturated (i.e., ratio less than 1). In distributions with strong right-skew, the mean can often be considerably larger than the median.

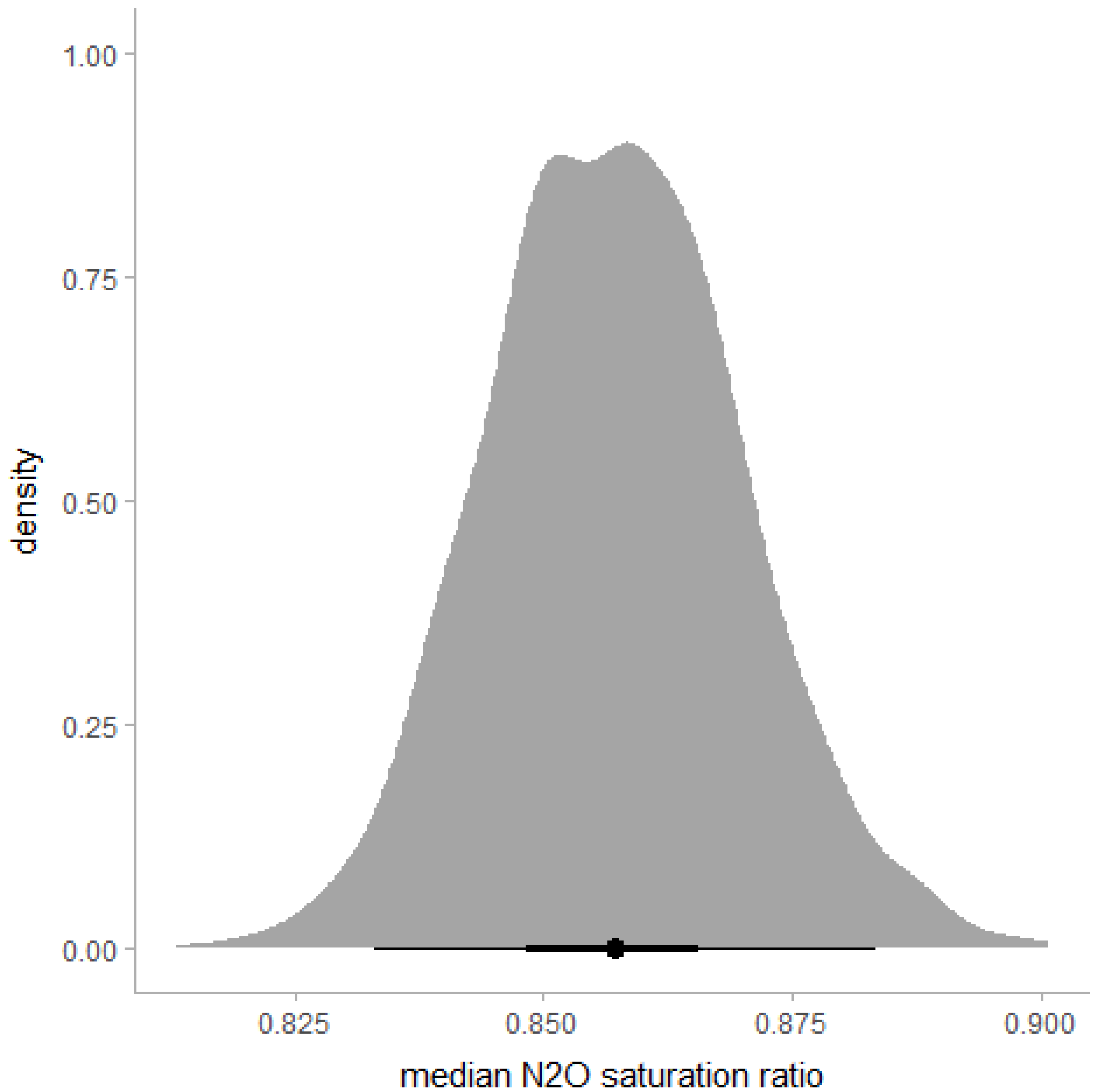

Below is a plot of the posterior mean estimate for the proportion of unsaturated lakes at the national scale.

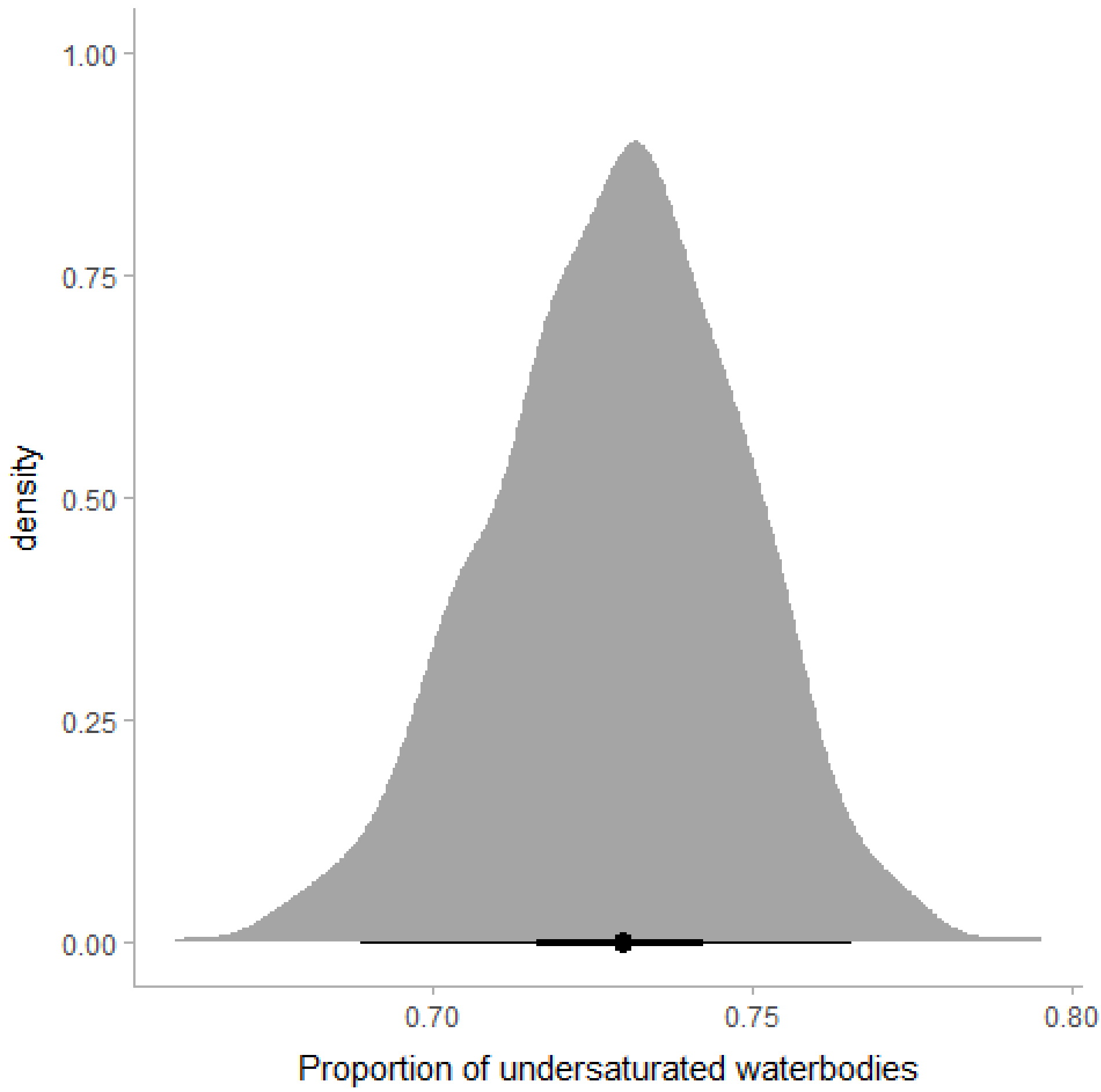

5.2.2 Ecoregion

Below are posterior estimates of the means for dissolved and equilibrium N2O and the saturation ratio by WSA9 ecoregion.

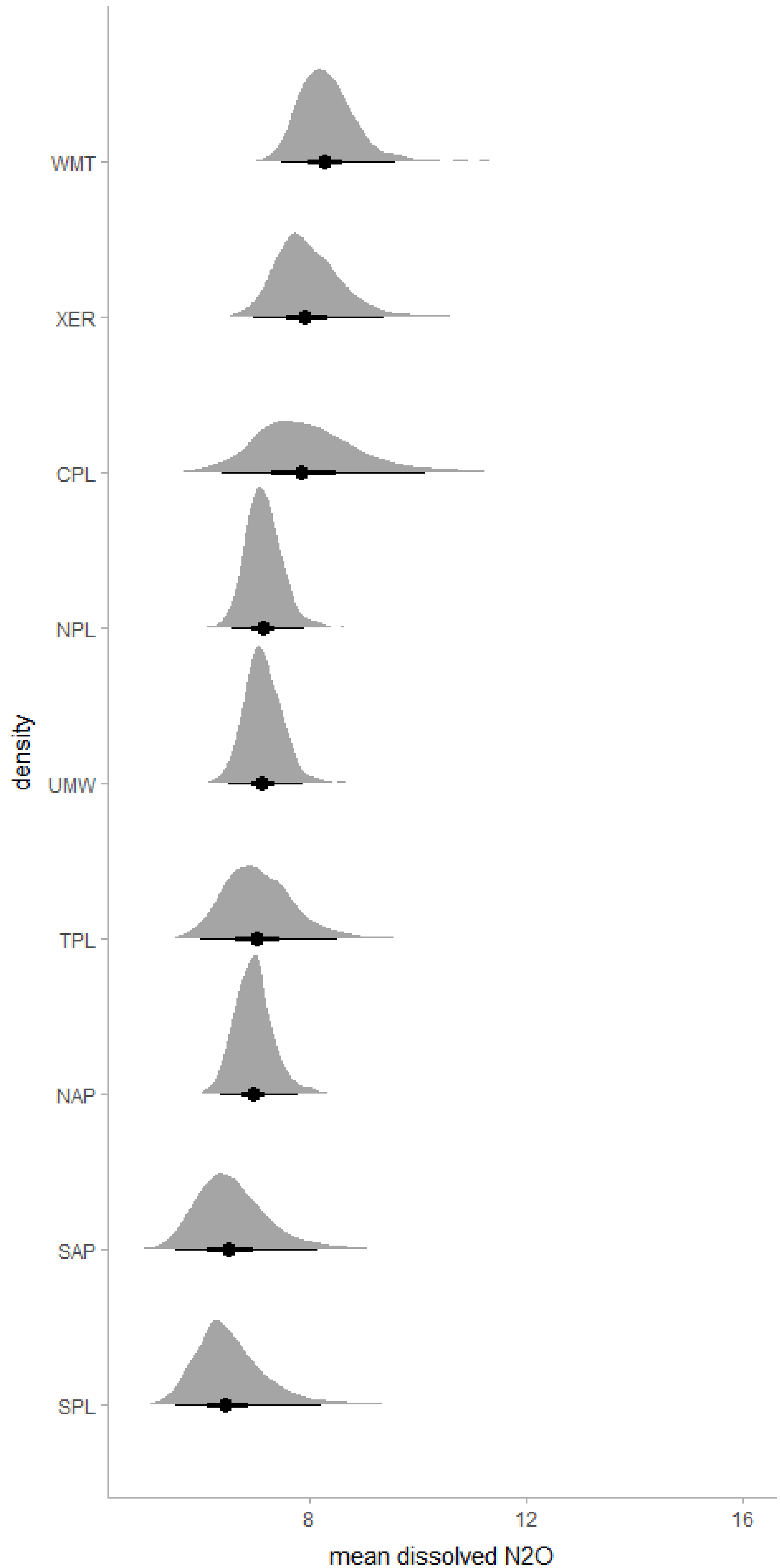
WMT
XER
CPL
NPL
UMW
TPL
NAP
SAP
SPL
density
8
12
16
mean dissolved N2O

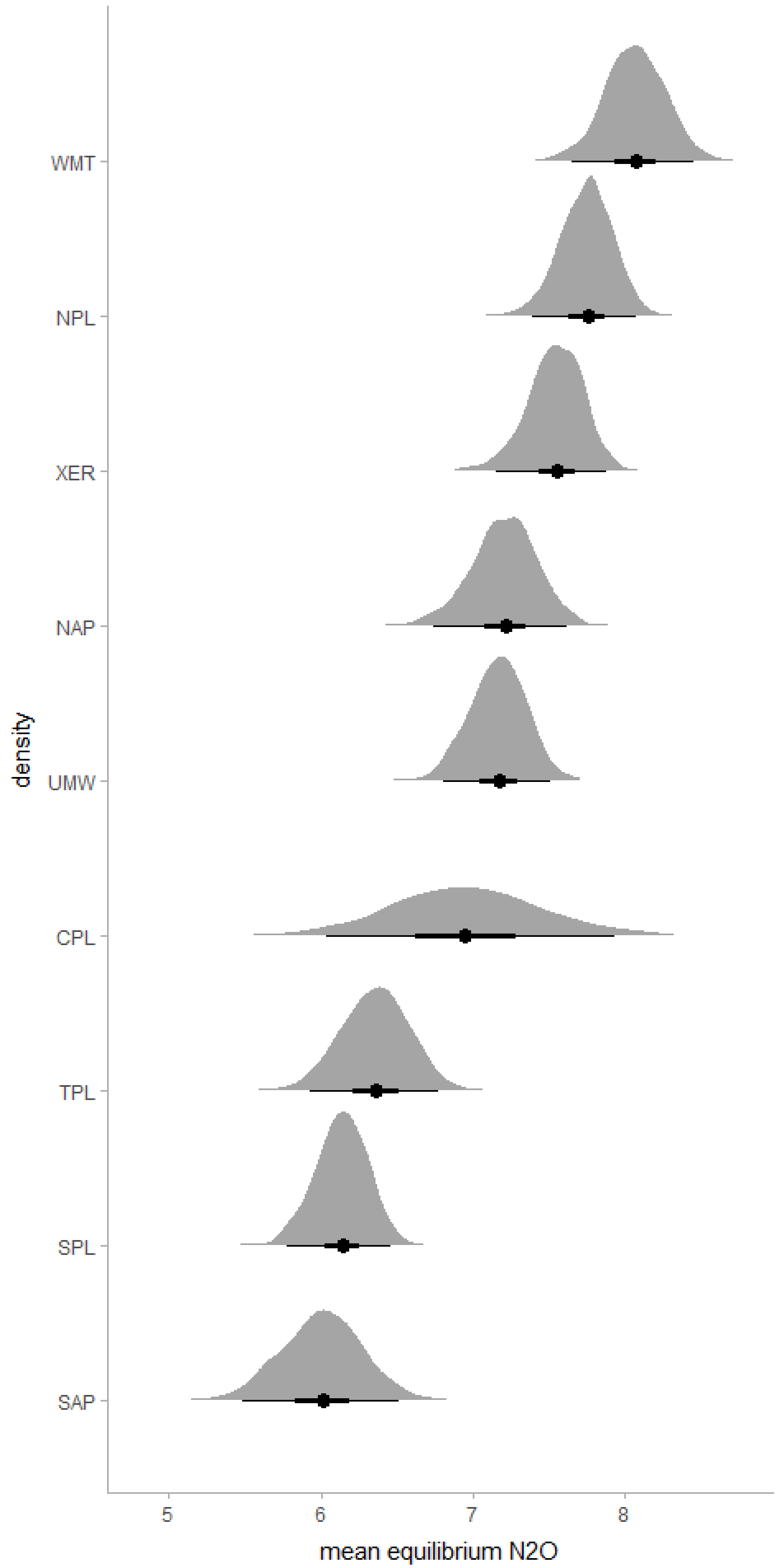

WMT
NPL
XER
NAP
UMW
CPL
TPL
SPL
SAP
density
5
6
7
8
mean equilibrium N2O

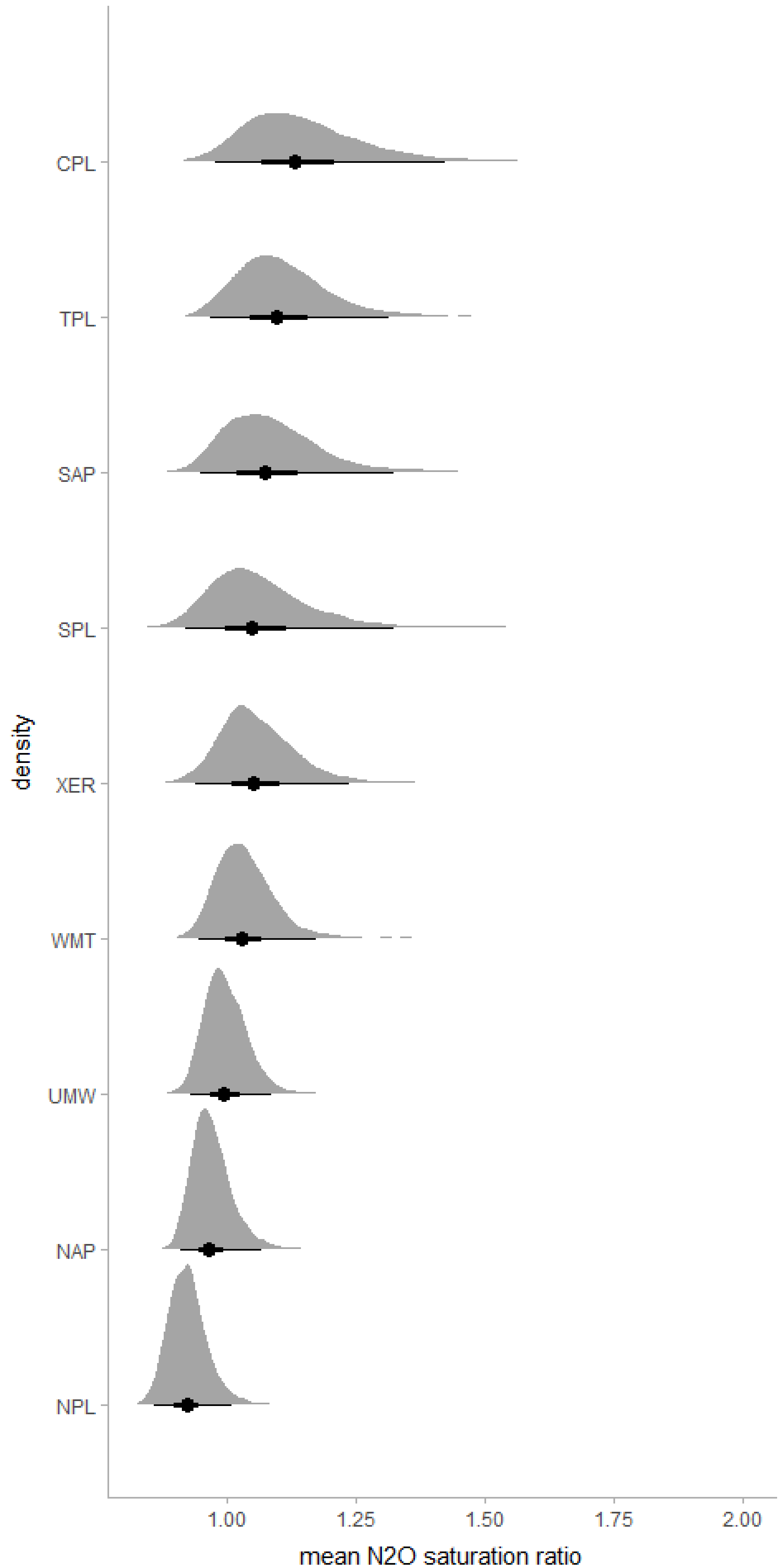

density
CPL
TPL
SAP
SPL
XER
WMT
UMW
NAP
NPL
1.00
1.25
1.50
1.75
2.00
mean N2O saturation ratio

A plot of the posterior estimates for the median saturation ratio below indicated that most lakes in each ecoregion were undersaturated (i.e., median < 1).

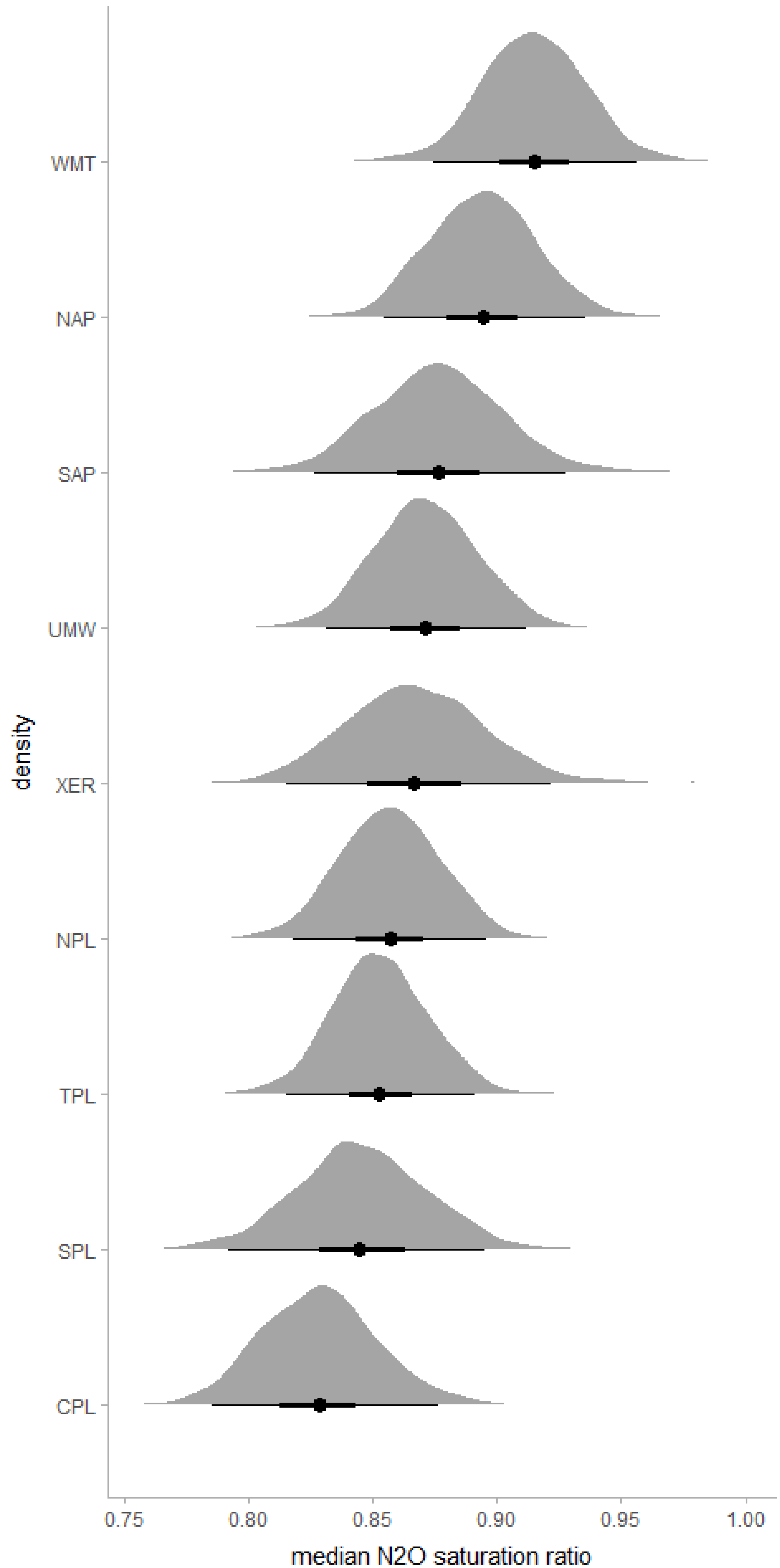

WMT
NAP
SAP
UMW
XER
NPL
TPL
SPL
CPL
density
0.75
0.80
0.85
0.90
0.95
1.00
median N2O saturation ratio

A plot of the estimates of the proportion of under-saturated lakes by ecoregion is below. These summaries again suggested that most lakes in each ecoregion were likely undersaturated (i.e., median << 1).

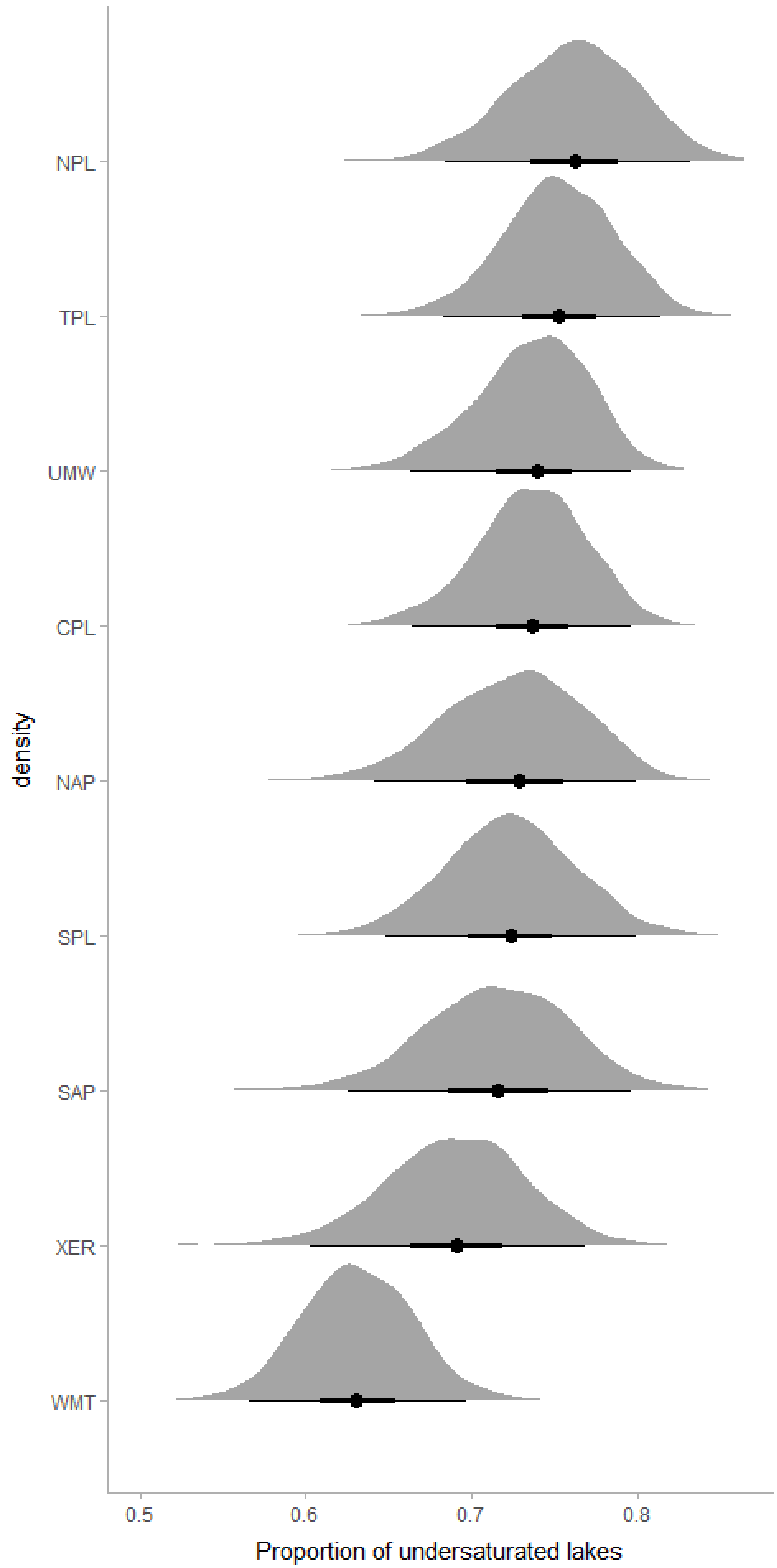
NPL
TPL
UMW
CPL
NAP
SPL
SAP
XER
WMT
density
0.5
0.6
0.7
0.8
Proportion of undersaturated lakes

5.2.3 State

Comparisons of mean estimates by state are below. Density polygons were not included to minimize the vertical plot space.

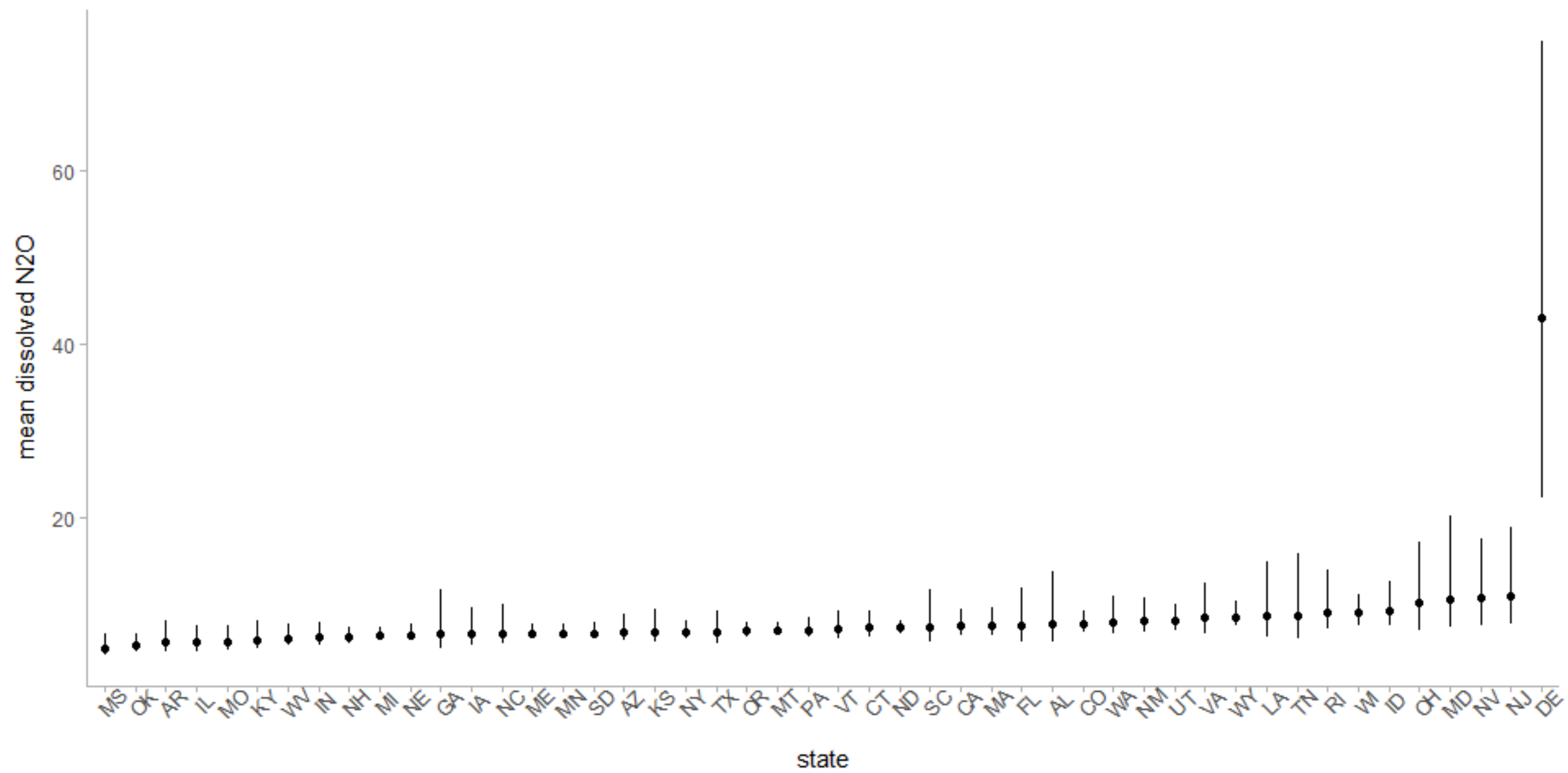

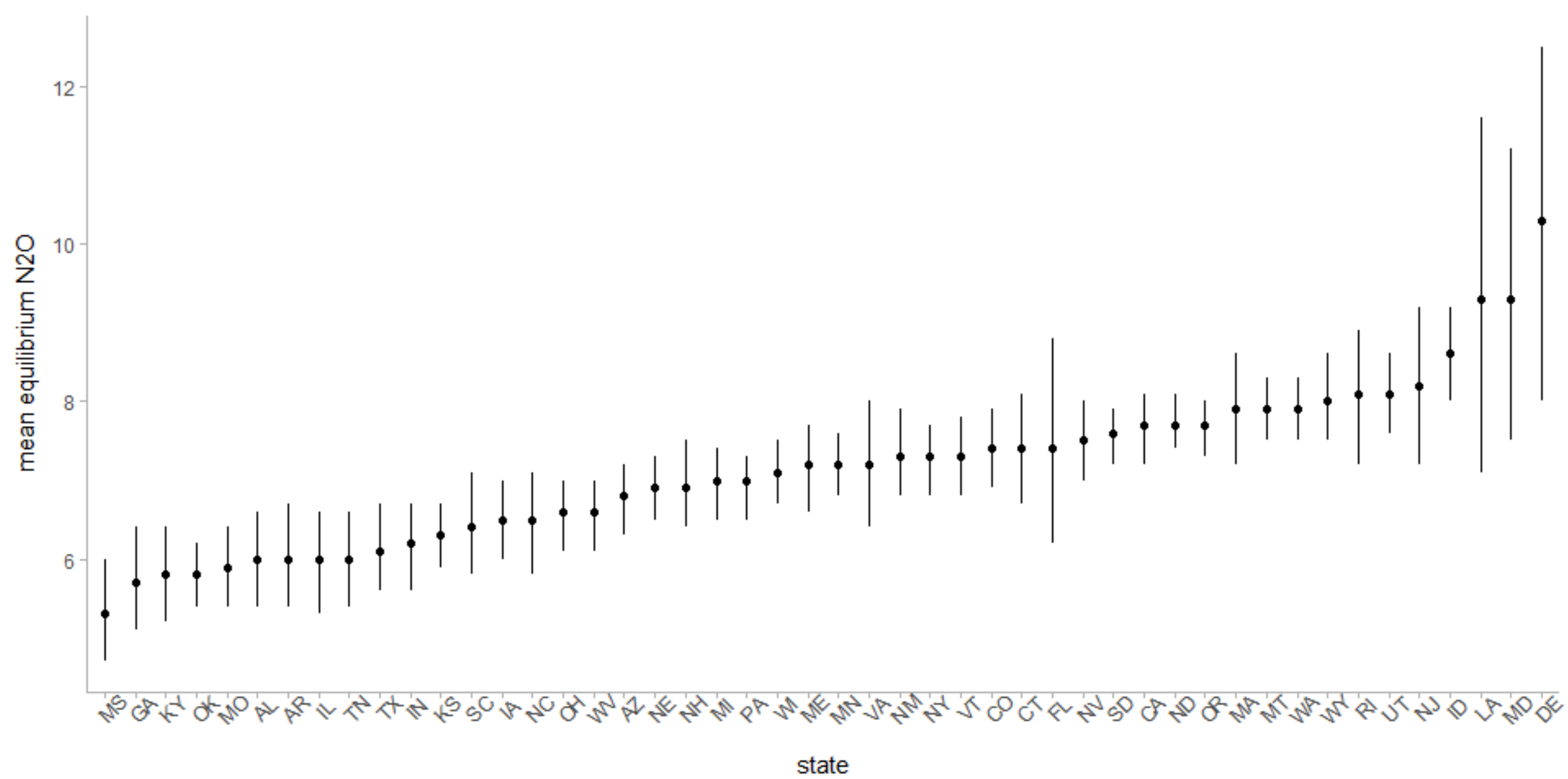

Below is a plot of estimates for the mean (black circles) and median (grey circles) saturation ratio by state. A horizontal, dashed, black line is shown at ratio = 1, indicating the boundary for under- *vs.* oversaturation. Only a few states (e.g., NV, DE) had median estimates that were 1 or greater, suggesting that, for most states, most lakes were estimated to be undersaturated.

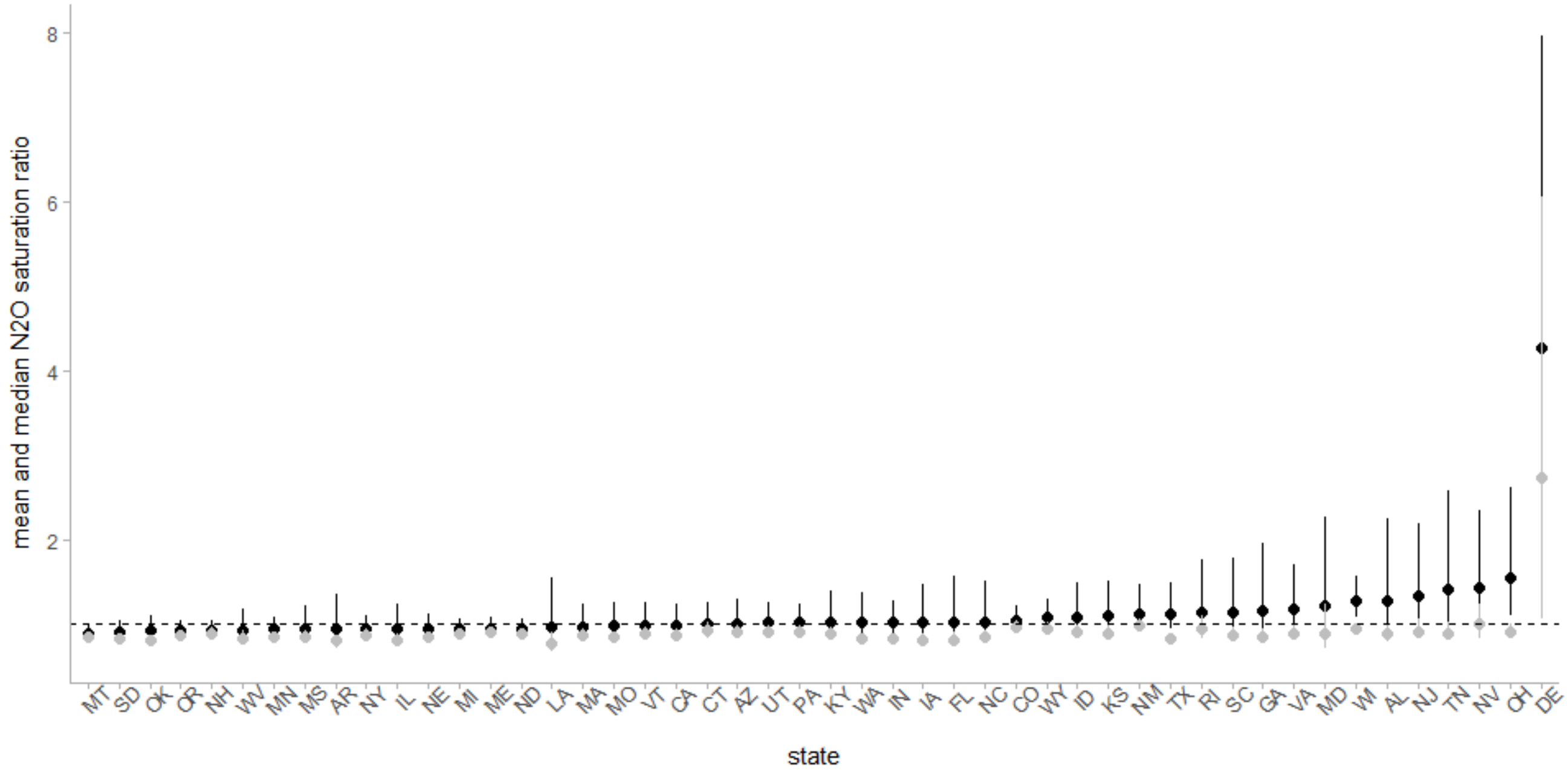

Finally, a plot of the estimated proportion of undersaturated lakes for each state in the target population. Point estimates are the posterior median of the proportion and bars are the upper and lower boundaries of the central 95th percentile of the posterior distributions of proportions.

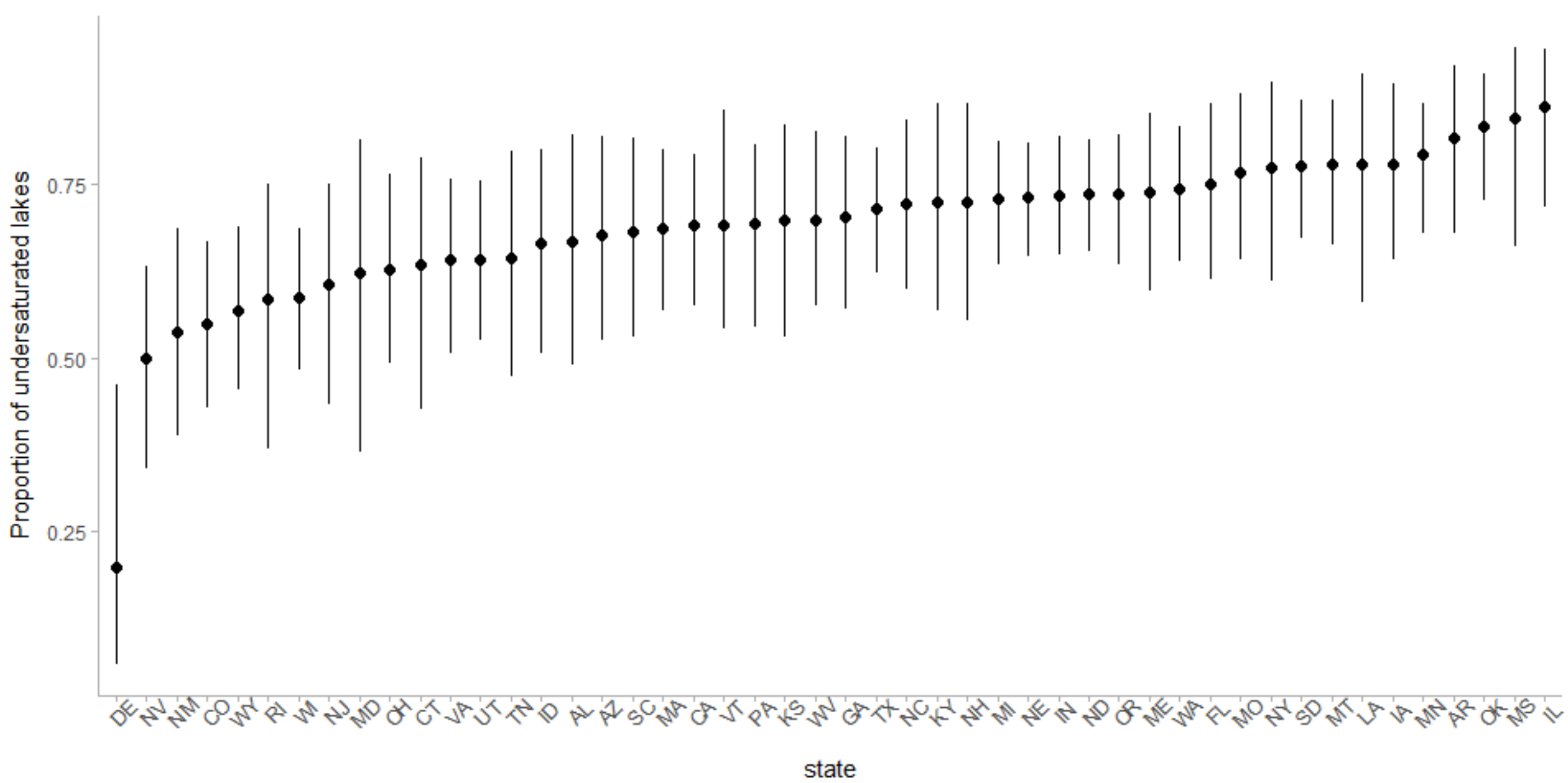

5.2.4 Size category

The estimated means and medians by size category are below for dissolved and equilibrium N2O and the saturation ratio.

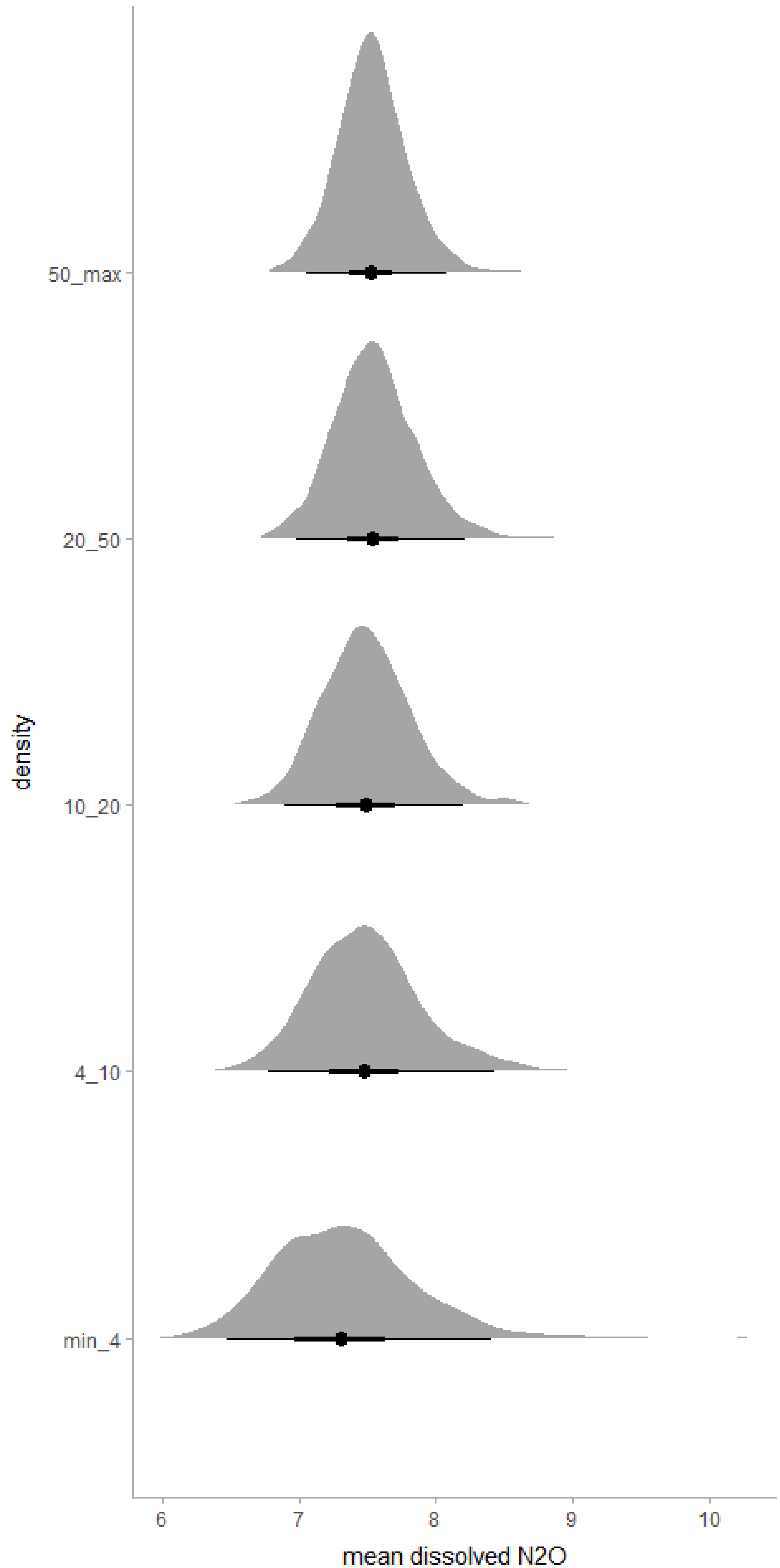
50_max
20_50
10_20
4_10
min_4
density
6
7
8
9
10
mean dissolved N2O

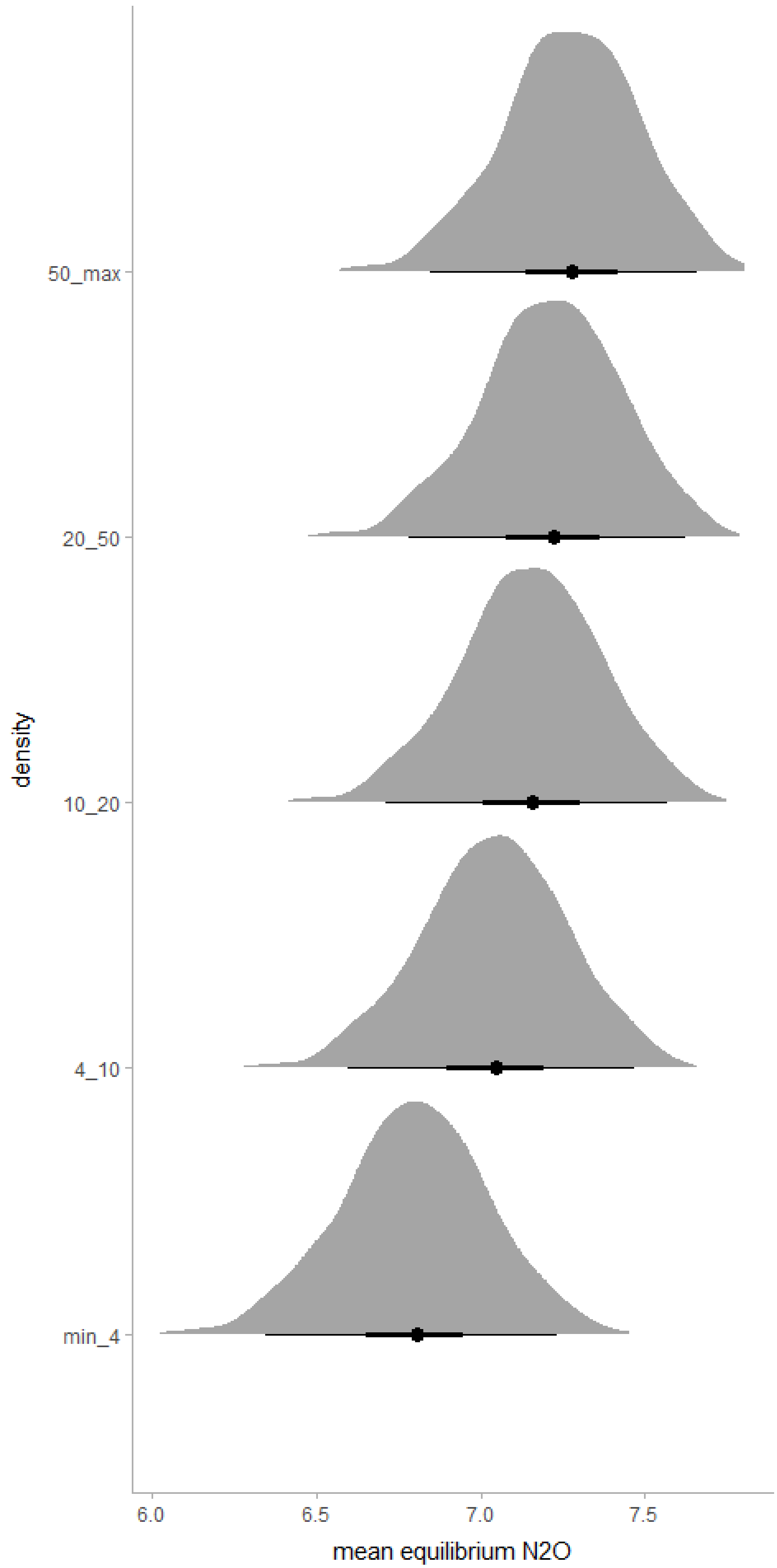

density
50_max
20_50
10_20
4_10
min_4
6.0
6.5
7.0
7.5
mean equilibrium N2O

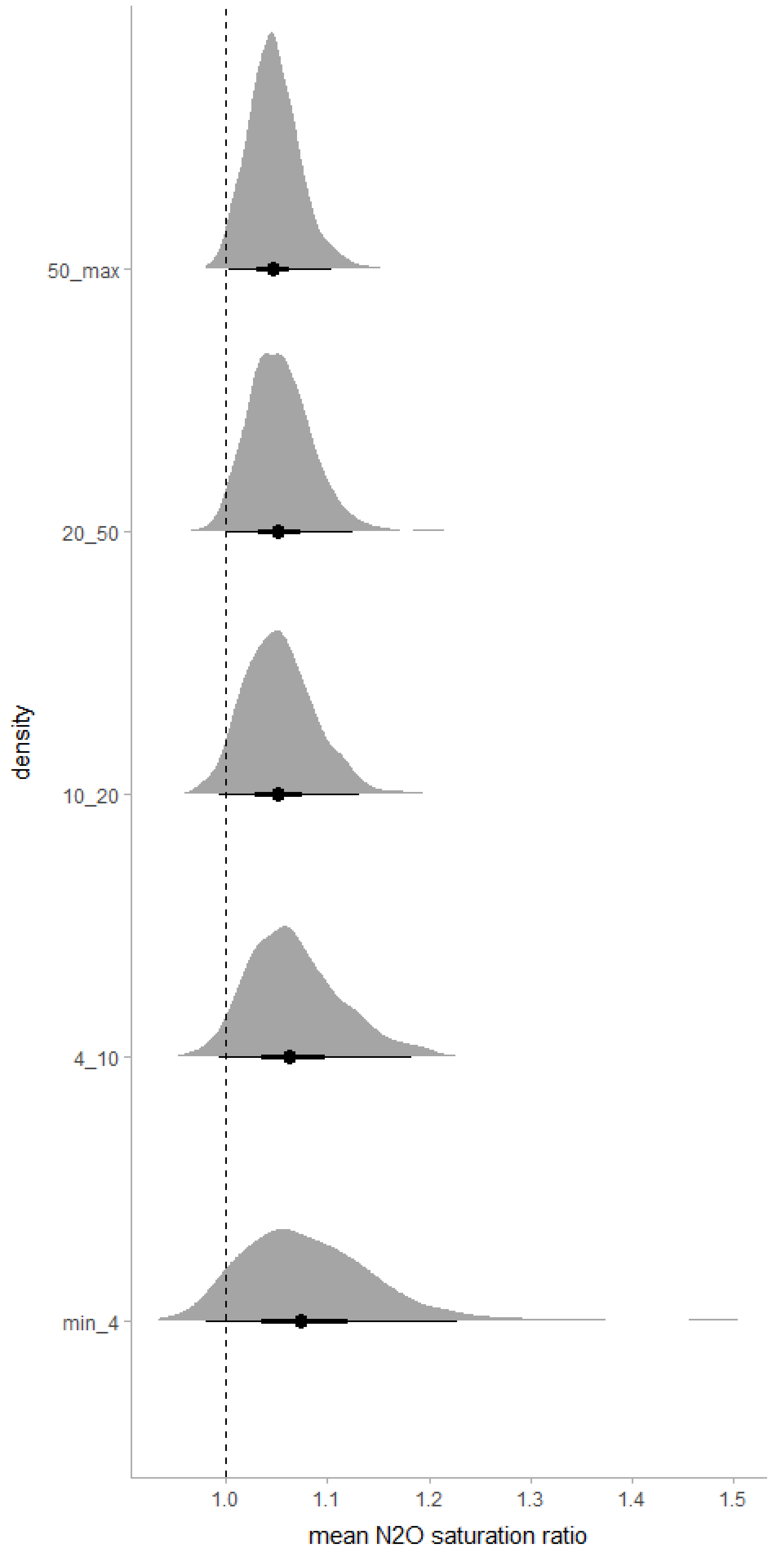
density
50_max
20_50
10_20
4_10
min_4
1.0
1.1
1.2
1.3
1.4
1.5
mean N2O saturation ratio

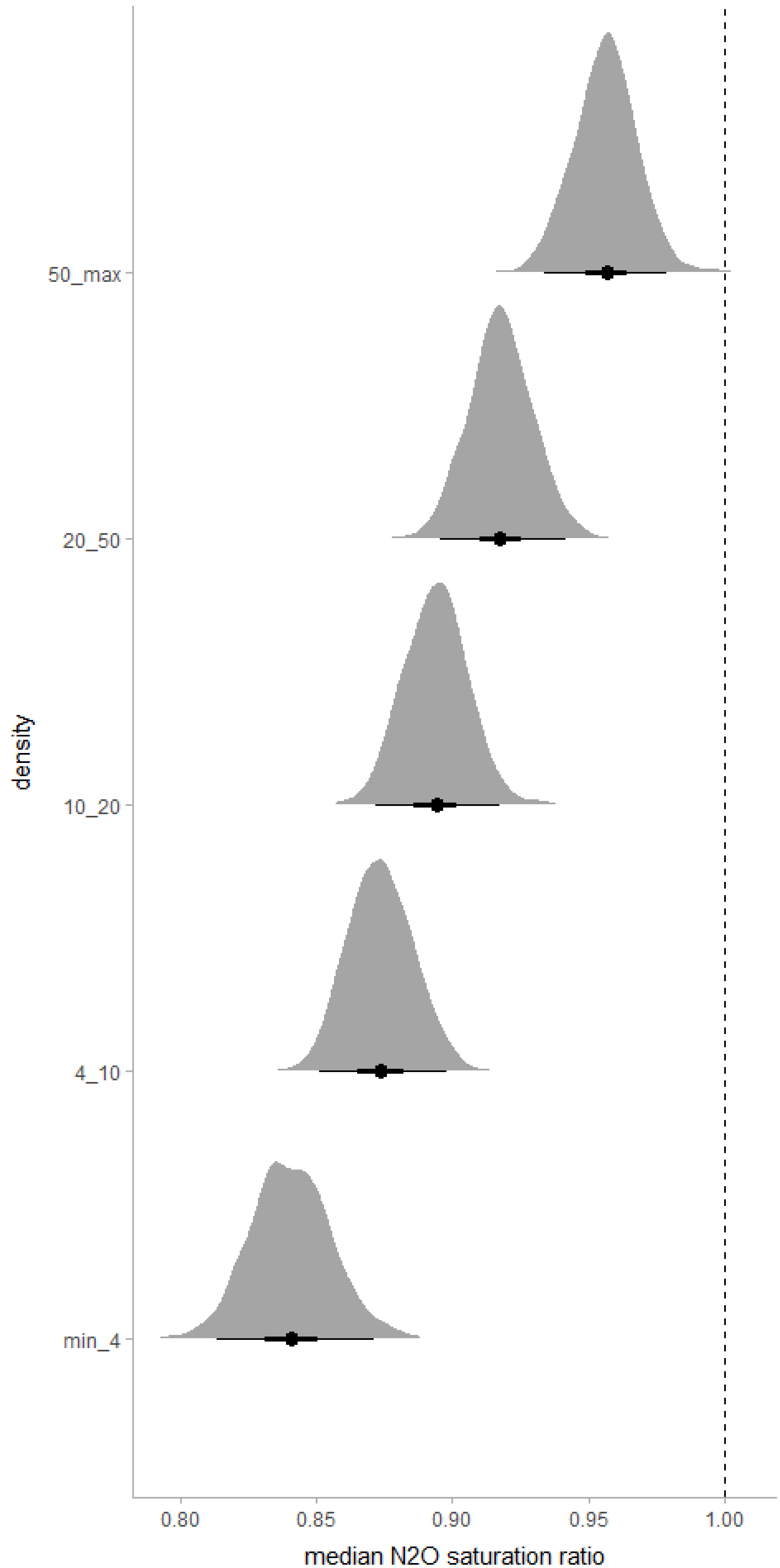

50_max
20_50
10_20
4_10
min_4
density
0.80
0.85
0.90
0.95
1.00
median N2O saturation ratio

Mean *vs.* median below.

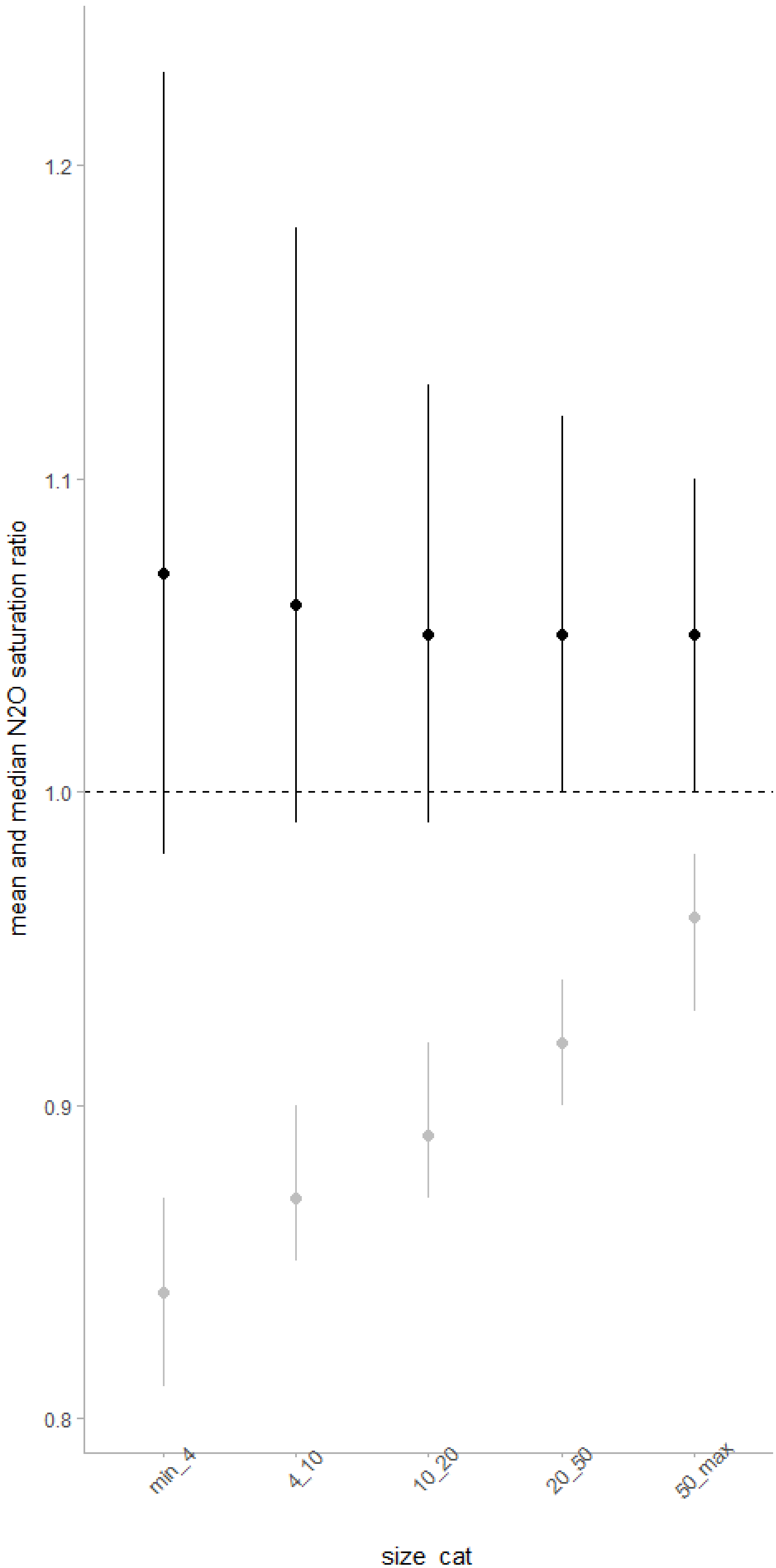

1.2
1.1
1.0
0.9
0.8
mean and median N2O saturation ratio
min_4
4_10
10_20
20_50
50_max
size_cat

And, finally, the estimated proportion of undersaturated lakes in the target population by size category.

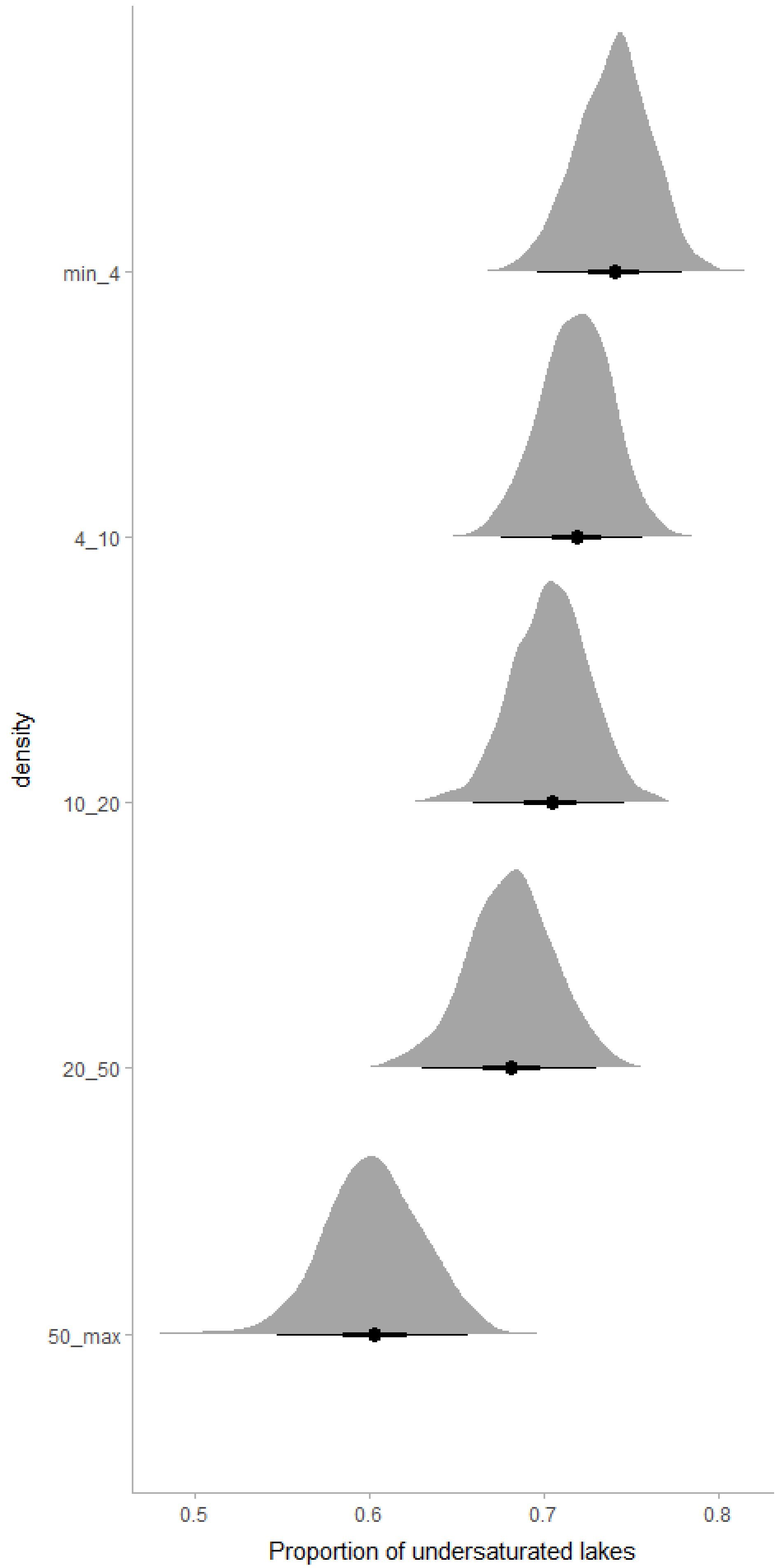

min_4
4_10
10_20
20_50
50_max
density
0.5
0.6
0.7
0.8
Proportion of undersaturated lakes

5.3 Model- *vs.* design-based

Below, estimates from the model-based approach are compared to previously calculated, design-based estimates. In general, the model-based estimates were similar to the design-based estimates. The model-based estimates were typically within the confidence bounds of the design-based estimates, but with much greater precision. Improved precision was expected due to the "shrinkage" induced by the multilevel parameterization, which affords some "borrowing" of information across the various levels of the survey factors.

5.3.1 Dissolved N2O

Below, National mean estimates for dissolved N2O from the model and design-based approaches were compared. The code and results for the design-based approach are located at: https://github.com/USEPA/DissolvedGasNla/tree/master/scripts

The sample-based estimates were also included as a reference and are represented by the black, vertical, dashed lines.

```
### Dissolved N2O estimates

n2o_survey_ests <- read.csv(file = "./../inputData/populationEstimates/Survey_Ests_N2
  tibble() %>%
  rename(estimate = Estimate,
         LCL = LCB95Pct,
         UCL = UCB95Pct) %>%
  select(Type, Subpopulation, estimate, LCL, UCL)

# ests by subpopulations
n2o_survey_ecoregion <- n2o_survey_ests %>%
  filter(Type == "AG_ECO9_NM") %>%
  rename(ecoregion = Subpopulation) %>%
  select(ecoregion, estimate, LCL, UCL) %>%
  mutate(ecoregion = recode(ecoregion,
                            "Coastal Plains" = "CPL",
                            "Northern Appalachians" = "NAP",
                            "Northern Plains" = "NPL",
                            "Southern Appalachians" = "SAP",
                            "Southern Plains" = "SPL",
                            "Temperate Plains" = "TPL",
                            "Upper Midwest" = "UMW",
                            "Western Mountains" = "WMT",
                            "Xeric" = "XER"))

n2o_survey_size <- n2o_survey_ests %>%
```

```
  filter(Type == "AREA_CAT6") %>%
  rename(size_cat = Subpopulation) %>%
  select(size_cat, estimate, LCL, UCL)

### Saturation ratio estimates
sat_survey_ests <- read.csv(file = "./../inputData/populationEstimates/Survey_Ests_N2
  tibble() %>%
  rename(estimate = Estimate,
         LCL = LCB95Pct,
         UCL = UCB95Pct) %>%
  select(Type, Subpopulation, estimate, LCL, UCL)
```

```
all_predictions %>%
  group_by(.draw) %>%
  summarise(mean_n2o = mean(n2o)) %>%
  summarise(estimate = round(median(mean_n2o), 2), # posterior median
    LCL = round(quantile(mean_n2o, probs = 0.025), 2),
    UCL = round(quantile(mean_n2o, probs = 0.975), 2)) %>%
  mutate(type = "model") %>%
  bind_rows(cbind(n2o_survey_ests[1, 3:5], type = rep("survey", 1))) %>%
  add_row(estimate = round(mean(df_model$n2o), 2),
          type = "sample") %>%
  print()
```

```
## # A tibble: 3 × 4
##   estimate   LCL   UCL type
##      <dbl> <dbl> <dbl> <chr>
## 1     7.35  6.59  8.37 model
## 2     8.05  7.02  9.09 survey
## 3     8.72 NA    NA    sample
```

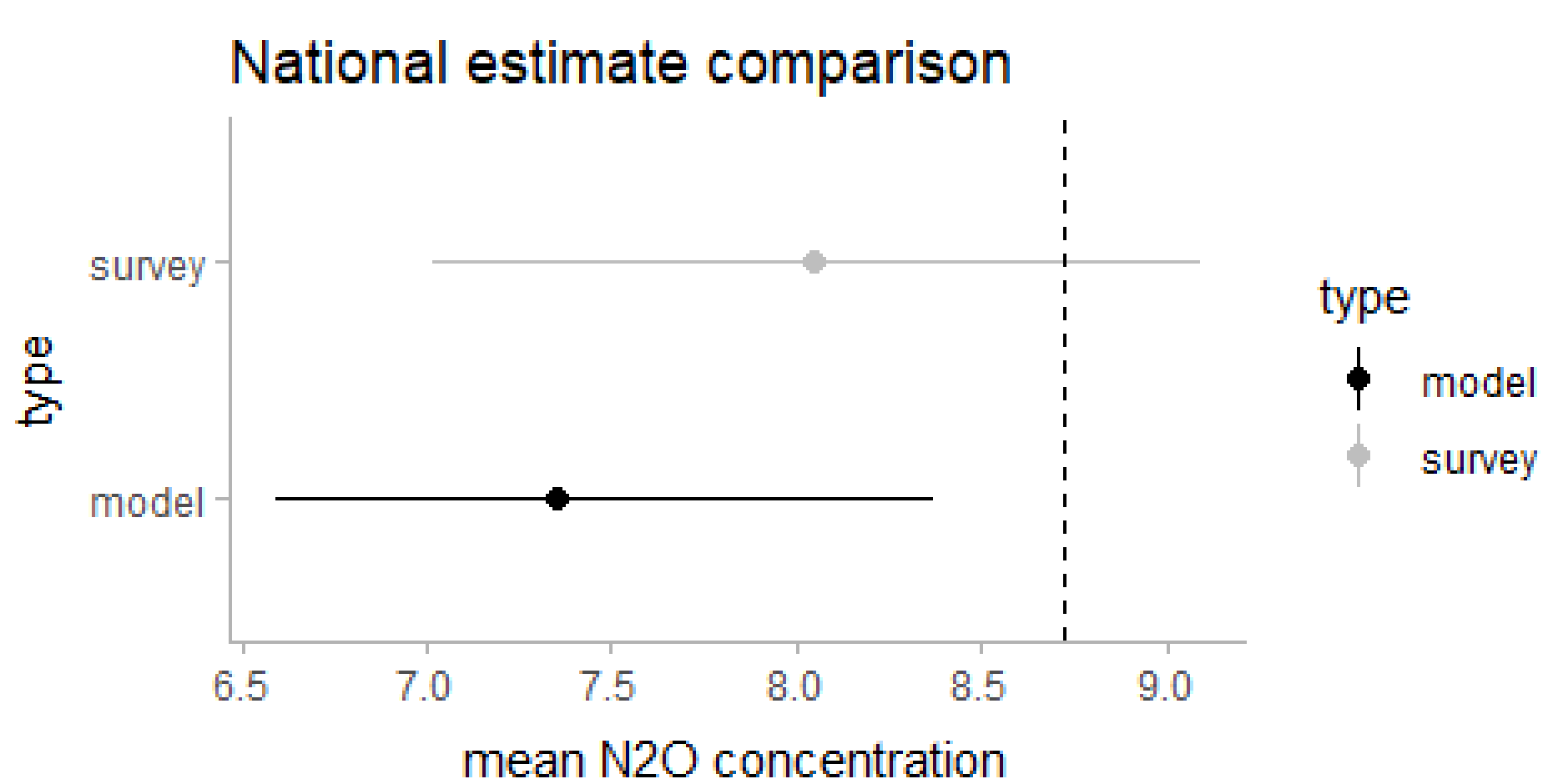

Below, estimates were compared by ecoregion.

```r
all_predictions %>%
  group_by(WSA9, .draw) %>%
  summarise(mean_n2o = mean(n2o)) %>%
  group_by(WSA9, .groups = "drop") %>%
  summarise(estimate = round(median(mean_n2o), 2),
    LCL = round(quantile(mean_n2o, probs = 0.025), 2),
    UCL = round(quantile(mean_n2o, probs = 0.975), 2),
    .groups = "drop") %>%
  mutate(ecoregion = factor(WSA9)) %>%
  mutate(type = "model") %>%
  select(ecoregion, estimate, LCL, UCL, type) %>%
  mutate(ecoregion = forcats::fct_reorder(ecoregion, estimate)) %>%
  bind_rows(cbind(n2o_survey_ecoregion, type = rep("survey", 9))) %>%
  #bind_rows(cbind(n2o_survey_ests[-10,], type = rep("survey", 9))) %>%
  arrange(ecoregion) %>%
  print()
```

```
## # A tibble: 18 × 5
##    ecoregion estimate   LCL   UCL type
##    <chr>        <dbl> <dbl> <dbl> <chr>
##  1 CPL           7.87  6.39 10.1  model
##  2 CPL           8.4   5.13 11.7  survey
##  3 NAP           6.96  6.37  7.78 model
##  4 NAP           7.65  7.21  8.09 survey
##  5 NPL           7.15  6.58  7.9  model
##  6 NPL           6.89  6.39  7.4  survey
##  7 SAP           6.51  5.54  8.14 model
##  8 SAP           7.06  5.93  8.18 survey
##  9 SPL           6.45  5.54  8.23 model
## 10 SPL           6.51  4.89  8.13 survey
## 11 TPL           7.02  6     8.52 model
## 12 TPL           7.77  5.94  9.6  survey
## 13 UMW           7.13  6.53  7.88 model
## 14 UMW          10.8   6.39 15.2  survey
## 15 WMT           8.28  7.49  9.59 model
## 16 WMT           7.75  7.12  8.38 survey
## 17 XER           7.91  6.98  9.38 model
## 18 XER          10.6   7.53 13.6  survey
```

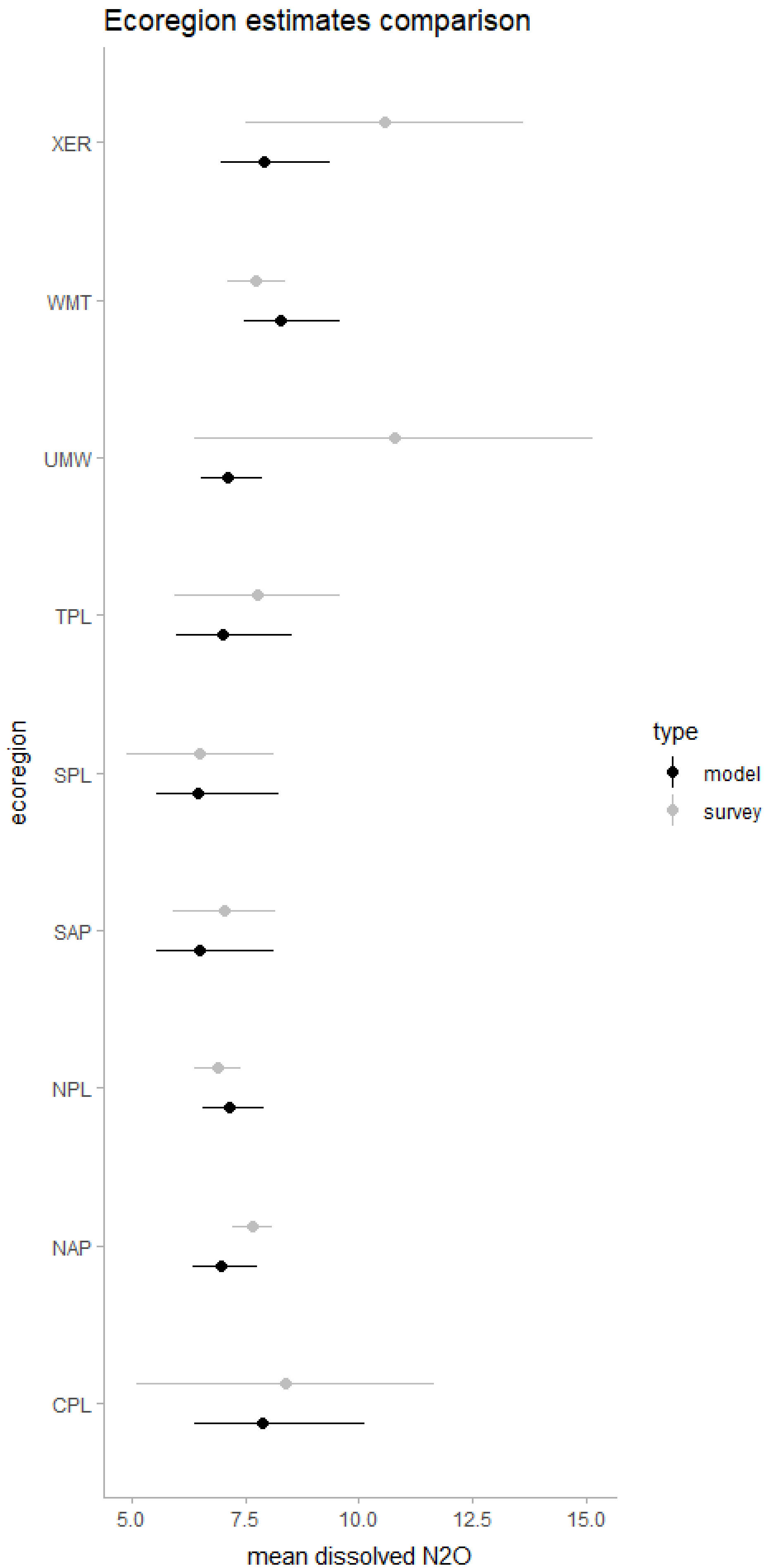
Ecoregion estimates comparison
XER
WMT
UMW
TPL
SPL
SAP
NPL
NAP
CPL
ecoregion
5.0
7.5
10.0
12.5
15.0
mean dissolved N2O
type
model
survey

Means were compared according to size categories below.

```
## # A tibble: 10 × 5
##    size   estimate   LCL   UCL type
##    <fct>     <dbl> <dbl> <dbl> <chr>
##  1 10_20      7.5   6.9   8.2  model
##  2 10_20      7.6   7.13  8.08 survey
##  3 4_10       7.5   6.8   8.4  model
##  4 4_10       7.64  6.51  8.78 survey
##  5 50_max     7.5   7     8.1  model
##  6 50_max     7.95  7.44  8.47 survey
##  7 min_4      7.3   6.5   8.4  model
##  8 min_4      8.17  6.41  9.92 survey
##  9 20_50      7.5   7     8.2  model
## 10 20_50      8.57  7.67  9.47 survey
```

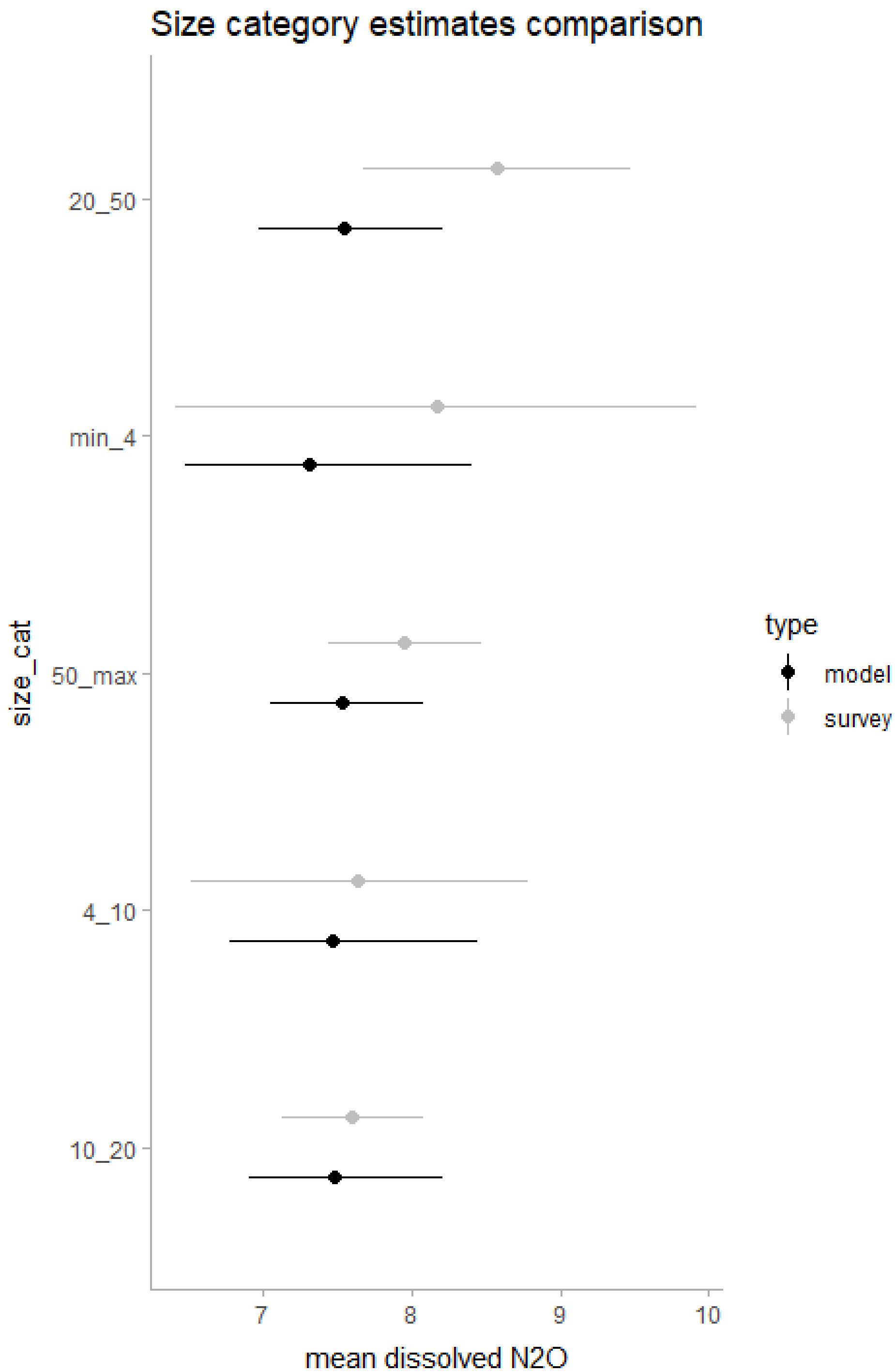

5.3.2 Saturation

Below, the same comparisons were made for the saturation estimates.

```
all_predictions %>%
  group_by(.draw) %>%
  summarise(mean_sat = mean(n2osat), .groups = "drop") %>%
  summarise(estimate = round(median(mean_sat), 3),
    LCL = round(quantile(mean_sat, probs = 0.025), 3),
    UCL = round(quantile(mean_sat, probs = 0.975), 3),
```

```r
    .groups = "drop") %>%
  mutate(type = "model") %>%
  bind_rows(cbind(sat_survey_ests[10, 2:4], type = rep("survey", 1))) %>%
  add_row(estimate = round(mean(df_model$n2o / df_model$n2o_eq), 3),
          type = "sample") %>%
  print()
```

```
## # A tibble: 3 × 4
##   estimate    LCL   UCL type
##      <dbl>  <dbl> <dbl> <chr>
## 1     1.07  0.988  1.20 model
## 2     1.10  0.952  1.24 survey
## 3     1.17 NA     NA    sample
```

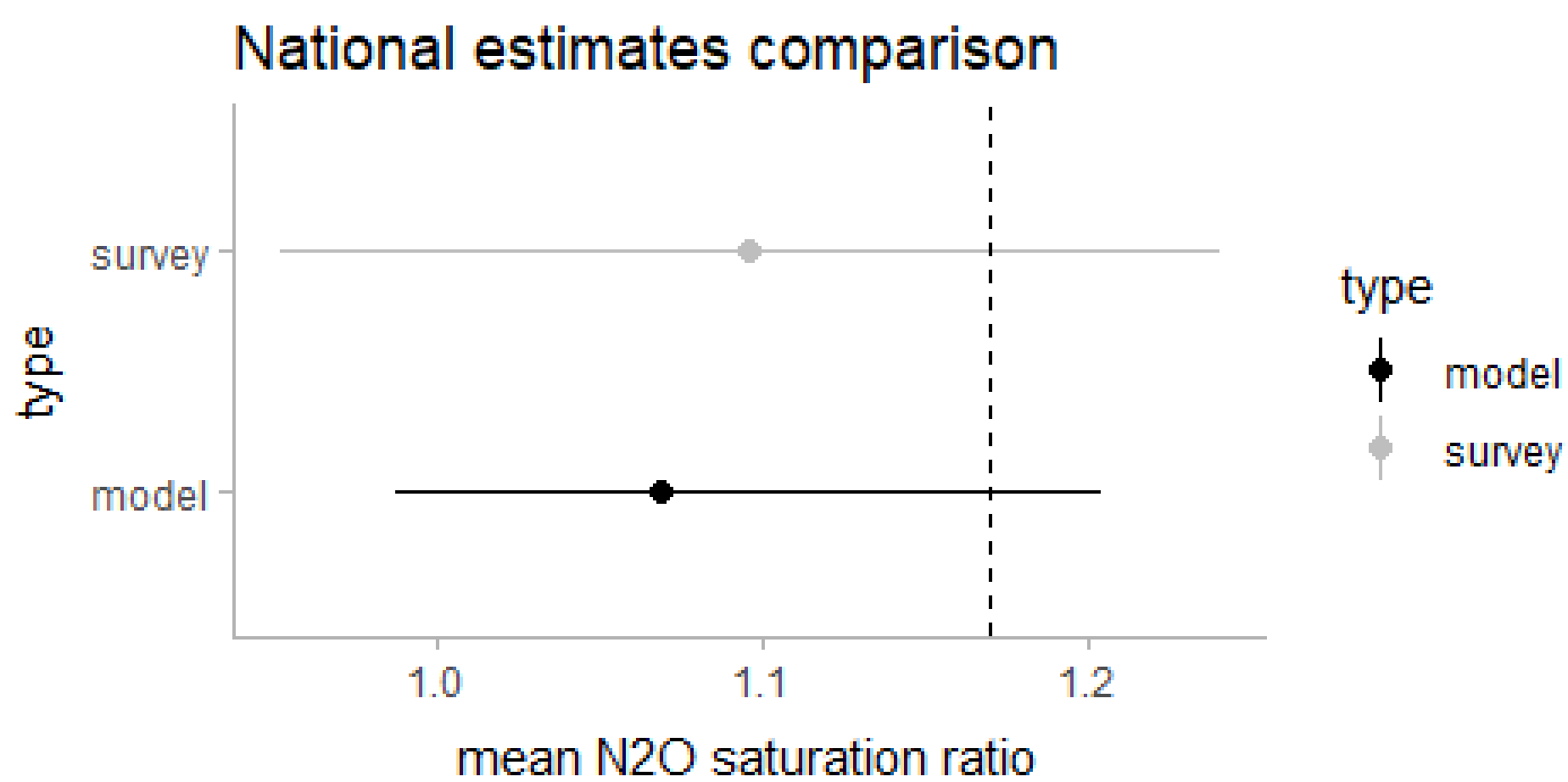

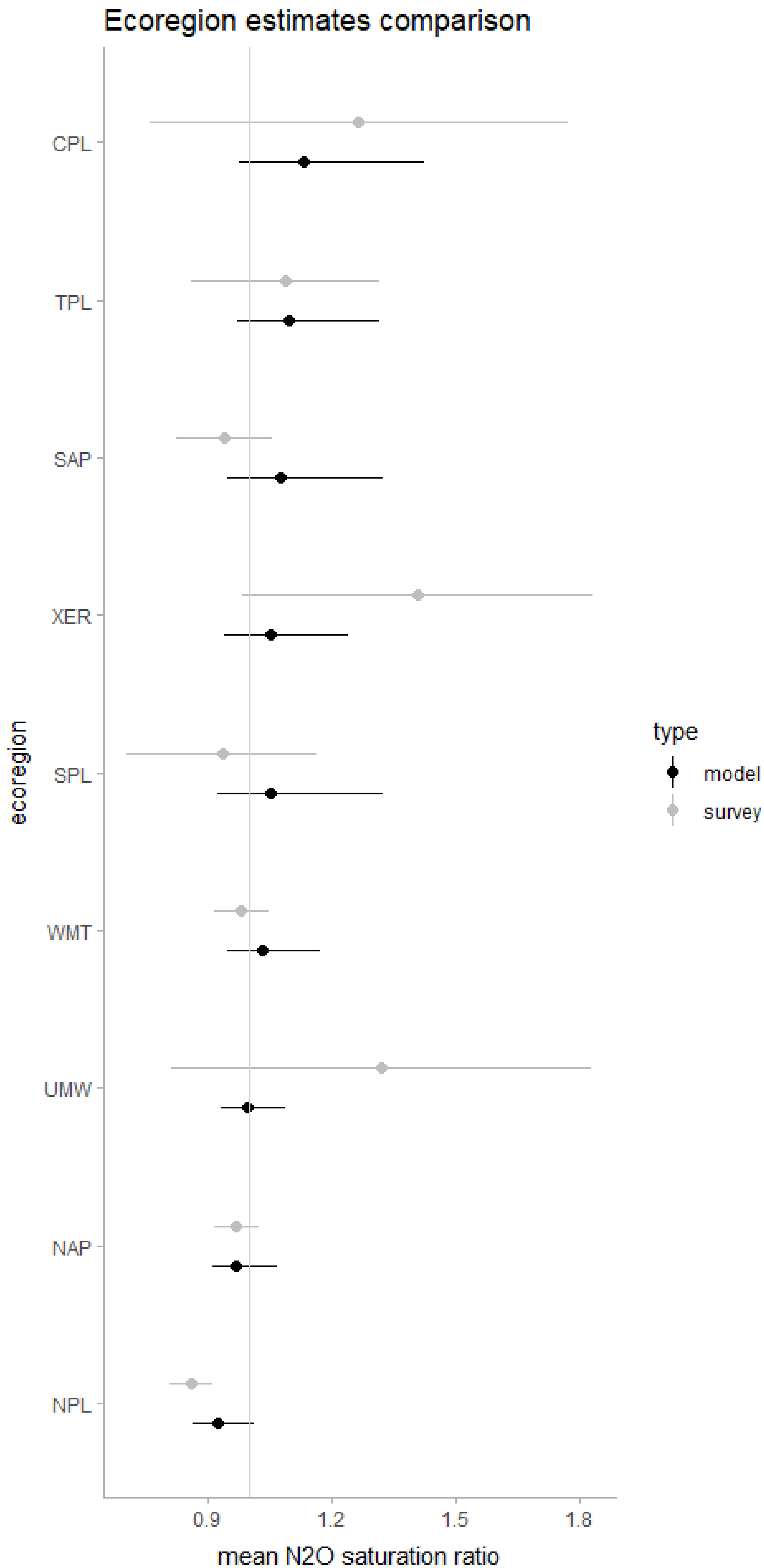
Ecoregion estimates comparison
CPL
TPL
SAP
XER
SPL
WMT
UMW
NAP
NPL
ecoregion
0.9
1.2
1.5
1.8
mean N2O saturation ratio
type
model
survey

```
## # A tibble: 10 × 5
##    size   estimate   LCL   UCL type
##    <ord>     <dbl> <dbl> <dbl> <chr>
##  1 min_4      1.07 0.98   1.23 model
##  2 min_4      1.12 0.874  1.37 survey
##  3 4_10       1.06 0.993  1.18 model
##  4 4_10       1.02 0.889  1.15 survey
##  5 10_20      1.05 0.993  1.13 model
##  6 10_20      1.02 0.956  1.08 survey
##  7 20_50      1.05 1.00   1.12 model
##  8 20_50      1.14 1.02   1.27 survey
##  9 50_max     1.05 1.00   1.10 model
## 10 50_max     1.06 0.987  1.12 survey
```

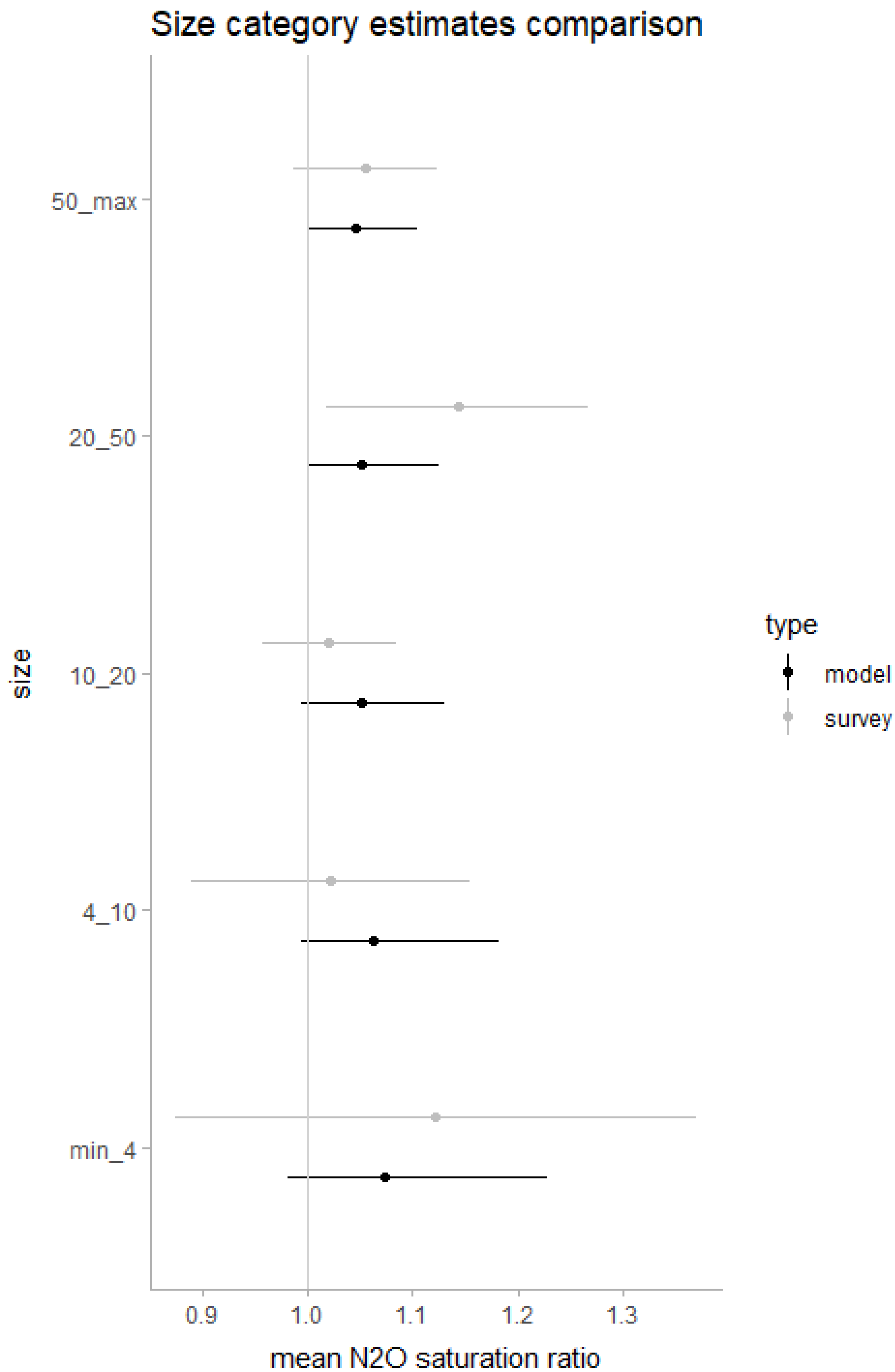

6 Session Info

```
sessionInfo()
```

```
## R version 4.4.0 (2024-04-24 ucrt)
## Platform: x86_64-w64-mingw32/x64
## Running under: Windows 11 x64 (build 22631)
```

```
##
## Matrix products: default
##
##
## locale:
## [1] LC_COLLATE=English_United States.utf8
## [2] LC_CTYPE=English_United States.utf8
## [3] LC_MONETARY=English_United States.utf8
## [4] LC_NUMERIC=C
## [5] LC_TIME=English_United States.utf8
##
## time zone: America/New_York
## tzcode source: internal
##
## attached base packages:
## [1] stats     graphics  grDevices utils     datasets  methods   base
##
## other attached packages:
##  [1] archive_1.1.11   arrow_19.0.1     tictoc_1.2.1     brms_2.22.0
##  [5] Rcpp_1.0.14      tidybayes_3.0.7  bayesplot_1.11.1 itertools_0.1-3
##  [9] iterators_1.0.14 foreach_1.5.2    future_1.34.0    lubridate_1.9.4
## [13] forcats_1.0.0    stringr_1.5.1    purrr_1.0.4      readr_2.1.5
## [17] tidyr_1.3.1      tibble_3.2.1     tidyverse_2.0.0  dplyr_1.1.4
## [21] ggrepel_0.9.6    kableExtra_1.4.0 gridExtra_2.3    ggExtra_0.10.1
## [25] moments_0.14.1   ggpubr_0.6.0     ggplot2_3.5.1
##
## loaded via a namespace (and not attached):
##  [1] inline_0.3.21        rlang_1.1.5          magrittr_2.0.3
##  [4] matrixStats_1.5.0    compiler_4.4.0       mgcv_1.9-1
##  [7] loo_2.8.0            reshape2_1.4.4       systemfonts_1.2.1
## [10] vctrs_0.6.5          crayon_1.5.3         pkgconfig_2.0.3
## [13] arrayhelpers_1.1-0   fastmap_1.2.0        backports_1.5.0
## [16] labeling_0.4.3       utf8_1.2.4           promises_1.3.2
## [19] rmarkdown_2.29       tzdb_0.4.0           ragg_1.3.3
## [22] bit_4.5.0.1          xfun_0.51            later_1.4.1
## [25] broom_1.0.7          parallel_4.4.0       R6_2.6.1
## [28] RColorBrewer_1.1-3   StanHeaders_2.32.10  stringi_1.8.4
## [31] parallelly_1.42.0    car_3.1-3            rstan_2.32.6
## [34] assertthat_0.2.1     knitr_1.49           splines_4.4.0
## [37] httpuv_1.6.15        Matrix_1.7-0         timechange_0.3.0
## [40] tidyselect_1.2.1     rstudioapi_0.17.1    abind_1.4-8
## [43] yaml_2.3.10          codetools_0.2-20     miniUI_0.1.1.1
## [46] pkgbuild_1.4.6       listenv_0.9.1        plyr_1.8.9
## [49] lattice_0.22-6       shiny_1.10.0         withr_3.0.2
## [52] bridgesampling_1.1-2 posterior_1.6.1      coda_0.19-4.1
## [55] evaluate_1.0.3       RcppParallel_5.1.10  ggdist_3.3.2
## [58] xml2_1.3.7           pillar_1.10.1        carData_3.0-5
## [61] tensorA_0.36.2.1     stats4_4.4.0         checkmate_2.3.2
## [64] distributional_0.5.0 generics_0.1.3       hms_1.1.3
```

```
## [67] rstantools_2.4.0     munsell_0.5.1       scales_1.3.0
## [70] globals_0.16.3       xtable_1.8-4        glue_1.8.0
## [73] tools_4.4.0          diffobj_0.3.5       ggsignif_0.6.4
## [76] mvtnorm_1.3-3        cowplot_1.1.3       grid_4.4.0
## [79] QuickJSR_1.6.0       colorspace_2.1-1    nlme_3.1-164
## [82] Formula_1.2-5        cli_3.6.4           textshaping_1.0.0
## [85] svUnit_1.0.6         viridisLite_0.4.2   svglite_2.1.3
## [88] Brobdingnag_1.2-9    gtable_0.3.6        rstatix_0.7.2
## [91] digest_0.6.37        farver_2.1.2        htmltools_0.5.8.1
## [94] lifecycle_1.0.4      mime_0.12           bit64_4.6.0-1
```

7 References

Bafumi, Joseph, and Andrew Gelman. 2007. "Fitting Multilevel Models When Predictors and Group Effects Correlate." Journal Article. *Social Science Research Network*, 1–14. https://ssrn.com/abstract=1010095.

Bürkner, Paul-Christian. 2017. "Brms: An r Package for Bayesian Multilevel Models Using Stan." Journal Article. *2017* 80 (1): 28. https://doi.org/10.18637/jss.v080.i01.

Gelman, Andrew, John B. Carlin, Hal S. Stern, David B. Dunson, Aki Vehtari, and Donald B. Rubin. 2014. *Bayesian Data Analysis*. Book. 4th ed. New York: CRC Press.

Gelman, Andrew, Jennifer Hill, and Aki. Vehtari. 2020. *Regression and Other Stories: Analytical Methods for Social Research*. Book. 1st ed. Cambridge: CRC Press.

Gelman, Andrew, and Thomas Little. 1997. "Postratification into Many Categories Using Hierarchical Logistic Regression." Journal Article. *Survey Methodology* 23 (2): 127–35.

Kennedy, Lauren, and Andrew Gelman. 2015. "Polarizing the Electoral Connection: Partisan Representation in Supreme Court Confirmation Politics." Journal Article. *The Journal of Politics* 77 (3): 787–804. https://psycnet.apa.org/doi/10.1037/met0000362.

———. 2021. "Know Your Population and Know Your Model: Using Model-Based Regression and Poststratification to Generalize Findings Beyond the Observed Sample." Journal Article. *Psychological Methods* 26 (5): 547–58. https://psycnet.apa.org/doi/10.1037/met0000362.

McElreath, Richard. 2020. *Statistical Rethinking: A Bayesian Course with Examples in r and Stan*. Book. 2nd ed. Boca Raton: CRC Press.

Merkle, Edgar C., Ellen Fitzsimmons, James Uanhoro, and Ben Goodrich. 2021. "Efficient Bayesian Structural Equation Modeling in Stan." Journal Article. *Journal of Statistical Software* 100 (6): 1–22. https://doi.org/https://doi.org/10.18637/jss.v100.i06.

Merkle, Edgar C., and Yves Rosseel. 2018. "Blavaan: Bayesian Structural Equation Models via Parameter Expansion." Journal Article. *Journal of Statistical Software* 85 (4): 1–30. https://doi.org/https://doi.org/10.18637/jss.v085.i04.

Park, David K., Andrew Gelman, and Joseph Bafumi. 2004. "Bayesian Multilevel Estimation with Poststratification: State-Level Estimates from National Polls." Journal Article. *Political Analysis* 12 (4): 375–85. https://doi.org/10.1093/pan/mph024.

Poggiato, Giovanni, Tamara Munkemuller, Daria Bystrova, Julyan Arbel, James S. Clark, and Wilfried Thuiller. 2021. "On the Interpretations of Joint Modeling in Community Ecology." Journal Article. *Trends in Ecology & Evolution* 36 (5): 391–401. https://doi.org/https://doi.org/10.1016/j.tree.2021.01.002.

R Core Team. 2021. *R: A Language and Environment for Statistical Computing*. Vienna, Austria: R Foundation for Statistical Computing. https://www.R-project.org/.

Stan Development Team. 2018a. "RStan: The r Interface to Stan." Journal Article. http://mc-stan.org.

———. 2018b. "Stan Modeling Language Users Guide and Reference Manual, Version 2.18.0." Journal Article. http://mc-stan.org.

———. 2018c. "The Stan Core Library, Version 2.18.0." Journal Article. http://mc-stan.org.

U.S. EPA. 2015. "National Lakes Assessment 2017 Survey Design." Report. https://www.epa.gov/sites/default/files/2020-08/documents/nla2017_design_documentation_20150928.pdf.

Warton, David I., Guillame F. Blanchet, Robert B. O'Hara, Otso Ovaskainen, Sara Taskinen, Steven C. Walker, and Francis K. C. Hui. 2015. "So Many Variables: Joint Modeling in Community Ecology." Journal Article. *Trends in Ecology & Evolution* 30 (12): 766–79. https://doi.org/https://doi.org/10.1016/j.tree.2015.09.007.

Webb, Jackie R., Nicole M. Hayes, Gavin L. Simpson, Peter R. Leavitt, Helen M. Baulch, and Kerri Finlay. 2019. "Widespread Nitrous Oxide Undersaturation in Farm Waterbodies Creates an Unexpected Greenhouse Gas Sink." Journal Article. *Proceedings of the National Academy of Sciences* 116 (20): 9814–19. https://doi.org/10.1073/pnas.1820389116.

Zachmann, Luke J., Erin M. Borgman, Dana L. Witwicki, Megan C. Swan, Cheryl McIntyre, and N. Thompson Hobbs. 2022. “Bayesian Models for Analysis of Inventory and Monitoring Data with Non-Ignorable Missingness.” Journal Article. *Journal of Agricultural, Biological and Environmental Statistics* 27: 125–48. https://doi.org/https://doi.org/10.1007/s13253-021-00473-z.

Supplemental Information 2: Sensitivity to measurement error in dissolved gas measurements

Roy Martin, Jake Beaulieu, Michael McManus 2025-03-06

- 1 Background
- 2 Dissolved concentration
  - 2.1 Calculate N_2O
  - 2.2 Empricial variation
  - 2.3 Simulate measurment error
    - 2.3.1 Equilibrated headspace
    - 2.3.2 Ambient air
    - 2.3.3 Water temperature
    - 2.3.4 Barometric pressure
    - 2.3.5 Water volume
    - 2.3.6 Mixing ratio
      - 2.3.6.1 Ambient air
        - 2.3.6.1.1 $R_{M(low)}$
        - 2.3.6.1.2 $R_{M(equal)}$
        - 2.3.6.1.3 $R_{M(high)}$
      - 2.3.6.2 Pure gas
        - 2.3.6.2.1 $R_{M(low)}$
        - 2.3.6.2.2 $R_{M(equal)}$
        - 2.3.6.2.3 $R_{M(high)}$
    - 2.3.7 All observables
  - 2.4 Summary of contributions to error
- 3 Equilibrium concentration
  - 3.1 Simulate equilibrium N_2O
  - 3.2 Simulate measurement error
    - 3.2.1 Ambient air
    - 3.2.2 Water temperature
    - 3.2.3 Barometric pressure
    - 3.2.4 All observables
  - 3.3 Summary of contributions to error

- 4 Saturation ratio
  - 4.1 Standard GC
    - 4.1.1 Ambient air, standard thermometer
      - 4.1.1.1 Map source-sink status
    - 4.1.2 Pure gas, standard thermometer
      - 4.1.2.1 Source-sink status
    - 4.1.3 Ambient air, high precision thermometer
      - 4.1.3.1 Source-sink status
    - 4.1.4 Pure gas, high precision thermometer
      - 4.1.4.1 Source-sink status
  - 4.2 High precision GC
    - 4.2.1 Pure gas, standard thermometer
      - 4.2.1.1 Source-sink status
    - 4.2.2 Pure gas, high precision thermometer
      - 4.2.2.1 Source-sink status
- 5 MIMS
  - 5.1 Dissolved concentration
    - 5.1.1 Standard GC
    - 5.1.2 High precision GC
  - 5.2 Saturation ratio
    - 5.2.1 Standard GC
      - 5.2.1.1 Standard field temperature
        - 5.2.1.1.1 Source-sink status
      - 5.2.1.2 High precision temperature
        - 5.2.1.2.1 Source-sink status
    - 5.2.2 High precision GC
      - 5.2.2.1 Standard temperature
        - 5.2.2.1.1 Source-sink status
      - 5.2.2.2 High precision temperature
        - 5.2.2.2.1 Source-sink status
- 6 Summary of ratio results
- 7 Update data with uncertainty info
- 8 Session Info


```r
library(knitr)
library(rmarkdown)
library(bayesplot)
library(kableExtra)
```

```
library(ggpubr)
library(tidyverse)
library(tidybayes)
library(ggplot2)
library(ggExtra)
library(gridExtra)
library(readxl)
library(janitor)
library(sf)
library(usmap)

# Identify local path for each user
localPath <- Sys.getenv("USERPROFILE")

# Define helper functions
# standardized formatting for column names
toEPA <- function(X1){
  names(X1) = tolower(names(X1))
  names(X1) = gsub(pattern = c("\\(| |#|)|/|-|\\+|:|_"), replacement = ".", x = na
  X1
}

options(max.print = 2000)
```

1 Background

In this document we explore a range of scenarios wherein measurement errors could arise in methods for determining the concentration of N_2O from field and laboratory measurements observed as part of the 2017 National Lakes Assessment (NLA).

2 Dissolved concentration

The equation for estimating the concentration (nM) of N_2O gas dissolved in water is:

$$C = B^{-6} \cdot \left(\frac{V_g(M_h - M_r)}{(8.3144598 \cdot K \cdot V_w) + (H^{\theta} \cdot e^{2700 \cdot (\frac{1}{K} - \frac{1}{298.15})} \cdot M_h)} \right)$$

where:

- C = concentration (nM) dissolved in water

- B = barometric pressure (kPa)
- V_g = volume of reference gas in headspace (mL)
- M_h = gas measured in the equilibrated headspace
- M_r = gas measured in the reference
- V_w = volume of water below the headspace (mL)
- K = temperature (K) (T_c + 273 below)
- H^θ = Henry's law constant (H_{N_2O} or H_{Ar} below)

and:

$$V = V_g + V_w$$
$$V_g = V \cdot R_M$$
$$V_w = V \cdot (1 - R_M)$$

where:

- V = Total volume of the mixing vessel (e.g. 140mL syringe)
- R_M = Gas mixing proportion (gas volume / mixing vessel volume)
- V_g = Volume of gas (mL) in the mixing vessel
- V_w = Volume of water (mL) in the mixing vessel

2.1 Calculate N_2O

Below is a summary of the empirical measurements of N2O (ppm) in the equilibrated headspace.

```
load("./../inputData/dg.RData")

# summary of measured N2O ppm in equilibrated headspace gas
dg %>%
  group_by(sample.source) %>%
  summarize(mean_n2O = mean(n2o.ppm.gc, na.rm = TRUE),
            median_n2o = median(n2o.ppm.gc, na.rm = TRUE)) #mean = 0.36, median = 0.
```

```
## # A tibble: 2 × 3
##   sample.source mean_n2O median_n2o
##   <chr>            <dbl>      <dbl>
```

```
## 1 AIR              0.309      0.310
## 2 DG               0.362      0.307
```

Calculate a single concentration without measurement error to represent a “true” value for dissolved N_2O. The median of the empirical measurements was used for values of M_h and M_r (M_a_n2o).

```
# Set volume of syringe/vessel
V <- 140 # 140mL syringe

# Set ratio of gas volume to water volume in the mixing vessel
R_M <- 0.25

B <- 99 # barometric pressure (kPa)
V_g <- V * R_M # volume of reference gas in headspace
V_w <- V * (1 - R_M) # volume of water below headspace
T_c <- 23 # temperature ( C )
M_h <- 0.307 # gas measured in headspace; Changes as function of R_M at constant C
M_a_n2o <- 0.310 # gas measured in reference (air)
H_n2o <- 0.00024 #mol m-3 Pa, range: 0.00018 - 0.00025; Henry's law constant
H_ar <- 0.000014 #mol m-3 Pa, range: 0.000013 - 0.000016; Henry's law constant

C <- 1e-6 * B * (V_g * (M_h - M_a_n2o) / (8.3144598 * (T_c + 273.15) * V_w) + H_n2

C <- C * 1e9 #convert mol to nmol

round(C, 1)
```

```
## [1] 7.7
```

```
#### Re-arrange for Mh
M_h <- (((C / 1e9) * 8.3144598 * (T_c + 273.15) * V_w) + (1e-6 * B * V_g * M_a_n2o

M_h
```

```
## [1] 0.307
```

2.2 Empricial variation

Measurement error for this analysis is assumed to be the standard deviation of repeated analyses of the analytical standards throughout the period of time that the NLA2017 samples were analyzed, which was σ = 0.0078725.

2.3 Simulate measurment error

Below, potential errors in GC measurements for are simulated and assessed s eparately for measurements of N_2O in:

- 1. the equilibrated headspace,
- 2. the air, and
- 3. the headspace + air

Error is then simulated and assessed separately for measurements of:

- 4. water temperature,
- 5. barometric pressure, and
- 6. water volume

Finally, all sources of error (1, 2, 4, 5, and 6) were simulated simultaneously and assessed as an estimate of the total variation in N_2O measurements. The estimated absolute error was added to each of the empirical point measurements recorded in this study, which resulted in an estimate of uncertainty due to measurement error for each empirical observation. The errors were also carried through for estimates of the saturation ratio and a categorical "source" or "sink" status was assigned for each site. This status was assigned such that any observations where the central 95th percentile of the measurement plus simulated error included 1.0 were deemed to be of "undeterminded" status. The source/sink/undetermined status for each site was then mapped.

Set the number of simulations for estimating error:

```
nsim <- 1e5
```

2.3.1 Equilibrated headspace

Simulate measurement error in dissolved N_2O due solely to variation in GC measurements of gas in the equlibrated headspace (M_h above).

```
# Create empty vector to fill below with simulated values for N2O observed with
# error due to variation in the GC measurments of gas in the equilibrated
# headspace. The subscript, "me", denotes an observed or measured value, which
# is contrasted with the known value, C, set above.
C_h_me <- rep(NA, nsim)

# The true value for the concentration of equilibrated gas in the headspace,
# M_h, was set above. This step simulates additive error in this measurement
# that is centered over the true value. The observed concentration of
# equilibrated gas, M_h_me, is simulated as normally distributed variable, with a
# M_h, and a standard deviation specified as the observed sd of replicate GC
# measurements in our lab.
M_h_me <- rnorm(nsim, M_h, n2o_sd_epa)

for(i in 1:nsim){
  C_h_me[i] <- 1e-6 * B * (V_g * (M_h_me[i] - M_a_n2o) / (8.3144598 * (T_c + 273.1
}

# combine into dataframe for ggplot.
C_h_df <- tibble(C_h_me = C_h_me, M_h_me = M_h_me)
# save absolute error for plot
C_h_me_abs <- (C_h_me * 1e9) - C
```

The results of the simulations are summarized using density histograms below.

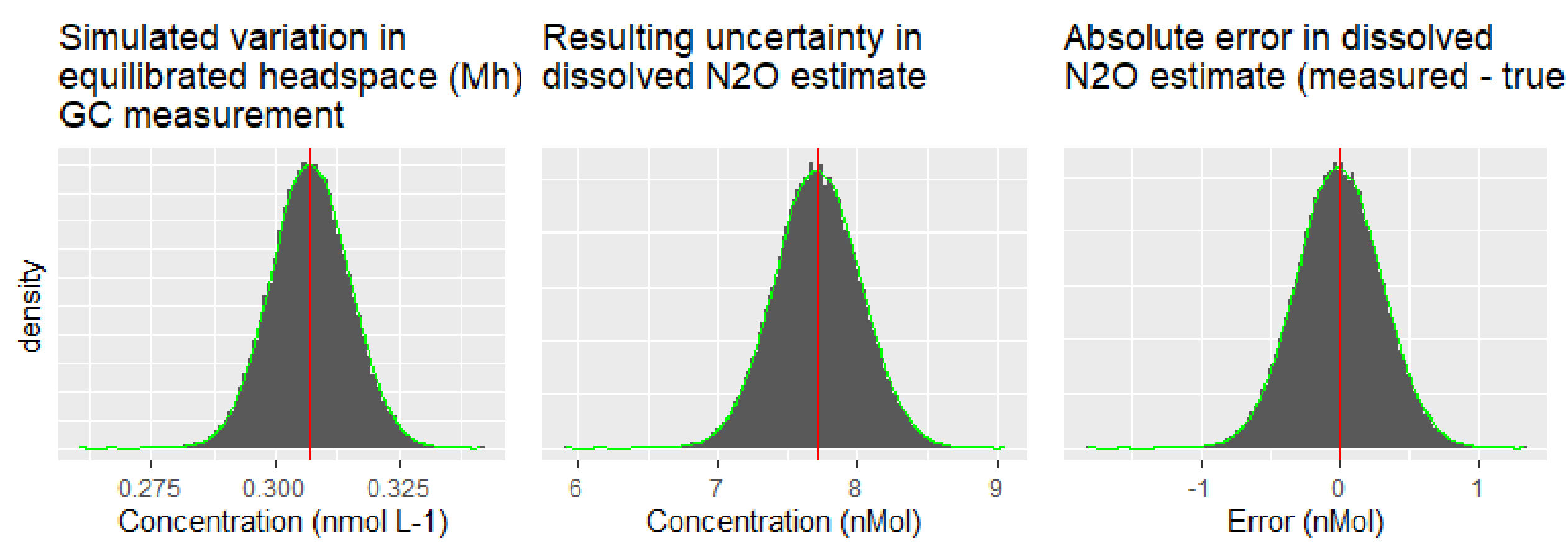

2.3.2 Ambient air

Error in the GC measurements of N_2O in the ambient air (M_r above) reference is simulated below. The result of the simulation is plotted as a histogram to illustrate the variation in the measurement. The resulting effect on the uncertainty in the simulated dissolved N2O observation is plotted along with the absolute error in the simulated measurement.

```
C_a_me <- rep(NA, nsim)

M_a_n2o_me <- rnorm(nsim, M_a_n2o, n2o_sd_epa) # m.e. measured gas in air

for(i in 1:nsim){
  C_a_me[i] <- 1e-6 * B * (V_g * (M_h - M_a_n2o_me[i]) / (8.3144598 * (T_c + 273.1
}

# combine into dataframe for ggplot. qplot, which accepts vectors,
# was deprecated in ggplot 3.4.0
C_a_df <- tibble(C_a_me = C_a_me, M_a_n2o_me = M_a_n2o_me)
# save absolute error for plot
C_a_me_abs<- (C_a_me * 1e9) - C
```

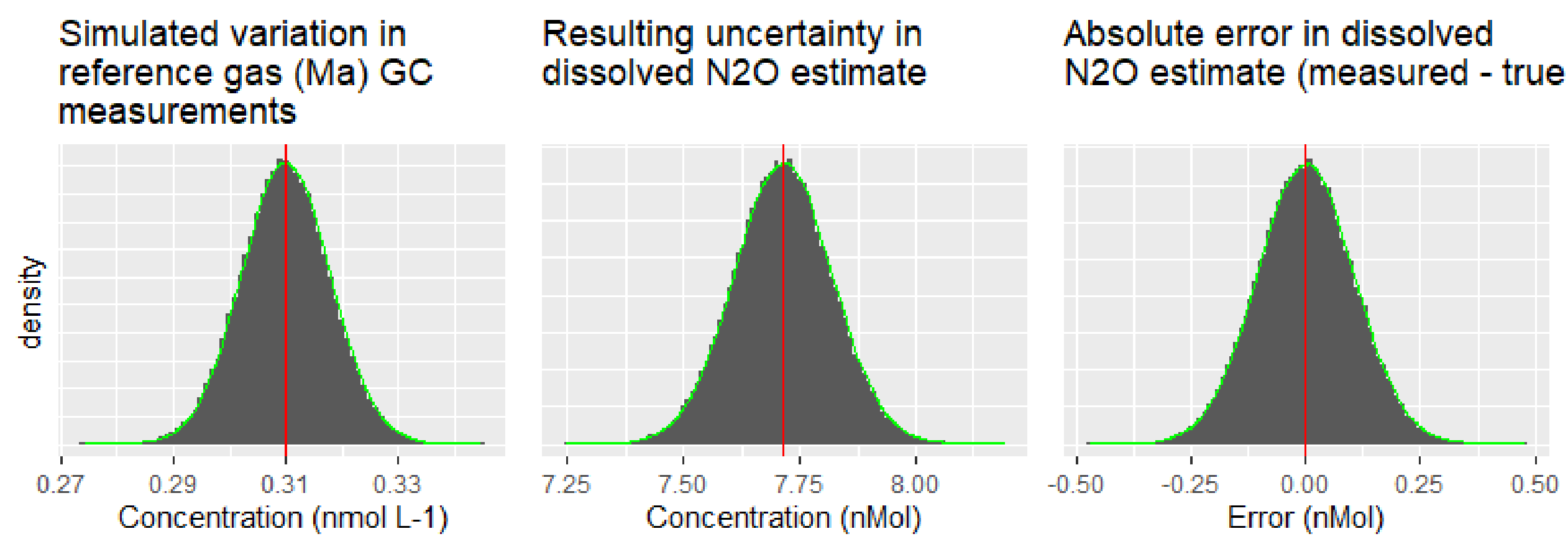

2.3.3 Water temperature

Next, error in water temperature measurements is simulated and its potential effect on the final dissolved N2O concentration assessed.

```
C_tc_me <- rep(NA, nsim)

# How to get standard deviation of temperature measurement?
# calibration certificate reports an uncertainty of +/-0.058C
# take standard deviation of simulated measurements with specified uncertainty
T_c_sd <- sd(runif(n = nsim, min = T_c - 0.058, max = T_c + 0.058)) # calibration
T_c_me <- rnorm(nsim, T_c, T_c_sd) #m.e. in measured water temp

for(i in 1:nsim){
  C_tc_me[i] <- 1e-6 * B * (V_g * (M_h - M_a_n2o) / (8.3144598 * (T_c_me[i] + 273.
}

# combine into dataframe for ggplot. qplot, which accepts vectors,
```

```
# was deprecated in ggplot 3.4.0
C_tc_df <- tibble(C_tc_me = C_tc_me, T_c_me = T_c_me)
# save absolute error for plot
C_tc_me_abs <- (C_tc_me * 1e9) - C
```

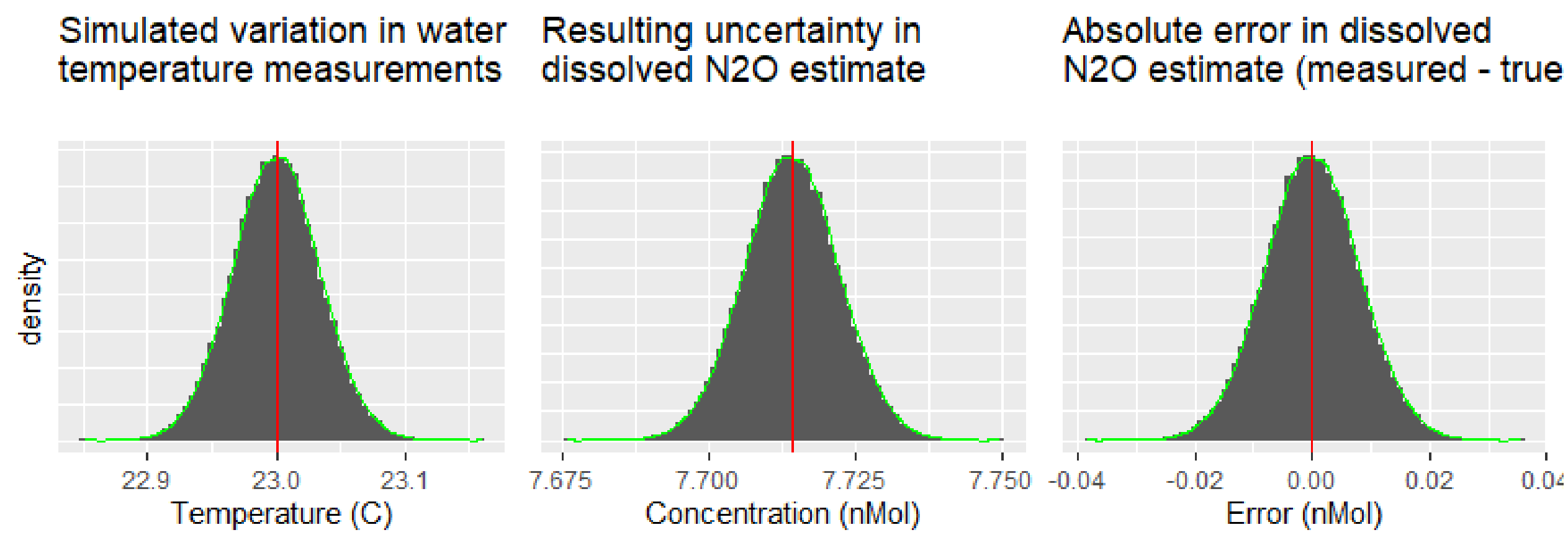

2.3.4 Barometric pressure

Next, error in barometric pressure measurements is simulated and its potential effect on the final dissolved N2O concentration assessed.

```
C_b_me <- rep(NA, nsim)

# How to get standard deviation of barometric pressure?
# The manual reports an "accuracy" of +/-3 mm Hg (+/-0.4kPa), but EPA calibration
# checks performed in the AWBERC metrology lab demonstrate much greater precision.
# below we calculat sd of replicate measurements made with 15 different barometers
# we then take the mean of the 15 sd values.

#2023 barometer verification in AWBERC metrology lab
B_sd <- mean(
  sd(c(999,998.6)*0.1), # YSI ProDSS
  sd(c(1000.848, 1000.848)*0.1), # EXO 21G104022
  sd(c(1000.982, 1000.982)*0.1), # EXO 18H111572
  sd(c(1000.982, 1000.982)*0.1), # EXO 22F105975
  sd(c(1001.382, 1001.382)*0.1), # EXO 21K100162
  sd(c(1000.98, 1000.85)*0.1), # EXO 21F103579
  sd(c(1000.31, 1000.44)*0.1), # MDS 16D100327
  sd(c(1001.25, 1001.25)*0.1), # MDS 12F102618
  sd(c(1000, 1000)*0.1), # MDS 16D100328
  sd(c(1002, 1002)*0.1), # MDS 02K599 AF
  sd(c(1003,1003)*0.1), # MDS 12J0793 AF
  sd(c(1000.85,1000.85)*0.1), # MDS 16E100831
  sd(c(1000.32, 1000.32)*0.1), # MDS 12F102619
```

```
  sd(c(1002, 1002)*0.1)) # MDS 15F104725

B_me <- rnorm(nsim, B, B_sd) #m.e. in measured barometric pressure

for(i in 1:nsim){
  C_b_me[i] <- 1e-6 * B_me[i] * (V_g * (M_h - M_a_n2o) / (8.3144598 * (T_c + 273.1
}

# combine into dataframe for ggplot. qplot, which accepts vectors,
# was deprecated in ggplot 3.4.0
C_b_df <- tibble(C_b_me = C_b_me, B_me = B_me)
# save absolute error for plot
C_b_me_abs <- (C_b_me * 1e9) - C
```

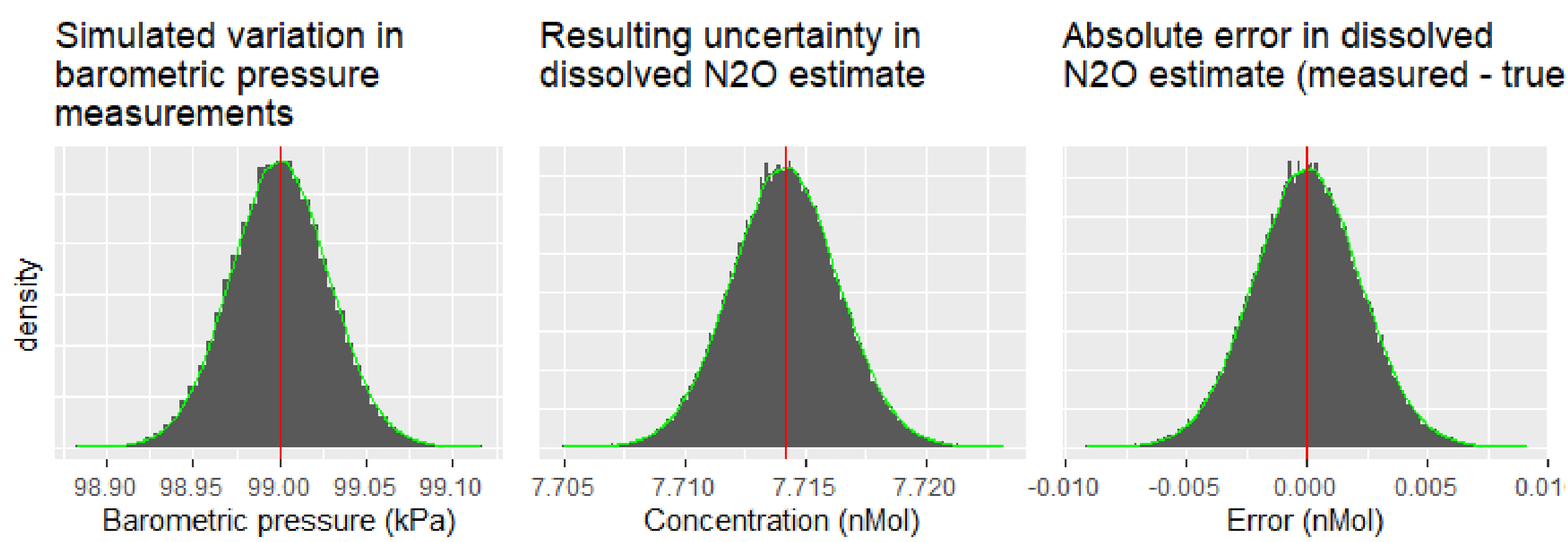

2.3.5 Water volume

Next, error in the volume of water (relative to volume of gas) in the sample vessel is simulated and its potential effect on the final dissolved N2O concentration assessed.

```
C_w_me <- rep(NA, nsim)

V_w_me <- rnorm(nsim, V_w, 1) #m.e. in measured water volume
V_g_me <- 140 - V_w_me

for(i in 1:nsim){
  C_w_me[i] <- 1e-6 * B * (V_g_me[i] * (M_h - M_a_n2o) / (8.3144598 * (T_c + 273.1
}

# combine into dataframe for ggplot. qplot, which accepts vectors,
# was deprecated in ggplot 3.4.0
C_w_df <- tibble(C_w_me = C_w_me, V_w_me = V_w_me)
```

```
# save absolute error for plot
C_w_me_abs <- (C_w_me * 1e9) - C
```

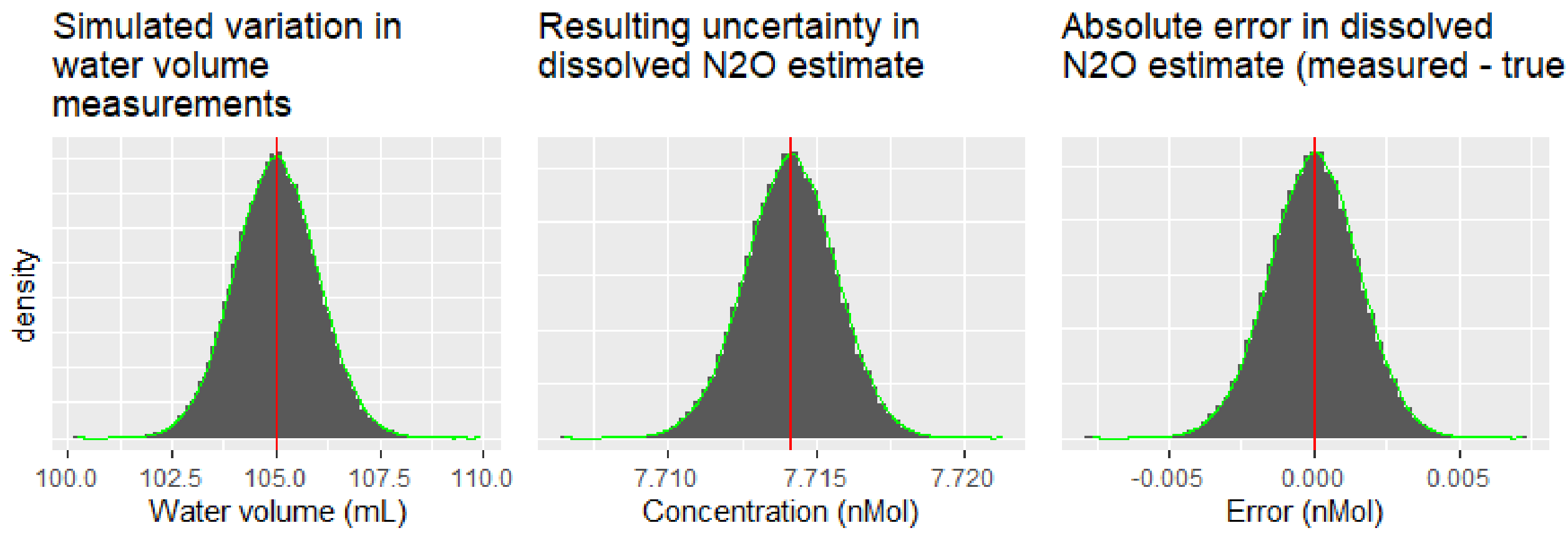

2.3.6 Mixing ratio

To check the effect of different mixing ratios, we re-arrange the equation to solve for M_h, given a known C from above where $C = 7.7$. For both ambient air and a pure gas (e.g., helium) as the headspace gas, we evaluate three scenarios for the mixing ratio, R_M. The R_M value indicates the proportion of gas relative to water in the headspace mixture. In the simulations below, we set R_M to be (1) very low ($R_{M(low)} = 0.1$), (2) at equal mixing proportions ($R_{M(equal)} = 0.5$), and (3) very high ($R_{M(high)} = 0.9$). For reference, in the simulations above, and in the empirical field measurments, R_M was set at 0.25. Also note that the total volume of the mixing vessel in the field study was 140mL, so the volume of gas in the mixing vessel was intended to be $R_M \cdot V_g = 35$mL and the volume of water was $R_M \cdot V_w = 105$mL. Finally, note that varying the total volume of the mixing vessel has no consequence for C, only the ratio of gas to water.

2.3.6.1 Ambient air

For the same fixed concentration, $C = 7.7$, using a low proportion of gas relative to water ($R_{M(low)} = 0.1$) in the equilibrated headspace results in a smaller value of M_h relative to the simulations above with $R_M = 0.25$. This simulation assumes ambient air is used as the headspace gas.

```
# Set volume of syringe/vessel
V <- 140 # 140mL syringe
```

```
# Set ratio of gas volume to water volume in the mixing vessel to something
# smaller than the scenario above
R_M_low <- 0.1

C <- C # known N2O concentration in sample from simulation above
B <- 99 # barometric pressure (kPa)
V_g_low <- V * R_M_low # volume of reference gas in headspace
V_w_low <- V * (1 - R_M_low) # volume of water below headspace
T_c <- 23 # temperature ( C )
M_a_n2o <- 0.310 # gas measured in reference (air)
H_n2o <- 0.00024 #mol m-3 Pa, range: 0.00018 - 0.00025; Henry's law constant

#### Re-arranged previous equation to solve for Mh (with low proportion of gas to
M_h_low <- (((C / 1e9) * 8.3144598 * (T_c + 273.15) * V_w_low) + (1e-6 * B * V_g_l

round(M_h_low, 3)
```

```
## [1] 0.306
```

Next, we calculate M_h for equal proportions of gas and water in the headspace, such that $R_{M(equal)} = 0.5$. In this case we get a slightly higher value for M_h relative to the low mixing proportion above.

```
# Set volume of syringe/vessel
V <- 140 # 140mL syringe

# Set ratio of gas volume to water volume in the mixing vessel to something
# smaller than the scenario above
R_M_equal <- 0.5

C <- C # known N2O concentration in sample from simulation above
B <- 99 # barometric pressure (kPa)
V_g_equal <- V * R_M_equal # volume of reference gas in headspace
V_w_equal <- V * (1 - R_M_equal) # volume of water below headspace
T_c <- 23 # temperature ( C )
M_a_n2o <- 0.310 # gas measured in reference (air)
H_n2o <- 0.00024 #mol m-3 Pa, range: 0.00018 - 0.00025; Henry's law constant

#### Re-arranged previous equation to solve for Mh
M_h_equal <- (((C / 1e9) * 8.3144598 * (T_c + 273.15) * V_w_equal) + (1e-6 * B * V

round(M_h_equal, 3)
```

```
## [1] 0.308
```

Next, we calculate M_h for a higher proportion of gas relative to water in the headspace, such that $R_{M(high)} = 0.9$. In this case we again get a higher value for M_h.

```
# Set volume of syringe/vessel
V <- 140 # 140mL syringe

# Set ratio of gas volume to water volume in the mixing vessel to something
# smaller than the scenario above
R_M_high <- 0.9

C <- C # known N2O concentration in sample from simulation above
B <- 99 # barometric pressure (kPa)
V_g_high <- V * R_M_high # volume of reference gas in headspace
V_w_high <- V * (1 - R_M_high) # volume of water below headspace
T_c <- 23 # temperature ( C )
M_a_n2o <- 0.310 # gas measured in reference (air)
H_n2o <- 0.00024 #mol m-3 Pa, range: 0.00018 - 0.00025; Henry's law constant

#### Re-arranged previous equation to solve for Mh
M_h_high <- (((C / 1e9) * 8.3144598 * (T_c + 273.15) * V_w_high) + (1e-6 * B * V_g

round(M_h_high, 3)
```

```
## [1] 0.31
```

Finally, we simulate C again with measurement error on M_h, but using the values of M_h obtained above using the 3 different values of R_M.

2.3.6.1.1 $R_{M(low)}$

```
C_h_me_low <- rep(NA, nsim)

M_h_me_low <- rnorm(nsim, M_h_low, n2o_sd_epa)

for(i in 1:nsim){
  C_h_me_low[i] <- 1e-6 * B * (V_g_low * (M_h_me_low[i] - M_a_n2o) / (8.3144598 *
}

# combine into dataframe for ggplot.
```

```
C_h_low_df <- tibble(C_h_me_low = C_h_me_low, M_h_me_low = M_h_me_low)
# save absolute error for plot
C_h_me_low_abs <- (C_h_me_low * 1e9) - C
```

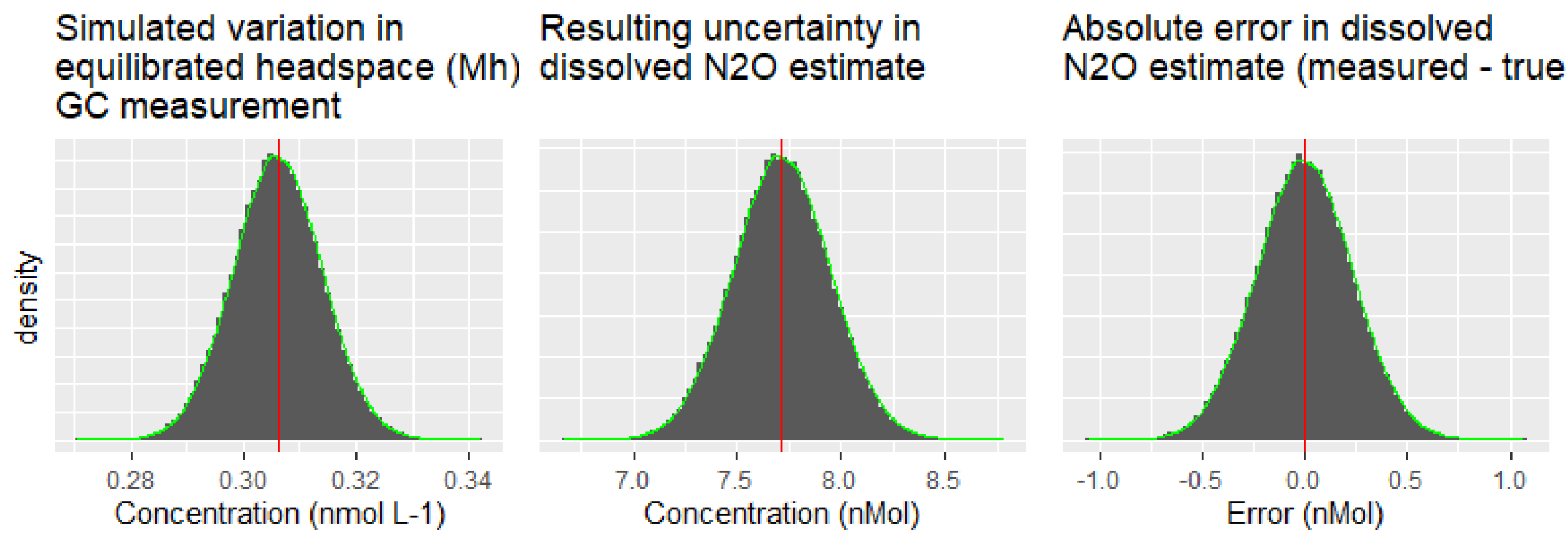

2.3.6.1.2 $R_{M\,(equal)}$

```
C_h_me_equal <- rep(NA, nsim)

M_h_me_equal <- rnorm(nsim, M_h_equal, n2o_sd_epa)

for(i in 1:nsim){
  C_h_me_equal[i] <- 1e-6 * B * (V_g_equal * (M_h_me_equal[i] - M_a_n2o) / (8.3144
}

# combine into dataframe for ggplot.
C_h_equal_df <- tibble(C_h_me_equal = C_h_me_equal, M_h_me_equal = M_h_me_equal)
# save absolute error for plot
C_h_me_equal_abs <- (C_h_me_equal * 1e9) - C
```

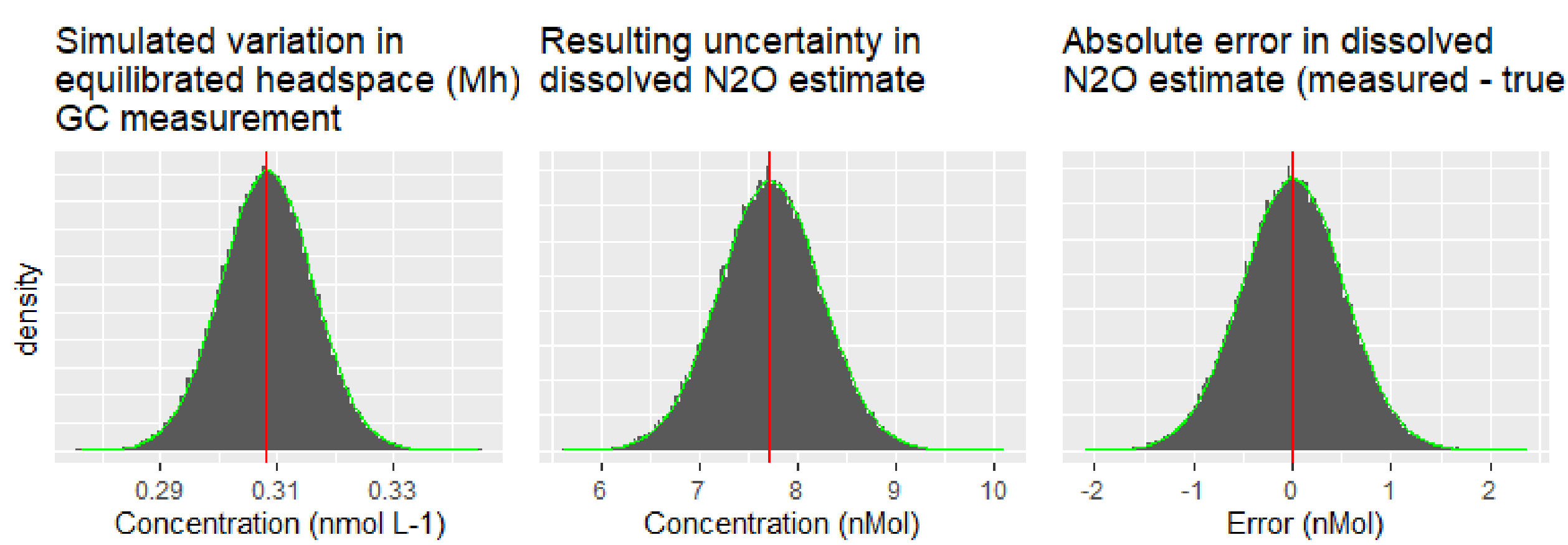

2.3.6.1.3 $R_{M\,(high)}$

```
C_h_me_high <- rep(NA, nsim)

M_h_me_high <- rnorm(nsim, M_h_high, n2o_sd_epa)

for(i in 1:nsim){
  C_h_me_high[i] <- 1e-6 * B * (V_g_high * (M_h_me_high[i] - M_a_n2o) / (8.3144598
}

# combine into dataframe for ggplot.
C_h_high_df <- tibble(C_h_me_high = C_h_me_high, M_h_me_high = M_h_me_high)
# save absolute error for plot
C_h_me_high_abs <- (C_h_me_high * 1e9) - C
```

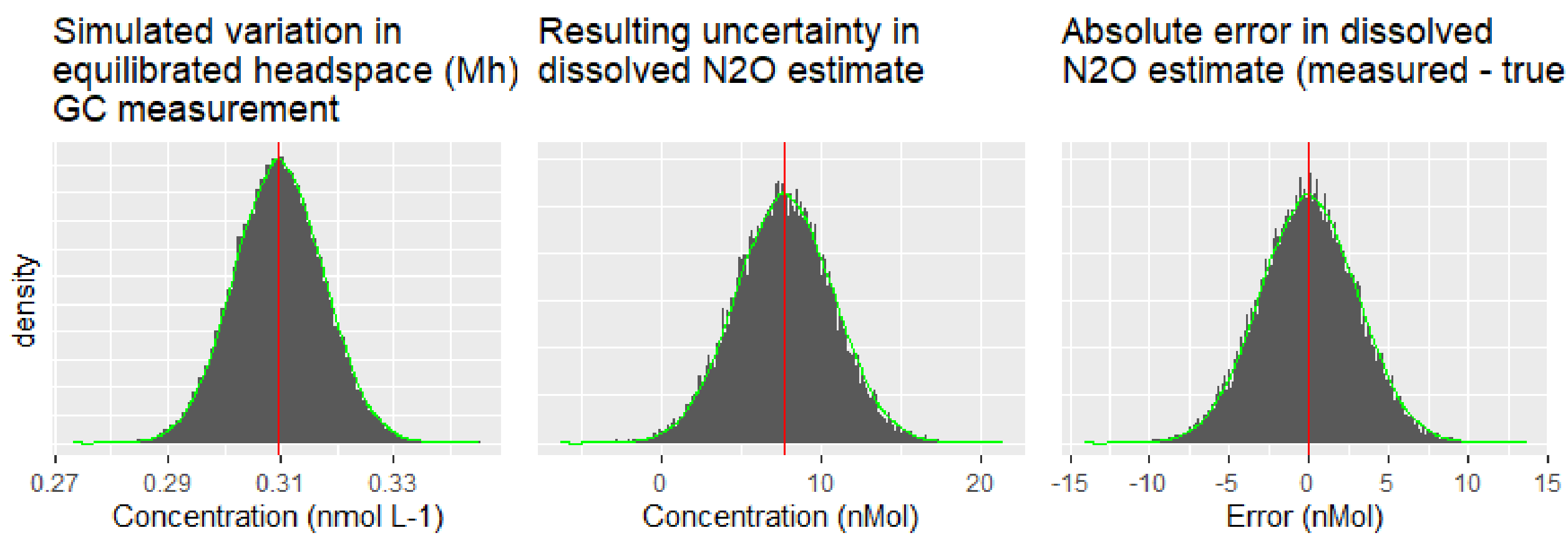

2.3.6.2 Pure gas

To check the effect of different mixing ratios using a pure gas such as helium as the headspace, we again re-arrange the equation to solve for M_h, given known C. In this case with a pure gas, we set $M_a = 0$.

For the same fixed concentration, C, using a low proportion of pure gas relative to water ($R_{M(low)} = 0.1$) in the equilibrated headspace results in a much smaller value of M_h relative to the original simulations (and field method) where $R_M = 0.25$. This is because the mixing ratio is very low and also because there is no need to account for ambient air in the headspace.

```
# Set volume of syringe/vessel
V <- 140 # 140mL syringe

# Set ratio of gas volume to water volume in the mixing vessel to something
# smaller than the scenario above
```

```
R_M_low <- 0.1

C <- C # known N2O concentration in sample from simulation above
B <- 99 # barometric pressure (kPa)
V_g_low <- V * R_M_low # volume of reference gas in headspace
V_w_low <- V * (1 - R_M_low) # volume of water below headspace
T_c <- 23 # temperature ( C )
H_n2o <- 0.00024 #mol m-3 Pa, range: 0.00018 - 0.00025; Henry's law constant

#### Re-arranged previous equation to solve for Mh
M_h_pg_low <- (((C / 1e9) * 8.3144598 * (T_c + 273.15) * V_w_low) + (1e-6 * B * V_

round(M_h_pg_low, 3)
```

```
## [1] 0.26
```

Next, we calculate M_h for equal proportions of pure gas and water in the headspace, such that $R_{M(equal)} = 0.5$. In this case we get a substantially lower value for M_h relative to the low mixing proportion above.

```
# Set volume of syringe/vessel
V <- 140 # 140mL syringe

# Set ratio of gas volume to water volume in the mixing vessel to something
# smaller than the scenario above
R_M_equal <- 0.5

C <- C # known N2O concentration in sample from simulation above
B <- 99 # barometric pressure (kPa)
V_g_equal <- V * R_M_equal # volume of reference gas in headspace
V_w_equal <- V * (1 - R_M_equal) # volume of water below headspace
T_c <- 23 # temperature ( C )
H_n2o <- 0.00024 #mol m-3 Pa, range: 0.00018 - 0.00025; Henry's law constant

#### Re-arranged previous equation to solve for Mh
M_h_pg_equal <- (((C / 1e9) * 8.3144598 * (T_c + 273.15) * V_w_equal) + (1e-6 * B

round(M_h_pg_equal, 3)
```

```
## [1] 0.118
```

Next, we calculate M_h for a higher proportion of pure gas relative to water in the headspace, such that $R_{M(high)} = 0.9$. In this case we again get a very low value for M_h.

```
# Set volume of syringe/vessel
V <- 140 # 140mL syringe

# Set ratio of gas volume to water volume in the mixing vessel to something
# smaller than the scenario above
R_M_high <- 0.9

C <- C # known N2O concentration in sample from simulation above
B <- 99 # barometric pressure (kPa)
V_g_high <- V * R_M_high # volume of reference gas in headspace
V_w_high <- V * (1 - R_M_high) # volume of water below headspace
T_c <- 23 # temperature ( C )
H_n2o <- 0.00024 #mol m-3 Pa, range: 0.00018 - 0.00025; Henry's law constant

#### Re-arranged previous equation to solve for Mh
M_h_pg_high <- (((C / 1e9) * 8.3144598 * (T_c + 273.15) * V_w_high) + (1e-6 * B *

round(M_h_pg_high, 3)
```

```
## [1] 0.02
```

Below, we again simulate C with measurment error on M_h, using the values of M_h obtained above using the 3 different values of R_M with the hypothetical pure gas headspace mixture.

2.3.6.2.1 $R_{M(low)}$

```
C_h_pg_me_low <- rep(NA, nsim)

M_h_pg_me_low <- rnorm(nsim, M_h_pg_low, n2o_sd_epa)

for(i in 1:nsim){
  C_h_pg_me_low[i] <- 1e-6 * B * (V_g_low * (M_h_pg_me_low[i] - 0) / (8.3144598 *
}

# combine into dataframe for ggplot.
C_h_pg_low_df <- tibble(C_h_pg_me_low = C_h_pg_me_low, M_h_pg_me_low = M_h_pg_me_l
```

```
# save absolute error for plot
C_h_pg_me_low_abs <- (C_h_pg_me_low * 1e9) - C
```

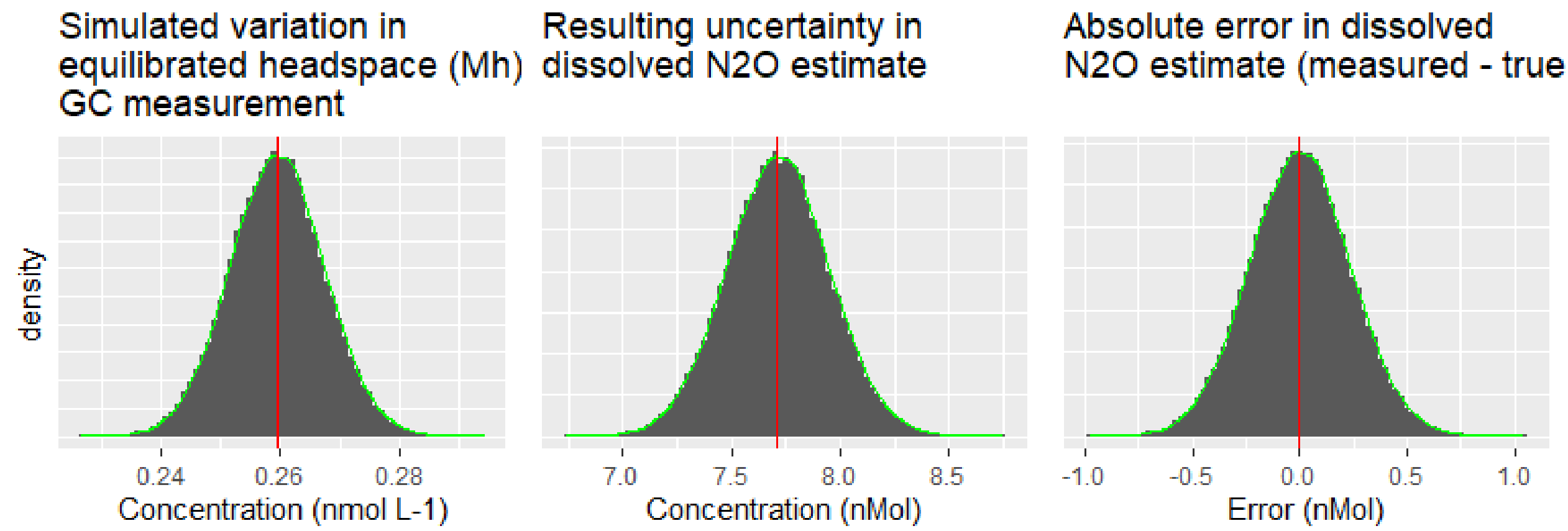

2.3.6.2.2 $R_{M(equal)}$

```
C_h_pg_me_equal <- rep(NA, nsim)

M_h_pg_me_equal <- rnorm(nsim, M_h_pg_equal, n2o_sd_epa)

for(i in 1:nsim){
  C_h_pg_me_equal[i] <- 1e-6 * B * (V_g_equal * (M_h_pg_me_equal[i] - 0) / (8.3144
}

# combine into dataframe for ggplot.
C_h_pg_equal_df <- tibble(C_h_pg_me_equal = C_h_pg_me_equal, M_h_pg_me_equal = M_h
# save absolute error for plot
C_h_pg_me_equal_abs <- (C_h_pg_me_equal * 1e9) - C
```

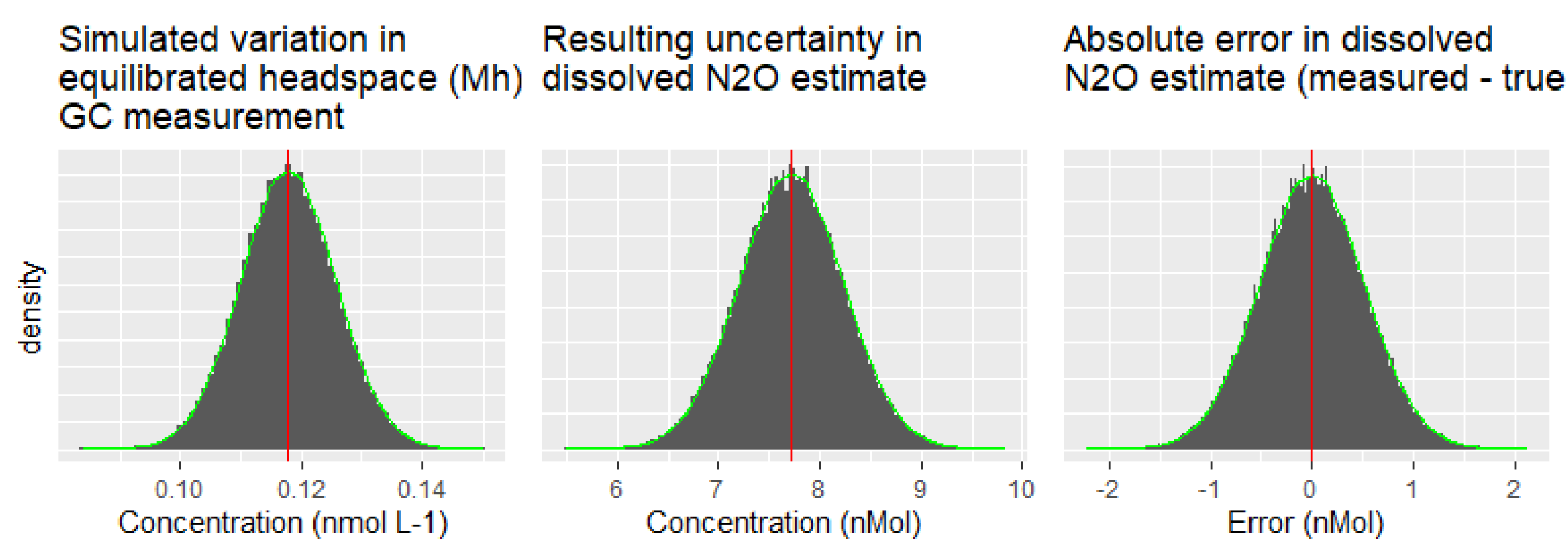

2.3.6.2.3 $R_{M(high)}$

```
C_h_pg_me_high <- rep(NA, nsim)

M_h_pg_me_high <- rnorm(nsim, M_h_pg_high, n2o_sd_epa)

for(i in 1:nsim){
  C_h_pg_me_high[i] <- 1e-6 * B * (V_g_high * (M_h_pg_me_high[i] - 0) / (8.3144598
}

# combine into dataframe for ggplot.
C_h_pg_high_df <- tibble(C_h_pg_me_high = C_h_pg_me_high, M_h_pg_me_high = M_h_pg_
# save absolute error for plot
C_h_pg_me_high_abs <- (C_h_pg_me_high * 1e9) - C
```

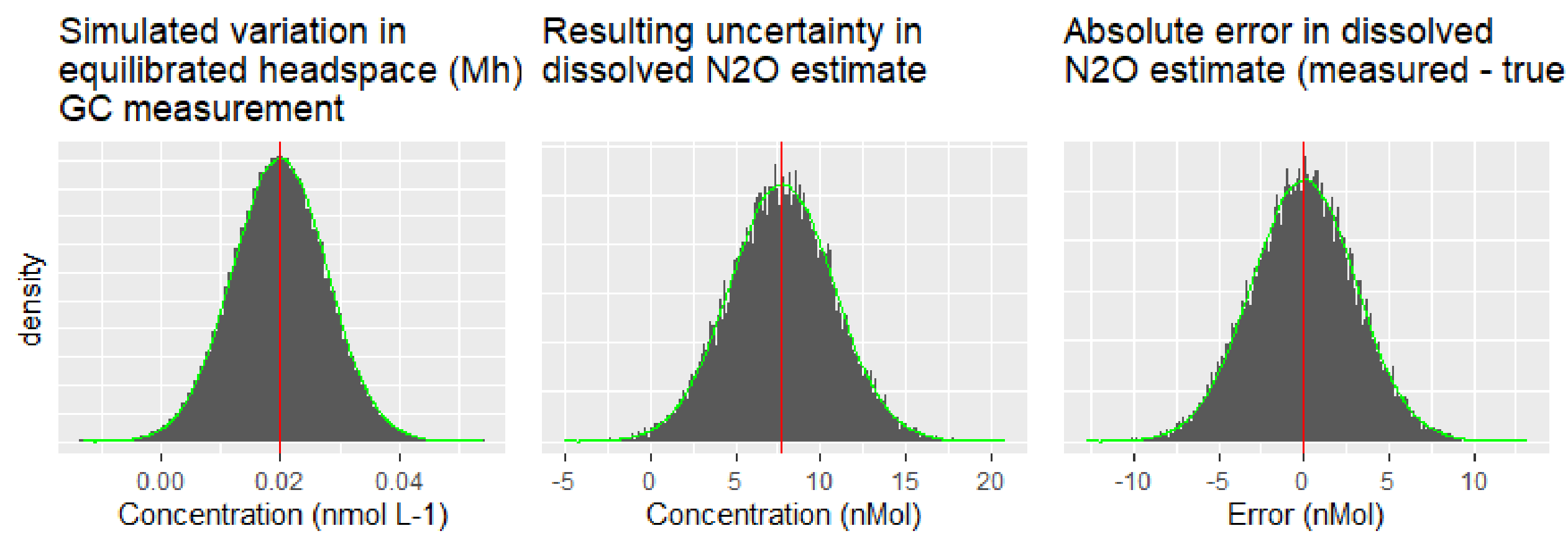

2.3.7 All observables

In order to get a sense of the total error in a measurement resulting from the sources above, we simulate measurement error for all of the observables (*i.e.*, GC measure of gas in equilibrated headspace, GC measure of gas in air, water temperature, and barometric pressure) and assess the resulting effects on the dissolved N2O concentration measure. In this scenario, it is assumed that, as with the empirical field measurements, air is used as the headspace gas and the mixing ratio is $R_M = $ 0.25.

```
C_all_me <- rep(NA, nsim)

for(i in 1:nsim){
  C_all_me[i] <- 1e-6 * B_me[i] * (V_g_me[i] * (M_h_me[i] - M_a_n2o_me[i] ) / (8.3
}

# combine into dataframe for ggplot. qplot, which accepts vectors,
# was deprecated in ggplot 3.4.0
C_all_df <- tibble(C_all_me = C_all_me)
```

```
# save absolute error for plot
C_all_me_abs <- (C_all_me * 1e9) - C
```

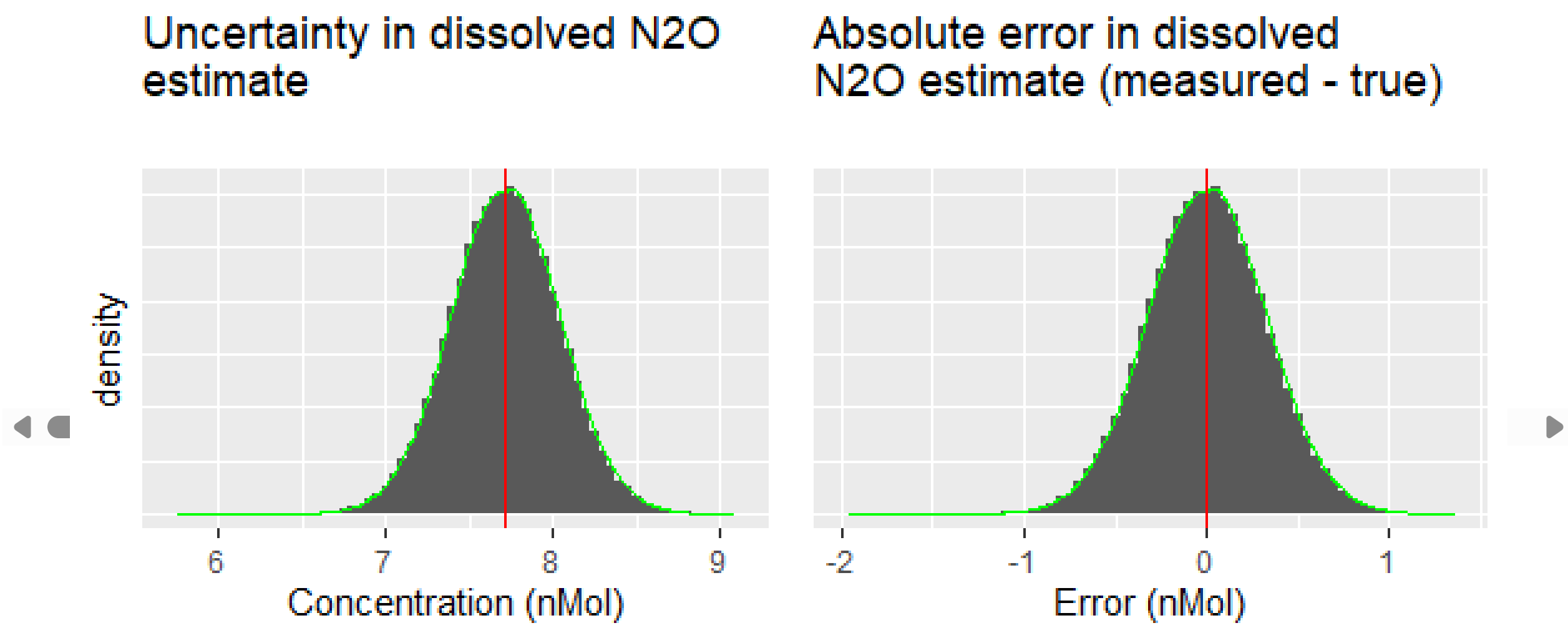

The estimated standard deviations of dissolved N2O measurements and the resulting 95% credible interval for error in measurements is printed below.

```
# standard deviation of a measurement is:
sd(C_all_me* 1e9) # 0.20 nmol L-1
```

```
## [1] 0.3230534
```

```
# 2 standard deviations
sd(C_all_me * 1e9) * 1.96 # +/- 0.395 nmol N2O
```

```
## [1] 0.6331846
```

Alternatively, calculate the central 95th percentile of the simulated measures.

```
# standard deviation of a measurement is:
round(sd(C_all_me_abs), 3) # 0.20 nmol L-1
```

```
## [1] 0.323
```

```
# the 95% CI for an observation with measurement error can be computed
# approximately as the value +/- 2 standard deviations; or the central 95th
# percentile of the absolute error distribution.
round(quantile((C + C_all_me_abs), probs = c(0.025, 0.975)), 3) # +/- nmol N2O
```

```
##  2.5% 97.5%
## 7.081 8.348
```

2.4 Summary of contributions to error

Below, we compare the total error estimated for all observables in the last simulation, to the error estimated from individual sources.

```
results_dissolved_N2O <- tibble(Eh_me= C_h_me_abs,
                                Air_me = C_a_me_abs,
                                Tc_me = C_tc_me_abs,
                                B_me = C_b_me_abs,
                                W_me = C_w_me_abs,
                                All_me = C_all_me_abs) %>%
  pivot_longer(cols = ends_with("me"), names_to = "Source", values_to = "Error")
```

```
results_dissolved_N2O %>%
  mutate(Source = factor(Source)) %>%
  mutate(Source = fct_relevel(Source, c("B_me",
                                        "W_me",
                                        "Tc_me",
                                        "Air_me",
                                        "Eh_me",
                                        "All_me"
                                        ))) %>%
  ggplot(aes(x = Error, y = Source)) +
  stat_pointinterval() +
  scale_y_discrete(labels=c("Eh_me" = "Equilibrated headspace",
                            "Air_me" = "Ambient air",
                            "Tc_me" = "Water temperature",
                            "B_me" = "Barometric pressure",
                            "W_me" = "Water volume",
                            "All_me" = "All sources")) +
  xlab("Absolute error in dissolved \nN2O estimate (measured - true) \n") +
  ylab("")
```

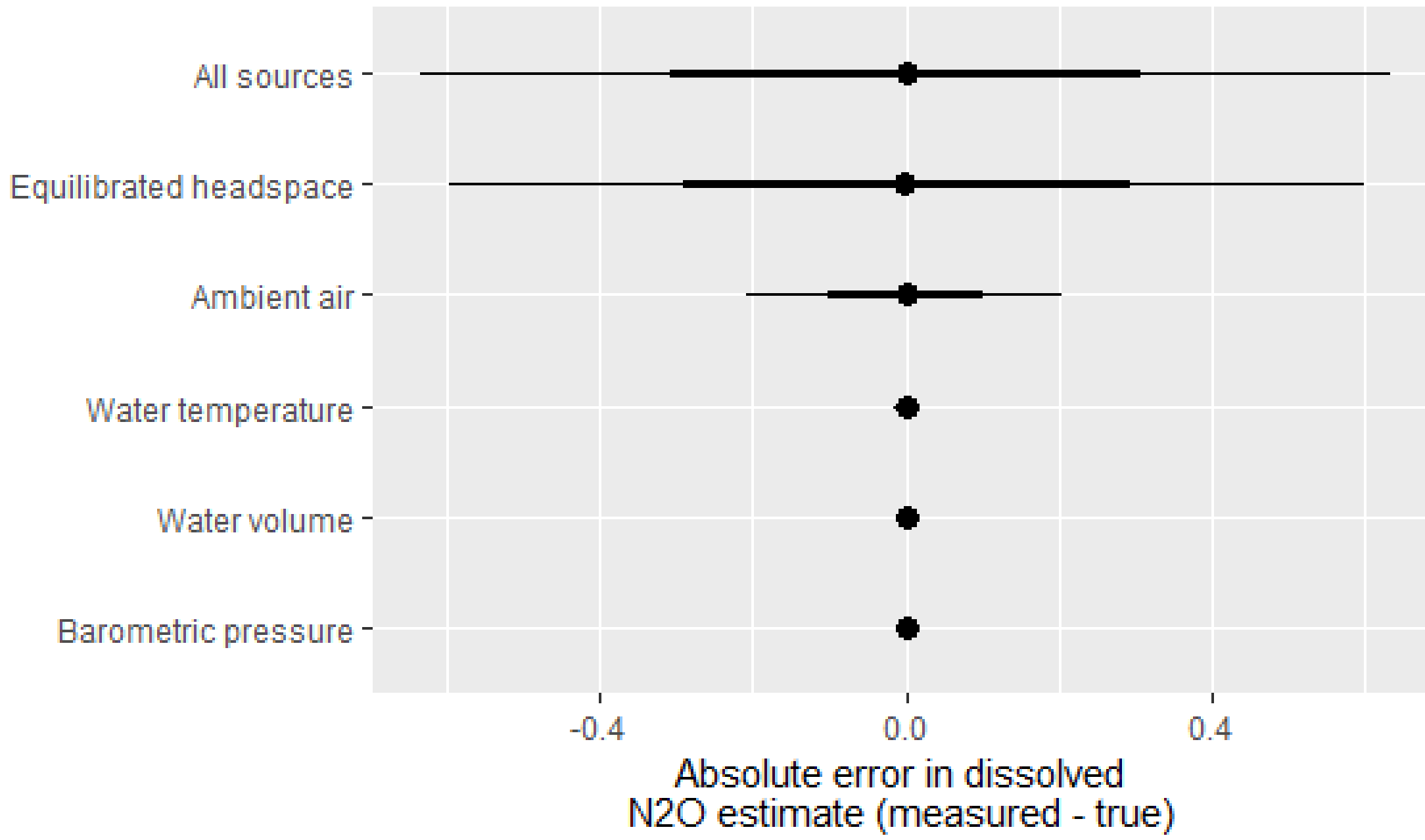

3 Equilibrium concentration

The equation for estimating the concentration (nM) of (N_2O) gas dissolved in water that is in equilibrium with the atmosphere is:

$$C_{eq} = 1^{-6} \cdot B \cdot C_a \cdot H^{\theta} \cdot e^{2700 \cdot \frac{1}{Tc} - \frac{1}{298.15}}$$

where:

- C_{eq} = The equilibrium concentration in nM
- B = barometric pressure (kPa)
- C_a = The air concentration in nM
- H^{θ} = Henry's law constant (H_{N_2O} below)
- T_c = temperature (K) = T_c (Celsius) + 273.15

3.1 Simulate equilibrium N_2O

A "known" concentration is calculated:

```
C_eq <- M_a_n2o * B * 1e-6 * (H_n2o  * exp(2700*(1 / (T_c + 273.15) - 1 / 298.15))
```

```
C_eq <- C_eq * 1e9 # convert mol to nmol
```

3.2 Simulate measurement error

3.2.1 Ambient air

Error in the GC measurements of N_2O in the ambient air (M_a) is simulated below. The resulting effects on the uncertainty of the equilibrium N2O measurement is plotted along with the absolute error in N2O as a result of this measurement.

```
C_a_eq_me <- rep( NA, nsim )

M_a_n2o_me <- rnorm( nsim, M_a_n2o, n2o_sd_epa ) #m.e. in measured gas in air


for(i in 1:nsim){
    C_a_eq_me[i] <- M_a_n2o_me[i] * B * 1e-6 * (H_n2o  * exp(2700*(1 / (T_c + 273.
}

C_a_eq_df <- tibble(C_a_eq_me = C_a_eq_me, M_a_n2o_me = M_a_n2o_me)
# save absolute error for plot
C_a_eq_me_abs <- (C_a_eq_me * 1e9) - C_eq
```

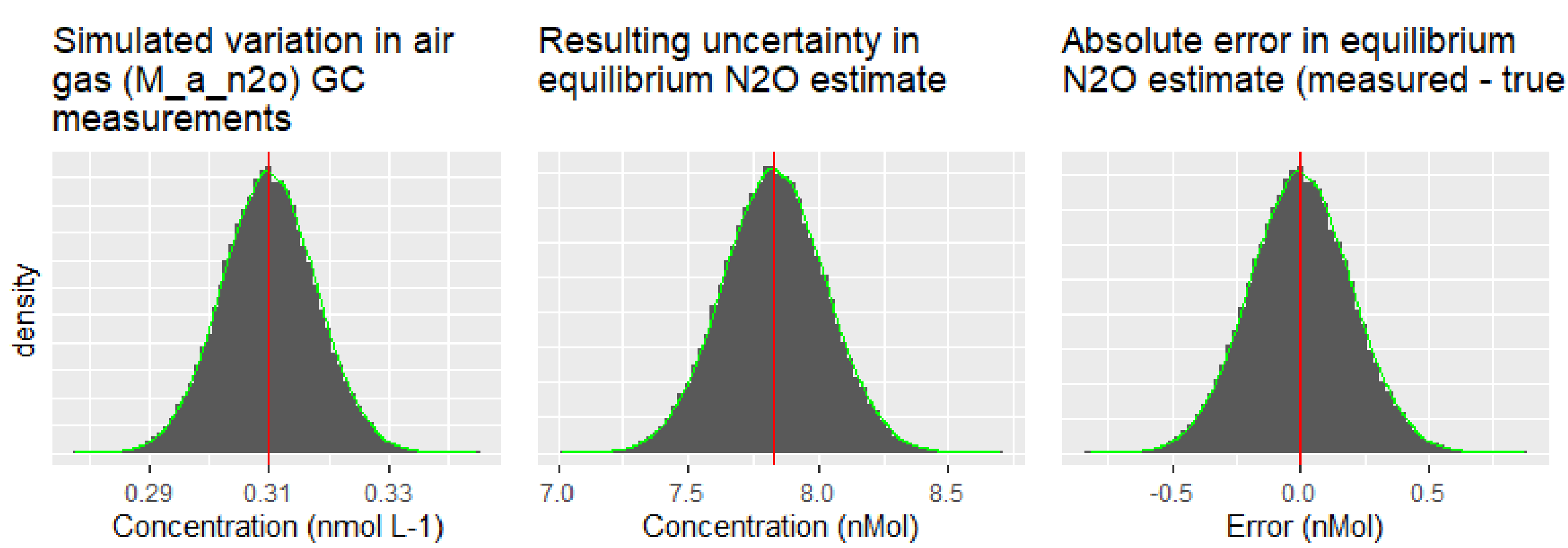

3.2.2 Water temperature

```
C_tc_eq_me <- rep(NA, nsim)

for(i in 1:nsim){
```

```
    C_tc_eq_me[i] <- M_a_n2o * B * 1e-6 * (H_n2o  * exp(2700 * (1 / (T_c_me[i] + 2
}

# combine into dataframe for ggplot. qplot, which accepts vectors,
# was deprecated in ggplot 3.4.0
C_tc_eq_df <- tibble(C_tc_eq_me = C_tc_eq_me, T_c_me = T_c_me)
# save absolute error for plot
C_tc_eq_me_abs <- (C_tc_eq_me * 1e9) - C_eq
```

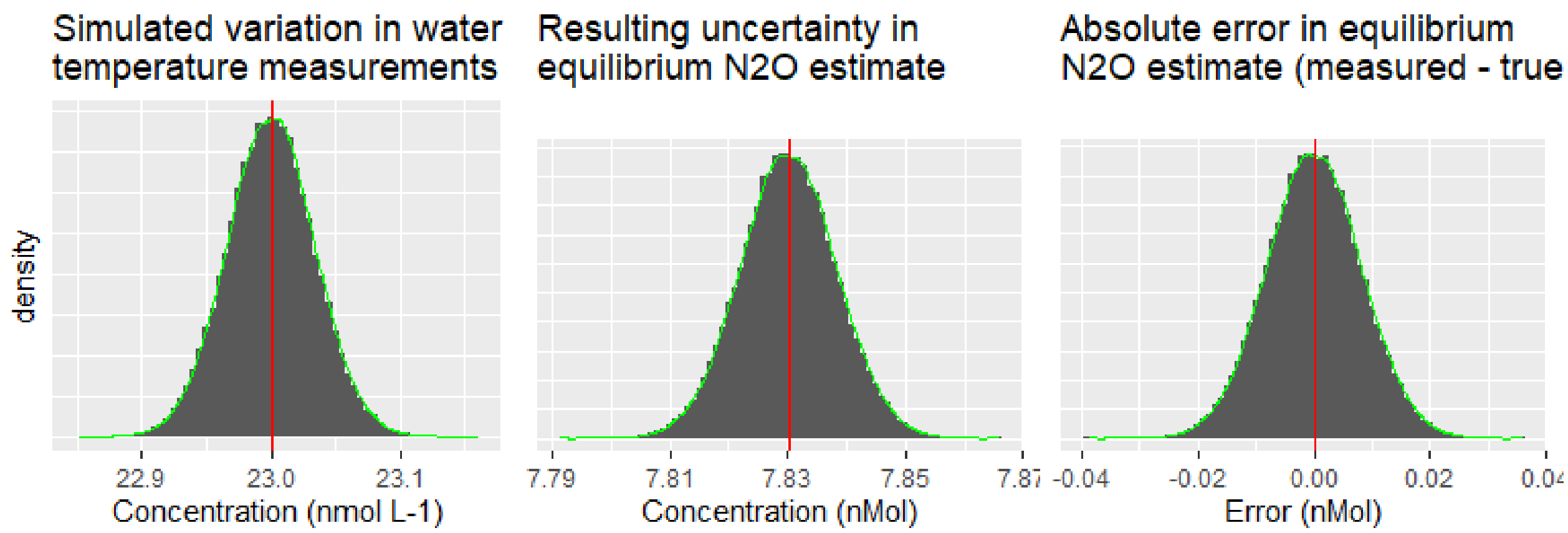

3.2.3 Barometric pressure

```
C_b_eq_me <- rep(NA, nsim)

for(i in 1:nsim){
    C_b_eq_me[i] <- M_a_n2o * B_me[i] * 1e-6 * (H_n2o  * exp(2700 * (1 / (T_c + 27
}

# combine into dataframe for ggplot. qplot, which accepts vectors,
# was deprecated in ggplot 3.4.0
C_b_eq_df <- tibble(C_b_eq_me = C_b_eq_me, B_me = B_me)
# save absolute error for plot
C_b_eq_me_abs <- (C_b_eq_me * 1e9) - C_eq
```

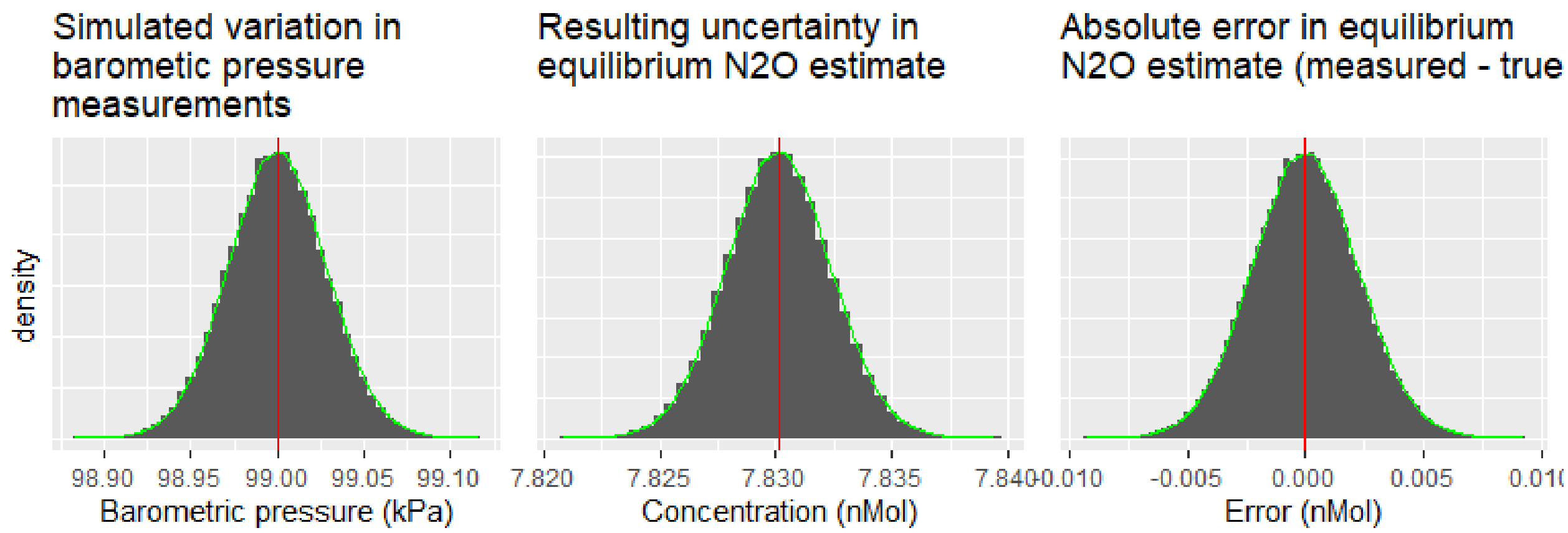

3.2.4 All observables

Simulate measurement error for all observables (*i.e.*, air, water temperature, and barometric pressure) and the resulting effects on equilibrium N2O observations.

```
C_all_eq_me <- rep( NA, nsim )

for(i in 1:nsim){
    C_all_eq_me[i] <- M_a_n2o_me[i] * B_me[i] * 1e-6 * (H_n2o  * exp(2700 * (1 / (
}

# combine into dataframe for ggplot. qplot, which accepts vectors,
# was deprecated in ggplot 3.4.0
C_all_eq_df <- tibble(C_all_eq_me = C_all_eq_me)
# save absolute error for plot
C_all_eq_me_abs <- (C_all_eq_me * 1e9) - C_eq
```

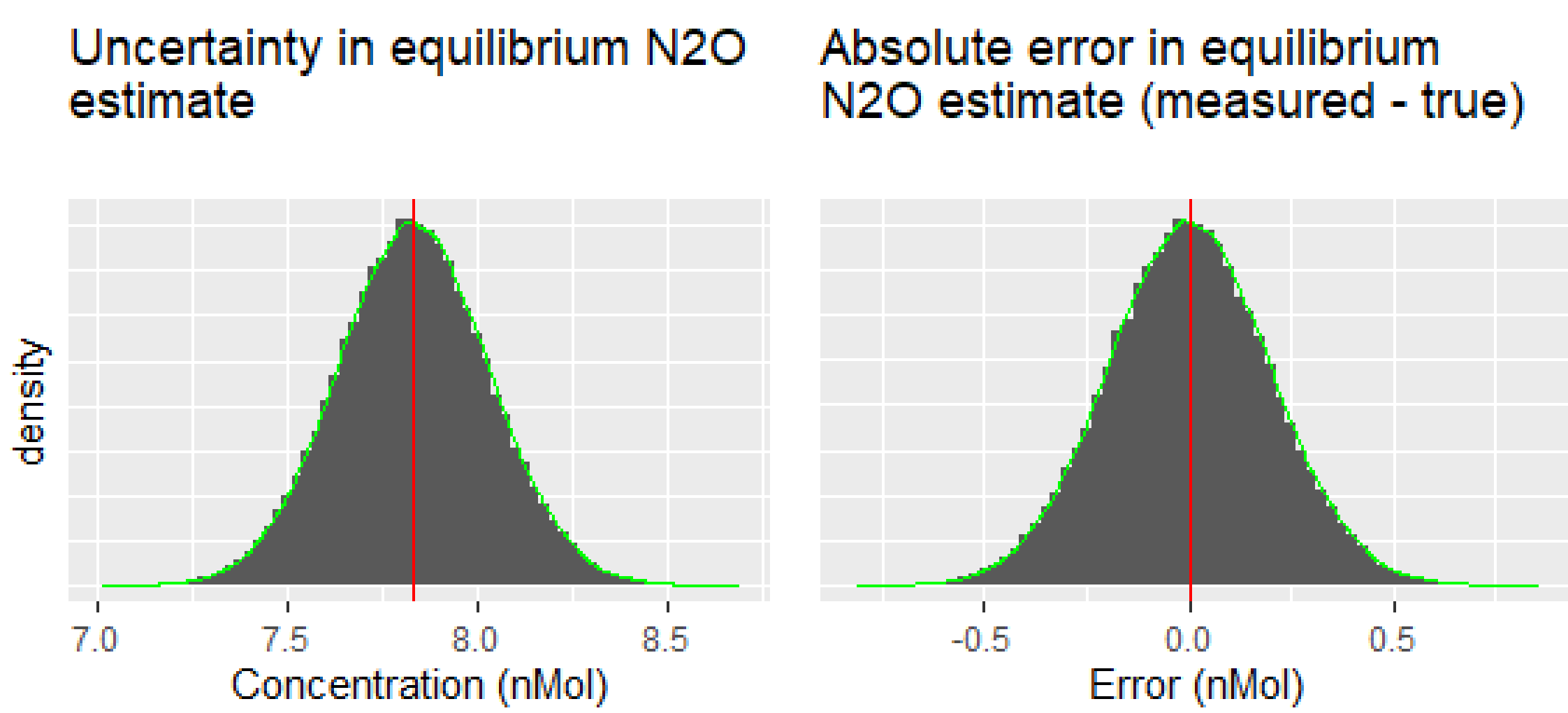


```
# standard deviation of a measurement is:
```

```
sd(C_all_eq_me* 1e9) # 0.20 nmol L-1
```

```
## [1] 0.1982652
```

```
# 2 standard deviations
sd(C_all_eq_me * 1e9) * 1.96 # +/- 0.395 nmol N2O
```

```
## [1] 0.3885998
```

```
# standard deviation of a measurement is:
round(sd(C_all_eq_me_abs), 3) # 0.20 nmol L-1
```

```
## [1] 0.198
```

```
# the 95% CI for an observation with measurement error can be computed
# approximately as the value +/- 2 standard deviations; or the central 95th
# percentile of the absolute error distribution.
round(quantile((C_eq + C_all_eq_me_abs), probs = c(0.025, 0.975)), 3) # +/- nmol N
```

```
##  2.5% 97.5%
## 7.442 8.221
```

3.3 Summary of contributions to error

```
results_equilibrium_N2O <- tibble(Air_me = C_a_eq_me_abs,
                                  Tc_me = C_tc_eq_me_abs,
                                  B_me = C_b_eq_me_abs,
                                  All_me = C_all_eq_me_abs) %>%
  pivot_longer(cols = ends_with("me"), names_to = "Source", values_to = "Error")
```

```
results_equilibrium_N2O %>%
  mutate(Source = factor(Source)) %>%
  mutate(Source = fct_relevel(Source, c("B_me",
```

```
                                                        "Tc_me",
                                                        "Air_me",
                                                        "All_me"
                                                        ))) %>%
ggplot(aes(x = Error, y = Source)) +
stat_pointinterval() +
scale_y_discrete(labels=c("Air_me" = "Ambient air",
                          "Tc_me" = "Water temperature",
                          "B_me" = "Barometric pressure",
                          "All_me" = "All sources")) +
xlab("Absolute error in equilibrium \nN2O estimate (measured - true) \n") +
ylab("")
```

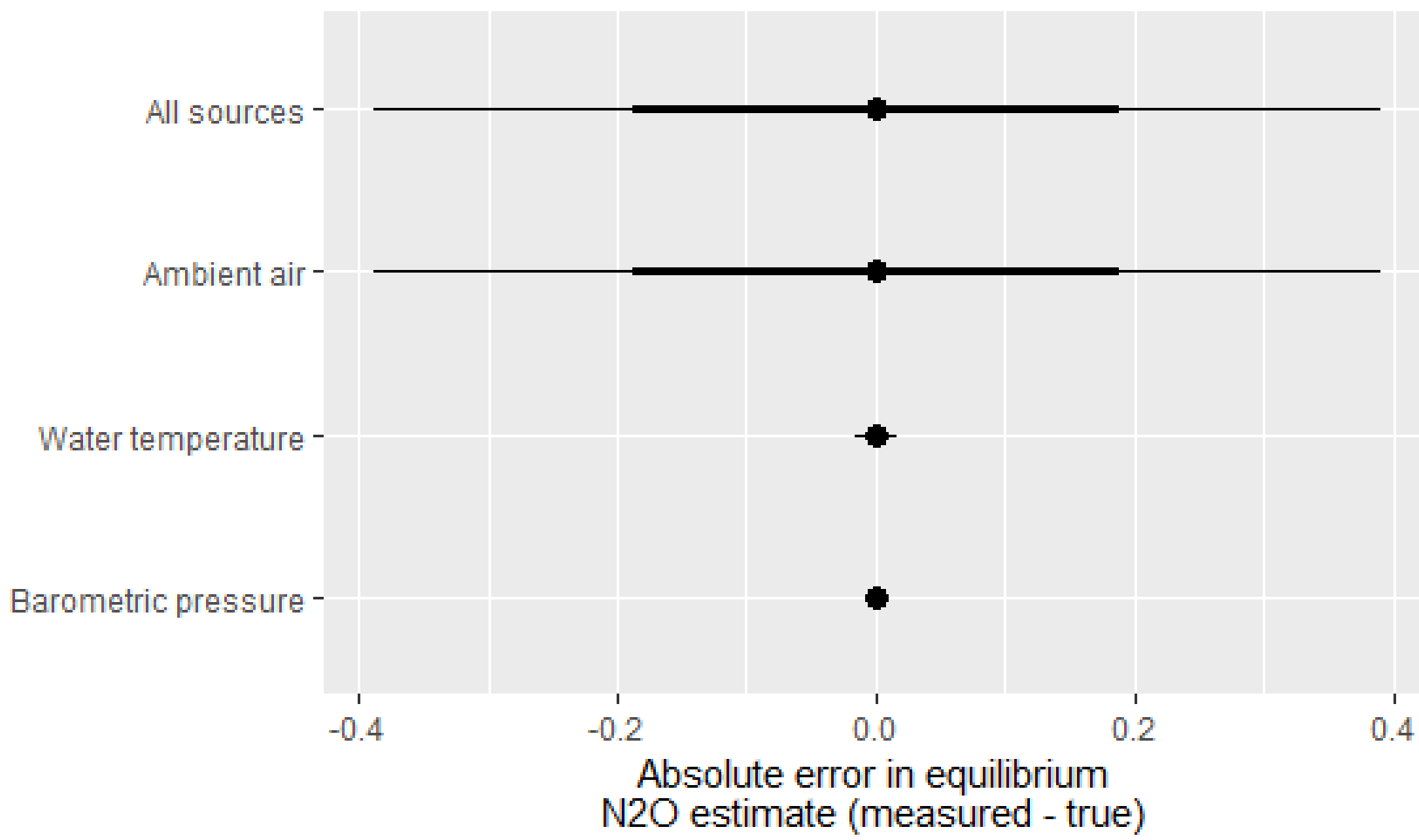

4 Saturation ratio

The equation for estimating the saturation ratio for (N_20) gas in water is:

$$S = \left(\frac{(1^{-6} \cdot B)(\frac{V_a(Mr_h - Mr_r)}{(8.3144598 \cdot K \cdot V_w) + H^{\theta} \cdot e^{2700 \cdot (\frac{1}{K} - \frac{1}{298.15})} \cdot Mr_h})}{1^{-6} \cdot B \cdot C_a \cdot e^{2700 \cdot \frac{1}{Tc} - \frac{1}{298.15}}} \right)$$

where:

- S = Saturatio ratio

- B = barometric pressure (kPa)
- C_a = the air concentration in nM
- V_a = volume of reference gas in air
- M_h = gas measured in the headspace
- M_r = gas measured in the reference (air)
- V_w = volume of water below the headspace
- T_c = temperature celsius
- H^{θ} = Henry's law constant (H_{N_2O} below)

Simulate a "true" saturation ratio.

```
S <- (1e-6 * B * (V_g * (M_h - M_a_n2o) / (8.3144598 * (T_c + 273.15) * V_w) + H_n
  (M_a_n2o * B * 1e-6 * (H_n2o  * exp(2700 * (1 / (T_c + 273.15) - 1 / 298.15))))
S
```

```
## [1] 0.9851878
```

Uncertainty in the saturation ratio, S, is simulated below for both a standard GC and a high precision GC. The GC used for the current study is optimized to quantify several analytes across a broad range of concentrations but greater precision can be achieved by optimizing the instrument for a single analyte across a narrower range of concentrations. For example, laboratories participating in an international analysis of N2O standards used for atmsopheric monitoring reported a mean standard deviation of 7.3^{-4}, which is 10-fold lower than the standard deviation of N2O measurements in this study (0.0078725). Utilizing more precise N2O measurements for the equilibrated headspace and/or air will reduce uncertainty in the estimated dissolved N2O concentration, dissolved N2O equilibrium value, and N2O saturation ratio.

In addition to the two different GC setups, uncertainty in S is also simulated for under additional scenarios using combinations of ambient air vs a pure gas as the headspace gas along with a standard *vs.* high precision thermometer for field temperature (C) measurements.

All of these simulations use the mixing ratio used for the empirical field measurements, where $R_M = 0.25$.

4.1 Standard GC

4.1.1 Ambient air, standard thermometer

Simulate uncertainty in the saturation ratio due to measurement error associated with the standard GC, using ambient air as the headspace gas, and a standard field thermometer for temperature. This scenario represents the approach used for the empirical observations used in this study.

```
S_a_me <- rep( NA, nsim )

for(i in 1:nsim){
S_a_me[i] <- (1e-6 * B_me[i] * (V_g_me[i] * (M_h_me[i] - M_a_n2o_me[i]) / (8.31445
  (M_a_n2o_me[i] * B_me[i] * 1e-6 * (H_n2o  * exp(2700*(1/(T_c_me[i] + 273.15) - 1
}

# combine into dataframe for ggplot. qplot, which accepts vectors,
# was deprecated in ggplot 3.4.0
S_a_df <- tibble(S_a_me = S_a_me)
# save absolute error for plot
S_a_me_abs <- S_a_me - S
```

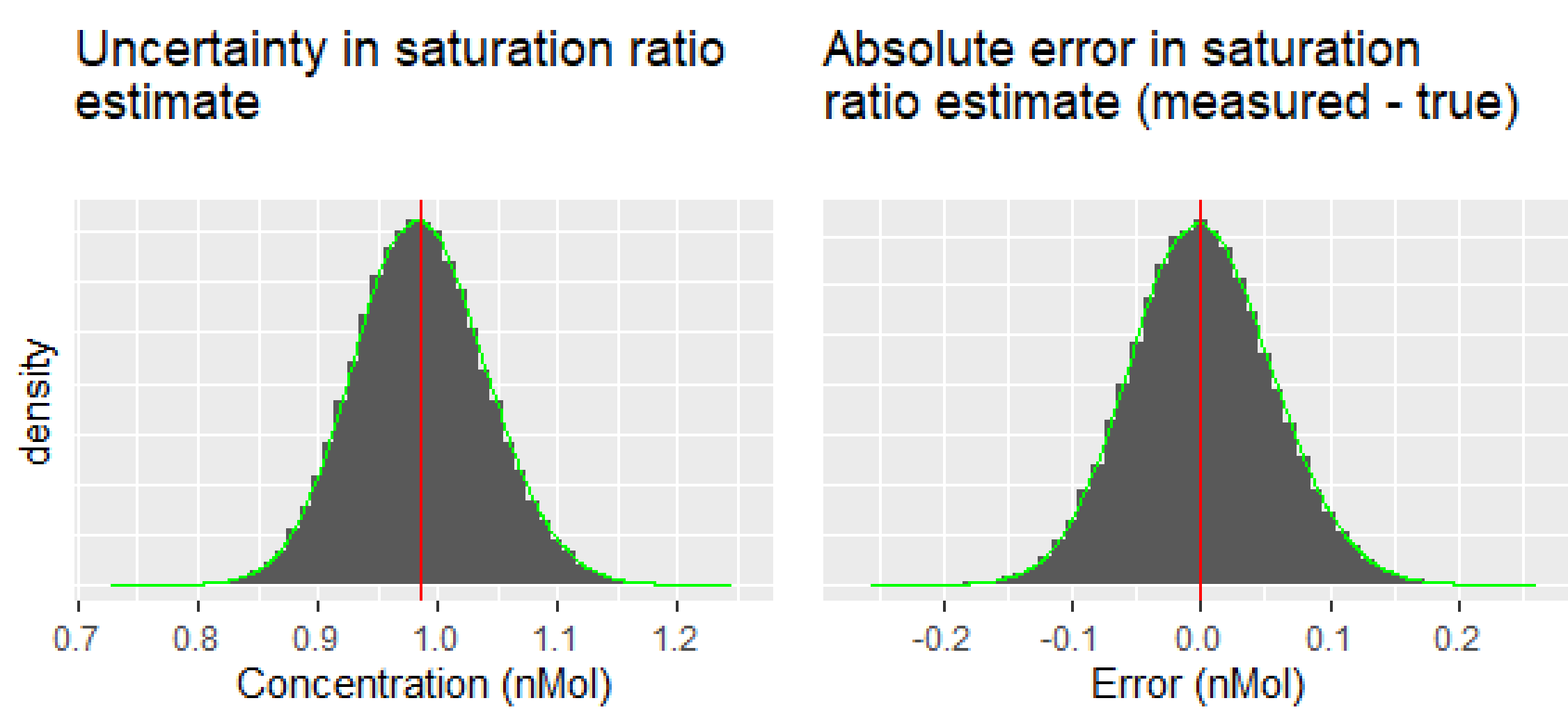


```
# standard deviation of a saturation ratio value s:
sd(S_a_me) # 0.026
```

```
## [1] 0.05487627
```

```
# the 95% CI is therefore the computed value +/- 2 standard deviations
sd(S_a_me) * 1.96 # +/- 0.05258214
```

```
## [1] 0.1075575
```

```
# so any ratio within 0.05 of 1.0 cannot be discerned as a source or sink?
```

```
# standard deviation of a measurement is:
round(sd(S_a_me_abs), 3) # 0.20 nmol L-1
```

```
## [1] 0.055
```

```
# central 95th percentile of absolute error
round(quantile(S_a_me_abs, probs = c(0.025, 0.975)), 3) # approx +/- 0.11
```

```
##   2.5%  97.5%
## -0.104  0.111
```

```
# the 95% CI for an observation with measurement error can be computed
# approximately as the value +/- 2 standard deviations; or the central 95th
# percentile of the absolute error distribution.
round(quantile((S + S_a_me_abs), probs = c(0.025, 0.975)), 3) # +/- nmol N2O
```

```
##  2.5% 97.5%
## 0.881 1.097
```

4.1.1.1 Map source-sink status

Below, the source-sink status of the empirical measurments used for this study are estimated, assuming the measurement errors estimated above. The proportion of observations assumed to be sinks or sources of N_2O is also estimated along with the estimate of the proportion of the lakes sampled for which the status was assumed to be

"undetermined" due to uncertainty arising from the estimated measurement error. A map of each of the sampled lakes' estimated status is also shown.

```
# two standard deviations = 0.3019449
# 95% CI is therefore x+/-0.3019449

#dg <- dg %>%
#  mutate(n2o.src.snk.with.all.error = case_when(
#    abs(n2o.sat.ratio - 1) < sd(C_me_ratio) ~ "undetermined",
#    TRUE ~ n2o.src.snk))
dg <- dg %>%
  mutate(n2o.src.snk.with.all.error = case_when(
    abs(n2o.sat.ratio - 1) < quantile(S_a_me_abs, probs = c(0.975)) ~ "undetermine
    TRUE ~ n2o.src.snk))

dg %>%
  filter(sample.source == "DG", visit.no == 1, sitetype == "PROB") %>%
  {table(.$n2o.src.snk.with.all.error)} %>%
  kable(digits = 2, caption = "Number of lakes classified as source/sink")
```

Var1	Freq
sink	377
source	183
undetermined	424

Number of lakes classified as source/sink

```
dg %>%
  filter(sample.source == "DG", visit.no == 1, sitetype == "PROB") %>%
  {prop.table(table(.$n2o.src.snk.with.all.error))} %>%
  kable(digits = 2, caption = "Proportion of lakes classified as source/sink")
```

Var1	Freq
sink	0.38
source	0.19
undetermined	0.43

Proportion of lakes classified as source/sink

```
# sf for plotting
dg.sf <- st_as_sf(dg, coords = c("map.lon.dd", "map.lat.dd"),
                  crs = 4269) %>% # standard for lat/lon
  st_transform(5070) # project to CONUS ALBERS for plotting
```

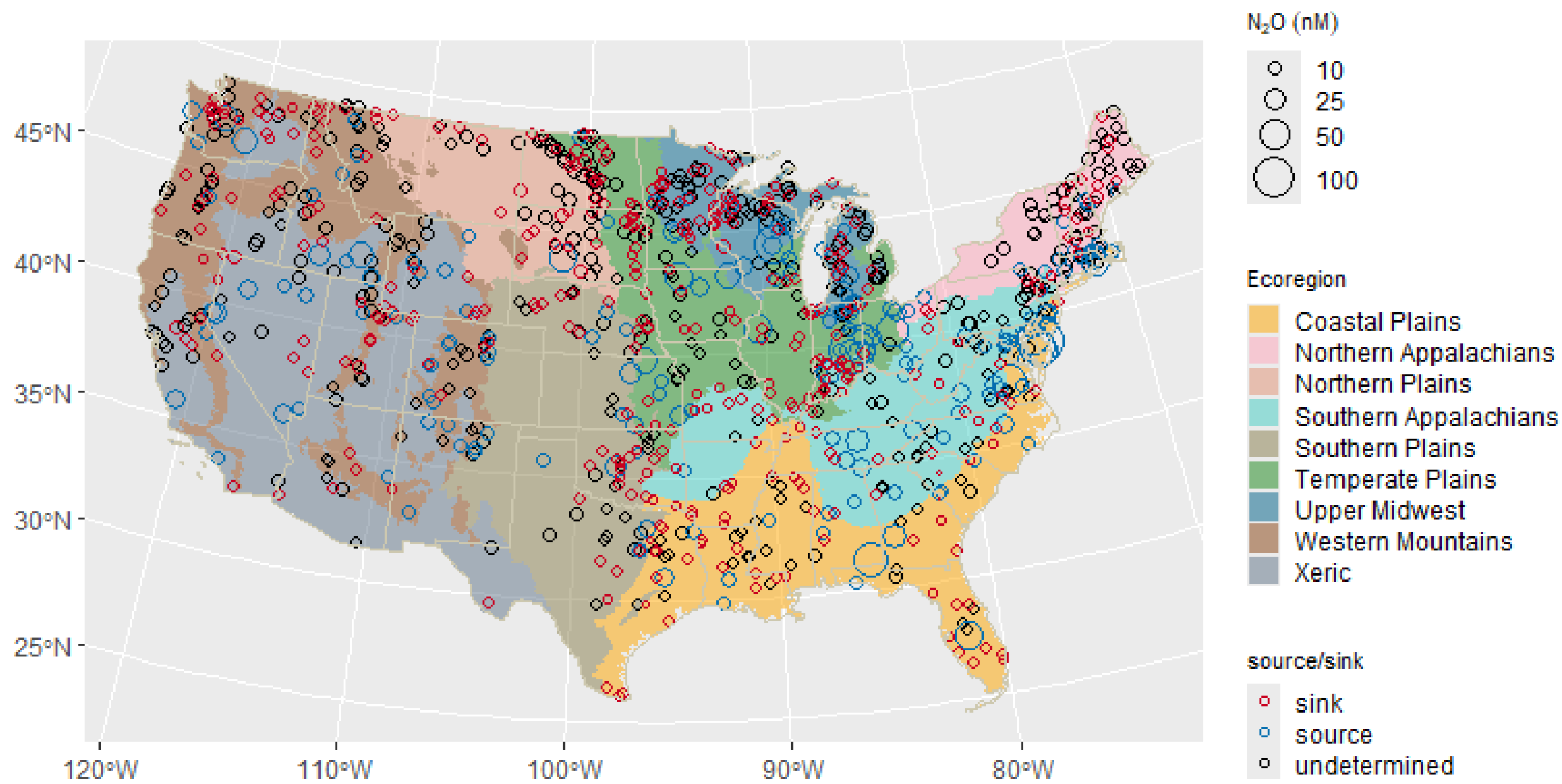

4.1.2 Pure gas, standard thermometer

Simulate uncertainty in the saturation ratio due to measurement error associated with the standard GC, using a pure gas (helium) as the headspace gas, and the standard field thermometer.

```
# set M_h to a lower value to generate a saturation ratio close to that used above
# M_h will be lower due to dilution with pure headspace gas (e.g. helium, nitrogen
M_h_pg <- 0.2

# this simulates measurement error on GC.
# assume TRUE value is M_h and error is simulated with with sd of replicate GC mea
M_h_pg_me <- rnorm(nsim, M_h_pg, n2o_sd_epa) # me in measured gas in headspace.
                                   # add random error to measured concentration

# under this scenario we don't need to subtract M_a_n2o (N2O concentration in air)
S_pg <- (1e-6 * B * (V_g * (M_h_pg - 0) / (8.3144598 * (T_c + 273.15) * V_w) + H_n
```

```
  (M_a_n2o * B * 1e-6 * (H_n2o  * exp(2700*(1/(T_c + 273.15) - 1/298.15))))

S_pg_me <- rep(NA, nsim)

for(i in 1:nsim){
S_pg_me[i] <- (1e-6 * B_me[i] * (V_g_me[i] * (M_h_pg_me[i] - 0) / (8.3144598 * (T_
  (M_a_n2o_me[i] * B_me[i] * 1e-6 * (H_n2o  * exp(2700*(1/(T_c_me[i] + 273.15) - 1
}

# combine into dataframe for ggplot. qplot, which accepts vectors,
# was deprecated in ggplot 3.4.0
S_pg_df <- tibble(S_pg_me = S_pg_me)
# save absolute error for plot
S_pg_me_abs <- S_pg_me - S_pg
```

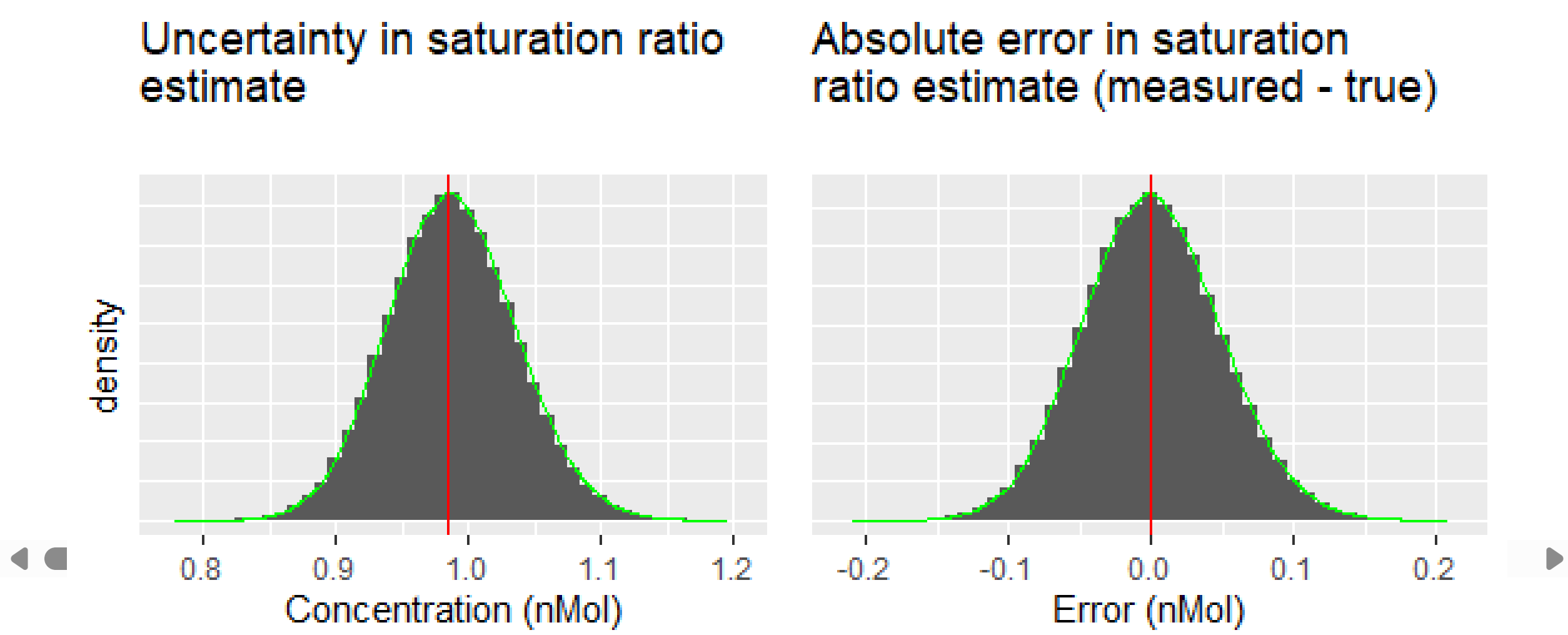


```
# standard deviation of a saturation ratio value s:
sd(S_pg_me) # 0.048
```

```
## [1] 0.0481663
```

```
# the 95% CI is therefore the computed value +/- 2 standard deviations
sd(S_pg_me) * 1.96 # +/- 0.094
```

```
## [1] 0.09440596
```

```
# so any ratio within 0.094 of 1.0 cannot be discerned as a source or sink
```

```
# standard deviation of a measurement is:
round(sd(S_pg_me_abs), 3) # 0.20 nmol L-1
```

```
## [1] 0.048
```

```
# central 95th percentile of absolute error
round(quantile(S_pg_me_abs, probs = c(0.025, 0.975)), 3) # approx +/- 0.11
```

```
##   2.5%  97.5%
## -0.091  0.097
```

```
# the 95% CI for an observation with measurement error can be computed
# approximately as the value +/- 2 standard deviations; or the central 95th
# percentile of the absolute error distribution.
round(quantile((S_pg + S_pg_me_abs), probs = c(0.025, 0.975)), 3) # +/- nmol N2O
```

```
##  2.5% 97.5%
## 0.896 1.085
```

4.1.2.1 Source-sink status

```
# two standard deviations = 0.3019449
# 95% CI is therefore x+/-0.3019449

#dg <- dg %>%
#  mutate(n2o.src.snk.with.all.error = case_when(
#    abs(n2o.sat.ratio - 1) < sd(C_me_ratio) ~ "undetermined",
#    TRUE ~ n2o.src.snk))
dg <- dg %>%
  mutate(n2o.src.snk.with.all.error = case_when(
    abs(n2o.sat.ratio - 1) < quantile(S_pg_me_abs, probs = c(0.975)) ~ "undetermin
    TRUE ~ n2o.src.snk))

dg %>%
  filter(sample.source == "DG", visit.no == 1, sitetype == "PROB") %>%
  {table(.$n2o.src.snk.with.all.error)} %>%
  kable(digits = 2, caption = "Number of lakes classified as source/sink")
```

Var1	Freq
sink	407
source	194
undetermined	383

Number of lakes classified as source/sink

```
dg %>%
  filter(sample.source == "DG", visit.no == 1, sitetype == "PROB") %>%
  {prop.table(table(.$n2o.src.snk.with.all.error))} %>%
  kable(digits = 2, caption = "Proportion of lakes classified as source/sink")
```

Var1	Freq
sink	0.41
source	0.20
undetermined	0.39

Proportion of lakes classified as source/sink

4.1.3 Ambient air, high precision thermometer

Simulate uncertainty in the saturation ratio due to measurement error associated with the standard GC and ambient air used as the headspace gas, and a high precision thermometer (e.g., MIMS) used for temperature measurements.

```
# High precision water batch used to control temperature of standards
T_c_mims_me <- rnorm(nsim, T_c, 0.005) # thermo stated bath precision is +/-0.01 C
# hist(T_c_mims_me) # sd of 0.005 gives approx distribution for the specified prec

T_c_df <- tibble(T_c_me = T_c_me,
                 T_c_me_abs = T_c_me - T_c,
                 T_c_mims_me = T_c_mims_me,
                 T_c_mims_me_abs = T_c_mims_me - T_c)
```

The precision gain from the MIMS thermometer relative to the standard field thermometer is roughly one order of magnitude.

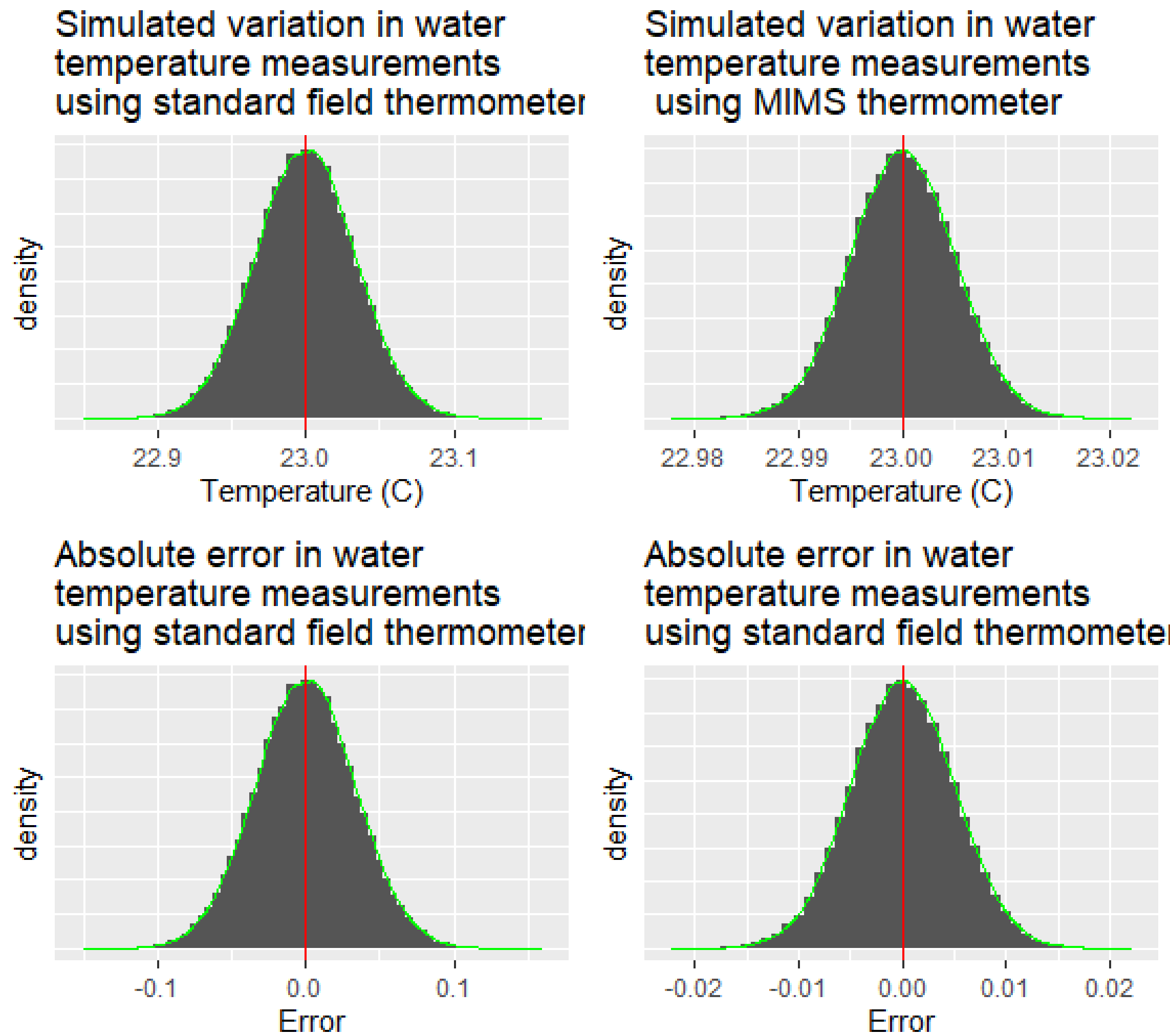

Simulate uncertainty in the saturation ratio when using ambient air in the headspace and high precision temperature measurements

```
S_a_hpt_me <- rep(NA, nsim)

for(i in 1:nsim){
S_a_hpt_me[i] <- (1e-6 * B_me[i] * (V_g_me[i] * (M_h_me[i] - M_a_n2o_me[i]) / (8.3
  (M_a_n2o_me[i] * B_me[i] * 1e-6 * (H_n2o  * exp(2700*(1/(T_c_mims_me[i] + 273.15
}

# combine into dataframe for ggplot. qplot, which accepts vectors,
# was deprecated in ggplot 3.4.0
S_a_hpt_df <- tibble(S_a_hpt_me = S_a_hpt_me)
# save absolute error for plot
S_a_hpt_me_abs <- S_a_hpt_me - S
```

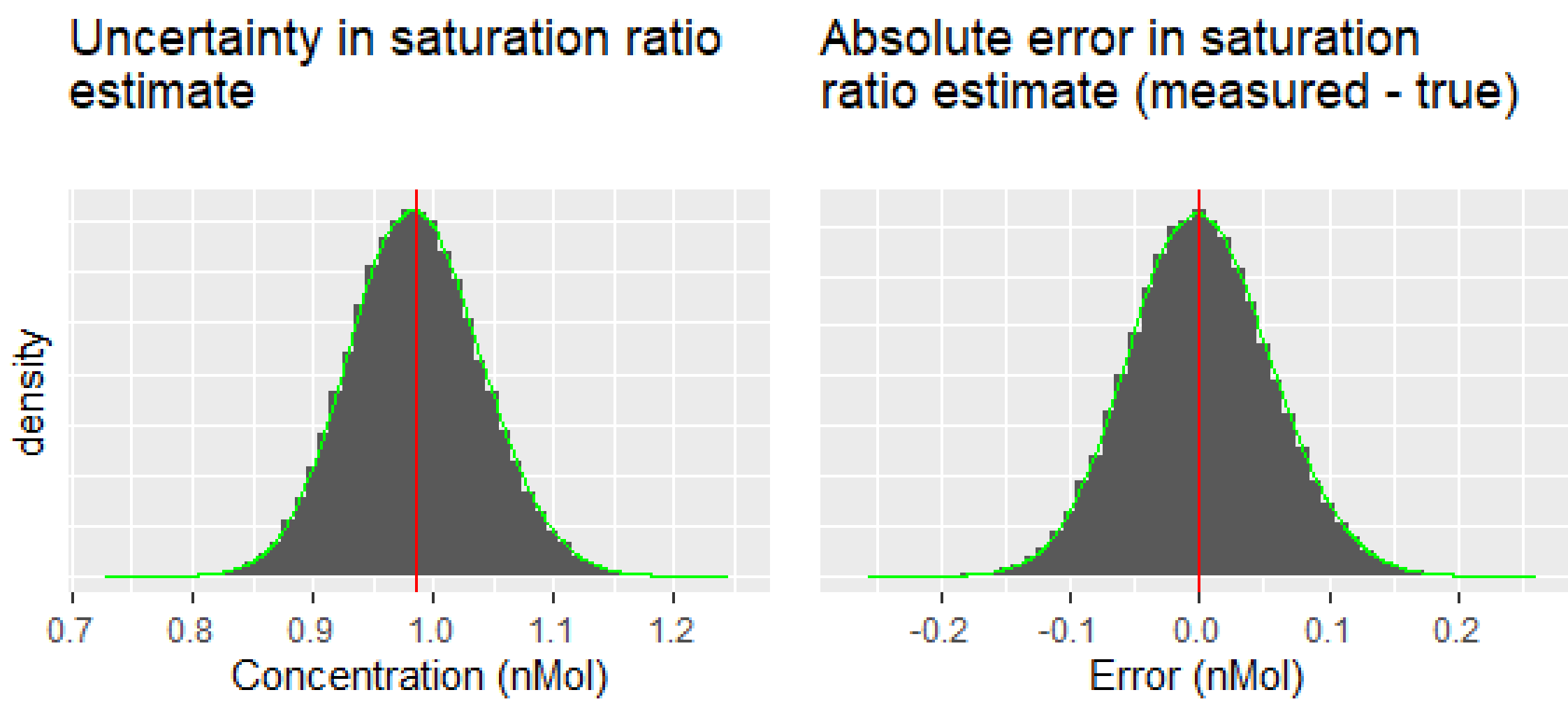


```
# standard deviation of a saturation ratio value s:
sd(S_a_hpt_me) # 0.026
```

```
## [1] 0.05487615
```

```
# the 95% CI is therefore the computed value +/- 2 standard deviations
sd(S_a_hpt_me) * 1.96 # +/- 0.05258214
```

```
## [1] 0.1075572
```

```
# so any ratio within 0.05 of 1.0 cannot be discerned as a source or sink?
```

```
# standard deviation of a measurement is:
round(sd(S_a_hpt_me_abs), 3) # 0.20 nmol L-1
```

```
## [1] 0.055
```

```
# central 95th percentile of absolute error
round(quantile(S_a_hpt_me_abs, probs = c(0.025, 0.975)), 3) # approx +/- 0.11
```

```
##   2.5%  97.5%
## -0.104  0.111
```

```
# the 95% CI for an observation with measurement error can be computed
# approximately as the value +/- 2 standard deviations; or the central 95th
# percentile of the absolute error distribution.
round(quantile((S + S_a_hpt_me_abs), probs = c(0.025, 0.975)), 3) # +/- nmol N2O
```

```
##  2.5% 97.5%
## 0.881 1.097
```

4.1.3.1 Source-sink status

```
# two standard deviations = 0.3019449
# 95% CI is therefore x+/-0.3019449

#dg <- dg %>%
#  mutate(n2o.src.snk.with.all.error = case_when(
#    abs(n2o.sat.ratio - 1) < sd(C_me_ratio) ~ "undetermined",
#    TRUE ~ n2o.src.snk))
dg <- dg %>%
  mutate(n2o.src.snk.with.all.error = case_when(
    abs(n2o.sat.ratio - 1) < quantile(S_a_hpt_me_abs, probs = c(0.975)) ~ "undeter
    TRUE ~ n2o.src.snk))

dg %>%
  filter(sample.source == "DG", visit.no == 1, sitetype == "PROB") %>%
  {table(.$n2o.src.snk.with.all.error)} %>%
  kable(digits = 2, caption = "Number of lakes classified as source/sink")
```

Var1	Freq
sink	377
source	183
undetermined	424

Number of lakes classified as source/sink

```
dg %>%
  filter(sample.source == "DG", visit.no == 1, sitetype == "PROB") %>%
```

```
{prop.table(table(.$n2o.src.snk.with.all.error))} %>%
kable(digits = 2, caption = "Proportion of lakes classified as source/sink")
```

Var1	Freq
sink	0.38
source	0.19
undetermined	0.43

Proportion of lakes classified as source/sink

4.1.4 Pure gas, high precision thermometer

Simulate uncertainty in the saturation ratio due to measurement error associated with the standard GC, a pure gas (helium) as the headspace gas, and using a high precision thermometer (e.g., MIMS).

```
S_pg_hpt_me <- rep(NA, nsim)

for(i in 1:nsim){
S_pg_hpt_me[i] <- (1e-6 * B_me[i] * (V_g_me[i] * (M_h_pg_me[i] - 0) / (8.3144598 *
  (M_a_n2o_me[i] * B_me[i] * 1e-6 * (H_n2o  * exp(2700*(1/(T_c_mims_me[i] + 273.15
}

# combine into dataframe for ggplot. qplot, which accepts vectors,
# was deprecated in ggplot 3.4.0
S_pg_hpt_df <- tibble(S_pg_hpt_me = S_pg_hpt_me)
# save absolute error for plot
S_pg_hpt_me_abs <- S_pg_hpt_me - S_pg
```

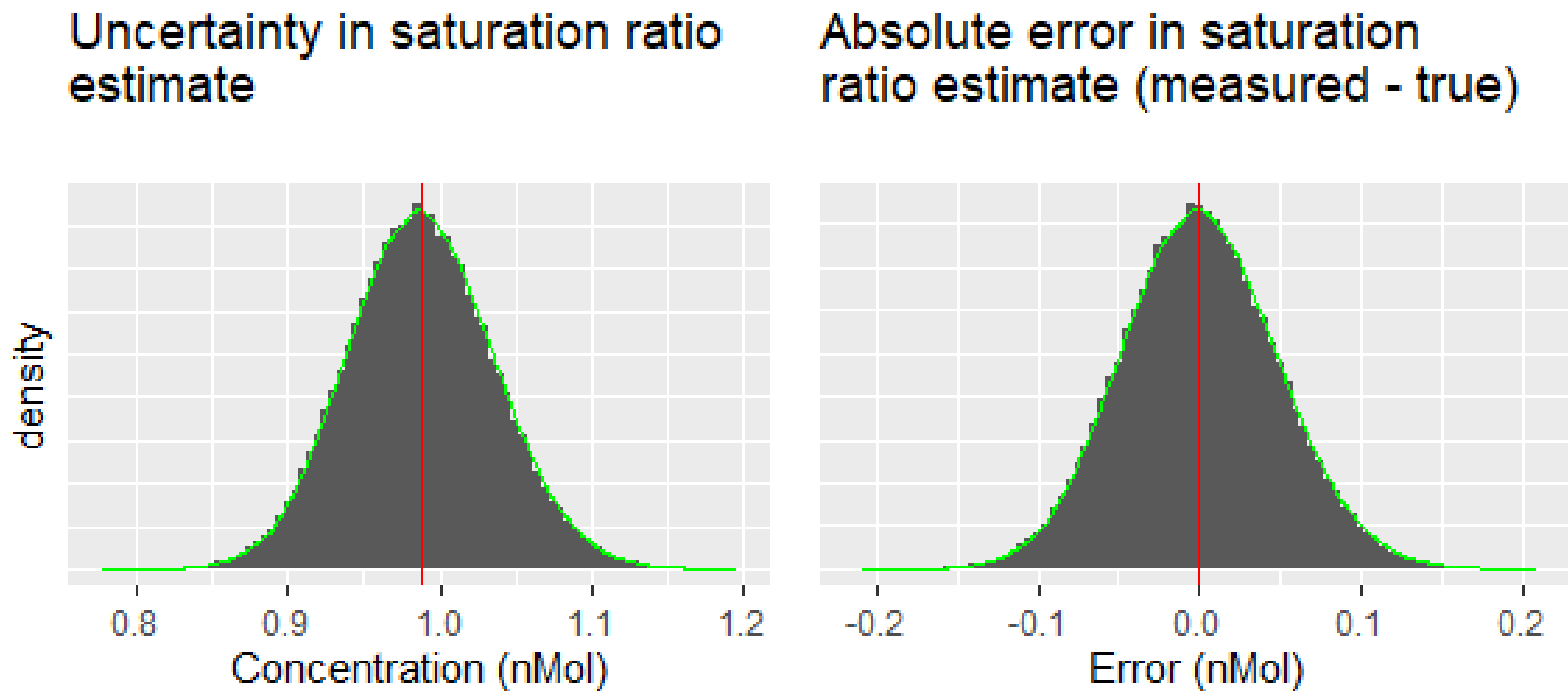


```
# standard deviation of a saturation ratio value s:
sd(S_pg_hpt_me) # 0.026
```

```
## [1] 0.04816535
```

```
# the 95% CI is therefore the computed value +/- 2 standard deviations
sd(S_pg_hpt_me) * 1.96 # +/- 0.05258214
```

```
## [1] 0.09440409
```

```
# so any ratio within 0.05 of 1.0 cannot be discerned as a source or sink?
```

```
# standard deviation of a measurement is:
round(sd(S_pg_hpt_me_abs), 3) # 0.20 nmol L-1
```

```
## [1] 0.048
```

```
# central 95th percentile of absolute error
round(quantile(S_pg_hpt_me_abs, probs = c(0.025, 0.975)), 3) # approx +/- 0.11
```

```
##   2.5%  97.5%
## -0.091  0.097
```

```
# the 95% CI for an observation with measurement error can be computed
# approximately as the value +/- 2 standard deviations; or the central 95th
# percentile of the absolute error distribution.
round(quantile((S_pg + S_pg_hpt_me_abs), probs = c(0.025, 0.975)), 3) # +/- nmol N
```

```
##  2.5% 97.5%
## 0.896 1.085
```

4.1.4.1 Source-sink status

```
# two standard deviations = 0.3019449
# 95% CI is therefore x+/-0.3019449

#dg <- dg %>%
#  mutate(n2o.src.snk.with.all.error = case_when(
#    abs(n2o.sat.ratio - 1) < sd(C_me_ratio) ~ "undetermined",
#    TRUE ~ n2o.src.snk))
dg <- dg %>%
  mutate(n2o.src.snk.with.all.error = case_when(
    abs(n2o.sat.ratio - 1) < quantile(S_pg_hpt_me_abs, probs = c(0.975)) ~ "undete
    TRUE ~ n2o.src.snk))

dg %>%
  filter(sample.source == "DG", visit.no == 1, sitetype == "PROB") %>%
  {table(.$n2o.src.snk.with.all.error)} %>%
  kable(digits = 2, caption = "Number of lakes classified as source/sink")
```

Var1	Freq
sink	407
source	194
undetermined	383

Number of lakes classified as source/sink

```
dg %>%
  filter(sample.source == "DG", visit.no == 1, sitetype == "PROB") %>%
```

```
{prop.table(table(.$n2o.src.snk.with.all.error))} %>%
kable(digits = 2, caption = "Proportion of lakes classified as source/sink")
```

Var1	Freq
sink	0.41
source	0.20
undetermined	0.39

Proportion of lakes classified as source/sink

4.2 High precision GC

The GC used in the current study is optimized to quantify several analytes across a broad range of concentrations but greater precision can be achieved by optimizing the instrument for a single analyte across a narrower range of concentrations. For example, laboratories participating in an international analysis of N2O standards used for atmsopheric monitoring reported a mean standard deviation of 7.3^{-4}, which is 10-fold lower than the standard deviation of N2O measurements in this study (0.0078725). Utilizing more precise N2O measurements for the equilibrated headspace and/or air will reduce uncertainty in the estimated dissolved N2O concentration, dissolved N2O equilibrium value, and N2O saturation ratio. Again, the simulations below assume the mixing ratio used in the empirical field observations, where $R_M = 0.25$.

4.2.1 Pure gas, standard thermometer

```
# set M_h to a lower value to generate a saturation ratio close to that used above
# M_h will be lower due to dilution with pure headspace gas (e.g. helium, nitrogen
M_h_pg_hpgc <- 0.2

# two standard deviations reported from each of three labs participating in
# a laboratory intercomparison study
n2o_sd_lit <- mean(c(.00033, .00034, .00031, 0.00030, 0.0016, 0.0015)) # from lite

# simulate improved measurement of N2O in equilibrated headspace gas
# assume TRUE value is M_h_pg_hpgc and error is simulated with with sd from litera
M_h_pg_hpgc_me <- rnorm(nsim, M_h_pg_hpgc, n2o_sd_lit)
```

```
# Set true value
# under this scenario we don't need to subtract M_a_n2o (N2O concentration in air)
S_pg_hpgc <- (1e-6 * B * (V_g * (M_h_pg_hpgc - 0) / (8.3144598 * (T_c + 273.15) * '
  (M_a_n2o * B * 1e-6 * (H_n2o  * exp(2700*(1/(T_c + 273.15) - 1/298.15))))

# Container for simulations
S_pg_hpgc_me <- rep(NA, 1e4)

for(i in 1:nsim){
S_pg_hpgc_me[i] <- (1e-6 * B_me[i] * (V_g_me[i] * (M_h_pg_hpgc_me[i] - 0) / (8.314
  (M_a_n2o_me[i] * B_me[i] * 1e-6 * (H_n2o  * exp(2700*(1 / (T_c_me[i] + 273.15) -
}

# combine into dataframe for ggplot. qplot, which accepts vectors,
# was deprecated in ggplot 3.4.0
S_pg_hpgc_df <- tibble(S_pg_hpgc_me = S_pg_hpgc_me)
# save absolute error for plot
S_pg_hpgc_me_abs <- S_pg_hpgc_me - S_pg_hpgc
```

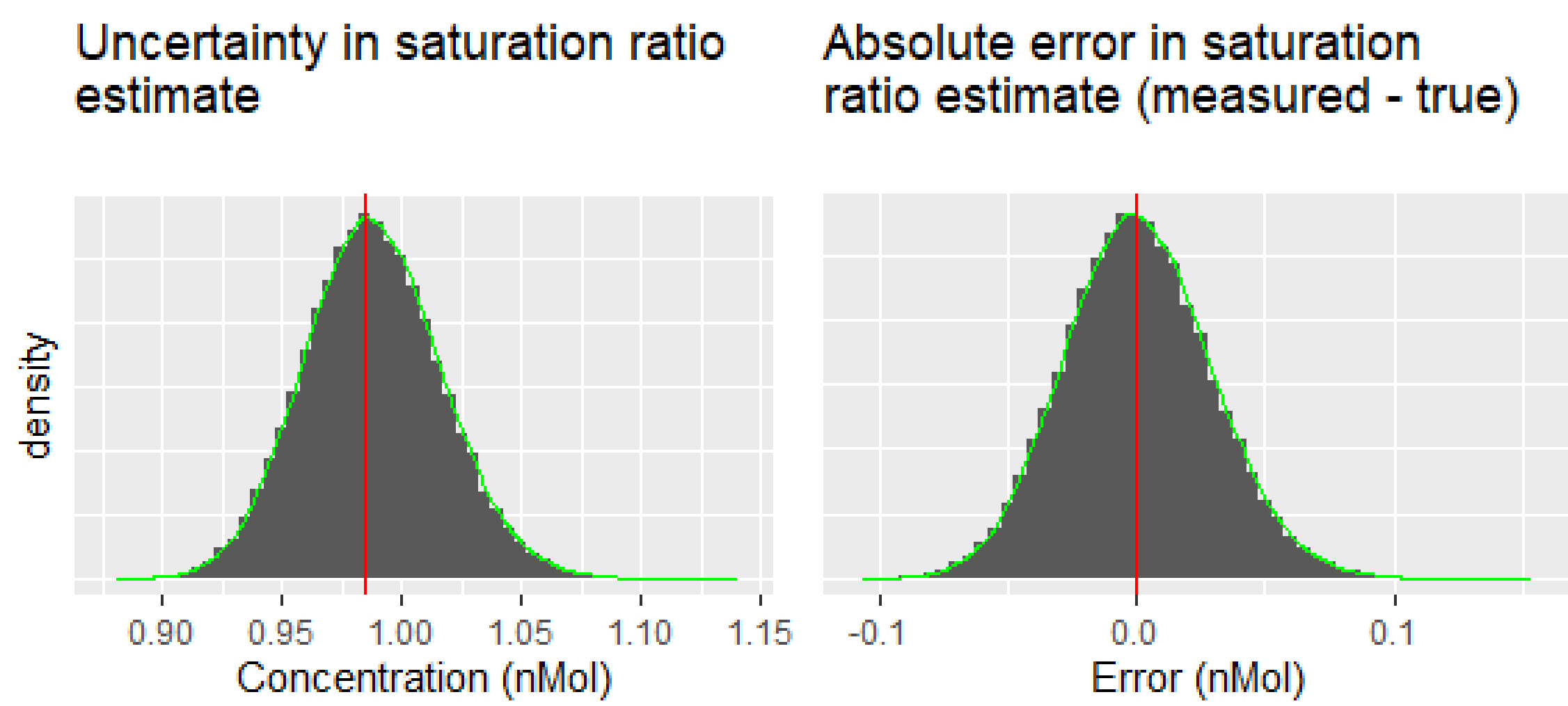


```
# standard deviation of a saturation ratio value s:
sd(S_pg_hpgc_me) # 0.029
```

```
## [1] 0.02850756
```

```
# the 95% CI is therefore the computed value +/- 2 standard deviations
sd(S_pg_hpgc_me) * 1.96 # +/- 0.0569
```

```
## [1] 0.05587482
```

```
# so any ratio within 0.0569 of 1.0 cannot be discerned as a source or sink
```

```
# standard deviation of a measurement is:
round(sd(S_pg_hpgc_me_abs), 3) # 0.20 nmol L-1
```

```
## [1] 0.029
```

```
# central 95th percentile of absolute error
round(quantile(S_pg_hpgc_me_abs, probs = c(0.025, 0.975)), 3) # approx +/- 0.11
```

```
##   2.5%  97.5%
## -0.054  0.058
```

```
# the 95% CI for an observation with measurement error can be computed
# approximately as the value +/- 2 standard deviations; or the central 95th
# percentile of the absolute error distribution.
round(quantile((S_pg_hpgc + S_pg_hpgc_me_abs), probs = c(0.025, 0.975)), 3) # +/-
```

```
##  2.5% 97.5%
## 0.934 1.046
```

4.2.1.1 Source-sink status

```
# two standard deviations = 0.3019449
# 95% CI is therefore x+/-0.3019449

#dg <- dg %>%
#  mutate(n2o.src.snk.with.all.error = case_when(
#    abs(n2o.sat.ratio - 1) < sd(C_me_ratio) ~ "undetermined",
#    TRUE ~ n2o.src.snk))
dg <- dg %>%
  mutate(n2o.src.snk.with.all.error = case_when(
    abs(n2o.sat.ratio - 1) < quantile(S_pg_hpgc_me_abs, probs = c(0.975)) ~ "undet
    TRUE ~ n2o.src.snk))

dg %>%
```

```
  filter(sample.source == "DG", visit.no == 1, sitetype == "PROB") %>%
  {table(.$n2o.src.snk.with.all.error)} %>%
  kable(digits = 2, caption = "Number of lakes classified as source/sink")
```

Var1	Freq
sink	521
source	248
undetermined	215

Number of lakes classified as source/sink

```
dg %>%
  filter(sample.source == "DG", visit.no == 1, sitetype == "PROB") %>%
  {prop.table(table(.$n2o.src.snk.with.all.error))} %>%
  kable(digits = 2, caption = "Proportion of lakes classified as source/sink")
```

Var1	Freq
sink	0.53
source	0.25
undetermined	0.22

Proportion of lakes classified as source/sink

4.2.2 Pure gas, high precision thermometer

```
# set M_h to a lower value to generate a saturation ratio close to that used above
# M_h will be lower due to dilution with pure headspace gas (e.g. helium, nitrogen
M_h_pg_hpgc_hpt <- 0.2

# two standard deviations reported from each of three labs participating in
# a laboratory intercomparison study
n2o_sd_lit <- mean(c(.00033, .00034, .00031, 0.00030, 0.0016, 0.0015)) # from lite

# simulate improved measurement of N2O in equilibrated headspace gas
# assume TRUE value is C_e and error is simulated with with sd from literature
M_h_pg_hpgc_hpt_me <- rnorm(nsim, M_h_pg_hpgc, n2o_sd_lit)
```

```
S_pg_hpgc_hpt_me <- rep(NA, 1e4)

for(i in 1:nsim){
S_pg_hpgc_hpt_me[i] <- (1e-6 * B_me[i] * (V_g_me[i] * (M_h_pg_hpgc_hpt_me[i] - 0)
  (M_a_n2o_me[i] * B_me[i] * 1e-6 * (H_n2o  * exp(2700*(1 / (T_c_me[i] + 273.15) -
}


# combine into dataframe for ggplot. qplot, which accepts vectors,
# was deprecated in ggplot 3.4.0
S_pg_hpgc_hpt_df <- tibble(S_pg_hpgc_hpt_me = S_pg_hpgc_hpt_me)
# save absolute error for plot
S_pg_hpgc_hpt_me_abs <- S_pg_hpgc_hpt_me - S_pg_hpgc
```

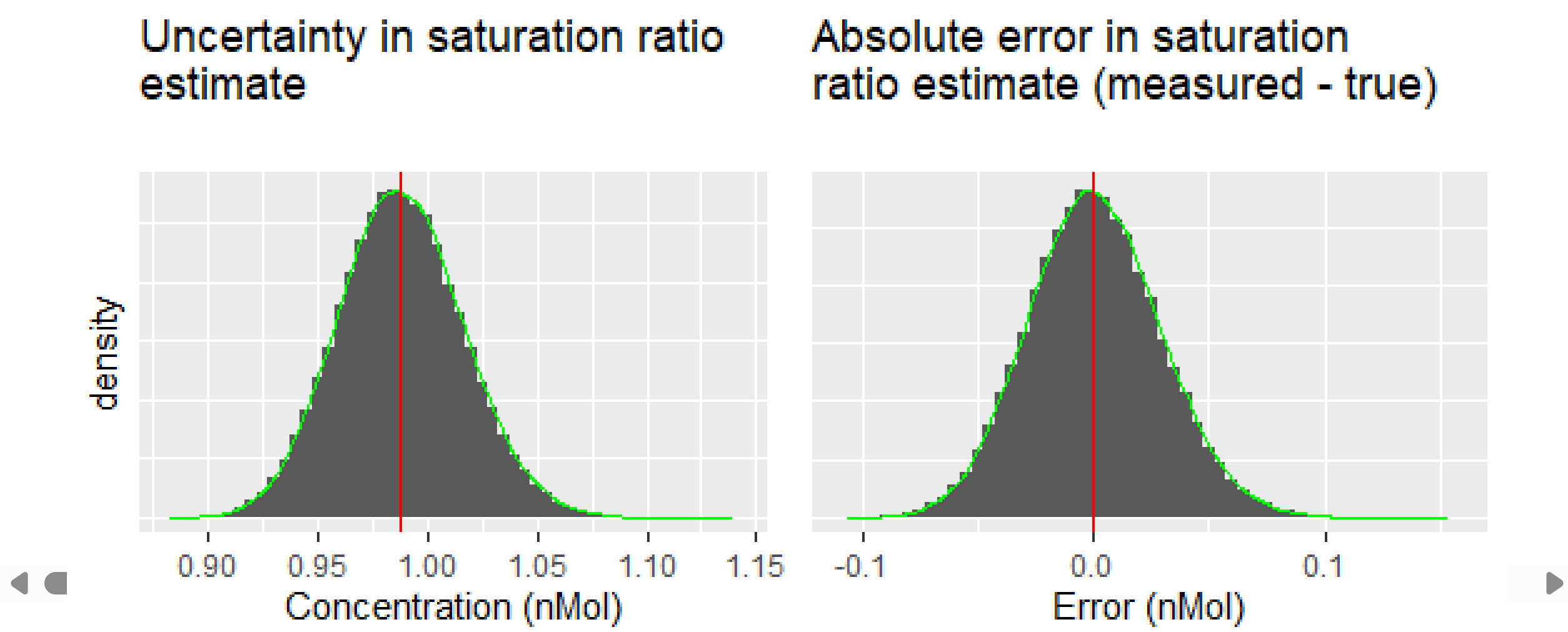


```
# standard deviation of a saturation ratio value s:
sd(S_pg_hpgc_hpt_me) # 0.029
```

```
## [1] 0.02849663
```

```
# the 95% CI is therefore the computed value +/- 2 standard deviations
sd(S_pg_hpgc_hpt_me) * 1.96 # +/- 0.0569
```

```
## [1] 0.05585339
```

```
# so any ratio within 0.0569 of 1.0 cannot be discerned as a source or sink
```

```
# standard deviation of a measurement is:
round(sd(S_pg_hpgc_hpt_me_abs), 3) # 0.20 nmol L-1
```

```
## [1] 0.028
```

```
# central 95th percentile of absolute error
round(quantile(S_pg_hpgc_hpt_me_abs, probs = c(0.025, 0.975)), 3) # approx +/- 0.1
```

```
##   2.5%  97.5%
## -0.053  0.059
```

```
# the 95% CI for an observation with measurement error can be computed
# approximately as the value +/- 2 standard deviations; or the central 95th
# percentile of the absolute error distribution.
round(quantile((S_pg_hpgc + S_pg_hpgc_hpt_me_abs), probs = c(0.025, 0.975)), 3) #
```

```
##  2.5% 97.5%
## 0.934 1.046
```

4.2.2.1 Source-sink status

```
# two standard deviations = 0.3019449
# 95% CI is therefore x+/-0.3019449

#dg <- dg %>%
#  mutate(n2o.src.snk.with.all.error = case_when(
#    abs(n2o.sat.ratio - 1) < sd(C_me_ratio) ~ "undetermined",
#    TRUE ~ n2o.src.snk))
dg <- dg %>%
  mutate(n2o.src.snk.with.all.error = case_when(
    abs(n2o.sat.ratio - 1) < quantile(S_pg_hpgc_hpt_me_abs, probs = c(0.975)) ~ "u
    TRUE ~ n2o.src.snk))

dg %>%
  filter(sample.source == "DG", visit.no == 1, sitetype == "PROB") %>%
```

```
  {table(.$n2o.src.snk.with.all.error)} %>%
  kable(digits = 2, caption = "Number of lakes classified as source/sink")
```

Var1	Freq
sink	521
source	247
undetermined	216

Number of lakes classified as source/sink

```
dg %>%
  filter(sample.source == "DG", visit.no == 1, sitetype == "PROB") %>%
  {prop.table(table(.$n2o.src.snk.with.all.error))} %>%
  kable(digits = 2, caption = "Proportion of lakes classified as source/sink")
```

Var1	Freq
sink	0.53
source	0.25
undetermined	0.22

Proportion of lakes classified as source/sink

5 MIMS

In Membrane Inlet Mass Spectrometry (MIMS), dissolved gases (typically N2, Ar, O2, N2O) are extracted from a water sample as it passes through a semi-permeable membrane. The abundance of the extracted gases are then measured using a quadrapole mass spectrometer. One advantage of this approach relative to the traditional headspace equilibrium method is that dissolved gases are extracted without contamination and/or dilution from a headspace gas (e.g. air, nitrogen, helium). Dilution by a pure gas isn't much of a concern because N2O detection limits are very low via GC, but correction for the presence of N2O in the headspace gas reduces measurement precision.

Dissolved N2O concentration in a MIMS sample can be calculated from the N2O signal strength and a calibration factor. The calibration factor is determined by analyzing a water sample with known N2O concentration. This is most frequently accomplished by equilibrating laboratory water with air at constant temperature, barometric pressure and N2O partial pressure. The dissolved N2O concentration of the equilibrated water is calculated from solubility laws (see Equilibrium concentration) and the calibration factor, f, is:

$$f_{N_2O} = \frac{C_s^{N_2O}}{X_s^{N_2O}}$$

where:

- $f_{N_2O} = N_2O \text{ calibration factor}$
- $C_s^{N_2O} = \text{Concentration of } N_2O \text{ in calibration standard}$
- $X_s^{N_2O} = \text{Signal strength of } N_2O \text{ in calibration standard}$

The N2O concentration of an unknown sample is then calculated as:

$$C_\theta^{N_2O} = \frac{X^{N_2O}}{f_{N_2O}}$$

where:

- $C_\theta^{N_2O} = N_2O \text{ concentration in sample}$
- $X_\theta^{N_2O} = \text{Signal strength of } N_2O \text{ in sample}$
- $f_{N_2O} = N_2O \text{ calibration factor}$

The equation simply scales the concentration of the N2O standard to the ratio of the MIMS signal strength of the unknown and standard. Via substitution and rearrangement, the equation can also be written as:

$$C_\theta^{N_2O} = \frac{X_\theta^{N_2O}}{X_s^{N_2O}} \cdot C_s^{N_2O}$$

MIMS provides greater precision when measuring gas ratios as compared to measuring a single gas. High precision gas ratio data can be converted to concentration data with the independent measurement of concentration of one of the component gases. N2O:Ar ratios are particularly useful for environmental samples because Ar is biologically inert,

therefore its concentration can be calculated from solubility, water temperature, barometric pressure, and atmospheric partial pressure (essentially constant across the globe). As with single gas measurements, N2O:Ar ratios are converted to N2O concentration using a calibration factor derived from measurement of laboratory water equilibrated with air:

$$f'_{N_2O} = \frac{R_s^C}{R_s^X}$$
$$R_s^C = \frac{C_s^{N_2O}}{C_s^{Ar}}$$
$$R_s^X = \frac{X_s^{N_2O}}{X_s^{Ar}}$$

where:

- $f'_{N_2O} = N_2O \text{ (ratio) calibration factor}$
- $R_s^C = \text{ Concetration ratio of } N_2O \text{ to Ar in calibration standard}$
- $R_s^X = \text{ Signal ratio of } N_2O \text{ to Ar in calibration standard}$

The N2O concentration of an unknown sample is then calculated as:

$$C_\theta^{N_2O} = R_s^X \cdot f'_{N_2O} \cdot C_s^{Ar}$$

where:

- $C_\theta^{N_2O} = \text{ Concentration of } N_2O \text{ in the sample}$
- $C_s^{Ar} = \text{ Concentration of Ar in the calibration standard}$

As before, the equation above scales the N2O:Ar ratio measured in the sample to that of the standard. Via substitution and rearrangement, the equation can be written as:

$$C_\theta^{N_2O} = \frac{R_\theta^X}{R_s^X} \cdot R_s^C \cdot C_s^{Ar}$$

where:

- $R_\theta^X = \text{ Signal ratio of } N_2O \text{ to Ar in the sample}$

Concentration of N2O and Ar in the standard, $C_s^{N_2O}$ is calculated from temperature of the equilibrated water, Tc, laboratory barometric pressure, B, and N2O and Ar partial pressure in the atmosphere, $C_a^{N_2O}$, C_a^{Ar}, respectively (see Equilibrium concentration). By substitution, the equation to estimate the concentration of N2O in the unknown sample becomes:

$$C_\theta^{N_2O} = \frac{R_\theta^X}{R_s^X} \times \frac{1^{-6} \cdot B \cdot C_a^{N_2O} \cdot (H_{N_2O} \cdot e^{2700 \cdot \frac{1}{Tc} - \frac{1}{298.15}})}{1^{-6} \cdot B \cdot C_a^{Ar} \cdot (H_{Ar} \cdot e^{1500 \cdot \frac{1}{Tc} - \frac{1}{298.15}})} \times 1^{-6} B \cdot C_a^{Ar} \cdot (H_{Ar} \cdot e^{1500 \cdot \frac{1}{Tc} - \frac{1}{298.15}})$$

where (repeating some variables from above for clarity):

- $C_\theta^{N_2O} =$ concentration of N_2O in the sample
- $R_\theta^X =$ Signal ratio of N_2O to Ar in the sample
- $R_s^X =$ Signal ratio of N_2O to Ar in calibration standard
- $B =$ barometric pressure (kPa)
- $Tc =$ temperature
- $C_a^{N_2O} =$ concentration of N_2O in the atmosphere
- $H_{N_2O} =$ Henry's law constant for N_2O
- $C_a^{Ar} =$ concentration of Ar in the atmosphere
- $H_{Ar} =$ Henry's law constant for Ar
- $Tc =$ Temperature of the equilibrated water

N2O concentration in laboratory air, ($C_a^{N_2O}$), is derived from direct measurement of N2O via gas chromatography and has a standard deviation of 0.0078725 using the method employed in this study (see "Empirical variation" under Dissolved concentration) although more precise methods are available (see $$Dissolved N_2O: high precision GC$). $Ar in the lab atmosphere (C^{Ar}_a) is 9340ppm and is assumed to be constant and perfectly known (e.g. zero error). Genther et al (2013) report a standard deviation of 0.022 for the N2O:Ar signal strength ratio but Speir et al (2023) report a CV of 0.05% (sd ~ 0.000096). Here we provisionally use the Speir value pending confirmation. MIMS uses a very precise thermometer (+/-0.01 C) to control the temperature of the water used as the analytical standard (simulated below). Measurement error in B was quantified above.

5.1 Dissolved concentration

5.1.1 Standard GC

```
#n2o_ar_standard_signal <- 0.27 # Speir et al. 0.27 pers comm. Genther et al. sect
#n2o_ar_sample_signal <- 0.23 # just a guess
#C_a_ar <- 9340 # ppm.  invariant
#T_c_mims <- T_c # set up for simulation of MIMS temperature bath (from GC simulat

R_X_s <- 0.27 # Speir et al. 0.27 pers comm. Genther et al. section 3.4 reports 0.
R_X_theta <- 0.23 # just a guess (signal ratio of N2O:Ar in sample)
C_Ar_a <- 9340 # ppm.  invariant (Concentration of Ar in atmospher)
# T_c_mims <- T_c # set up for simulation of MIMS temperature bath (from GC simula


# this simulates MIMS measurement.
# assume TRUE value is n2o_ar_standard_signal and error is simulated with CV from
# Speier et al reports "...our precision was good among all sample and standard re
# Using that CV to model measurement error in the N2O:Ar ratio yields a sd of diss
# the sd when measured via GC, which is weird.  Speier reported that the CV is for
# Genther et al reports a n2o:Ar sd of 0.022, but this also generated a sd for dis
# measured with GC!  Here we use the Speir data
R_X_s_sd <- 0.0005 * R_X_s # sd = CV * mean. 0.05% = 0.0005
R_X_theta_sd <- 0.0005 * R_X_theta # sd = CV * mean. 0.05% = 0.0005

R_X_s_me <- rnorm(nsim, R_X_s, R_X_s_sd) # me in N2O:Ar of standard
R_X_theta_me <- rnorm(nsim, R_X_theta, R_X_theta_sd) # me in N2O:Ar of sample


# High precision water batch used to control temperature of standards
T_c_mims_me <- rnorm(nsim, T_c, 0.005) # thermo stated bath precision is +/-0.01 C
# hist(T_c_mims_me) # sd of 0.005 gives approx distribution for the specified prec

# True dissolved N2O concentration
C_mims <- ((R_X_theta / R_X_s) * # N2O:Ar ratios for sample and standard
  ((1e-6 * B * M_a_n2o * (H_n2o * exp( 2700 * (1 / (T_c + 273.15) - 1 / 298.15))))
  (1e-6 * B * C_Ar_a * (H_ar * exp( 1500 * (1 / (T_c + 273.15) - 1 / 298.15))))) *
  (1e-6 * B * C_Ar_a * (H_ar  * exp(1500 *(1/(T_c + 273.15) - 1/298.15)))))

C_mims <- C_mims * 1e9 #convert mol to nmol

# variation in measured N2O concentration
C_mims_hpt_me <- rep(NA, nsim)

for(i in 1:nsim){
```

```
C_mims_hpt_me[i] <- (R_X_theta_me[i] / R_X_s_me[i]) * # N2O:Ar ratios for sample a
  ((1e-6 * B_me[i] * M_a_n2o_me[i] * (H_n2o * exp(2700 * (1 / (T_c_mims_me[i] + 27
  (1e-6 * B_me[i] * C_Ar_a * (H_ar * exp(1500 * (1 / (T_c_mims_me[i] + 273.15) - 1
  (1e-6 * B_me[i] * C_Ar_a * (H_ar * exp(1500 * (1 / (T_c_mims_me[i] + 273.15) - 1
}


# combine into dataframe for ggplot.
C_mims_hpt_df <- tibble(C_mims_hpt_me = C_mims_hpt_me * 1e9)
# save absolute error for plot
C_mims_hpt_me_abs <- (C_mims_hpt_me * 1e9) - C_mims
```

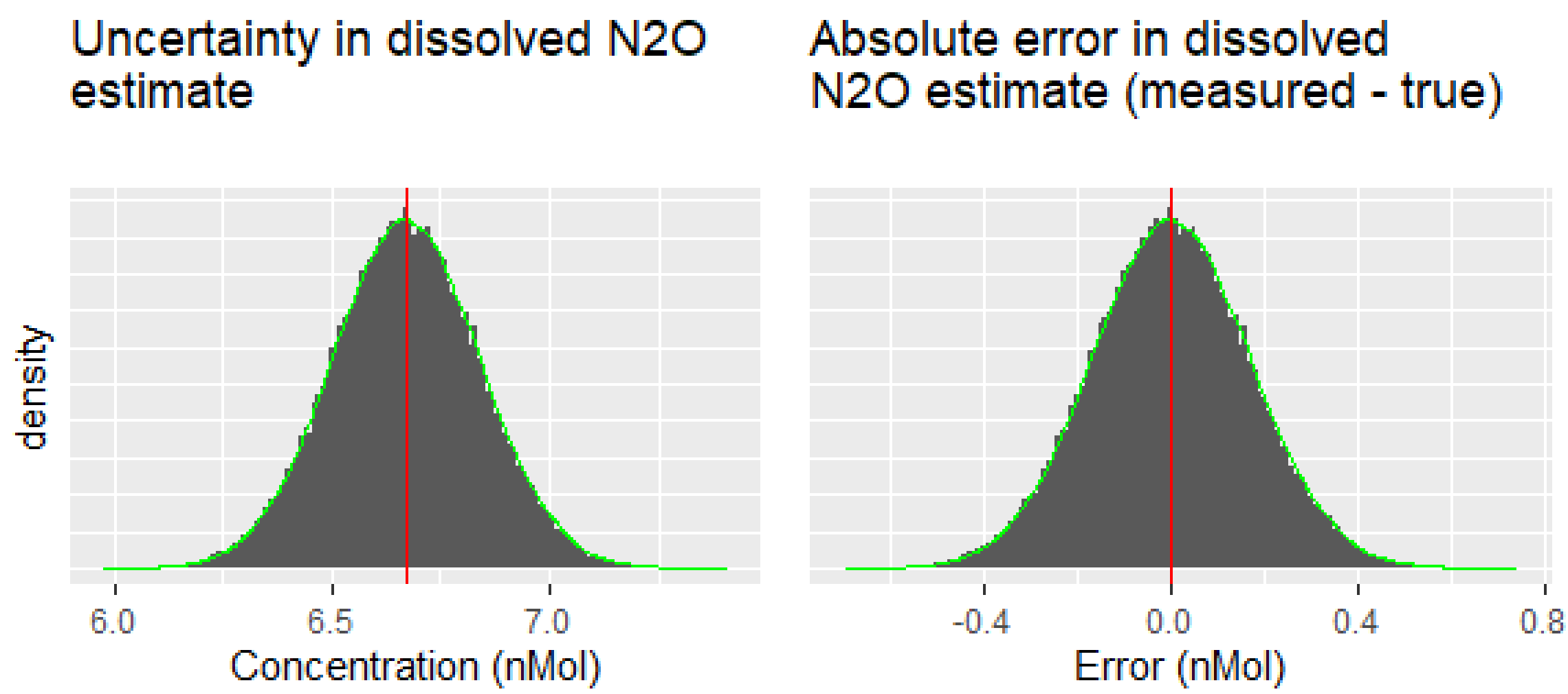


```
# standard deviation of dissolved N2O via MIMS:
sd(C_mims_hpt_me * 1e9) # 0.169 using Speir CV to calculate N2O:Ar sd.   0.15 with
```

```
## [1] 0.1688075
```

```
# 1.96 x sd
sd(C_mims_hpt_me * 1e9) * 1.96
```

```
## [1] 0.3308626
```

```
# CV of dissolved N2O
(sd(C_mims_hpt_me * 1e9) / mean(C_mims_hpt_me * 1e9)) * 100 # 0.025 = 2.5% vs 12%
```

```
## [1] 2.530701
```

```
# standard deviation of a measurement is:
round(sd(C_mims_hpt_me_abs), 3) # 0.20 nmol L-1
```

```
## [1] 0.169
```

```
# central 95th percentile of absolute error
round(quantile(C_mims_hpt_me_abs, probs = c(0.025, 0.975)), 3) # approx +/- 0.11
```

```
##   2.5%  97.5%
## -0.330  0.333
```

```
# the 95% CI for an observation with measurement error can be computed
# approximately as the value +/- 2 standard deviations; or the central 95th
# percentile of the absolute error distribution.
round(quantile((C_mims + C_mims_hpt_me_abs), probs = c(0.025, 0.975)), 3) # +/- nm
```

```
##  2.5% 97.5%
## 6.340 7.003
```

5.1.2 High precision GC

```
# simulate improved measurement precision of GC measurement of N2O in air
M_a_n2o_lit_me <- rnorm(nsim, M_a_n2o, n2o_sd_lit) # from intn'l labs = 0.00073 vs
```

```
# variation in measured N2O concentration
C_mims_hpgc_hpt_me <- rep(NA, nsim)

for(i in 1:nsim){
C_mims_hpgc_hpt_me[i] <- (R_X_theta_me[i] / R_X_s_me[i]) * # N2O:Ar ratios for sam
  ((1e-6 * B_me[i] * M_a_n2o_lit_me[i] * (H_n2o * exp(2700 * (1 / (T_c_mims_me[i]
  (1e-6 * B_me[i] * C_Ar_a * (H_ar * exp(1500 * (1 / (T_c_mims_me[i] + 273.15) - 1
```

```
  (1e-6 * B_me[i] * C_Ar_a * (H_ar  * exp(1500 * (1 / (T_c_mims_me[i] + 273.15) -
}


# combine into dataframe for ggplot.
C_mims_hpgc_hpt_df <- tibble(C_mims_hpgc_hpt_me = C_mims_hpgc_hpt_me * 1e9)
# save absolute error for plot
C_mims_hpgc_hpt_me_abs <- (C_mims_hpgc_hpt_me * 1e9) - C_mims
```

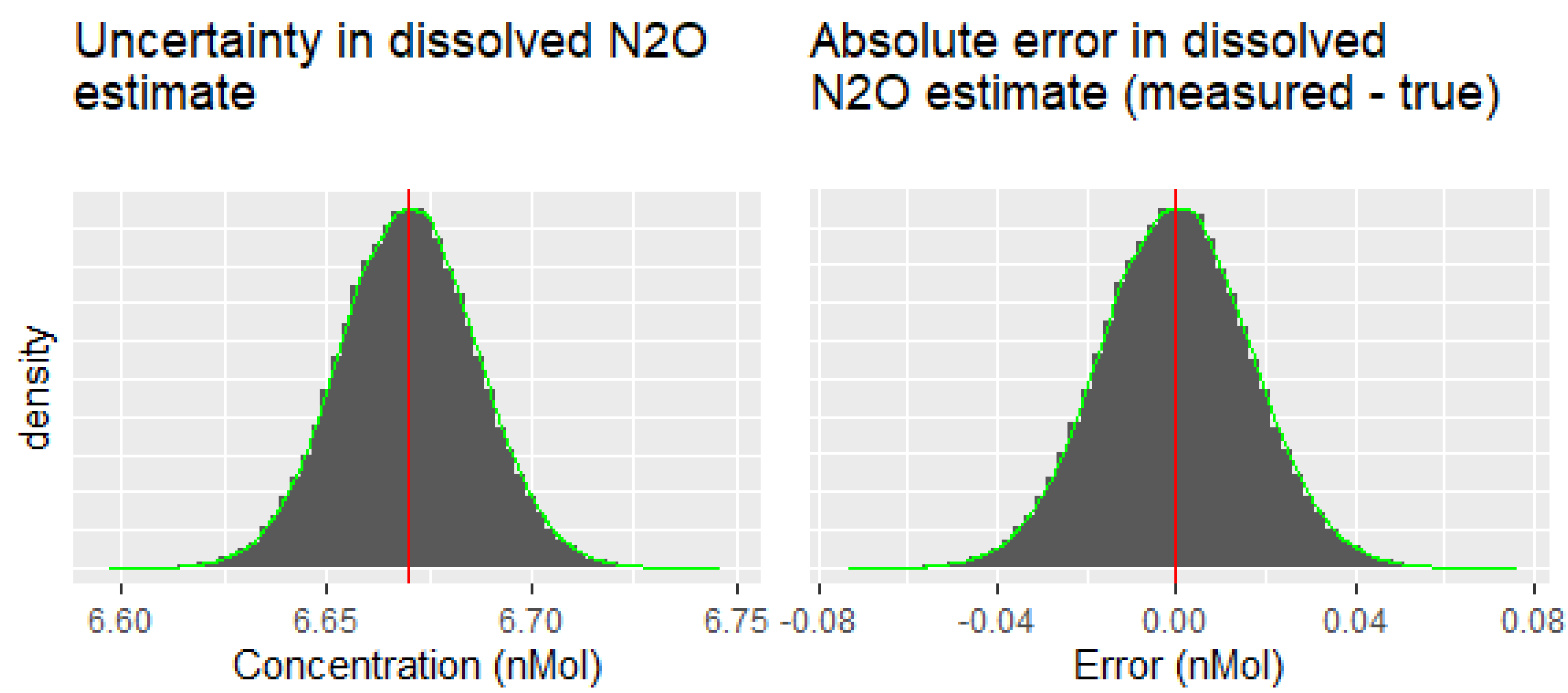


```
# standard deviation of dissolved N2O via MIMS:
sd(C_mims_hpgc_hpt_me * 1e9) # 0.0166 using Speir CV to calculate N2O:Ar sd. 0.15
```

```
## [1] 0.01658186
```

```
sd(C_mims_hpgc_hpt_me * 1e9) * 1.96 # sd +/- 1.96 (for conf intervals on error)
```

```
## [1] 0.03250045
```

```
# CV of dissolved N2O
sd(C_mims_hpgc_hpt_me * 1e9) / mean(C_mims_hpgc_hpt_me * 1e9) * 100 # 0.0025 = 0.2
```

```
## [1] 0.2486012
```

```
# standard deviation of a measurement is:
round(sd(C_mims_hpgc_hpt_me_abs), 3)
```

```
## [1] 0.017
```

```
# central 95th percentile of absolute error
round(quantile(C_mims_hpgc_hpt_me_abs, probs = c(0.025, 0.975)), 3)
```

```
##   2.5%  97.5%
## -0.032  0.033
```

```
# the 95% CI for an observation with measurement error can be computed
# approximately as the value +/- 2 standard deviations; or the central 95th
# percentile of the absolute error distribution.
round(quantile((C_mims + C_mims_hpgc_hpt_me_abs), probs = c(0.025, 0.975)), 3) # +
```

```
##  2.5% 97.5%
## 6.638 6.703
```

5.2 Saturation ratio

Calculate the “true” ratio via MIMS

```
# True ratio
S_mims <- ((R_X_theta / R_X_s) * # N2O:Ar ratios for sample and standard
  ( (1e-6 * B * M_a_n2o * (H_n2o * exp(2700 * (1 / (T_c + 273.15) - 1 / 298.15))))
  (1e-6 * B * C_Ar_a * (H_ar * exp(1500 * (1 / (T_c + 273.15) - 1 / 298.15)) ))) *
  (1e-6 * B * C_Ar_a *(H_ar  * exp(1500 * (1 / (T_c + 273.15) - 1 / 298.15))))) /
  (M_a_n2o * B * 1e-6 * (H_n2o  * exp(2700 * (1 / (T_c + 273.15) - 1 / 298.15))))
```

5.2.1 Standard GC

5.2.1.1 Standard field temperature

This simulation is for field measurment of temperature for the equilibrium concentration with the standard glass thermometer.

```
# ratio simulated with measurement error in mims
S_mims_me <- rep(NA, nsim)

for( i in 1:nsim){
S_mims_me[i] <- (R_X_theta_me[i] / R_X_s_me[i]) * # N2O:Ar ratios for sample and s
  ((1e-6 * B_me[i] * M_a_n2o_me[i] * (H_n2o * exp(2700 * (1 / (T_c_mims_me[i] + 27
  (1e-6 * B_me[i] * C_Ar_a * (H_ar * exp(1500 * (1 / (T_c_mims_me[i] + 273.15) - 1
  (1e-6 * B_me[i] * C_Ar_a * (H_ar  * exp(1500 *(1/(T_c_mims_me[i] + 273.15) - 1/2
  (M_a_n2o_me[i] * B_me[i] * 1e-6 * (H_n2o  * exp(2700 * (1 / (T_c_me[i] + 273.15)
}


# combine into dataframe for ggplot.
S_mims_df <- tibble(S_mims_me = S_mims_me)
# save absolute error for plot
S_mims_me_abs <- S_mims_me - S_mims
```

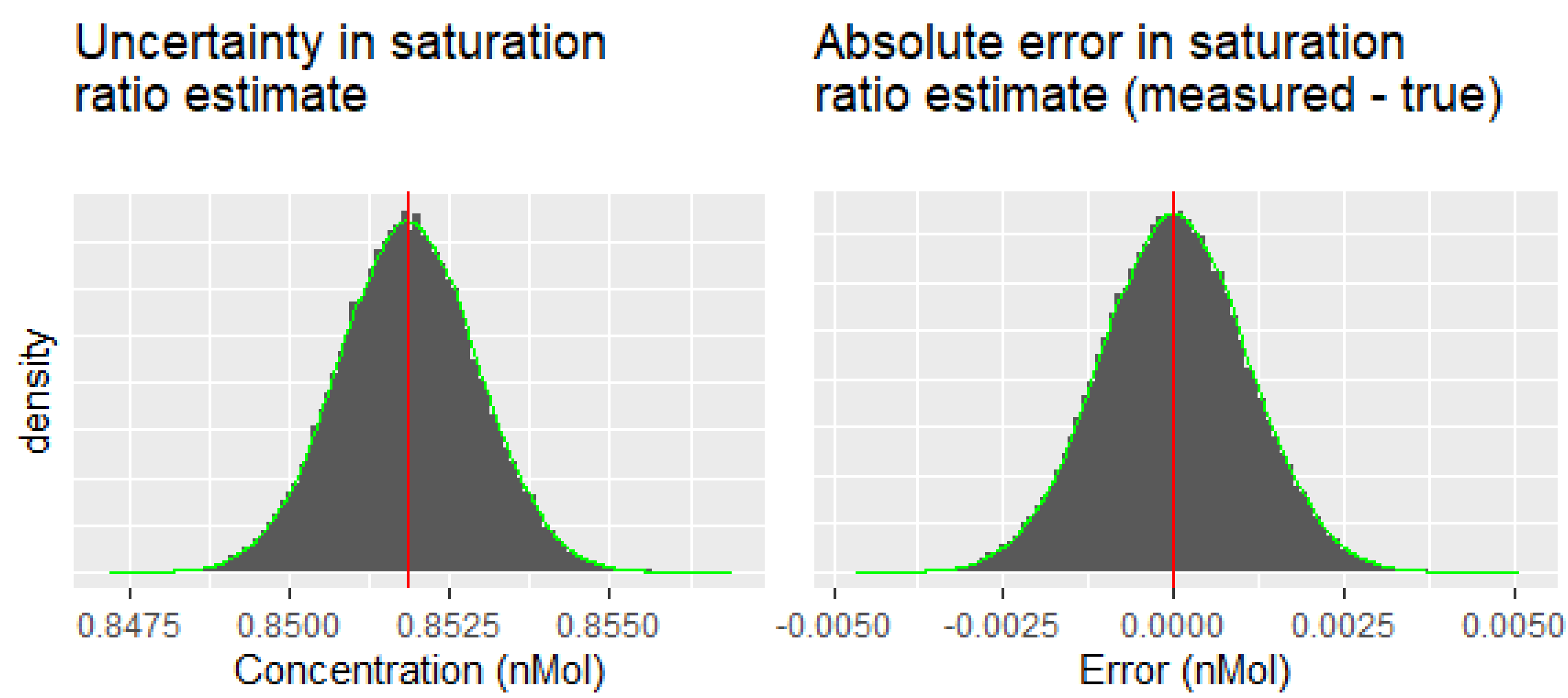


```
# standard deviation of a saturation ratio value s:
sd(S_mims_me) # 0.0011


## [1] 0.001077392


# the 95% CI is therefore the computed value +/- 2 standard deviations
sd(S_mims_me) * 1.96 # +/- 0.00216
```

```
## [1] 0.002111688
```

```
# so any ratio within 0.00216 of 1.0 cannot be discerned as a source or sink

# CV of sat
sd(S_mims_me) / mean(S_mims_me) * 100 # 0.13% CV
```

```
## [1] 0.1264766
```

```
# standard deviation of a measurement is:
round(sd(S_mims_me_abs), 3) # 0.20 nmol L-1
```

```
## [1] 0.001
```

```
# central 95th percentile of absolute error
round(quantile(S_mims_me_abs, probs = c(0.025, 0.975)), 3) # approx +/- 0.11
```

```
##   2.5%  97.5%
## -0.002  0.002
```

```
# the 95% CI for an observation with measurement error can be computed
# approximately as the value +/- 2 standard deviations; or the central 95th
# percentile of the absolute error distribution.
round(quantile((S_mims + S_mims_me_abs), probs = c(0.025, 0.975)), 3) # +/- nmol N
```

```
##  2.5% 97.5%
## 0.850 0.854
```

5.2.1.1.1 Source-sink status

```
# two standard deviations = 0.3019449
# 95% CI is therefore x+/-0.3019449

#dg <- dg %>%
```

```
#  mutate(n2o.src.snk.with.all.error = case_when(
#    abs(n2o.sat.ratio - 1) < sd(C_me_ratio) ~ "undetermined",
#    TRUE ~ n2o.src.snk))
dg <- dg %>%
  mutate(n2o.src.snk.with.all.error = case_when(
    abs(n2o.sat.ratio - 1) < quantile(S_mims_me_abs, probs = c(0.975)) ~ "undeterm
    TRUE ~ n2o.src.snk))

dg %>%
  filter(sample.source == "DG", visit.no == 1, sitetype == "PROB") %>%
  {table(.$n2o.src.snk.with.all.error)} %>%
  kable(digits = 2, caption = "Number of lakes classified as source/sink")
```

Var1	Freq
sink	650
source	322
undetermined	12

Number of lakes classified as source/sink

```
dg %>%
  filter(sample.source == "DG", visit.no == 1, sitetype == "PROB") %>%
  {prop.table(table(.$n2o.src.snk.with.all.error))} %>%
  kable(digits = 2, caption = "Proportion of lakes classified as source/sink")
```

Var1	Freq
sink	0.66
source	0.33
undetermined	0.01

Proportion of lakes classified as source/sink

5.2.1.2 High precision temperature

```
# ratio simulated with measurement error in mims
S_mims_hpt_me <- rep(NA, nsim)
```

```
for( i in 1:nsim){
S_mims_hpt_me[i] <- (R_X_theta_me[i] / R_X_s_me[i]) * # N2O:Ar ratios for sample a
  ((1e-6 * B_me[i] * M_a_n2o_me[i] * (H_n2o * exp(2700 * (1 / (T_c_mims_me[i] + 27
  (1e-6 * B_me[i] * C_Ar_a * (H_ar * exp(1500 * (1 / (T_c_mims_me[i] + 273.15) - 1
  (1e-6 * B_me[i] * C_Ar_a * (H_ar  * exp(1500 *(1/(T_c_mims_me[i] + 273.15) - 1/2
  (M_a_n2o_me[i] * B_me[i] * 1e-6 * (H_n2o  * exp(2700 * (1 / (T_c_mims_me[i] + 27
}


# combine into dataframe for ggplot.
S_mims_hpt_df <- tibble(S_mims_hpt_me = S_mims_hpt_me)
# save absolute error for plot
S_mims_hpt_me_abs <- S_mims_hpt_me - S_mims
```

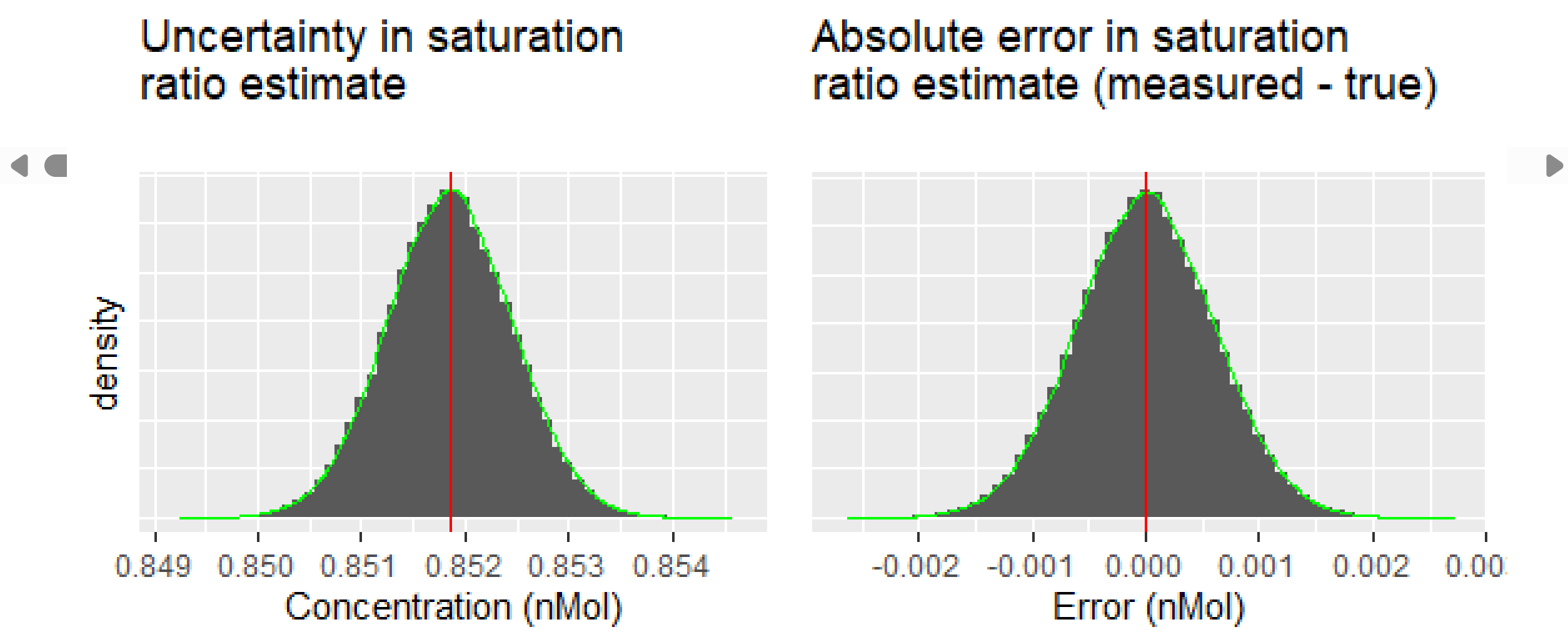


```
# standard deviation of a saturation ratio value s:
sd(S_mims_hpt_me) # 0.0011


## [1] 0.0006029935


# the 95% CI is therefore the computed value +/- 2 standard deviations
sd(S_mims_hpt_me) * 1.96 # +/- 0.00216


## [1] 0.001181867


# so any ratio within 0.00216 of 1.0 cannot be discerned as a source or sink
```

```r
# CV of sat
sd(S_mims_hpt_me) / mean(S_mims_hpt_me) * 100 # 0.13% CV
```

```
## [1] 0.07078621
```

```r
# standard deviation of a measurement is:
round(sd(S_mims_hpt_me_abs), 3) # 0.20 nmol L-1
```

```
## [1] 0.001
```

```r
# central 95th percentile of absolute error
round(quantile(S_mims_hpt_me_abs, probs = c(0.025, 0.975)), 3) # approx +/- 0.11
```

```
##   2.5%  97.5%
## -0.001  0.001
```

```r
# the 95% CI for an observation with measurement error can be computed
# approximately as the value +/- 2 standard deviations; or the central 95th
# percentile of the absolute error distribution.
round(quantile((S_mims + S_mims_hpt_me_abs), probs = c(0.025, 0.975)), 3) # +/- nm
```

```
##  2.5% 97.5%
## 0.851 0.853
```

5.2.1.2.1 Source-sink status

```r
# two standard deviations = 0.3019449
# 95% CI is therefore x+/-0.3019449

#dg <- dg %>%
#  mutate(n2o.src.snk.with.all.error = case_when(
#    abs(n2o.sat.ratio - 1) < sd(C_me_ratio) ~ "undetermined",
#    TRUE ~ n2o.src.snk))
dg <- dg %>%
  mutate(n2o.src.snk.with.all.error = case_when(
    abs(n2o.sat.ratio - 1) < quantile(S_mims_hpt_me_abs, probs = c(0.975)) ~ "unde
```

```
    TRUE ~ n2o.src.snk))

dg %>%
  filter(sample.source == "DG", visit.no == 1, sitetype == "PROB") %>%
  {table(.$n2o.src.snk.with.all.error)} %>%
  kable(digits = 2, caption = "Number of lakes classified as source/sink")
```

Var1	Freq
sink	654
source	323
undetermined	7

Number of lakes classified as source/sink

```
dg %>%
  filter(sample.source == "DG", visit.no == 1, sitetype == "PROB") %>%
  {prop.table(table(.$n2o.src.snk.with.all.error))} %>%
  kable(digits = 2, caption = "Proportion of lakes classified as source/sink")
```

Var1	Freq
sink	0.66
source	0.33
undetermined	0.01

Proportion of lakes classified as source/sink

5.2.2 High precision GC

5.2.2.1 Standard temperature

This simulation is for field measurment of temperature for the equilibrium concentration with the standard glass thermometer.

```
S_mims_hpgc_me <- rep(NA, nsim)

for( i in 1:nsim){
```

```
S_mims_hpgc_me[i] <- (R_X_theta_me[i] / R_X_s_me[i]) * # N2O:Ar ratios for sample
  ((1e-6 * B_me[i] * M_a_n2o_lit_me[i] * (H_n2o * exp(2700 * (1 / (T_c_mims_me[i]
  (1e-6 * B_me[i] * C_Ar_a * (H_ar * exp(1500 * (1 / (T_c_mims_me[i] + 273.15) - 1
  (1e-6 * B_me[i] * C_Ar_a * (H_ar  * exp(1500 *(1/(T_c_mims_me[i] + 273.15) - 1/2
  (M_a_n2o_lit_me[i] * B_me[i] * 1e-6 * (H_n2o  * exp(2700 * (1 / (T_c_me[i] + 273
}

# combine into dataframe for ggplot.
S_mims_hpgc_df <- tibble(S_mims_hpgc_me = S_mims_hpgc_me)
# save absolute error for plot
S_mims_hpgc_me_abs <- S_mims_hpgc_me - S_mims
```

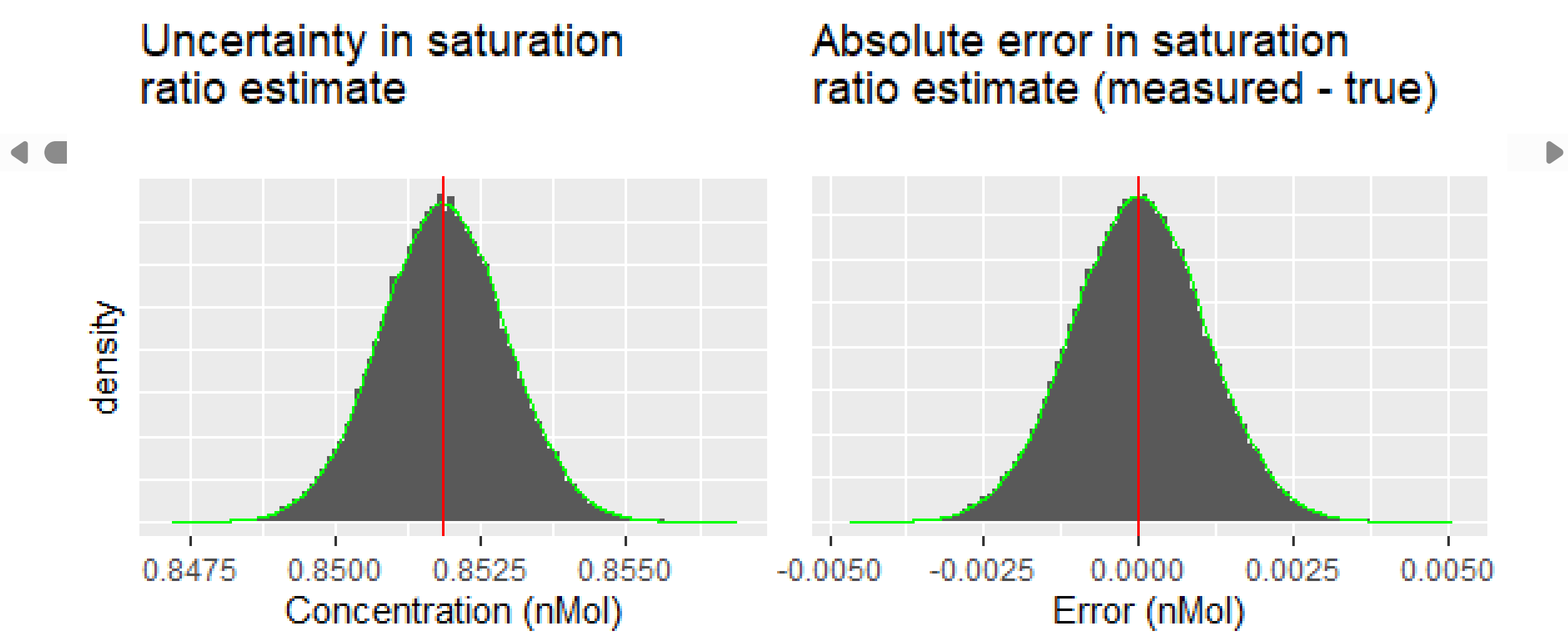


```
# standard deviation of a saturation ratio value s:
sd(S_mims_hpgc_me) # 0.0011
```

```
## [1] 0.001077392
```

```
# the 95% CI is therefore the computed value +/- 2 standard deviations
sd(S_mims_hpgc_me) * 1.96 # +/- 0.00216
```

```
## [1] 0.002111688
```

```
# so any ratio within 0.00216 of 1.0 cannot be discerned as a source or sink

# CV of sat
sd(S_mims_hpgc_me) / mean(S_mims_hpgc_me) * 100 # 0.07% CV
```

```
## [1] 0.1264766
```

```
# standard deviation of a measurement is:
round(sd(S_mims_hpgc_me_abs), 3) # 0.20 nmol L-1
```

```
## [1] 0.001
```

```
# central 95th percentile of absolute error
round(quantile(S_mims_hpgc_me_abs, probs = c(0.025, 0.975)), 3) # approx +/- 0.11
```

```
##   2.5%  97.5%
## -0.002  0.002
```

```
# the 95% CI for an observation with measurement error can be computed
# approximately as the value +/- 2 standard deviations; or the central 95th
# percentile of the absolute error distribution.
round(quantile((S_mims + S_mims_hpgc_me_abs), probs = c(0.025, 0.975)), 3) # +/- n
```

```
##  2.5% 97.5%
## 0.850 0.854
```

5.2.2.1.1 Source-sink status

```
# two standard deviations = 0.3019449
# 95% CI is therefore x+/-0.3019449

#dg <- dg %>%
#  mutate(n2o.src.snk.with.all.error = case_when(
#    abs(n2o.sat.ratio - 1) < sd(C_me_ratio) ~ "undetermined",
#    TRUE ~ n2o.src.snk))
dg <- dg %>%
  mutate(n2o.src.snk.with.all.error = case_when(
    abs(n2o.sat.ratio - 1) < quantile(S_mims_hpgc_me_abs, probs = c(0.975)) ~ "und
    TRUE ~ n2o.src.snk))

dg %>%
```

```
  filter(sample.source == "DG", visit.no == 1, sitetype == "PROB") %>%
  {table(.$n2o.src.snk.with.all.error)} %>%
  kable(digits = 2, caption = "Number of lakes classified as source/sink")
```

Var1	Freq
sink	650
source	322
undetermined	12

Number of lakes classified as source/sink

```
dg %>%
  filter(sample.source == "DG", visit.no == 1, sitetype == "PROB") %>%
  {prop.table(table(.$n2o.src.snk.with.all.error))} %>%
  kable(digits = 2, caption = "Proportion of lakes classified as source/sink")
```

Var1	Freq
sink	0.66
source	0.33
undetermined	0.01

Proportion of lakes classified as source/sink

5.2.2.2 High precision temperature

The thermometers used to measure water temperature in this study have an uncertainty of +/- 0.058C but electronic thermisters are commonly available (e.g. YSI sondes) with an uncertainty of +/-0.01 Celsius. More precise water temperature measurements will reduce uncertainty in the equilibrium N2O conentration and the saturation ratio.

```
S_mims_hpgc_hpt_me <- rep(NA, nsim)

for( i in 1:nsim){
S_mims_hpgc_hpt_me[i] <- (R_X_theta_me[i] / R_X_s_me[i]) * # N2O:Ar ratios for sam
  ((1e-6 * B_me[i] * M_a_n2o_lit_me[i] * (H_n2o * exp(2700 * (1 / (T_c_mims_me[i]
  (1e-6 * B_me[i] * C_Ar_a * (H_ar * exp(1500 * (1 / (T_c_mims_me[i] + 273.15) - 1
```

```
  (1e-6 * B_me[i] * C_Ar_a * (H_ar  * exp(1500 *(1/(T_c_mims_me[i] + 273.15) - 1/2
  (M_a_n2o_lit_me[i] * B_me[i] * 1e-6 * (H_n2o  * exp(2700 * (1 / (T_c_mims_me[i]
}


# combine into dataframe for ggplot.
S_mims_hpgc_hpt_df <- tibble(S_mims_hpgc_hpt_me = S_mims_hpgc_hpt_me)

# save absolute error for plot
S_mims_hpgc_hpt_me_abs <- S_mims_hpgc_hpt_me - S_mims
```

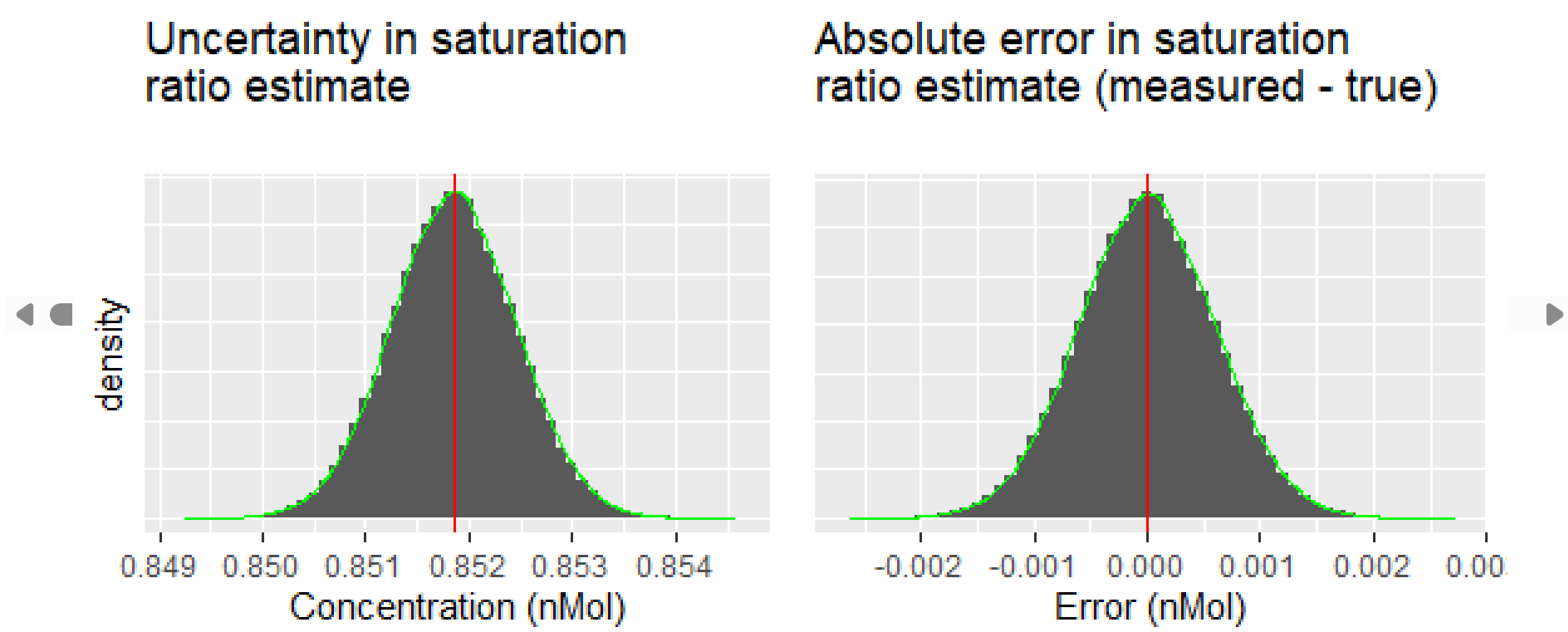


```
# standard deviation of a saturation ratio value s:
sd(S_mims_hpgc_hpt_me) # 0.0006603624
```

```
## [1] 0.0006029935
```

```
# the 95% CI is therefore the computed value +/- 2 standard deviations
sd(S_mims_hpgc_hpt_me) * 1.96 # +/- 0.00129431
```

```
## [1] 0.001181867
```

```
# so any ratio within 0.00129 of 1.0 cannot be discerned as a source or sink
```

```
# standard deviation of a measurement is:
round(sd(S_mims_hpgc_hpt_me_abs), 3) # 0.20 nmol L-1
```

```
## [1] 0.001
```

```
# central 95th percentile of absolute error
round(quantile(S_mims_hpgc_hpt_me_abs, probs = c(0.025, 0.975)), 3) # approx +/- 0
```

```
##   2.5%  97.5%
## -0.001  0.001
```

```
# the 95% CI for an observation with measurement error can be computed
# approximately as the value +/- 2 standard deviations; or the central 95th
# percentile of the absolute error distribution.
round(quantile((S_mims + S_mims_hpgc_hpt_me_abs), probs = c(0.025, 0.975)), 3) # +
```

```
##  2.5% 97.5%
## 0.851 0.853
```

5.2.2.2.1 Source-sink status

```
# two standard deviations = 0.3019449
# 95% CI is therefore x+/-0.3019449

#dg <- dg %>%
#  mutate(n2o.src.snk.with.all.error = case_when(
#    abs(n2o.sat.ratio - 1) < sd(C_me_ratio) ~ "undetermined",
#    TRUE ~ n2o.src.snk))
dg <- dg %>%
  mutate(n2o.src.snk.with.all.error = case_when(
    abs(n2o.sat.ratio - 1) < quantile(S_mims_hpgc_hpt_me_abs, probs = c(0.975)) ~
    TRUE ~ n2o.src.snk))

dg %>%
  filter(sample.source == "DG", visit.no == 1, sitetype == "PROB") %>%
  {table(.$n2o.src.snk.with.all.error)} %>%
  kable(digits = 2, caption = "Number of lakes classified as source/sink")
```

Var1	Freq
sink	654
source	323
undetermined	7

Number of lakes classified as source/sink

```
dg %>%
  filter(sample.source == "DG", visit.no == 1, sitetype == "PROB") %>%
  {prop.table(table(.$n2o.src.snk.with.all.error))} %>%
  kable(digits = 2, caption = "Proportion of lakes classified as source/sink")
```

Var1	Freq
sink	0.66
source	0.33
undetermined	0.01

Proportion of lakes classified as source/sink

6 Summary of ratio results

Precision of the N2O saturation ratio can be improved using high precision GC analytics and N2O-free headspace gas (e.g. helium, nitrogen) but the greatest improvement is seen when dissolved N2O is directly measured using MIMS.

```
#results <- tibble(ratio_sd = c(0.055,
#                                0.048,
#                                0.029,
#                                0.001207747,
#                                0.001207747,
#                                0.000690139),
#                   method = c("GC",
#                              "GC + helium",
#                              "High precision GC + helium",
#                              "MIMS",
```

```
#                             "MIMS + high precision GC",
#                             "MIMS + high precision GC + \nhigh precision water t


#results %>%
#  mutate(lower.95 = C_ratio - (1.96 * ratio_sd),
#         upper.95 = C_ratio + (1.96 * ratio_sd),
#         C_ratio = C_ratio) %>%
#  ggplot(aes(C_ratio, method)) +
#  geom_point() +
#  geom_segment(aes(x=lower.95, xend = upper.95, y=method, yend=method)) +
#  xlab("True saturation ratio (black dot) +/- measurement error (1.96 standard de
#  theme(axis.text.x = element_text(angle = 45, vjust = 0.9, hjust = 1))

results <- tibble(GC_me= S_a_me_abs,
                  GC_He_me = S_pg_me_abs,
                  GC_hpt_me = S_a_hpt_me_abs,
                  GC_He_hpt_me = S_pg_hpt_me_abs,
                  HPGC_He_me = S_pg_hpgc_me_abs,
                  HPGC_He_hpt_me = S_pg_hpgc_hpt_me_abs,
                  MIMS_me = S_mims_me_abs,
                  MIMS_hpt_me = S_mims_hpt_me_abs,
                  MIMS_HPGC_me = S_mims_hpgc_me_abs,
                  MIMS_HPGC_HPT_me = S_mims_hpgc_hpt_me_abs) %>%
  pivot_longer(cols = ends_with("me"), names_to = "Method", values_to = "Error")


results %>%
  mutate(Method = factor(Method)) %>%
  mutate(Method = fct_relevel(Method, c("MIMS_HPGC_HPT_me",
                                        "MIMS_HPGC_me",
                                        "MIMS_hpt_me",
                                        "MIMS_me",
                                        "HPGC_He_hpt_me",
                                        "HPGC_He_me",
                                        "GC_He_hpt_me",
                                        "GC_hpt_me",
                                        "GC_He_me",
                                        "GC_me"
                                        ))) %>%
  ggplot(aes(x = Error, y = Method)) +
  stat_pointinterval() +
  scale_y_discrete(labels=c("GC_me" = "Standard GC, ambient air, \n standard field
                            "GC_He_me" = "Standard GC, helium, \n standard field t
                            "GC_hpt_me" = "Standard GC, ambient air, \n high preci
                            "GC_He_hpt_me" = "Standard GC, helium, \nhigh precisio
                            "HPGC_He_me" = "High precision GC, helium, \nstandard
                            "HPGC_He_hpt_me" = "High precision GC, helium, \nhigh
```

```
                          "MIMS_me" = "MIMS: standard GC, \nstandard field tempe
                          "MIMS_hpt_me" = "MIMS: standard GC, \nhigh precision t
                          "MIMS_HPGC_me" = "MIMS: high precision GC, \nstandard 
                          "MIMS_HPGC_HPT_me" = "MIMS: high precision GC, \nhigh 
  xlab("Absolute error in saturation ratio") +
  ylab("")
```

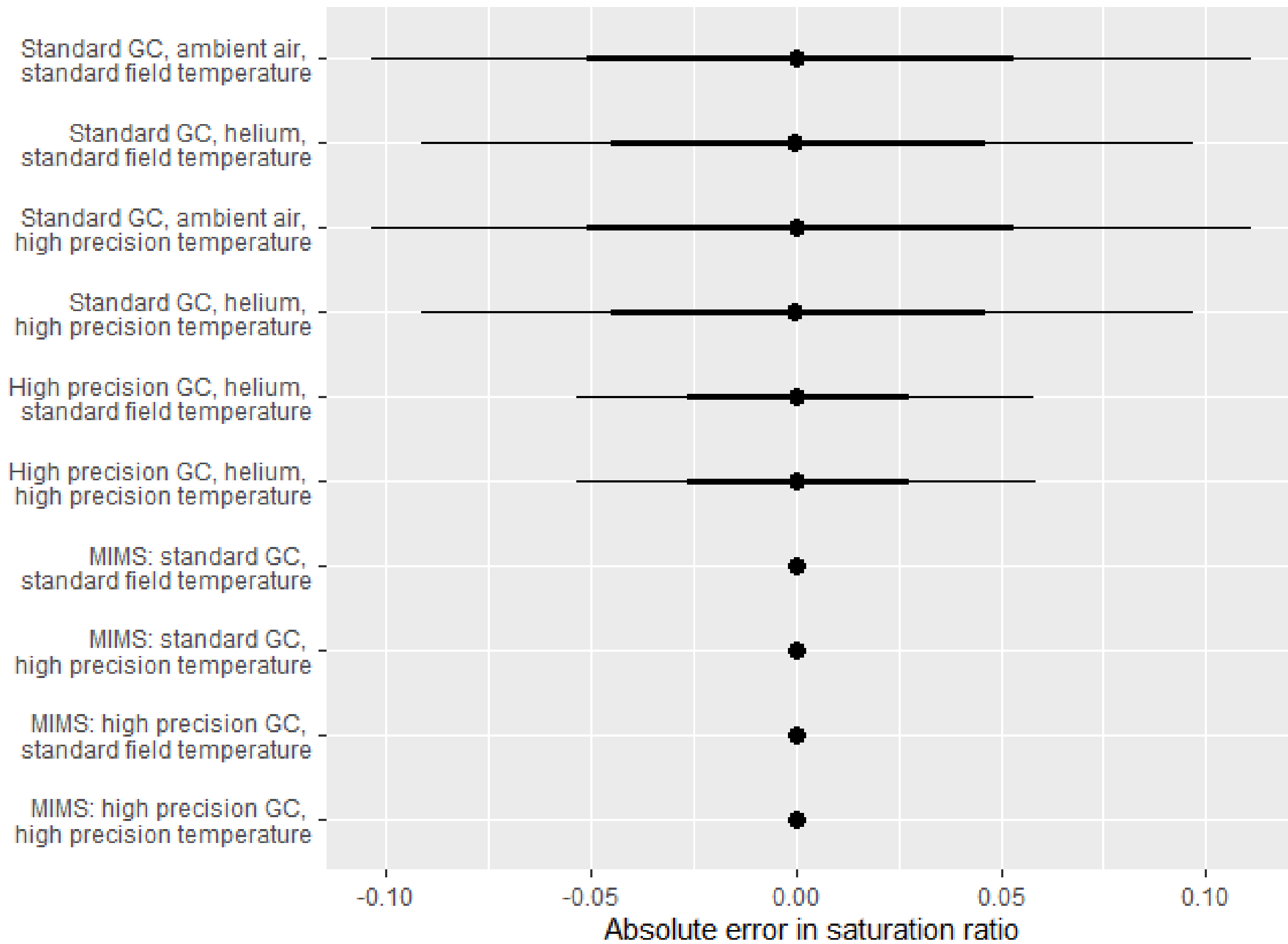

Because the MIMS method is so much more precise than the traditional method, the differences between the MIMS-based variations above are difficult to see. Below, just the MIMS-based variations are compared.

Precision of the N2O saturation ratio can be improved using high precision GC analytics and N2O-free headspace gas (e.g. helium, nitrogen) but the greatest improvement is seen when dissolved N2O is directly measured using MIMS.

```
results_MIMS <- tibble(MIMS_me = S_mims_me_abs,
                       MIMS_hpt_me = S_mims_hpt_me_abs,
                       MIMS_HPGC_me = S_mims_hpgc_me_abs,
                       MIMS_HPGC_HPT_me = S_mims_hpgc_hpt_me_abs) %>%
  pivot_longer(cols = ends_with("me"), names_to = "Method", values_to = "Error")
```

```
results_MIMS %>%
  mutate(Method = factor(Method)) %>%
  mutate(Method = fct_relevel(Method, c("MIMS_HPGC_HPT_me",
                                        "MIMS_HPGC_me",
                                        "MIMS_hpt_me",
                                        "MIMS_me"
                                        ))) %>%
  ggplot(aes(x = Error, y = Method)) +
  stat_pointinterval() +
  scale_y_discrete(labels=c("MIMS_me" = "MIMS: standard GC, \nstandard field tempe
                            "MIMS_hpt_me" = "MIMS: standard GC, \nhigh precision t
                            "MIMS_HPGC_me" = "MIMS: high precision GC, \nstandard
                            "MIMS_HPGC_HPT_me" = "MIMS: high precision GC, \nhigh
  xlab("Absolute error in saturation ratio") +
  ylab("")
```

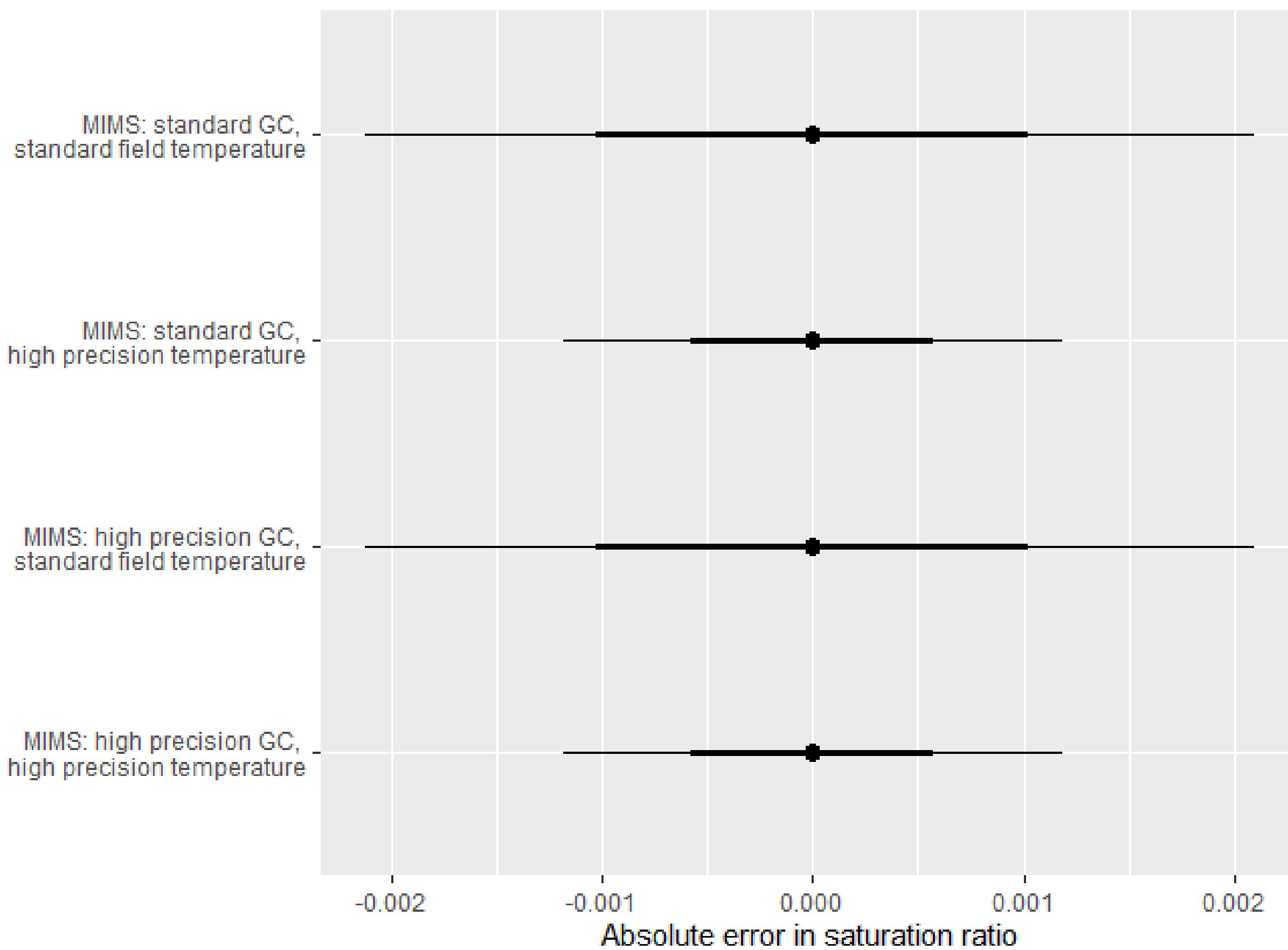

7 Update data with uncertainty info

Finally, we update the dg data frame to include information on estimated measurement error

```
dg <- dg %>%
  mutate(# recompute using error from uncertainty simulations
         n2o.src.snk.error = case_when(
           abs(n2o.sat.ratio - 1) < quantile(S_a_me_abs, probs = c(0.975)) ~ "unde
           TRUE ~ n2o.src.snk))

save(dg, file = "./../inputData/dg.RData")
```

8 Session Info

```
sessionInfo()
```

```
## R version 4.4.1 (2024-06-14 ucrt)
## Platform: x86_64-w64-mingw32/x64
## Running under: Windows 11 x64 (build 22631)
## 
## Matrix products: default
## 
## 
## locale:
## [1] LC_COLLATE=English_United States.utf8 
## [2] LC_CTYPE=English_United States.utf8 
## [3] LC_MONETARY=English_United States.utf8
## [4] LC_NUMERIC=C 
## [5] LC_TIME=English_United States.utf8 
## 
## time zone: America/New_York
## tzcode source: internal
## 
## attached base packages:
## [1] stats     graphics  grDevices utils     datasets  methods   base     
## 
## other attached packages:
##  [1] usmap_0.7.1      sf_1.0-19        janitor_2.2.1    readxl_1.4.4    
##  [5] gridExtra_2.3    ggExtra_0.10.1   tidybayes_3.0.7  lubridate_1.9.4 
##  [9] forcats_1.0.0    stringr_1.5.1    dplyr_1.1.4      purrr_1.0.4     
## [13] readr_2.1.5      tidyr_1.3.1      tibble_3.2.1     tidyverse_2.0.0 
## [17] ggpubr_0.6.0     ggplot2_3.5.1    kableExtra_1.4.0 bayesplot_1.11.1
```

```
## [21] rmarkdown_2.29   knitr_1.49
##
## loaded via a namespace (and not attached):
##  [1] DBI_1.2.3            rlang_1.1.5          magrittr_2.0.3
##  [4] snakecase_0.11.1     e1071_1.7-16         compiler_4.4.1
##  [7] systemfonts_1.2.1    vctrs_0.6.5          pkgconfig_2.0.3
## [10] arrayhelpers_1.1-0   fastmap_1.2.0        backports_1.5.0
## [13] labeling_0.4.3       utf8_1.2.4           promises_1.3.2
## [16] tzdb_0.4.0           xfun_0.51            later_1.4.1
## [19] broom_1.0.7          R6_2.6.1             stringi_1.8.4
## [22] car_3.1-3            cellranger_1.1.0     Rcpp_1.0.14
## [25] httpuv_1.6.15        timechange_0.3.0     tidyselect_1.2.1
## [28] rstudioapi_0.17.1    abind_1.4-8          yaml_2.3.10
## [31] miniUI_0.1.1.1       lattice_0.22-6       shiny_1.10.0
## [34] withr_3.0.2          posterior_1.6.1      coda_0.19-4.1
## [37] evaluate_1.0.3       units_0.8-5          proxy_0.4-27
## [40] ggdist_3.3.2         xml2_1.3.7           pillar_1.10.1
## [43] carData_3.0-5        tensorA_0.36.2.1     KernSmooth_2.23-22
## [46] checkmate_2.3.2      distributional_0.5.0 generics_0.1.3
## [49] hms_1.1.3            munsell_0.5.1        scales_1.3.0
## [52] xtable_1.8-4         class_7.3-22         glue_1.8.0
## [55] tools_4.4.1          ggsignif_0.6.4       cowplot_1.1.3
## [58] usmapdata_0.3.0      grid_4.4.1           colorspace_2.1-1
## [61] Formula_1.2-5        cli_3.6.4            svUnit_1.0.6
## [64] viridisLite_0.4.2    svglite_2.1.3        gtable_0.3.6
## [67] rstatix_0.7.2        digest_0.6.37        classInt_0.4-11
## [70] farver_2.1.2         htmltools_0.5.8.1    lifecycle_1.0.4
## [73] mime_0.12
```
