# Supplementary material for: Pervasive summertime nitrous oxide undersaturation in U.S. lakes and reservoirs
Source: Nat Commun. 2026 Jul 11;17:8551. doi: 10.1038/s41467-026-74705-6 (PMC13482806; doi:10.1038/s41467-026-74705-6)
Supplement: Supplementary file 2 [file 41467_2026_74705_MOESM2_ESM.pdf]

nature portfolio

Corresponding author(s): Jake Beaulieu

Last updated by author(s): Apr 27, 2026

Reporting Summary

Nature Portfolio wishes to improve the reproducibility of the work that we publish. This form provides structure for consistency and transparency in reporting. For further information on Nature Portfolio policies, see our Editorial Policies and the Editorial Policy Checklist.

Statistics

For all statistical analyses, confirm that the following items are present in the figure legend, table legend, main text, or Methods section.

n/a	Confirmed	
☐	☒	The exact sample size (n) for each experimental group/condition, given as a discrete number and unit of measurement
☐	☒	A statement on whether measurements were taken from distinct samples or whether the same sample was measured repeatedly
☐	☒	The statistical test(s) used AND whether they are one- or two-sided<br>*Only common tests should be described solely by name; describe more complex techniques in the Methods section.*
☐	☒	A description of all covariates tested
☐	☒	A description of any assumptions or corrections, such as tests of normality and adjustment for multiple comparisons
☐	☒	A full description of the statistical parameters including central tendency (e.g. means) or other basic estimates (e.g. regression coefficient) AND variation (e.g. standard deviation) or associated estimates of uncertainty (e.g. confidence intervals)
☒	☐	For null hypothesis testing, the test statistic (e.g. F, t, r) with confidence intervals, effect sizes, degrees of freedom and P value noted<br>*Give P values as exact values whenever suitable.*
☐	☒	For Bayesian analysis, information on the choice of priors and Markov chain Monte Carlo settings
☐	☒	For hierarchical and complex designs, identification of the appropriate level for tests and full reporting of outcomes
☒	☐	Estimates of effect sizes (e.g. Cohen's d, Pearson's r), indicating how they were calculated

Our web collection on statistics for biologists contains articles on many of the points above.

Software and code

Policy information about availability of computer code

Data collection	no software used to collect the data
Data analysis	R version 4.4.0

For manuscripts utilizing custom algorithms or software that are central to the research but not yet described in published literature, software must be made available to editors and reviewers. We strongly encourage code deposition in a community repository (e.g. GitHub). See the Nature Portfolio guidelines for submitting code & software for further information.

Data

Policy information about availability of data

All manuscripts must include a data availability statement. This statement should provide the following information, where applicable:

- Accession codes, unique identifiers, or web links for publicly available datasets
- A description of any restrictions on data availability
- For clinical datasets or third party data, please ensure that the statement adheres to our policy

Provide your data availability statement here.

Research involving human participants, their data, or biological material

Policy information about studies with human participants or human data. See also policy information about sex, gender (identity/presentation), and sexual orientation and race, ethnicity and racism.

Reporting on sex and gender	*Use the terms sex (biological attribute) and gender (shaped by social and cultural circumstances) carefully in order to avoid confusing both terms. Indicate if findings apply to only one sex or gender; describe whether sex and gender were considered in study design; whether sex and/or gender was determined based on self-reporting or assigned and methods used. Provide in the source data disaggregated sex and gender data, where this information has been collected, and if consent has been obtained for sharing of individual-level data; provide overall numbers in this Reporting Summary. Please state if this information has not been collected. Report sex- and gender-based analyses where performed, justify reasons for lack of sex- and gender-based analysis.*
Reporting on race, ethnicity, or other socially relevant groupings	*Please specify the socially constructed or socially relevant categorization variable(s) used in your manuscript and explain why they were used. Please note that such variables should not be used as proxies for other socially constructed/relevant variables (for example, race or ethnicity should not be used as a proxy for socioeconomic status). Provide clear definitions of the relevant terms used, how they were provided (by the participants/respondents, the researchers, or third parties), and the method(s) used to classify people into the different categories (e.g. self-report, census or administrative data, social media data, etc.) Please provide details about how you controlled for confounding variables in your analyses.*
Population characteristics	*Describe the covariate-relevant population characteristics of the human research participants (e.g. age, genotypic information, past and current diagnosis and treatment categories). If you filled out the behavioural & social sciences study design questions and have nothing to add here, write "See above."*
Recruitment	*Describe how participants were recruited. Outline any potential self-selection bias or other biases that may be present and how these are likely to impact results.*
Ethics oversight	*Identify the organization(s) that approved the study protocol.*

Note that full information on the approval of the study protocol must also be provided in the manuscript.

Field-specific reporting

Please select the one below that is the best fit for your research. If you are not sure, read the appropriate sections before making your selection.

☐ Life sciences ☐ Behavioural & social sciences ☒ Ecological, evolutionary & environmental sciences

For a reference copy of the document with all sections, see nature.com/documents/nr-reporting-summary-flat.pdf

Ecological, evolutionary & environmental sciences study design

All studies must disclose on these points even when the disclosure is negative.

Study description	A probabilistic survey of dissolved N2O concentration is US waterbodies.
Research sample	A probabilistic sample of US waterbodies defined as pond, natural lake or reservoir at least 2.47 acres (1 hectare) in area, at least 3.3 feet (1 meter) deep and with at least a quarter acre (0.1 hectare) of open water. In addition, lakes were required to have a lake water minimum residence time of one week. The Great Lakes and the Great Salt Lake were not included in the survey, nor were commercial treatment and/or disposal ponds, tidally influenced lakes or ephemeral lakes.
Sampling strategy	The sample was selected using a Generalized Random Tessellation Stratified (GRTS) survey design. The sample size was chosen to provide full coverage of US waterbodies and to allow for analysis of waterbody condition across ecrogeions, waterbody size, state, as well as detect temporal trends in waterboy conditions across repeated surveys. Minimum sample sizes required to meet these objectives were based on a detailed analysis of the signal to noise ratio for each waterbody condition indicator.
Data collection	This national-scale study entailed data collection efforts by numerous federal, state, tribal, and private entities. Data collection was overseen by the USEPA under a Quality Assurance Project Plan.
Timing and spatial scale	Samples were collected from one location within each waterbody between 02 May 2017 and 02 October 2017. 984 lakes were sampled on a single date and 95 were re-visited for a second sample.
Data exclusions	No data were excluded from the analysis.
Reproducibility	Reproducibility was assessed by training the model using data from the first site visit, then using the model to predict the values observed during the second site visit. Coefficient of determination between predicted and observed was 0.61 and this uncertainty is reflected in the credible intervals reported in the paper.
Randomization	The survey design was stratified by US State with unequal probability category for waterbody size.

Blinding	The Investigators were not blinded during sample collection. Blinding was not possible as Investigators were aware of the location ans size of the waterbody during sampling. Blinding is not relevant for national-scale surveys were analysis are performed using computational algorithms.

Did the study involve field work? ☒ Yes ☐ No

Field work, collection and transport

Field conditions	Sampling was conducted during the US summer season and included a broad range of air temperatures, wind speed, and solar irradiance.
Location	Exact sampling location in each of the 984 waterboides is available in the data repository. See Data Availability section.
Access & import/export	Waterbodies were accessed via boat ramps when available, else accessed from shore. Landowner permission was secured when needed, if permission was denied, the waterbody was replaced with another from an oversample site generated via the survey design.
Disturbance	Waterbodies were not disturbed by our sampling methods.

Reporting for specific materials, systems and methods

We require information from authors about some types of materials, experimental systems and methods used in many studies. Here, indicate whether each material, system or method listed is relevant to your study. If you are not sure if a list item applies to your research, read the appropriate section before selecting a response.

Materials & experimental systems

n/a	Involved in the study
☒	☐ Antibodies
☒	☐ Eukaryotic cell lines
☒	☐ Palaeontology and archaeology
☒	☐ Animals and other organisms
☒	☐ Clinical data
☒	☐ Dual use research of concern
☒	☐ Plants

Methods

n/a	Involved in the study
☒	☐ ChIP-seq
☒	☐ Flow cytometry
☒	☐ MRI-based neuroimaging

Plants

Seed stocks	*Report on the source of all seed stocks or other plant material used. If applicable, state the seed stock centre and catalogue number. If plant specimens were collected from the field, describe the collection location, date and sampling procedures.*
Novel plant genotypes	*Describe the methods by which all novel plant genotypes were produced. This includes those generated by transgenic approaches, gene editing, chemical/radiation-based mutagenesis and hybridization. For transgenic lines, describe the transformation method, the number of independent lines analyzed and the generation upon which experiments were performed. For gene-edited lines, describe the editor used, the endogenous sequence targeted for editing, the targeting guide RNA sequence (if applicable) and how the editor was applied.*
Authentication	*Describe any authentication procedures for each seed stock used or novel genotype generated. Describe any experiments used to assess the effect of a mutation and, where applicable, how potential secondary effects (e.g. second site T-DNA insertions, mosiacism, off-target gene editing) were examined.*
