# Supplementary material for: Pervasive summertime nitrous oxide undersaturation in U.S. lakes and reservoirs
Source: Nat Commun. 2026 Jul 11;17:8551. doi: 10.1038/s41467-026-74705-6 (PMC13482806; doi:10.1038/s41467-026-74705-6)
Supplement: Supplementary file 3 [file 41467_2026_74705_MOESM3_ESM.pdf]

nature portfolio

Peer Review File

Pervasive summertime nitrous oxide undersaturation in U.S. lakes and reservoirs

Corresponding Author: Dr Jake Beaulieu

This file contains all reviewer reports in order by version, followed by all author rebuttals in order by version.

Version 0:

Reviewer comments:

Reviewer #1

(Remarks to the Author)
This analysis explores the prevalence of N2O undersaturation in lakes and reservoirs. It leverages a very large dataset of pN2O measurements from almost 1000 lakes (part of the National Lake Assessment) and a model that uses these data to predict N2O concentrations in over 450,000 lakes across the continental US. These numbers are impressive and add to the validity of the results. I find the analysis generally convincing (except for one main issue described below), and the results further confirm recent studies that show that N2O undersaturation is relatively common in freshwaters (e.g., Aho et al., 2023; Soued et al., 2016; Webb et al., 2019), despite the prevailing assumption in the literature that freshwaters only act as N2O sources.

My main comment is that the dataset (although large!) is based on summer sampling in 2017. I think that manuscript should be reframed to reflect that this analysis is based on summer data only. Without a better representation of seasonal/annual dynamics, the analysis is only relevant to summer N2O concentrations and emissions. Therefore, I recommend reframing the manuscript to address this sampling limitation, rather than save it for a paragraph at the end of the discussion. In other words, this analysis is exciting, but it’s only for one season (summer) and so the conclusions should be presented in this context, rather than as an assessment of lentic N2O emissions more broadly/conclusively.

Other comments.

Title:
The title should be modified to reflect “summer” undersaturation

Abstract:
L10-12 “Here we use a new national-scale dataset of dissolved N2O *concentrations* and a Bayesian hierarchical model”
L13-14 “We found that N2O undersaturation was pervasive throughout the CONUS *in summer 2017*, with an estimated 72.9% (95% credible interval: 68.9 - 76.6%) of lakes functioning as N2O sinks.”
L21-24 Beyond the uncertainty in the analysis, the summer-only timeframe would also preclude determination of net source vs net sink behavior.

Introduction:
L26-28 If possible, mention that N2O is a greenhouse gas with high global warming potential. Or perhaps a “trace gas” with “radiative forcing potential”, if needed.
L31-33 I generally agree with this comment but it overlooks that there have been efforts in this direction. E.g., the Wang et al. 2023 model allows for N2O uptake in a lake/reservoir emission estimates and Aho et al. 2023 includes outputs from a global model that shows ~40% of freshwater samples are undersaturated in N2O (which is very similar to your result).
L34-35 considering adding a couple of citations

Results/discussion:
Body text narrative should also be revised to reflect the “summer snapshot” nature of the data and analysis
L74 The link to SI_2 in the pdf doesn’t work for me but I was able to access through the main Github
L74-75 “we can confidently report that a minimum of 40 percent of the sampled lakes were functioning as sinks *in summer 2017*”

Figure 3. “Estimated interactive effects of surface water temperature (A and B) and waterbody size (*C* and D)”
Figure 3. What does subplot B tell us that subplot A doesn’t? Same question for subplot D compared to subplot C.

Methods:
L479 Was each lake sampled once? This detail is probably described in the NLA docs, but would be good to include here too.

(Remarks on code availability)

Reviewer #2

(Remarks to the Author)

Key results
The study by Beaulieu et al. reports Pervasive nitrous oxide undersaturation in U.S. lakes and reservoirs based on N2O concentration measurements in 984 water bodies across the United States. The dataset consists of 1.7 times the number of water bodies included previous national or regional surveys in different continents (North America, Europe) and therefore gives strong support to earlier findings of widespread summertime N2O undersaturation in water bodies. Notably, special attention was given to sample large lakes, which have remained understudied but contribute to 80% of the surface area of the water bodies in the US. The elegant spatial study design allows the authors to upscale results to 465,896 waterbodies in the conterminous U.S. (CONUS), using Bayesian approach. Despite the large sampling effort, the upscaled estimates of N2O emissions are poorly constrained. It is too uncertain to determine if CONUS lakes are a net summertime source or sink of N2O, which the authors discuss and consider in the context of previous measurement and modelling results. I appreciate this detailed uncertainty analysis. However, one of the largest uncertainties of the study lies in estimating annual emissions based on single samples collected from 271 individual lakes during summertime only.

Validity
For the N2O concentrations and their drivers in the set of sampled lakes, the data interpretation is sound, and the conclusion are valid and robust. Also the Bayesian modelling seems a useful approach to illustrate the uncertainties in upscaling. My concern is that the annual N2O estimates based on single summertime measurement may not be valid or robust enough to be included.

Significance
The variation in N2O in any ecosystems has remained difficult to explain, model and predict. This large dataset allows to analyze main drivers of N2O concentrations in waterbodies: pNO3, temperature and lake surface area, and their interaction in various U.S. ecoregions comprehensively. Several explanations of the observed patterns and possible underlying mechanisms are discussed in detail. This is a significant step forward in the field. The upscaling approach should also be transferable to other fields of study.

Data and methodology
In general, the data and methods are valid and presented in sufficient detail in the main part of the manuscript. More information is needed how lake–atmosphere gas exchange: N2O efflux and influx, were calculated based on N2O concentration data. Please clarify whether the fluxes were calculated for each study lake or for certain lake categories at an ecoregion level. Were gas transfer coefficients (k values) or wind speeds measured at the time of the sampling at each lake or were published k values used? How was the lake surface area considered in estimation of wind speed for study lakes (if wind speed was not measured)? How were wind speeds modelled for POI to estimate flux? Wind speed is discussed on L192, but it is unclear how wind speed is considered in calculations. Please describe these steps as wind speed will affect the evasion differently in lakes of different size (large lakes with long fetches and small lakes L193-195).
In general, the quality of Figures is good. Figure 1. is not accessible, it is difficult to distinguish red N2O sink circles because of lack of contrast to colors for ecoregions, particularly Western Mountains. Please consider e.g. white dots/ lighter colors for ecoregions.

Analytical approach
Upscaled estimates for pN2O are based on lake and sampling characteristics: *Survey*, *Area*, *Elev*, Lat, Day. The sole geographical variables were not good predictors but produced biased distribution of N2O. The POI lakes had no records of key drivers of pN2O: water chemistry, e.g. pNO3, or temperature, if I have understood correctly. Therefore, a complicated approach with intermediate variables: predicted pNO3 and Temperature derived from *Survey*, *Area* Temp and *Survey*, *Lat*, *Elev*, *Day*, respectively, was needed to estimate pN2O in POI waterbodies.
This makes the reader wonder what the added value of this approach is compared to simply presenting median concentrations and fluxes multiplied by the area of respective lake size class in that ecoregion and for the CONUS. How much is the uncertainly reduced or estimates’ accuracy improved when using Bayesian approach? This should also be discussed briefly when comparing with previous models and measurements.

Suggested improvements
As described above, the annual estimates based on single summertime sampling are highly uncertain extrapolations, which the authors correctly mention. A study on 112 seasonally ice-covered lakes (Kortelainen et al. 2020) showed that including winter data resulted in fourfold annual N2O emission estimates compared to summer only measurements. Whereas three

subtropical reservoirs had highest emissions in fall (Musenze et al. 2014), and a temperate river in summer (Beaulieu et al. 2010). This literature (cited in ms) indicates that the annual estimates from the 9 different ecoregions from Coastal plains to Northern mountains are likely be differently affected by the lack of spring, fall and winter measurements. Allocation the sampling effort across the four seasons could have reduced the uncertainty in annual estimates and improved the study. I suggest removing any annual estimates and focusing on results from summertime, for which there are measured data.

Clarity and context, References
This manuscript reads very well, and I also appreciate the balanced use of literature.

(Remarks on code availability)
I have read the SI 1 to review the steps taken in the modelling and the justifications for different assumptions and choices of variables, but the assessment of the details or running of actual code is outside the scope of my expertise.
SI 2 was not accessible without login credentials.

Version 1:

Reviewer comments:

Reviewer #1

(Remarks to the Author)
I reviewed a previous version of this manuscript. The authors responded to all of my previous comments adequately. The current version of the manuscript well-written, convincing, and interesting. I think that it will be an important contribution to understanding the role of inland waters in N2O budgets.

(Remarks on code availability)

Reviewer #2

(Remarks to the Author)
I appreciate the authors' efforts in clarifying the manuscript and can conclude that my suggestions for improvements are now addressed adequately.

(Remarks on code availability)

Open Access This Peer Review File is licensed under a Creative Commons Attribution 4.0 International License, which permits use, sharing, adaptation, distribution and reproduction in any medium or format, as long as you give appropriate credit to the original author(s) and the source, provide a link to the Creative Commons license, and indicate if changes were made.

In cases where reviewers are anonymous, credit should be given to 'Anonymous Referee' and the source.
The images or other third party material in this Peer Review File are included in the article's Creative Commons license, unless indicated otherwise in a credit line to the material. If material is not included in the article's Creative Commons license and your intended use is not permitted by statutory regulation or exceeds the permitted use, you will need to obtain permission directly from the copyright holder.
To view a copy of this license, visit https://creativecommons.org/licenses/by/4.0/

Reviewer	Original line #	Revised line #	Comment	Response
#1	General comment / Results and Discussion		I think that manuscript should be reframed to reflect that this analysis is based on summer data only.	• We revised the title to specify that our results apply to the summer season.<br>• We now specify "summer" each time we refer to a result (e.g. "summertime N_2O concentration", "…N_2O sinks during the summer months"). This is done in <u>**23 separate locations**</u> throughout the manuscript.<br>• We moved and expanded our discussion of seasonality (new line numbers 240 – 246, 268 – 269).
#1	title		The title should be modified to reflect "summer" undersaturation	Changed to: "Pervasive summertime nitrous oxide undersaturation in U.S. lakes and reservoirs"
#1	10-12	10-12	Here we use a new national-scale dataset of dissolved N2O *concentrations* and a Bayesian hierarchical model	Done
#1	13-14	13-14	We found that N2O undersaturation was pervasive throughout the CONUS *in summer 2017*, with an estimated 72.9% (95% credible interval: 68.9 - 76.6%) of lakes functioning as N2O sinks."	Done

#1	21-24	20-22	Beyond the uncertainty in the analysis, the summer-only timeframe would also preclude determination of net source vs net sink behavior	Changed to: Pervasive N_2O undersaturation in CONUS waterbodies during the summer highlights the need to revisit N_2O models which presume surface waters are a N_2O source.
#1	26-28	27-28	If possible, mention that N2O is a greenhouse gas with high global warming potential. Or perhaps a “trace gas” with “radiative forcing potential”, if needed	Added “…and has a radiative forcing potential 273 times that of carbon dioxide.”
#1	31-33	29-32	I generally agree with this comment but it overlooks that there have been efforts in this direction. E.g., the Wang et al. 2023 model allows for N2O uptake in a lake/reservoir emission estimates and Aho et al. 2023 includes outputs from a global model that shows ~40% of freshwater samples are undersaturated in N2O (which is very similar to your result).	Added a mention of the Aho et al estimate. We withhold mention of Wang et al until lines 267-273 where we can give it a full treatment.
#1	34-35	37-38	considering adding a couple of citations	Added a reference to a recent review article: Pan, H., Z. Zhou, S. Zhang, F. Wang and J. Wei (2023). "N2O Emissions from Aquatic Ecosystems: A Review." Atmosphere **14**(8): 1291.

#1	74	77	link to SI_2 in the pdf doesn't work for me but I was able to access through the main Github	Done
#1	74-75	75-78	we can confidently report that a minimum of 40 percent of the sampled lakes were functioning as sinks *in summer 2017*	This section was slightly reworked and we now reference the first and second site visits, both of which occurred during the summer. The first sentence on the subsequent paragraph, which discusses the same result, specifies "…functioned as net N2O sinks during the summer months…"
#1	Figure 3		"Estimated interactive effects of surface water temperature (A and B) and waterbody size (*C* and D)"	Done
#1	Figure 3		What does subplot B tell us that subplot A doesn't? Same question for subplot D compared to subplot C.	Each pair of plots (A/B, C/D) convey the same information, but emphasize different aspects of the interactions. Panel A emphasizes that the N2O ~ temperature relationship is conditional upon nitrate concentration. Panel B emphasizes that the N2O ~ NO3 relationship is conditional on temperature. Similar rationale for plots C and D.

				We believe that all 4 panels are required to communicate these complicated interactions.
#1	479	491-492	Was each lake sampled once? This detail is probably described in the NLA docs, but would be good to include here too.	Now specified in final sentence of Survey Design section.
# 2	general		However, one of the largest uncertainties of the study lies in estimating annual emissions based on single samples collected from 271 individual lakes during summertime only.	We rescaled the flux estimates to the summer months. No annual estimates are presented. See lines 214-215, 252-257, and 258-260, for an expanded discussion of seasonality and rationale for not upscaling to the full calendar year.
# 2	general		My concern is that the annual N2O estimates based on single summertime measurement may not be valid or robust enough to be included.	See above
# 2	193-195	601-615	More information is needed how lake–atmosphere gas exchange: N2O efflux and influx, were calculated based on N2O concentration data. Please clarify whether the fluxes were calculated for each study lake or for certain lake categories at an ecoregion level. Were gas transfer coefficients (k values) or wind speeds measured	Thank you for pointing out this important oversight. This information is now presented in the new "Emission estimates and upscaling" subsection of the "Methods".

			at the time of the sampling at each lake or were published k values used? How was the lake surface area considered in estimation of wind speed for study lakes (if wind speed was not measured)? How were wind speeds modelled for POI to estimate flux? Wind speed is discussed on L192, but it is unclear how wind speed is considered in calculations. Please describe these steps as wind speed will affect the evasion differently in lakes of different size (large lakes with long fetches and small lakes L193-195).	
# 2	Figure 1		it is difficult to distinguish red N2O sink circles because of lack of contrast to colors for ecoregions, particularly Western Mountains. Please consider e.g. white dots/ lighter colors for ecoregions.	Figure has been recolored.
# 2	Analytical approach	538-545	How much is the uncertainly reduced or estimates' accuracy improved when using Bayesian approach? This should also be discussed briefly when comparing with previous models and measurements.	• The purpose of the Bayesian approach isn't directly to improve precision or accuracy, per se, but to more clearly represent uncertainties. The Bayesian approach allowed us to explicitly carry uncertainties forward from our regression modeling into our derived emission and flux estimates.

				There is no straightforward way to do this in a frequentist setting (538-540).<br><br>• As for “comparing with previous models and measurements” please see the expanded “Statistical Inference” section (lines 540-545) of the manuscript describing a comparison of our Bayesian estimates to that derived from more traditional, frequentist-based estimates (full details of both approaches are presented in the SI_1). We demonstrate that the Bayesian results are more precise and shrunk towards the grand mean (as is expected based on cited literature and references), relative to the traditional modeling approach.
# 2	Suggested improvements		I suggest removing any annual estimates and focusing on results from summertime, for which there are measured data.	Done
# 2			SI 2 was not accessible without login credentials.	Apologies for the broken link. This has been fixed.
